# Amino acid torsion angles enable prediction of protein fold classification

**DOI:** 10.1038/s41598-020-78465-1

**Published:** 2020-12-10

**Authors:** Kun Tian, Xin Zhao, Xiaogeng Wan, Stephen S.-T. Yau

**Affiliations:** 1 School of Mathematics, Renmin University of China, Beijing, 100872 People’s Republic of China; 2 Department of Cryptography and Technology, Beijing Electronic Science and Technology Institute, Beijing, 100070 People’s Republic of China; 3 Department of Mathematical Sciences, Tsinghua University, Beijing, 100084 People’s Republic of China

**Keywords:** Computational biology and bioinformatics, Structural biology

## Abstract

Protein structure can provide insights that help biologists to predict and understand protein functions and interactions. However, the number of known protein structures has not kept pace with the number of protein sequences determined by high-throughput sequencing. Current techniques used to determine the structure of proteins are complex and require a lot of time to analyze the experimental results, especially for large protein molecules. The limitations of these methods have motivated us to create a new approach for protein structure prediction. Here we describe a new approach to predict of protein structures and structure classes from amino acid sequences. Our prediction model performs well in comparison with previous methods when applied to the structural classification of two CATH datasets with more than 5000 protein domains. The average accuracy is 92.5% for structure classification, which is higher than that of previous research. We also used our model to predict four known protein structures with a single amino acid sequence, while many other existing methods could only obtain one possible structure for a given sequence. The results show that our method provides a new effective and reliable tool for protein structure prediction research.

## Introduction

The resolution of protein three-dimensional structure is one of the most important research problems in the field of structural biology. The structure of a protein is directly related to its function, and structural prediction is an important goal of bioinformatics and theoretical chemistry, with great potential benefits in the fields of medicine and biotechnology. Hence, how to predict three-dimensional structures from protein sequences has been an unsolved and significant problem. Although amino acid sequences determine protein structures, other factors also contribute to structural modification, which demands us find an efficient technique to delineate the global properties of protein structure space^1–4^. Current techniques for the determination of protein structures include X-ray crystallography, nuclear-magnetic-resonance (NMR) spectroscopy and so on. With modern new techniques, such as machine learning methods, a lot of new approaches appear in protein structure prediction work^5–19^. For example, Chou et al. develop methods to predict protein structural classes^8,9^. Brevern et al. define a structural alphabet, which allows the local approximation of the 3D protein structure by using a Bayesian approach based on the relation of protein block amino acid propensity^11^. Wood et al. provide a method called DESTRUCT using a sequence and structure representation and an iterative prediction algorithm^12^. Jung et al. have created a web server providing structural information and analysis based on the backbone torsional representation of a protein structure^13^. Wei et al. introduce the use of protein topological features captured by persistent homology for protein classification^14^. More and more software tools have appeared recently, including structure prediction, protein threading, homology modeling, and so on. For example, RaptorX^20^ is a web server predicting structure using a deep learning model. I-TASSER^21^ could also be used for protein structure prediction, while it is based on the profile–profile threading alignment. HHpred^22^ is a server for homology modeling and structure prediction. However, these methods often require time-consuming analysis of experimental results, especially for large protein molecules which make them unreliable and ineffective for structure prediction. Thus, the speed of computation and accuracy still have room for improvement. A fundamental theorem in protein science indicates that a protein sequence can completely determine the 3D structure. The unique structure, which is at the lowest free energy, shall be predicted from the sequence. The multiple forms of the structure are the results of biochemical environments, for example, binding to ions, DNA, small molecules, or being at different PH. Here we focus on predicting multiple different structures for one protein sequence. Many existing methods may have limitations and drawbacks for predicting multiple structures of sequence since these tools only obtain the most likely possible structure for each sequence. Therefore, it is necessary to develop a more accurate, fast and effective method to delineate the relationship between sequence code and structure space.

Here, we have therefore attempted to develop a methodology that uses primary amino acid sequence information to make a precise and effective prediction of the possible structures for a particular protein and to visualize the comparison between the native structure and the predicted structure. Our method is based on the integration and analysis of torsion angle information from the Protein Data Bank (PDB) database, which contains information from over 10 million torsion angles. By taking into account the torsion angles between protein sequences, our algorithm improves structure prediction in general. It not only determines the class of the most likely structure for a given amino acid sequence, but it can also predict and model multiple structures of the same sequence, something many other software tools are not able to achieve this point. We performed our method and compared our results with previously published methods^8,9,23^ for prediction of protein structure classes in two large CATH protein structure classification datasets^24^. The CATH database contains a hierarchical classification of protein domains on the basis of class (C), architecture (A), topology (T), and homologous superfamily (H). We used the same dataset as that in Rackovsky’s research^23^. Rackovsky presented a ten-dimension vector method based on the physical properties of protein sequence and got an average of 79.5% accuracy. Our new prediction method performed well with an average of 92.5% accuracy for structure classification, which is a great improvement than Rackovsky’s previous research^23^. This method was also applied to a single amino acid sequence to model four different known protein structures. We also used the RaptorX and I-TASSER methods to predict the structure of the same sequence and compared the results with our method. The precision and reliability of our results were verified by calculating the dissimilarity of the predicted and actual protein structures. We used the root-mean-square deviation (RMSD) measure, the TM-score value, and the Yau–Hausdorff distance to calculate dissimilarity^25,26^. The Yau–Hausdorff distance is a metric to measure the difference of two proteins of any lengths based on the three-dimensional coordinates of their atoms which does not need aligning and superimposing two structures^25,26^. Our results demonstrate that this new approach is efficient and reliable on protein structure prediction, and can obtain multiple different structures for the same sequence, improve protein-folding recognition, classification of structural motifs, and refinement of sequence alignment.

## Results

### Prediction of protein structure classes in the CATH dataset

We used our torsion angle method to predict the most likely structure of each protein domain in two subsets of the CATH dataset. The ‘59 CAT’ group consisted of 59 CAT classes with at least 20 members (a total of 4319 sequences), whereas the ‘60 CAT’ group consisted of 60 CAT classes with 10–19 members (a total of 821 sequences). For each protein domain, we regarded its predicted classification correct if the class of predicted structure was the same as that of the empirically determined one. The accuracy rate of this prediction was defined as the number of correct classifications divided by the total number of proteins that were classified. We compared our results with those of a previous study that used a 10-dimensional vector method to analyze protein structure classes^23^. We also applied the methods developed by Chou on the same dataset^8,9^. Complete results are shown in Table 1. From this table, we can find that the accuracies by our method are higher than the other methods, which indicate our torsion angle method performs as well or better than the previous method for prediction of all the domain categories.

**Table 1:** Comparison of the accuracies of different methods for the prediction of protein structural classes.

Class	60 CAT group accuracies			59 CAT group accuracies		
	C = 1	C = 2	C = 3	C = 1	C = 2	C = 3
Protein numbers	195	145	481	762	1220	2337
Torsion angle method	87%	87%	96%	94%	97%	94%
10-dimensional vector method^23^	66%	56%	73%	92%	97%	93%
Method in Ref.^8^	50%	77%	90%	47%	76%	60%
Euclidian distance method^9^	74%	59%	61%	67%	69%	60%
Hamming distance method^9^	72%	54%	61%	62%	66%	61%

Each group is divided into alpha structure (C = 1), beta structure (C = 2) and mixed structure (C = 3) classes.

### Prediction of multiple protein structures from a single amino acid sequence

**Figure 1:**
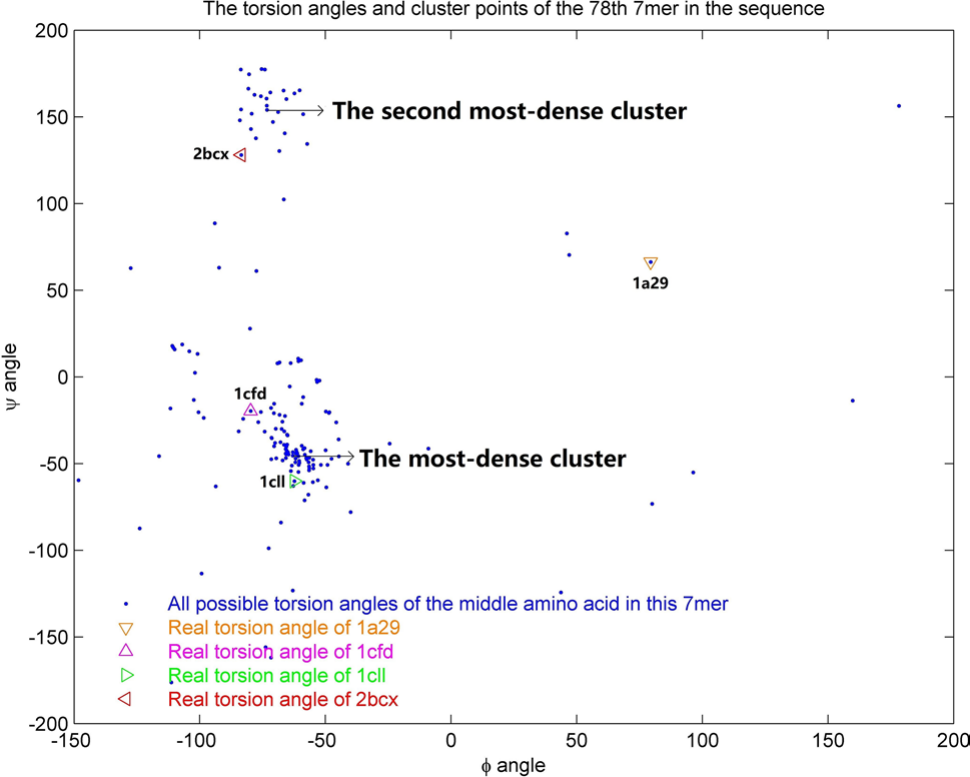
The torsion angles of the 78th amino acid heptamer in a sequence that results in four protein structures (1a29, 1cfd, 1cll, and 2bcx). The blue points represent all possible torsion angles and the torsion angles corresponding to each of the four protein structures are indicated. The most-dense cluster and second most-dense cluster used for constructing the predicted structures are pointed out.

**Figure 2:**
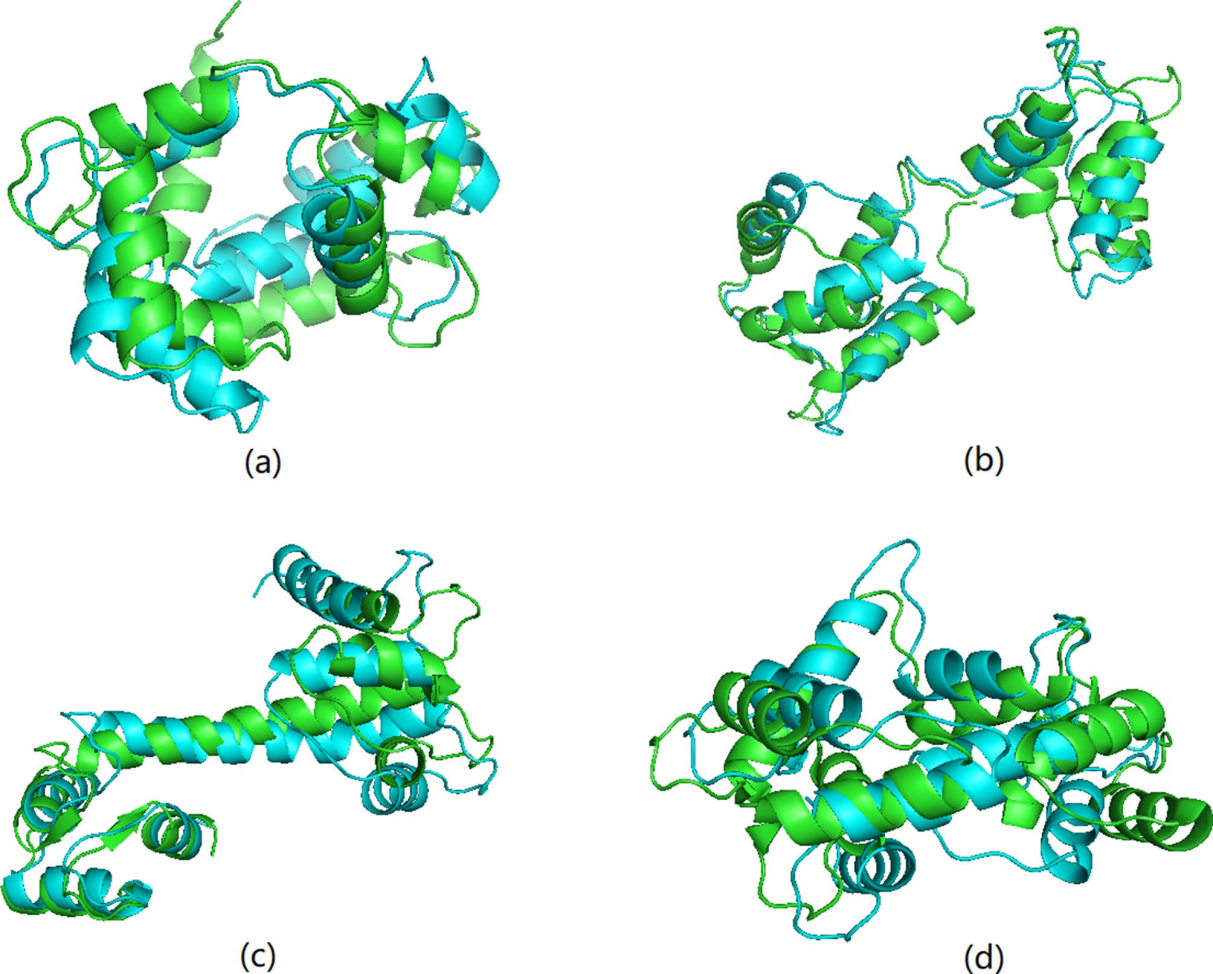
Alignment of empirically determined and predicted structures corresponding to a single amino acid sequence using our method. Known structures are shown in green, and predicted structures in blue, for (**a**) protein 1a29, (**b**) protein 1cfd, (**c**) protein 1cll, and (**d**) protein 2bcx.

**Figure 3:**
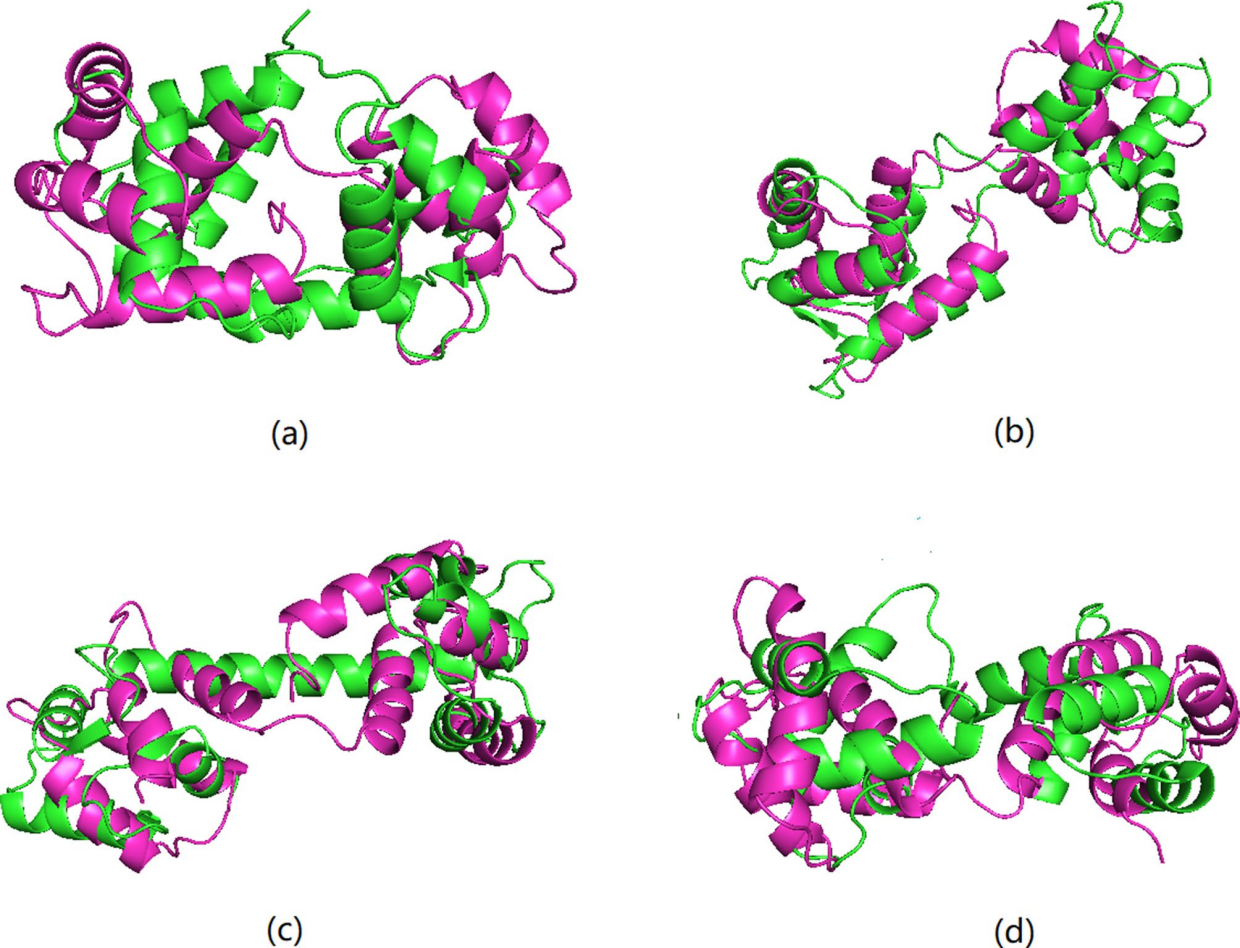
Alignment of empirically determined and predicted structures corresponding to a single amino acid sequence using RaptorX method. Known structures are shown in green, and predicted structure in purple, for (**a**) protein 1a29, (**b**) protein 1cfd, (**c**) protein 1cll, and (**d**) protein 2bcx. Here the purple structures in (**a**), (**b**), (**c**) and (**d**) are the same one obtained by RaptorX method.

Our method was tested by analysis of a 148 amino acid sequence, to predict four known protein structures (1a29, 1cfd, 1cll and 2bcx) based on this sequence. We first checked the locations for each of the 142 heptamers appeared in the 96,501 reliable protein structures database and collected the torsion angle points associated with the central amino acid of the heptamer. The torsion angles of the 78th heptamer are shown in Fig. 1 as an example, and detailed steps for constructing the four predicted structures corresponding to the four proteins with the same sequence are explained in the “Methods” section. The alignments between the known and predicted protein structures using our method are shown in Fig. 2. The Yau–Hausdorff distances, RMSD values and TM-score values for each pair of structures are calculated in Table 2. Since we only construct the main chain structures by torsion angles, these distances are computed after deleting the side residue parts of the native structures. We also used the RaptorX and I-TASSER methods to predict the protein structure for this amino acid sequence. These methods could only provide one most likely structure which performed not well in predicting multiple structures for a specific sequence. The Yau–Hausdorff distances, RMSD values and TM-score values between the constructed structure of each method and the four known ones are listed in Table 2, and the alignments between the known and predicted protein structures using RaptorX method are shown in Fig. 3. The purple structures in (a), (b), (c) and (d) in Fig. 3 are the same one obtained by RaptorX method. In Table 2, both Yau–Hausdorff distance and RMSD measure between each of the constructed structure performed by our method and the empirically determined one is smaller than those of RaptorX and I-TASSER methods, and the TM-score values are reverse. It also indicates that the predicted structures of our method are more similar than those of RaptorX by comparing Figs. 2 and 3. Although the predicted and known protein structures do not completely overlap by our method that is probably because the torsion angles of the predicted structure are not the same as the empirically determined one, the distances are small enough (with the diameter of every structure being larger than 50 angstroms) to indicate that each pair of structures is similar, demonstrating that this methodology can predict empirically determined structures from a specific amino acid sequence.

**Table 2:** Yau–Hausdorff distances, RMSD values and TM-score values between the empirically determined and predicted structures of the four proteins with the same amino acid sequence using our method and RaptorX method.

Protein ID	1a29	1cfd	1cll	2bcx
Yau–Hausdorff distance by our method	1.901	2.574	1.124	2.743
Yau–Hausdorff distance by RaptorX method	5.830	2.654	4.295	2.899
Yau–Hausdorff distance by I-TASSER method	1.925	6.530	8.441	3.170
RMSD by our method	3.704	4.786	3.330	5.821
RMSD by RaptorX method	14.929	6.782	11.620	12.221
RMSD by I-TASSER method	4.655	4.849	3.982	9.288
TM-score by our method	0.596	0.631	0.718	0.557
TM-score by RaptorX method	0.321	0.496	0.324	0.328
TM-score by I-TASSER method	0.372	0.397	0.428	0.460

## Discussion

### Structural dynamics of proteins with the same sequence

One most significant potential application of our method is it could be applied to predict the structure of a sequence for which there is no prior structural information. Given a protein sequence without structural information, we can predict the most likely structure for it. Another potential application may be used in structural dynamics. As the four known protein structures all correspond to the same amino acid sequence, it is possible that each structure could transform into one of the other structures. As described above, all possible torsion angles for each heptamer are calculated, enabling construction of all possible structures of the sequence. The dynamic process of transformation between protein structures with the same amino acid sequence can be constructed based on these possible structures. The transformation among the predicted structures can be ordered according to a metric, such as minimize the Yau–Hausdorff distances, beginning with one known structure and finishing with the other. The further in-depth study will discuss the structural dynamics.

## Conclusions

With the continuing development of sequencing technologies, methods are required for prediction of protein structures from amino acid sequences. In this study, we have provided an unsupervised method for protein structure prediction and constructing structures using the amino acid sequence via integrating and analyzing large torsion angle information in the Protein Data Bank. We reconstruct the structures of four proteins with the same sequence and compare the results with those obtained by RaptorX and I-TASSER methods, which could only predict one possible structure for a given sequence. One can clearly view the similarity comparison and calculate the value using different kinds of scores, such as the Yau–hausdorff distance^25,26^, RMSD, and TM-score between the native structure and constructed structure, then verify the precision of our method. It can generate multiple structures according to the amino acid sequence as well as provide a most likely structure to determine the property of the protein sequence. The new prediction model performs well, with an average of 92.5% accuracy for structure classification on two large CATH datasets, which makes a great improvement than many other methods^8,9,23^. This demonstrates our method is efficient and reliable on protein structure prediction study.

## Methods

### Datasets

To determine the possible torsion angles of the central residues of amino acid heptamers, 96,501 reliable protein structures were downloaded from the PDB website to provide a structure database (see Supplementary information). All the coordinates of protein atoms are in the PDB files. We used the ‘ramachandran.m’ function in MATLAB software to compute the torsion angles of these 96,501 structures. This function in MATLAB software could read PDB files and record the coordinates of atoms, then compute all the torsion angles.

The CATH database contains sequence and structure information for a large number of protein domains, organized hierarchically by class, architecture, topology and homology. Our method was compared with previous methods for its ability to predict the class assignment of two groups of protein domains, as defined previously^8,9,23^. We used the same dataset as that of Rackovsky’s^23^. The classes are: ‘C = 1’, α-helical structures; ‘C = 2’, β-sheet/barrel structures; and ‘C = 3’, mixed α/β structures. After deleting the sequences with fewer than 60 amino acids from the CathDomainSeqs.S35.ATOM.v3.1.020 database and restricting our attention to the CAT classes, the ‘59 CAT’ group consisted of 59 CAT classes with at least 20 members (a total of 4319 domain sequences), whereas the ‘60 CAT’ group consisted of 60 CAT classes with 10–19 members (a total of 821 domain sequences).

### Determination of torsion angle clusters

For each sequence *S* of length *N* in the CAT groups, the *N* − 6 possible amino acid heptamers are determined. For example, the nonameric sequence ‘CGDYAHCKS’ has three heptamers ‘CGDYAHC’, ‘GDYAHCK’ and ‘DYAHCKS’. It is a common sense that the first three neighboring amino acids have an effect on the fourth amino acid torsion angles, therefore pentamers are not enough for determining the amino acid torsion angles. Although the first amino acid has an effect on the fifth amino acid, it is weak, so the use of nonamers is not necessary. That is why heptamers are chosen for collecting the torsion angles information of amino acids.

For each heptamer of *S*, all occurrences in the structure database are identified, along with all pairs of torsion angles associated with the central amino acid of the heptamer. A pair of torsion angles can be treated as coordinates of a point in a plane. All identified torsion angle pairs for a heptamer’s central amino acid are plotted in a plane. The most-dense cluster is determined by taking each integer point as a center to draw circles of the same size and choosing the center of the corresponding circle that has the highest number of torsion angles as the cluster. This process is repeated for each of the *N − *6 heptamers in *S*.

### Predicting the most likely protein structure

In this study, the predicted structure refers to main chain structure. Since the main chain is determined if all the torsion angles are fixed, we can use these angles to construct the main chain structure by Pymol software. For each sequence *S*, the main chain protein structures are predicted on the basis of the most-dense clusters of torsion angle pairs for the *N − *6 heptamers. The first cluster (for the first heptamer) represents the torsion angles between the fourth and fifth amino acids of *S*. In Pymol, the first cluster is used to set the torsion angles between these two amino acids. The second cluster represents the torsion angles between the fifth and sixth amino acids, and so on. With these torsion angles, the positions of each amino acid are fixed in Pymol, enabling prediction of the most likely structure of *S*.

### Classification of protein structures

Two methods are used for determination of the classification which each constructed most likely protein structure belongs to. One approach uses the Definition of Secondary Structure of Proteins (DSSP) tool for standardization of structure assignment^27^. DSSP is a software of structure assignments for all protein structures entries. It is used for determining the classification of our prediction of the structure of the most likely protein by putting the predicted structure into the software and running the program directly.

A second approach uses the Ramachandran plot method to visualize energetically allowed regions for backbone dihedral angles ψ against φ of amino acid residues in protein structures^28^. Because dihedral-angle values are circular and $- 180^{^\circ }$ is equal to $180^{^\circ }$, the edges of the Ramachandran plot ‘wrap’ right-to-left and bottom-to-top. For two torsion angles $\left( {\psi_{1} ,\varphi_{1} } \right)$ and $\left( {\psi_{2} ,\varphi_{2} } \right)$, where $- 180^{^\circ } \le \psi_{1} ,\varphi_{1} ,\psi_{2} ,\varphi_{2} \le 180^{^\circ }$, the distance between $\psi_{1}$ and $\psi_{2}$ is ${\text{min}}\left\{ {\left| {\psi_{1} - \psi_{2} } \right|,360^{^\circ } - \left| {\psi_{1} - \psi_{2} } \right|} \right\}$. Similarly, the distance between $\varphi_{1}$ and $\varphi_{2}$ is ${\text{min}}\left\{ {\left| {\varphi_{1} - \varphi_{2} } \right|,360^{^\circ } - \left| {\varphi_{1} - \varphi_{2} } \right|} \right\}$. So the distance *D* between the two torsion angles is computed as follows:

$$ \begin{aligned} & D\left( {\left( {\psi_{1} ,\varphi_{1} } \right),\left( {\psi_{2} ,\varphi_{2} } \right)} \right) \\ & \quad = \sqrt {\left( {{\text{min}}\left\{ {\left| {\psi_{1} - \psi_{2} } \right|,360^{^\circ } - \left| {\psi_{1} - \psi_{2} } \right|} \right\}} \right)^{2} + \left( {{\text{min}}\left\{ {\left| {\varphi_{1} - \varphi_{2} } \right|,360^{^\circ } - \left| {\varphi_{1} - \varphi_{2} } \right|} \right\}} \right)^{2} } . \\ \end{aligned} $$

The regions where the majority of the torsion angles lie are different for each of the protein structure classes ‘C = 1’, ‘C = 2’ and ‘C = 3’. For example, most of the torsion angles of protein structures in class ‘C = 1’ lie in the upper left side of the Ramachandran plot. Based on this location feature of the three classes, classifications of our predictions of the most likely protein structures are determined by identification of the regions in which most of the torsion angles are located in the Ramachandran plot.

### Constructing multiple protein structures for a given sequence

Given an amino acid sequence *S* of length *N*, we can predict all possible structures for it. As described above, all occurrences of the torsion angles associated with the central amino acid of the *N* − 6 heptamers in sequence *S* are determined from the structure database at first. Not only the most-dense cluster is determined for predicting the structure, but also the second most-dense cluster is used as another choice for some heptamers with large number of appearance times in the structure database when constructing multiple structures for the sequence *S*. Among the whole possible structures constructed by these cluster points, the ones which have the minimum Yau–Hausdorff distance with the known structures are chosen as the multiple predicted structures for sequence *S*.

### Yau–Hausdorff distance between protein structures

The Yau–Hausdorff distance is used to calculate the dissimilarity between protein structures here^25,26^. Each protein structure is regarded as a three-dimensional point set consisting of all the atom coordinates. Define the minimum one-dimensional Hausdorff distance of two finite point sets $A_{1}$ and $B_{1}$ in ${\mathbb{R}}$ as

$$ H^{1} \left( {A_{1} ,B_{1} } \right) = \mathop {\min }\limits_{{t \in {\mathbb{R}}}} h\left( {A_{1} + t,B_{1} } \right), $$

where *h* is the Hausdorff distance

$$ h\left( {A_{1} ,B_{1} } \right) = \max \left\{ {\mathop {\max }\limits_{{a \in A_{1} }} \mathop {\min }\limits_{{b \in B_{1} }} d\left( {a,b} \right),\mathop {\max }\limits_{{b \in B_{1} }} \mathop {\min }\limits_{{a \in A_{1} }} d\left( {b,a} \right)} \right\}, $$

here $d\left( {a,b} \right)$ is the Euclidean distance between two points *a* and *b*, and $h\left( {A_{1} + t,B_{1} } \right)$ stands for the Hausdorff distance between $A_{1}$ and $B_{1}$ after shifting $A_{1}$ by *t*. The Yau–Hausdorff distance $D\left( {A,B} \right)$ of two point sets *A* and *B* in ${\mathbb{R}}^{3}$ is then defined in terms of $H^{1}$:

$$ D\left( {A,B} \right) = \max \left\{ {\mathop {\max }\limits_{{\theta^{2} }} \mathop {\min }\limits_{{\varphi^{2} }} H^{1} \left( {P_{x} \left( {A^{{\theta^{2} }} } \right),P_{x} \left( {B^{{\varphi^{2} }} } \right)} \right),\mathop {\max }\limits_{{\varphi^{2} }} \mathop {\min }\limits_{{\theta^{2} }} H^{1} \left( {P_{x} \left( {A^{{\theta^{2} }} } \right),P_{x} \left( {B^{{\varphi^{2} }} } \right)} \right)} \right\}, $$

where $P_{x} \left( {A^{{\theta^{2} }} } \right)$ is a one-dimensional point set representing the projection of *A* on the x-axis after being rotated by three-dimensional rotation angle $\theta^{2}$.

The Yau–Hausdorff distance is a natural metric which takes all possible translation and rotation into consideration for calculating the dissimilarity between protein structures. Comparing with aligning methods, the computational complexity has been reduced by projecting three-dimensional point sets into one-dimensional space in calculation without losing any information.

## Supplementary information

Supplementary Information.

## Data Availability

The datasets used in this study could be found in Supplementary information.

## Abbreviations

NMR: Nuclear magnetic resonance
PDB: Protein data bank
CATH: Class, architecture, topology and homologous superfamily
RMSD: Root mean square deviation
DSSP: Definition of secondary structure of proteins
