# Supplementary material for: Amino acid torsion angles enable prediction of protein fold classification
Source: Sci Rep. 2020 Dec 10;10:21773. doi: 10.1038/s41598-020-78465-1 (PMC7729947; doi:10.1038/s41598-020-78465-1)
Supplement: Supplementary file 1 — Supplementary Information. [file 41598_2020_78465_MOESM1_ESM.docx]

Amino acid torsion angles enable prediction of protein fold classification

Kun Tian^a#^, Xin Zhao^b#^, Xiaogeng Wan^c^, Stephen Yau^c*^

^a^School of Mathematics, Renmin University of China, Beijing 100872, PR China

^b^Department of Cryptography and Technology, Beijing Electronic Science and Technology Institute, Beijing 100070, PR China

^c^Department of Mathematical Sciences, Tsinghua University, Beijing 100084, PR China

^#^ These authors contributed equally to this work.

* To whom correspondence should be addressed: [yau@uic.edu](mailto:yau@uic.edu)

Supplementary information file——flow diagram of our method


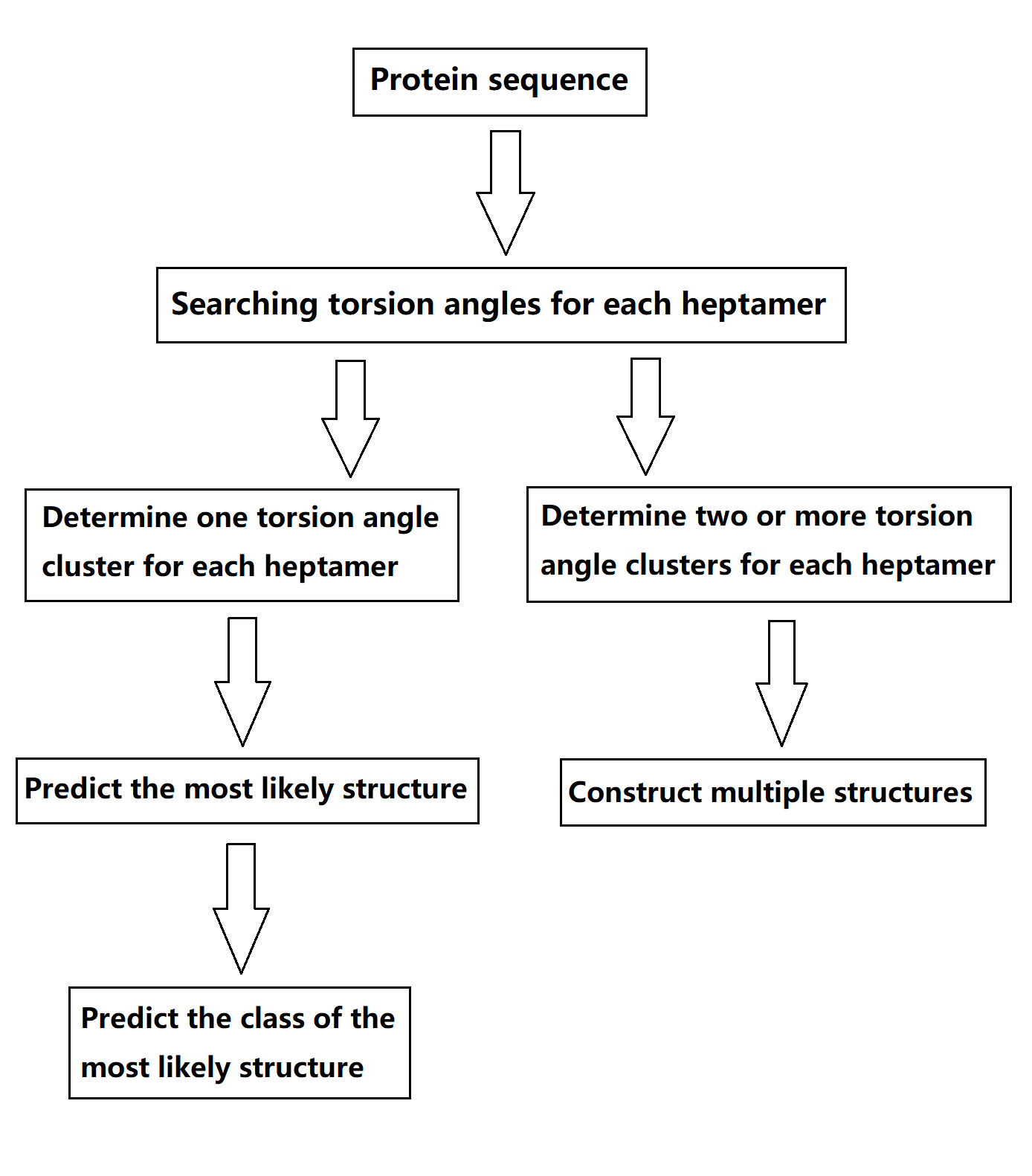


Supplementary information file——96501 reliable protein IDs

101M 102L 102M 103L 103M 104L 104M 105M 106M 107L 107M 108L 108M 109L 109M 10GS 110L 110M 111L 111M 112L 112M 113L 114L 115L 117E 118L 119L 11AS 11BA 11BG 11GS 120L 121P 122L 123L 125L 126L 127L 128L 129L 12AS 12CA 12E8 12GS 130L 131L 132L 133L 134L 135L 137L 138L 139L 13GS 13PK 140L 141L 142L 143L 144L 145L 146L 147L 148L 149L 14GS 150L 151L 152L 153L 154L 155C 155L 156L 157L 158L 159L 15C8 160L 161L 162L 163L 164L 165L 166L 167L 168L 169L 16GS 16PK 16VP 170L 171L 172L 173L 174L 175L 176L 177L 178L 17GS 180L 181L 182L 183L 184L 185L 186L 187L 188L 189L 18GS 190L 1914 191L 192L 193L 194L 195L 196L 197L 198L 199L 19GS 19HC 1A00 1A01 1A04 1A05 1A06 1A07 1A08 1A09 1A0B 1A0C 1A0D 1A0E 1A0F 1A0G 1A0H 1A0I 1A0J 1A0K 1A0L 1A0M 1A0O 1A0P 1A0Q 1A0R 1A0S 1A0T 1A0U 1A0Z 1A12 1A14 1A15 1A16 1A17 1A18 1A19 1A1A 1A1B 1A1C 1A1E 1A1M 1A1N 1A1O 1A1Q 1A1R 1A1S 1A1X 1A21 1A22 1A25 1A26 1A27 1A28 1A29 1A2A 1A2B 1A2C 1A2D 1A2F 1A2G 1A2J 1A2K 1A2L 1A2M 1A2N 1A2O 1A2P 1A2Q 1A2T 1A2U 1A2V 1A2W 1A2X 1A2Y 1A2Z 1A30 1A32 1A33 1A37 1A38 1A39 1A3A 1A3B 1A3C 1A3D 1A3E 1A3F 1A3G 1A3H 1A3I 1A3J 1A3K 1A3L 1A3N 1A3O 1A3R 1A3S 1A3T 1A3U 1A3V 1A3W 1A3X 1A3Y 1A3Z 1A40 1A41 1A42 1A43 1A44 1A45 1A46 1A47 1A48 1A49 1A4A 1A4B 1A4C 1A4E 1A4F 1A4G 1A4H 1A4I 1A4J 1A4K 1A4L 1A4M 1A4O 1A4P 1A4Q 1A4R 1A4S 1A4U 1A4V 1A4W 1A4X 1A4Y 1A4Z 1A50 1A52 1A53 1A54 1A55 1A58 1A59 1A5A 1A5B 1A5C 1A5D 1A5F 1A5G 1A5H 1A5I 1A5K 1A5L 1A5M 1A5N 1A5O 1A5P 1A5Q 1A5S 1A5T 1A5U 1A5V 1A5W 1A5X 1A5Y 1A5Z 1A61 1A62 1A64 1A65 1A68 1A69 1A6A 1A6C 1A6D 1A6E 1A6F 1A6G 1A6I 1A6J 1A6K 1A6L 1A6M 1A6N 1A6P 1A6Q 1A6R 1A6T 1A6U 1A6V 1A6W 1A6Z 1A70 1A71 1A72 1A75 1A76 1A77 1A78 1A79 1A7A 1A7B 1A7C 1A7D 1A7E 1A7G 1A7H 1A7J 1A7K 1A7L 1A7N 1A7O 1A7P 1A7Q 1A7R 1A7S 1A7T 1A7U 1A7V 1A7W 1A7X 1A7Y 1A7Z 1A80 1A81 1A82 1A85 1A86 1A87 1A88 1A8A 1A8B 1A8D 1A8E 1A8F 1A8G 1A8H 1A8I 1A8J 1A8K 1A8L 1A8M 1A8O 1A8P 1A8Q 1A8R 1A8S 1A8T 1A8U 1A8V 1A8Y 1A8Z 1A92 1A94 1A95 1A96 1A97 1A98 1A99 1A9B 1A9C 1A9E 1A9M 1A9O 1A9P 1A9Q 1A9R 1A9S 1A9T 1A9U 1A9W 1A9X 1A9Y 1A9Z 1AA0 1AA1 1AA2 1AA4 1AA5 1AA6 1AA7 1AAC 1AAJ 1AAL 1AAM 1AAN 1AAP 1AAQ 1AAR 1AAT 1AAW 1AAX 1AAZ 1AB0 1AB1 1AB4 1AB5 1AB6 1AB8 1AB9 1ABA 1ABB 1ABE 1ABF 1ABI 1ABJ 1ABN 1ABO 1ABQ 1ABR 1ABS 1ABW 1ABY 1AC1 1AC4 1AC5 1AC6 1AC8 1ACB 1ACC 1ACD 1ACF 1ACJ 1ACL 1ACM 1ACO 1ACV 1ACX 1ACY 1AD0 1AD1 1AD2 1AD3 1AD4 1AD5 1AD6 1AD8 1AD9 1ADB 1ADC 1ADD 1ADE 1ADF 1ADG 1ADI 1ADJ 1ADL 1ADO 1ADQ 1ADS 1ADU 1ADV 1ADW 1ADY 1AE1 1AE2 1AE3 1AE4 1AE5 1AE6 1AE7 1AE8 1AE9 1AEB 1AEC 1AED 1AEE 1AEF 1AEG 1AEH 1AEI 1AEJ 1AEK 1AEM 1AEN 1AEO 1AEP 1AEQ 1AER 1AES 1AET 1AEU 1AEV 1AEW 1AEX 1AF0 1AF2 1AF3 1AF4 1AF5 1AF6 1AF7 1AF9 1AFA 1AFB 1AFC 1AFD 1AFE 1AFK 1AFL 1AFQ 1AFR 1AFS 1AFU 1AFV 1AFW 1AG0 1AG1 1AG6 1AG8 1AG9 1AGB 1AGC 1AGD 1AGE 1AGF 1AGI 1AGJ 1AGM 1AGN 1AGP 1AGQ 1AGR 1AGS 1AGW 1AGX 1AGY 1AH0 1AH3 1AH4 1AH5 1AH6 1AH7 1AH8 1AHA 1AHB 1AHC 1AHE 1AHF 1AHG 1AHH 1AHI 1AHJ 1AHN 1AHO 1AHP 1AHQ 1AHR 1AHS 1AHT 1AHU 1AHV 1AHW 1AHX 1AHY 1AHZ 1AI1 1AI2 1AI3 1AI4 1AI5 1AI6 1AI7 1AI8 1AI9 1AIA 1AIB 1AIC 1AID 1AIE 1AIF 1AIG 1AIH 1AII 1AIJ 1AIK 1AIL 1AIM 1AIN 1AIP 1AIQ 1AIR 1AIU 1AIV 1AIX 1AIZ 1AJ0 1AJ2 1AJ5 1AJ6 1AJ7 1AJ8 1AJ9 1AJA 1AJB 1AJC 1AJD 1AJG 1AJH 1AJJ 1AJK 1AJM 1AJN 1AJO 1AJP 1AJQ 1AJR 1AJS 1AJV 1AJX 1AJZ 1AK0 1AK1 1AK2 1AK4 1AK5 1AK9 1AKA 1AKB 1AKC 1AKD 1AKE 1AKG 1AKI 1AKJ 1AKL 1AKM 1AKN 1AKO 1AKQ 1AKR 1AKS 1AKT 1AKU 1AKV 1AKW 1AKY 1AKZ 1AL0 1AL1 1AL2 1AL3 1AL4 1AL6 1AL7 1AL8 1ALA 1ALB 1ALC 1ALD 1ALH 1ALI 1ALJ 1ALK 1ALL 1ALN 1ALQ 1ALU 1ALV 1ALW 1ALX 1ALY 1ALZ 1AM1 1AM2 1AM4 1AM5 1AM6 1AM7 1AMA 1AME 1AMF 1AMH 1AMI 1AMJ 1AMK 1AMM 1AMN 1AMO 1AMP 1AMQ 1AMR 1AMS 1AMT 1AMU 1AMW 1AMX 1AMY 1AMZ 1AN0 1AN1 1AN5 1AN7 1AN8 1AN9 1ANB 1ANC 1AND 1ANE 1ANF 1ANG 1ANI 1ANJ 1ANK 1ANN 1ANT 1ANU 1ANV 1ANW 1ANX 1AO0 1AO3 1AO5 1AO6 1AO7 1AOA 1AOB 1AOC 1AOD 1AOE 1AOF 1AOG 1AOH 1AOJ 1AOK 1AOL 1AOM 1AON 1AOP 1AOQ 1AOR 1AOS 1AOV 1AOW 1AOX 1AOZ 1AP2 1AP5 1AP6 1AP9 1APA 1APB 1APH 1APM 1APN 1APT 1APU 1APV 1APW 1APX 1APY 1APZ 1AQ0 1AQ1 1AQ2 1AQ6 1AQ7 1AQ8 1AQB 1AQC 1AQD 1AQE 1AQF 1AQH 1AQI 1AQJ 1AQK 1AQL 1AQM 1AQN 1AQP 1AQT 1AQU 1AQV 1AQW 1AQX 1AQY 1AQZ 1AR0 1AR1 1AR2 1AR4 1AR5 1AR6 1AR7 1AR8 1AR9 1ARB 1ARC 1ARG 1ARH 1ARI 1ARL 1ARM 1ARO 1ARP 1ARS 1ART 1ARU 1ARV 1ARW 1ARX 1ARY 1ARZ 1AS0 1AS2 1AS3 1AS4 1AS6 1AS7 1AS8 1ASA 1ASB 1ASC 1ASD 1ASE 1ASF 1ASG 1ASH 1ASJ 1ASK 1ASL 1ASM 1ASN 1ASO 1ASP 1ASQ 1ASS 1AST 1ASU 1ASV 1ASW 1ASX 1AT0 1AT1 1AT3 1AT5 1AT6 1ATG 1ATH 1ATI 1ATJ 1ATK 1ATL 1ATN 1ATP 1ATR 1ATS 1ATT 1ATU 1ATZ 1AU0 1AU1 1AU2 1AU3 1AU4 1AU8 1AU9 1AUA 1AUC 1AUE 1AUG 1AUI 1AUJ 1AUK 1AUM 1AUN 1AUO 1AUP 1AUQ 1AUR 1AUS 1AUT 1AUV 1AUW 1AUX 1AUY 1AV1 1AV2 1AV4 1AV5 1AV7 1AV8 1AVA 1AVB 1AVC 1AVD 1AVE 1AVF 1AVG 1AVH 1AVK 1AVL 1AVM 1AVN 1AVO 1AVP 1AVQ 1AVR 1AVS 1AVT 1AVU 1AVV 1AVW 1AVX 1AVY 1AVZ 1AW1 1AW2 1AW5 1AW7 1AW8 1AW9 1AWB 1AWD 1AWF 1AWH 1AWI 1AWP 1AWQ 1AWR 1AWS 1AWT 1AWU 1AWV 1AX0 1AX1 1AX2 1AX4 1AX8 1AX9 1AXA 1AXB 1AXC 1AXD 1AXE 1AXG 1AXI 1AXK 1AXM 1AXN 1AXQ 1AXR 1AXS 1AXT 1AXW 1AXY 1AXZ 1AY0 1AY1 1AY2 1AY4 1AY5 1AY6 1AY7 1AY8 1AY9 1AYA 1AYB 1AYC 1AYD 1AYE 1AYF 1AYI 1AYL 1AYM 1AYN 1AYO 1AYP 1AYR 1AYU 1AYV 1AYW 1AYX 1AYY 1AYZ 1AZ1 1AZ2 1AZ3 1AZ4 1AZ5 1AZ8 1AZB 1AZC 1AZD 1AZF 1AZI 1AZL 1AZM 1AZN 1AZO 1AZR 1AZS 1AZT 1AZU 1AZV 1AZW 1AZX 1AZY 1AZZ 1B00 1B02 1B04 1B05 1B06 1B07 1B08 1B09 1B0A 1B0B 1B0C 1B0D 1B0E 1B0F 1B0G 1B0H 1B0I 1B0J 1B0K 1B0L 1B0M 1B0N 1B0O 1B0P 1B0R 1B0T 1B0U 1B0V 1B0W 1B0X 1B0Y 1B0Z 1B11 1B12 1B13 1B14 1B15 1B16 1B17 1B18 1B19 1B1B 1B1C 1B1E 1B1H 1B1I 1B1J 1B1U 1B1X 1B1Y 1B1Z 1B20 1B21 1B24 1B25 1B26 1B27 1B2A 1B2B 1B2C 1B2D 1B2E 1B2F 1B2G 1B2H 1B2J 1B2K 1B2L 1B2O 1B2P 1B2R 1B2S 1B2U 1B2V 1B2W 1B2X 1B2Y 1B2Z 1B30 1B31 1B32 1B33 1B34 1B35 1B37 1B38 1B39 1B3A 1B3B 1B3D 1B3E 1B3F 1B3G 1B3H 1B3J 1B3K 1B3L 1B3N 1B3O 1B3Q 1B3R 1B3S 1B3U 1B3V 1B3W 1B3X 1B3Y 1B3Z 1B40 1B41 1B42 1B43 1B44 1B46 1B47 1B48 1B49 1B4A 1B4B 1B4D 1B4E 1B4F 1B4H 1B4J 1B4K 1B4L 1B4N 1B4P 1B4S 1B4T 1B4U 1B4V 1B4W 1B4X 1B4Z 1B51 1B52 1B54 1B55 1B56 1B57 1B58 1B59 1B5D 1B5E 1B5F 1B5G 1B5H 1B5I 1B5J 1B5L 1B5M 1B5O 1B5P 1B5Q 1B5S 1B5T 1B5U 1B5V 1B5W 1B5X 1B5Y 1B5Z 1B62 1B63 1B65 1B66 1B67 1B68 1B6A 1B6B 1B6C 1B6D 1B6E 1B6G 1B6H 1B6I 1B6J 1B6K 1B6L 1B6M 1B6P 1B6Q 1B6R 1B6S 1B6T 1B6U 1B6V 1B6W 1B6Z 1B70 1B71 1B73 1B74 1B76 1B77 1B78 1B79 1B7A 1B7B 1B7D 1B7E 1B7G 1B7H 1B7I 1B7J 1B7K 1B7L 1B7M 1B7N 1B7O 1B7P 1B7Q 1B7R 1B7S 1B7T 1B7U 1B7V 1B7X 1B7Y 1B7Z 1B80 1B82 1B85 1B86 1B87 1B88 1B89 1B8A 1B8C 1B8D 1B8E 1B8F 1B8G 1B8H 1B8J 1B8K 1B8L 1B8M 1B8N 1B8O 1B8P 1B8R 1B8S 1B8U 1B8V 1B8X 1B8Y 1B8Z 1B90 1B92 1B93 1B98 1B99 1B9A 1B9B 1B9C 1B9D 1B9E 1B9F 1B9H 1B9I 1B9J 1B9K 1B9L 1B9M 1B9N 1B9O 1B9S 1B9T 1B9V 1B9W 1B9X 1B9Y 1B9Z 1BA0 1BA1 1BA2 1BA3 1BA7 1BA8 1BAB 1BAF 1BAG 1BAI 1BAJ 1BAM 1BAN 1BAO 1BAP 1BAR 1BAS 1BAV 1BAW 1BAY 1BAZ 1BB0 1BB1 1BB3 1BB4 1BB5 1BB6 1BB7 1BB9 1BBB 1BBC 1BBD 1BBH 1BBJ 1BBP 1BBR 1BBS 1BBT 1BBU 1BBW 1BBZ 1BC0 1BC1 1BC2 1BC3 1BC5 1BCC 1BCD 1BCF 1BCG 1BCH 1BCJ 1BCK 1BCM 1BCO 1BCP 1BCR 1BCS 1BCU 1BCW 1BCX 1BCY 1BCZ 1BD0 1BD2 1BD3 1BD4 1BD7 1BD8 1BD9 1BDA 1BDB 1BDF 1BDG 1BDJ 1BDL 1BDM 1BDO 1BDQ 1BDR 1BDU 1BDW 1BDY 1BE0 1BE3 1BE4 1BE6 1BE7 1BE8 1BE9 1BEA 1BEB 1BEC 1BED 1BEE 1BEH 1BEJ 1BEK 1BEL 1BEM 1BEN 1BEO 1BEP 1BEQ 1BES 1BET 1BEU 1BEV 1BEX 1BEY 1BEZ 1BF2 1BF3 1BF6 1BFA 1BFB 1BFC 1BFD 1BFE 1BFF 1BFG 1BFK 1BFN 1BFO 1BFP 1BFR 1BFS 1BFT 1BFU 1BFV 1BG0 1BG2 1BG3 1BG4 1BG5 1BG6 1BG7 1BG8 1BG9 1BGA 1BGC 1BGD 1BGE 1BGF 1BGG 1BGI 1BGJ 1BGN 1BGO 1BGP 1BGQ 1BGS 1BGT 1BGU 1BGV 1BGW 1BGX 1BGY 1BH0 1BH2 1BH3 1BH5 1BH6 1BH8 1BH9 1BHC 1BHD 1BHE 1BHF 1BHG 1BHH 1BHJ 1BHL 1BHN 1BHO 1BHP 1BHQ 1BHS 1BHT 1BHW 1BHX 1BHY 1BHZ 1BI0 1BI1 1BI2 1BI3 1BI4 1BI5 1BI7 1BI8 1BI9 1BIA 1BIB 1BIC 1BID 1BIF 1BIH 1BII 1BIJ 1BIK 1BIL 1BIM 1BIN 1BIO 1BIQ 1BIR 1BIS 1BIT 1BIU 1BIW 1BIX 1BIY 1BIZ 1BJ1 1BJ3 1BJ4 1BJ5 1BJ7 1BJ9 1BJA 1BJE 1BJF 1BJG 1BJI 1BJJ 1BJK 1BJM 1BJN 1BJO 1BJP 1BJQ 1BJR 1BJT 1BJU 1BJV 1BJW 1BJZ 1BK0 1BK1 1BK2 1BK4 1BK5 1BK6 1BK7 1BK9 1BKA 1BKB 1BKC 1BKD 1BKE 1BKF 1BKG 1BKH 1BKJ 1BKL 1BKM 1BKN 1BKO 1BKP 1BKR 1BKS 1BKV 1BKW 1BKX 1BKY 1BKZ 1BL3 1BL4 1BL5 1BL6 1BL7 1BL8 1BL9 1BLB 1BLC 1BLE 1BLF 1BLH 1BLI 1BLL 1BLN 1BLP 1BLS 1BLU 1BLX 1BLZ 1BM0 1BM1 1BM2 1BM3 1BM7 1BM8 1BM9 1BMA 1BMB 1BMC 1BMD 1BMF 1BMG 1BMK 1BML 1BMM 1BMN 1BMO 1BMP 1BMQ 1BMS 1BMT 1BMZ 1BN1 1BN3 1BN4 1BN5 1BN6 1BN7 1BN8 1BNC 1BND 1BNE 1BNF 1BNG 1BNI 1BNJ 1BNL 1BNM 1BNN 1BNQ 1BNS 1BNT 1BNU 1BNV 1BNW 1BO1 1BO4 1BO5 1BO6 1BO7 1BO8 1BOA 1BOB 1BOF 1BOG 1BOH 1BOI 1BOL 1BOO 1BOQ 1BOS 1BOT 1BOU 1BOW 1BOX 1BOY 1BOZ 1BP0 1BP1 1BP2 1BP3 1BP4 1BP5 1BP6 1BPB 1BPD 1BPE 1BPH 1BPI 1BPJ 1BPL 1BPM 1BPN 1BPO 1BPQ 1BPT 1BPW 1BQ1 1BQ2 1BQ3 1BQ4 1BQ5 1BQ6 1BQ7 1BQ8 1BQ9 1BQA 1BQB 1BQC 1BQD 1BQE 1BQG 1BQH 1BQI 1BQK 1BQL 1BQM 1BQN 1BQO 1BQP 1BQQ 1BQR 1BQS 1BQU 1BQY 1BR1 1BR2 1BR4 1BR5 1BR6 1BR8 1BR9 1BRA 1BRB 1BRC 1BRE 1BRF 1BRG 1BRH 1BRI 1BRJ 1BRK 1BRL 1BRM 1BRO 1BRP 1BRQ 1BRR 1BRS 1BRT 1BRU 1BRW 1BRX 1BRY 1BS0 1BS1 1BS2 1BS3 1BS4 1BS5 1BS6 1BS7 1BS8 1BS9 1BSA 1BSB 1BSC 1BSD 1BSE 1BSF 1BSG 1BSI 1BSJ 1BSK 1BSL 1BSM 1BSO 1BSP 1BSQ 1BSR 1BSV 1BSW 1BSX 1BSY 1BSZ 1BT0 1BT1 1BT2 1BT3 1BT4 1BT5 1BT6 1BT8 1BT9 1BTC 1BTE 1BTG 1BTH 1BTI 1BTJ 1BTK 1BTL 1BTM 1BTN 1BTO 1BTP 1BTU 1BTW 1BTX 1BTY 1BTZ 1BU1 1BU2 1BU3 1BU4 1BU5 1BU6 1BU7 1BU8 1BUC 1BUD 1BUE 1BUG 1BUH 1BUI 1BUL 1BUN 1BUO 1BUP 1BUU 1BUV 1BUW 1BUX 1BV1 1BV3 1BV4 1BV7 1BV9 1BVA 1BVB 1BVC 1BVD 1BVI 1BVK 1BVL 1BVN 1BVP 1BVQ 1BVR 1BVS 1BVT 1BVU 1BVV 1BVW 1BVX 1BVY 1BVZ 1BW0 1BW8 1BW9 1BWA 1BWB 1BWC 1BWD 1BWF 1BWH 1BWI 1BWJ 1BWK 1BWL 1BWN 1BWO 1BWP 1BWQ 1BWR 1BWS 1BWU 1BWV 1BWW 1BWZ 1BX0 1BX1 1BX2 1BX3 1BX4 1BX6 1BX7 1BX8 1BX9 1BXA 1BXB 1BXC 1BXE 1BXG 1BXH 1BXI 1BXK 1BXM 1BXN 1BXO 1BXQ 1BXR 1BXS 1BXT 1BXU 1BXV 1BXW 1BXX 1BXY 1BXZ 1BY2 1BY3 1BY5 1BY7 1BY8 1BY9 1BYA 1BYB 1BYC 1BYD 1BYE 1BYF 1BYG 1BYH 1BYI 1BYL 1BYO 1BYP 1BYQ 1BYR 1BYS 1BYU 1BYW 1BYZ 1BZ0 1BZ1 1BZ4 1BZ5 1BZ6 1BZ7 1BZ8 1BZ9 1BZA 1BZC 1BZD 1BZE 1BZH 1BZJ 1BZL 1BZM 1BZO 1BZP 1BZQ 1BZR 1BZS 1BZW 1BZX 1BZY 1BZZ 1C02 1C03 1C08 1C09 1C0B 1C0C 1C0E 1C0F 1C0G 1C0I 1C0K 1C0L 1C0M 1C0N 1C0P 1C0Q 1C0R 1C0T 1C0U 1C10 1C12 1C14 1C16 1C1A 1C1B 1C1C 1C1D 1C1E 1C1F 1C1G 1C1H 1C1J 1C1K 1C1L 1C1M 1C1N 1C1O 1C1P 1C1Q 1C1R 1C1S 1C1T 1C1U 1C1V 1C1W 1C1X 1C1Y 1C1Z 1C21 1C22 1C23 1C24 1C25 1C26 1C27 1C28 1C29 1C2A 1C2B 1C2D 1C2E 1C2F 1C2G 1C2H 1C2I 1C2J 1C2K 1C2L 1C2M 1C2O 1C2P 1C2R 1C2T 1C2Y 1C30 1C39 1C3A 1C3B 1C3C 1C3D 1C3E 1C3F 1C3G 1C3H 1C3I 1C3J 1C3K 1C3L 1C3M 1C3N 1C3O 1C3P 1C3Q 1C3R 1C3S 1C3U 1C3V 1C3W 1C3X 1C40 1C41 1C43 1C44 1C45 1C46 1C47 1C48 1C4A 1C4C 1C4D 1C4F 1C4G 1C4K 1C4O 1C4P 1C4Q 1C4R 1C4T 1C4U 1C4V 1C4W 1C4X 1C4Y 1C4Z 1C50 1C51 1C52 1C53 1C5B 1C5C 1C5D 1C5E 1C5F 1C5G 1C5H 1C5I 1C5K 1C5L 1C5M 1C5N 1C5O 1C5P 1C5Q 1C5R 1C5S 1C5T 1C5U 1C5V 1C5W 1C5X 1C5Y 1C5Z 1C60 1C61 1C62 1C63 1C64 1C65 1C66 1C67 1C68 1C69 1C6A 1C6B 1C6C 1C6D 1C6E 1C6F 1C6G 1C6H 1C6I 1C6J 1C6K 1C6L 1C6M 1C6N 1C6O 1C6P 1C6Q 1C6R 1C6T 1C6V 1C6X 1C6Y 1C6Z 1C70 1C72 1C74 1C75 1C76 1C77 1C78 1C79 1C7B 1C7C 1C7D 1C7E 1C7F 1C7G 1C7H 1C7I 1C7J 1C7K 1C7N 1C7O 1C7P 1C7Q 1C7R 1C7S 1C7T 1C7Z 1C80 1C81 1C82 1C83 1C84 1C85 1C86 1C87 1C88 1C8B 1C8D 1C8E 1C8F 1C8G 1C8H 1C8I 1C8J 1C8K 1C8L 1C8M 1C8N 1C8O 1C8Q 1C8R 1C8S 1C8T 1C8U 1C8V 1C8W 1C8X 1C8Y 1C8Z 1C90 1C91 1C92 1C93 1C94 1C96 1C97 1C9C 1C9D 1C9E 1C9H 1C9I 1C9J 1C9K 1C9L 1C9M 1C9N 1C9O 1C9P 1C9T 1C9U 1C9V 1C9W 1C9X 1C9Y 1CA0 1CA1 1CA2 1CA3 1CA4 1CA7 1CA8 1CA9 1CAA 1CAD 1CAG 1CAH 1CAI 1CAJ 1CAK 1CAL 1CAM 1CAN 1CAO 1CAQ 1CAU 1CAV 1CAW 1CAX 1CAY 1CAZ 1CB0 1CB2 1CB4 1CB5 1CB6 1CB7 1CB8 1CBF 1CBG 1CBI 1CBJ 1CBK 1CBL 1CBM 1CBN 1CBO 1CBQ 1CBR 1CBS 1CBU 1CBW 1CBX 1CBY 1CC0 1CC1 1CC2 1CC3 1CC4 1CC5 1CC6 1CC7 1CC8 1CCA 1CCB 1CCC 1CCD 1CCE 1CCG 1CCI 1CCJ 1CCK 1CCL 1CCP 1CCR 1CCS 1CCT 1CCU 1CCW 1CCZ 1CD0 1CD1 1CD2 1CD3 1CD5 1CD8 1CD9 1CDC 1CDD 1CDE 1CDG 1CDH 1CDI 1CDJ 1CDK 1CDL 1CDM 1CDO 1CDP 1CDT 1CDU 1CDY 1CDZ 1CE0 1CE1 1CE2 1CE5 1CE6 1CE7 1CE8 1CE9 1CEA 1CEB 1CEC 1CEF 1CEG 1CEH 1CEI 1CEL 1CEM 1CEN 1CEO 1CEQ 1CER 1CES 1CET 1CEV 1CEW 1CEX 1CF0 1CF1 1CF2 1CF3 1CF5 1CF8 1CF9 1CFB 1CFD 1CFM 1CFN 1CFQ 1CFR 1CFS 1CFT 1CFV 1CFW 1CFY 1CFZ 1CG0 1CG1 1CG2 1CG3 1CG4 1CG5 1CG6 1CG8 1CG9 1CGD 1CGE 1CGF 1CGH 1CGI 1CGJ 1CGK 1CGL 1CGN 1CGO 1CGQ 1CGS 1CGT 1CGU 1CGV 1CGW 1CGX 1CGY 1CGZ 1CH0 1CH1 1CH2 1CH3 1CH4 1CH5 1CH7 1CH8 1CH9 1CHD 1CHG 1CHH 1CHI 1CHJ 1CHK 1CHM 1CHN 1CHO 1CHP 1CHQ 1CHU 1CHW 1CHZ 1CI0 1CI1 1CI3 1CI4 1CI6 1CI7 1CI8 1CI9 1CIA 1CIB 1CIC 1CID 1CIE 1CIF 1CIG 1CIH 1CII 1CIJ 1CIK 1CIL 1CIM 1CIN 1CIO 1CIP 1CIQ 1CIU 1CIV 1CIW 1CIY 1CIZ 1CJ0 1CJ1 1CJ2 1CJ3 1CJ4 1CJ6 1CJ7 1CJ8 1CJ9 1CJA 1CJB 1CJC 1CJD 1CJE 1CJF 1CJK 1CJL 1CJM 1CJP 1CJQ 1CJR 1CJS 1CJT 1CJU 1CJV 1CJW 1CJX 1CJY 1CK0 1CK1 1CK3 1CK4 1CK6 1CK7 1CKA 1CKB 1CKC 1CKD 1CKE 1CKF 1CKG 1CKH 1CKI 1CKJ 1CKL 1CKM 1CKN 1CKO 1CKP 1CKS 1CKU 1CL0 1CL1 1CL2 1CL5 1CL6 1CL7 1CLA 1CLC 1CLE 1CLI 1CLK 1CLL 1CLM 1CLO 1CLP 1CLS 1CLU 1CLV 1CLW 1CLX 1CLY 1CLZ 1CM0 1CM1 1CM2 1CM3 1CM4 1CM5 1CM7 1CM8 1CM9 1CMB 1CMC 1CMI 1CMJ 1CMK 1CML 1CMN 1CMP 1CMQ 1CMS 1CMT 1CMU 1CMV 1CMX 1CMY 1CN1 1CN3 1CN4 1CNB 1CNC 1CNE 1CNF 1CNG 1CNH 1CNI 1CNJ 1CNK 1CNM 1CNO 1CNQ 1CNR 1CNS 1CNT 1CNU 1CNV 1CNW 1CNX 1CNY 1CNZ 1CO6 1CO7 1CO8 1CO9 1COA 1COB 1COF 1COH 1COI 1COJ 1COL 1COM 1CON 1COS 1COT 1COV 1COW 1COY 1COZ 1CP0 1CP2 1CP3 1CP4 1CP5 1CP6 1CP7 1CP9 1CPB 1CPC 1CPD 1CPE 1CPF 1CPG 1CPH 1CPI 1CPJ 1CPM 1CPN 1CPO 1CPQ 1CPR 1CPS 1CPT 1CPU 1CPW 1CPX 1CPY 1CQ1 1CQ3 1CQ4 1CQ6 1CQ7 1CQ8 1CQ9 1CQA 1CQD 1CQE 1CQF 1CQI 1CQJ 1CQK 1CQM 1CQN 1CQP 1CQQ 1CQR 1CQS 1CQV 1CQW 1CQX 1CQY 1CQZ 1CR0 1CR1 1CR2 1CR4 1CR5 1CR6 1CR7 1CR9 1CRA 1CRB 1CRC 1CRG 1CRH 1CRI 1CRJ 1CRK 1CRL 1CRM 1CRN 1CRU 1CRW 1CRY 1CRZ 1CS0 1CS1 1CS3 1CS4 1CS6 1CS8 1CSB 1CSC 1CSE 1CSG 1CSH 1CSI 1CSJ 1CSK 1CSM 1CSN 1CSO 1CSP 1CSQ 1CSR 1CSS 1CSU 1CSV 1CSW 1CSX 1CT0 1CT1 1CT2 1CT4 1CT5 1CT8 1CT9 1CTE 1CTF 1CTJ 1CTM 1CTN 1CTP 1CTQ 1CTR 1CTS 1CTT 1CTU 1CTW 1CTX 1CTY 1CTZ 1CU0 1CU1 1CU2 1CU3 1CU4 1CU5 1CU6 1CUA 1CUB 1CUC 1CUD 1CUE 1CUF 1CUG 1CUH 1CUI 1CUJ 1CUK 1CUL 1CUN 1CUO 1CUP 1CUQ 1CUS 1CUU 1CUV 1CUW 1CUX 1CUY 1CUZ 1CV0 1CV1 1CV2 1CV3 1CV4 1CV5 1CV6 1CV7 1CV8 1CVA 1CVB 1CVC 1CVD 1CVE 1CVF 1CVH 1CVI 1CVK 1CVL 1CVM 1CVN 1CVR 1CVS 1CVU 1CVW 1CVZ 1CW1 1CW2 1CW3 1CW4 1CW7 1CWA 1CWB 1CWC 1CWD 1CWE 1CWF 1CWH 1CWI 1CWJ 1CWK 1CWL 1CWM 1CWN 1CWO 1CWQ 1CWR 1CWS 1CWT 1CWU 1CWV 1CWY 1CX2 1CX4 1CX6 1CX7 1CX8 1CX9 1CXA 1CXC 1CXE 1CXF 1CXH 1CXI 1CXK 1CXL 1CXP 1CXQ 1CXU 1CXV 1CXY 1CXZ 1CY0 1CY1 1CY2 1CY4 1CY5 1CY6 1CY7 1CY8 1CY9 1CYC 1CYD 1CYF 1CYG 1CYI 1CYJ 1CYN 1CYO 1CYW 1CYX 1CYY 1CZ1 1CZ3 1CZ7 1CZ8 1CZ9 1CZA 1CZB 1CZC 1CZD 1CZE 1CZF 1CZG 1CZH 1CZI 1CZJ 1CZK 1CZL 1CZM 1CZN 1CZO 1CZP 1CZQ 1CZR 1CZS 1CZT 1CZU 1CZV 1CZW 1CZY 1CZZ 1D00 1D01 1D03 1D04 1D06 1D07 1D09 1D0A 1D0B 1D0C 1D0D 1D0G 1D0H 1D0I 1D0J 1D0K 1D0L 1D0M 1D0N 1D0O 1D0Q 1D0S 1D0V 1D0X 1D0Y 1D0Z 1D1A 1D1B 1D1C 1D1G 1D1I 1D1J 1D1K 1D1L 1D1M 1D1P 1D1Q 1D1S 1D1T 1D1V 1D1W 1D1X 1D1Y 1D1Z 1D2A 1D2C 1D2E 1D2F 1D2G 1D2H 1D2K 1D2M 1D2N 1D2O 1D2P 1D2Q 1D2R 1D2S 1D2T 1D2U 1D2V 1D2W 1D2Y 1D2Z 1D3A 1D3B 1D3C 1D3D 1D3F 1D3G 1D3H 1D3J 1D3K 1D3L 1D3M 1D3N 1D3P 1D3Q 1D3S 1D3T 1D3V 1D3W 1D3Y 1D4A 1D4C 1D4D 1D4E 1D4F 1D4H 1D4I 1D4J 1D4K 1D4L 1D4M 1D4N 1D4O 1D4P 1D4S 1D4T 1D4V 1D4W 1D4X 1D4Y 1D4Z 1D5A 1D5B 1D5C 1D5D 1D5E 1D5F 1D5H 1D5I 1D5J 1D5L 1D5M 1D5N 1D5R 1D5S 1D5T 1D5W 1D5X 1D5Z 1D6A 1D6E 1D6F 1D6H 1D6I 1D6J 1D6M 1D6N 1D6O 1D6P 1D6Q 1D6R 1D6S 1D6U 1D6V 1D6W 1D6Y 1D6Z 1D7A 1D7B 1D7C 1D7D 1D7E 1D7F 1D7H 1D7I 1D7J 1D7K 1D7L 1D7M 1D7O 1D7P 1D7R 1D7S 1D7U 1D7V 1D7W 1D7X 1D7Y 1D8A 1D8C 1D8D 1D8E 1D8F 1D8H 1D8I 1D8L 1D8M 1D8S 1D8T 1D8U 1D8W 1D9C 1D9E 1D9G 1D9I 1D9K 1D9Q 1D9U 1D9V 1D9W 1D9X 1D9Y 1D9Z 1DAA 1DAB 1DAD 1DAE 1DAF 1DAG 1DAH 1DAI 1DAJ 1DAK 1DAM 1DAN 1DAO 1DAP 1DAR 1DAT 1DAW 1DAY 1DAZ 1DB1 1DB2 1DB3 1DB4 1DB5 1DBA 1DBB 1DBF 1DBG 1DBH 1DBI 1DBJ 1DBK 1DBM 1DBN 1DBO 1DBP 1DBQ 1DBR 1DBS 1DBT 1DBU 1DBV 1DBW 1DBX 1DBZ 1DC3 1DC4 1DC5 1DC6 1DC9 1DCA 1DCB 1DCC 1DCD 1DCE 1DCF 1DCH 1DCI 1DCK 1DCL 1DCM 1DCN 1DCO 1DCP 1DCQ 1DCS 1DCU 1DCY 1DD1 1DD3 1DD4 1DD5 1DD6 1DD7 1DD8 1DD9 1DDE 1DDG 1DDH 1DDI 1DDJ 1DDK 1DDO 1DDR 1DDS 1DDT 1DDU 1DDV 1DDW 1DDX 1DDZ 1DE0 1DE4 1DE5 1DE6 1DE7 1DEA 1DEB 1DED 1DEE 1DEG 1DEH 1DEI 1DEJ 1DEK 1DEL 1DEO 1DEQ 1DET 1DEU 1DEV 1DEX 1DF0 1DF1 1DF4 1DF5 1DF7 1DF8 1DFA 1DFB 1DFC 1DFF 1DFG 1DFH 1DFI 1DFJ 1DFK 1DFL 1DFN 1DFO 1DFP 1DFQ 1DFV 1DFX 1DG1 1DG3 1DG5 1DG6 1DG7 1DG8 1DG9 1DGB 1DGD 1DGE 1DGF 1DGG 1DGH 1DGJ 1DGK 1DGL 1DGM 1DGP 1DGR 1DGS 1DGW 1DHF 1DHG 1DHI 1DHJ 1DHK 1DHN 1DHP 1DHR 1DHS 1DHT 1DHY 1DI0 1DI1 1DI3 1DI4 1DI5 1DI6 1DI7 1DI8 1DI9 1DIA 1DIB 1DIC 1DID 1DIE 1DIF 1DIG 1DIH 1DII 1DIK 1DIL 1DIM 1DIN 1DIO 1DIQ 1DIR 1DIT 1DIV 1DIW 1DIX 1DIY 1DJ0 1DJ1 1DJ2 1DJ3 1DJ5 1DJ7 1DJ8 1DJ9 1DJA 1DJB 1DJC 1DJE 1DJG 1DJH 1DJI 1DJL 1DJN 1DJO 1DJP 1DJQ 1DJR 1DJS 1DJT 1DJU 1DJW 1DJX 1DJY 1DJZ 1DK0 1DK4 1DK5 1DK7 1DK8 1DKA 1DKD 1DKE 1DKF 1DKG 1DKH 1DKI 1DKJ 1DKK 1DKL 1DKM 1DKN 1DKO 1DKP 1DKQ 1DKR 1DKS 1DKT 1DKU 1DKW 1DKX 1DKY 1DKZ 1DL2 1DL3 1DL5 1DL7 1DLA 1DLB 1DLC 1DLE 1DLF 1DLG 1DLH 1DLI 1DLJ 1DLK 1DLL 1DLM 1DLO 1DLP 1DLQ 1DLR 1DLS 1DLT 1DLU 1DLV 1DLW 1DLY 1DM0 1DM1 1DM2 1DM3 1DM4 1DM5 1DM6 1DM7 1DM8 1DM9 1DMA 1DMB 1DMG 1DMH 1DMI 1DMJ 1DMK 1DML 1DMM 1DMN 1DMP 1DMQ 1DMR 1DMS 1DMT 1DMW 1DMX 1DMY 1DN0 1DN2 1DNA 1DNC 1DNL 1DNP 1DNU 1DNV 1DNW 1DO0 1DO1 1DO2 1DO3 1DO4 1DO5 1DO6 1DO7 1DO8 1DOA 1DOB 1DOC 1DOD 1DOE 1DOF 1DOG 1DOH 1DOI 1DOJ 1DOK 1DOL 1DOR 1DOS 1DOT 1DOV 1DOW 1DOZ 1DP0 1DP2 1DP4 1DP5 1DP6 1DP8 1DP9 1DPB 1DPC 1DPD 1DPE 1DPF 1DPG 1DPH 1DPI 1DPJ 1DPM 1DPO 1DPP 1DPR 1DPS 1DPT 1DPW 1DPX 1DPY 1DPZ 1DQ0 1DQ1 1DQ2 1DQ3 1DQ4 1DQ5 1DQ6 1DQ7 1DQ8 1DQ9 1DQA 1DQD 1DQE 1DQG 1DQI 1DQJ 1DQK 1DQL 1DQM 1DQN 1DQO 1DQP 1DQQ 1DQR 1DQS 1DQT 1DQU 1DQV 1DQW 1DQX 1DQY 1DQZ 1DR0 1DR1 1DR2 1DR3 1DR4 1DR5 1DR6 1DR7 1DR8 1DR9 1DRA 1DRB 1DRE 1DRF 1DRH 1DRJ 1DRK 1DRM 1DRT 1DRU 1DRV 1DRW 1DRY 1DS0 1DS1 1DS2 1DS3 1DS4 1DS5 1DS6 1DS7 1DS8 1DSB 1DSE 1DSF 1DSG 1DSL 1DSN 1DSO 1DSP 1DSS 1DST 1DSU 1DSX 1DSY 1DT0 1DT1 1DT2 1DT3 1DT4 1DT5 1DT6 1DT9 1DTD 1DTE 1DTG 1DTH 1DTI 1DTJ 1DTL 1DTM 1DTN 1DTO 1DTP 1DTQ 1DTS 1DTT 1DTU 1DTW 1DTX 1DTY 1DTZ 1DU3 1DU4 1DU5 1DUA 1DUB 1DUC 1DUD 1DUE 1DUG 1DUI 1DUK 1DUN 1DUO 1DUP 1DUR 1DUS 1DUT 1DUV 1DUW 1DUY 1DUZ 1DV1 1DV2 1DV3 1DV6 1DV7 1DV8 1DVA 1DVB 1DVE 1DVF 1DVG 1DVI 1DVJ 1DVK 1DVM 1DVN 1DVO 1DVP 1DVQ 1DVR 1DVS 1DVT 1DVU 1DVX 1DVY 1DVZ 1DW0 1DW1 1DW2 1DW3 1DW6 1DW9 1DWA 1DWB 1DWC 1DWD 1DWE 1DWF 1DWG 1DWH 1DWI 1DWJ 1DWK 1DWN 1DWO 1DWP 1DWQ 1DWR 1DWS 1DWT 1DWU 1DWV 1DWW 1DWX 1DX4 1DX5 1DX6 1DX9 1DXC 1DXD 1DXE 1DXF 1DXG 1DXH 1DXI 1DXJ 1DXK 1DXL 1DXM 1DXO 1DXP 1DXQ 1DXR 1DXS 1DXT 1DXU 1DXV 1DXX 1DXY 1DY0 1DY1 1DY2 1DY3 1DY4 1DY5 1DY6 1DY7 1DY8 1DY9 1DYA 1DYB 1DYC 1DYD 1DYE 1DYF 1DYG 1DYH 1DYI 1DYJ 1DYK 1DYM 1DYN 1DYO 1DYP 1DYQ 1DYR 1DYS 1DYT 1DYU 1DYW 1DYZ 1DZ0 1DZ3 1DZ4 1DZ6 1DZ8 1DZ9 1DZA 1DZB 1DZE 1DZF 1DZG 1DZH 1DZI 1DZJ 1DZK 1DZL 1DZM 1DZN 1DZO 1DZP 1DZQ 1DZR 1DZT 1DZU 1DZV 1DZW 1DZX 1DZY 1DZZ 1E00 1E02 1E03 1E04 1E05 1E06 1E0B 1E0C 1E0D 1E0F 1E0J 1E0K 1E0O 1E0P 1E0R 1E0S 1E0T 1E0U 1E0V 1E0W 1E0X 1E0Y 1E12 1E14 1E15 1E18 1E19 1E1A 1E1C 1E1D 1E1E 1E1F 1E1H 1E1K 1E1L 1E1M 1E1N 1E1O 1E1Q 1E1R 1E1T 1E1V 1E1X 1E1Y 1E1Z 1E20 1E21 1E22 1E24 1E25 1E26 1E27 1E28 1E29 1E2A 1E2D 1E2E 1E2F 1E2G 1E2H 1E2I 1E2J 1E2K 1E2L 1E2M 1E2N 1E2O 1E2P 1E2Q 1E2R 1E2S 1E2T 1E2U 1E2V 1E2W 1E2X 1E2Y 1E2Z 1E30 1E31 1E32 1E33 1E34 1E35 1E36 1E37 1E38 1E39 1E3A 1E3B 1E3C 1E3D 1E3E 1E3F 1E3G 1E3H 1E3I 1E3J 1E3K 1E3L 1E3P 1E3Q 1E3R 1E3S 1E3U 1E3V 1E3W 1E3X 1E3Z 1E40 1E42 1E43 1E44 1E46 1E47 1E48 1E49 1E4A 1E4B 1E4C 1E4D 1E4E 1E4F 1E4G 1E4H 1E4I 1E4J 1E4K 1E4L 1E4M 1E4N 1E4O 1E4V 1E4W 1E4X 1E4Y 1E50 1E51 1E54 1E55 1E56 1E57 1E58 1E59 1E5A 1E5D 1E5E 1E5F 1E5H 1E5I 1E5J 1E5K 1E5L 1E5M 1E5N 1E5O 1E5P 1E5Q 1E5R 1E5S 1E5T 1E5V 1E5W 1E5X 1E5Y 1E5Z 1E60 1E61 1E62 1E63 1E64 1E65 1E66 1E67 1E69 1E6A 1E6B 1E6C 1E6D 1E6E 1E6F 1E6G 1E6H 1E6I 1E6J 1E6K 1E6L 1E6M 1E6N 1E6O 1E6P 1E6Q 1E6R 1E6S 1E6U 1E6V 1E6W 1E6X 1E6Y 1E6Z 1E70 1E71 1E72 1E73 1E77 1E78 1E79 1E7A 1E7B 1E7C 1E7D 1E7E 1E7F 1E7G 1E7H 1E7I 1E7L 1E7M 1E7N 1E7O 1E7P 1E7Q 1E7R 1E7S 1E7U 1E7V 1E7W 1E7Y 1E7Z 1E80 1E81 1E82 1E83 1E84 1E85 1E86 1E87 1E89 1E8A 1E8C 1E8D 1E8F 1E8G 1E8H 1E8I 1E8K 1E8M 1E8N 1E8T 1E8U 1E8V 1E8W 1E8X 1E8Y 1E8Z 1E90 1E92 1E93 1E94 1E96 1E97 1E98 1E99 1E9A 1E9B 1E9C 1E9D 1E9E 1E9F 1E9G 1E9H 1E9I 1E9L 1E9M 1E9N 1E9O 1E9P 1E9Q 1E9R 1E9S 1E9V 1E9W 1E9X 1E9Y 1E9Z 1EA0 1EA1 1EA2 1EA3 1EA5 1EA6 1EA7 1EA8 1EA9 1EAA 1EAB 1EAC 1EAD 1EAE 1EAF 1EAG 1EAH 1EAI 1EAJ 1EAK 1EAM 1EAN 1EAO 1EAP 1EAQ 1EAR 1EAS 1EAT 1EAU 1EAV 1EAW 1EAX 1EAY 1EAZ 1EB0 1EB1 1EB2 1EB3 1EB4 1EB6 1EB7 1EB8 1EB9 1EBA 1EBB 1EBC 1EBD 1EBE 1EBF 1EBG 1EBH 1EBK 1EBL 1EBO 1EBP 1EBT 1EBU 1EBV 1EBW 1EBY 1EBZ 1EC0 1EC1 1EC2 1EC3 1EC5 1EC7 1EC8 1EC9 1ECA 1ECB 1ECC 1ECD 1ECE 1ECF 1ECG 1ECJ 1ECL 1ECM 1ECN 1ECO 1ECP 1ECQ 1ECS 1ECV 1ECW 1ECX 1ECY 1ECZ 1ED1 1ED3 1ED4 1ED5 1ED6 1ED8 1ED9 1EDB 1EDD 1EDE 1EDG 1EDH 1EDM 1EDN 1EDO 1EDQ 1EDT 1EDU 1EDY 1EDZ 1EE0 1EE1 1EE2 1EE3 1EE4 1EE5 1EE6 1EE8 1EE9 1EEA 1EED 1EEF 1EEH 1EEI 1EEJ 1EEM 1EEN 1EEO 1EEP 1EEQ 1EER 1EET 1EEU 1EEX 1EEY 1EEZ 1EF0 1EF1 1EF2 1EF3 1EF7 1EF8 1EF9 1EFC 1EFD 1EFG 1EFH 1EFI 1EFK 1EFL 1EFM 1EFN 1EFP 1EFQ 1EFR 1EFT 1EFU 1EFV 1EFX 1EFY 1EFZ 1EG1 1EG2 1EG3 1EG4 1EG5 1EG7 1EG9 1EGA 1EGC 1EGD 1EGE 1EGG 1EGH 1EGI 1EGJ 1EGM 1EGN 1EGP 1EGQ 1EGU 1EGV 1EGY 1EGZ 1EH1 1EH3 1EH4 1EH5 1EH6 1EH7 1EH8 1EH9 1EHA 1EHB 1EHC 1EHD 1EHE 1EHF 1EHG 1EHH 1EHI 1EHK 1EHN 1EHW 1EHY 1EI1 1EI3 1EI5 1EI6 1EI7 1EI8 1EI9 1EIA 1EIB 1EIC 1EID 1EIE 1EIF 1EIL 1EIN 1EIQ 1EIR 1EIS 1EIX 1EIZ 1EJ0 1EJ1 1EJ2 1EJ3 1EJ4 1EJ6 1EJ7 1EJ8 1EJA 1EJB 1EJC 1EJD 1EJE 1EJF 1EJG 1EJH 1EJI 1EJJ 1EJL 1EJM 1EJN 1EJO 1EJR 1EJS 1EJT 1EJU 1EJV 1EJW 1EJX 1EJY 1EK0 1EK1 1EK2 1EK3 1EK4 1EK5 1EK6 1EK8 1EK9 1EKB 1EKE 1EKF 1EKG 1EKJ 1EKK 1EKL 1EKM 1EKO 1EKP 1EKQ 1EKR 1EKS 1EKU 1EKV 1EKX 1EL1 1EL3 1EL4 1EL5 1EL6 1EL7 1EL8 1EL9 1ELA 1ELB 1ELC 1ELD 1ELE 1ELF 1ELG 1ELI 1ELJ 1ELK 1ELL 1ELM 1ELO 1ELP 1ELQ 1ELR 1ELS 1ELT 1ELU 1ELV 1ELW 1ELX 1ELY 1ELZ 1EM1 1EM2 1EM6 1EM7 1EM8 1EM9 1EMA 1EMB 1EMC 1EMD 1EME 1EMF 1EMG 1EMK 1EML 1EMM 1EMR 1EMS 1EMT 1EMU 1EMV 1EMY 1EN2 1EN4 1EN5 1EN6 1EN7 1ENA 1ENC 1ENF 1ENH 1ENI 1ENJ 1ENK 1ENM 1ENO 1ENP 1ENQ 1ENR 1ENS 1ENT 1ENU 1ENV 1ENX 1ENY 1ENZ 1EO2 1EO5 1EO6 1EO7 1EO8 1EO9 1EOA 1EOB 1EOC 1EOD 1EOE 1EOF 1EOG 1EOH 1EOI 1EOJ 1EOK 1EOL 1EOM 1EOS 1EOU 1EOV 1EOW 1EP0 1EP1 1EP2 1EP3 1EP4 1EP5 1EP6 1EP7 1EP8 1EP9 1EPA 1EPB 1EPF 1EPL 1EPM 1EPN 1EPO 1EPP 1EPQ 1EPR 1EPS 1EPT 1EPU 1EPV 1EPW 1EPX 1EPY 1EPZ 1EQ2 1EQ4 1EQ5 1EQ6 1EQ7 1EQ9 1EQA 1EQB 1EQC 1EQD 1EQE 1EQF 1EQG 1EQH 1EQJ 1EQM 1EQN 1EQP 1EQR 1EQT 1EQU 1EQV 1EQW 1EQY 1ER8 1ERB 1ERE 1ERJ 1ERK 1ERM 1ERN 1ERO 1ERQ 1ERR 1ERT 1ERU 1ERV 1ERW 1ERX 1ERZ 1ES0 1ES1 1ES2 1ES3 1ES4 1ES5 1ES6 1ES7 1ES8 1ES9 1ESA 1ESB 1ESC 1ESD 1ESE 1ESF 1ESI 1ESJ 1ESL 1ESM 1ESN 1ESO 1ESP 1ESQ 1ESR 1EST 1ESU 1ESV 1ESW 1ESZ 1ET0 1ET1 1ET5 1ET6 1ET7 1ET8 1ET9 1ETA 1ETB 1ETE 1ETH 1ETJ 1ETK 1ETL 1ETM 1ETN 1ETO 1ETP 1ETQ 1ETR 1ETS 1ETT 1ETU 1ETV 1ETW 1ETX 1ETY 1ETZ 1EU1 1EU3 1EU4 1EU5 1EU8 1EUA 1EUC 1EUD 1EUE 1EUF 1EUG 1EUH 1EUI 1EUJ 1EUM 1EUN 1EUO 1EUP 1EUR 1EUS 1EUT 1EUU 1EUV 1EUW 1EUZ 1EV1 1EV2 1EV3 1EV4 1EV5 1EV6 1EV7 1EV8 1EV9 1EVE 1EVF 1EVG 1EVH 1EVI 1EVJ 1EVK 1EVL 1EVQ 1EVR 1EVS 1EVT 1EVU 1EVX 1EVY 1EVZ 1EW0 1EW2 1EW3 1EW4 1EW6 1EW8 1EW9 1EWA 1EWC 1EWD 1EWE 1EWF 1EWH 1EWJ 1EWK 1EWL 1EWM 1EWO 1EWP 1EWR 1EWT 1EWV 1EWX 1EWY 1EWZ 1EX0 1EX1 1EX2 1EX3 1EX4 1EX5 1EX6 1EX7 1EX8 1EX9 1EXA 1EXB 1EXC 1EXF 1EXM 1EXN 1EXP 1EXQ 1EXR 1EXS 1EXT 1EXU 1EXV 1EXW 1EXX 1EXZ 1EY0 1EY2 1EY3 1EY4 1EY5 1EY6 1EY7 1EY8 1EY9 1EYA 1EYB 1EYC 1EYD 1EYE 1EYH 1EYI 1EYJ 1EYK 1EYL 1EYM 1EYN 1EYP 1EYQ 1EYR 1EYS 1EYT 1EYV 1EYW 1EYX 1EYY 1EYZ 1EZ0 1EZ1 1EZ2 1EZ3 1EZ4 1EZ6 1EZ8 1EZ9 1EZF 1EZG 1EZI 1EZJ 1EZK 1EZL 1EZM 1EZQ 1EZR 1EZS 1EZU 1EZV 1EZW 1EZX 1EZZ 1F00 1F02 1F05 1F06 1F07 1F08 1F09 1F0B 1F0C 1F0I 1F0J 1F0K 1F0L 1F0M 1F0N 1F0P 1F0Q 1F0R 1F0S 1F0T 1F0U 1F0W 1F0X 1F0Y 1F10 1F11 1F12 1F13 1F14 1F15 1F17 1F18 1F1A 1F1B 1F1C 1F1D 1F1E 1F1F 1F1G 1F1H 1F1J 1F1M 1F1O 1F1R 1F1S 1F1U 1F1V 1F1W 1F1X 1F1Z 1F20 1F21 1F23 1F24 1F25 1F26 1F28 1F29 1F2A 1F2B 1F2C 1F2D 1F2E 1F2F 1F2J 1F2K 1F2L 1F2M 1F2N 1F2O 1F2P 1F2Q 1F2S 1F2T 1F2U 1F2V 1F2W 1F2X 1F2Y 1F2Z 1F30 1F31 1F32 1F33 1F34 1F35 1F36 1F37 1F38 1F39 1F3A 1F3B 1F3D 1F3E 1F3F 1F3G 1F3H 1F3J 1F3L 1F3M 1F3O 1F3P 1F3T 1F3U 1F3V 1F3W 1F3X 1F3Z 1F41 1F42 1F45 1F46 1F47 1F48 1F4A 1F4B 1F4C 1F4D 1F4E 1F4F 1F4G 1F4H 1F4J 1F4L 1F4M 1F4N 1F4O 1F4P 1F4Q 1F4T 1F4U 1F4V 1F4W 1F4X 1F4Y 1F4Z 1F50 1F51 1F52 1F56 1F57 1F58 1F59 1F5A 1F5B 1F5C 1F5F 1F5J 1F5K 1F5L 1F5M 1F5N 1F5O 1F5P 1F5Q 1F5R 1F5S 1F5V 1F5W 1F5Z 1F60 1F61 1F63 1F65 1F6A 1F6B 1F6D 1F6F 1F6K 1F6L 1F6M 1F6N 1F6P 1F6R 1F6S 1F6T 1F6W 1F6Y 1F73 1F74 1F75 1F76 1F77 1F7A 1F7B 1F7C 1F7D 1F7K 1F7L 1F7N 1F7O 1F7P 1F7Q 1F7R 1F7S 1F7T 1F7Z 1F80 1F82 1F86 1F88 1F89 1F8A 1F8B 1F8C 1F8D 1F8E 1F8F 1F8G 1F8I 1F8M 1F8N 1F8Q 1F8R 1F8S 1F8T 1F8U 1F8W 1F8X 1F8Y 1F90 1F91 1F92 1F93 1F94 1F97 1F98 1F99 1F9A 1F9B 1F9C 1F9D 1F9E 1F9F 1F9G 1F9H 1F9I 1F9J 1F9K 1F9M 1F9N 1F9O 1F9P 1F9Q 1F9R 1F9S 1F9T 1F9U 1F9V 1F9W 1F9Z 1FA0 1FA2 1FA5 1FA6 1FA7 1FA8 1FA9 1FAA 1FAE 1FAG 1FAH 1FAI 1FAJ 1FAK 1FAN 1FAO 1FAP 1FAS 1FAT 1FAV 1FAW 1FAX 1FAY 1FAZ 1FB0 1FB1 1FB2 1FB5 1FB6 1FB7 1FB8 1FBA 1FBC 1FBD 1FBE 1FBF 1FBG 1FBH 1FBI 1FBL 1FBM 1FBN 1FBO 1FBP 1FBQ 1FBS 1FBT 1FBU 1FBV 1FBW 1FBX 1FBY 1FBZ 1FC0 1FC1 1FC2 1FC3 1FC4 1FC5 1FC6 1FC7 1FC9 1FCA 1FCB 1FCC 1FCD 1FCE 1FCF 1FCG 1FCH 1FCJ 1FCK 1FCM 1FCN 1FCO 1FCP 1FCQ 1FCS 1FCU 1FCV 1FCX 1FCY 1FCZ 1FD0 1FD2 1FD3 1FD4 1FD7 1FD9 1FDA 1FDB 1FDD 1FDH 1FDI 1FDJ 1FDK 1FDL 1FDN 1FDO 1FDP 1FDQ 1FDR 1FDS 1FDT 1FDU 1FDV 1FDW 1FDY 1FDZ 1FE0 1FE1 1FE2 1FE3 1FE4 1FE5 1FE6 1FE8 1FEA 1FEB 1FEC 1FEE 1FEH 1FEJ 1FEL 1FEM 1FEN 1FEP 1FER 1FEV 1FEW 1FEZ 1FF0 1FF2 1FF3 1FF4 1FF5 1FF9 1FFA 1FFB 1FFC 1FFD 1FFE 1FFF 1FFG 1FFH 1FFI 1FFL 1FFN 1FFO 1FFP 1FFQ 1FFR 1FFS 1FFT 1FFU 1FFV 1FFW 1FFX 1FG2 1FG3 1FG4 1FG5 1FG6 1FG7 1FG8 1FG9 1FGA 1FGB 1FGC 1FGG 1FGH 1FGI 1FGJ 1FGK 1FGL 1FGM 1FGN 1FGO 1FGQ 1FGR 1FGS 1FGT 1FGU 1FGV 1FGX 1FGY 1FGZ 1FH0 1FH2 1FH5 1FH7 1FH8 1FH9 1FHA 1FHD 1FHE 1FHF 1FHG 1FHH 1FHI 1FHJ 1FHL 1FHM 1FHN 1FHU 1FHV 1FHW 1FHX 1FI1 1FI2 1FI4 1FI8 1FIA 1FIB 1FIC 1FID 1FIE 1FIF 1FIG 1FIH 1FIK 1FIL 1FIM 1FIN 1FIO 1FIP 1FIQ 1FIT 1FIV 1FIW 1FIY 1FIZ 1FJ0 1FJ1 1FJ2 1FJ3 1FJ4 1FJ6 1FJ8 1FJ9 1FJH 1FJJ 1FJM 1FJO 1FJQ 1FJR 1FJS 1FJT 1FJU 1FJV 1FJW 1FK0 1FK1 1FK2 1FK3 1FK4 1FK5 1FK6 1FK7 1FK8 1FK9 1FKB 1FKD 1FKF 1FKG 1FKH 1FKI 1FKJ 1FKK 1FKL 1FKM 1FKN 1FKO 1FKP 1FKQ 1FKV 1FKW 1FKX 1FL0 1FL1 1FL2 1FL3 1FL5 1FL6 1FL7 1FL9 1FLA 1FLC 1FLD 1FLE 1FLG 1FLH 1FLJ 1FLK 1FLL 1FLM 1FLN 1FLP 1FLQ 1FLR 1FLT 1FLU 1FLV 1FLW 1FLY 1FLZ 1FM0 1FM2 1FM4 1FM5 1FM6 1FM7 1FM8 1FM9 1FMA 1FMB 1FMC 1FMD 1FMG 1FMI 1FMJ 1FMK 1FML 1FMO 1FMT 1FMU 1FMV 1FMW 1FMX 1FMZ 1FN0 1FN3 1FN4 1FN5 1FN6 1FN8 1FN9 1FNA 1FNB 1FNC 1FND 1FNE 1FNF 1FNG 1FNH 1FNI 1FNJ 1FNK 1FNL 1FNM 1FNN 1FNO 1FNP 1FNQ 1FNS 1FNT 1FNU 1FNV 1FNW 1FNY 1FNZ 1FO0 1FO1 1FO2 1FO3 1FO4 1FO6 1FO8 1FO9 1FOA 1FOB 1FOC 1FOD 1FOE 1FOF 1FOH 1FOI 1FOJ 1FOL 1FON 1FOO 1FOP 1FOR 1FOT 1FOU 1FP1 1FP2 1FP3 1FP4 1FP5 1FP6 1FP7 1FP8 1FP9 1FPB 1FPC 1FPD 1FPE 1FPF 1FPG 1FPH 1FPI 1FPJ 1FPK 1FPL 1FPM 1FPN 1FPO 1FPP 1FPQ 1FPR 1FPS 1FPT 1FPU 1FPV 1FPX 1FPY 1FPZ 1FQ0 1FQ1 1FQ3 1FQ4 1FQ5 1FQ6 1FQ7 1FQ8 1FQ9 1FQA 1FQB 1FQC 1FQD 1FQE 1FQF 1FQG 1FQI 1FQJ 1FQK 1FQL 1FQM 1FQN 1FQO 1FQR 1FQT 1FQV 1FQW 1FQX 1FR1 1FR2 1FR3 1FR4 1FR5 1FR6 1FR7 1FR8 1FR9 1FRB 1FRD 1FRF 1FRG 1FRH 1FRI 1FRJ 1FRK 1FRL 1FRM 1FRN 1FRO 1FRP 1FRQ 1FRR 1FRS 1FRT 1FRV 1FRW 1FRX 1FRZ 1FS0 1FS1 1FS2 1FS3 1FS4 1FS5 1FS6 1FS7 1FS8 1FS9 1FSA 1FSC 1FSE 1FSF 1FSG 1FSI 1FSJ 1FSK 1FSL 1FSN 1FSO 1FSQ 1FSR 1FSS 1FST 1FSU 1FSW 1FSX 1FSY 1FSZ 1FT0 1FT1 1FT2 1FT3 1FT4 1FT5 1FT6 1FT7 1FT8 1FT9 1FTA 1FTC 1FTE 1FTF 1FTG 1FTH 1FTJ 1FTK 1FTL 1FTM 1FTN 1FTO 1FTP 1FTQ 1FTR 1FTS 1FTW 1FTX 1FTY 1FU0 1FU1 1FU4 1FU7 1FU8 1FUA 1FUE 1FUG 1FUI 1FUJ 1FUK 1FUN 1FUO 1FUP 1FUQ 1FUR 1FUS 1FUT 1FUU 1FUX 1FUY 1FV0 1FV1 1FV2 1FV3 1FV9 1FVA 1FVC 1FVD 1FVE 1FVF 1FVG 1FVH 1FVI 1FVJ 1FVK 1FVM 1FVO 1FVP 1FVR 1FVT 1FVU 1FVV 1FVX 1FW0 1FW1 1FW2 1FW3 1FW4 1FW8 1FW9 1FWA 1FWB 1FWC 1FWD 1FWE 1FWF 1FWG 1FWH 1FWI 1FWJ 1FWK 1FWL 1FWM 1FWN 1FWR 1FWS 1FWT 1FWU 1FWV 1FWW 1FWX 1FWY 1FWZ 1FX0 1FX1 1FX2 1FX3 1FX4 1FX5 1FX6 1FX7 1FX8 1FX9 1FXA 1FXD 1FXF 1FXH 1FXI 1FXJ 1FXK 1FXO 1FXP 1FXQ 1FXR 1FXS 1FXU 1FXV 1FXW 1FXX 1FXY 1FXZ 1FY1 1FY2 1FY3 1FY4 1FY5 1FY6 1FY7 1FY8 1FY9 1FYA 1FYD 1FYE 1FYF 1FYH 1FYN 1FYR 1FYS 1FYT 1FYU 1FYV 1FYW 1FYX 1FYZ 1FZ0 1FZ1 1FZ2 1FZ3 1FZ4 1FZ5 1FZ6 1FZ7 1FZ8 1FZ9 1FZA 1FZB 1FZC 1FZD 1FZE 1FZF 1FZG 1FZH 1FZI 1FZJ 1FZK 1FZM 1FZO 1FZQ 1FZR 1FZU 1FZV 1FZW 1FZY 1FZZ 1G01 1G02 1G05 1G06 1G07 1G08 1G09 1G0A 1G0B 1G0C 1G0D 1G0E 1G0F 1G0G 1G0H 1G0I 1G0J 1G0K 1G0L 1G0M 1G0N 1G0O 1G0P 1G0Q 1G0R 1G0S 1G0T 1G0U 1G0V 1G0W 1G0X 1G0Y 1G0Z 1G12 1G13 1G15 1G16 1G17 1G18 1G19 1G1A 1G1B 1G1C 1G1D 1G1F 1G1G 1G1H 1G1I 1G1J 1G1K 1G1L 1G1M 1G1O 1G1Q 1G1R 1G1S 1G1T 1G1U 1G1V 1G1W 1G1Y 1G20 1G21 1G23 1G24 1G27 1G28 1G29 1G2A 1G2B 1G2C 1G2I 1G2K 1G2L 1G2M 1G2N 1G2O 1G2P 1G2Q 1G2R 1G2U 1G2V 1G2W 1G2X 1G2Y 1G2Z 1G30 1G31 1G32 1G33 1G35 1G36 1G37 1G39 1G3B 1G3C 1G3D 1G3E 1G3I 1G3J 1G3K 1G3L 1G3M 1G3N 1G3O 1G3P 1G3Q 1G3R 1G3S 1G3T 1G3U 1G3W 1G3Y 1G3Z 1G40 1G41 1G42 1G43 1G44 1G45 1G46 1G48 1G49 1G4A 1G4B 1G4C 1G4E 1G4H 1G4I 1G4J 1G4K 1G4M 1G4O 1G4P 1G4R 1G4S 1G4T 1G4U 1G4V 1G4W 1G4X 1G4Y 1G50 1G51 1G52 1G53 1G54 1G55 1G57 1G58 1G5A 1G5B 1G5C 1G5F 1G5G 1G5H 1G5I 1G5N 1G5P 1G5Q 1G5R 1G5S 1G5T 1G5U 1G5X 1G5Y 1G5Z 1G60 1G61 1G62 1G63 1G64 1G65 1G66 1G67 1G68 1G69 1G6A 1G6B 1G6C 1G6G 1G6H 1G6I 1G6K 1G6L 1G6N 1G6O 1G6Q 1G6R 1G6S 1G6T 1G6U 1G6V 1G6W 1G6X 1G6Y 1G71 1G72 1G73 1G74 1G76 1G77 1G78 1G79 1G7A 1G7B 1G7C 1G7F 1G7G 1G7H 1G7I 1G7J 1G7K 1G7L 1G7M 1G7N 1G7P 1G7Q 1G7R 1G7S 1G7T 1G7U 1G7V 1G7W 1G7X 1G7Y 1G81 1G82 1G83 1G85 1G86 1G87 1G88 1G8A 1G8E 1G8F 1G8G 1G8H 1G8I 1G8J 1G8K 1G8L 1G8M 1G8O 1G8P 1G8Q 1G8R 1G8S 1G8T 1G8W 1G8X 1G8Y 1G8Z 1G93 1G94 1G95 1G96 1G97 1G98 1G99 1G9A 1G9B 1G9C 1G9D 1G9F 1G9G 1G9H 1G9I 1G9J 1G9K 1G9M 1G9N 1G9O 1G9Q 1G9R 1G9S 1G9T 1G9U 1G9V 1G9W 1G9X 1GA0 1GA1 1GA2 1GA4 1GA6 1GA7 1GA8 1GA9 1GAD 1GAE 1GAF 1GAG 1GAH 1GAI 1GAJ 1GAK 1GAL 1GAM 1GAN 1GAO 1GAQ 1GAR 1GAV 1GAW 1GAY 1GAZ 1GB0 1GB2 1GB3 1GB5 1GB6 1GB7 1GB8 1GB9 1GBA 1GBB 1GBC 1GBD 1GBE 1GBF 1GBG 1GBH 1GBI 1GBJ 1GBK 1GBL 1GBM 1GBN 1GBO 1GBS 1GBT 1GBU 1GBV 1GBW 1GBX 1GBY 1GBZ 1GC0 1GC1 1GC2 1GC3 1GC4 1GC5 1GC6 1GC7 1GC8 1GC9 1GCA 1GCB 1GCD 1GCE 1GCG 1GCI 1GCJ 1GCK 1GCL 1GCM 1GCN 1GCO 1GCP 1GCQ 1GCS 1GCT 1GCU 1GCV 1GCW 1GCY 1GCZ 1GD0 1GD1 1GD6 1GD7 1GD8 1GD9 1GDD 1GDE 1GDH 1GDI 1GDJ 1GDK 1GDL 1GDN 1GDQ 1GDR 1GDU 1GDV 1GDW 1GDX 1GE0 1GE1 1GE2 1GE3 1GE4 1GE5 1GE6 1GE7 1GE8 1GEB 1GEC 1GED 1GEE 1GEF 1GEG 1GEH 1GEI 1GEJ 1GEK 1GEM 1GEN 1GEQ 1GER 1GES 1GET 1GEU 1GEV 1GEW 1GEX 1GEY 1GEZ 1GF0 1GF3 1GF4 1GF5 1GF6 1GF7 1GF8 1GF9 1GFA 1GFE 1GFF 1GFG 1GFH 1GFI 1GFJ 1GFK 1GFL 1GFM 1GFN 1GFO 1GFP 1GFQ 1GFR 1GFS 1GFT 1GFU 1GFV 1GFW 1GFY 1GFZ 1GG0 1GG1 1GG2 1GG3 1GG4 1GG5 1GG6 1GG8 1GG9 1GGB 1GGC 1GGD 1GGE 1GGF 1GGG 1GGH 1GGI 1GGJ 1GGK 1GGL 1GGM 1GGN 1GGO 1GGP 1GGQ 1GGT 1GGU 1GGV 1GGX 1GGY 1GGZ 1GH0 1GH2 1GH4 1GH6 1GH7 1GHA 1GHB 1GHD 1GHE 1GHF 1GHG 1GHI 1GHL 1GHM 1GHP 1GHQ 1GHR 1GHS 1GHV 1GHW 1GHX 1GHY 1GHZ 1GI0 1GI1 1GI2 1GI3 1GI4 1GI5 1GI6 1GI7 1GI8 1GI9 1GIA 1GIC 1GIF 1GIG 1GIH 1GII 1GIJ 1GIK 1GIL 1GIM 1GIN 1GIQ 1GIR 1GIS 1GIT 1GIU 1GJ4 1GJ5 1GJ6 1GJ7 1GJ8 1GJ9 1GJA 1GJB 1GJC 1GJD 1GJM 1GJN 1GJO 1GJP 1GJQ 1GJR 1GJU 1GJV 1GJW 1GJY 1GK0 1GK1 1GK2 1GK3 1GK4 1GK6 1GK7 1GK8 1GK9 1GKA 1GKB 1GKC 1GKD 1GKE 1GKF 1GKH 1GKI 1GKJ 1GKK 1GKL 1GKM 1GKO 1GKP 1GKQ 1GKR 1GKU 1GKX 1GKY 1GKZ 1GL0 1GL1 1GL2 1GL3 1GL4 1GL6 1GL7 1GL9 1GLA 1GLB 1GLC 1GLD 1GLE 1GLF 1GLG 1GLH 1GLI 1GLJ 1GLL 1GLM 1GLN 1GLO 1GLP 1GLQ 1GLV 1GM4 1GM6 1GM7 1GM8 1GM9 1GMB 1GMC 1GMD 1GME 1GMG 1GMH 1GMI 1GMJ 1GMK 1GML 1GMM 1GMN 1GMO 1GMP 1GMQ 1GMR 1GMU 1GMV 1GMW 1GMX 1GMY 1GMZ 1GN0 1GN1 1GN2 1GN3 1GN4 1GN6 1GN8 1GN9 1GND 1GNE 1GNG 1GNH 1GNI 1GNJ 1GNK 1GNL 1GNM 1GNN 1GNO 1GNP 1GNQ 1GNR 1GNS 1GNT 1GNU 1GNV 1GNW 1GNX 1GNY 1GNZ 1GO2 1GO3 1GO4 1GO6 1GO7 1GO8 1GOA 1GOB 1GOC 1GOD 1GOF 1GOG 1GOH 1GOI 1GOJ 1GOK 1GOL 1GOM 1GON 1GOO 1GOQ 1GOR 1GOS 1GOT 1GOU 1GOV 1GOW 1GOX 1GOY 1GOZ 1GP0 1GP1 1GP2 1GP3 1GP4 1GP5 1GP6 1GP7 1GP9 1GPA 1GPB 1GPC 1GPD 1GPE 1GPF 1GPH 1GPI 1GPJ 1GPK 1GPL 1GPM 1GPN 1GPO 1GPP 1GPQ 1GPR 1GPU 1GPW 1GPY 1GPZ 1GQ1 1GQ2 1GQ3 1GQ4 1GQ5 1GQ6 1GQ7 1GQ8 1GQ9 1GQA 1GQB 1GQC 1GQE 1GQF 1GQG 1GQH 1GQI 1GQJ 1GQK 1GQL 1GQM 1GQN 1GQO 1GQP 1GQQ 1GQR 1GQS 1GQT 1GQV 1GQW 1GQY 1GQZ 1GR0 1GR1 1GR2 1GR3 1GR7 1GRA 1GRB 1GRC 1GRE 1GRF 1GRG 1GRH 1GRI 1GRJ 1GRL 1GRN 1GRO 1GRP 1GRQ 1GRR 1GRT 1GRV 1GRW 1GS0 1GS3 1GS4 1GS5 1GS6 1GS7 1GS8 1GS9 1GSA 1GSB 1GSC 1GSD 1GSE 1GSF 1GSH 1GSI 1GSJ 1GSK 1GSL 1GSM 1GSN 1GSO 1GSP 1GSQ 1GSS 1GSU 1GSV 1GSW 1GSX 1GSY 1GSZ 1GT1 1GT3 1GT4 1GT5 1GT6 1GT7 1GT8 1GT9 1GTA 1GTB 1GTD 1GTE 1GTG 1GTH 1GTI 1GTJ 1GTK 1GTL 1GTM 1GTO 1GTP 1GTQ 1GTT 1GTU 1GTV 1GTZ 1GU0 1GU1 1GU2 1GU3 1GU6 1GU7 1GU8 1GU9 1GUA 1GUB 1GUD 1GUE 1GUF 1GUG 1GUH 1GUI 1GUJ 1GUK 1GUL 1GUM 1GUN 1GUO 1GUP 1GUQ 1GUS 1GUT 1GUU 1GUV 1GUX 1GUY 1GUZ 1GV0 1GV1 1GV2 1GV3 1GV4 1GV5 1GV7 1GV8 1GV9 1GVC 1GVD 1GVE 1GVF 1GVG 1GVH 1GVI 1GVJ 1GVK 1GVL 1GVM 1GVN 1GVO 1GVP 1GVQ 1GVR 1GVS 1GVT 1GVU 1GVV 1GVW 1GVX 1GVY 1GVZ 1GW0 1GW1 1GW2 1GW6 1GW9 1GWA 1GWB 1GWC 1GWD 1GWE 1GWF 1GWG 1GWH 1GWI 1GWJ 1GWK 1GWL 1GWM 1GWN 1GWO 1GWQ 1GWR 1GWS 1GWT 1GWU 1GWV 1GWW 1GWX 1GWY 1GWZ 1GX0 1GX1 1GX2 1GX3 1GX4 1GX5 1GX6 1GX8 1GX9 1GXA 1GXB 1GXC 1GXD 1GXF 1GXJ 1GXK 1GXL 1GXM 1GXN 1GXO 1GXQ 1GXR 1GXS 1GXT 1GXU 1GXW 1GXY 1GXZ 1GY0 1GY1 1GY2 1GY3 1GY5 1GY6 1GY7 1GY8 1GY9 1GYB 1GYC 1GYD 1GYE 1GYG 1GYH 1GYJ 1GYK 1GYL 1GYM 1GYN 1GYO 1GYP 1GYQ 1GYR 1GYT 1GYU 1GYV 1GYW 1GYX 1GYY 1GZ0 1GZ1 1GZ2 1GZ3 1GZ4 1GZ5 1GZ6 1GZ7 1GZ8 1GZ9 1GZA 1GZB 1GZC 1GZD 1GZE 1GZF 1GZG 1GZH 1GZI 1GZJ 1GZK 1GZL 1GZM 1GZN 1GZO 1GZP 1GZQ 1GZR 1GZS 1GZT 1GZU 1GZV 1GZW 1GZX 1GZY 1GZZ 1H00 1H01 1H02 1H03 1H04 1H05 1H07 1H08 1H09 1H0A 1H0B 1H0C 1H0D 1H0G 1H0H 1H0I 1H0J 1H0K 1H0N 1H0O 1H0P 1H0R 1H0S 1H0V 1H0W 1H0X 1H0Y 1H10 1H11 1H12 1H13 1H14 1H15 1H16 1H17 1H18 1H19 1H1A 1H1B 1H1C 1H1D 1H1H 1H1I 1H1L 1H1M 1H1N 1H1O 1H1P 1H1Q 1H1R 1H1S 1H1T 1H1V 1H1W 1H1X 1H1Y 1H1Z 1H21 1H22 1H23 1H24 1H25 1H26 1H27 1H28 1H29 1H2A 1H2B 1H2E 1H2F 1H2G 1H2H 1H2I 1H2J 1H2K 1H2L 1H2M 1H2N 1H2P 1H2Q 1H2R 1H2S 1H2T 1H2U 1H2V 1H2W 1H2X 1H2Y 1H2Z 1H30 1H31 1H32 1H33 1H34 1H35 1H36 1H37 1H39 1H3A 1H3B 1H3C 1H3D 1H3F 1H3G 1H3I 1H3J 1H3L 1H3M 1H3N 1H3O 1H3P 1H3Q 1H3T 1H3U 1H3V 1H3W 1H3X 1H3Y 1H41 1H42 1H43 1H44 1H45 1H46 1H47 1H48 1H49 1H4A 1H4C 1H4D 1H4E 1H4F 1H4G 1H4H 1H4I 1H4J 1H4K 1H4L 1H4M 1H4N 1H4O 1H4P 1H4R 1H4T 1H4U 1H4V 1H4W 1H4X 1H4Y 1H4Z 1H50 1H51 1H52 1H53 1H54 1H55 1H56 1H57 1H58 1H59 1H5A 1H5B 1H5C 1H5D 1H5E 1H5F 1H5G 1H5H 1H5I 1H5J 1H5K 1H5L 1H5M 1H5N 1H5Q 1H5R 1H5S 1H5T 1H5U 1H5V 1H5W 1H5X 1H5Y 1H5Z 1H60 1H61 1H62 1H63 1H64 1H65 1H66 1H68 1H69 1H6A 1H6B 1H6C 1H6D 1H6E 1H6G 1H6H 1H6J 1H6K 1H6L 1H6M 1H6N 1H6O 1H6P 1H6R 1H6S 1H6T 1H6U 1H6V 1H6W 1H6X 1H6Y 1H70 1H71 1H72 1H73 1H74 1H75 1H76 1H78 1H79 1H7A 1H7B 1H7C 1H7E 1H7F 1H7G 1H7H 1H7I 1H7K 1H7L 1H7M 1H7N 1H7O 1H7P 1H7Q 1H7R 1H7S 1H7T 1H7U 1H7W 1H7X 1H7Z 1H80 1H81 1H82 1H83 1H84 1H85 1H86 1H87 1H8D 1H8E 1H8F 1H8G 1H8H 1H8I 1H8K 1H8L 1H8N 1H8O 1H8P 1H8S 1H8T 1H8U 1H8V 1H8X 1H8Y 1H8Z 1H91 1H93 1H94 1H96 1H97 1H98 1H99 1H9A 1H9B 1H9G 1H9H 1H9I 1H9J 1H9K 1H9L 1H9M 1H9N 1H9O 1H9P 1H9Q 1H9R 1H9S 1H9U 1H9V 1H9W 1H9X 1H9Y 1H9Z 1HA0 1HA1 1HA2 1HA3 1HA4 1HA5 1HA7 1HAB 1HAC 1HAG 1HAH 1HAI 1HAK 1HAN 1HAR 1HAU 1HAV 1HAW 1HAX 1HAY 1HAZ 1HB0 1HB1 1HB2 1HB3 1HB4 1HB6 1HB8 1HBA 1HBB 1HBG 1HBH 1HBI 1HBJ 1HBK 1HBM 1HBN 1HBO 1HBP 1HBQ 1HBR 1HBS 1HBT 1HBU 1HBV 1HBY 1HBZ 1HC0 1HC1 1HC7 1HC9 1HCA 1HCB 1HCF 1HCG 1HCH 1HCI 1HCJ 1HCK 1HCL 1HCM 1HCN 1HCO 1HCU 1HCV 1HCX 1HCY 1HCZ 1HD2 1HD3 1HD5 1HD7 1HD8 1HDA 1HDB 1HDC 1HDE 1HDF 1HDG 1HDH 1HDI 1HDK 1HDM 1HDO 1HDQ 1HDR 1HDS 1HDT 1HDU 1HDX 1HDY 1HDZ 1HE1 1HE2 1HE3 1HE4 1HE5 1HE7 1HE8 1HE9 1HEA 1HEB 1HEC 1HED 1HEE 1HEF 1HEG 1HEI 1HEK 1HEL 1HEM 1HEN 1HEO 1HEP 1HEQ 1HER 1HES 1HET 1HEU 1HEW 1HEX 1HEY 1HEZ 1HF2 1HF3 1HF4 1HF6 1HF8 1HFA 1HFB 1HFC 1HFD 1HFE 1HFJ 1HFK 1HFO 1HFP 1HFQ 1HFR 1HFS 1HFU 1HFW 1HFX 1HFY 1HFZ 1HG0 1HG1 1HG2 1HG3 1HG4 1HG5 1HG7 1HG8 1HGA 1HGB 1HGC 1HGD 1HGE 1HGF 1HGG 1HGH 1HGI 1HGJ 1HGT 1HGU 1HGW 1HGX 1HGY 1HH1 1HH2 1HH3 1HH4 1HH5 1HH6 1HH7 1HH8 1HH9 1HHA 1HHC 1HHF 1HHG 1HHH 1HHI 1HHJ 1HHK 1HHL 1HHO 1HHP 1HHQ 1HHS 1HHU 1HHY 1HHZ 1HI1 1HI2 1HI3 1HI4 1HI5 1HI6 1HI8 1HI9 1HIA 1HIB 1HIG 1HIH 1HII 1HIJ 1HIK 1HIL 1HIM 1HIN 1HIO 1HIP 1HIV 1HIW 1HIX 1HIY 1HIZ 1HJ1 1HJ3 1HJ4 1HJ5 1HJ6 1HJ8 1HJ9 1HJA 1HJE 1HJF 1HJG 1HJJ 1HJK 1HJL 1HJO 1HJP 1HJQ 1HJR 1HJS 1HJT 1HJU 1HJV 1HJW 1HJX 1HJZ 1HK0 1HK1 1HK2 1HK3 1HK4 1HK5 1HK7 1HK8 1HK9 1HKA 1HKB 1HKC 1HKD 1HKF 1HKG 1HKH 1HKI 1HKJ 1HKK 1HKL 1HKM 1HKN 1HKQ 1HKU 1HKV 1HKW 1HKX 1HL2 1HL3 1HL4 1HL5 1HL6 1HL7 1HL8 1HL9 1HLA 1HLB 1HLC 1HLD 1HLE 1HLF 1HLG 1HLK 1HLM 1HLP 1HLQ 1HLT 1HLU 1HLW 1HM0 1HM2 1HM3 1HM4 1HM5 1HM6 1HM7 1HM8 1HM9 1HMC 1HMD 1HMK 1HML 1HMO 1HMP 1HMR 1HMS 1HMT 1HMU 1HMV 1HMW 1HMY 1HN0 1HN1 1HN2 1HN4 1HN9 1HNA 1HNB 1HNC 1HND 1HNE 1HNF 1HNG 1HNH 1HNI 1HNJ 1HNK 1HNL 1HNN 1HNO 1HNU 1HNV 1HNY 1HO1 1HO3 1HO4 1HO5 1HO8 1HOC 1HOE 1HON 1HOO 1HOP 1HOR 1HOS 1HOT 1HOW 1HOX 1HOZ 1HP0 1HP1 1HP4 1HP5 1HP7 1HPB 1HPC 1HPG 1HPI 1HPL 1HPM 1HPO 1HPS 1HPT 1HPU 1HPV 1HPX 1HPZ 1HQ0 1HQ2 1HQ3 1HQ4 1HQ5 1HQ6 1HQ8 1HQA 1HQC 1HQD 1HQE 1HQF 1HQG 1HQH 1HQJ 1HQK 1HQL 1HQM 1HQN 1HQO 1HQP 1HQQ 1HQR 1HQS 1HQT 1HQU 1HQV 1HQW 1HQX 1HQY 1HQZ 1HR3 1HR6 1HR7 1HR8 1HR9 1HRB 1HRC 1HRD 1HRH 1HRI 1HRK 1HRM 1HRN 1HRO 1HRP 1HRS 1HRT 1HRU 1HRV 1HS6 1HSA 1HSB 1HSE 1HSG 1HSH 1HSI 1HSJ 1HSK 1HSL 1HSO 1HSR 1HSS 1HST 1HSW 1HSX 1HSY 1HSZ 1HT0 1HT1 1HT2 1HT3 1HT5 1HT6 1HT8 1HT9 1HTA 1HTB 1HTD 1HTE 1HTF 1HTG 1HTI 1HTJ 1HTL 1HTM 1HTN 1HTO 1HTP 1HTQ 1HTR 1HTT 1HTV 1HTW 1HTY 1HTZ 1HU3 1HU8 1HU9 1HUC 1HUF 1HUG 1HUH 1HUJ 1HUK 1HUL 1HUP 1HUQ 1HUR 1HUS 1HUU 1HUV 1HUW 1HUX 1HUY 1HV0 1HV1 1HV4 1HV5 1HV6 1HV7 1HV8 1HV9 1HVA 1HVB 1HVC 1HVD 1HVE 1HVF 1HVG 1HVH 1HVI 1HVJ 1HVK 1HVL 1HVQ 1HVR 1HVS 1HVV 1HVX 1HVY 1HW1 1HW3 1HW4 1HW5 1HW6 1HW7 1HW8 1HW9 1HWG 1HWH 1HWI 1HWJ 1HWK 1HWL 1HWM 1HWN 1HWO 1HWP 1HWR 1HWU 1HWW 1HWY 1HWZ 1HX0 1HX1 1HX3 1HX5 1HX6 1HX8 1HX9 1HXA 1HXB 1HXC 1HXD 1HXE 1HXF 1HXG 1HXH 1HXI 1HXJ 1HXK 1HXL 1HXM 1HXN 1HXP 1HXQ 1HXR 1HXS 1HXT 1HXU 1HXW 1HXX 1HXY 1HXZ 1HY0 1HY1 1HY2 1HY3 1HY5 1HY7 1HYB 1HYE 1HYF 1HYG 1HYH 1HYL 1HYN 1HYO 1HYP 1HYQ 1HYR 1HYT 1HYU 1HYV 1HYZ 1HZ1 1HZ4 1HZ5 1HZ6 1HZ9 1HZA 1HZB 1HZC 1HZD 1HZF 1HZG 1HZH 1HZI 1HZJ 1HZO 1HZP 1HZT 1HZU 1HZV 1HZW 1HZX 1HZY 1HZZ 1I00 1I01 1I04 1I05 1I06 1I07 1I08 1I09 1I0A 1I0B 1I0C 1I0D 1I0E 1I0H 1I0I 1I0L 1I0R 1I0S 1I0V 1I0X 1I0Z 1I10 1I12 1I13 1I14 1I19 1I1A 1I1B 1I1C 1I1D 1I1E 1I1F 1I1G 1I1H 1I1I 1I1J 1I1K 1I1L 1I1M 1I1N 1I1O 1I1Q 1I1R 1I1W 1I1X 1I1Y 1I1Z 1I20 1I21 1I22 1I24 1I27 1I29 1I2A 1I2B 1I2C 1I2D 1I2E 1I2F 1I2G 1I2H 1I2K 1I2L 1I2M 1I2N 1I2O 1I2P 1I2Q 1I2R 1I2S 1I2T 1I2W 1I2Z 1I30 1I31 1I32 1I33 1I36 1I37 1I38 1I39 1I3A 1I3C 1I3D 1I3E 1I3F 1I3G 1I3H 1I3I 1I3K 1I3L 1I3M 1I3N 1I3O 1I3P 1I3Q 1I3R 1I3S 1I3U 1I3V 1I3Z 1I40 1I41 1I43 1I44 1I45 1I48 1I49 1I4A 1I4D 1I4E 1I4F 1I4G 1I4H 1I4J 1I4K 1I4L 1I4M 1I4N 1I4O 1I4P 1I4Q 1I4R 1I4S 1I4T 1I4U 1I4W 1I4X 1I4Y 1I4Z 1I50 1I51 1I52 1I53 1I54 1I55 1I57 1I58 1I59 1I5A 1I5B 1I5C 1I5D 1I5E 1I5F 1I5G 1I5I 1I5K 1I5N 1I5O 1I5P 1I5Q 1I5R 1I5S 1I5X 1I5Y 1I5Z 1I60 1I69 1I6A 1I6B 1I6I 1I6K 1I6L 1I6M 1I6N 1I6O 1I6P 1I6Q 1I6S 1I6T 1I6V 1I6W 1I6X 1I70 1I71 1I72 1I73 1I74 1I75 1I76 1I77 1I78 1I79 1I7A 1I7B 1I7C 1I7E 1I7F 1I7G 1I7H 1I7I 1I7K 1I7L 1I7M 1I7N 1I7O 1I7P 1I7Q 1I7R 1I7S 1I7T 1I7U 1I7W 1I7X 1I7Y 1I7Z 1I80 1I81 1I82 1I83 1I85 1I86 1I88 1I89 1I8A 1I8B 1I8D 1I8F 1I8I 1I8J 1I8K 1I8L 1I8N 1I8O 1I8P 1I8Q 1I8T 1I8U 1I8V 1I8Z 1I90 1I91 1I92 1I9A 1I9B 1I9C 1I9D 1I9E 1I9G 1I9H 1I9I 1I9J 1I9L 1I9M 1I9N 1I9O 1I9P 1I9Q 1I9R 1I9S 1I9T 1I9W 1I9Y 1I9Z 1IA1 1IA2 1IA3 1IA4 1IA5 1IA6 1IA7 1IA8 1IA9 1IAA 1IAB 1IAC 1IAD 1IAE 1IAG 1IAH 1IAI 1IAJ 1IAK 1IAL 1IAM 1IAN 1IAO 1IAP 1IAQ 1IAR 1IAS 1IAT 1IAU 1IAV 1IAX 1IAY 1IAZ 1IB0 1IB1 1IB2 1IB4 1IB5 1IB6 1IBB 1IBC 1IBD 1IBE 1IBF 1IBG 1IBH 1IBJ 1IBQ 1IBR 1IBS 1IBT 1IBU 1IBV 1IBW 1IBY 1IBZ 1IC0 1IC1 1IC2 1IC4 1IC5 1IC6 1IC7 1ICC 1ICE 1ICF 1ICI 1ICJ 1ICM 1ICN 1ICP 1ICQ 1ICR 1ICS 1ICT 1ICU 1ICV 1ICW 1ICX 1ID0 1ID1 1ID2 1ID4 1ID5 1IDA 1IDB 1IDC 1IDD 1IDE 1IDF 1IDJ 1IDK 1IDM 1IDN 1IDO 1IDP 1IDQ 1IDR 1IDS 1IDT 1IDU 1IE0 1IE3 1IE4 1IE7 1IE8 1IE9 1IEA 1IEB 1IEC 1IED 1IEE 1IEF 1IEG 1IEI 1IEJ 1IEL 1IEM 1IEP 1IEQ 1IER 1IES 1IEV 1IEW 1IEX 1IF2 1IF4 1IF5 1IF6 1IF7 1IF8 1IF9 1IFA 1IFB 1IFC 1IFG 1IFH 1IFQ 1IFR 1IFS 1IFT 1IFU 1IFV 1IFX 1IG0 1IG1 1IG3 1IG5 1IG8 1IGB 1IGC 1IGD 1IGF 1IGI 1IGJ 1IGM 1IGO 1IGP 1IGQ 1IGR 1IGS 1IGT 1IGU 1IGV 1IGW 1IGX 1IGY 1IGZ 1IH7 1IH8 1IHB 1IHC 1IHD 1IHG 1IHI 1IHJ 1IHK 1IHM 1IHN 1IHO 1IHP 1IHR 1IHS 1IHT 1IHU 1IHX 1IHY 1IHZ 1II0 1II2 1II3 1II4 1II5 1II6 1II7 1II8 1II9 1IIB 1IIC 1IID 1IIG 1IIH 1III 1IIK 1IIL 1IIM 1IIN 1IIP 1IIQ 1IIR 1IIT 1IIU 1IIW 1IIZ 1IJ0 1IJ1 1IJ2 1IJ3 1IJ5 1IJ6 1IJ8 1IJ9 1IJB 1IJD 1IJE 1IJF 1IJG 1IJH 1IJI 1IJJ 1IJK 1IJL 1IJN 1IJQ 1IJR 1IJT 1IJU 1IJV 1IJX 1IJY 1IK3 1IK4 1IK6 1IK7 1IK9 1IKA 1IKE 1IKF 1IKG 1IKI 1IKJ 1IKN 1IKO 1IKP 1IKQ 1IKT 1IKV 1IKW 1IKX 1IKY 1IL0 1IL1 1IL3 1IL4 1IL5 1IL9 1ILD 1ILE 1ILG 1ILH 1ILK 1ILR 1ILS 1ILT 1ILU 1ILV 1ILW 1ILX 1ILZ 1IM0 1IM2 1IM3 1IM4 1IM5 1IM6 1IM8 1IM9 1IMA 1IMB 1IMC 1IMD 1IME 1IMF 1IMJ 1IMV 1IMX 1IN0 1IN4 1IN5 1IN6 1IN7 1IN8 1INC 1IND 1INE 1INF 1ING 1INH 1INI 1INJ 1INL 1INN 1INO 1INP 1INQ 1INR 1INU 1INV 1INW 1INX 1INY 1IO0 1IO1 1IO2 1IO3 1IO7 1IO8 1IO9 1IOA 1IOC 1IOD 1IOE 1IOF 1IOI 1IOK 1IOL 1IOM 1ION 1IOO 1IOP 1IOQ 1IOR 1IOS 1IOT 1IOV 1IOW 1IOZ 1IP1 1IP2 1IP3 1IP4 1IP5 1IP6 1IP7 1IPA 1IPB 1IPC 1IPD 1IPE 1IPF 1IPH 1IPI 1IPJ 1IPK 1IPS 1IPW 1IQ0 1IQ1 1IQ4 1IQ5 1IQ6 1IQ7 1IQ8 1IQ9 1IQA 1IQB 1IQC 1IQD 1IQE 1IQF 1IQG 1IQH 1IQI 1IQJ 1IQK 1IQL 1IQM 1IQN 1IQP 1IQQ 1IQR 1IQU 1IQV 1IQW 1IQX 1IQY 1IQZ 1IR0 1IR1 1IR2 1IR3 1IR6 1IR7 1IR8 1IR9 1IRA 1IRB 1IRC 1IRD 1IRE 1IRI 1IRJ 1IRK 1IRM 1IRN 1IRO 1IRQ 1IRU 1IRV 1IRW 1IRX 1IS0 1IS1 1IS2 1IS3 1IS4 1IS5 1IS6 1IS7 1IS8 1IS9 1ISA 1ISB 1ISC 1ISE 1ISF 1ISG 1ISH 1ISI 1ISJ 1ISM 1ISN 1ISO 1ISP 1ISQ 1ISR 1ISS 1IST 1ISU 1ISV 1ISW 1ISX 1ISY 1ISZ 1IT0 1IT2 1IT3 1IT6 1IT7 1IT8 1IT9 1ITB 1ITC 1ITG 1ITH 1ITK 1ITO 1ITQ 1ITT 1ITU 1ITV 1ITW 1ITX 1ITZ 1IU1 1IU4 1IU5 1IU7 1IU8 1IU9 1IUA 1IUB 1IUC 1IUD 1IUE 1IUG 1IUH 1IUJ 1IUK 1IUL 1IUN 1IUO 1IUP 1IUQ 1IUS 1IUT 1IUU 1IUV 1IUW 1IUX 1IUZ 1IV1 1IV2 1IV3 1IV4 1IV5 1IV7 1IV8 1IV9 1IVB 1IVC 1IVD 1IVE 1IVF 1IVG 1IVH 1IVI 1IVJ 1IVL 1IVN 1IVO 1IVP 1IVQ 1IVR 1IVU 1IVV 1IVW 1IVX 1IVY 1IW0 1IW1 1IW2 1IW6 1IW7 1IW8 1IW9 1IWA 1IWB 1IWD 1IWE 1IWG 1IWH 1IWI 1IWJ 1IWK 1IWL 1IWM 1IWN 1IWO 1IWP 1IWQ 1IWT 1IWU 1IWV 1IWW 1IWX 1IWY 1IWZ 1IX0 1IX1 1IX2 1IX3 1IX4 1IX6 1IX7 1IX8 1IX9 1IXB 1IXC 1IXE 1IXF 1IXG 1IXH 1IXI 1IXK 1IXL 1IXM 1IXN 1IXO 1IXP 1IXQ 1IXR 1IXS 1IXV 1IXX 1IXZ 1IY0 1IY1 1IY2 1IY7 1IY8 1IY9 1IYB 1IYD 1IYE 1IYH 1IYI 1IYJ 1IYK 1IYL 1IYN 1IYO 1IYP 1IYQ 1IYS 1IYW 1IYX 1IYZ 1IZ0 1IZ1 1IZ2 1IZ3 1IZ4 1IZ5 1IZ6 1IZ7 1IZ8 1IZ9 1IZA 1IZB 1IZC 1IZD 1IZE 1IZH 1IZI 1IZJ 1IZK 1IZL 1IZM 1IZN 1IZO 1IZP 1IZQ 1IZR 1IZY 1IZZ 1J00 1J01 1J02 1J04 1J05 1J06 1J07 1J08 1J09 1J0A 1J0B 1J0C 1J0D 1J0E 1J0H 1J0I 1J0J 1J0K 1J0M 1J0N 1J0O 1J0P 1J0R 1J0W 1J0X 1J0Y 1J0Z 1J10 1J11 1J12 1J14 1J15 1J16 1J17 1J18 1J19 1J1A 1J1B 1J1C 1J1D 1J1E 1J1F 1J1G 1J1I 1J1J 1J1L 1J1M 1J1N 1J1O 1J1P 1J1Q 1J1R 1J1S 1J1T 1J1W 1J1X 1J1Y 1J1Z 1J20 1J21 1J22 1J23 1J24 1J25 1J27 1J2A 1J2C 1J2E 1J2F 1J2G 1J2J 1J2L 1J2P 1J2Q 1J2R 1J2T 1J2U 1J2V 1J2W 1J2X 1J2Y 1J2Z 1J30 1J31 1J32 1J33 1J34 1J35 1J36 1J37 1J38 1J39 1J3A 1J3B 1J3F 1J3H 1J3I 1J3J 1J3K 1J3L 1J3M 1J3N 1J3P 1J3Q 1J3R 1J3U 1J3W 1J3Y 1J3Z 1J40 1J41 1J42 1J48 1J49 1J4A 1J4B 1J4E 1J4G 1J4H 1J4I 1J4J 1J4N 1J4R 1J4S 1J4T 1J4U 1J4X 1J51 1J52 1J53 1J54 1J55 1J58 1J5P 1J5Q 1J5S 1J5T 1J5U 1J5W 1J5X 1J5Y 1J6O 1J6P 1J6R 1J6U 1J6V 1J6W 1J6X 1J6Z 1J70 1J71 1J72 1J73 1J74 1J77 1J78 1J79 1J7A 1J7B 1J7C 1J7D 1J7E 1J7G 1J7I 1J7J 1J7K 1J7L 1J7N 1J7S 1J7U 1J7V 1J7W 1J7X 1J7Y 1J7Z 1J80 1J81 1J82 1J83 1J84 1J85 1J86 1J87 1J88 1J89 1J8A 1J8B 1J8D 1J8E 1J8F 1J8H 1J8M 1J8Q 1J8R 1J8S 1J8T 1J8U 1J8V 1J8Y 1J90 1J91 1J93 1J95 1J96 1J97 1J98 1J99 1J9A 1J9B 1J9C 1J9E 1J9G 1J9J 1J9K 1J9L 1J9M 1J9Q 1J9R 1J9S 1J9T 1J9W 1J9Y 1J9Z 1JA0 1JA1 1JA3 1JA8 1JA9 1JAB 1JAC 1JAD 1JAE 1JAF 1JAH 1JAI 1JAK 1JAL 1JAM 1JAN 1JAO 1JAP 1JAQ 1JAT 1JAW 1JAX 1JAY 1JAZ 1JB0 1JB1 1JB2 1JB3 1JB4 1JB5 1JB6 1JB9 1JBB 1JBC 1JBE 1JBG 1JBK 1JBM 1JBO 1JBP 1JBQ 1JBU 1JBV 1JBW 1JBY 1JBZ 1JC0 1JC1 1JC4 1JC5 1JC7 1JC9 1JCA 1JCC 1JCD 1JCE 1JCF 1JCG 1JCH 1JCI 1JCJ 1JCK 1JCL 1JCM 1JCN 1JCQ 1JCR 1JCS 1JCT 1JCV 1JCX 1JCY 1JCZ 1JD0 1JD1 1JD2 1JD3 1JD4 1JD5 1JD6 1JD7 1JD9 1JDA 1JDB 1JDC 1JDD 1JDE 1JDF 1JDH 1JDI 1JDJ 1JDL 1JDN 1JDO 1JDP 1JDR 1JDS 1JDT 1JDU 1JDV 1JDW 1JDX 1JDY 1JDZ 1JE0 1JE1 1JE5 1JE6 1JEA 1JEB 1JEC 1JED 1JEE 1JEF 1JEH 1JEJ 1JEK 1JEN 1JEO 1JEP 1JEQ 1JER 1JET 1JEU 1JEV 1JEZ 1JF0 1JF1 1JF2 1JF3 1JF4 1JF5 1JF6 1JF7 1JF8 1JF9 1JFA 1JFB 1JFC 1JFD 1JFG 1JFH 1JFL 1JFM 1JFQ 1JFR 1JFU 1JFV 1JFX 1JFZ 1JG0 1JG1 1JG2 1JG3 1JG4 1JG5 1JG6 1JG7 1JG8 1JG9 1JGC 1JGD 1JGE 1JGI 1JGJ 1JGL 1JGM 1JGS 1JGT 1JGU 1JGV 1JGW 1JGX 1JGY 1JGZ 1JH0 1JH1 1JH5 1JH6 1JH7 1JH8 1JHA 1JHC 1JHD 1JHE 1JHF 1JHG 1JHH 1JHJ 1JHK 1JHL 1JHM 1JHN 1JHO 1JHP 1JHQ 1JHR 1JHS 1JHT 1JHU 1JHV 1JHW 1JHX 1JHY 1JHZ 1JI0 1JI1 1JI2 1JI3 1JI4 1JI5 1JI6 1JI7 1JIA 1JIB 1JIE 1JIF 1JIG 1JIH 1JII 1JIJ 1JIK 1JIL 1JIM 1JIN 1JIO 1JIP 1JIQ 1JIR 1JIS 1JIT 1JIU 1JIV 1JIW 1JIX 1JIY 1JIZ 1JJ0 1JJ1 1JJ3 1JJ7 1JJ9 1JJA 1JJB 1JJC 1JJE 1JJF 1JJH 1JJI 1JJK 1JJO 1JJT 1JJU 1JJV 1JJW 1JK0 1JK3 1JK4 1JK6 1JK7 1JK8 1JK9 1JKA 1JKB 1JKC 1JKD 1JKE 1JKF 1JKG 1JKH 1JKI 1JKJ 1JKK 1JKL 1JKM 1JKS 1JKT 1JKU 1JKV 1JKW 1JKX 1JKY 1JL0 1JL1 1JL2 1JL3 1JL4 1JL5 1JL6 1JL7 1JL8 1JL9 1JLA 1JLB 1JLC 1JLD 1JLE 1JLF 1JLG 1JLH 1JLJ 1JLK 1JLL 1JLM 1JLN 1JLQ 1JLR 1JLS 1JLT 1JLU 1JLV 1JLW 1JLX 1JLY 1JM0 1JM1 1JM6 1JMA 1JMB 1JME 1JMF 1JMG 1JMH 1JMI 1JMJ 1JMK 1JML 1JMM 1JMO 1JMS 1JMT 1JMU 1JMV 1JMW 1JMX 1JMY 1JMZ 1JN0 1JN1 1JN2 1JN3 1JN4 1JN5 1JN6 1JN9 1JNB 1JND 1JNE 1JNF 1JNH 1JNI 1JNK 1JNL 1JNN 1JNP 1JNQ 1JNR 1JNU 1JNV 1JNW 1JNX 1JNY 1JNZ 1JO0 1JO8 1JOA 1JOB 1JOC 1JOD 1JOE 1JOF 1JOG 1JOH 1JOI 1JOJ 1JOL 1JOM 1JON 1JOP 1JOS 1JOT 1JOU 1JOV 1JOW 1JP3 1JP4 1JP5 1JP6 1JP7 1JP8 1JP9 1JPA 1JPB 1JPC 1JPD 1JPE 1JPF 1JPG 1JPH 1JPI 1JPJ 1JPK 1JPL 1JPM 1JPN 1JPO 1JPP 1JPR 1JPS 1JPT 1JPU 1JPV 1JPW 1JPX 1JPY 1JPZ 1JQ0 1JQ3 1JQ5 1JQ6 1JQ7 1JQ8 1JQ9 1JQA 1JQB 1JQC 1JQD 1JQE 1JQF 1JQG 1JQH 1JQI 1JQJ 1JQK 1JQL 1JQN 1JQO 1JQP 1JQQ 1JQU 1JQV 1JQW 1JQX 1JQY 1JQZ 1JR0 1JR1 1JR2 1JR3 1JR4 1JR7 1JR8 1JR9 1JRA 1JRB 1JRC 1JRE 1JRG 1JRH 1JRI 1JRK 1JRL 1JRO 1JRP 1JRQ 1JRR 1JRS 1JRT 1JRX 1JRY 1JRZ 1JS0 1JS1 1JS2 1JS3 1JS4 1JS6 1JS8 1JS9 1JSC 1JSD 1JSE 1JSF 1JSG 1JSH 1JSI 1JSL 1JSM 1JSN 1JSO 1JSR 1JSS 1JST 1JSU 1JSV 1JSW 1JSX 1JSY 1JSZ 1JT1 1JT2 1JT3 1JT4 1JT5 1JT6 1JT7 1JT9 1JTA 1JTC 1JTD 1JTE 1JTF 1JTG 1JTH 1JTI 1JTK 1JTM 1JTN 1JTO 1JTP 1JTQ 1JTS 1JTT 1JTU 1JTV 1JTX 1JTY 1JTZ 1JU2 1JU3 1JU4 1JU6 1JU9 1JUB 1JUD 1JUE 1JUF 1JUG 1JUH 1JUI 1JUJ 1JUK 1JUL 1JUM 1JUO 1JUP 1JUQ 1JUS 1JUT 1JUV 1JUY 1JV0 1JV1 1JV2 1JV3 1JV4 1JV5 1JV6 1JV7 1JVA 1JVB 1JVD 1JVG 1JVI 1JVJ 1JVK 1JVL 1JVM 1JVN 1JVO 1JVP 1JVQ 1JVS 1JVT 1JVU 1JVV 1JVW 1JVX 1JVY 1JVZ 1JW0 1JW1 1JW4 1JW5 1JW6 1JW8 1JW9 1JWA 1JWB 1JWF 1JWG 1JWH 1JWI 1JWJ 1JWK 1JWM 1JWN 1JWO 1JWP 1JWQ 1JWR 1JWS 1JWT 1JWU 1JWV 1JWX 1JWY 1JWZ 1JX0 1JX1 1JX2 1JX6 1JX7 1JX9 1JXA 1JXB 1JXE 1JXG 1JXH 1JXI 1JXJ 1JXK 1JXM 1JXN 1JXO 1JXP 1JXQ 1JXT 1JXU 1JXV 1JXW 1JXX 1JXY 1JXZ 1JY0 1JY1 1JY2 1JY3 1JY5 1JY7 1JY8 1JYA 1JYB 1JYC 1JYD 1JYE 1JYF 1JYH 1JYI 1JYJ 1JYK 1JYL 1JYM 1JYN 1JYO 1JYQ 1JYR 1JYS 1JYU 1JYV 1JYW 1JYX 1JZ2 1JZ3 1JZ4 1JZ5 1JZ6 1JZ7 1JZ8 1JZA 1JZB 1JZD 1JZE 1JZF 1JZG 1JZH 1JZI 1JZJ 1JZK 1JZL 1JZM 1JZN 1JZO 1JZQ 1JZR 1JZS 1JZT 1JZW 1K02 1K03 1K04 1K05 1K06 1K07 1K08 1K0A 1K0B 1K0C 1K0D 1K0E 1K0F 1K0G 1K0I 1K0J 1K0K 1K0L 1K0M 1K0N 1K0O 1K0R 1K0U 1K0W 1K0Y 1K0Z 1K12 1K1A 1K1B 1K1D 1K1E 1K1F 1K1I 1K1J 1K1K 1K1L 1K1M 1K1N 1K1O 1K1P 1K1Q 1K1S 1K1T 1K1U 1K1W 1K1X 1K1Y 1K20 1K21 1K22 1K23 1K24 1K25 1K26 1K27 1K28 1K2A 1K2B 1K2C 1K2D 1K2E 1K2F 1K2I 1K2O 1K2P 1K2R 1K2S 1K2T 1K2U 1K2V 1K2W 1K2X 1K2Y 1K30 1K32 1K33 1K34 1K35 1K38 1K39 1K3A 1K3B 1K3C 1K3D 1K3E 1K3F 1K3I 1K3L 1K3O 1K3R 1K3S 1K3T 1K3U 1K3V 1K3Y 1K3Z 1K40 1K41 1K44 1K46 1K47 1K49 1K4C 1K4D 1K4E 1K4F 1K4G 1K4H 1K4I 1K4J 1K4K 1K4L 1K4M 1K4N 1K4O 1K4P 1K4Q 1K4V 1K4W 1K4Y 1K4Z 1K50 1K51 1K52 1K53 1K54 1K55 1K56 1K57 1K58 1K59 1K5A 1K5B 1K5C 1K5D 1K5G 1K5H 1K5J 1K5M 1K5N 1K5P 1K5Q 1K5S 1K5U 1K5V 1K62 1K63 1K66 1K68 1K6A 1K6C 1K6D 1K6E 1K6F 1K6I 1K6J 1K6K 1K6L 1K6M 1K6N 1K6P 1K6Q 1K6R 1K6S 1K6T 1K6U 1K6V 1K6W 1K6X 1K6Y 1K6Z 1K70 1K72 1K74 1K75 1K77 1K7C 1K7D 1K7E 1K7F 1K7G 1K7H 1K7I 1K7J 1K7K 1K7L 1K7Q 1K7S 1K7T 1K7U 1K7V 1K7W 1K7X 1K7Y 1K83 1K86 1K88 1K89 1K8C 1K8D 1K8F 1K8I 1K8K 1K8Q 1K8R 1K8T 1K8U 1K8X 1K8Y 1K8Z 1K90 1K92 1K93 1K94 1K95 1K96 1K97 1K98 1K9A 1K9B 1K9D 1K9E 1K9F 1K9I 1K9J 1K9K 1K9O 1K9P 1K9S 1K9T 1K9U 1K9V 1K9X 1K9Y 1K9Z 1KA0 1KA1 1KA2 1KA4 1KA8 1KA9 1KAA 1KAB 1KAC 1KAE 1KAF 1KAG 1KAH 1KAK 1KAM 1KAN 1KAO 1KAP 1KAQ 1KAR 1KAS 1KAV 1KAW 1KAX 1KAY 1KAZ 1KB0 1KB3 1KB5 1KB9 1KBA 1KBB 1KBC 1KBG 1KBI 1KBJ 1KBK 1KBL 1KBN 1KBO 1KBP 1KBQ 1KBR 1KBV 1KBW 1KBY 1KBZ 1KC1 1KC2 1KC3 1KC5 1KC7 1KCA 1KCB 1KCC 1KCD 1KCE 1KCF 1KCG 1KCK 1KCL 1KCM 1KCQ 1KCR 1KCS 1KCT 1KCU 1KCV 1KCW 1KCX 1KCZ 1KD0 1KD2 1KD7 1KD8 1KD9 1KDA 1KDB 1KDC 1KDD 1KDG 1KDI 1KDJ 1KDK 1KDM 1KDN 1KDO 1KDP 1KDQ 1KDR 1KDS 1KDT 1KDV 1KDW 1KDY 1KDZ 1KE0 1KE1 1KE2 1KE3 1KE4 1KE5 1KE6 1KE7 1KE8 1KE9 1KEA 1KEB 1KEC 1KEE 1KEH 1KEI 1KEJ 1KEK 1KEL 1KEM 1KEN 1KEO 1KEP 1KEQ 1KER 1KET 1KEU 1KEV 1KEW 1KEX 1KEY 1KEZ 1KF0 1KF2 1KF3 1KF4 1KF5 1KF6 1KF7 1KF8 1KF9 1KFA 1KFB 1KFC 1KFD 1KFE 1KFF 1KFG 1KFI 1KFJ 1KFK 1KFL 1KFM 1KFN 1KFQ 1KFR 1KFU 1KFW 1KFX 1KFY 1KG0 1KG2 1KG3 1KG4 1KG5 1KG6 1KG7 1KG8 1KG9 1KGA 1KGB 1KGC 1KGD 1KGE 1KGF 1KGG 1KGI 1KGJ 1KGN 1KGO 1KGP 1KGQ 1KGS 1KGT 1KGU 1KGW 1KGX 1KGY 1KGZ 1KH0 1KH1 1KH2 1KH3 1KH4 1KH5 1KH7 1KH8 1KH9 1KHB 1KHC 1KHD 1KHE 1KHF 1KHG 1KHH 1KHI 1KHJ 1KHK 1KHL 1KHN 1KHO 1KHP 1KHQ 1KHR 1KHT 1KHU 1KHV 1KHW 1KHX 1KHY 1KHZ 1KI0 1KI1 1KI2 1KI3 1KI4 1KI6 1KI7 1KI8 1KI9 1KIA 1KIB 1KIC 1KID 1KIE 1KIF 1KIG 1KIJ 1KIL 1KIM 1KIP 1KIQ 1KIR 1KIT 1KIU 1KIV 1KIY 1KIZ 1KJ1 1KJ2 1KJ3 1KJ4 1KJ7 1KJ8 1KJ9 1KJF 1KJG 1KJH 1KJI 1KJJ 1KJL 1KJM 1KJN 1KJO 1KJP 1KJQ 1KJR 1KJT 1KJV 1KJW 1KJX 1KJY 1KJZ 1KK0 1KK1 1KK2 1KK3 1KK4 1KK5 1KK6 1KK7 1KK8 1KK9 1KKB 1KKC 1KKE 1KKF 1KKH 1KKJ 1KKK 1KKL 1KKM 1KKO 1KKP 1KKQ 1KKR 1KKT 1KKU 1KL1 1KL2 1KL3 1KL4 1KL5 1KL6 1KL7 1KL9 1KLF 1KLG 1KLI 1KLJ 1KLK 1KLL 1KLM 1KLO 1KLT 1KLU 1KLX 1KLY 1KLZ 1KM0 1KM1 1KM2 1KM3 1KM4 1KM5 1KM6 1KM8 1KM9 1KMB 1KMC 1KME 1KMH 1KMI 1KMJ 1KMK 1KMM 1KMN 1KMO 1KMP 1KMQ 1KMS 1KMT 1KMV 1KMY 1KMZ 1KN0 1KN1 1KN2 1KN3 1KN4 1KN9 1KNA 1KNB 1KNC 1KND 1KNE 1KNF 1KNG 1KNI 1KNJ 1KNK 1KNL 1KNM 1KNO 1KNP 1KNQ 1KNR 1KNT 1KNU 1KNV 1KNW 1KNX 1KNY 1KO0 1KO1 1KO2 1KO3 1KO4 1KO5 1KO6 1KO7 1KO8 1KO9 1KOA 1KOB 1KOE 1KOF 1KOH 1KOI 1KOJ 1KOK 1KOL 1KON 1KOO 1KOP 1KOQ 1KOR 1KOU 1KP0 1KP2 1KP3 1KP4 1KP5 1KP6 1KP8 1KP9 1KPA 1KPB 1KPC 1KPE 1KPF 1KPG 1KPH 1KPI 1KPK 1KPL 1KPM 1KPR 1KPS 1KPT 1KPU 1KPV 1KQ0 1KQ1 1KQ3 1KQ4 1KQ5 1KQ6 1KQ7 1KQ9 1KQA 1KQB 1KQC 1KQD 1KQF 1KQG 1KQJ 1KQL 1KQM 1KQN 1KQO 1KQP 1KQR 1KQU 1KQW 1KQX 1KQY 1KQZ 1KR0 1KR1 1KR2 1KR3 1KR4 1KR5 1KR6 1KR7 1KRA 1KRB 1KRC 1KRE 1KRF 1KRH 1KRJ 1KRL 1KRM 1KRN 1KRO 1KRQ 1KRR 1KRU 1KRV 1KS2 1KS3 1KS4 1KS5 1KS7 1KS8 1KS9 1KSC 1KSD 1KSF 1KSG 1KSH 1KSI 1KSJ 1KSK 1KSL 1KSN 1KSO 1KSS 1KSU 1KSV 1KSW 1KSZ 1KT0 1KT1 1KT2 1KT3 1KT4 1KT5 1KT6 1KT7 1KT8 1KT9 1KTA 1KTB 1KTC 1KTD 1KTE 1KTG 1KTH 1KTI 1KTJ 1KTK 1KTL 1KTN 1KTO 1KTP 1KTQ 1KTR 1KTS 1KTT 1KTV 1KTW 1KTZ 1KU0 1KU1 1KU2 1KU3 1KU5 1KU6 1KU8 1KU9 1KUF 1KUG 1KUH 1KUI 1KUJ 1KUK 1KUT 1KUU 1KUV 1KUX 1KUY 1KV0 1KV1 1KV2 1KV3 1KV5 1KV6 1KV7 1KV8 1KV9 1KVA 1KVB 1KVC 1KVD 1KVE 1KVK 1KVL 1KVM 1KVO 1KVQ 1KVR 1KVS 1KVT 1KVU 1KVW 1KVX 1KVY 1KW0 1KW1 1KW2 1KW3 1KW4 1KW5 1KW6 1KW7 1KW8 1KW9 1KWA 1KWB 1KWC 1KWF 1KWG 1KWH 1KWI 1KWK 1KWM 1KWN 1KWO 1KWP 1KWQ 1KWR 1KWS 1KWT 1KWU 1KWV 1KWW 1KWX 1KWY 1KWZ 1KX0 1KX1 1KX8 1KX9 1KXA 1KXB 1KXC 1KXD 1KXE 1KXF 1KXG 1KXH 1KXI 1KXJ 1KXM 1KXN 1KXO 1KXP 1KXQ 1KXR 1KXT 1KXU 1KXV 1KXW 1KXX 1KXY 1KXZ 1KY0 1KY1 1KY2 1KY3 1KY4 1KY5 1KY6 1KY7 1KY8 1KY9 1KYA 1KYC 1KYD 1KYF 1KYH 1KYI 1KYN 1KYO 1KYP 1KYQ 1KYR 1KYS 1KYT 1KYU 1KYV 1KYW 1KYX 1KYY 1KYZ 1KZ1 1KZ4 1KZ6 1KZ7 1KZ8 1KZ9 1KZA 1KZB 1KZC 1KZD 1KZE 1KZF 1KZG 1KZH 1KZI 1KZJ 1KZK 1KZL 1KZM 1KZN 1KZO 1KZP 1KZQ 1KZU 1KZY 1KZZ 1L00 1L01 1L02 1L03 1L04 1L05 1L06 1L07 1L08 1L09 1L0A 1L0B 1L0C 1L0D 1L0E 1L0F 1L0G 1L0H 1L0I 1L0J 1L0K 1L0L 1L0N 1L0O 1L0P 1L0Q 1L0S 1L0V 1L0W 1L0X 1L0Y 1L0Z 1L10 1L11 1L12 1L13 1L14 1L15 1L16 1L17 1L18 1L19 1L1D 1L1E 1L1F 1L1G 1L1J 1L1L 1L1N 1L1O 1L1Q 1L1R 1L1S 1L1Y 1L20 1L21 1L22 1L23 1L24 1L25 1L26 1L27 1L28 1L29 1L2A 1L2E 1L2F 1L2G 1L2H 1L2I 1L2J 1L2L 1L2O 1L2P 1L2Q 1L2S 1L2T 1L2U 1L2W 1L30 1L31 1L32 1L33 1L34 1L35 1L36 1L37 1L38 1L39 1L3A 1L3B 1L3C 1L3F 1L3I 1L3J 1L3K 1L3P 1L3R 1L3W 1L40 1L41 1L42 1L43 1L44 1L45 1L46 1L47 1L48 1L49 1L4A 1L4B 1L4D 1L4E 1L4F 1L4G 1L4H 1L4I 1L4K 1L4L 1L4M 1L4N 1L4U 1L4X 1L4Y 1L4Z 1L50 1L51 1L52 1L53 1L54 1L55 1L56 1L57 1L58 1L59 1L5A 1L5B 1L5F 1L5G 1L5H 1L5J 1L5K 1L5L 1L5M 1L5N 1L5O 1L5P 1L5Q 1L5R 1L5S 1L5T 1L5V 1L5W 1L5X 1L5Y 1L5Z 1L60 1L61 1L62 1L63 1L64 1L65 1L66 1L67 1L68 1L69 1L6F 1L6G 1L6I 1L6J 1L6L 1L6M 1L6O 1L6P 1L6R 1L6S 1L6W 1L6X 1L6Y 1L6Z 1L70 1L71 1L72 1L73 1L74 1L75 1L76 1L77 1L79 1L7A 1L7C 1L7D 1L7E 1L7F 1L7G 1L7H 1L7I 1L7J 1L7K 1L7L 1L7M 1L7N 1L7O 1L7P 1L7Q 1L7R 1L7T 1L7V 1L7X 1L7Z 1L80 1L81 1L82 1L83 1L84 1L85 1L86 1L87 1L88 1L89 1L8A 1L8B 1L8D 1L8F 1L8G 1L8H 1L8I 1L8J 1L8K 1L8L 1L8N 1L8O 1L8P 1L8Q 1L8R 1L8S 1L8T 1L8W 1L8X 1L90 1L91 1L92 1L93 1L94 1L95 1L96 1L97 1L98 1L99 1L9B 1L9C 1L9D 1L9E 1L9G 1L9H 1L9J 1L9K 1L9L 1L9M 1L9N 1L9O 1L9P 1L9Q 1L9R 1L9S 1L9T 1L9U 1L9V 1L9W 1L9X 1L9Y 1LA1 1LA2 1LA6 1LAA 1LAF 1LAG 1LAH 1LAM 1LAN 1LAP 1LAR 1LAV 1LAW 1LAX 1LAY 1LB1 1LB3 1LB4 1LB5 1LB6 1LB8 1LB9 1LBA 1LBB 1LBC 1LBD 1LBE 1LBF 1LBH 1LBI 1LBK 1LBL 1LBM 1LBQ 1LBS 1LBT 1LBU 1LBV 1LBW 1LBX 1LBY 1LBZ 1LC0 1LC3 1LC5 1LC7 1LC8 1LCA 1LCB 1LCE 1LCF 1LCI 1LCJ 1LCK 1LCL 1LCN 1LCO 1LCP 1LCS 1LCT 1LCU 1LCV 1LCW 1LCY 1LCZ 1LD3 1LD7 1LD8 1LD9 1LDA 1LDB 1LDC 1LDD 1LDE 1LDF 1LDG 1LDI 1LDJ 1LDK 1LDM 1LDN 1LDO 1LDP 1LDQ 1LDS 1LDT 1LDY 1LE2 1LE4 1LE6 1LE7 1LEC 1LED 1LEE 1LEG 1LEH 1LEK 1LEL 1LEM 1LEN 1LEO 1LEP 1LES 1LEV 1LEW 1LEZ 1LF0 1LF1 1LF2 1LF3 1LF4 1LF5 1LF6 1LF7 1LF8 1LF9 1LFA 1LFB 1LFD 1LFG 1LFH 1LFI 1LFK 1LFL 1LFM 1LFO 1LFP 1LFQ 1LFT 1LFV 1LFW 1LFY 1LFZ 1LG1 1LG2 1LG5 1LG6 1LG7 1LG9 1LGA 1LGB 1LGC 1LGD 1LGF 1LGH 1LGN 1LGP 1LGQ 1LGR 1LGT 1LGU 1LGV 1LGW 1LGX 1LGY 1LH0 1LH1 1LH2 1LH3 1LH5 1LH6 1LH7 1LHC 1LHD 1LHE 1LHF 1LHG 1LHH 1LHI 1LHJ 1LHK 1LHL 1LHM 1LHN 1LHO 1LHP 1LHR 1LHS 1LHT 1LHU 1LHV 1LHW 1LHY 1LHZ 1LI0 1LI1 1LI2 1LI3 1LI4 1LI5 1LI6 1LI7 1LI9 1LIA 1LIB 1LIC 1LID 1LIE 1LIF 1LIH 1LII 1LIJ 1LIK 1LIL 1LIN 1LIO 1LIS 1LIT 1LJ0 1LJ1 1LJ2 1LJ3 1LJ4 1LJ5 1LJ7 1LJ8 1LJ9 1LJE 1LJF 1LJG 1LJH 1LJI 1LJJ 1LJK 1LJL 1LJM 1LJN 1LJO 1LJP 1LJR 1LJT 1LJU 1LJW 1LJY 1LK0 1LK2 1LK3 1LK5 1LK6 1LK7 1LK9 1LKA 1LKB 1LKC 1LKD 1LKE 1LKF 1LKI 1LKK 1LKL 1LKM 1LKO 1LKP 1LKR 1LKS 1LKT 1LKV 1LKX 1LKY 1LKZ 1LL0 1LL1 1LL2 1LL3 1LL4 1LL5 1LL6 1LL7 1LL9 1LLA 1LLB 1LLC 1LLD 1LLF 1LLH 1LLN 1LLO 1LLP 1LLQ 1LLR 1LLS 1LLT 1LLU 1LLW 1LLZ 1LM1 1LM3 1LM4 1LM5 1LM6 1LM7 1LM8 1LMA 1LMC 1LME 1LMH 1LMI 1LMK 1LML 1LMN 1LMO 1LMP 1LMQ 1LMT 1LMW 1LN0 1LN1 1LN2 1LN3 1LN4 1LN8 1LNA 1LNB 1LNC 1LND 1LNE 1LNF 1LNH 1LNI 1LNL 1LNM 1LNQ 1LNS 1LNU 1LNW 1LNX 1LNY 1LNZ 1LO0 1LO2 1LO3 1LO4 1LO5 1LO6 1LO7 1LO8 1LO9 1LOA 1LOB 1LOC 1LOD 1LOE 1LOF 1LOG 1LOH 1LOJ 1LOK 1LOL 1LOM 1LON 1LOO 1LOP 1LOQ 1LOR 1LOS 1LOT 1LOU 1LOV 1LOW 1LOX 1LOY 1LOZ 1LP1 1LP3 1LP4 1LP6 1LP8 1LP9 1LPA 1LPB 1LPC 1LPD 1LPE 1LPF 1LPG 1LPH 1LPI 1LPJ 1LPK 1LPL 1LPM 1LPN 1LPO 1LPP 1LPS 1LPU 1LPY 1LPZ 1LQ0 1LQ2 1LQ8 1LQ9 1LQA 1LQB 1LQD 1LQE 1LQF 1LQG 1LQJ 1LQK 1LQL 1LQM 1LQO 1LQP 1LQS 1LQT 1LQU 1LQV 1LQW 1LQX 1LQY 1LR0 1LR2 1LR3 1LR4 1LR5 1LR6 1LR7 1LR8 1LR9 1LRA 1LRH 1LRI 1LRJ 1LRK 1LRL 1LRM 1LRN 1LRO 1LRP 1LRQ 1LRT 1LRU 1LRV 1LRW 1LRY 1LRZ 1LS1 1LS3 1LS5 1LS6 1LS9 1LSA 1LSB 1LSC 1LSD 1LSE 1LSF 1LSG 1LSH 1LSJ 1LSL 1LSM 1LSN 1LSO 1LSP 1LSQ 1LSS 1LST 1LSU 1LSV 1LSW 1LSX 1LSY 1LSZ 1LT0 1LT1 1LT3 1LT4 1LT5 1LT6 1LT7 1LT8 1LT9 1LTA 1LTB 1LTD 1LTE 1LTG 1LTH 1LTI 1LTJ 1LTK 1LTL 1LTM 1LTO 1LTQ 1LTR 1LTS 1LTT 1LTU 1LTV 1LTW 1LTX 1LTZ 1LU0 1LU1 1LU2 1LU4 1LU9 1LUA 1LUC 1LUE 1LUF 1LUG 1LUJ 1LUL 1LUQ 1LUR 1LUV 1LUW 1LUZ 1LV0 1LV1 1LV2 1LV7 1LV8 1LVA 1LVB 1LVC 1LVE 1LVF 1LVG 1LVH 1LVK 1LVL 1LVM 1LVN 1LVO 1LVU 1LVW 1LVY 1LW0 1LW1 1LW2 1LW3 1LW4 1LW5 1LW6 1LW7 1LW9 1LWB 1LWC 1LWD 1LWE 1LWF 1LWG 1LWH 1LWI 1LWJ 1LWK 1LWL 1LWN 1LWO 1LWU 1LWX 1LX5 1LX6 1LX7 1LXA 1LXC 1LXD 1LXE 1LXI 1LXJ 1LXK 1LXM 1LXN 1LXT 1LXY 1LXZ 1LY0 1LY1 1LY2 1LY3 1LY4 1LY8 1LY9 1LYA 1LYB 1LYC 1LYD 1LYE 1LYF 1LYG 1LYH 1LYI 1LYJ 1LYK 1LYL 1LYN 1LYO 1LYQ 1LYS 1LYV 1LYW 1LYX 1LYY 1LYZ 1LZ0 1LZ1 1LZ2 1LZ4 1LZ5 1LZ6 1LZ7 1LZ8 1LZ9 1LZA 1LZB 1LZC 1LZD 1LZE 1LZG 1LZH 1LZI 1LZJ 1LZK 1LZL 1LZO 1LZQ 1LZR 1LZS 1LZT 1LZV 1LZW 1LZX 1LZY 1LZZ 1M00 1M01 1M03 1M04 1M05 1M08 1M0B 1M0D 1M0I 1M0K 1M0L 1M0M 1M0N 1M0O 1M0P 1M0Q 1M0S 1M0T 1M0U 1M0W 1M0Z 1M10 1M13 1M14 1M15 1M16 1M17 1M1B 1M1C 1M1D 1M1E 1M1F 1M1G 1M1H 1M1J 1M1L 1M1M 1M1N 1M1O 1M1P 1M1Q 1M1R 1M1S 1M1T 1M1U 1M1X 1M1Y 1M1Z 1M20 1M21 1M22 1M24 1M26 1M27 1M2A 1M2B 1M2D 1M2G 1M2H 1M2I 1M2J 1M2K 1M2M 1M2N 1M2O 1M2P 1M2Q 1M2R 1M2T 1M2V 1M2W 1M2X 1M2Z 1M32 1M33 1M34 1M35 1M38 1M3D 1M3E 1M3I 1M3J 1M3K 1M3S 1M3U 1M3W 1M3X 1M3Y 1M3Z 1M40 1M41 1M43 1M44 1M45 1M46 1M47 1M48 1M49 1M4A 1M4B 1M4C 1M4D 1M4G 1M4H 1M4I 1M4J 1M4K 1M4L 1M4M 1M4N 1M4R 1M4S 1M4T 1M4U 1M4V 1M4W 1M4Y 1M4Z 1M51 1M52 1M53 1M54 1M55 1M56 1M57 1M59 1M5A 1M5B 1M5C 1M5D 1M5E 1M5F 1M5H 1M5I 1M5N 1M5Q 1M5S 1M5T 1M5U 1M5W 1M5Y 1M61 1M63 1M64 1M65 1M66 1M67 1M68 1M6B 1M6C 1M6D 1M6E 1M6H 1M6I 1M6J 1M6K 1M6M 1M6N 1M6O 1M6P 1M6S 1M6T 1M6U 1M6V 1M6W 1M6Y 1M6Z 1M70 1M71 1M72 1M73 1M74 1M75 1M76 1M78 1M79 1M7A 1M7B 1M7D 1M7E 1M7G 1M7H 1M7I 1M7J 1M7N 1M7O 1M7P 1M7Q 1M7R 1M7S 1M7U 1M7V 1M7W 1M7X 1M7Y 1M7Z 1M83 1M85 1M8A 1M8D 1M8E 1M8F 1M8G 1M8H 1M8I 1M8J 1M8K 1M8N 1M8P 1M8R 1M8S 1M8T 1M8U 1M8Z 1M93 1M98 1M99 1M9A 1M9B 1M9C 1M9D 1M9E 1M9F 1M9H 1M9I 1M9J 1M9K 1M9M 1M9N 1M9P 1M9Q 1M9R 1M9S 1M9T 1M9U 1M9X 1M9Y 1M9Z 1MA0 1MA1 1MA3 1MA9 1MAA 1MAB 1MAC 1MAE 1MAF 1MAH 1MAI 1MAL 1MAM 1MAP 1MAQ 1MAR 1MAS 1MAT 1MAU 1MAV 1MAW 1MAX 1MAY 1MAZ 1MB0 1MB1 1MB2 1MB3 1MB4 1MB8 1MB9 1MBA 1MBB 1MBC 1MBD 1MBI 1MBL 1MBM 1MBN 1MBO 1MBQ 1MBS 1MBT 1MBU 1MBV 1MBX 1MBY 1MBZ 1MC0 1MC1 1MC2 1MC3 1MC4 1MC5 1MC8 1MC9 1MCB 1MCC 1MCD 1MCE 1MCF 1MCH 1MCI 1MCJ 1MCK 1MCL 1MCN 1MCO 1MCP 1MCQ 1MCR 1MCS 1MCT 1MCV 1MCW 1MCX 1MCY 1MCZ 1MD0 1MD2 1MD3 1MD4 1MD6 1MD7 1MD8 1MD9 1MDA 1MDB 1MDC 1MDF 1MDL 1MDN 1MDO 1MDP 1MDQ 1MDR 1MDT 1MDU 1MDV 1MDW 1MDX 1MDZ 1ME3 1ME4 1ME5 1ME6 1ME7 1ME8 1ME9 1MEC 1MEE 1MEG 1MEH 1MEI 1MEJ 1MEL 1MEM 1MEN 1MEO 1MEP 1MER 1MES 1MET 1MEU 1MEW 1MEX 1MEZ 1MF0 1MF1 1MF2 1MF4 1MF7 1MF8 1MFA 1MFB 1MFC 1MFD 1MFE 1MFF 1MFG 1MFI 1MFL 1MFM 1MFP 1MFR 1MFT 1MFU 1MFV 1MFW 1MFZ 1MG0 1MG1 1MG2 1MG3 1MG4 1MG5 1MG6 1MG7 1MG9 1MGN 1MGO 1MGP 1MGQ 1MGR 1MGT 1MGV 1MGW 1MGY 1MH0 1MH1 1MH2 1MH3 1MH4 1MH5 1MH7 1MH8 1MH9 1MHC 1MHE 1MHH 1MHL 1MHM 1MHN 1MHO 1MHP 1MHQ 1MHW 1MHX 1MHY 1MHZ 1MI0 1MI1 1MI3 1MI4 1MI5 1MI7 1MI8 1MID 1MIE 1MIF 1MIH 1MIJ 1MIK 1MIL 1MIM 1MIO 1MIQ 1MIR 1MIU 1MIV 1MIW 1MIX 1MIY 1MIZ 1MJ0 1MJ3 1MJ4 1MJ5 1MJ7 1MJ8 1MJ9 1MJA 1MJB 1MJC 1MJF 1MJG 1MJH 1MJJ 1MJK 1MJL 1MJN 1MJS 1MJT 1MJU 1MJV 1MJW 1MJX 1MJY 1MJZ 1MK0 1MK1 1MK2 1MK4 1MK5 1MK7 1MK8 1MK9 1MKA 1MKB 1MKD 1MKF 1MKG 1MKH 1MKI 1MKJ 1MKK 1MKM 1MKO 1MKP 1MKQ 1MKR 1MKS 1MKT 1MKU 1MKV 1MKW 1MKX 1MKY 1MKZ 1ML0 1ML1 1ML2 1ML3 1ML4 1ML6 1ML7 1ML8 1ML9 1MLA 1MLB 1MLC 1MLD 1MLF 1MLG 1MLH 1MLI 1MLJ 1MLK 1MLL 1MLM 1MLN 1MLO 1MLQ 1MLR 1MLS 1MLU 1MLV 1MLW 1MLY 1MLZ 1MM6 1MM7 1MM9 1MMA 1MMB 1MMD 1MMF 1MMG 1MMI 1MMJ 1MMK 1MML 1MMM 1MMN 1MMO 1MMP 1MMQ 1MMR 1MMT 1MMU 1MMV 1MMW 1MMX 1MMY 1MMZ 1MN0 1MN1 1MN2 1MN3 1MN4 1MN6 1MN7 1MN8 1MN9 1MNA 1MNC 1MND 1MNE 1MNF 1MNG 1MNH 1MNI 1MNJ 1MNK 1MNO 1MNP 1MNQ 1MNS 1MNU 1MNZ 1MO0 1MO1 1MO2 1MO3 1MO4 1MO5 1MO6 1MO9 1MOA 1MOB 1MOC 1MOD 1MOE 1MOF 1MOG 1MOH 1MOJ 1MOK 1MOL 1MOM 1MOO 1MOP 1MOQ 1MOR 1MOS 1MOU 1MOV 1MOX 1MOY 1MOZ 1MP0 1MP2 1MP3 1MP4 1MP5 1MP8 1MP9 1MPA 1MPB 1MPC 1MPD 1MPF 1MPG 1MPJ 1MPL 1MPM 1MPN 1MPO 1MPP 1MPQ 1MPR 1MPS 1MPT 1MPU 1MPW 1MPX 1MPY 1MQ0 1MQ4 1MQ5 1MQ6 1MQ7 1MQ8 1MQ9 1MQA 1MQB 1MQD 1MQE 1MQF 1MQG 1MQH 1MQI 1MQJ 1MQK 1MQL 1MQM 1MQN 1MQO 1MQP 1MQQ 1MQR 1MQS 1MQT 1MQV 1MQW 1MR1 1MR2 1MR3 1MR5 1MR7 1MR8 1MR9 1MRA 1MRC 1MRD 1MRE 1MRF 1MRG 1MRH 1MRI 1MRJ 1MRK 1MRL 1MRN 1MRO 1MRP 1MRQ 1MRR 1MRS 1MRU 1MRV 1MRW 1MRX 1MRY 1MRZ 1MS0 1MS1 1MS3 1MS4 1MS5 1MS6 1MS7 1MS8 1MS9 1MSA 1MSB 1MSC 1MSD 1MSI 1MSJ 1MSK 1MSM 1MSN 1MSO 1MSP 1MSS 1MST 1MSV 1MT0 1MT1 1MT3 1MT5 1MT6 1MT7 1MT8 1MT9 1MTB 1MTC 1MTI 1MTJ 1MTK 1MTN 1MTO 1MTP 1MTR 1MTS 1MTU 1MTV 1MTW 1MTY 1MTZ 1MU0 1MU2 1MU4 1MU5 1MU6 1MU7 1MU8 1MU9 1MUA 1MUC 1MUD 1MUE 1MUF 1MUG 1MUI 1MUJ 1MUK 1MUL 1MUM 1MUN 1MUO 1MUP 1MUQ 1MUU 1MUW 1MUY 1MV5 1MV8 1MV9 1MVA 1MVB 1MVC 1MVE 1MVF 1MVH 1MVK 1MVL 1MVN 1MVO 1MVP 1MVQ 1MVS 1MVT 1MVU 1MVX 1MVY 1MW0 1MW1 1MW2 1MW3 1MW5 1MW7 1MW9 1MWA 1MWC 1MWD 1MWE 1MWH 1MWK 1MWM 1MWO 1MWP 1MWQ 1MWR 1MWS 1MWT 1MWU 1MWV 1MWW 1MX0 1MX1 1MX2 1MX3 1MX4 1MX5 1MX6 1MX9 1MXA 1MXB 1MXC 1MXD 1MXE 1MXF 1MXG 1MXH 1MXI 1MXO 1MXR 1MXS 1MXT 1MXU 1MXV 1MXW 1MXX 1MXY 1MXZ 1MY0 1MY1 1MY2 1MY3 1MY4 1MY5 1MY6 1MY7 1MY8 1MYG 1MYH 1MYI 1MYJ 1MYK 1MYL 1MYM 1MYP 1MYR 1MYT 1MYW 1MYZ 1MZ0 1MZ4 1MZ5 1MZ6 1MZ8 1MZ9 1MZA 1MZB 1MZC 1MZD 1MZE 1MZF 1MZG 1MZH 1MZJ 1MZL 1MZM 1MZN 1MZO 1MZR 1MZS 1MZU 1MZV 1MZW 1MZY 1MZZ 1N00 1N04 1N05 1N06 1N07 1N08 1N0E 1N0F 1N0G 1N0H 1N0I 1N0J 1N0L 1N0N 1N0Q 1N0R 1N0S 1N0T 1N0U 1N0V 1N0W 1N0X 1N0Y 1N10 1N11 1N12 1N13 1N15 1N18 1N19 1N1A 1N1B 1N1C 1N1D 1N1E 1N1F 1N1G 1N1I 1N1J 1N1L 1N1M 1N1P 1N1Q 1N1S 1N1T 1N1V 1N1X 1N1Y 1N1Z 1N20 1N21 1N22 1N23 1N24 1N25 1N26 1N28 1N29 1N2A 1N2B 1N2C 1N2D 1N2E 1N2F 1N2G 1N2H 1N2I 1N2J 1N2K 1N2L 1N2M 1N2N 1N2O 1N2R 1N2S 1N2T 1N2V 1N2X 1N2Z 1N31 1N3B 1N3I 1N3L 1N3N 1N3O 1N3P 1N3Q 1N3R 1N3S 1N3T 1N3U 1N3W 1N3X 1N3Y 1N3Z 1N40 1N41 1N42 1N43 1N44 1N45 1N46 1N47 1N49 1N4A 1N4D 1N4F 1N4G 1N4H 1N4J 1N4K 1N4M 1N4O 1N4P 1N4Q 1N4R 1N4S 1N4U 1N4V 1N4W 1N4X 1N50 1N51 1N52 1N54 1N55 1N57 1N59 1N5A 1N5B 1N5D 1N5I 1N5J 1N5K 1N5L 1N5M 1N5N 1N5O 1N5Q 1N5R 1N5S 1N5T 1N5U 1N5V 1N5W 1N5X 1N5Z 1N60 1N61 1N62 1N63 1N64 1N67 1N68 1N69 1N6A 1N6B 1N6C 1N6D 1N6E 1N6F 1N6H 1N6I 1N6K 1N6L 1N6M 1N6N 1N6O 1N6P 1N6R 1N6X 1N6Y 1N70 1N71 1N73 1N75 1N76 1N7D 1N7E 1N7F 1N7G 1N7H 1N7I 1N7J 1N7K 1N7M 1N7N 1N7O 1N7P 1N7Q 1N7R 1N7S 1N7U 1N7V 1N7W 1N7X 1N7Y 1N7Z 1N80 1N81 1N82 1N83 1N84 1N86 1N8B 1N8E 1N8F 1N8I 1N8J 1N8K 1N8N 1N8O 1N8P 1N8Q 1N8S 1N8T 1N8U 1N8V 1N8W 1N8Y 1N8Z 1N90 1N92 1N93 1N94 1N95 1N97 1N99 1N9A 1N9B 1N9E 1N9F 1N9G 1N9H 1N9I 1N9K 1N9L 1N9M 1N9N 1N9O 1N9P 1N9R 1N9S 1N9W 1N9X 1N9Y 1N9Z 1NA0 1NA1 1NA3 1NA5 1NA6 1NA7 1NA8 1NAA 1NAE 1NAF 1NAG 1NAH 1NAI 1NAK 1NAL 1NAM 1NAN 1NAP 1NAQ 1NAR 1NAS 1NAT 1NAV 1NAW 1NAX 1NAY 1NAZ 1NB0 1NB2 1NB3 1NB4 1NB5 1NB6 1NB8 1NB9 1NBA 1NBB 1NBC 1NBE 1NBF 1NBH 1NBI 1NBM 1NBO 1NBP 1NBQ 1NBU 1NBV 1NBW 1NBX 1NBY 1NBZ 1NC1 1NC2 1NC3 1NC4 1NC5 1NC6 1NC7 1NC9 1NCA 1NCB 1NCC 1NCD 1NCE 1NCF 1NCG 1NCH 1NCI 1NCJ 1NCL 1NCN 1NCO 1NCQ 1NCR 1NCW 1NCX 1NCY 1NCZ 1ND0 1ND1 1ND2 1ND3 1ND4 1ND5 1ND6 1ND7 1NDA 1NDB 1NDC 1NDD 1NDE 1NDF 1NDG 1NDH 1NDI 1NDJ 1NDK 1NDL 1NDM 1NDO 1NDP 1NDQ 1NDR 1NDS 1NDT 1NDU 1NDV 1NDW 1NDY 1NDZ 1NE2 1NE4 1NE6 1NE7 1NE8 1NE9 1NEC 1NED 1NEG 1NEJ 1NEK 1NEL 1NEN 1NEP 1NES 1NEU 1NEX 1NEY 1NEZ 1NF0 1NF1 1NF2 1NF3 1NF4 1NF5 1NF6 1NF7 1NF8 1NF9 1NFB 1NFD 1NFF 1NFG 1NFH 1NFI 1NFJ 1NFN 1NFO 1NFP 1NFQ 1NFR 1NFS 1NFT 1NFU 1NFV 1NFW 1NFX 1NFY 1NFZ 1NG0 1NG1 1NG2 1NG3 1NG4 1NG5 1NG6 1NGA 1NGB 1NGC 1NGD 1NGE 1NGF 1NGG 1NGH 1NGI 1NGJ 1NGK 1NGN 1NGP 1NGQ 1NGS 1NGW 1NGX 1NGY 1NGZ 1NH0 1NH1 1NH6 1NH7 1NH8 1NH9 1NHB 1NHC 1NHE 1NHG 1NHH 1NHI 1NHJ 1NHK 1NHL 1NHP 1NHQ 1NHR 1NHS 1NHT 1NHU 1NHV 1NHW 1NHX 1NHY 1NHZ 1NI0 1NI1 1NI2 1NI3 1NI4 1NI5 1NI6 1NI9 1NIA 1NIB 1NIC 1NID 1NIE 1NIF 1NIG 1NIH 1NIJ 1NIK 1NIO 1NIP 1NIR 1NIS 1NIT 1NIU 1NIV 1NIW 1NJ1 1NJ2 1NJ4 1NJ5 1NJ6 1NJ8 1NJ9 1NJA 1NJB 1NJC 1NJD 1NJE 1NJF 1NJG 1NJH 1NJJ 1NJK 1NJR 1NJS 1NJT 1NJU 1NK1 1NKD 1NKG 1NKH 1NKI 1NKK 1NKM 1NKN 1NKO 1NKQ 1NKR 1NKS 1NKT 1NKV 1NKX 1NKZ 1NL0 1NL1 1NL2 1NL3 1NL4 1NL5 1NL6 1NL7 1NL9 1NLB 1NLD 1NLF 1NLI 1NLJ 1NLK 1NLM 1NLN 1NLQ 1NLR 1NLS 1NLT 1NLU 1NLV 1NLX 1NLY 1NLZ 1NM0 1NM1 1NM2 1NM3 1NM5 1NM6 1NM8 1NM9 1NMA 1NMB 1NMC 1NMD 1NME 1NMK 1NML 1NMM 1NMN 1NMO 1NMP 1NMQ 1NMS 1NMT 1NMU 1NMX 1NMY 1NMZ 1NN0 1NN1 1NN2 1NN3 1NN4 1NN5 1NN6 1NN7 1NNA 1NNB 1NNC 1NND 1NNF 1NNH 1NNI 1NNK 1NNL 1NNO 1NNP 1NNQ 1NNR 1NNS 1NNT 1NNU 1NNW 1NNX 1NNY 1NO1 1NO3 1NO4 1NO5 1NO6 1NO7 1NO9 1NOA 1NOB 1NOC 1NOD 1NOF 1NOG 1NOH 1NOI 1NOJ 1NOK 1NOL 1NOM 1NON 1NOO 1NOS 1NOT 1NOU 1NOV 1NOW 1NOX 1NOZ 1NP0 1NP1 1NP2 1NP3 1NP4 1NP6 1NP7 1NP8 1NPA 1NPB 1NPC 1NPD 1NPE 1NPF 1NPG 1NPH 1NPI 1NPJ 1NPK 1NPL 1NPM 1NPN 1NPO 1NPP 1NPR 1NPS 1NPT 1NPU 1NPV 1NPW 1NPX 1NPY 1NPZ 1NQ0 1NQ1 1NQ2 1NQ3 1NQ5 1NQ6 1NQ7 1NQ9 1NQA 1NQB 1NQC 1NQD 1NQE 1NQF 1NQG 1NQH 1NQI 1NQJ 1NQK 1NQL 1NQM 1NQN 1NQO 1NQP 1NQT 1NQU 1NQV 1NQW 1NQX 1NQY 1NQZ 1NR0 1NR1 1NR2 1NR4 1NR5 1NR6 1NR7 1NR9 1NRF 1NRG 1NRI 1NRJ 1NRK 1NRL 1NRN 1NRO 1NRP 1NRQ 1NRR 1NRS 1NRV 1NRW 1NRX 1NRZ 1NS0 1NS2 1NS3 1NS4 1NS5 1NS6 1NS7 1NS8 1NS9 1NSA 1NSB 1NSC 1NSD 1NSE 1NSF 1NSG 1NSI 1NSJ 1NSK 1NSL 1NSM 1NSN 1NSP 1NSQ 1NSR 1NSS 1NST 1NSU 1NSV 1NSW 1NSX 1NSY 1NSZ 1NT0 1NT1 1NT2 1NT3 1NT4 1NT9 1NTD 1NTE 1NTF 1NTG 1NTH 1NTK 1NTM 1NTN 1NTO 1NTV 1NTY 1NTZ 1NU0 1NU1 1NU2 1NU3 1NU4 1NU5 1NU6 1NU7 1NU8 1NU9 1NUA 1NUB 1NUC 1NUD 1NUE 1NUF 1NUG 1NUH 1NUI 1NUK 1NUL 1NUN 1NUO 1NUP 1NUQ 1NUR 1NUS 1NUT 1NUU 1NUW 1NUX 1NUY 1NUZ 1NV0 1NV1 1NV2 1NV3 1NV4 1NV5 1NV6 1NV7 1NV8 1NV9 1NVA 1NVB 1NVD 1NVE 1NVF 1NVG 1NVI 1NVJ 1NVK 1NVM 1NVQ 1NVR 1NVS 1NVT 1NVU 1NVV 1NVW 1NVX 1NW1 1NW2 1NW3 1NW4 1NW5 1NW6 1NW7 1NW8 1NW9 1NWA 1NWC 1NWE 1NWG 1NWH 1NWI 1NWK 1NWL 1NWM 1NWN 1NWO 1NWP 1NWR 1NWS 1NWT 1NWU 1NWW 1NWZ 1NX0 1NX1 1NX2 1NX3 1NX4 1NX6 1NX8 1NX9 1NXB 1NXC 1NXD 1NXE 1NXF 1NXG 1NXH 1NXJ 1NXK 1NXM 1NXO 1NXP 1NXQ 1NXS 1NXT 1NXU 1NXV 1NXW 1NXX 1NXY 1NXZ 1NY0 1NY1 1NY2 1NY3 1NY5 1NY6 1NY7 1NYC 1NYE 1NYH 1NYK 1NYL 1NYM 1NYQ 1NYR 1NYS 1NYT 1NYU 1NYW 1NYX 1NYY 1NZ0 1NZ2 1NZ3 1NZ4 1NZ5 1NZ6 1NZ7 1NZA 1NZC 1NZD 1NZE 1NZF 1NZI 1NZJ 1NZK 1NZL 1NZN 1NZO 1NZQ 1NZR 1NZU 1NZV 1NZW 1NZX 1NZY 1NZZ 1O00 1O01 1O02 1O03 1O04 1O05 1O06 1O07 1O08 1O0A 1O0D 1O0E 1O0F 1O0H 1O0M 1O0N 1O0O 1O0Q 1O0R 1O0S 1O0T 1O0V 1O0W 1O0X 1O12 1O13 1O16 1O17 1O1H 1O1I 1O1J 1O1K 1O1L 1O1M 1O1N 1O1O 1O1P 1O1R 1O1S 1O1T 1O1X 1O1Y 1O1Z 1O20 1O22 1O23 1O24 1O25 1O26 1O27 1O28 1O29 1O2A 1O2B 1O2D 1O2E 1O2G 1O2H 1O2I 1O2J 1O2K 1O2L 1O2M 1O2N 1O2O 1O2P 1O2Q 1O2R 1O2S 1O2T 1O2U 1O2V 1O2W 1O2X 1O2Y 1O2Z 1O30 1O31 1O32 1O33 1O34 1O35 1O36 1O37 1O38 1O39 1O3A 1O3B 1O3C 1O3D 1O3E 1O3F 1O3G 1O3H 1O3I 1O3J 1O3K 1O3L 1O3M 1O3N 1O3O 1O3P 1O3U 1O3W 1O3X 1O3Y 1O41 1O42 1O43 1O44 1O45 1O46 1O47 1O48 1O49 1O4A 1O4B 1O4C 1O4D 1O4E 1O4F 1O4G 1O4H 1O4I 1O4J 1O4K 1O4L 1O4M 1O4N 1O4O 1O4P 1O4Q 1O4R 1O4S 1O4T 1O4U 1O4V 1O4W 1O4Y 1O4Z 1O50 1O51 1O54 1O57 1O58 1O59 1O5A 1O5B 1O5C 1O5D 1O5E 1O5F 1O5G 1O5H 1O5I 1O5J 1O5K 1O5L 1O5M 1O5O 1O5Q 1O5R 1O5T 1O5U 1O5W 1O5X 1O5Z 1O60 1O61 1O62 1O63 1O64 1O65 1O66 1O67 1O68 1O69 1O6A 1O6B 1O6C 1O6D 1O6E 1O6F 1O6G 1O6H 1O6I 1O6J 1O6K 1O6L 1O6O 1O6P 1O6Q 1O6R 1O6S 1O6T 1O6U 1O6V 1O6Y 1O6Z 1O70 1O71 1O72 1O73 1O75 1O76 1O77 1O79 1O7A 1O7D 1O7E 1O7F 1O7G 1O7H 1O7I 1O7J 1O7K 1O7L 1O7M 1O7N 1O7O 1O7P 1O7Q 1O7S 1O7T 1O7U 1O7V 1O7W 1O7X 1O7Y 1O7Z 1O80 1O81 1O82 1O83 1O84 1O85 1O86 1O87 1O88 1O89 1O8A 1O8B 1O8C 1O8D 1O8E 1O8F 1O8G 1O8H 1O8I 1O8J 1O8K 1O8L 1O8M 1O8N 1O8O 1O8P 1O8Q 1O8S 1O8U 1O8V 1O8W 1O8X 1O90 1O91 1O92 1O93 1O94 1O95 1O96 1O97 1O98 1O99 1O9B 1O9C 1O9D 1O9E 1O9F 1O9G 1O9H 1O9I 1O9J 1O9K 1O9L 1O9N 1O9O 1O9P 1O9Q 1O9R 1O9S 1O9T 1O9U 1O9V 1O9W 1O9X 1O9Y 1O9Z 1OA0 1OA1 1OA2 1OA3 1OA4 1OA7 1OA8 1OA9 1OAA 1OAB 1OAC 1OAD 1OAE 1OAF 1OAG 1OAH 1OAI 1OAJ 1OAK 1OAL 1OAN 1OAO 1OAP 1OAQ 1OAR 1OAS 1OAT 1OAU 1OAX 1OAY 1OAZ 1OB0 1OB1 1OB3 1OB4 1OB6 1OB7 1OB8 1OB9 1OBA 1OBB 1OBC 1OBD 1OBF 1OBG 1OBH 1OBI 1OBJ 1OBK 1OBL 1OBM 1OBN 1OBO 1OBP 1OBQ 1OBR 1OBS 1OBT 1OBU 1OBV 1OBW 1OBX 1OBY 1OBZ 1OC0 1OC1 1OC2 1OC3 1OC4 1OC5 1OC6 1OC7 1OC8 1OC9 1OCB 1OCC 1OCE 1OCH 1OCJ 1OCK 1OCL 1OCM 1OCN 1OCO 1OCQ 1OCR 1OCS 1OCU 1OCV 1OCW 1OCX 1OCY 1OCZ 1OD0 1OD1 1OD2 1OD3 1OD4 1OD5 1OD6 1OD7 1OD8 1OD9 1ODA 1ODB 1ODC 1ODD 1ODE 1ODF 1ODI 1ODJ 1ODK 1ODL 1ODM 1ODN 1ODO 1ODS 1ODT 1ODU 1ODV 1ODW 1ODX 1ODY 1ODZ 1OE0 1OE1 1OE2 1OE3 1OE7 1OE8 1OE9 1OEB 1OEC 1OEE 1OEJ 1OEK 1OEL 1OEM 1OEN 1OEO 1OEP 1OES 1OET 1OEU 1OEV 1OEW 1OEX 1OEY 1OEZ 1OF0 1OF1 1OF2 1OF3 1OF4 1OF5 1OF6 1OF8 1OFA 1OFB 1OFC 1OFD 1OFE 1OFF 1OFG 1OFH 1OFI 1OFJ 1OFK 1OFL 1OFM 1OFN 1OFO 1OFP 1OFQ 1OFR 1OFS 1OFT 1OFU 1OFV 1OFW 1OFY 1OFZ 1OG0 1OG1 1OG2 1OG3 1OG4 1OG5 1OG6 1OGA 1OGB 1OGC 1OGD 1OGE 1OGF 1OGG 1OGH 1OGI 1OGJ 1OGK 1OGL 1OGM 1OGO 1OGP 1OGQ 1OGS 1OGT 1OGU 1OGV 1OGW 1OGX 1OGY 1OGZ 1OH0 1OH2 1OH3 1OH4 1OH9 1OHA 1OHB 1OHC 1OHD 1OHE 1OHF 1OHG 1OHH 1OHJ 1OHK 1OHL 1OHO 1OHP 1OHQ 1OHR 1OHS 1OHT 1OHU 1OHV 1OHW 1OHY 1OHZ 1OI0 1OI1 1OI2 1OI3 1OI4 1OI6 1OI7 1OI8 1OI9 1OIA 1OIB 1OID 1OIE 1OIF 1OIH 1OII 1OIJ 1OIK 1OIL 1OIM 1OIN 1OIO 1OIP 1OIQ 1OIR 1OIS 1OIT 1OIU 1OIV 1OIW 1OIX 1OIY 1OIZ 1OJ1 1OJ4 1OJ5 1OJ6 1OJ7 1OJ9 1OJA 1OJC 1OJD 1OJH 1OJI 1OJJ 1OJK 1OJL 1OJM 1OJN 1OJO 1OJP 1OJQ 1OJR 1OJT 1OJV 1OJW 1OJX 1OJY 1OJZ 1OK0 1OK1 1OK2 1OK3 1OK4 1OK6 1OK7 1OK8 1OK9 1OKB 1OKC 1OKE 1OKG 1OKH 1OKI 1OKJ 1OKK 1OKL 1OKM 1OKN 1OKO 1OKQ 1OKR 1OKS 1OKT 1OKV 1OKW 1OKX 1OKY 1OKZ 1OL0 1OL1 1OL2 1OL5 1OL6 1OL7 1OLA 1OLC 1OLL 1OLM 1OLO 1OLP 1OLQ 1OLR 1OLS 1OLT 1OLU 1OLX 1OLZ 1OM0 1OM1 1OM3 1OM4 1OM5 1OM6 1OM7 1OM8 1OM9 1OMD 1OME 1OMI 1OMJ 1OMO 1OMP 1OMR 1OMS 1OMV 1OMW 1OMX 1OMY 1OMZ 1ON0 1ON1 1ON2 1ON3 1ON6 1ON7 1ON8 1ON9 1ONA 1ONC 1ONE 1ONF 1ONG 1ONH 1ONI 1ONJ 1ONK 1ONL 1ONN 1ONO 1ONP 1ONQ 1ONR 1ONS 1ONW 1ONX 1ONY 1ONZ 1OO0 1OO2 1OO5 1OO6 1OO8 1OOC 1OOE 1OOF 1OOG 1OOH 1OOI 1OOJ 1OOK 1OON 1OOP 1OOQ 1OOT 1OOW 1OOY 1OOZ 1OP0 1OP2 1OP3 1OP5 1OP8 1OP9 1OPA 1OPB 1OPC 1OPD 1OPE 1OPF 1OPG 1OPH 1OPJ 1OPK 1OPL 1OPM 1OPO 1OPR 1OPS 1OPX 1OPY 1OQ1 1OQ4 1OQ5 1OQ7 1OQ9 1OQB 1OQC 1OQD 1OQE 1OQF 1OQG 1OQH 1OQJ 1OQL 1OQM 1OQN 1OQO 1OQQ 1OQR 1OQS 1OQU 1OQV 1OQW 1OQX 1OR0 1OR2 1OR3 1OR4 1OR6 1OR7 1OR8 1ORB 1ORC 1ORD 1ORE 1ORF 1ORG 1ORH 1ORI 1ORJ 1ORK 1ORO 1ORQ 1ORR 1ORS 1ORT 1ORU 1ORV 1ORW 1ORY 1OS0 1OS1 1OS2 1OS3 1OS4 1OS5 1OS6 1OS7 1OS8 1OS9 1OSA 1OSC 1OSD 1OSE 1OSF 1OSG 1OSH 1OSI 1OSJ 1OSM 1OSN 1OSP 1OSS 1OSV 1OSY 1OSZ 1OT1 1OT2 1OT3 1OT5 1OT6 1OT7 1OT8 1OT9 1OTA 1OTB 1OTD 1OTE 1OTF 1OTG 1OTH 1OTI 1OTJ 1OTK 1OTM 1OTN 1OTO 1OTP 1OTS 1OTT 1OTU 1OTV 1OTW 1OTX 1OTY 1OU0 1OU4 1OU5 1OU6 1OU8 1OU9 1OUA 1OUB 1OUC 1OUD 1OUE 1OUF 1OUG 1OUH 1OUI 1OUJ 1OUK 1OUL 1OUM 1OUN 1OUO 1OUR 1OUS 1OUT 1OUU 1OUV 1OUW 1OUX 1OUY 1OV3 1OV4 1OV5 1OV6 1OV7 1OV8 1OV9 1OVA 1OVB 1OVD 1OVE 1OVG 1OVH 1OVJ 1OVK 1OVL 1OVM 1OVN 1OVO 1OVP 1OVR 1OVS 1OVT 1OVU 1OVV 1OVW 1OVZ 1OW0 1OW1 1OW2 1OW3 1OW4 1OW6 1OW7 1OW8 1OWB 1OWC 1OWD 1OWE 1OWH 1OWI 1OWJ 1OWK 1OWL 1OWM 1OWN 1OWO 1OWP 1OWQ 1OWS 1OWY 1OWZ 1OX0 1OX1 1OX3 1OX4 1OX5 1OX6 1OX7 1OX8 1OX9 1OXA 1OXB 1OXC 1OXD 1OXE 1OXF 1OXG 1OXH 1OXJ 1OXK 1OXL 1OXM 1OXN 1OXO 1OXP 1OXQ 1OXR 1OXS 1OXT 1OXU 1OXV 1OXW 1OXX 1OXY 1OXZ 1OY0 1OY1 1OY3 1OY5 1OY6 1OY7 1OY8 1OY9 1OYA 1OYB 1OYC 1OYD 1OYE 1OYF 1OYG 1OYH 1OYJ 1OYK 1OYL 1OYN 1OYO 1OYP 1OYQ 1OYR 1OYS 1OYT 1OYU 1OYV 1OYW 1OYX 1OYY 1OYZ 1OZ0 1OZ1 1OZ2 1OZ3 1OZ6 1OZ7 1OZ9 1OZA 1OZB 1OZE 1OZF 1OZG 1OZH 1OZL 1OZM 1OZN 1OZP 1OZQ 1OZR 1OZT 1OZU 1OZV 1OZW 1OZY 1P01 1P02 1P03 1P04 1P05 1P06 1P09 1P0B 1P0C 1P0D 1P0E 1P0F 1P0H 1P0I 1P0K 1P0M 1P0N 1P0P 1P0Q 1P0S 1P0V 1P0W 1P0X 1P0Y 1P0Z 1P10 1P11 1P12 1P13 1P14 1P15 1P16 1P17 1P18 1P19 1P1B 1P1C 1P1F 1P1G 1P1H 1P1I 1P1J 1P1K 1P1L 1P1M 1P1N 1P1O 1P1Q 1P1R 1P1U 1P1V 1P1W 1P1X 1P1Z 1P22 1P27 1P28 1P29 1P2A 1P2B 1P2C 1P2D 1P2E 1P2F 1P2G 1P2H 1P2I 1P2J 1P2K 1P2L 1P2M 1P2N 1P2O 1P2P 1P2Q 1P2R 1P2S 1P2T 1P2U 1P2V 1P2X 1P2Y 1P2Z 1P30 1P31 1P32 1P33 1P35 1P36 1P37 1P38 1P39 1P3C 1P3D 1P3E 1P3H 1P3J 1P3N 1P3Q 1P3R 1P3T 1P3U 1P3V 1P3W 1P3Y 1P42 1P43 1P44 1P45 1P46 1P48 1P49 1P4A 1P4B 1P4C 1P4D 1P4F 1P4G 1P4H 1P4I 1P4J 1P4K 1P4L 1P4M 1P4N 1P4O 1P4P 1P4R 1P4T 1P4U 1P4V 1P4X 1P50 1P52 1P53 1P56 1P57 1P5B 1P5C 1P5D 1P5E 1P5F 1P5G 1P5H 1P5J 1P5Q 1P5R 1P5S 1P5T 1P5U 1P5V 1P5X 1P5Y 1P5Z 1P60 1P61 1P62 1P63 1P64 1P65 1P69 1P6A 1P6B 1P6C 1P6D 1P6E 1P6F 1P6H 1P6I 1P6J 1P6K 1P6L 1P6M 1P6N 1P6O 1P6P 1P6W 1P6X 1P6Y 1P72 1P73 1P74 1P75 1P77 1P7B 1P7C 1P7G 1P7I 1P7J 1P7K 1P7L 1P7N 1P7O 1P7P 1P7Q 1P7R 1P7S 1P7T 1P7V 1P7W 1P7Y 1P7Z 1P80 1P81 1P84 1P8C 1P8D 1P8F 1P8H 1P8I 1P8J 1P8L 1P8M 1P8N 1P8O 1P8P 1P8Q 1P8R 1P8S 1P8T 1P8U 1P8V 1P8X 1P8Z 1P90 1P91 1P92 1P93 1P99 1P9A 1P9B 1P9E 1P9G 1P9H 1P9I 1P9L 1P9M 1P9N 1P9O 1P9P 1P9Q 1P9R 1P9S 1P9U 1P9W 1P9Y 1PA0 1PA1 1PA2 1PA3 1PA7 1PA9 1PAD 1PAE 1PAF 1PAG 1PAH 1PAL 1PAM 1PAQ 1PAU 1PAX 1PAZ 1PB0 1PB1 1PB3 1PB7 1PB8 1PB9 1PBB 1PBC 1PBD 1PBE 1PBF 1PBG 1PBH 1PBI 1PBJ 1PBK 1PBN 1PBO 1PBP 1PBQ 1PBT 1PBV 1PBW 1PBX 1PBY 1PC3 1PC4 1PC5 1PC6 1PC8 1PC9 1PCA 1PCF 1PCG 1PCH 1PCI 1PCJ 1PCK 1PCL 1PCM 1PCQ 1PCR 1PCS 1PCV 1PCW 1PCX 1PCZ 1PD0 1PD1 1PD2 1PD3 1PD5 1PD8 1PD9 1PDA 1PDB 1PDG 1PDH 1PDK 1PDO 1PDQ 1PDR 1PDU 1PDV 1PDW 1PDY 1PDZ 1PE0 1PE1 1PE5 1PE6 1PE7 1PE8 1PE9 1PEA 1PEB 1PED 1PEE 1PEF 1PEG 1PEK 1PEM 1PEN 1PEO 1PEQ 1PEU 1PEV 1PEW 1PEX 1PEY 1PEZ 1PF3 1PF5 1PF7 1PF8 1PF9 1PFB 1PFC 1PFF 1PFG 1PFK 1PFO 1PFP 1PFQ 1PFR 1PFU 1PFV 1PFW 1PFX 1PFY 1PFZ 1PG0 1PG2 1PG3 1PG4 1PG5 1PG6 1PG7 1PG8 1PGA 1PGB 1PGE 1PGF 1PGG 1PGI 1PGJ 1PGN 1PGO 1PGP 1PGQ 1PGR 1PGS 1PGT 1PGU 1PGV 1PGW 1PGX 1PH0 1PHA 1PHB 1PHC 1PHD 1PHE 1PHF 1PHG 1PHH 1PHK 1PHM 1PHN 1PHO 1PHP 1PHQ 1PHR 1PHS 1PHT 1PHW 1PHZ 1PI1 1PI2 1PI3 1PI4 1PI5 1PI6 1PID 1PIE 1PIF 1PIG 1PII 1PIL 1PIM 1PIN 1PIO 1PIP 1PIQ 1PIU 1PIV 1PIW 1PIX 1PIY 1PIZ 1PJ0 1PJ1 1PJ2 1PJ3 1PJ4 1PJ5 1PJ6 1PJ7 1PJ8 1PJ9 1PJA 1PJB 1PJC 1PJH 1PJK 1PJL 1PJM 1PJN 1PJP 1PJQ 1PJR 1PJS 1PJT 1PJU 1PJX 1PK0 1PK1 1PK3 1PK4 1PK5 1PK6 1PK7 1PK8 1PK9 1PKD 1PKE 1PKF 1PKG 1PKH 1PKJ 1PKK 1PKL 1PKM 1PKN 1PKO 1PKP 1PKQ 1PKR 1PKU 1PKV 1PKW 1PKX 1PKY 1PKZ 1PL0 1PL1 1PL2 1PL3 1PL4 1PL5 1PL6 1PL7 1PL8 1PL9 1PLC 1PLF 1PLG 1PLJ 1PLK 1PLL 1PLQ 1PLR 1PLU 1PM1 1PM2 1PM3 1PM4 1PM7 1PM9 1PMA 1PMB 1PMD 1PME 1PMH 1PMI 1PMJ 1PMK 1PML 1PMM 1PMN 1PMO 1PMP 1PMT 1PMU 1PMV 1PMY 1PN0 1PN2 1PN3 1PN4 1PN9 1PNC 1PND 1PNE 1PNF 1PNG 1PNK 1PNL 1PNM 1PNO 1PNQ 1PNT 1PNV 1PNZ 1PO0 1PO1 1PO2 1PO3 1PO5 1PO7 1PO8 1PO9 1POA 1POB 1POC 1POD 1POE 1POH 1POI 1POJ 1POK 1POO 1POP 1POS 1POT 1POV 1POW 1POX 1POY 1PP0 1PP1 1PP2 1PP3 1PP4 1PP6 1PP9 1PPA 1PPB 1PPC 1PPD 1PPE 1PPF 1PPG 1PPH 1PPI 1PPJ 1PPK 1PPL 1PPM 1PPN 1PPO 1PPP 1PPR 1PPT 1PPV 1PPW 1PPY 1PPZ 1PQ0 1PQ1 1PQ2 1PQ3 1PQ4 1PQ5 1PQ6 1PQ7 1PQ8 1PQ9 1PQA 1PQC 1PQD 1PQE 1PQF 1PQH 1PQI 1PQJ 1PQK 1PQM 1PQO 1PQP 1PQU 1PQV 1PQW 1PQY 1PQZ 1PR0 1PR1 1PR2 1PR3 1PR4 1PR5 1PR6 1PR9 1PRC 1PRE 1PRG 1PRH 1PRN 1PRO 1PRQ 1PRT 1PRW 1PRX 1PRY 1PRZ 1PS0 1PS1 1PS3 1PS5 1PS6 1PS7 1PS8 1PS9 1PSA 1PSC 1PSD 1PSH 1PSI 1PSJ 1PSK 1PSN 1PSO 1PSP 1PSQ 1PSR 1PSS 1PST 1PSU 1PSW 1PSZ 1PT0 1PT1 1PT2 1PT5 1PT6 1PT7 1PT8 1PT9 1PTA 1PTD 1PTF 1PTG 1PTH 1PTJ 1PTK 1PTM 1PTO 1PTQ 1PTR 1PTS 1PTT 1PTU 1PTV 1PTW 1PTX 1PTY 1PTZ 1PU0 1PU2 1PU4 1PU5 1PU6 1PU7 1PU8 1PU9 1PUA 1PUB 1PUC 1PUD 1PUG 1PUI 1PUJ 1PUM 1PUO 1PUU 1PV1 1PV2 1PV5 1PV6 1PV7 1PV8 1PV9 1PVA 1PVB 1PVC 1PVD 1PVF 1PVG 1PVH 1PVJ 1PVL 1PVM 1PVN 1PVS 1PVT 1PVU 1PVV 1PVW 1PVX 1PVY 1PW1 1PW2 1PW3 1PW4 1PW5 1PW6 1PW7 1PW8 1PW9 1PWA 1PWB 1PWC 1PWD 1PWE 1PWG 1PWH 1PWL 1PWM 1PWO 1PWP 1PWQ 1PWT 1PWU 1PWV 1PWW 1PWX 1PWY 1PWZ 1PX0 1PX2 1PX3 1PX4 1PX5 1PX6 1PX7 1PX8 1PXA 1PXB 1PXC 1PXD 1PXG 1PXH 1PXI 1PXJ 1PXK 1PXL 1PXM 1PXN 1PXO 1PXP 1PXR 1PXS 1PXT 1PXU 1PXV 1PXW 1PXX 1PXY 1PXZ 1PY0 1PY1 1PY2 1PY3 1PY4 1PY5 1PY6 1PY9 1PYA 1PYB 1PYD 1PYE 1PYF 1PYG 1PYH 1PYK 1PYL 1PYM 1PYN 1PYO 1PYP 1PYQ 1PYS 1PYT 1PYU 1PYW 1PYX 1PYY 1PYZ 1PZ0 1PZ1 1PZ2 1PZ3 1PZ4 1PZ5 1PZ7 1PZ8 1PZ9 1PZA 1PZB 1PZC 1PZD 1PZE 1PZF 1PZG 1PZH 1PZI 1PZJ 1PZK 1PZL 1PZM 1PZN 1PZO 1PZP 1PZS 1PZT 1PZV 1PZW 1PZX 1PZY 1PZZ 1Q03 1Q04 1Q05 1Q06 1Q07 1Q08 1Q09 1Q0A 1Q0B 1Q0C 1Q0D 1Q0E 1Q0F 1Q0G 1Q0H 1Q0K 1Q0L 1Q0M 1Q0N 1Q0O 1Q0P 1Q0Q 1Q0R 1Q0S 1Q0U 1Q0X 1Q0Y 1Q0Z 1Q11 1Q12 1Q13 1Q14 1Q15 1Q16 1Q17 1Q18 1Q19 1Q1A 1Q1B 1Q1C 1Q1E 1Q1F 1Q1G 1Q1H 1Q1J 1Q1K 1Q1L 1Q1M 1Q1N 1Q1P 1Q1Q 1Q1R 1Q1S 1Q1T 1Q1U 1Q1W 1Q1Y 1Q1Z 1Q20 1Q21 1Q22 1Q23 1Q24 1Q25 1Q2B 1Q2C 1Q2D 1Q2E 1Q2H 1Q2L 1Q2O 1Q2P 1Q2Q 1Q2U 1Q2V 1Q2W 1Q2X 1Q2Y 1Q31 1Q32 1Q33 1Q34 1Q35 1Q36 1Q39 1Q3A 1Q3B 1Q3C 1Q3D 1Q3E 1Q3G 1Q3H 1Q3I 1Q3K 1Q3L 1Q3N 1Q3O 1Q3P 1Q3Q 1Q3R 1Q3S 1Q3W 1Q3X 1Q40 1Q41 1Q42 1Q43 1Q44 1Q45 1Q46 1Q47 1Q4A 1Q4B 1Q4C 1Q4D 1Q4E 1Q4G 1Q4J 1Q4K 1Q4L 1Q4N 1Q4O 1Q4Q 1Q4R 1Q4S 1Q4T 1Q4U 1Q4V 1Q4W 1Q4X 1Q50 1Q51 1Q52 1Q54 1Q57 1Q5D 1Q5E 1Q5H 1Q5I 1Q5J 1Q5K 1Q5M 1Q5N 1Q5O 1Q5P 1Q5Q 1Q5R 1Q5T 1Q5U 1Q5V 1Q5X 1Q5Y 1Q5Z 1Q61 1Q62 1Q63 1Q65 1Q66 1Q67 1Q6C 1Q6D 1Q6E 1Q6F 1Q6G 1Q6H 1Q6I 1Q6J 1Q6K 1Q6L 1Q6M 1Q6N 1Q6O 1Q6P 1Q6Q 1Q6R 1Q6S 1Q6T 1Q6U 1Q6V 1Q6W 1Q6X 1Q6Y 1Q6Z 1Q72 1Q73 1Q74 1Q77 1Q78 1Q79 1Q7A 1Q7B 1Q7C 1Q7D 1Q7E 1Q7F 1Q7G 1Q7H 1Q7L 1Q7M 1Q7Q 1Q7R 1Q7S 1Q7T 1Q7Z 1Q83 1Q84 1Q85 1Q87 1Q88 1Q89 1Q8A 1Q8B 1Q8C 1Q8D 1Q8F 1Q8H 1Q8I 1Q8J 1Q8M 1Q8O 1Q8P 1Q8Q 1Q8R 1Q8S 1Q8T 1Q8U 1Q8V 1Q8W 1Q8Y 1Q8Z 1Q90 1Q91 1Q92 1Q94 1Q95 1Q97 1Q98 1Q99 1Q9B 1Q9C 1Q9D 1Q9E 1Q9H 1Q9I 1Q9J 1Q9K 1Q9L 1Q9M 1Q9O 1Q9S 1Q9U 1Q9W 1QA0 1QA1 1QA2 1QA3 1QA7 1QA9 1QAB 1QAC 1QAD 1QAE 1QAF 1QAG 1QAH 1QAK 1QAL 1QAM 1QAN 1QAO 1QAP 1QAQ 1QAS 1QAT 1QAU 1QAV 1QAW 1QAX 1QAY 1QAZ 1QB0 1QB1 1QB2 1QB3 1QB4 1QB5 1QB6 1QB7 1QB8 1QB9 1QBA 1QBB 1QBE 1QBG 1QBI 1QBK 1QBL 1QBM 1QBN 1QBO 1QBQ 1QBR 1QBS 1QBT 1QBU 1QBV 1QBZ 1QC5 1QC6 1QC7 1QC9 1QCA 1QCB 1QCC 1QCD 1QCF 1QCG 1QCI 1QCJ 1QCN 1QCO 1QCP 1QCQ 1QCR 1QCS 1QCW 1QCX 1QCY 1QCZ 1QD0 1QD1 1QD2 1QD5 1QD6 1QD8 1QD9 1QDB 1QDC 1QDD 1QDE 1QDL 1QDM 1QDN 1QDO 1QDQ 1QDR 1QDS 1QDT 1QDU 1QDV 1QDW 1QE0 1QE1 1QE3 1QE5 1QE6 1QEW 1QEX 1QEZ 1QF0 1QF1 1QF2 1QF3 1QF4 1QF5 1QF7 1QF8 1QF9 1QFC 1QFE 1QFF 1QFG 1QFH 1QFI 1QFJ 1QFK 1QFL 1QFM 1QFO 1QFP 1QFS 1QFT 1QFU 1QFV 1QFW 1QFX 1QFY 1QFZ 1QG0 1QG2 1QG3 1QG4 1QG5 1QG6 1QG7 1QG8 1QGA 1QGD 1QGE 1QGF 1QGH 1QGI 1QGJ 1QGK 1QGL 1QGN 1QGO 1QGQ 1QGR 1QGS 1QGT 1QGU 1QGV 1QGW 1QGX 1QGY 1QGZ 1QH0 1QH1 1QH3 1QH4 1QH5 1QH6 1QH7 1QH8 1QH9 1QHA 1QHB 1QHC 1QHD 1QHE 1QHF 1QHG 1QHH 1QHI 1QHJ 1QHL 1QHM 1QHN 1QHO 1QHP 1QHQ 1QHR 1QHS 1QHT 1QHU 1QHV 1QHW 1QHX 1QHY 1QHZ 1QI0 1QI1 1QI2 1QI3 1QI4 1QI5 1QI6 1QI7 1QI8 1QI9 1QIA 1QIB 1QIC 1QID 1QIE 1QIF 1QIG 1QIH 1QII 1QIJ 1QIK 1QIL 1QIM 1QIN 1QIO 1QIP 1QIQ 1QIR 1QIS 1QIT 1QIU 1QIV 1QIW 1QIX 1QIY 1QIZ 1QJ0 1QJ1 1QJ3 1QJ4 1QJ5 1QJ6 1QJ7 1QJ8 1QJ9 1QJA 1QJB 1QJC 1QJD 1QJE 1QJF 1QJG 1QJH 1QJI 1QJJ 1QJM 1QJP 1QJQ 1QJS 1QJU 1QJV 1QJW 1QJX 1QJY 1QJZ 1QK0 1QK1 1QK2 1QK3 1QK4 1QK5 1QK8 1QKA 1QKB 1QKC 1QKD 1QKE 1QKI 1QKJ 1QKK 1QKM 1QKN 1QKO 1QKP 1QKQ 1QKR 1QKS 1QKT 1QKU 1QKW 1QKX 1QKZ 1QL0 1QL3 1QL4 1QL6 1QL7 1QL8 1QL9 1QLB 1QLE 1QLF 1QLG 1QLH 1QLJ 1QLL 1QLM 1QLP 1QLQ 1QLR 1QLS 1QLT 1QLU 1QLV 1QLW 1QM4 1QM5 1QM6 1QM7 1QM8 1QMA 1QMB 1QMD 1QME 1QMF 1QMG 1QMH 1QMI 1QMJ 1QML 1QMN 1QMO 1QMP 1QMQ 1QMR 1QMT 1QMU 1QMV 1QMY 1QMZ 1QN2 1QNF 1QNG 1QNH 1QNI 1QNJ 1QNL 1QNM 1QNN 1QNO 1QNP 1QNQ 1QNR 1QNS 1QNT 1QNU 1QNV 1QNW 1QNX 1QNY 1QO0 1QO1 1QO2 1QO3 1QO4 1QO5 1QO7 1QO8 1QO9 1QOA 1QOB 1QOF 1QOG 1QOH 1QOI 1QOJ 1QOK 1QOL 1QOM 1QON 1QOO 1QOP 1QOQ 1QOR 1QOS 1QOT 1QOU 1QOV 1QOW 1QOX 1QOY 1QOZ 1QP1 1QP8 1QPA 1QPB 1QPC 1QPD 1QPE 1QPF 1QPG 1QPJ 1QPK 1QPL 1QPN 1QPO 1QPP 1QPQ 1QPR 1QPV 1QPW 1QPX 1QQ0 1QQ1 1QQ2 1QQ4 1QQ5 1QQ6 1QQ7 1QQ9 1QQC 1QQD 1QQE 1QQF 1QQG 1QQH 1QQJ 1QQK 1QQL 1QQM 1QQN 1QQO 1QQP 1QQQ 1QQR 1QQS 1QQT 1QQU 1QQW 1QQY 1QR0 1QR1 1QR2 1QR3 1QR4 1QR6 1QR7 1QR8 1QR9 1QRA 1QRB 1QRC 1QRD 1QRE 1QRF 1QRG 1QRK 1QRL 1QRM 1QRN 1QRP 1QRQ 1QRR 1QRW 1QRX 1QRZ 1QS0 1QS1 1QS2 1QS4 1QS5 1QS7 1QS8 1QS9 1QSA 1QSB 1QSC 1QSD 1QSE 1QSF 1QSG 1QSH 1QSI 1QSJ 1QSM 1QSN 1QSO 1QSP 1QSQ 1QSR 1QST 1QSU 1QSW 1QT1 1QT3 1QT4 1QT5 1QT6 1QT7 1QT8 1QT9 1QTB 1QTC 1QTD 1QTE 1QTF 1QTH 1QTI 1QTJ 1QTK 1QTN 1QTO 1QTP 1QTR 1QTS 1QTV 1QTW 1QTX 1QTY 1QTZ 1QU0 1QU1 1QU4 1QU7 1QU9 1QUA 1QUB 1QUD 1QUE 1QUF 1QUG 1QUH 1QUI 1QUJ 1QUK 1QUL 1QUN 1QUO 1QUP 1QUQ 1QUR 1QUS 1QUT 1QUU 1QUV 1QV0 1QV1 1QV6 1QV7 1QV9 1QVA 1QVB 1QVC 1QVE 1QVI 1QVJ 1QVN 1QVO 1QVR 1QVS 1QVT 1QVU 1QVV 1QVW 1QVY 1QVZ 1QW0 1QW2 1QW4 1QW5 1QW6 1QW7 1QW8 1QW9 1QWC 1QWD 1QWG 1QWH 1QWI 1QWJ 1QWK 1QWL 1QWM 1QWN 1QWO 1QWR 1QWS 1QWT 1QWU 1QWX 1QWY 1QWZ 1QX1 1QX2 1QX3 1QX4 1QX5 1QX6 1QX7 1QX8 1QXA 1QXD 1QXE 1QXH 1QXJ 1QXK 1QXL 1QXM 1QXO 1QXP 1QXR 1QXS 1QXT 1QXW 1QXX 1QXY 1QXZ 1QY0 1QY1 1QY2 1QY3 1QY4 1QY5 1QY6 1QY7 1QY8 1QY9 1QYA 1QYB 1QYC 1QYD 1QYE 1QYF 1QYG 1QYI 1QYM 1QYN 1QYO 1QYQ 1QYR 1QYS 1QYU 1QYV 1QYW 1QYX 1QYY 1QYZ 1QZ0 1QZ1 1QZ2 1QZ3 1QZ4 1QZ5 1QZ6 1QZ7 1QZ8 1QZ9 1QZF 1QZM 1QZN 1QZQ 1QZR 1QZT 1QZU 1QZV 1QZX 1QZY 1QZZ 1R00 1R03 1R08 1R09 1R0B 1R0C 1R0D 1R0E 1R0F 1R0G 1R0H 1R0I 1R0J 1R0K 1R0L 1R0M 1R0P 1R0Q 1R0R 1R0S 1R0U 1R0V 1R0W 1R0X 1R0Y 1R0Z 1R10 1R11 1R12 1R13 1R14 1R15 1R16 1R17 1R18 1R19 1R1A 1R1C 1R1D 1R1G 1R1H 1R1I 1R1J 1R1K 1R1L 1R1M 1R1N 1R1O 1R1P 1R1Q 1R1R 1R1S 1R1T 1R1U 1R1V 1R1W 1R1X 1R1Y 1R1Z 1R20 1R22 1R23 1R24 1R26 1R27 1R28 1R29 1R2B 1R2C 1R2D 1R2E 1R2F 1R2G 1R2H 1R2I 1R2J 1R2K 1R2M 1R2Q 1R2R 1R2S 1R2T 1R30 1R31 1R33 1R34 1R35 1R37 1R38 1R39 1R3C 1R3D 1R3F 1R3H 1R3I 1R3J 1R3K 1R3L 1R3M 1R3N 1R3Q 1R3R 1R3S 1R3T 1R3U 1R3V 1R3W 1R3Y 1R42 1R43 1R44 1R45 1R46 1R47 1R4A 1R4B 1R4C 1R4F 1R4L 1R4M 1R4N 1R4P 1R4Q 1R4S 1R4U 1R4V 1R4W 1R4X 1R4Z 1R50 1R51 1R52 1R53 1R54 1R55 1R56 1R58 1R59 1R5A 1R5B 1R5C 1R5D 1R5G 1R5H 1R5I 1R5J 1R5K 1R5L 1R5M 1R5N 1R5O 1R5P 1R5Q 1R5T 1R5U 1R5V 1R5W 1R5X 1R5Y 1R5Z 1R61 1R62 1R64 1R65 1R66 1R67 1R69 1R6A 1R6B 1R6C 1R6D 1R6F 1R6G 1R6J 1R6K 1R6L 1R6M 1R6N 1R6O 1R6Q 1R6T 1R6U 1R6V 1R6W 1R6X 1R6Y 1R6Z 1R74 1R75 1R76 1R77 1R78 1R7A 1R7H 1R7I 1R7J 1R7L 1R7O 1R7R 1R7S 1R7T 1R7U 1R7V 1R7X 1R7Y 1R80 1R81 1R82 1R85 1R86 1R87 1R88 1R89 1R8A 1R8B 1R8C 1R8G 1R8H 1R8I 1R8J 1R8K 1R8L 1R8M 1R8N 1R8O 1R8Q 1R8S 1R8W 1R8X 1R8Y 1R94 1R95 1R9C 1R9D 1R9G 1R9H 1R9J 1R9L 1R9M 1R9N 1R9O 1R9Q 1R9W 1R9X 1R9Y 1R9Z 1RA0 1RA1 1RA2 1RA3 1RA4 1RA5 1RA6 1RA7 1RA8 1RA9 1RAA 1RAB 1RAC 1RAD 1RAE 1RAF 1RAG 1RAH 1RAI 1RAJ 1RAK 1RAL 1RAO 1RAP 1RAQ 1RAR 1RAS 1RAT 1RAV 1RAY 1RAZ 1RB0 1RB2 1RB3 1RB4 1RB5 1RB6 1RB7 1RB9 1RBA 1RBB 1RBC 1RBD 1RBE 1RBF 1RBG 1RBH 1RBI 1RBL 1RBM 1RBN 1RBO 1RBP 1RBQ 1RBR 1RBS 1RBT 1RBU 1RBV 1RBW 1RBX 1RBY 1RBZ 1RC0 1RC1 1RC2 1RC4 1RC5 1RC6 1RC9 1RCA 1RCB 1RCC 1RCD 1RCE 1RCF 1RCG 1RCI 1RCJ 1RCM 1RCO 1RCP 1RCQ 1RCT 1RCU 1RCV 1RCW 1RCX 1RCY 1RD3 1RD4 1RD5 1RD6 1RD7 1RD8 1RD9 1RDA 1RDB 1RDC 1RDD 1RDF 1RDG 1RDH 1RDI 1RDJ 1RDK 1RDL 1RDM 1RDN 1RDO 1RDP 1RDQ 1RDR 1RDS 1RDT 1RDV 1RDW 1RDX 1RDY 1RDZ 1RE0 1RE1 1RE2 1RE3 1RE4 1RE5 1RE7 1RE8 1RE9 1REA 1REC 1RED 1REE 1REF 1REG 1REI 1REJ 1REK 1REM 1REO 1REQ 1RER 1REU 1REV 1REW 1REX 1REY 1REZ 1RF0 1RF1 1RF2 1RF3 1RF4 1RF5 1RF6 1RF7 1RF9 1RFB 1RFD 1RFE 1RFG 1RFJ 1RFK 1RFN 1RFP 1RFQ 1RFS 1RFT 1RFU 1RFV 1RFX 1RFY 1RFZ 1RG0 1RG5 1RG7 1RG8 1RG9 1RGA 1RGB 1RGC 1RGE 1RGF 1RGG 1RGH 1RGI 1RGK 1RGL 1RGN 1RGP 1RGQ 1RGS 1RGV 1RGX 1RGY 1RGZ 1RH1 1RH2 1RH3 1RH4 1RH5 1RH7 1RH9 1RHA 1RHB 1RHC 1RHD 1RHF 1RHG 1RHH 1RHI 1RHJ 1RHK 1RHL 1RHM 1RHO 1RHP 1RHQ 1RHR 1RHS 1RHU 1RHY 1RHZ 1RI1 1RI2 1RI3 1RI4 1RI5 1RI6 1RI7 1RI8 1RIB 1RID 1RIE 1RIF 1RIH 1RII 1RIL 1RIN 1RIQ 1RIR 1RIS 1RIT 1RIU 1RIV 1RIW 1RIY 1RJ1 1RJ2 1RJ4 1RJ5 1RJ6 1RJ7 1RJ8 1RJ9 1RJB 1RJC 1RJD 1RJE 1RJF 1RJG 1RJK 1RJL 1RJM 1RJN 1RJO 1RJP 1RJQ 1RJR 1RJU 1RJW 1RJX 1RJY 1RJZ 1RK0 1RK1 1RK2 1RK3 1RK4 1RK5 1RK6 1RK8 1RKA 1RKB 1RKC 1RKD 1RKE 1RKG 1RKH 1RKI 1RKM 1RKP 1RKQ 1RKR 1RKS 1RKT 1RKU 1RKV 1RKW 1RKX 1RKY 1RL0 1RL2 1RL3 1RL4 1RL6 1RL8 1RL9 1RLA 1RLB 1RLC 1RLD 1RLH 1RLI 1RLJ 1RLK 1RLM 1RLO 1RLR 1RLS 1RLT 1RLU 1RLV 1RLW 1RLZ 1RM0 1RM3 1RM4 1RM5 1RM6 1RM8 1RM9 1RMD 1RMF 1RMG 1RMH 1RMM 1RMO 1RMP 1RMQ 1RMR 1RMS 1RMT 1RMU 1RMY 1RMZ 1RN1 1RN4 1RN7 1RN8 1RNC 1RND 1RNE 1RNF 1RNH 1RNI 1RNJ 1RNL 1RNM 1RNN 1RNO 1RNQ 1RNR 1RNT 1RNU 1RNV 1RNW 1RNX 1RNY 1RNZ 1RO0 1RO2 1RO5 1RO6 1RO7 1RO8 1RO9 1ROA 1ROB 1ROC 1ROM 1ROP 1ROR 1ROS 1ROV 1ROW 1ROZ 1RP0 1RP1 1RP3 1RP4 1RP5 1RP7 1RP8 1RP9 1RPA 1RPF 1RPG 1RPH 1RPI 1RPJ 1RPK 1RPL 1RPM 1RPN 1RPO 1RPQ 1RPS 1RPT 1RPW 1RPX 1RPY 1RQ0 1RQ1 1RQ2 1RQ3 1RQ4 1RQ5 1RQ7 1RQ9 1RQA 1RQB 1RQC 1RQD 1RQE 1RQF 1RQG 1RQH 1RQI 1RQJ 1RQK 1RQL 1RQN 1RQP 1RQQ 1RQR 1RQW 1RQX 1RR2 1RR6 1RR7 1RR9 1RRA 1RRE 1RRF 1RRG 1RRH 1RRI 1RRK 1RRL 1RRM 1RRO 1RRP 1RRV 1RRW 1RRX 1RRY 1RS0 1RS2 1RS4 1RS6 1RS7 1RS8 1RS9 1RSC 1RSD 1RSE 1RSG 1RSI 1RSM 1RSN 1RSR 1RSS 1RST 1RSU 1RSV 1RSY 1RSZ 1RT1 1RT2 1RT3 1RT4 1RT5 1RT6 1RT7 1RT8 1RT9 1RTB 1RTC 1RTE 1RTF 1RTG 1RTH 1RTI 1RTJ 1RTK 1RTL 1RTM 1RTP 1RTQ 1RTR 1RTS 1RTT 1RTU 1RTV 1RTW 1RTX 1RTY 1RTZ 1RU0 1RU1 1RU2 1RU3 1RU4 1RU7 1RU9 1RUA 1RUC 1RUD 1RUE 1RUF 1RUG 1RUH 1RUI 1RUJ 1RUK 1RUL 1RUM 1RUP 1RUQ 1RUR 1RUS 1RUT 1RUV 1RUW 1RUY 1RUZ 1RV0 1RV1 1RV3 1RV4 1RV6 1RV7 1RV8 1RV9 1RVD 1RVE 1RVF 1RVG 1RVJ 1RVK 1RVT 1RVU 1RVV 1RVW 1RVX 1RVY 1RVZ 1RW0 1RW1 1RW4 1RW8 1RW9 1RWA 1RWB 1RWC 1RWE 1RWF 1RWG 1RWH 1RWI 1RWJ 1RWK 1RWL 1RWM 1RWN 1RWO 1RWP 1RWQ 1RWR 1RWT 1RWV 1RWW 1RWX 1RWY 1RWZ 1RX0 1RX1 1RX2 1RX3 1RX4 1RX5 1RX6 1RX7 1RX8 1RX9 1RXC 1RXD 1RXE 1RXF 1RXG 1RXH 1RXI 1RXJ 1RXK 1RXM 1RXO 1RXP 1RXQ 1RXS 1RXT 1RXU 1RXX 1RXY 1RXZ 1RY0 1RY2 1RY5 1RY6 1RY7 1RY8 1RY9 1RYA 1RYB 1RYC 1RYD 1RYE 1RYF 1RYH 1RYI 1RYL 1RYM 1RYN 1RYO 1RYP 1RYQ 1RYT 1RYW 1RYX 1RYY 1RYZ 1RZ0 1RZ1 1RZ2 1RZ3 1RZ4 1RZ5 1RZ6 1RZ7 1RZ8 1RZA 1RZB 1RZC 1RZD 1RZE 1RZF 1RZG 1RZH 1RZI 1RZJ 1RZK 1RZL 1RZM 1RZN 1RZO 1RZP 1RZQ 1RZU 1RZV 1RZX 1RZY 1RZZ 1S00 1S01 1S02 1S06 1S07 1S08 1S09 1S0A 1S0B 1S0C 1S0D 1S0E 1S0F 1S0G 1S0H 1S0I 1S0J 1S0L 1S0P 1S0Q 1S0R 1S0U 1S0W 1S0X 1S0Y 1S0Z 1S12 1S13 1S14 1S16 1S17 1S18 1S19 1S1A 1S1C 1S1D 1S1E 1S1F 1S1G 1S1J 1S1M 1S1P 1S1Q 1S1R 1S1S 1S1T 1S1U 1S1V 1S1W 1S1X 1S1Y 1S1Z 1S20 1S21 1S22 1S26 1S28 1S29 1S2A 1S2B 1S2C 1S2D 1S2E 1S2G 1S2I 1S2J 1S2K 1S2L 1S2M 1S2N 1S2O 1S2P 1S2Q 1S2T 1S2U 1S2V 1S2W 1S2X 1S2Y 1S2Z 1S30 1S31 1S35 1S36 1S38 1S39 1S3B 1S3C 1S3D 1S3E 1S3F 1S3G 1S3H 1S3I 1S3J 1S3K 1S3L 1S3M 1S3N 1S3O 1S3P 1S3Q 1S3R 1S3S 1S3T 1S3U 1S3V 1S3W 1S3X 1S3Y 1S3Z 1S44 1S46 1S48 1S49 1S4B 1S4C 1S4D 1S4E 1S4F 1S4I 1S4K 1S4M 1S4N 1S4O 1S4P 1S4Q 1S4R 1S4S 1S4U 1S4V 1S4Y 1S50 1S51 1S52 1S53 1S54 1S55 1S56 1S57 1S58 1S59 1S5A 1S5B 1S5C 1S5D 1S5E 1S5F 1S5G 1S5H 1S5I 1S5J 1S5K 1S5L 1S5M 1S5N 1S5O 1S5P 1S5S 1S5T 1S5U 1S5V 1S5W 1S5X 1S5Y 1S5Z 1S60 1S61 1S63 1S64 1S66 1S67 1S68 1S69 1S6A 1S6B 1S6C 1S6F 1S6H 1S6P 1S6Q 1S6R 1S6V 1S6Y 1S6Z 1S70 1S73 1S78 1S7C 1S7D 1S7F 1S7G 1S7H 1S7I 1S7J 1S7K 1S7L 1S7M 1S7N 1S7O 1S7Q 1S7R 1S7S 1S7T 1S7U 1S7V 1S7W 1S7X 1S7Y 1S7Z 1S80 1S81 1S82 1S83 1S84 1S85 1S89 1S8A 1S8C 1S8D 1S8E 1S8F 1S8G 1S8H 1S8I 1S8J 1S8L 1S8N 1S8O 1S94 1S95 1S96 1S98 1S99 1S9A 1S9C 1S9D 1S9E 1S9G 1S9H 1S9I 1S9J 1S9P 1S9Q 1S9R 1S9T 1S9U 1S9V 1S9W 1S9X 1S9Y 1S9Z 1SA0 1SA1 1SA4 1SA5 1SAC 1SAR 1SAT 1SAU 1SAV 1SAW 1SAY 1SAZ 1SB1 1SB2 1SB3 1SB7 1SB8 1SB9 1SBB 1SBC 1SBD 1SBE 1SBF 1SBG 1SBH 1SBI 1SBK 1SBM 1SBN 1SBP 1SBQ 1SBR 1SBS 1SBT 1SBW 1SBX 1SBY 1SBZ 1SC0 1SC1 1SC3 1SC4 1SC5 1SC6 1SC8 1SC9 1SCA 1SCB 1SCD 1SCE 1SCF 1SCH 1SCI 1SCJ 1SCK 1SCM 1SCN 1SCQ 1SCR 1SCS 1SCU 1SCW 1SCZ 1SD0 1SD1 1SD2 1SD3 1SD4 1SD5 1SD6 1SD7 1SD8 1SD9 1SDA 1SDB 1SDD 1SDE 1SDI 1SDJ 1SDK 1SDL 1SDM 1SDN 1SDO 1SDQ 1SDT 1SDU 1SDV 1SDW 1SDX 1SDY 1SDZ 1SE0 1SE2 1SE3 1SE4 1SE6 1SE8 1SEB 1SED 1SEF 1SEG 1SEH 1SEI 1SEJ 1SEK 1SEL 1SEM 1SEN 1SEP 1SEQ 1SES 1SET 1SEV 1SEZ 1SF2 1SF3 1SF5 1SF8 1SF9 1SFC 1SFD 1SFE 1SFF 1SFH 1SFI 1SFJ 1SFK 1SFL 1SFN 1SFP 1SFQ 1SFR 1SFS 1SFT 1SFX 1SFY 1SG0 1SG1 1SG2 1SG3 1SG4 1SG6 1SG8 1SG9 1SGC 1SGD 1SGE 1SGF 1SGH 1SGI 1SGJ 1SGK 1SGL 1SGM 1SGN 1SGP 1SGQ 1SGR 1SGT 1SGU 1SGV 1SGW 1SGY 1SGZ 1SH0 1SH2 1SH3 1SH5 1SH6 1SH7 1SH8 1SH9 1SHA 1SHB 1SHD 1SHF 1SHG 1SHH 1SHJ 1SHK 1SHL 1SHM 1SHN 1SHO 1SHQ 1SHR 1SHS 1SHT 1SHU 1SHV 1SHW 1SHX 1SHY 1SHZ 1SI0 1SI1 1SI4 1SI5 1SI6 1SI7 1SI8 1SI9 1SIB 1SID 1SIE 1SIF 1SIG 1SIH 1SII 1SIJ 1SIO 1SIP 1SIQ 1SIR 1SIU 1SIV 1SIW 1SIX 1SIZ 1SJ0 1SJ1 1SJ2 1SJ5 1SJ7 1SJ8 1SJ9 1SJA 1SJB 1SJC 1SJD 1SJE 1SJH 1SJI 1SJM 1SJN 1SJP 1SJS 1SJV 1SJW 1SJX 1SJY 1SJZ 1SK0 1SK1 1SK2 1SK3 1SK4 1SK6 1SK7 1SK8 1SK9 1SKA 1SKB 1SKF 1SKG 1SKJ 1SKO 1SKQ 1SKU 1SKV 1SKX 1SKY 1SKZ 1SL3 1SL4 1SL5 1SL6 1SL7 1SL8 1SL9 1SLA 1SLB 1SLC 1SLD 1SLE 1SLF 1SLG 1SLH 1SLI 1SLL 1SLM 1SLN 1SLQ 1SLT 1SLU 1SLV 1SLW 1SLX 1SLY 1SM2 1SM3 1SM4 1SM8 1SM9 1SMA 1SMB 1SMC 1SMD 1SME 1SMF 1SMH 1SMI 1SMJ 1SMK 1SML 1SMM 1SMN 1SMO 1SMP 1SMQ 1SMR 1SMS 1SMT 1SMU 1SMV 1SMW 1SMX 1SMY 1SN0 1SN1 1SN2 1SN4 1SN5 1SN7 1SN8 1SN9 1SNA 1SNB 1SNC 1SND 1SNE 1SNF 1SNG 1SNK 1SNM 1SNN 1SNO 1SNP 1SNQ 1SNR 1SNT 1SNU 1SNX 1SNY 1SNZ 1SO0 1SO2 1SO3 1SO4 1SO5 1SO6 1SO7 1SO8 1SOA 1SOF 1SOG 1SOI 1SOJ 1SOK 1SOM 1SON 1SOO 1SOQ 1SOS 1SOT 1SOV 1SOW 1SOX 1SOZ 1SP3 1SP4 1SP5 1SP8 1SP9 1SPA 1SPB 1SPD 1SPE 1SPG 1SPH 1SPI 1SPJ 1SPP 1SPQ 1SPR 1SPS 1SPU 1SPV 1SPX 1SQ0 1SQ1 1SQ2 1SQ3 1SQ4 1SQ5 1SQ6 1SQ7 1SQ9 1SQA 1SQB 1SQC 1SQD 1SQE 1SQF 1SQG 1SQH 1SQI 1SQJ 1SQK 1SQL 1SQM 1SQN 1SQO 1SQP 1SQQ 1SQS 1SQT 1SQU 1SQV 1SQW 1SQX 1SQY 1SQZ 1SR0 1SR4 1SR5 1SR6 1SR7 1SR8 1SR9 1SRA 1SRD 1SRE 1SRF 1SRG 1SRH 1SRI 1SRJ 1SRN 1SRP 1SRQ 1SRR 1SRU 1SRV 1SRX 1SRY 1SS4 1SS8 1SS9 1SSA 1SSB 1SSC 1SSD 1SSG 1SSH 1SSM 1SSQ 1SST 1SSW 1SSX 1SSY 1ST0 1ST2 1ST3 1ST4 1ST6 1ST8 1ST9 1STA 1STB 1STC 1STD 1STE 1STF 1STG 1STH 1STM 1STN 1STO 1STP 1STQ 1STR 1STS 1STY 1STZ 1SU0 1SU1 1SU2 1SU3 1SU4 1SU5 1SU6 1SU7 1SU8 1SU9 1SUA 1SUB 1SUC 1SUD 1SUE 1SUF 1SUG 1SUI 1SUJ 1SUL 1SUM 1SUO 1SUP 1SUQ 1SUR 1SUS 1SUU 1SUW 1SUX 1SV0 1SV2 1SV3 1SV4 1SV5 1SV6 1SV9 1SVA 1SVB 1SVD 1SVE 1SVF 1SVG 1SVH 1SVI 1SVK 1SVL 1SVM 1SVN 1SVO 1SVP 1SVS 1SVT 1SVU 1SVV 1SVW 1SVX 1SVY 1SVZ 1SW0 1SW1 1SW2 1SW3 1SW4 1SW5 1SW6 1SW7 1SWA 1SWB 1SWC 1SWD 1SWE 1SWF 1SWG 1SWH 1SWI 1SWJ 1SWK 1SWL 1SWM 1SWN 1SWO 1SWP 1SWQ 1SWR 1SWS 1SWT 1SWU 1SWV 1SWW 1SWX 1SWY 1SWZ 1SX2 1SX3 1SX4 1SX6 1SX7 1SXA 1SXB 1SXC 1SXG 1SXH 1SXI 1SXJ 1SXK 1SXN 1SXR 1SXS 1SXT 1SXU 1SXV 1SXW 1SXX 1SXY 1SXZ 1SY0 1SY1 1SY2 1SY3 1SY6 1SY7 1SYB 1SYC 1SYD 1SYE 1SYF 1SYG 1SYH 1SYI 1SYK 1SYL 1SYN 1SYO 1SYQ 1SYR 1SYS 1SYT 1SYV 1SYX 1SYY 1SZ0 1SZ2 1SZ3 1SZ6 1SZ7 1SZ8 1SZ9 1SZA 1SZB 1SZC 1SZD 1SZE 1SZF 1SZG 1SZH 1SZI 1SZJ 1SZK 1SZM 1SZN 1SZO 1SZP 1SZQ 1SZR 1SZS 1SZT 1SZU 1SZW 1SZX 1SZZ 1T00 1T01 1T02 1T04 1T06 1T07 1T08 1T09 1T0A 1T0B 1T0F 1T0H 1T0I 1T0J 1T0L 1T0M 1T0N 1T0O 1T0P 1T0Q 1T0R 1T0S 1T0T 1T0U 1T0Z 1T10 1T11 1T13 1T14 1T15 1T16 1T18 1T19 1T1A 1T1B 1T1C 1T1D 1T1E 1T1F 1T1G 1T1I 1T1J 1T1L 1T1N 1T1R 1T1S 1T1U 1T1V 1T1W 1T1X 1T1Y 1T1Z 1T20 1T21 1T22 1T24 1T25 1T26 1T27 1T29 1T2A 1T2B 1T2C 1T2D 1T2E 1T2F 1T2H 1T2I 1T2J 1T2L 1T2N 1T2O 1T2P 1T2Q 1T2U 1T2V 1T2W 1T2X 1T31 1T32 1T33 1T34 1T35 1T36 1T37 1T3A 1T3B 1T3C 1T3D 1T3E 1T3F 1T3G 1T3H 1T3I 1T3J 1T3L 1T3M 1T3P 1T3Q 1T3R 1T3S 1T3T 1T3U 1T3W 1T3X 1T3Y 1T3Z 1T40 1T41 1T43 1T44 1T45 1T46 1T47 1T48 1T49 1T4A 1T4B 1T4C 1T4D 1T4E 1T4F 1T4G 1T4J 1T4K 1T4M 1T4O 1T4P 1T4Q 1T4R 1T4S 1T4T 1T4U 1T4V 1T4W 1T56 1T57 1T5A 1T5B 1T5C 1T5D 1T5E 1T5F 1T5G 1T5H 1T5I 1T5J 1T5K 1T5L 1T5O 1T5P 1T5R 1T5S 1T5T 1T5W 1T5X 1T5Y 1T5Z 1T60 1T61 1T62 1T63 1T64 1T65 1T66 1T67 1T68 1T69 1T6A 1T6B 1T6C 1T6D 1T6E 1T6F 1T6G 1T6H 1T6I 1T6J 1T6K 1T6L 1T6M 1T6N 1T6O 1T6P 1T6Q 1T6S 1T6T 1T6U 1T6V 1T6X 1T6Y 1T6Z 1T70 1T71 1T72 1T73 1T74 1T75 1T76 1T77 1T79 1T7A 1T7B 1T7C 1T7D 1T7E 1T7F 1T7H 1T7I 1T7J 1T7K 1T7L 1T7M 1T7N 1T7O 1T7Q 1T7R 1T7S 1T7T 1T7V 1T7W 1T7X 1T7Y 1T7Z 1T80 1T82 1T83 1T85 1T86 1T87 1T88 1T89 1T8A 1T8B 1T8F 1T8G 1T8H 1T8K 1T8L 1T8M 1T8N 1T8O 1T8P 1T8Q 1T8R 1T8S 1T8T 1T8U 1T8W 1T8X 1T8Y 1T8Z 1T90 1T91 1T92 1T93 1T94 1T95 1T96 1T97 1T98 1T99 1T9A 1T9B 1T9C 1T9D 1T9F 1T9G 1T9H 1T9K 1T9M 1T9N 1T9O 1T9P 1T9Q 1T9R 1T9S 1T9T 1T9U 1T9V 1T9W 1T9X 1T9Y 1T9Z 1TA0 1TA1 1TA2 1TA3 1TA4 1TA6 1TA8 1TA9 1TAB 1TAD 1TAE 1TAF 1TAG 1TAH 1TAL 1TAQ 1TAR 1TAS 1TAT 1TAW 1TAY 1TAZ 1TB0 1TB3 1TB4 1TB5 1TB6 1TB7 1TBB 1TBE 1TBF 1TBG 1TBH 1TBJ 1TBL 1TBP 1TBQ 1TBR 1TBT 1TBU 1TBW 1TBX 1TBY 1TBZ 1TC0 1TC1 1TC2 1TC5 1TC6 1TC8 1TCA 1TCB 1TCC 1TCD 1TCF 1TCM 1TCO 1TCR 1TCS 1TCU 1TCV 1TCW 1TCX 1TCY 1TCZ 1TD0 1TD1 1TD2 1TD3 1TD4 1TD5 1TD6 1TD7 1TD9 1TDA 1TDB 1TDC 1TDE 1TDF 1TDG 1TDH 1TDI 1TDJ 1TDK 1TDL 1TDN 1TDO 1TDQ 1TDR 1TDT 1TDU 1TDV 1TDW 1TDY 1TE0 1TE1 1TE2 1TE3 1TE5 1TE6 1TEC 1TED 1TEE 1TEF 1TEG 1TEH 1TEI 1TEJ 1TEL 1TEM 1TEN 1TEQ 1TES 1TET 1TEU 1TEV 1TEW 1TEX 1TF0 1TF1 1TF2 1TF4 1TF5 1TF7 1TF8 1TF9 1TFA 1TFC 1TFD 1TFE 1TFF 1TFG 1TFH 1TFJ 1TFK 1TFM 1TFO 1TFP 1TFR 1TFU 1TFV 1TFX 1TFZ 1TG0 1TG1 1TG2 1TG3 1TG4 1TG5 1TG6 1TG7 1TG8 1TG9 1TGB 1TGC 1TGG 1TGJ 1TGK 1TGL 1TGM 1TGN 1TGO 1TGR 1TGS 1TGT 1TGU 1TGV 1TGX 1TGY 1TGZ 1TH0 1TH1 1TH2 1TH3 1TH4 1TH6 1TH7 1TH8 1TH9 1THA 1THB 1THC 1THE 1THF 1THG 1THI 1THJ 1THK 1THL 1THM 1THN 1THO 1THP 1THQ 1THR 1THS 1THT 1THU 1THV 1THW 1THX 1THY 1THZ 1TI1 1TI7 1TI8 1TIA 1TIB 1TIC 1TID 1TIE 1TIF 1TIG 1TII 1TIJ 1TIK 1TIL 1TIM 1TIO 1TIP 1TIQ 1TIS 1TIW 1TIY 1TJ0 1TJ1 1TJ2 1TJ3 1TJ4 1TJ5 1TJ6 1TJ7 1TJ9 1TJB 1TJC 1TJD 1TJE 1TJF 1TJG 1TJH 1TJI 1TJJ 1TJK 1TJL 1TJM 1TJN 1TJO 1TJP 1TJR 1TJS 1TJT 1TJU 1TJV 1TJW 1TJX 1TJY 1TK1 1TK2 1TK3 1TK4 1TK6 1TK9 1TKA 1TKB 1TKC 1TKE 1TKF 1TKG 1TKH 1TKI 1TKJ 1TKK 1TKL 1TKO 1TKP 1TKR 1TKS 1TKT 1TKU 1TKX 1TKY 1TKZ 1TL1 1TL2 1TL3 1TL7 1TL9 1TLA 1TLB 1TLC 1TLD 1TLF 1TLG 1TLI 1TLJ 1TLK 1TLL 1TLM 1TLO 1TLP 1TLQ 1TLS 1TLT 1TLU 1TLV 1TLW 1TLX 1TLY 1TLZ 1TM0 1TM1 1TM2 1TM3 1TM4 1TM5 1TM7 1TMB 1TMC 1TME 1TMF 1TMG 1TMH 1TMI 1TMJ 1TMK 1TML 1TMM 1TMN 1TMO 1TMQ 1TMT 1TMU 1TMX 1TMY 1TN0 1TN3 1TN4 1TN5 1TN6 1TN7 1TN8 1TNB 1TND 1TNF 1TNG 1TNH 1TNI 1TNJ 1TNK 1TNL 1TNO 1TNR 1TNU 1TNV 1TNY 1TNZ 1TO0 1TO1 1TO2 1TO3 1TO4 1TO5 1TO6 1TO9 1TOA 1TOC 1TOE 1TOG 1TOH 1TOI 1TOJ 1TOK 1TOL 1TOM 1TON 1TOO 1TOP 1TOQ 1TOU 1TOV 1TOW 1TOX 1TP0 1TP2 1TP3 1TP5 1TP6 1TP7 1TP8 1TP9 1TPA 1TPB 1TPC 1TPD 1TPE 1TPF 1TPH 1TPK 1TPL 1TPO 1TPP 1TPS 1TPT 1TPU 1TPV 1TPW 1TPX 1TPY 1TPZ 1TQ0 1TQ2 1TQ3 1TQ4 1TQ5 1TQ6 1TQ7 1TQ8 1TQ9 1TQB 1TQC 1TQD 1TQF 1TQG 1TQH 1TQI 1TQJ 1TQL 1TQM 1TQN 1TQO 1TQP 1TQQ 1TQS 1TQT 1TQU 1TQV 1TQW 1TQX 1TQY 1TR0 1TR1 1TR2 1TR5 1TR7 1TR8 1TR9 1TRB 1TRD 1TRE 1TRG 1TRH 1TRI 1TRK 1TRM 1TRN 1TRP 1TRQ 1TRY 1TRZ 1TS0 1TS2 1TS3 1TS4 1TS5 1TS6 1TS7 1TS8 1TS9 1TSD 1TSF 1TSH 1TSI 1TSJ 1TSL 1TSM 1TSN 1TSP 1TSQ 1TSU 1TSV 1TSW 1TSX 1TSY 1TSZ 1TT0 1TT1 1TT2 1TT4 1TT5 1TT6 1TT7 1TT8 1TT9 1TTA 1TTB 1TTC 1TTH 1TTI 1TTJ 1TTM 1TTO 1TTP 1TTQ 1TTR 1TTW 1TTZ 1TU0 1TU1 1TU3 1TU4 1TU5 1TU6 1TU7 1TU8 1TU9 1TUA 1TUC 1TUD 1TUE 1TUF 1TUG 1TUH 1TUI 1TUK 1TUL 1TUO 1TUU 1TUV 1TUW 1TUX 1TUY 1TV2 1TV3 1TV4 1TV5 1TV6 1TV7 1TV8 1TVB 1TVD 1TVE 1TVF 1TVG 1TVH 1TVL 1TVN 1TVO 1TVP 1TVQ 1TVR 1TVU 1TVV 1TVW 1TVX 1TVY 1TVZ 1TW0 1TW1 1TW2 1TW3 1TW4 1TW5 1TW6 1TW7 1TW9 1TWA 1TWB 1TWC 1TWD 1TWE 1TWF 1TWG 1TWH 1TWI 1TWJ 1TWL 1TWM 1TWN 1TWQ 1TWR 1TWS 1TWU 1TWW 1TWX 1TWY 1TWZ 1TX0 1TX2 1TX4 1TX6 1TX7 1TX8 1TX9 1TXC 1TXD 1TXF 1TXG 1TXI 1TXJ 1TXK 1TXL 1TXN 1TXO 1TXQ 1TXR 1TXT 1TXU 1TXX 1TXY 1TXZ 1TY0 1TY2 1TY4 1TY8 1TY9 1TYA 1TYB 1TYC 1TYD 1TYE 1TYF 1TYG 1TYH 1TYJ 1TYL 1TYM 1TYN 1TYO 1TYP 1TYQ 1TYR 1TYS 1TYT 1TYU 1TYV 1TYW 1TYX 1TYY 1TYZ 1TZ0 1TZ2 1TZ3 1TZ6 1TZ7 1TZ8 1TZ9 1TZA 1TZB 1TZC 1TZD 1TZE 1TZF 1TZG 1TZH 1TZI 1TZJ 1TZK 1TZL 1TZM 1TZN 1TZO 1TZP 1TZQ 1TZS 1TZT 1TZU 1TZV 1TZW 1TZX 1TZY 1TZZ 1U00 1U02 1U04 1U05 1U06 1U07 1U08 1U09 1U0A 1U0E 1U0F 1U0G 1U0H 1U0J 1U0K 1U0L 1U0M 1U0N 1U0O 1U0Q 1U0R 1U0S 1U0T 1U0U 1U0V 1U0W 1U0X 1U0Y 1U0Z 1U10 1U11 1U12 1U13 1U14 1U15 1U16 1U17 1U18 1U19 1U1B 1U1C 1U1D 1U1E 1U1F 1U1G 1U1H 1U1I 1U1J 1U1S 1U1T 1U1U 1U1V 1U1W 1U1X 1U1Z 1U20 1U21 1U22 1U24 1U25 1U26 1U27 1U28 1U29 1U2B 1U2C 1U2D 1U2E 1U2G 1U2H 1U2J 1U2K 1U2L 1U2M 1U2O 1U2P 1U2Q 1U2R 1U2S 1U2T 1U2V 1U2W 1U2X 1U2Y 1U2Z 1U30 1U31 1U32 1U33 1U36 1U3A 1U3C 1U3D 1U3F 1U3G 1U3H 1U3I 1U3J 1U3L 1U3P 1U3Q 1U3R 1U3S 1U3T 1U3U 1U3V 1U3W 1U3Y 1U3Z 1U40 1U41 1U42 1U43 1U46 1U4C 1U4D 1U4E 1U4F 1U4G 1U4H 1U4J 1U4L 1U4M 1U4N 1U4O 1U4P 1U4Q 1U4R 1U4S 1U53 1U54 1U55 1U56 1U58 1U59 1U5A 1U5B 1U5C 1U5D 1U5E 1U5F 1U5G 1U5H 1U5I 1U5J 1U5K 1U5O 1U5P 1U5Q 1U5R 1U5T 1U5U 1U5V 1U5W 1U5X 1U5Y 1U5Z 1U60 1U61 1U65 1U67 1U68 1U69 1U6A 1U6D 1U6E 1U6G 1U6H 1U6I 1U6J 1U6K 1U6L 1U6M 1U6Q 1U6R 1U6S 1U6T 1U6Z 1U70 1U71 1U72 1U73 1U74 1U75 1U76 1U77 1U79 1U7B 1U7C 1U7D 1U7F 1U7G 1U7H 1U7I 1U7K 1U7L 1U7N 1U7O 1U7P 1U7R 1U7S 1U7T 1U7U 1U7V 1U7W 1U7X 1U7Z 1U80 1U83 1U84 1U87 1U88 1U8A 1U8C 1U8E 1U8F 1U8G 1U8H 1U8I 1U8J 1U8K 1U8L 1U8M 1U8N 1U8O 1U8P 1U8Q 1U8S 1U8T 1U8U 1U8V 1U8W 1U8X 1U8Y 1U8Z 1U90 1U91 1U92 1U93 1U94 1U95 1U98 1U99 1U9A 1U9B 1U9C 1U9D 1U9E 1U9F 1U9G 1U9H 1U9I 1U9J 1U9K 1U9L 1U9M 1U9N 1U9O 1U9P 1U9Q 1U9R 1U9T 1U9U 1U9V 1U9W 1U9X 1U9Y 1U9Z 1UA2 1UA3 1UA4 1UA5 1UA6 1UA7 1UA8 1UAC 1UAD 1UAE 1UAG 1UAI 1UAJ 1UAK 1UAL 1UAM 1UAN 1UAQ 1UAR 1UAS 1UAT 1UAX 1UAY 1UAZ 1UB0 1UB2 1UB3 1UB4 1UB5 1UB6 1UB7 1UB9 1UBB 1UBC 1UBE 1UBF 1UBG 1UBH 1UBI 1UBJ 1UBK 1UBL 1UBM 1UBN 1UBO 1UBP 1UBQ 1UBR 1UBS 1UBT 1UBU 1UBV 1UBW 1UBX 1UBY 1UBZ 1UC0 1UC2 1UC3 1UC4 1UC5 1UC7 1UC8 1UC9 1UCA 1UCB 1UCC 1UCD 1UCF 1UCG 1UCH 1UCI 1UCJ 1UCK 1UCL 1UCN 1UCO 1UCQ 1UCR 1UCS 1UCT 1UCW 1UCX 1UCY 1UD0 1UD1 1UD2 1UD3 1UD4 1UD5 1UD6 1UD8 1UD9 1UDA 1UDB 1UDC 1UDD 1UDE 1UDG 1UDH 1UDI 1UDN 1UDO 1UDQ 1UDR 1UDS 1UDT 1UDU 1UDV 1UDW 1UDX 1UDY 1UDZ 1UE0 1UE1 1UE5 1UE6 1UE7 1UE8 1UEA 1UEB 1UEC 1UED 1UEF 1UEG 1UEH 1UEI 1UEJ 1UEK 1UER 1UES 1UET 1UEU 1UEV 1UEX 1UF2 1UF3 1UF4 1UF5 1UF7 1UF8 1UF9 1UFA 1UFB 1UFH 1UFI 1UFJ 1UFK 1UFL 1UFO 1UFP 1UFQ 1UFR 1UFU 1UFV 1UFY 1UG3 1UG4 1UG6 1UG9 1UGA 1UGB 1UGC 1UGD 1UGE 1UGF 1UGG 1UGH 1UGI 1UGM 1UGN 1UGP 1UGQ 1UGR 1UGS 1UGU 1UGW 1UGX 1UGY 1UH0 1UH1 1UH2 1UH3 1UH4 1UH5 1UH7 1UH8 1UH9 1UHA 1UHB 1UHD 1UHE 1UHG 1UHH 1UHI 1UHJ 1UHK 1UHL 1UHN 1UHO 1UHV 1UI0 1UI1 1UI5 1UI6 1UI7 1UI8 1UI9 1UIA 1UIB 1UIC 1UID 1UIE 1UIF 1UIG 1UIH 1UII 1UIJ 1UIK 1UIM 1UIN 1UIO 1UIP 1UIR 1UIS 1UIU 1UIV 1UIW 1UIX 1UIY 1UIZ 1UJ0 1UJ1 1UJ2 1UJ3 1UJ4 1UJ5 1UJ6 1UJ8 1UJB 1UJC 1UJJ 1UJK 1UJM 1UJN 1UJP 1UJQ 1UJW 1UJZ 1UK0 1UK1 1UK2 1UK3 1UK4 1UK6 1UK7 1UK8 1UK9 1UKA 1UKB 1UKC 1UKE 1UKF 1UKG 1UKH 1UKI 1UKJ 1UKK 1UKL 1UKM 1UKO 1UKP 1UKQ 1UKR 1UKS 1UKT 1UKU 1UKV 1UKW 1UKY 1UKZ 1UL1 1UL3 1UL9 1ULA 1ULB 1ULC 1ULD 1ULE 1ULF 1ULG 1ULH 1ULI 1ULJ 1ULK 1ULM 1ULN 1ULQ 1ULR 1ULS 1ULT 1ULU 1ULV 1ULW 1ULX 1ULY 1ULZ 1UM0 1UM2 1UM4 1UM5 1UM6 1UM8 1UM9 1UMA 1UMB 1UMC 1UMD 1UMF 1UMG 1UMH 1UMI 1UMJ 1UMK 1UML 1UMN 1UMO 1UMP 1UMR 1UMU 1UMV 1UMW 1UMX 1UMY 1UMZ 1UN0 1UN1 1UN2 1UN3 1UN4 1UN5 1UN8 1UN9 1UNA 1UNB 1UNE 1UNF 1UNG 1UNH 1UNK 1UNL 1UNN 1UNO 1UNP 1UNQ 1UNR 1UNS 1UNT 1UNU 1UNV 1UNW 1UNX 1UNY 1UNZ 1UO0 1UO1 1UO2 1UO3 1UO4 1UO5 1UO6 1UO9 1UOB 1UOC 1UOD 1UOE 1UOF 1UOG 1UOH 1UOJ 1UOK 1UOL 1UOM 1UOO 1UOP 1UOQ 1UOR 1UOS 1UOT 1UOU 1UOV 1UOW 1UOY 1UOZ 1UP0 1UP1 1UP2 1UP3 1UP4 1UP5 1UP6 1UP7 1UP8 1UP9 1UPA 1UPB 1UPC 1UPD 1UPF 1UPG 1UPI 1UPJ 1UPK 1UPL 1UPM 1UPP 1UPQ 1UPR 1UPS 1UPT 1UPU 1UPV 1UPW 1UPX 1UQ4 1UQ5 1UQR 1UQS 1UQT 1UQU 1UQW 1UQX 1UQY 1UQZ 1UR0 1UR1 1UR2 1UR3 1UR4 1UR5 1UR8 1UR9 1URA 1URB 1URC 1URD 1URG 1URH 1URI 1URJ 1URL 1URM 1URO 1URP 1URQ 1URR 1URS 1URT 1URU 1URV 1URW 1URX 1URY 1URZ 1US0 1US1 1US2 1US3 1US4 1US5 1US6 1US7 1US8 1USB 1USC 1USD 1USE 1USF 1USG 1USH 1USI 1USK 1USL 1USM 1USN 1USO 1USP 1USQ 1USR 1USU 1USV 1USW 1USX 1USY 1USZ 1UT0 1UT1 1UT2 1UT4 1UT5 1UT6 1UT7 1UT8 1UT9 1UTB 1UTC 1UTE 1UTG 1UTH 1UTI 1UTJ 1UTK 1UTL 1UTM 1UTN 1UTO 1UTP 1UTQ 1UTT 1UTU 1UTX 1UTY 1UTZ 1UU0 1UU1 1UU2 1UU3 1UU4 1UU5 1UU6 1UU7 1UU8 1UU9 1UUE 1UUF 1UUG 1UUH 1UUJ 1UUM 1UUN 1UUO 1UUP 1UUQ 1UUR 1UUS 1UUV 1UUW 1UUX 1UUY 1UUZ 1UV0 1UV4 1UV5 1UV6 1UV7 1UVA 1UVB 1UVC 1UVH 1UVO 1UVP 1UVQ 1UVR 1UVS 1UVT 1UVU 1UVX 1UVY 1UVZ 1UW1 1UW3 1UW4 1UW5 1UW6 1UW7 1UW8 1UW9 1UWA 1UWB 1UWC 1UWE 1UWF 1UWG 1UWH 1UWI 1UWJ 1UWK 1UWL 1UWM 1UWN 1UWP 1UWQ 1UWR 1UWS 1UWT 1UWU 1UWV 1UWW 1UWX 1UWY 1UWZ 1UX0 1UX1 1UX2 1UX4 1UX5 1UX6 1UX7 1UX8 1UX9 1UXA 1UXB 1UXE 1UXG 1UXH 1UXI 1UXJ 1UXK 1UXL 1UXM 1UXN 1UXO 1UXP 1UXQ 1UXR 1UXS 1UXT 1UXU 1UXV 1UXW 1UXX 1UXY 1UXZ 1UY0 1UY1 1UY2 1UY3 1UY4 1UY6 1UY7 1UY8 1UY9 1UYC 1UYD 1UYE 1UYF 1UYG 1UYH 1UYI 1UYJ 1UYK 1UYL 1UYM 1UYN 1UYO 1UYP 1UYQ 1UYR 1UYS 1UYT 1UYU 1UYV 1UYW 1UYX 1UYY 1UYZ 1UZ0 1UZ1 1UZ2 1UZ3 1UZ4 1UZ5 1UZ6 1UZ8 1UZ9 1UZA 1UZB 1UZD 1UZE 1UZF 1UZG 1UZH 1UZI 1UZJ 1UZK 1UZL 1UZM 1UZN 1UZP 1UZQ 1UZR 1UZU 1UZV 1UZW 1UZX 1UZY 1UZZ 1V00 1V02 1V03 1V04 1V05 1V07 1V08 1V0A 1V0B 1V0C 1V0D 1V0E 1V0F 1V0H 1V0J 1V0K 1V0L 1V0M 1V0N 1V0O 1V0P 1V0R 1V0S 1V0T 1V0U 1V0V 1V0W 1V0Y 1V0Z 1V10 1V11 1V13 1V16 1V18 1V19 1V1A 1V1B 1V1F 1V1G 1V1H 1V1I 1V1J 1V1K 1V1M 1V1O 1V1P 1V1Q 1V1R 1V1S 1V1T 1V25 1V26 1V29 1V2A 1V2B 1V2D 1V2E 1V2F 1V2G 1V2H 1V2I 1V2J 1V2K 1V2L 1V2M 1V2N 1V2O 1V2P 1V2Q 1V2R 1V2S 1V2T 1V2U 1V2V 1V2W 1V2X 1V2Z 1V30 1V33 1V34 1V35 1V37 1V39 1V3B 1V3C 1V3D 1V3E 1V3H 1V3I 1V3J 1V3K 1V3L 1V3M 1V3Q 1V3R 1V3S 1V3T 1V3U 1V3V 1V3W 1V3X 1V3Y 1V3Z 1V40 1V41 1V43 1V45 1V47 1V48 1V4A 1V4B 1V4E 1V4F 1V4G 1V4H 1V4I 1V4J 1V4K 1V4L 1V4N 1V4P 1V4S 1V4T 1V4U 1V4V 1V4W 1V4X 1V4Y 1V51 1V53 1V54 1V55 1V57 1V58 1V59 1V5B 1V5C 1V5D 1V5E 1V5F 1V5G 1V5H 1V5I 1V5V 1V5W 1V5X 1V5Y 1V5Z 1V67 1V6A 1V6C 1V6D 1V6H 1V6I 1V6J 1V6K 1V6L 1V6M 1V6N 1V6O 1V6P 1V6Q 1V6S 1V6T 1V6U 1V6V 1V6W 1V6X 1V6Y 1V6Z 1V70 1V71 1V72 1V73 1V74 1V75 1V76 1V77 1V79 1V7A 1V7C 1V7H 1V7L 1V7M 1V7N 1V7O 1V7P 1V7Q 1V7R 1V7S 1V7T 1V7U 1V7V 1V7W 1V7X 1V7Y 1V7Z 1V82 1V83 1V84 1V8B 1V8C 1V8D 1V8E 1V8F 1V8G 1V8H 1V8I 1V8J 1V8K 1V8L 1V8M 1V8N 1V8O 1V8P 1V8Q 1V8R 1V8S 1V8T 1V8U 1V8V 1V8W 1V8X 1V8Y 1V8Z 1V93 1V94 1V96 1V97 1V98 1V9A 1V9C 1V9D 1V9E 1V9F 1V9H 1V9I 1V9K 1V9L 1V9M 1V9N 1V9O 1V9P 1V9Q 1V9S 1V9T 1V9U 1V9Y 1V9Z 1VA0 1VA4 1VA5 1VA6 1VA7 1VAC 1VAD 1VAF 1VAG 1VAH 1VAI 1VAJ 1VAK 1VAL 1VAM 1VAO 1VAP 1VAR 1VAT 1VAU 1VAV 1VAX 1VAY 1VB0 1VB2 1VB3 1VB4 1VB5 1VB6 1VB9 1VBA 1VBB 1VBC 1VBD 1VBE 1VBF 1VBG 1VBH 1VBI 1VBJ 1VBK 1VBL 1VBM 1VBN 1VBO 1VBP 1VBR 1VBS 1VBT 1VBU 1VBV 1VBW 1VC1 1VC2 1VC3 1VC4 1VC8 1VC9 1VCA 1VCB 1VCC 1VCD 1VCE 1VCF 1VCG 1VCH 1VCI 1VCJ 1VCK 1VCL 1VCM 1VCN 1VCO 1VCP 1VCQ 1VCR 1VCT 1VCU 1VCV 1VCW 1VCY 1VCZ 1VD1 1VD3 1VD5 1VD6 1VDC 1VDD 1VDE 1VDF 1VDG 1VDH 1VDK 1VDM 1VDN 1VDP 1VDQ 1VDR 1VDS 1VDT 1VDV 1VDW 1VDX 1VDZ 1VE0 1VE1 1VE2 1VE3 1VE4 1VE5 1VE6 1VE7 1VE9 1VEA 1VEB 1VEC 1VED 1VEF 1VEI 1VEL 1VEM 1VEN 1VEO 1VEP 1VEQ 1VER 1VES 1VET 1VEU 1VEV 1VEW 1VEY 1VEZ 1VF1 1VF2 1VF3 1VF4 1VF5 1VF6 1VF7 1VF8 1VFA 1VFB 1VFD 1VFE 1VFF 1VFH 1VFJ 1VFL 1VFM 1VFN 1VFO 1VFP 1VFQ 1VFR 1VFS 1VFT 1VFU 1VFV 1VFW 1VFX 1VFY 1VFZ 1VG0 1VG1 1VG2 1VG3 1VG4 1VG6 1VG7 1VG8 1VG9 1VGA 1VGC 1VGE 1VGF 1VGG 1VGI 1VGJ 1VGK 1VGL 1VGM 1VGN 1VGO 1VGP 1VGQ 1VGR 1VGT 1VGU 1VGV 1VGW 1VGX 1VGY 1VGZ 1VH0 1VH1 1VH2 1VH3 1VH4 1VH5 1VH6 1VH7 1VH8 1VH9 1VHA 1VHB 1VHC 1VHD 1VHE 1VHF 1VHG 1VHH 1VHI 1VHJ 1VHK 1VHL 1VHM 1VHN 1VHO 1VHQ 1VHR 1VHS 1VHT 1VHU 1VHV 1VHW 1VHX 1VHY 1VHZ 1VI0 1VI1 1VI2 1VI3 1VI4 1VI5 1VI6 1VI7 1VI8 1VI9 1VIA 1VIC 1VID 1VIE 1VIF 1VIJ 1VIK 1VIM 1VIN 1VIO 1VIP 1VIQ 1VIS 1VIT 1VIU 1VIV 1VIW 1VIX 1VIY 1VIZ 1VJ0 1VJ1 1VJ2 1VJ3 1VJ5 1VJ7 1VJ9 1VJA 1VJB 1VJC 1VJD 1VJE 1VJF 1VJG 1VJH 1VJI 1VJK 1VJL 1VJM 1VJN 1VJO 1VJQ 1VJR 1VJS 1VJT 1VJU 1VJV 1VJW 1VJX 1VJY 1VJZ 1VK0 1VK1 1VK2 1VK3 1VK4 1VK5 1VK6 1VK8 1VK9 1VKA 1VKB 1VKC 1VKD 1VKE 1VKF 1VKG 1VKH 1VKI 1VKJ 1VKK 1VKL 1VKM 1VKN 1VKO 1VKP 1VKQ 1VKU 1VKW 1VKY 1VKZ 1VL0 1VL1 1VL2 1VL4 1VL5 1VL6 1VL7 1VL8 1VL9 1VLA 1VLB 1VLC 1VLG 1VLH 1VLI 1VLJ 1VLK 1VLL 1VLM 1VLN 1VLO 1VLP 1VLQ 1VLR 1VLS 1VLT 1VLU 1VLV 1VLW 1VLX 1VLY 1VLZ 1VM0 1VM1 1VM6 1VM7 1VM8 1VM9 1VMA 1VMB 1VMD 1VME 1VMF 1VMG 1VMH 1VMI 1VMJ 1VMK 1VMO 1VNC 1VNE 1VNF 1VNG 1VNH 1VNI 1VNS 1VOK 1VOM 1VOT 1VP2 1VP3 1VP4 1VP5 1VP6 1VP7 1VP8 1VP9 1VPA 1VPB 1VPD 1VPE 1VPF 1VPH 1VPI 1VPK 1VPL 1VPM 1VPN 1VPO 1VPP 1VPQ 1VPR 1VPS 1VPT 1VPV 1VPX 1VPY 1VPZ 1VQ0 1VQ1 1VQ2 1VQ3 1VQA 1VQB 1VQC 1VQD 1VQE 1VQF 1VQG 1VQH 1VQI 1VQJ 1VQQ 1VQR 1VQS 1VQT 1VQU 1VQV 1VQW 1VQY 1VQZ 1VR0 1VR1 1VR2 1VR3 1VR4 1VR5 1VR6 1VR7 1VR8 1VR9 1VRA 1VRB 1VRD 1VRG 1VRH 1VRK 1VRM 1VRN 1VRP 1VRQ 1VRS 1VRT 1VRU 1VRW 1VRX 1VRZ 1VS0 1VS1 1VS3 1VSB 1VSC 1VSD 1VSE 1VSF 1VSG 1VSH 1VSI 1VSJ 1VSK 1VSL 1VSM 1VSN 1VSO 1VSR 1VST 1VSU 1VSV 1VTK 1VUB 1VWA 1VWB 1VWC 1VWD 1VWE 1VWF 1VWG 1VWH 1VWI 1VWJ 1VWK 1VWL 1VWM 1VWN 1VWO 1VWP 1VWQ 1VWR 1VWT 1VXA 1VXB 1VXC 1VXD 1VXE 1VXF 1VXG 1VXH 1VXO 1VXR 1VYA 1VYB 1VYD 1VYF 1VYG 1VYH 1VYI 1VYJ 1VYK 1VYM 1VYO 1VYP 1VYQ 1VYR 1VYS 1VYT 1VYU 1VYV 1VYW 1VYZ 1VZ0 1VZ2 1VZ3 1VZ4 1VZ5 1VZ6 1VZ7 1VZ8 1VZA 1VZB 1VZC 1VZD 1VZE 1VZG 1VZH 1VZI 1VZJ 1VZM 1VZO 1VZQ 1VZT 1VZU 1VZV 1VZW 1VZX 1VZY 1VZZ 1W00 1W01 1W02 1W03 1W04 1W05 1W06 1W07 1W08 1W0C 1W0D 1W0E 1W0F 1W0G 1W0H 1W0I 1W0J 1W0K 1W0M 1W0N 1W0O 1W0P 1W0V 1W0W 1W0X 1W0Y 1W0Z 1W10 1W11 1W12 1W13 1W14 1W15 1W16 1W17 1W18 1W19 1W1A 1W1B 1W1D 1W1G 1W1H 1W1I 1W1J 1W1K 1W1L 1W1M 1W1O 1W1P 1W1Q 1W1R 1W1S 1W1T 1W1U 1W1V 1W1W 1W1X 1W1Y 1W1Z 1W20 1W21 1W22 1W23 1W24 1W25 1W26 1W27 1W28 1W29 1W2A 1W2C 1W2D 1W2E 1W2F 1W2G 1W2H 1W2I 1W2K 1W2L 1W2M 1W2N 1W2O 1W2P 1W2T 1W2U 1W2V 1W2W 1W2X 1W2Y 1W2Z 1W30 1W31 1W32 1W33 1W34 1W35 1W37 1W39 1W3A 1W3B 1W3C 1W3E 1W3F 1W3G 1W3H 1W3I 1W3J 1W3K 1W3L 1W3M 1W3N 1W3O 1W3P 1W3Q 1W3R 1W3S 1W3T 1W3U 1W3V 1W3W 1W3X 1W3Y 1W3Z 1W40 1W41 1W42 1W44 1W45 1W46 1W47 1W48 1W49 1W4A 1W4B 1W4C 1W4L 1W4N 1W4O 1W4P 1W4Q 1W4R 1W4S 1W4T 1W4V 1W4W 1W4X 1W4Y 1W4Z 1W50 1W51 1W52 1W53 1W54 1W55 1W56 1W57 1W58 1W59 1W5A 1W5B 1W5C 1W5D 1W5E 1W5F 1W5G 1W5H 1W5I 1W5J 1W5K 1W5L 1W5M 1W5N 1W5O 1W5P 1W5Q 1W5R 1W5S 1W5T 1W5U 1W5V 1W5W 1W5X 1W5Y 1W5Z 1W60 1W61 1W62 1W63 1W66 1W68 1W69 1W6C 1W6F 1W6G 1W6H 1W6I 1W6J 1W6K 1W6L 1W6M 1W6N 1W6O 1W6P 1W6Q 1W6R 1W6S 1W6T 1W6U 1W6W 1W6X 1W6Y 1W6Z 1W70 1W72 1W73 1W74 1W75 1W76 1W77 1W78 1W79 1W7B 1W7C 1W7F 1W7G 1W7H 1W7I 1W7J 1W7K 1W7L 1W7M 1W7N 1W7O 1W7P 1W7Q 1W7R 1W7S 1W7T 1W7U 1W7V 1W7W 1W7X 1W7Z 1W80 1W81 1W82 1W83 1W84 1W85 1W87 1W88 1W89 1W8A 1W8B 1W8C 1W8D 1W8E 1W8F 1W8G 1W8H 1W8I 1W8J 1W8K 1W8L 1W8M 1W8N 1W8O 1W8P 1W8Q 1W8S 1W8T 1W8U 1W8V 1W8W 1W8X 1W8Y 1W8Z 1W90 1W91 1W92 1W93 1W94 1W96 1W97 1W98 1W99 1W9A 1W9B 1W9C 1W9D 1W9E 1W9F 1W9G 1W9H 1W9I 1W9J 1W9K 1W9L 1W9M 1W9O 1W9P 1W9Q 1W9S 1W9T 1W9U 1W9V 1W9W 1W9X 1W9Y 1W9Z 1WA0 1WA1 1WA2 1WA3 1WA4 1WA5 1WA6 1WA9 1WAA 1WAB 1WAC 1WAD 1WAE 1WAF 1WAJ 1WAK 1WAL 1WAM 1WAO 1WAP 1WAQ 1WAR 1WAS 1WAT 1WAU 1WAV 1WAW 1WAX 1WAY 1WB0 1WB4 1WB5 1WB6 1WB7 1WB8 1WBA 1WBC 1WBE 1WBF 1WBG 1WBH 1WBI 1WBJ 1WBK 1WBL 1WBM 1WBN 1WBO 1WBP 1WBQ 1WBS 1WBT 1WBU 1WBV 1WBW 1WBX 1WBY 1WBZ 1WC0 1WC1 1WC2 1WC3 1WC4 1WC5 1WC6 1WC7 1WC8 1WC9 1WCB 1WCC 1WCD 1WCE 1WCF 1WCG 1WCH 1WCI 1WCK 1WCM 1WCQ 1WCS 1WCU 1WCV 1WCW 1WCX 1WCY 1WCZ 1WD3 1WD4 1WD5 1WD6 1WD7 1WD8 1WD9 1WDA 1WDC 1WDD 1WDE 1WDF 1WDG 1WDI 1WDJ 1WDK 1WDL 1WDM 1WDN 1WDP 1WDQ 1WDR 1WDS 1WDT 1WDU 1WDV 1WDW 1WDX 1WDY 1WDZ 1WE0 1WE1 1WE2 1WE4 1WE5 1WEF 1WEG 1WEH 1WEI 1WEJ 1WEK 1WER 1WF3 1WFA 1WFB 1WFC 1WFX 1WG0 1WG3 1WG8 1WGB 1WGC 1WGI 1WGJ 1WGT 1WGZ 1WHI 1WHO 1WHP 1WHS 1WHT 1WHZ 1WIO 1WIP 1WIQ 1WIW 1WIY 1WJ9 1WJG 1WJX 1WK2 1WK4 1WK8 1WK9 1WKA 1WKB 1WKC 1WKD 1WKE 1WKF 1WKG 1WKH 1WKJ 1WKK 1WKL 1WKM 1WKO 1WKP 1WKQ 1WKR 1WKU 1WKV 1WKW 1WKX 1WKY 1WKZ 1WL0 1WL1 1WL2 1WL3 1WL4 1WL5 1WL6 1WL7 1WL8 1WL9 1WLA 1WLC 1WLD 1WLE 1WLF 1WLG 1WLH 1WLI 1WLJ 1WLK 1WLR 1WLS 1WLT 1WLU 1WLV 1WLW 1WLY 1WLZ 1WM0 1WM1 1WM2 1WM3 1WM5 1WM6 1WM9 1WMA 1WMB 1WMD 1WME 1WMF 1WMG 1WMH 1WMI 1WMK 1WMM 1WMN 1WMO 1WMP 1WMR 1WMS 1WMU 1WMW 1WMX 1WMY 1WMZ 1WN0 1WN1 1WN2 1WN3 1WN5 1WN6 1WN7 1WN9 1WNA 1WNB 1WNC 1WND 1WNF 1WNG 1WNH 1WNI 1WNL 1WNO 1WNR 1WNS 1WNT 1WNU 1WNV 1WNW 1WNX 1WNY 1WNZ 1WO2 1WO8 1WOA 1WOB 1WOC 1WOD 1WOF 1WOG 1WOH 1WOI 1WOJ 1WOK 1WOL 1WOM 1WOO 1WOP 1WOQ 1WOR 1WOS 1WOU 1WOV 1WOW 1WOX 1WOY 1WOZ 1WP0 1WP1 1WP4 1WP5 1WP6 1WP7 1WP8 1WP9 1WPA 1WPB 1WPC 1WPG 1WPL 1WPM 1WPN 1WPO 1WPP 1WPQ 1WPR 1WPS 1WPT 1WPV 1WPW 1WPX 1WPY 1WQ1 1WQ3 1WQ4 1WQ5 1WQ6 1WQ7 1WQ8 1WQ9 1WQA 1WQF 1WQG 1WQH 1WQJ 1WQL 1WQM 1WQN 1WQO 1WQP 1WQQ 1WQR 1WQS 1WQV 1WQW 1WR2 1WR5 1WR6 1WR8 1WRA 1WRB 1WRD 1WRI 1WRJ 1WRK 1WRL 1WRM 1WRN 1WRO 1WRP 1WRR 1WRU 1WRV 1WRZ 1WS0 1WS1 1WS2 1WS3 1WS4 1WS5 1WS6 1WS7 1WS8 1WS9 1WSA 1WSB 1WSC 1WSD 1WSE 1WSF 1WSG 1WSH 1WSI 1WSJ 1WSP 1WSR 1WSS 1WST 1WSV 1WSW 1WSZ 1WT0 1WT1 1WT2 1WT3 1WT5 1WT6 1WT9 1WTA 1WTC 1WTD 1WTF 1WTG 1WTH 1WTJ 1WTL 1WTM 1WTN 1WTY 1WU1 1WU2 1WU3 1WU4 1WU5 1WU6 1WU7 1WU8 1WU9 1WUA 1WUB 1WUD 1WUE 1WUF 1WUH 1WUI 1WUJ 1WUK 1WUL 1WUN 1WUO 1WUP 1WUQ 1WUR 1WUT 1WUU 1WUV 1WUW 1WUY 1WV0 1WV1 1WV2 1WV3 1WV4 1WV7 1WV8 1WV9 1WVA 1WVB 1WVC 1WVE 1WVF 1WVG 1WVH 1WVI 1WVJ 1WVM 1WVN 1WVP 1WVQ 1WVR 1WVT 1WVU 1WVV 1WVW 1WVX 1WVY 1WW1 1WW2 1WW3 1WW4 1WW5 1WW6 1WW7 1WW8 1WW9 1WWA 1WWB 1WWC 1WWH 1WWI 1WWJ 1WWK 1WWL 1WWM 1WWP 1WWR 1WWS 1WWW 1WWZ 1WX0 1WX1 1WX2 1WX4 1WX5 1WXC 1WXD 1WXE 1WXF 1WXG 1WXH 1WXI 1WXJ 1WXO 1WXQ 1WXR 1WXW 1WXX 1WXY 1WXZ 1WY0 1WY1 1WY2 1WY3 1WY4 1WY5 1WY6 1WY7 1WY9 1WYB 1WYC 1WYD 1WYE 1WYG 1WYI 1WYK 1WYT 1WYU 1WYV 1WYW 1WYX 1WYY 1WYZ 1WZ1 1WZ3 1WZ7 1WZ8 1WZ9 1WZA 1WZB 1WZC 1WZD 1WZE 1WZF 1WZG 1WZI 1WZK 1WZL 1WZM 1WZN 1WZO 1WZU 1WZV 1WZW 1WZX 1WZY 1WZZ 1X01 1X03 1X04 1X06 1X07 1X08 1X09 1X0A 1X0C 1X0G 1X0I 1X0J 1X0K 1X0L 1X0M 1X0P 1X0R 1X0S 1X0T 1X0U 1X0V 1X0X 1X10 1X11 1X12 1X13 1X14 1X15 1X19 1X1A 1X1B 1X1C 1X1D 1X1E 1X1H 1X1I 1X1J 1X1K 1X1N 1X1O 1X1P 1X1Q 1X1R 1X1S 1X1T 1X1U 1X1V 1X1W 1X1X 1X1Y 1X1Z 1X23 1X24 1X25 1X27 1X28 1X29 1X2A 1X2B 1X2E 1X2G 1X2H 1X2I 1X2J 1X2R 1X2T 1X2W 1X31 1X33 1X35 1X36 1X38 1X39 1X3E 1X3F 1X3G 1X3K 1X3L 1X3M 1X3N 1X3O 1X3S 1X3W 1X3X 1X3Z 1X42 1X46 1X54 1X55 1X56 1X6I 1X6J 1X6L 1X6M 1X6N 1X6O 1X6P 1X6Q 1X6R 1X6U 1X6V 1X6X 1X6Y 1X6Z 1X70 1X71 1X74 1X75 1X76 1X77 1X78 1X79 1X7A 1X7B 1X7D 1X7E 1X7F 1X7G 1X7H 1X7I 1X7J 1X7N 1X7O 1X7P 1X7Q 1X7R 1X7S 1X7T 1X7U 1X7V 1X7W 1X7X 1X7Y 1X7Z 1X80 1X81 1X82 1X83 1X84 1X86 1X87 1X88 1X89 1X8B 1X8C 1X8D 1X8E 1X8F 1X8G 1X8H 1X8I 1X8J 1X8K 1X8L 1X8M 1X8N 1X8O 1X8P 1X8Q 1X8R 1X8S 1X8T 1X8U 1X8V 1X8X 1X8Y 1X8Z 1X90 1X91 1X92 1X94 1X96 1X97 1X98 1X99 1X9D 1X9E 1X9F 1X9G 1X9H 1X9I 1X9J 1X9P 1X9Q 1X9R 1X9T 1X9U 1X9Y 1X9Z 1XA0 1XA1 1XA3 1XA4 1XA5 1XA6 1XA7 1XA8 1XA9 1XAA 1XAB 1XAC 1XAD 1XAE 1XAF 1XAG 1XAH 1XAI 1XAJ 1XAK 1XAL 1XAN 1XAO 1XAP 1XAR 1XAS 1XAT 1XAU 1XAW 1XB0 1XB1 1XB2 1XB3 1XB4 1XB6 1XB7 1XB8 1XB9 1XBA 1XBB 1XBC 1XBF 1XBI 1XBN 1XBO 1XBS 1XBT 1XBU 1XBV 1XBW 1XBX 1XBY 1XBZ 1XC1 1XC3 1XC4 1XC6 1XC7 1XCA 1XCB 1XCC 1XCD 1XCF 1XCG 1XCH 1XCJ 1XCK 1XCL 1XCM 1XCO 1XCP 1XCQ 1XCR 1XCT 1XCV 1XCW 1XCX 1XD0 1XD1 1XD2 1XD3 1XD4 1XD5 1XD6 1XD7 1XD8 1XD9 1XDA 1XDB 1XDC 1XDD 1XDF 1XDG 1XDH 1XDI 1XDJ 1XDK 1XDL 1XDM 1XDN 1XDO 1XDP 1XDQ 1XDS 1XDT 1XDU 1XDV 1XDW 1XDY 1XDZ 1XE0 1XE1 1XE3 1XE4 1XE5 1XE6 1XE7 1XE8 1XEA 1XEB 1XEC 1XED 1XEF 1XEG 1XEI 1XEJ 1XEK 1XEL 1XEM 1XEN 1XEO 1XEP 1XEQ 1XER 1XES 1XET 1XEU 1XEV 1XEW 1XEX 1XEY 1XEZ 1XF0 1XF1 1XF3 1XF4 1XF5 1XF6 1XF8 1XF9 1XFA 1XFB 1XFC 1XFD 1XFF 1XFG 1XFH 1XFI 1XFJ 1XFK 1XFO 1XFP 1XFS 1XFU 1XFV 1XFW 1XFX 1XFY 1XFZ 1XG0 1XG2 1XG3 1XG4 1XG5 1XG6 1XG7 1XG8 1XGD 1XGE 1XGF 1XGI 1XGJ 1XGK 1XGM 1XGN 1XGO 1XGP 1XGQ 1XGR 1XGS 1XGT 1XGU 1XGV 1XGW 1XGY 1XGZ 1XH0 1XH1 1XH2 1XH3 1XH4 1XH5 1XH6 1XH7 1XH8 1XH9 1XHA 1XHB 1XHC 1XHD 1XHE 1XHF 1XHG 1XHK 1XHL 1XHM 1XHN 1XHO 1XHX 1XHY 1XI0 1XI2 1XI3 1XI6 1XI8 1XI9 1XIA 1XIB 1XIC 1XID 1XIE 1XIF 1XIG 1XIH 1XII 1XIJ 1XIK 1XIL 1XIM 1XIN 1XIO 1XIP 1XIQ 1XIS 1XIU 1XIV 1XIW 1XIX 1XIY 1XIZ 1XJ0 1XJ2 1XJ3 1XJ4 1XJ5 1XJ6 1XJ7 1XJA 1XJB 1XJC 1XJD 1XJE 1XJF 1XJG 1XJI 1XJJ 1XJK 1XJL 1XJM 1XJN 1XJO 1XJQ 1XJT 1XJU 1XJW 1XJZ 1XK0 1XK1 1XK2 1XK3 1XK4 1XK5 1XK6 1XK7 1XK8 1XK9 1XKA 1XKB 1XKD 1XKF 1XKG 1XKH 1XKI 1XKJ 1XKK 1XKL 1XKN 1XKO 1XKP 1XKQ 1XKR 1XKS 1XKT 1XKU 1XKV 1XKW 1XKX 1XKY 1XKZ 1XL0 1XL1 1XL2 1XL3 1XL4 1XL5 1XL6 1XL7 1XL8 1XL9 1XLA 1XLB 1XLC 1XLD 1XLE 1XLF 1XLG 1XLH 1XLI 1XLJ 1XLK 1XLL 1XLM 1XLN 1XLO 1XLP 1XLQ 1XLR 1XLS 1XLT 1XLU 1XLV 1XLW 1XLX 1XLY 1XLZ 1XM1 1XM2 1XM3 1XM4 1XM5 1XM6 1XM7 1XM8 1XM9 1XMA 1XMB 1XMC 1XMD 1XME 1XMF 1XMG 1XMH 1XMI 1XMJ 1XMK 1XML 1XMM 1XMN 1XMP 1XMS 1XMT 1XMU 1XMV 1XMX 1XMY 1XMZ 1XN0 1XN1 1XN2 1XN3 1XN4 1XNB 1XNC 1XND 1XNF 1XNG 1XNH 1XNI 1XNJ 1XNK 1XNN 1XNV 1XNW 1XNX 1XNY 1XNZ 1XO1 1XO2 1XO5 1XO6 1XO7 1XOC 1XOD 1XOE 1XOF 1XOG 1XOI 1XOM 1XON 1XOQ 1XOR 1XOS 1XOT 1XOU 1XOV 1XOW 1XOZ 1XP0 1XP1 1XP3 1XP4 1XP5 1XP6 1XP8 1XP9 1XPB 1XPC 1XPG 1XPH 1XPI 1XPJ 1XPK 1XPL 1XPM 1XPP 1XPQ 1XPS 1XPT 1XPY 1XPZ 1XQ0 1XQ1 1XQ3 1XQ4 1XQ5 1XQ6 1XQ7 1XQ9 1XQA 1XQB 1XQC 1XQD 1XQE 1XQF 1XQG 1XQH 1XQI 1XQJ 1XQK 1XQL 1XQM 1XQO 1XQP 1XQR 1XQS 1XQU 1XQV 1XQW 1XQX 1XQY 1XQZ 1XR1 1XR2 1XR3 1XR4 1XR5 1XR6 1XR7 1XR8 1XR9 1XRA 1XRB 1XRC 1XRE 1XRF 1XRG 1XRH 1XRI 1XRJ 1XRK 1XRL 1XRM 1XRN 1XRO 1XRP 1XRQ 1XRR 1XRS 1XRT 1XRU 1XRV 1XRX 1XRY 1XS0 1XS1 1XS2 1XS4 1XS5 1XS6 1XS7 1XSE 1XSI 1XSJ 1XSK 1XSM 1XSO 1XSQ 1XSR 1XSS 1XSV 1XSZ 1XT0 1XT3 1XT4 1XT5 1XT6 1XT8 1XT9 1XTA 1XTB 1XTC 1XTD 1XTE 1XTF 1XTG 1XTI 1XTJ 1XTK 1XTL 1XTM 1XTN 1XTO 1XTP 1XTQ 1XTR 1XTS 1XTT 1XTU 1XTV 1XTY 1XTZ 1XU1 1XU2 1XU3 1XU4 1XU5 1XU7 1XU8 1XU9 1XUA 1XUB 1XUC 1XUD 1XUF 1XUG 1XUH 1XUI 1XUJ 1XUK 1XUO 1XUP 1XUQ 1XUR 1XUU 1XUV 1XUZ 1XV2 1XV5 1XV8 1XV9 1XVA 1XVB 1XVC 1XVD 1XVE 1XVF 1XVG 1XVI 1XVJ 1XVL 1XVM 1XVO 1XVP 1XVQ 1XVS 1XVT 1XVU 1XVV 1XVW 1XVX 1XVY 1XW2 1XW3 1XW4 1XW5 1XW6 1XW7 1XW8 1XW9 1XWA 1XWB 1XWC 1XWD 1XWF 1XWG 1XWI 1XWJ 1XWK 1XWL 1XWM 1XWO 1XWQ 1XWR 1XWS 1XWT 1XWV 1XWW 1XWY 1XX1 1XX2 1XX4 1XX5 1XX6 1XX7 1XX9 1XXA 1XXB 1XXC 1XXD 1XXF 1XXG 1XXH 1XXI 1XXJ 1XXL 1XXM 1XXN 1XXO 1XXP 1XXQ 1XXR 1XXS 1XXT 1XXU 1XXV 1XXW 1XXX 1XY0 1XY1 1XY2 1XY3 1XY7 1XYA 1XYB 1XYC 1XYE 1XYF 1XYG 1XYH 1XYL 1XYM 1XYN 1XYO 1XYP 1XYS 1XYV 1XYY 1XYZ 1XZ0 1XZ1 1XZ2 1XZ3 1XZ4 1XZ5 1XZ6 1XZ7 1XZ8 1XZA 1XZB 1XZC 1XZD 1XZE 1XZF 1XZG 1XZH 1XZI 1XZJ 1XZK 1XZL 1XZM 1XZN 1XZO 1XZP 1XZQ 1XZU 1XZV 1XZW 1XZX 1XZZ 1Y01 1Y02 1Y07 1Y08 1Y09 1Y0A 1Y0B 1Y0C 1Y0D 1Y0E 1Y0G 1Y0H 1Y0K 1Y0L 1Y0M 1Y0N 1Y0O 1Y0P 1Y0R 1Y0S 1Y0T 1Y0U 1Y0V 1Y0W 1Y0X 1Y0Y 1Y0Z 1Y10 1Y11 1Y12 1Y13 1Y14 1Y17 1Y18 1Y19 1Y1A 1Y1D 1Y1E 1Y1F 1Y1G 1Y1H 1Y1I 1Y1J 1Y1K 1Y1L 1Y1M 1Y1N 1Y1O 1Y1P 1Y1Q 1Y1R 1Y1S 1Y1T 1Y1U 1Y1V 1Y1X 1Y1Z 1Y20 1Y21 1Y22 1Y23 1Y25 1Y28 1Y2A 1Y2B 1Y2C 1Y2D 1Y2E 1Y2F 1Y2G 1Y2H 1Y2I 1Y2J 1Y2K 1Y2M 1Y2O 1Y2Q 1Y2T 1Y2U 1Y2V 1Y2W 1Y2X 1Y2Z 1Y30 1Y31 1Y33 1Y34 1Y35 1Y37 1Y38 1Y3A 1Y3B 1Y3C 1Y3D 1Y3F 1Y3G 1Y3H 1Y3I 1Y3N 1Y3P 1Y3Q 1Y3T 1Y3U 1Y3V 1Y3W 1Y3X 1Y3Y 1Y42 1Y43 1Y44 1Y45 1Y46 1Y47 1Y48 1Y4A 1Y4B 1Y4C 1Y4D 1Y4F 1Y4G 1Y4H 1Y4I 1Y4J 1Y4K 1Y4L 1Y4M 1Y4P 1Y4Q 1Y4R 1Y4S 1Y4T 1Y4U 1Y4V 1Y4W 1Y4Y 1Y4Z 1Y50 1Y51 1Y52 1Y53 1Y54 1Y55 1Y56 1Y57 1Y59 1Y5A 1Y5B 1Y5E 1Y5F 1Y5H 1Y5I 1Y5J 1Y5K 1Y5L 1Y5M 1Y5N 1Y5R 1Y5U 1Y5V 1Y5W 1Y5X 1Y5Y 1Y60 1Y62 1Y63 1Y64 1Y65 1Y66 1Y67 1Y6A 1Y6B 1Y6E 1Y6H 1Y6I 1Y6J 1Y6K 1Y6L 1Y6M 1Y6N 1Y6O 1Y6P 1Y6Q 1Y6R 1Y6V 1Y6W 1Y6X 1Y6Z 1Y71 1Y75 1Y79 1Y7A 1Y7B 1Y7C 1Y7D 1Y7E 1Y7G 1Y7H 1Y7I 1Y7L 1Y7M 1Y7O 1Y7P 1Y7R 1Y7T 1Y7U 1Y7V 1Y7W 1Y7Y 1Y7Z 1Y80 1Y81 1Y82 1Y83 1Y85 1Y88 1Y89 1Y8A 1Y8C 1Y8E 1Y8G 1Y8H 1Y8I 1Y8J 1Y8K 1Y8N 1Y8O 1Y8P 1Y8Q 1Y8R 1Y8T 1Y8W 1Y8X 1Y8Y 1Y91 1Y92 1Y93 1Y94 1Y96 1Y97 1Y98 1Y9A 1Y9B 1Y9D 1Y9E 1Y9G 1Y9I 1Y9K 1Y9L 1Y9M 1Y9Q 1Y9R 1Y9T 1Y9U 1Y9W 1Y9Z 1YA0 1YA3 1YA4 1YA5 1YA7 1YA8 1YA9 1YAA 1YAB 1YAC 1YAD 1YAE 1YAF 1YAG 1YAH 1YAI 1YAJ 1YAK 1YAL 1YAM 1YAN 1YAO 1YAP 1YAQ 1YAR 1YAS 1YAT 1YAU 1YAV 1YAX 1YAZ 1YB0 1YB1 1YB2 1YB3 1YB4 1YB5 1YB6 1YB7 1YBA 1YBD 1YBE 1YBF 1YBG 1YBH 1YBI 1YBK 1YBM 1YBO 1YBQ 1YBT 1YBU 1YBV 1YBW 1YBX 1YBY 1YBZ 1YC0 1YC1 1YC2 1YC3 1YC4 1YC5 1YC6 1YC7 1YC8 1YC9 1YCA 1YCB 1YCC 1YCD 1YCE 1YCF 1YCG 1YCH 1YCI 1YCJ 1YCK 1YCL 1YCN 1YCO 1YCP 1YCQ 1YCR 1YCS 1YCY 1YCZ 1YD0 1YD1 1YD2 1YD3 1YD4 1YD5 1YD6 1YD7 1YD8 1YD9 1YDA 1YDB 1YDC 1YDD 1YDE 1YDF 1YDG 1YDH 1YDI 1YDK 1YDL 1YDM 1YDN 1YDO 1YDP 1YDR 1YDS 1YDT 1YDV 1YDW 1YDX 1YDY 1YDZ 1YE0 1YE1 1YE2 1YE3 1YE4 1YE5 1YE6 1YE8 1YE9 1YEA 1YEB 1YEC 1YED 1YEE 1YEF 1YEG 1YEH 1YEI 1YEJ 1YEK 1YEM 1YEN 1YEO 1YEP 1YEQ 1YER 1YES 1YET 1YEU 1YEV 1YEW 1YEX 1YEY 1YF0 1YF1 1YF2 1YF4 1YF5 1YF6 1YF8 1YF9 1YFD 1YFE 1YFF 1YFK 1YFM 1YFN 1YFO 1YFP 1YFQ 1YFR 1YFS 1YFT 1YFU 1YFW 1YFX 1YFY 1YFZ 1YG2 1YG5 1YG6 1YG8 1YG9 1YGA 1YGB 1YGC 1YGD 1YGE 1YGF 1YGG 1YGH 1YGJ 1YGK 1YGP 1YGR 1YGS 1YGT 1YGU 1YGY 1YGZ 1YH2 1YH3 1YH8 1YH9 1YHA 1YHB 1YHC 1YHE 1YHF 1YHG 1YHH 1YHI 1YHJ 1YHK 1YHL 1YHM 1YHN 1YHR 1YHS 1YHT 1YHU 1YHV 1YHW 1YHY 1YHZ 1YI0 1YI1 1YI3 1YI4 1YI5 1YI6 1YI7 1YI8 1YI9 1YIA 1YIB 1YID 1YIE 1YIF 1YIG 1YIH 1YII 1YIK 1YIL 1YIM 1YIN 1YIO 1YIP 1YIQ 1YIR 1YIS 1YIV 1YIW 1YIX 1YIY 1YIZ 1YJ0 1YJ1 1YJ2 1YJ3 1YJ4 1YJ5 1YJ6 1YJ7 1YJ8 1YJA 1YJB 1YJC 1YJD 1YJE 1YJF 1YJG 1YJH 1YJK 1YJL 1YJM 1YJO 1YJP 1YJQ 1YJS 1YJX 1YJY 1YJZ 1YK0 1YK1 1YK3 1YK4 1YK5 1YK7 1YK8 1YK9 1YKB 1YKC 1YKD 1YKE 1YKF 1YKH 1YKI 1YKJ 1YKK 1YKL 1YKM 1YKN 1YKO 1YKP 1YKR 1YKS 1YKT 1YKW 1YKX 1YKY 1YKZ 1YL0 1YL1 1YL5 1YL6 1YL7 1YLA 1YLC 1YLD 1YLE 1YLF 1YLH 1YLI 1YLJ 1YLK 1YLL 1YLM 1YLN 1YLO 1YLP 1YLQ 1YLR 1YLT 1YLU 1YLV 1YLW 1YLX 1YLY 1YLZ 1YM0 1YM1 1YM2 1YM3 1YM4 1YM5 1YM7 1YM8 1YM9 1YMA 1YMB 1YMC 1YMD 1YME 1YMG 1YMH 1YMK 1YML 1YMM 1YMN 1YMP 1YMQ 1YMR 1YMS 1YMT 1YMU 1YMV 1YMW 1YMX 1YMY 1YN3 1YN4 1YN5 1YN6 1YN7 1YN8 1YN9 1YNA 1YNB 1YND 1YNF 1YNH 1YNI 1YNJ 1YNK 1YNL 1YNM 1YNN 1YNO 1YNP 1YNQ 1YNR 1YNS 1YNT 1YNU 1YNV 1YNY 1YNZ 1YO0 1YO1 1YO2 1YO3 1YO6 1YO7 1YO8 1YOA 1YOB 1YOC 1YOD 1YOE 1YOG 1YOH 1YOI 1YOJ 1YOK 1YOL 1YOM 1YON 1YOO 1YOU 1YOV 1YOW 1YOX 1YOY 1YOZ 1YP0 1YP1 1YP2 1YP3 1YP4 1YP5 1YP6 1YP7 1YP9 1YPA 1YPB 1YPC 1YPE 1YPF 1YPG 1YPH 1YPI 1YPJ 1YPK 1YPL 1YPM 1YPN 1YPO 1YPP 1YPQ 1YPR 1YPT 1YPU 1YPV 1YPX 1YPY 1YPZ 1YQ1 1YQ2 1YQ3 1YQ4 1YQ5 1YQ6 1YQ7 1YQ8 1YQ9 1YQB 1YQC 1YQD 1YQE 1YQF 1YQG 1YQH 1YQJ 1YQN 1YQO 1YQP 1YQQ 1YQS 1YQT 1YQU 1YQV 1YQW 1YQX 1YQY 1YQZ 1YR0 1YR2 1YR3 1YR5 1YR6 1YR7 1YR8 1YR9 1YRA 1YRB 1YRC 1YRD 1YRE 1YRF 1YRG 1YRH 1YRI 1YRK 1YRL 1YRO 1YRP 1YRQ 1YRR 1YRS 1YRT 1YRU 1YRV 1YRW 1YRX 1YRY 1YRZ 1YS0 1YS1 1YS2 1YS3 1YS4 1YS6 1YS7 1YS9 1YSB 1YSC 1YSD 1YSJ 1YSL 1YSO 1YSP 1YSQ 1YSR 1YST 1YSZ 1YT0 1YT1 1YT2 1YT3 1YT4 1YT5 1YT7 1YT8 1YT9 1YTA 1YTC 1YTD 1YTE 1YTG 1YTH 1YTI 1YTJ 1YTK 1YTL 1YTM 1YTN 1YTO 1YTQ 1YTS 1YTT 1YTV 1YTW 1YTZ 1YU0 1YU1 1YU2 1YU3 1YU4 1YU5 1YU6 1YU7 1YU8 1YU9 1YUC 1YUD 1YUE 1YUH 1YUK 1YUL 1YUM 1YUN 1YUO 1YUP 1YUW 1YUX 1YUY 1YUZ 1YV0 1YV1 1YV2 1YV3 1YV4 1YV5 1YV6 1YV7 1YV9 1YVB 1YVD 1YVE 1YVF 1YVG 1YVH 1YVI 1YVJ 1YVK 1YVL 1YVM 1YVN 1YVO 1YVQ 1YVR 1YVS 1YVT 1YVU 1YVW 1YVX 1YVY 1YVZ 1YW0 1YW1 1YW2 1YW4 1YW5 1YW6 1YW7 1YW8 1YW9 1YWA 1YWB 1YWC 1YWD 1YWF 1YWG 1YWH 1YWK 1YWM 1YWN 1YWO 1YWP 1YWQ 1YWR 1YWT 1YWV 1YX1 1YX2 1YX9 1YXA 1YXB 1YXC 1YXD 1YXH 1YXI 1YXJ 1YXK 1YXL 1YXM 1YXO 1YXQ 1YXS 1YXT 1YXU 1YXV 1YXW 1YXX 1YXY 1YY3 1YY4 1YY5 1YY6 1YY7 1YY8 1YY9 1YYA 1YYD 1YYE 1YYF 1YYG 1YYH 1YYL 1YYM 1YYN 1YYP 1YYQ 1YYR 1YYS 1YYT 1YYU 1YYV 1YYY 1YYZ 1YZ0 1YZ1 1YZ3 1YZ4 1YZ5 1YZ6 1YZ7 1YZE 1YZF 1YZG 1YZH 1YZI 1YZK 1YZL 1YZM 1YZN 1YZP 1YZQ 1YZR 1YZT 1YZU 1YZV 1YZW 1YZX 1YZY 1YZZ 1Z01 1Z02 1Z03 1Z05 1Z06 1Z07 1Z08 1Z0A 1Z0B 1Z0C 1Z0D 1Z0E 1Z0F 1Z0G 1Z0H 1Z0I 1Z0J 1Z0K 1Z0M 1Z0N 1Z0P 1Z0S 1Z0T 1Z0U 1Z0V 1Z0W 1Z0X 1Z0Z 1Z10 1Z11 1Z12 1Z13 1Z14 1Z15 1Z16 1Z17 1Z18 1Z1A 1Z1E 1Z1F 1Z1H 1Z1I 1Z1J 1Z1L 1Z1N 1Z1P 1Z1Q 1Z1R 1Z1S 1Z1W 1Z1X 1Z1Y 1Z21 1Z22 1Z24 1Z25 1Z26 1Z27 1Z28 1Z29 1Z2A 1Z2B 1Z2C 1Z2I 1Z2L 1Z2M 1Z2N 1Z2O 1Z2P 1Z2U 1Z2V 1Z2W 1Z2X 1Z2Z 1Z32 1Z33 1Z34 1Z35 1Z36 1Z37 1Z38 1Z39 1Z3A 1Z3C 1Z3D 1Z3E 1Z3G 1Z3H 1Z3I 1Z3L 1Z3M 1Z3N 1Z3P 1Z3Q 1Z3S 1Z3T 1Z3U 1Z3V 1Z3W 1Z3X 1Z3Y 1Z3Z 1Z40 1Z41 1Z42 1Z44 1Z45 1Z47 1Z48 1Z4A 1Z4E 1Z4I 1Z4J 1Z4K 1Z4L 1Z4M 1Z4N 1Z4O 1Z4P 1Z4Q 1Z4R 1Z4S 1Z4U 1Z4V 1Z4W 1Z4X 1Z4Y 1Z4Z 1Z50 1Z52 1Z53 1Z54 1Z55 1Z56 1Z57 1Z59 1Z5A 1Z5B 1Z5C 1Z5G 1Z5H 1Z5L 1Z5M 1Z5N 1Z5O 1Z5P 1Z5R 1Z5S 1Z5U 1Z5V 1Z5W 1Z5X 1Z5Y 1Z5Z 1Z62 1Z67 1Z68 1Z69 1Z6A 1Z6B 1Z6D 1Z6E 1Z6F 1Z6G 1Z6I 1Z6J 1Z6K 1Z6L 1Z6M 1Z6N 1Z6O 1Z6P 1Z6Q 1Z6R 1Z6S 1Z6T 1Z6U 1Z6X 1Z6Y 1Z6Z 1Z70 1Z71 1Z72 1Z73 1Z74 1Z75 1Z76 1Z77 1Z78 1Z7A 1Z7B 1Z7C 1Z7D 1Z7E 1Z7G 1Z7H 1Z7J 1Z7K 1Z7L 1Z7M 1Z7N 1Z7Q 1Z7S 1Z7U 1Z7W 1Z7X 1Z7Y 1Z81 1Z82 1Z83 1Z84 1Z85 1Z88 1Z89 1Z8A 1Z8C 1Z8D 1Z8F 1Z8G 1Z8H 1Z8I 1Z8J 1Z8K 1Z8L 1Z8N 1Z8O 1Z8P 1Z8Q 1Z8T 1Z8U 1Z8W 1Z8X 1Z90 1Z91 1Z92 1Z93 1Z94 1Z95 1Z96 1Z97 1Z98 1Z9A 1Z9D 1Z9F 1Z9G 1Z9H 1Z9J 1Z9K 1Z9L 1Z9M 1Z9N 1Z9O 1Z9P 1Z9S 1Z9T 1Z9U 1Z9W 1Z9X 1Z9Y 1Z9Z 1ZA0 1ZA1 1ZA2 1ZA3 1ZA4 1ZA5 1ZA6 1ZA7 1ZAB 1ZAF 1ZAG 1ZAH 1ZAI 1ZAJ 1ZAK 1ZAL 1ZAN 1ZAO 1ZAP 1ZAR 1ZAT 1ZAU 1ZAV 1ZAW 1ZAX 1ZB1 1ZB5 1ZB6 1ZB7 1ZB8 1ZB9 1ZBA 1ZBC 1ZBD 1ZBE 1ZBF 1ZBG 1ZBK 1ZBM 1ZBO 1ZBP 1ZBQ 1ZBR 1ZBS 1ZBT 1ZBU 1ZBV 1ZBW 1ZBX 1ZBY 1ZBZ 1ZC0 1ZC2 1ZC3 1ZC4 1ZC6 1ZC9 1ZCA 1ZCB 1ZCC 1ZCD 1ZCE 1ZCF 1ZCH 1ZCJ 1ZCK 1ZCL 1ZCM 1ZCN 1ZCO 1ZCP 1ZCR 1ZCT 1ZCU 1ZCV 1ZCW 1ZCY 1ZCZ 1ZD0 1ZD1 1ZD2 1ZD3 1ZD4 1ZD5 1ZD6 1ZD7 1ZD8 1ZD9 1ZDE 1ZDF 1ZDG 1ZDL 1ZDM 1ZDN 1ZDP 1ZDQ 1ZDR 1ZDS 1ZDT 1ZDU 1ZDW 1ZDY 1ZE1 1ZE3 1ZE8 1ZEA 1ZEB 1ZED 1ZEE 1ZEF 1ZEG 1ZEH 1ZEI 1ZEJ 1ZEL 1ZEM 1ZEN 1ZEO 1ZEQ 1ZES 1ZFJ 1ZFK 1ZFN 1ZFP 1ZFQ 1ZG3 1ZG4 1ZG6 1ZG7 1ZG8 1ZG9 1ZGA 1ZGB 1ZGC 1ZGD 1ZGE 1ZGF 1ZGH 1ZGI 1ZGJ 1ZGK 1ZGL 1ZGN 1ZGO 1ZGP 1ZGQ 1ZGR 1ZGS 1ZGT 1ZGV 1ZGX 1ZGY 1ZGZ 1ZH0 1ZH1 1ZH2 1ZH4 1ZH6 1ZH7 1ZH8 1ZH9 1ZHA 1ZHB 1ZHF 1ZHG 1ZHH 1ZHI 1ZHJ 1ZHK 1ZHL 1ZHM 1ZHN 1ZHP 1ZHQ 1ZHR 1ZHS 1ZHT 1ZHV 1ZHW 1ZHX 1ZHY 1ZHZ 1ZI0 1ZI1 1ZI3 1ZI4 1ZI5 1ZI6 1ZI7 1ZI8 1ZI9 1ZIA 1ZIB 1ZIC 1ZID 1ZIE 1ZII 1ZIJ 1ZIK 1ZIL 1ZIM 1ZIN 1ZIO 1ZIP 1ZIQ 1ZIR 1ZIS 1ZIU 1ZIV 1ZIW 1ZIX 1ZIY 1ZIZ 1ZJ0 1ZJ1 1ZJ2 1ZJ3 1ZJ4 1ZJ5 1ZJ6 1ZJ7 1ZJ8 1ZJ9 1ZJA 1ZJB 1ZJC 1ZJD 1ZJH 1ZJI 1ZJJ 1ZJK 1ZJL 1ZJO 1ZJP 1ZJR 1ZJY 1ZJZ 1ZK0 1ZK1 1ZK2 1ZK3 1ZK4 1ZK5 1ZK7 1ZK8 1ZK9 1ZKA 1ZKB 1ZKC 1ZKD 1ZKE 1ZKF 1ZKG 1ZKI 1ZKJ 1ZKK 1ZKL 1ZKM 1ZKN 1ZKO 1ZKP 1ZKQ 1ZKR 1ZKW 1ZKX 1ZKY 1ZKZ 1ZL0 1ZL1 1ZL2 1ZL5 1ZL6 1ZL7 1ZL9 1ZLB 1ZLD 1ZLE 1ZLF 1ZLH 1ZLI 1ZLJ 1ZLM 1ZLP 1ZLQ 1ZLR 1ZLS 1ZLT 1ZLU 1ZLV 1ZLW 1ZLX 1ZLY 1ZLZ 1ZM0 1ZM1 1ZM2 1ZM3 1ZM4 1ZM6 1ZM7 1ZM8 1ZM9 1ZMA 1ZMB 1ZMC 1ZMD 1ZMF 1ZMG 1ZMH 1ZMI 1ZMJ 1ZMK 1ZML 1ZMM 1ZMN 1ZMO 1ZMP 1ZMQ 1ZMR 1ZMS 1ZMT 1ZMU 1ZMV 1ZMW 1ZMX 1ZMY 1ZN2 1ZN3 1ZN6 1ZN7 1ZN8 1ZN9 1ZNB 1ZNC 1ZND 1ZNE 1ZNG 1ZNH 1ZNI 1ZNJ 1ZNK 1ZNL 1ZNN 1ZNO 1ZNP 1ZNQ 1ZNV 1ZNW 1ZNX 1ZNY 1ZNZ 1ZO2 1ZO4 1ZO8 1ZO9 1ZOA 1ZOB 1ZOD 1ZOE 1ZOF 1ZOG 1ZOH 1ZOI 1ZOL 1ZOM 1ZON 1ZOO 1ZOP 1ZOQ 1ZOR 1ZOS 1ZOT 1ZOV 1ZOW 1ZOX 1ZOY 1ZP0 1ZP2 1ZP3 1ZP4 1ZP5 1ZP6 1ZP7 1ZP8 1ZP9 1ZPA 1ZPB 1ZPC 1ZPD 1ZPE 1ZPG 1ZPK 1ZPL 1ZPQ 1ZPR 1ZPS 1ZPT 1ZPU 1ZPV 1ZPW 1ZPZ 1ZQ1 1ZQ5 1ZQ7 1ZQ9 1ZQU 1ZQV 1ZQW 1ZQX 1ZQY 1ZQZ 1ZR0 1ZR3 1ZR5 1ZR6 1ZR8 1ZRB 1ZRH 1ZRK 1ZRL 1ZRM 1ZRN 1ZRO 1ZRQ 1ZRS 1ZRT 1ZRU 1ZRZ 1ZS0 1ZS2 1ZS3 1ZS6 1ZS7 1ZS8 1ZS9 1ZSA 1ZSB 1ZSC 1ZSD 1ZSF 1ZSH 1ZSJ 1ZSK 1ZSL 1ZSN 1ZSO 1ZSP 1ZSQ 1ZSR 1ZSV 1ZSW 1ZSX 1ZSY 1ZSZ 1ZT1 1ZT2 1ZT3 1ZT4 1ZT5 1ZT7 1ZT9 1ZTB 1ZTC 1ZTD 1ZTE 1ZTF 1ZTH 1ZTJ 1ZTK 1ZTL 1ZTM 1ZTP 1ZTQ 1ZTU 1ZTV 1ZTX 1ZTY 1ZTZ 1ZU0 1ZU3 1ZU4 1ZU5 1ZU8 1ZUA 1ZUC 1ZUD 1ZUH 1ZUI 1ZUJ 1ZUK 1ZUM 1ZUN 1ZUO 1ZUP 1ZUQ 1ZUR 1ZUT 1ZUU 1ZUW 1ZUX 1ZUY 1ZUZ 1ZV1 1ZV2 1ZV4 1ZV5 1ZV7 1ZV8 1ZV9 1ZVA 1ZVB 1ZVC 1ZVD 1ZVE 1ZVF 1ZVG 1ZVH 1ZVI 1ZVJ 1ZVK 1ZVL 1ZVM 1ZVN 1ZVP 1ZVQ 1ZVR 1ZVS 1ZVT 1ZVU 1ZVW 1ZVX 1ZVY 1ZVZ 1ZW0 1ZW1 1ZW2 1ZW3 1ZW5 1ZW6 1ZW9 1ZWH 1ZWI 1ZWJ 1ZWK 1ZWL 1ZWN 1ZWP 1ZWS 1ZWW 1ZWX 1ZWY 1ZWZ 1ZX1 1ZX2 1ZX3 1ZX5 1ZX6 1ZX8 1ZX9 1ZXB 1ZXC 1ZXE 1ZXI 1ZXJ 1ZXK 1ZXL 1ZXM 1ZXN 1ZXO 1ZXQ 1ZXT 1ZXU 1ZXV 1ZXX 1ZXY 1ZXZ 1ZY0 1ZY1 1ZY2 1ZY4 1ZY5 1ZY7 1ZY8 1ZY9 1ZYB 1ZYC 1ZYD 1ZYE 1ZYJ 1ZYK 1ZYL 1ZYM 1ZYN 1ZYO 1ZYP 1ZYR 1ZYS 1ZYU 1ZYV 1ZYW 1ZYX 1ZYZ 1ZZ0 1ZZ1 1ZZ2 1ZZ3 1ZZ6 1ZZ7 1ZZ8 1ZZ9 1ZZB 1ZZC 1ZZD 1ZZE 1ZZG 1ZZH 1ZZK 1ZZL 1ZZM 1ZZO 1ZZQ 1ZZR 1ZZS 1ZZT 1ZZU 1ZZW 1ZZY 1ZZZ 200L 201L 205L 206L 207L 208L 209L 20GS 210L 211L 212L 213L 214L 215L 216L 217L 218L 219L 21BI 220L 221L 221P 222L 223L 224L 225L 226L 227L 228L 229L 22GS 230L 231L 232L 233L 234L 235L 236L 237L 238L 239L 240L 241L 242L 243L 244L 245L 246L 247L 248L 249L 250L 251L 252L 253L 254L 255L 256B 256L 257L 258L 259L 25C8 260L 261L 262L 2A01 2A03 2A06 2A08 2A0B 2A0C 2A0F 2A0J 2A0K 2A0L 2A0M 2A0N 2A0Q 2A0S 2A0U 2A0W 2A0X 2A0Y 2A0Z 2A10 2A11 2A13 2A14 2A15 2A18 2A19 2A1A 2A1B 2A1D 2A1E 2A1F 2A1H 2A1I 2A1J 2A1K 2A1L 2A1M 2A1N 2A1O 2A1S 2A1T 2A1U 2A1V 2A1W 2A1X 2A1Y 2A21 2A22 2A25 2A26 2A27 2A28 2A2A 2A2C 2A2D 2A2F 2A2G 2A2I 2A2J 2A2K 2A2L 2A2M 2A2N 2A2O 2A2Q 2A2R 2A2S 2A2U 2A2X 2A2Z 2A30 2A31 2A32 2A33 2A35 2A38 2A39 2A3A 2A3B 2A3C 2A3E 2A3F 2A3G 2A3H 2A3I 2A3K 2A3L 2A3M 2A3N 2A3P 2A3Q 2A3R 2A3T 2A3U 2A3W 2A3X 2A3Y 2A3Z 2A40 2A41 2A42 2A45 2A46 2A47 2A48 2A49 2A4A 2A4C 2A4D 2A4E 2A4F 2A4G 2A4K 2A4L 2A4M 2A4N 2A4O 2A4Q 2A4R 2A4T 2A4V 2A4W 2A4X 2A4Z 2A50 2A52 2A53 2A54 2A56 2A57 2A58 2A59 2A5A 2A5B 2A5C 2A5D 2A5F 2A5G 2A5H 2A5I 2A5J 2A5K 2A5L 2A5S 2A5T 2A5U 2A5V 2A5W 2A5X 2A5Y 2A5Z 2A61 2A62 2A65 2A67 2A68 2A69 2A6A 2A6B 2A6C 2A6D 2A6E 2A6H 2A6I 2A6J 2A6K 2A6L 2A6M 2A6N 2A6P 2A6Q 2A6R 2A6S 2A6T 2A6V 2A6W 2A6X 2A6Y 2A6Z 2A70 2A71 2A72 2A73 2A74 2A75 2A77 2A78 2A79 2A7A 2A7B 2A7C 2A7D 2A7F 2A7G 2A7H 2A7I 2A7J 2A7K 2A7L 2A7M 2A7N 2A7P 2A7Q 2A7R 2A7S 2A7T 2A7W 2A7X 2A81 2A83 2A84 2A85 2A86 2A87 2A88 2A89 2A8A 2A8B 2A8C 2A8D 2A8E 2A8F 2A8G 2A8H 2A8I 2A8J 2A8K 2A8L 2A8M 2A8N 2A8P 2A8Q 2A8R 2A8S 2A8T 2A8U 2A8W 2A8X 2A8Y 2A8Z 2A90 2A91 2A92 2A94 2A96 2A97 2A98 2A99 2A9A 2A9B 2A9C 2A9D 2A9E 2A9F 2A9G 2A9I 2A9J 2A9K 2A9M 2A9N 2A9O 2A9P 2A9Q 2A9R 2A9S 2A9U 2A9V 2A9W 2A9Y 2A9Z 2AA0 2AA1 2AA2 2AA3 2AA4 2AA5 2AA6 2AA7 2AA9 2AAA 2AAB 2AAC 2AAD 2AAE 2AAF 2AAG 2AAI 2AAJ 2AAK 2AAL 2AAM 2AAN 2AAO 2AAQ 2AAT 2AAW 2AAX 2AAY 2AAZ 2AB0 2AB1 2AB2 2AB5 2AB6 2AB8 2ABA 2ABB 2ABE 2ABH 2ABI 2ABJ 2ABK 2ABL 2ABM 2ABQ 2ABR 2ABS 2ABW 2ABX 2ABZ 2AC1 2AC2 2AC3 2AC4 2AC5 2AC7 2ACA 2ACE 2ACF 2ACG 2ACH 2ACI 2ACK 2ACL 2ACO 2ACP 2ACQ 2ACR 2ACS 2ACT 2ACU 2ACV 2ACW 2ACX 2ACY 2ACZ 2AD1 2AD5 2AD6 2AD7 2AD8 2ADA 2ADD 2ADE 2ADF 2ADG 2ADI 2ADJ 2ADM 2ADO 2ADP 2ADQ 2ADU 2ADV 2AE0 2AE1 2AE2 2AE3 2AE4 2AE5 2AE6 2AE7 2AE8 2AEB 2AEC 2AEE 2AEF 2AEG 2AEH 2AEI 2AEJ 2AEK 2AEL 2AEM 2AEN 2AEO 2AEP 2AEQ 2AER 2AES 2AET 2AEU 2AEV 2AEW 2AEX 2AEY 2AEZ 2AF0 2AF3 2AF4 2AF5 2AF6 2AF7 2AF9 2AFA 2AFB 2AFC 2AFG 2AFH 2AFI 2AFM 2AFN 2AFO 2AFQ 2AFR 2AFS 2AFT 2AFU 2AFV 2AFW 2AFX 2AFY 2AFZ 2AG0 2AG1 2AG2 2AG3 2AG4 2AG5 2AG6 2AG8 2AG9 2AGC 2AGD 2AGE 2AGG 2AGI 2AGJ 2AGK 2AGL 2AGS 2AGT 2AGV 2AGW 2AGX 2AGY 2AGZ 2AH0 2AH1 2AH2 2AH4 2AH5 2AH6 2AH7 2AH8 2AH9 2AHA 2AHB 2AHC 2AHD 2AHE 2AHF 2AHG 2AHJ 2AHK 2AHL 2AHM 2AHN 2AHO 2AHP 2AHR 2AHS 2AHU 2AHV 2AHW 2AHX 2AHY 2AHZ 2AI0 2AI1 2AI2 2AI3 2AI7 2AI8 2AI9 2AIA 2AIB 2AID 2AIE 2AIF 2AIG 2AII 2AIJ 2AIK 2AIM 2AIO 2AIP 2AIQ 2AIR 2AIU 2AIX 2AJ2 2AJ3 2AJ4 2AJ6 2AJ7 2AJ8 2AJ9 2AJA 2AJB 2AJC 2AJD 2AJF 2AJG 2AJH 2AJI 2AJL 2AJP 2AJR 2AJS 2AJT 2AJU 2AJV 2AJX 2AJY 2AJZ 2AK1 2AK2 2AK3 2AK4 2AK5 2AK7 2AKA 2AKC 2AKF 2AKJ 2AKM 2AKO 2AKP 2AKQ 2AKR 2AKW 2AKY 2AKZ 2AL0 2AL1 2AL2 2AL4 2AL5 2AL6 2AL7 2ALA 2ALD 2ALE 2ALF 2ALG 2ALL 2ALM 2ALP 2ALR 2ALU 2ALV 2ALW 2ALX 2ALY 2AM1 2AM2 2AM3 2AM4 2AM5 2AM9 2AMA 2AMB 2AMC 2AMD 2AME 2AMF 2AMG 2AMH 2AMJ 2AML 2AMM 2AMO 2AMP 2AMQ 2AMS 2AMT 2AMU 2AMV 2AMX 2AMY 2AN0 2AN1 2AN2 2AN3 2AN4 2AN5 2AN6 2AN9 2ANB 2ANC 2ANE 2ANG 2ANH 2ANI 2ANJ 2ANK 2ANL 2ANM 2ANO 2ANP 2ANQ 2ANS 2ANT 2ANU 2ANV 2ANW 2ANX 2ANY 2ANZ 2AO2 2AO6 2AO7 2AO9 2AOA 2AOB 2AOC 2AOD 2AOE 2AOF 2AOG 2AOH 2AOI 2AOJ 2AOP 2AOS 2AOT 2AOU 2AOV 2AOW 2AOX 2AOZ 2AP1 2AP2 2AP3 2AP6 2AP9 2APB 2APC 2APF 2APG 2APH 2APJ 2APL 2APO 2APQ 2APR 2APS 2APT 2APV 2APW 2APX 2AQ1 2AQ2 2AQ3 2AQ5 2AQ6 2AQ7 2AQ8 2AQ9 2AQB 2AQD 2AQH 2AQI 2AQJ 2AQK 2AQL 2AQN 2AQO 2AQP 2AQQ 2AQR 2AQS 2AQT 2AQU 2AQV 2AQW 2AQX 2AQZ 2AR0 2AR1 2AR3 2AR5 2AR6 2AR7 2AR8 2AR9 2ARA 2ARB 2ARC 2ARD 2ARE 2ARH 2ARJ 2ARK 2ARL 2ARM 2ARO 2ARP 2ARQ 2ARR 2ARS 2ART 2ARU 2ARV 2ARX 2ARY 2ARZ 2AS0 2AS1 2AS2 2AS3 2AS4 2AS6 2AS8 2AS9 2ASC 2ASF 2ASH 2ASI 2ASK 2ASM 2ASN 2ASO 2ASP 2ASR 2ASS 2AST 2ASU 2ASV 2AT0 2AT1 2AT2 2AT3 2AT5 2AT6 2AT8 2ATB 2ATC 2ATE 2ATF 2ATH 2ATI 2ATJ 2ATK 2ATM 2ATO 2ATP 2ATQ 2ATR 2ATS 2ATV 2ATX 2ATZ 2AU1 2AU3 2AU5 2AU6 2AU7 2AU8 2AU9 2AUA 2AUB 2AUC 2AUD 2AUG 2AUH 2AUJ 2AUK 2AUM 2AUN 2AUO 2AUP 2AUQ 2AUR 2AUS 2AUT 2AUU 2AUW 2AUX 2AUY 2AUZ 2AV0 2AV1 2AV3 2AV4 2AV5 2AV6 2AV7 2AV8 2AV9 2AVD 2AVF 2AVI 2AVK 2AVM 2AVN 2AVO 2AVP 2AVQ 2AVS 2AVT 2AVU 2AVV 2AVW 2AW1 2AW2 2AW3 2AW5 2AW6 2AW9 2AWA 2AWC 2AWD 2AWF 2AWG 2AWH 2AWI 2AWJ 2AWK 2AWL 2AWM 2AWN 2AWO 2AWP 2AWU 2AWW 2AWX 2AWY 2AWZ 2AX0 2AX1 2AX2 2AX3 2AX4 2AX6 2AX7 2AX8 2AX9 2AXA 2AXC 2AXE 2AXF 2AXG 2AXH 2AXI 2AXJ 2AXM 2AXN 2AXO 2AXP 2AXQ 2AXR 2AXT 2AXU 2AXV 2AXW 2AXZ 2AY0 2AY1 2AY2 2AY3 2AY4 2AY5 2AY6 2AY7 2AY8 2AY9 2AYD 2AYE 2AYH 2AYI 2AYL 2AYN 2AYO 2AYP 2AYQ 2AYR 2AYS 2AYT 2AYU 2AYV 2AYW 2AZ1 2AZ3 2AZ4 2AZ5 2AZ8 2AZ9 2AZA 2AZB 2AZC 2AZD 2AZE 2AZJ 2AZK 2AZL 2AZM 2AZN 2AZO 2AZP 2AZQ 2AZR 2AZT 2AZU 2AZW 2AZY 2AZZ 2B00 2B01 2B02 2B03 2B04 2B05 2B06 2B07 2B08 2B0A 2B0C 2B0J 2B0L 2B0M 2B0O 2B0P 2B0Q 2B0R 2B0S 2B0T 2B0U 2B0V 2B0Z 2B10 2B11 2B12 2B13 2B14 2B15 2B16 2B17 2B18 2B1A 2B1E 2B1F 2B1G 2B1H 2B1I 2B1J 2B1K 2B1L 2B1M 2B1N 2B1P 2B1Q 2B1R 2B1V 2B1X 2B1Y 2B1Z 2B20 2B21 2B22 2B23 2B24 2B25 2B26 2B29 2B2A 2B2C 2B2F 2B2H 2B2I 2B2J 2B2K 2B2N 2B2O 2B2Q 2B2R 2B2S 2B2T 2B2U 2B2V 2B2W 2B2X 2B2Y 2B30 2B31 2B33 2B34 2B35 2B36 2B37 2B39 2B3B 2B3D 2B3F 2B3G 2B3H 2B3K 2B3L 2B3M 2B3O 2B3P 2B3Q 2B3R 2B3S 2B3T 2B3U 2B3V 2B3X 2B3Y 2B3Z 2B42 2B43 2B44 2B45 2B46 2B48 2B49 2B4A 2B4B 2B4C 2B4D 2B4E 2B4F 2B4G 2B4H 2B4I 2B4J 2B4K 2B4L 2B4M 2B4O 2B4P 2B4Q 2B4R 2B4S 2B4T 2B4U 2B4V 2B4W 2B4X 2B4Y 2B4Z 2B50 2B51 2B52 2B53 2B54 2B55 2B56 2B58 2B59 2B5A 2B5D 2B5E 2B5F 2B5G 2B5H 2B5I 2B5J 2B5L 2B5M 2B5N 2B5O 2B5R 2B5S 2B5T 2B5U 2B5V 2B5W 2B5Z 2B60 2B61 2B65 2B67 2B69 2B6A 2B6C 2B6E 2B6H 2B6M 2B6N 2B6P 2B6T 2B6W 2B6X 2B6Y 2B6Z 2B70 2B71 2B72 2B73 2B74 2B75 2B76 2B77 2B78 2B7A 2B7B 2B7C 2B7D 2B7F 2B7H 2B7J 2B7K 2B7L 2B7M 2B7N 2B7O 2B7P 2B7Q 2B7R 2B7S 2B7U 2B7X 2B7Y 2B7Z 2B81 2B82 2B83 2B8E 2B8H 2B8I 2B8J 2B8K 2B8L 2B8M 2B8N 2B8O 2B8P 2B8Q 2B8T 2B8U 2B8V 2B8W 2B8X 2B8Y 2B8Z 2B90 2B91 2B92 2B94 2B96 2B97 2B98 2B99 2B9A 2B9B 2B9C 2B9D 2B9E 2B9F 2B9H 2B9I 2B9J 2B9L 2B9R 2B9U 2B9V 2B9W 2B9X 2B9Y 2BA0 2BA1 2BA2 2BA9 2BAA 2BAB 2BAC 2BAG 2BAJ 2BAK 2BAL 2BAN 2BAP 2BAQ 2BAS 2BAT 2BAW 2BAX 2BAY 2BAZ 2BB0 2BB2 2BB3 2BB4 2BB5 2BB6 2BB7 2BB9 2BBA 2BBB 2BBC 2BBD 2BBE 2BBF 2BBH 2BBJ 2BBK 2BBO 2BBQ 2BBR 2BBS 2BBT 2BBW 2BBZ 2BC0 2BC1 2BC2 2BC3 2BC4 2BC5 2BC9 2BCC 2BCD 2BCE 2BCG 2BCH 2BCJ 2BCK 2BCM 2BCN 2BCO 2BCP 2BCT 2BCX 2BD0 2BD1 2BD2 2BD3 2BD4 2BD5 2BD7 2BD8 2BD9 2BDA 2BDB 2BDC 2BDD 2BDE 2BDF 2BDG 2BDH 2BDI 2BDJ 2BDL 2BDM 2BDN 2BDQ 2BDR 2BDT 2BDU 2BDV 2BDW 2BDX 2BDY 2BDZ 2BE1 2BE2 2BE3 2BE4 2BE5 2BE6 2BE7 2BE9 2BEA 2BEB 2BEC 2BED 2BEF 2BEH 2BEI 2BEJ 2BEK 2BEL 2BEM 2BEN 2BEO 2BEP 2BEQ 2BER 2BES 2BET 2BEU 2BEV 2BEW 2BEX 2BEZ 2BF0 2BF1 2BF2 2BF3 2BF4 2BF5 2BF6 2BF7 2BF8 2BF9 2BFA 2BFB 2BFC 2BFD 2BFE 2BFF 2BFG 2BFH 2BFI 2BFK 2BFL 2BFM 2BFN 2BFO 2BFP 2BFQ 2BFR 2BFU 2BFV 2BFW 2BFX 2BFY 2BFZ 2BG1 2BG2 2BG5 2BG6 2BG7 2BG8 2BGA 2BGC 2BGD 2BGE 2BGH 2BGI 2BGJ 2BGK 2BGL 2BGM 2BGN 2BGQ 2BGR 2BGS 2BGT 2BGU 2BGV 2BH0 2BH1 2BH3 2BH4 2BH5 2BH7 2BH8 2BH9 2BHA 2BHB 2BHC 2BHD 2BHE 2BHF 2BHG 2BHH 2BHI 2BHJ 2BHK 2BHL 2BHM 2BHN 2BHO 2BHP 2BHQ 2BHR 2BHS 2BHT 2BHU 2BHV 2BHW 2BHX 2BHY 2BHZ 2BI0 2BI1 2BI2 2BI3 2BI4 2BI5 2BI7 2BI8 2BI9 2BIA 2BIB 2BIE 2BIF 2BIG 2BIH 2BII 2BIJ 2BIK 2BIL 2BIM 2BIN 2BIO 2BIP 2BIQ 2BIR 2BIS 2BIT 2BIU 2BIV 2BIW 2BIX 2BIY 2BJ0 2BJ1 2BJ3 2BJ4 2BJ7 2BJ8 2BJ9 2BJA 2BJB 2BJD 2BJE 2BJF 2BJG 2BJH 2BJI 2BJJ 2BJK 2BJM 2BJN 2BJO 2BJQ 2BJR 2BJS 2BJU 2BJV 2BJW 2BJY 2BK0 2BK3 2BK4 2BK5 2BK6 2BK8 2BK9 2BKA 2BKB 2BKC 2BKE 2BKF 2BKG 2BKH 2BKI 2BKJ 2BKK 2BKL 2BKM 2BKN 2BKO 2BKP 2BKQ 2BKR 2BKS 2BKT 2BKU 2BKV 2BKW 2BKX 2BKY 2BKZ 2BL0 2BL1 2BL2 2BL4 2BL7 2BL8 2BL9 2BLA 2BLB 2BLC 2BLE 2BLF 2BLG 2BLH 2BLI 2BLJ 2BLL 2BLM 2BLN 2BLO 2BLP 2BLQ 2BLR 2BLS 2BLU 2BLV 2BLW 2BLX 2BLY 2BLZ 2BM0 2BM1 2BM2 2BM3 2BM4 2BM5 2BM6 2BM7 2BM8 2BM9 2BMA 2BMB 2BMC 2BMD 2BME 2BMF 2BMG 2BMH 2BMI 2BMJ 2BMK 2BML 2BMM 2BMO 2BMQ 2BMR 2BMU 2BMV 2BMW 2BMX 2BMY 2BMZ 2BN0 2BN1 2BN2 2BN3 2BN4 2BN7 2BND 2BNE 2BNF 2BNG 2BNH 2BNI 2BNJ 2BNK 2BNL 2BNM 2BNN 2BNO 2BNP 2BNQ 2BNR 2BNS 2BNU 2BNX 2BO0 2BO1 2BO2 2BO3 2BO4 2BO6 2BO7 2BO8 2BO9 2BOA 2BOB 2BOC 2BOD 2BOE 2BOF 2BOG 2BOH 2BOI 2BOJ 2BOK 2BOL 2BON 2BOO 2BOQ 2BOS 2BOU 2BOV 2BOW 2BOX 2BOY 2BOZ 2BP0 2BP1 2BP2 2BP3 2BP5 2BP6 2BP7 2BP8 2BPB 2BPC 2BPD 2BPE 2BPH 2BPI 2BPM 2BPO 2BPP 2BPQ 2BPS 2BPT 2BPU 2BPV 2BPW 2BPX 2BPY 2BPZ 2BQ0 2BQ1 2BQ4 2BQ6 2BQ7 2BQ8 2BQA 2BQB 2BQC 2BQD 2BQE 2BQF 2BQG 2BQH 2BQI 2BQJ 2BQK 2BQL 2BQM 2BQN 2BQO 2BQP 2BQQ 2BQV 2BQW 2BQX 2BQY 2BQZ 2BR1 2BR2 2BR3 2BR4 2BR5 2BR6 2BR7 2BR8 2BR9 2BRA 2BRB 2BRC 2BRE 2BRF 2BRG 2BRH 2BRI 2BRJ 2BRK 2BRL 2BRM 2BRN 2BRO 2BRP 2BRQ 2BRR 2BRS 2BRT 2BRV 2BRW 2BRX 2BRY 2BS2 2BS3 2BS4 2BS5 2BS6 2BS7 2BS8 2BS9 2BSA 2BSB 2BSC 2BSD 2BSE 2BSF 2BSH 2BSI 2BSJ 2BSK 2BSL 2BSM 2BSP 2BSR 2BSS 2BST 2BSW 2BSX 2BSY 2BSZ 2BT0 2BT1 2BT2 2BT3 2BT4 2BT6 2BT7 2BT8 2BT9 2BTC 2BTD 2BTF 2BTI 2BTJ 2BTL 2BTM 2BTN 2BTO 2BTP 2BTQ 2BTR 2BTS 2BTU 2BTV 2BTW 2BTY 2BTZ 2BU2 2BU3 2BU4 2BU5 2BU6 2BU7 2BU8 2BU9 2BUA 2BUB 2BUC 2BUE 2BUF 2BUH 2BUI 2BUJ 2BUK 2BUM 2BUO 2BUP 2BUQ 2BUR 2BUT 2BUU 2BUV 2BUW 2BUX 2BUY 2BUZ 2BV0 2BV1 2BV2 2BV3 2BV4 2BV5 2BV6 2BV7 2BV8 2BV9 2BVA 2BVC 2BVD 2BVE 2BVF 2BVG 2BVH 2BVJ 2BVL 2BVM 2BVN 2BVO 2BVP 2BVQ 2BVR 2BVS 2BVT 2BVU 2BVV 2BVW 2BVX 2BVY 2BVZ 2BW0 2BW1 2BW3 2BW4 2BW5 2BW7 2BW8 2BW9 2BWA 2BWB 2BWC 2BWD 2BWE 2BWF 2BWG 2BWH 2BWI 2BWJ 2BWK 2BWL 2BWM 2BWN 2BWO 2BWP 2BWQ 2BWR 2BWS 2BWT 2BWU 2BWV 2BWW 2BWX 2BWY 2BX3 2BX4 2BX5 2BX6 2BX7 2BX8 2BX9 2BXA 2BXB 2BXC 2BXD 2BXE 2BXF 2BXG 2BXH 2BXI 2BXJ 2BXK 2BXL 2BXM 2BXN 2BXO 2BXP 2BXQ 2BXR 2BXS 2BXT 2BXU 2BXV 2BXW 2BXX 2BXY 2BXZ 2BY0 2BY1 2BY2 2BY3 2BY4 2BY5 2BY6 2BY7 2BY8 2BY9 2BYA 2BYB 2BYC 2BYD 2BYG 2BYH 2BYI 2BYJ 2BYK 2BYL 2BYM 2BYN 2BYO 2BYP 2BYQ 2BYR 2BYS 2BYV 2BYW 2BYX 2BYY 2BYZ 2BZ0 2BZ1 2BZ3 2BZ4 2BZ5 2BZ6 2BZ7 2BZ8 2BZ9 2BZA 2BZC 2BZD 2BZG 2BZH 2BZI 2BZJ 2BZK 2BZL 2BZN 2BZR 2BZS 2BZU 2BZV 2BZW 2BZX 2BZY 2BZZ 2C00 2C01 2C02 2C03 2C04 2C05 2C07 2C08 2C0A 2C0C 2C0D 2C0E 2C0F 2C0G 2C0H 2C0I 2C0J 2C0K 2C0L 2C0M 2C0N 2C0O 2C0P 2C0Q 2C0R 2C0T 2C0U 2C0Y 2C0Z 2C10 2C11 2C12 2C13 2C14 2C15 2C16 2C18 2C19 2C1A 2C1B 2C1C 2C1D 2C1E 2C1F 2C1G 2C1H 2C1I 2C1J 2C1L 2C1M 2C1N 2C1O 2C1P 2C1Q 2C1S 2C1T 2C1U 2C1V 2C1W 2C1X 2C1Y 2C1Z 2C20 2C21 2C23 2C24 2C25 2C26 2C27 2C29 2C2A 2C2B 2C2C 2C2F 2C2G 2C2H 2C2I 2C2J 2C2K 2C2L 2C2M 2C2N 2C2O 2C2P 2C2Q 2C2S 2C2T 2C2U 2C2V 2C2W 2C2X 2C2Y 2C2Z 2C30 2C31 2C32 2C35 2C36 2C37 2C38 2C39 2C3A 2C3B 2C3C 2C3D 2C3E 2C3F 2C3G 2C3H 2C3I 2C3J 2C3K 2C3L 2C3M 2C3N 2C3O 2C3P 2C3Q 2C3S 2C3T 2C3U 2C3V 2C3W 2C3X 2C3Y 2C3Z 2C40 2C41 2C42 2C43 2C44 2C45 2C46 2C47 2C49 2C4A 2C4B 2C4C 2C4D 2C4E 2C4F 2C4G 2C4H 2C4I 2C4J 2C4K 2C4L 2C4M 2C4N 2C4P 2C4T 2C4U 2C4V 2C4W 2C4X 2C53 2C54 2C56 2C57 2C58 2C59 2C5A 2C5B 2C5C 2C5D 2C5E 2C5F 2C5G 2C5H 2C5I 2C5J 2C5K 2C5L 2C5N 2C5O 2C5Q 2C5S 2C5U 2C5V 2C5W 2C5X 2C5Y 2C60 2C61 2C63 2C64 2C65 2C66 2C67 2C68 2C69 2C6C 2C6D 2C6E 2C6F 2C6G 2C6H 2C6I 2C6J 2C6K 2C6L 2C6M 2C6N 2C6O 2C6P 2C6Q 2C6R 2C6S 2C6T 2C6U 2C6W 2C6X 2C6Z 2C70 2C71 2C72 2C73 2C74 2C75 2C76 2C77 2C78 2C79 2C7B 2C7F 2C7G 2C7I 2C7J 2C7K 2C7L 2C7M 2C7N 2C7S 2C7T 2C7U 2C7V 2C7W 2C7X 2C7Y 2C7Z 2C80 2C81 2C82 2C83 2C84 2C86 2C88 2C89 2C8A 2C8B 2C8C 2C8D 2C8E 2C8F 2C8G 2C8H 2C8J 2C8K 2C8L 2C8M 2C8N 2C8O 2C8P 2C8Q 2C8R 2C8S 2C8T 2C8U 2C8V 2C8W 2C8X 2C8Y 2C8Z 2C90 2C91 2C92 2C93 2C94 2C95 2C96 2C97 2C98 2C99 2C9A 2C9B 2C9C 2C9D 2C9E 2C9H 2C9I 2C9J 2C9K 2C9M 2C9O 2C9P 2C9Q 2C9R 2C9S 2C9T 2C9U 2C9V 2C9W 2C9X 2C9Y 2C9Z 2CA0 2CA1 2CA2 2CA3 2CA4 2CA5 2CA6 2CA8 2CA9 2CAB 2CAD 2CAG 2CAH 2CAI 2CAJ 2CAK 2CAL 2CAM 2CAN 2CAQ 2CAR 2CAS 2CAU 2CAV 2CAY 2CAZ 2CB0 2CB1 2CB2 2CB3 2CB4 2CB5 2CB6 2CB8 2CB9 2CBA 2CBB 2CBC 2CBD 2CBE 2CBF 2CBG 2CBI 2CBJ 2CBL 2CBM 2CBN 2CBO 2CBP 2CBQ 2CBR 2CBS 2CBT 2CBU 2CBV 2CBX 2CBY 2CBZ 2CC0 2CC1 2CC2 2CC3 2CC6 2CC7 2CC8 2CC9 2CCA 2CCB 2CCC 2CCD 2CCE 2CCF 2CCG 2CCH 2CCI 2CCJ 2CCK 2CCL 2CCM 2CCN 2CCP 2CCQ 2CCR 2CCS 2CCT 2CCU 2CCV 2CCW 2CCY 2CD0 2CD2 2CD7 2CD8 2CD9 2CDA 2CDB 2CDC 2CDE 2CDF 2CDG 2CDH 2CDN 2CDO 2CDP 2CDQ 2CDR 2CDS 2CDT 2CDU 2CDV 2CDY 2CDZ 2CE0 2CE1 2CE2 2CE3 2CE4 2CE6 2CE7 2CE8 2CE9 2CEA 2CEI 2CEJ 2CEK 2CEL 2CEM 2CEN 2CEO 2CEP 2CEQ 2CER 2CES 2CET 2CEU 2CEV 2CEX 2CEY 2CF2 2CF4 2CF5 2CF6 2CF7 2CF8 2CF9 2CFA 2CFB 2CFC 2CFD 2CFE 2CFF 2CFG 2CFH 2CFI 2CFK 2CFL 2CFM 2CFO 2CFP 2CFQ 2CFR 2CFS 2CFT 2CFU 2CFV 2CFW 2CFX 2CFY 2CFZ 2CG0 2CG1 2CG2 2CG3 2CG4 2CG5 2CG6 2CG7 2CG8 2CG9 2CGA 2CGE 2CGF 2CGH 2CGI 2CGJ 2CGK 2CGL 2CGN 2CGO 2CGQ 2CGR 2CGU 2CGV 2CGW 2CGX 2CGY 2CGZ 2CH1 2CH2 2CH4 2CH5 2CH6 2CH7 2CH8 2CH9 2CHA 2CHB 2CHC 2CHD 2CHE 2CHF 2CHG 2CHH 2CHI 2CHL 2CHM 2CHN 2CHO 2CHP 2CHQ 2CHR 2CHS 2CHT 2CHU 2CHV 2CHW 2CHX 2CHY 2CHZ 2CI0 2CI1 2CI2 2CI3 2CI4 2CI5 2CI6 2CI7 2CI8 2CI9 2CIA 2CIB 2CIC 2CIE 2CIF 2CIG 2CIH 2CII 2CIJ 2CIK 2CIM 2CIN 2CIO 2CIP 2CIQ 2CIR 2CIS 2CIT 2CIU 2CIV 2CIW 2CIX 2CIY 2CIZ 2CJ0 2CJ1 2CJ2 2CJ3 2CJ4 2CJ5 2CJ6 2CJ7 2CJ8 2CJ9 2CJA 2CJB 2CJC 2CJD 2CJE 2CJF 2CJG 2CJH 2CJI 2CJJ 2CJL 2CJM 2CJP 2CJQ 2CJR 2CJS 2CJT 2CJU 2CJW 2CJX 2CJY 2CJZ 2CK0 2CK1 2CK2 2CK3 2CKB 2CKD 2CKE 2CKF 2CKG 2CKH 2CKI 2CKJ 2CKK 2CKL 2CKM 2CKO 2CKP 2CKQ 2CKR 2CKS 2CKW 2CKX 2CKZ 2CL0 2CL2 2CL3 2CL4 2CL5 2CL6 2CL7 2CL8 2CLA 2CLB 2CLC 2CLD 2CLE 2CLF 2CLH 2CLI 2CLK 2CLL 2CLM 2CLO 2CLP 2CLQ 2CLR 2CLS 2CLT 2CLU 2CLV 2CLW 2CLX 2CLY 2CLZ 2CM0 2CM1 2CM2 2CM3 2CM4 2CM5 2CM6 2CM7 2CM8 2CM9 2CMA 2CMB 2CMC 2CMD 2CME 2CMF 2CMG 2CMH 2CMJ 2CMK 2CML 2CMM 2CMN 2CMO 2CMP 2CMR 2CMT 2CMU 2CMV 2CMW 2CMY 2CMZ 2CN0 2CN1 2CN2 2CN3 2CN4 2CN5 2CN6 2CN7 2CN8 2CNA 2CNB 2CNC 2CND 2CNE 2CNF 2CNG 2CNH 2CNI 2CNK 2CNL 2CNM 2CNN 2CNO 2CNQ 2CNS 2CNT 2CNU 2CNV 2CNW 2CNX 2CNY 2CNZ 2CO0 2CO1 2CO2 2CO3 2CO4 2CO5 2CO6 2CO7 2COG 2COI 2COJ 2COL 2COQ 2COV 2CP4 2CPG 2CPK 2CPL 2CPO 2CPP 2CPU 2CQS 2CQT 2CQZ 2CRK 2CRO 2CS7 2CSB 2CSC 2CSD 2CSG 2CSL 2CSM 2CSN 2CST 2CSU 2CT9 2CTB 2CTC 2CTH 2CTS 2CTV 2CTX 2CTZ 2CU0 2CU2 2CU3 2CU5 2CU6 2CU9 2CUA 2CUK 2CUL 2CUN 2CUO 2CUT 2CUU 2CUW 2CUY 2CUZ 2CV3 2CV4 2CV6 2CV8 2CV9 2CVB 2CVC 2CVD 2CVE 2CVF 2CVH 2CVI 2CVJ 2CVK 2CVL 2CVO 2CVP 2CVQ 2CVS 2CVT 2CVU 2CVV 2CVW 2CVX 2CVY 2CVZ 2CW0 2CW2 2CW3 2CW4 2CW5 2CW6 2CW7 2CW8 2CW9 2CWA 2CWC 2CWD 2CWE 2CWF 2CWG 2CWH 2CWI 2CWJ 2CWK 2CWL 2CWM 2CWN 2CWO 2CWP 2CWQ 2CWR 2CWS 2CWT 2CWU 2CWV 2CWW 2CWX 2CWY 2CWZ 2CX0 2CX1 2CX3 2CX4 2CX5 2CX6 2CX7 2CX8 2CX9 2CXA 2CXB 2CXC 2CXD 2CXE 2CXF 2CXG 2CXH 2CXI 2CXK 2CXL 2CXN 2CXO 2CXP 2CXQ 2CXR 2CXS 2CXT 2CXU 2CXV 2CXX 2CXY 2CY0 2CY1 2CY2 2CY3 2CY4 2CY5 2CY6 2CY7 2CY8 2CY9 2CYA 2CYB 2CYC 2CYD 2CYE 2CYF 2CYG 2CYH 2CYJ 2CYM 2CYP 2CYX 2CYY 2CYZ 2CZ0 2CZ1 2CZ2 2CZ3 2CZ4 2CZ5 2CZ6 2CZ7 2CZ8 2CZ9 2CZC 2CZD 2CZE 2CZF 2CZG 2CZH 2CZI 2CZK 2CZL 2CZQ 2CZR 2CZS 2CZT 2CZU 2CZV 2CZW 2D00 2D01 2D02 2D03 2D04 2D05 2D06 2D07 2D09 2D0A 2D0B 2D0C 2D0D 2D0E 2D0F 2D0G 2D0H 2D0I 2D0J 2D0K 2D0N 2D0O 2D0P 2D0Q 2D0S 2D0T 2D0U 2D0V 2D0W 2D10 2D11 2D13 2D16 2D1C 2D1E 2D1F 2D1G 2D1H 2D1I 2D1J 2D1K 2D1L 2D1N 2D1O 2D1P 2D1Q 2D1R 2D1S 2D1T 2D1V 2D1W 2D1X 2D1Y 2D1Z 2D20 2D22 2D23 2D24 2D26 2D27 2D28 2D29 2D2A 2D2C 2D2D 2D2E 2D2F 2D2G 2D2H 2D2I 2D2J 2D2M 2D2N 2D2O 2D2Q 2D2R 2D2S 2D2V 2D2X 2D2Z 2D30 2D31 2D32 2D33 2D36 2D37 2D38 2D39 2D3A 2D3B 2D3C 2D3D 2D3E 2D3F 2D3G 2D3H 2D3I 2D3K 2D3L 2D3M 2D3N 2D3P 2D3Q 2D3R 2D3S 2D3T 2D3U 2D3V 2D3W 2D3Y 2D3Z 2D40 2D41 2D42 2D43 2D44 2D48 2D4A 2D4C 2D4D 2D4E 2D4F 2D4G 2D4H 2D4I 2D4J 2D4K 2D4L 2D4M 2D4N 2D4O 2D4P 2D4Q 2D4R 2D4U 2D4V 2D4W 2D4X 2D4Y 2D4Z 2D51 2D52 2D54 2D58 2D59 2D5A 2D5B 2D5C 2D5D 2D5F 2D5G 2D5H 2D5I 2D5J 2D5K 2D5L 2D5M 2D5N 2D5R 2D5W 2D5X 2D5Y 2D5Z 2D60 2D61 2D62 2D63 2D64 2D65 2D66 2D68 2D69 2D6B 2D6C 2D6K 2D6L 2D6M 2D6N 2D6O 2D6P 2D6Y 2D73 2D74 2D7C 2D7E 2D7F 2D7G 2D7H 2D7I 2D7J 2D7R 2D7S 2D7T 2D7U 2D7V 2D7Y 2D7Z 2D80 2D81 2D8A 2D8D 2D8E 2D8L 2D8N 2D8O 2D8P 2D8W 2D91 2D97 2D98 2D9Q 2D9R 2DAA 2DAB 2DAP 2DB0 2DB4 2DB7 2DBB 2DBI 2DBL 2DBN 2DBO 2DBQ 2DBR 2DBS 2DBT 2DBU 2DBV 2DBW 2DBX 2DBY 2DBZ 2DC0 2DC1 2DC3 2DC4 2DC5 2DC6 2DC7 2DC8 2DC9 2DCA 2DCB 2DCC 2DCD 2DCF 2DCH 2DCJ 2DCK 2DCL 2DCM 2DCN 2DCT 2DCU 2DCY 2DCZ 2DD4 2DD5 2DD7 2DD8 2DD9 2DDA 2DDB 2DDC 2DDD 2DDF 2DDH 2DDK 2DDM 2DDO 2DDQ 2DDR 2DDS 2DDT 2DDU 2DDW 2DDX 2DDZ 2DE0 2DE2 2DE3 2DE4 2DE5 2DE6 2DE7 2DE8 2DE9 2DEA 2DEB 2DEC 2DEG 2DEH 2DEI 2DEJ 2DEK 2DEO 2DEP 2DEQ 2DEV 2DEW 2DEX 2DEY 2DF3 2DF4 2DF5 2DF6 2DF7 2DF8 2DFA 2DFB 2DFC 2DFD 2DFE 2DFF 2DFH 2DFI 2DFJ 2DFK 2DFL 2DFN 2DFP 2DFT 2DFU 2DFV 2DFX 2DFY 2DG0 2DG1 2DG2 2DG3 2DG4 2DG5 2DG6 2DG7 2DG8 2DG9 2DGA 2DGB 2DGD 2DGE 2DGJ 2DGK 2DGL 2DGM 2DGN 2DH1 2DH2 2DH3 2DH4 2DH5 2DH6 2DHB 2DHC 2DHD 2DHE 2DHF 2DHH 2DHN 2DHO 2DHQ 2DHR 2DHT 2DI3 2DI4 2DIE 2DIJ 2DIK 2DIO 2DJ5 2DJ6 2DJF 2DJG 2DJH 2DJI 2DJL 2DJW 2DJX 2DJZ 2DKA 2DKB 2DKC 2DKD 2DKE 2DKF 2DKG 2DKH 2DKI 2DKJ 2DKK 2DKN 2DKO 2DKV 2DL2 2DLA 2DLB 2DLD 2DLF 2DLI 2DLN 2DM5 2DM6 2DM9 2DMA 2DMR 2DN1 2DN2 2DN3 2DNS 2DO2 2DOB 2DOH 2DOI 2DOJ 2DOK 2DOO 2DOQ 2DOR 2DOU 2DP3 2DP4 2DP5 2DP8 2DP9 2DPE 2DPF 2DPG 2DPH 2DPK 2DPL 2DPM 2DPN 2DPP 2DPQ 2DPR 2DPS 2DPT 2DPW 2DPX 2DPY 2DPZ 2DQ0 2DQ3 2DQ4 2DQ6 2DQ7 2DQA 2DQB 2DQC 2DQD 2DQE 2DQF 2DQG 2DQH 2DQI 2DQJ 2DQK 2DQL 2DQM 2DQN 2DQR 2DQS 2DQT 2DQU 2DQV 2DQW 2DQX 2DQY 2DQZ 2DR0 2DR1 2DR3 2DR6 2DRC 2DRD 2DRE 2DRH 2DRI 2DRJ 2DRK 2DRM 2DRO 2DRQ 2DRR 2DRS 2DRT 2DRU 2DRV 2DRW 2DRX 2DRY 2DRZ 2DS0 2DS1 2DS2 2DS5 2DS6 2DS7 2DS8 2DS9 2DSA 2DSB 2DSC 2DSD 2DSF 2DSG 2DSH 2DSI 2DSJ 2DSK 2DSL 2DSN 2DSO 2DSP 2DSQ 2DSR 2DST 2DSU 2DSV 2DSW 2DSX 2DSY 2DSZ 2DT0 2DT1 2DT2 2DT3 2DT4 2DT5 2DT8 2DT9 2DTC 2DTD 2DTE 2DTG 2DTH 2DTI 2DTJ 2DTM 2DTN 2DTO 2DTR 2DTS 2DTT 2DTW 2DTX 2DTY 2DTZ 2DU0 2DU1 2DU2 2DU7 2DU8 2DU9 2DUA 2DUB 2DUC 2DUD 2DUE 2DUF 2DUG 2DUH 2DUI 2DUJ 2DUK 2DUL 2DUM 2DUO 2DUP 2DUQ 2DUR 2DUT 2DUU 2DUV 2DUX 2DUY 2DUZ 2DV0 2DV1 2DV2 2DV3 2DV4 2DV5 2DV6 2DV7 2DV9 2DVA 2DVB 2DVC 2DVD 2DVE 2DVF 2DVG 2DVK 2DVL 2DVM 2DVN 2DVO 2DVP 2DVQ 2DVR 2DVS 2DVT 2DVU 2DVV 2DVW 2DVX 2DVY 2DVZ 2DW0 2DW1 2DW2 2DW4 2DW5 2DW6 2DW7 2DWA 2DWB 2DWC 2DWD 2DWE 2DWG 2DWH 2DWI 2DWJ 2DWK 2DWO 2DWP 2DWQ 2DWR 2DWS 2DWT 2DWU 2DWW 2DWX 2DWY 2DWZ 2DX0 2DX1 2DX5 2DX6 2DX7 2DX8 2DXA 2DXB 2DXC 2DXD 2DXE 2DXF 2DXL 2DXN 2DXP 2DXQ 2DXR 2DXS 2DXT 2DXU 2DXV 2DXW 2DXX 2DXY 2DY0 2DY1 2DY2 2DY3 2DY5 2DY9 2DYA 2DYB 2DYC 2DYH 2DYI 2DYJ 2DYK 2DYL 2DYM 2DYN 2DYO 2DYP 2DYQ 2DYR 2DYS 2DYT 2DYU 2DYV 2DYX 2DYY 2DZ9 2DZA 2DZB 2DZC 2DZD 2DZE 2DZN 2DZO 2DZP 2DZS 2DZT 2DZU 2DZV 2DZW 2DZX 2DZY 2DZZ 2E00 2E01 2E02 2E03 2E07 2E08 2E09 2E0A 2E0C 2E0I 2E0J 2E0K 2E0L 2E0M 2E0N 2E0O 2E0P 2E0Q 2E0S 2E0T 2E0W 2E0X 2E0Y 2E0Z 2E10 2E11 2E12 2E15 2E16 2E17 2E18 2E1A 2E1B 2E1D 2E1E 2E1F 2E1H 2E1M 2E1N 2E1P 2E1Q 2E1R 2E1S 2E1T 2E1U 2E1V 2E1W 2E1Y 2E1Z 2E20 2E21 2E22 2E24 2E25 2E26 2E27 2E28 2E2A 2E2B 2E2C 2E2D 2E2E 2E2G 2E2K 2E2L 2E2M 2E2N 2E2O 2E2P 2E2Q 2E2R 2E2T 2E2U 2E2V 2E2X 2E2Y 2E31 2E32 2E33 2E37 2E39 2E3A 2E3B 2E3C 2E3D 2E3H 2E3I 2E3J 2E3K 2E3M 2E3N 2E3O 2E3P 2E3Q 2E3R 2E3S 2E3T 2E3U 2E3V 2E3W 2E3X 2E3Z 2E40 2E41 2E46 2E47 2E48 2E49 2E4A 2E4F 2E4G 2E4L 2E4M 2E4N 2E4O 2E4P 2E4Q 2E4R 2E4T 2E4U 2E4V 2E4W 2E4X 2E4Y 2E4Z 2E50 2E51 2E53 2E54 2E55 2E56 2E59 2E5A 2E5B 2E5C 2E5D 2E5F 2E5M 2E5V 2E5W 2E5X 2E5Y 2E64 2E65 2E66 2E67 2E68 2E69 2E6A 2E6B 2E6C 2E6D 2E6E 2E6F 2E6G 2E6H 2E6K 2E6L 2E6M 2E6U 2E6V 2E6X 2E6Y 2E74 2E75 2E76 2E77 2E7A 2E7D 2E7E 2E7F 2E7I 2E7J 2E7L 2E7P 2E7Q 2E7R 2E7S 2E7T 2E7U 2E7V 2E7W 2E7X 2E7Y 2E7Z 2E80 2E81 2E82 2E83 2E84 2E85 2E86 2E87 2E88 2E89 2E8A 2E8B 2E8C 2E8E 2E8F 2E8G 2E8H 2E8I 2E8Q 2E8R 2E8S 2E8T 2E8U 2E8V 2E8W 2E8X 2E8Y 2E8Z 2E90 2E91 2E92 2E93 2E94 2E95 2E98 2E99 2E9A 2E9B 2E9C 2E9D 2E9E 2E9F 2E9L 2E9M 2E9N 2E9O 2E9P 2E9Q 2E9S 2E9U 2E9V 2E9W 2E9X 2E9Y 2EA1 2EA2 2EA3 2EA4 2EA7 2EA9 2EAA 2EAB 2EAC 2EAD 2EAE 2EAK 2EAL 2EAQ 2EAR 2EAT 2EAU 2EAV 2EAW 2EAX 2EAY 2EB0 2EB1 2EB2 2EB3 2EB4 2EB5 2EB6 2EB7 2EB8 2EB9 2EBA 2EBB 2EBD 2EBE 2EBF 2EBG 2EBH 2EBJ 2EBN 2EBO 2EBS 2EBY 2EC2 2EC5 2EC6 2EC8 2EC9 2ECE 2ECF 2ECK 2ECO 2ECP 2ECQ 2ECR 2ECS 2ECU 2ED3 2ED4 2ED5 2ED6 2EDA 2EDC 2EDM 2EEK 2EEN 2EEO 2EEP 2EEQ 2EER 2EEX 2EEY 2EEZ 2EF0 2EF1 2EF2 2EF4 2EF5 2EF6 2EF7 2EF8 2EF9 2EFB 2EFC 2EFD 2EFE 2EFF 2EFG 2EFH 2EFJ 2EFK 2EFL 2EFN 2EFO 2EFP 2EFQ 2EFR 2EFS 2EFT 2EFU 2EFV 2EFX 2EFY 2EG1 2EG2 2EG3 2EG4 2EG5 2EG6 2EG7 2EG8 2EG9 2EGB 2EGD 2EGG 2EGH 2EGI 2EGJ 2EGK 2EGL 2EGN 2EGO 2EGR 2EGS 2EGT 2EGU 2EGV 2EGW 2EGY 2EGZ 2EH1 2EH2 2EH3 2EH4 2EH5 2EH6 2EH7 2EH8 2EH9 2EHA 2EHB 2EHC 2EHD 2EHG 2EHH 2EHJ 2EHL 2EHO 2EHP 2EHQ 2EHS 2EHT 2EHU 2EHW 2EHZ 2EI0 2EI1 2EI2 2EI3 2EI4 2EI5 2EI6 2EI7 2EI8 2EI9 2EIA 2EIB 2EIC 2EID 2EIE 2EIF 2EIG 2EIH 2EII 2EIJ 2EIK 2EIL 2EIM 2EIN 2EIO 2EIP 2EIQ 2EIR 2EIS 2EIT 2EIU 2EIV 2EIW 2EIX 2EIY 2EIZ 2EJ0 2EJ1 2EJ2 2EJ3 2EJ5 2EJ6 2EJ8 2EJ9 2EJA 2EJB 2EJC 2EJD 2EJF 2EJG 2EJJ 2EJK 2EJL 2EJN 2EJQ 2EJR 2EJT 2EJU 2EJV 2EJW 2EJX 2EJZ 2EK0 2EK1 2EK2 2EK3 2EK4 2EK5 2EK6 2EK7 2EK8 2EK9 2EKA 2EKB 2EKC 2EKD 2EKE 2EKG 2EKL 2EKM 2EKN 2EKP 2EKQ 2EKS 2EKT 2EKU 2EKY 2EKZ 2EL0 2EL1 2EL2 2EL3 2EL7 2EL9 2ELA 2ELB 2ELC 2ELD 2ELE 2EMD 2EMN 2EMO 2EMQ 2EMR 2EMS 2EMT 2EMU 2EN5 2ENB 2END 2ENG 2ENI 2ENR 2ENU 2ENW 2ENX 2EO0 2EO4 2EO5 2EO7 2EO8 2EOA 2EP5 2EP7 2EPE 2EPF 2EPG 2EPH 2EPI 2EPJ 2EPK 2EPL 2EPM 2EPN 2EPO 2EQ5 2EQ6 2EQ7 2EQ8 2EQ9 2EQA 2EQB 2EQD 2EQL 2ER0 2ER6 2ER7 2ER9 2ERA 2ERB 2ERC 2ERF 2ERH 2ERJ 2ERK 2ERL 2ERO 2ERP 2ERQ 2ERV 2ERW 2ERX 2ERY 2ERZ 2ES0 2ES3 2ES4 2ES7 2ES9 2ESA 2ESB 2ESC 2ESD 2ESF 2ESH 2ESK 2ESL 2ESM 2ESN 2ESO 2ESP 2ESQ 2ESR 2ESS 2EST 2ESU 2ESV 2ESW 2ET1 2ET2 2ET6 2ET7 2ETA 2ETB 2ETC 2ETD 2ETE 2ETF 2ETH 2ETJ 2ETK 2ETL 2ETM 2ETN 2ETR 2ETS 2ETV 2ETX 2EU1 2EU2 2EU3 2EU7 2EU8 2EU9 2EUA 2EUC 2EUD 2EUF 2EUG 2EUH 2EUI 2EUK 2EUL 2EUM 2EUN 2EUO 2EUP 2EUQ 2EUR 2EUS 2EUT 2EUU 2EV0 2EV1 2EV2 2EV3 2EV4 2EV5 2EV6 2EV9 2EVA 2EVB 2EVC 2EVD 2EVE 2EVK 2EVL 2EVM 2EVO 2EVP 2EVR 2EVS 2EVT 2EVU 2EVV 2EVW 2EVX 2EW0 2EW1 2EW2 2EW5 2EW6 2EW7 2EW8 2EWA 2EWB 2EWC 2EWE 2EWF 2EWG 2EWH 2EWI 2EWK 2EWM 2EWN 2EWO 2EWP 2EWR 2EWS 2EWT 2EWU 2EWV 2EWW 2EWY 2EX0 2EX1 2EX2 2EX3 2EX4 2EX6 2EX8 2EX9 2EXA 2EXB 2EXC 2EXE 2EXH 2EXI 2EXJ 2EXK 2EXL 2EXM 2EXO 2EXR 2EXS 2EXT 2EXU 2EXV 2EXW 2EXX 2EXY 2EXZ 2EY1 2EY2 2EY4 2EY5 2EY6 2EYF 2EYH 2EYI 2EYJ 2EYL 2EYM 2EYN 2EYO 2EYP 2EYQ 2EYR 2EYS 2EYT 2EYU 2EZ0 2EZ1 2EZ2 2EZ4 2EZ7 2EZ8 2EZ9 2EZT 2EZU 2F00 2F01 2F02 2F06 2F07 2F08 2F0A 2F0C 2F0D 2F0E 2F0F 2F0G 2F0H 2F0I 2F0J 2F0K 2F0L 2F0M 2F0N 2F0O 2F0P 2F0Q 2F0R 2F0S 2F0T 2F0U 2F0V 2F0W 2F0X 2F0Y 2F0Z 2F10 2F11 2F12 2F13 2F14 2F15 2F16 2F17 2F18 2F19 2F1A 2F1B 2F1C 2F1D 2F1F 2F1G 2F1H 2F1I 2F1J 2F1K 2F1L 2F1M 2F1N 2F1O 2F1R 2F1S 2F1T 2F1V 2F1W 2F1X 2F1Y 2F1Z 2F20 2F21 2F22 2F23 2F24 2F25 2F26 2F27 2F28 2F29 2F2A 2F2B 2F2C 2F2E 2F2F 2F2G 2F2H 2F2K 2F2L 2F2N 2F2O 2F2P 2F2Q 2F2S 2F2T 2F2U 2F2V 2F2W 2F2X 2F30 2F31 2F32 2F34 2F35 2F36 2F37 2F38 2F3B 2F3C 2F3D 2F3E 2F3F 2F3G 2F3K 2F3L 2F3M 2F3N 2F3O 2F3P 2F3Q 2F3R 2F3S 2F3T 2F3U 2F3X 2F3Y 2F3Z 2F41 2F42 2F43 2F44 2F46 2F47 2F48 2F49 2F4A 2F4B 2F4E 2F4F 2F4G 2F4I 2F4J 2F4K 2F4L 2F4M 2F4N 2F4O 2F4P 2F4Q 2F4W 2F4Y 2F4Z 2F51 2F53 2F54 2F56 2F57 2F58 2F59 2F5A 2F5B 2F5C 2F5D 2F5E 2F5F 2F5G 2F5I 2F5J 2F5K 2F5M 2F5T 2F5U 2F5V 2F5W 2F5X 2F5Y 2F5Z 2F60 2F61 2F62 2F64 2F66 2F67 2F68 2F69 2F6A 2F6B 2F6C 2F6D 2F6E 2F6F 2F6G 2F6H 2F6I 2F6J 2F6K 2F6L 2F6M 2F6N 2F6P 2F6Q 2F6R 2F6S 2F6T 2F6U 2F6V 2F6W 2F6X 2F6Y 2F6Z 2F70 2F71 2F73 2F74 2F78 2F7A 2F7B 2F7C 2F7D 2F7E 2F7F 2F7I 2F7K 2F7L 2F7M 2F7N 2F7O 2F7P 2F7Q 2F7R 2F7S 2F7T 2F7V 2F7W 2F7X 2F7Y 2F7Z 2F80 2F81 2F82 2F83 2F84 2F86 2F89 2F8A 2F8C 2F8D 2F8E 2F8F 2F8G 2F8H 2F8I 2F8J 2F8L 2F8M 2F8O 2F8P 2F8Q 2F8V 2F8Y 2F8Z 2F90 2F91 2F92 2F93 2F94 2F95 2F96 2F97 2F98 2F99 2F9A 2F9B 2F9C 2F9D 2F9F 2F9G 2F9H 2F9I 2F9J 2F9K 2F9L 2F9M 2F9N 2F9O 2F9P 2F9Q 2F9R 2F9S 2F9T 2F9U 2F9V 2F9W 2F9Y 2F9Z 2FA0 2FA1 2FA2 2FA3 2FA4 2FA5 2FA7 2FA8 2FA9 2FAC 2FAD 2FAE 2FAF 2FAH 2FAI 2FAK 2FAL 2FAM 2FAO 2FAP 2FAQ 2FAR 2FAT 2FAU 2FAV 2FAW 2FAX 2FAZ 2FB0 2FB2 2FB3 2FB4 2FB5 2FB6 2FB8 2FB9 2FBA 2FBB 2FBD 2FBE 2FBH 2FBI 2FBJ 2FBK 2FBL 2FBM 2FBN 2FBO 2FBP 2FBQ 2FBR 2FBT 2FBV 2FBW 2FBX 2FBY 2FBZ 2FC0 2FC1 2FC2 2FC3 2FCA 2FCB 2FCF 2FCH 2FCJ 2FCK 2FCL 2FCM 2FCN 2FCO 2FCP 2FCQ 2FCR 2FCS 2FCT 2FCU 2FCV 2FCW 2FD2 2FD3 2FD4 2FD5 2FD6 2FD7 2FD9 2FDA 2FDB 2FDD 2FDE 2FDJ 2FDM 2FDN 2FDO 2FDP 2FDQ 2FDR 2FDS 2FDU 2FDV 2FDW 2FDX 2FDY 2FE1 2FE3 2FE4 2FE5 2FE6 2FE7 2FE8 2FEA 2FEC 2FED 2FEE 2FEF 2FEL 2FEM 2FEN 2FEO 2FEP 2FEQ 2FER 2FES 2FET 2FEU 2FEX 2FEZ 2FF1 2FF2 2FF3 2FF4 2FF5 2FF6 2FF7 2FFA 2FFB 2FFC 2FFD 2FFF 2FFG 2FFH 2FFI 2FFJ 2FFL 2FFM 2FFN 2FFQ 2FFR 2FFS 2FFU 2FFV 2FFX 2FFY 2FFZ 2FG0 2FG1 2FG4 2FG5 2FG6 2FG7 2FG8 2FG9 2FGB 2FGC 2FGE 2FGF 2FGG 2FGH 2FGI 2FGJ 2FGK 2FGL 2FGN 2FGO 2FGQ 2FGR 2FGS 2FGT 2FGU 2FGV 2FGW 2FGY 2FGZ 2FH1 2FH2 2FH3 2FH4 2FH5 2FH6 2FH7 2FH8 2FH9 2FHA 2FHB 2FHC 2FHD 2FHE 2FHF 2FHG 2FHH 2FHI 2FHJ 2FHK 2FHL 2FHN 2FHP 2FHQ 2FHR 2FHS 2FHT 2FHX 2FHY 2FHZ 2FI0 2FI1 2FI3 2FI4 2FI5 2FI7 2FI9 2FIA 2FIB 2FIC 2FID 2FIE 2FIF 2FIK 2FIM 2FIP 2FIQ 2FIR 2FIT 2FIU 2FIV 2FIW 2FIX 2FIY 2FJ0 2FJ1 2FJ2 2FJ8 2FJ9 2FJA 2FJB 2FJC 2FJD 2FJE 2FJF 2FJG 2FJH 2FJI 2FJK 2FJM 2FJN 2FJP 2FJR 2FJS 2FJT 2FJU 2FJY 2FJZ 2FK0 2FK1 2FK2 2FK3 2FK5 2FK7 2FK8 2FK9 2FKA 2FKB 2FKD 2FKE 2FKF 2FKG 2FKJ 2FKK 2FKL 2FKM 2FKN 2FKO 2FKP 2FKW 2FKY 2FKZ 2FL0 2FL1 2FL2 2FL4 2FL5 2FL6 2FL7 2FLA 2FLB 2FLE 2FLF 2FLH 2FLI 2FLK 2FLM 2FLO 2FLQ 2FLR 2FLS 2FLT 2FLU 2FLV 2FLW 2FLZ 2FM0 2FM1 2FM2 2FM5 2FM6 2FM7 2FM8 2FM9 2FMA 2FMB 2FMD 2FME 2FMF 2FMG 2FMH 2FMI 2FMJ 2FMK 2FML 2FMM 2FMN 2FMO 2FMU 2FMX 2FMY 2FMZ 2FN0 2FN1 2FN3 2FN4 2FN6 2FN7 2FN8 2FN9 2FNA 2FNC 2FNE 2FNI 2FNJ 2FNK 2FNM 2FNN 2FNO 2FNP 2FNQ 2FNS 2FNT 2FNU 2FNW 2FNX 2FO0 2FO3 2FO4 2FO5 2FO7 2FO9 2FOA 2FOB 2FOC 2FOD 2FOE 2FOF 2FOG 2FOH 2FOI 2FOJ 2FOK 2FOL 2FOM 2FON 2FOO 2FOP 2FOQ 2FOR 2FOS 2FOT 2FOU 2FOV 2FOX 2FOY 2FOZ 2FP0 2FP1 2FP2 2FP3 2FP4 2FP7 2FP8 2FP9 2FPB 2FPC 2FPD 2FPE 2FPF 2FPG 2FPH 2FPI 2FPK 2FPL 2FPM 2FPN 2FPO 2FPP 2FPQ 2FPR 2FPS 2FPT 2FPU 2FPV 2FPW 2FPX 2FPY 2FPZ 2FQ1 2FQ3 2FQ4 2FQ6 2FQ9 2FQD 2FQE 2FQF 2FQG 2FQI 2FQL 2FQM 2FQO 2FQP 2FQQ 2FQT 2FQW 2FQX 2FQY 2FR0 2FR1 2FR2 2FR3 2FR5 2FR6 2FR7 2FR8 2FRA 2FRD 2FRE 2FRF 2FRG 2FRH 2FRI 2FRJ 2FRK 2FRP 2FRQ 2FRS 2FRV 2FRX 2FRZ 2FS2 2FS3 2FS4 2FS5 2FS6 2FS7 2FS8 2FS9 2FSA 2FSD 2FSE 2FSF 2FSG 2FSH 2FSI 2FSJ 2FSK 2FSL 2FSM 2FSN 2FSO 2FSQ 2FSR 2FSS 2FST 2FSU 2FSV 2FSW 2FSX 2FSY 2FSZ 2FT0 2FT1 2FT2 2FT3 2FT6 2FT7 2FT8 2FT9 2FTA 2FTB 2FTD 2FTK 2FTL 2FTM 2FTN 2FTO 2FTP 2FTQ 2FTR 2FTS 2FTW 2FTX 2FTY 2FTZ 2FU0 2FU2 2FU3 2FU4 2FU5 2FU6 2FU7 2FU8 2FU9 2FUA 2FUB 2FUC 2FUD 2FUE 2FUF 2FUG 2FUJ 2FUK 2FUL 2FUM 2FUN 2FUP 2FUQ 2FUR 2FUS 2FUT 2FUV 2FUZ 2FV0 2FV1 2FV2 2FV5 2FV7 2FV8 2FV9 2FVC 2FVD 2FVG 2FVH 2FVJ 2FVK 2FVL 2FVM 2FVU 2FVV 2FVX 2FVY 2FVZ 2FW0 2FW1 2FW2 2FW3 2FW4 2FW5 2FW6 2FW7 2FW8 2FW9 2FWA 2FWB 2FWE 2FWF 2FWG 2FWH 2FWI 2FWJ 2FWM 2FWN 2FWO 2FWP 2FWQ 2FWR 2FWT 2FWV 2FWW 2FWY 2FWZ 2FX0 2FX2 2FX3 2FX4 2FX5 2FX6 2FX7 2FX8 2FX9 2FXA 2FXD 2FXE 2FXF 2FXG 2FXH 2FXI 2FXJ 2FXK 2FXL 2FXM 2FXO 2FXQ 2FXR 2FXS 2FXT 2FXU 2FXV 2FY2 2FY3 2FY4 2FY5 2FY6 2FY7 2FY8 2FYA 2FYB 2FYC 2FYD 2FYE 2FYF 2FYG 2FYI 2FYK 2FYM 2FYN 2FYO 2FYP 2FYQ 2FYR 2FYS 2FYT 2FYU 2FYV 2FYW 2FYX 2FYY 2FYZ 2FZ1 2FZ3 2FZ4 2FZ6 2FZ8 2FZ9 2FZB 2FZC 2FZD 2FZE 2FZF 2FZG 2FZH 2FZI 2FZJ 2FZK 2FZL 2FZM 2FZN 2FZP 2FZS 2FZT 2FZU 2FZV 2FZW 2FZZ 2G00 2G01 2G02 2G03 2G04 2G06 2G07 2G08 2G09 2G0A 2G0B 2G0C 2G0D 2G0E 2G0F 2G0G 2G0H 2G0I 2G0J 2G0N 2G0R 2G0S 2G0T 2G0V 2G0W 2G0X 2G0Y 2G0Z 2G10 2G11 2G12 2G13 2G14 2G15 2G16 2G17 2G18 2G19 2G1A 2G1H 2G1J 2G1K 2G1L 2G1M 2G1N 2G1O 2G1Q 2G1R 2G1S 2G1T 2G1U 2G1Y 2G20 2G21 2G22 2G24 2G25 2G26 2G27 2G28 2G29 2G2C 2G2D 2G2F 2G2H 2G2I 2G2L 2G2N 2G2O 2G2P 2G2Q 2G2R 2G2S 2G2U 2G2W 2G2X 2G2Y 2G2Z 2G30 2G33 2G34 2G36 2G37 2G38 2G39 2G3A 2G3B 2G3D 2G3F 2G3H 2G3I 2G3J 2G3K 2G3M 2G3N 2G3O 2G3P 2G3R 2G3T 2G3V 2G3W 2G3X 2G3Y 2G3Z 2G40 2G41 2G42 2G43 2G44 2G45 2G47 2G48 2G49 2G4C 2G4D 2G4E 2G4F 2G4G 2G4H 2G4I 2G4J 2G4K 2G4L 2G4M 2G4N 2G4O 2G4P 2G4Q 2G4R 2G4S 2G4T 2G4U 2G4V 2G4W 2G4X 2G4Y 2G4Z 2G50 2G51 2G52 2G54 2G55 2G56 2G58 2G59 2G5B 2G5C 2G5D 2G5F 2G5G 2G5H 2G5I 2G5L 2G5N 2G5O 2G5P 2G5R 2G5T 2G5U 2G5V 2G5W 2G5X 2G5Z 2G60 2G62 2G63 2G64 2G66 2G67 2G69 2G6B 2G6D 2G6E 2G6F 2G6G 2G6H 2G6I 2G6J 2G6K 2G6L 2G6M 2G6N 2G6O 2G6P 2G6Q 2G6T 2G6V 2G6W 2G6X 2G6Y 2G6Z 2G70 2G71 2G72 2G73 2G74 2G75 2G76 2G77 2G78 2G79 2G7B 2G7C 2G7E 2G7F 2G7G 2G7I 2G7K 2G7L 2G7M 2G7N 2G7O 2G7P 2G7Q 2G7R 2G7S 2G7U 2G7Y 2G7Z 2G80 2G81 2G82 2G83 2G84 2G85 2G86 2G87 2G88 2G89 2G8A 2G8C 2G8D 2G8E 2G8G 2G8J 2G8L 2G8N 2G8Q 2G8R 2G8S 2G8T 2G8X 2G8Y 2G8Z 2G93 2G94 2G95 2G96 2G97 2G98 2G99 2G9A 2G9D 2G9E 2G9F 2G9G 2G9H 2G9I 2G9K 2G9N 2G9Q 2G9R 2G9T 2G9U 2G9V 2G9W 2G9X 2G9Y 2G9Z 2GA0 2GA1 2GA2 2GA3 2GA4 2GA6 2GA8 2GA9 2GAA 2GAB 2GAC 2GAE 2GAF 2GAG 2GAH 2GAI 2GAJ 2GAK 2GAL 2GAM 2GAN 2GAO 2GAR 2GAS 2GAU 2GAW 2GAX 2GAZ 2GB0 2GB2 2GB3 2GB4 2GB5 2GBA 2GBB 2GBC 2GBF 2GBG 2GBI 2GBJ 2GBK 2GBL 2GBM 2GBN 2GBO 2GBP 2GBR 2GBT 2GBU 2GBV 2GBW 2GBX 2GBY 2GBZ 2GC0 2GC1 2GC2 2GC3 2GC4 2GC5 2GC6 2GC7 2GC8 2GC9 2GCA 2GCB 2GCD 2GCE 2GCG 2GCH 2GCI 2GCJ 2GCL 2GCN 2GCO 2GCP 2GCQ 2GCT 2GCU 2GCY 2GD0 2GD1 2GD2 2GD4 2GD5 2GD6 2GD8 2GD9 2GDC 2GDD 2GDE 2GDF 2GDG 2GDJ 2GDM 2GDN 2GDO 2GDQ 2GDR 2GDS 2GDU 2GDV 2GDZ 2GE3 2GE7 2GE8 2GEB 2GEC 2GED 2GEE 2GEF 2GEH 2GEJ 2GEK 2GEL 2GEM 2GEN 2GEP 2GER 2GES 2GET 2GEU 2GEV 2GEW 2GEX 2GEY 2GEZ 2GF0 2GF2 2GF3 2GF4 2GF6 2GF7 2GF9 2GFA 2GFB 2GFC 2GFD 2GFE 2GFF 2GFG 2GFH 2GFI 2GFJ 2GFK 2GFN 2GFO 2GFP 2GFQ 2GFS 2GFT 2GFV 2GFW 2GFX 2GFY 2GG0 2GG2 2GG3 2GG4 2GG5 2GG6 2GG7 2GG8 2GG9 2GGA 2GGB 2GGC 2GGD 2GGE 2GGG 2GGH 2GGI 2GGJ 2GGK 2GGL 2GGM 2GGN 2GGO 2GGQ 2GGS 2GGT 2GGU 2GGV 2GGX 2GGZ 2GH0 2GH1 2GH2 2GH4 2GH5 2GH6 2GH7 2GH8 2GH9 2GHA 2GHB 2GHC 2GHD 2GHE 2GHG 2GHH 2GHI 2GHJ 2GHK 2GHL 2GHM 2GHO 2GHP 2GHQ 2GHR 2GHS 2GHT 2GHU 2GHV 2GHW 2GHY 2GHZ 2GI0 2GI3 2GI7 2GI9 2GIA 2GIB 2GID 2GIF 2GIL 2GIM 2GIN 2GIQ 2GIR 2GIT 2GIU 2GIV 2GIX 2GIY 2GIZ 2GJ1 2GJ2 2GJ3 2GJ4 2GJ5 2GJ6 2GJ7 2GJ8 2GJ9 2GJA 2GJD 2GJG 2GJJ 2GJK 2GJL 2GJM 2GJN 2GJP 2GJR 2GJS 2GJT 2GJU 2GJV 2GJX 2GJZ 2GK0 2GK1 2GK2 2GK3 2GK4 2GK6 2GK7 2GK9 2GKE 2GKG 2GKI 2GKJ 2GKL 2GKM 2GKN 2GKO 2GKP 2GKR 2GKS 2GKT 2GKV 2GKW 2GL0 2GL2 2GL3 2GL5 2GL7 2GL8 2GL9 2GLF 2GLJ 2GLK 2GLL 2GLM 2GLN 2GLP 2GLQ 2GLR 2GLS 2GLT 2GLU 2GLV 2GLX 2GLZ 2GM1 2GM3 2GM5 2GM7 2GM8 2GM9 2GME 2GMF 2GMH 2GMI 2GMJ 2GMK 2GML 2GMM 2GMN 2GMP 2GMQ 2GMR 2GMS 2GMT 2GMU 2GMV 2GMW 2GMX 2GMY 2GN0 2GN1 2GN2 2GN3 2GN4 2GN5 2GN6 2GN7 2GN8 2GN9 2GNA 2GNB 2GNC 2GND 2GNF 2GNG 2GNH 2GNI 2GNJ 2GNK 2GNL 2GNM 2GNN 2GNO 2GNP 2GNQ 2GNS 2GNT 2GNU 2GNV 2GNW 2GNX 2GO1 2GO2 2GO3 2GO4 2GO7 2GO8 2GOJ 2GOK 2GOL 2GOM 2GON 2GOO 2GOP 2GOU 2GOX 2GOY 2GP0 2GP1 2GP3 2GP4 2GP5 2GP6 2GP7 2GP9 2GPA 2GPB 2GPC 2GPE 2GPH 2GPI 2GPJ 2GPL 2GPN 2GPO 2GPP 2GPR 2GPS 2GPT 2GPU 2GPV 2GPW 2GPY 2GPZ 2GQ0 2GQ1 2GQ2 2GQ3 2GQ8 2GQ9 2GQA 2GQD 2GQF 2GQG 2GQN 2GQP 2GQQ 2GQR 2GQS 2GQT 2GQU 2GQV 2GQW 2GQX 2GR0 2GR1 2GR2 2GR3 2GR6 2GR7 2GR8 2GR9 2GRA 2GRC 2GRE 2GRF 2GRH 2GRJ 2GRK 2GRL 2GRM 2GRN 2GRO 2GRP 2GRQ 2GRR 2GRT 2GRU 2GRV 2GRX 2GRY 2GRZ 2GS2 2GS3 2GS4 2GS5 2GS6 2GS7 2GS8 2GS9 2GSA 2GSC 2GSD 2GSE 2GSF 2GSG 2GSH 2GSI 2GSJ 2GSK 2GSL 2GSM 2GSN 2GSO 2GSP 2GSQ 2GSR 2GSS 2GST 2GSU 2GSV 2GSW 2GSY 2GSZ 2GT1 2GT2 2GT4 2GT7 2GT8 2GT9 2GTA 2GTB 2GTC 2GTD 2GTE 2GTF 2GTG 2GTH 2GTI 2GTK 2GTL 2GTM 2GTN 2GTP 2GTQ 2GTR 2GTS 2GTU 2GTW 2GTX 2GTY 2GTZ 2GU0 2GU1 2GU2 2GU3 2GU4 2GU5 2GU6 2GU7 2GU8 2GU9 2GUB 2GUC 2GUD 2GUE 2GUF 2GUG 2GUH 2GUI 2GUJ 2GUK 2GUM 2GUO 2GUP 2GUS 2GUU 2GUV 2GUW 2GUX 2GUY 2GUZ 2GV0 2GV2 2GV5 2GV6 2GV7 2GV8 2GV9 2GVC 2GVD 2GVF 2GVG 2GVH 2GVI 2GVJ 2GVK 2GVL 2GVM 2GVN 2GVQ 2GVU 2GVV 2GVW 2GVX 2GVY 2GVZ 2GW1 2GW2 2GW3 2GW4 2GW5 2GW8 2GWC 2GWD 2GWF 2GWG 2GWH 2GWJ 2GWK 2GWL 2GWM 2GWN 2GWO 2GWR 2GWW 2GWX 2GX0 2GX2 2GX4 2GX5 2GX6 2GX8 2GX9 2GXF 2GXG 2GXQ 2GXS 2GXU 2GY5 2GY7 2GYD 2GYI 2GYK 2GYO 2GYP 2GYQ 2GYR 2GYS 2GYU 2GYV 2GYW 2GYY 2GYZ 2GZ1 2GZ2 2GZ3 2GZ4 2GZ5 2GZ6 2GZ7 2GZ8 2GZ9 2GZA 2GZB 2GZD 2GZE 2GZF 2GZG 2GZH 2GZI 2GZJ 2GZL 2GZM 2GZQ 2GZR 2GZS 2GZV 2GZW 2GZX 2H00 2H01 2H02 2H03 2H04 2H06 2H07 2H08 2H09 2H0A 2H0B 2H0D 2H0E 2H0F 2H0G 2H0H 2H0I 2H0J 2H0K 2H0L 2H0M 2H0Q 2H0R 2H0T 2H0U 2H0V 2H0Y 2H10 2H11 2H12 2H13 2H14 2H15 2H16 2H17 2H18 2H19 2H1A 2H1B 2H1C 2H1D 2H1E 2H1F 2H1G 2H1H 2H1I 2H1J 2H1L 2H1N 2H1P 2H1R 2H1S 2H1T 2H1U 2H1V 2H1W 2H1X 2H1Y 2H21 2H23 2H24 2H26 2H28 2H29 2H2A 2H2B 2H2C 2H2D 2H2E 2H2F 2H2G 2H2H 2H2I 2H2J 2H2K 2H2N 2H2P 2H2Q 2H2R 2H2S 2H2T 2H2U 2H2W 2H2Y 2H2Z 2H30 2H31 2H32 2H34 2H36 2H39 2H3B 2H3D 2H3E 2H3G 2H3H 2H3L 2H3M 2H3N 2H3P 2H3R 2H3U 2H3W 2H3X 2H40 2H42 2H43 2H44 2H46 2H47 2H48 2H4C 2H4E 2H4F 2H4G 2H4H 2H4I 2H4J 2H4K 2H4L 2H4M 2H4N 2H4O 2H4P 2H4Q 2H4R 2H4T 2H4U 2H4V 2H4W 2H4X 2H4Y 2H4Z 2H51 2H52 2H54 2H55 2H56 2H57 2H58 2H59 2H5A 2H5C 2H5D 2H5E 2H5F 2H5G 2H5I 2H5J 2H5K 2H5L 2H5N 2H5O 2H5P 2H5Q 2H5R 2H5S 2H5U 2H5X 2H5Y 2H5Z 2H61 2H62 2H63 2H64 2H65 2H66 2H68 2H6A 2H6B 2H6C 2H6D 2H6E 2H6F 2H6G 2H6H 2H6I 2H6J 2H6K 2H6L 2H6M 2H6N 2H6O 2H6P 2H6Q 2H6R 2H6S 2H6T 2H6U 2H6V 2H6X 2H6Y 2H6Z 2H70 2H71 2H72 2H73 2H74 2H75 2H76 2H77 2H79 2H7C 2H7J 2H7O 2H7Q 2H7R 2H7S 2H7V 2H7W 2H7X 2H7Y 2H7Z 2H84 2H85 2H88 2H89 2H8D 2H8E 2H8F 2H8G 2H8H 2H8I 2H8K 2H8L 2H8M 2H8N 2H8O 2H8P 2H8Q 2H8U 2H8V 2H8X 2H8Z 2H90 2H92 2H94 2H96 2H98 2H99 2H9A 2H9B 2H9C 2H9D 2H9E 2H9F 2H9G 2H9H 2H9I 2H9J 2H9K 2H9L 2H9M 2H9N 2H9P 2H9T 2H9U 2H9V 2H9W 2H9Y 2HA0 2HA2 2HA3 2HA4 2HA5 2HA6 2HA7 2HA8 2HA9 2HAD 2HAE 2HAF 2HAG 2HAH 2HAI 2HAK 2HAL 2HAM 2HAQ 2HAR 2HAS 2HAU 2HAV 2HAW 2HAY 2HAZ 2HB0 2HB1 2HB2 2HB3 2HB4 2HB5 2HB6 2HB7 2HB8 2HB9 2HBA 2HBB 2HBC 2HBD 2HBE 2HBF 2HBG 2HBH 2HBJ 2HBK 2HBL 2HBM 2HBO 2HBQ 2HBR 2HBS 2HBT 2HBU 2HBV 2HBW 2HBX 2HBY 2HBZ 2HC0 2HC1 2HC2 2HC4 2HC8 2HC9 2HCA 2HCB 2HCD 2HCF 2HCG 2HCH 2HCI 2HCJ 2HCK 2HCM 2HCN 2HCO 2HCR 2HCS 2HCT 2HCU 2HCV 2HCZ 2HD0 2HD1 2HD3 2HD4 2HD5 2HD6 2HD9 2HDA 2HDB 2HDF 2HDH 2HDI 2HDJ 2HDK 2HDN 2HDO 2HDQ 2HDR 2HDS 2HDU 2HDV 2HDW 2HDX 2HDZ 2HE0 2HE2 2HE3 2HE4 2HE5 2HE7 2HE8 2HE9 2HEA 2HEB 2HEC 2HED 2HEE 2HEF 2HEG 2HEH 2HEI 2HEJ 2HEK 2HEL 2HEN 2HES 2HET 2HEU 2HEV 2HEW 2HEX 2HEY 2HEZ 2HF0 2HF1 2HF2 2HF3 2HF4 2HF7 2HF8 2HF9 2HFB 2HFC 2HFE 2HFF 2HFG 2HFJ 2HFK 2HFN 2HFO 2HFP 2HFS 2HFT 2HFU 2HFW 2HFZ 2HG0 2HG1 2HG2 2HG3 2HG4 2HG5 2HG8 2HG9 2HGD 2HGS 2HGT 2HGV 2HGW 2HGX 2HGY 2HGZ 2HH0 2HH1 2HH5 2HH6 2HH7 2HH9 2HHA 2HHB 2HHC 2HHD 2HHE 2HHF 2HHG 2HHJ 2HHK 2HHL 2HHM 2HHN 2HHP 2HHZ 2HI0 2HI1 2HI2 2HI4 2HI7 2HI8 2HI9 2HIA 2HIB 2HIG 2HIH 2HII 2HIJ 2HIK 2HIM 2HIN 2HIO 2HIP 2HIQ 2HIS 2HIT 2HIV 2HIW 2HIX 2HIY 2HIZ 2HJ0 2HJ1 2HJ3 2HJ4 2HJ6 2HJ9 2HJB 2HJD 2HJE 2HJF 2HJG 2HJH 2HJK 2HJL 2HJM 2HJN 2HJO 2HJP 2HJR 2HJS 2HJV 2HJW 2HK0 2HK1 2HK2 2HK3 2HK5 2HK6 2HK7 2HK8 2HK9 2HKA 2HKD 2HKE 2HKF 2HKH 2HKI 2HKJ 2HKK 2HKL 2HKM 2HKN 2HKO 2HKP 2HKQ 2HKR 2HKU 2HKV 2HKX 2HKZ 2HL0 2HL1 2HL2 2HL3 2HL4 2HL5 2HL6 2HL7 2HL8 2HL9 2HLA 2HLC 2HLD 2HLE 2HLF 2HLH 2HLJ 2HLN 2HLO 2HLP 2HLQ 2HLR 2HLS 2HLV 2HLY 2HLZ 2HM1 2HM7 2HMA 2HMB 2HMC 2HMF 2HMG 2HMH 2HMJ 2HMK 2HML 2HMM 2HMN 2HMO 2HMP 2HMQ 2HMS 2HMT 2HMU 2HMV 2HMW 2HMY 2HMZ 2HN1 2HN2 2HN7 2HN9 2HNC 2HND 2HNE 2HNF 2HNG 2HNH 2HNI 2HNK 2HNL 2HNP 2HNQ 2HNT 2HNU 2HNV 2HNW 2HNX 2HNY 2HNZ 2HO0 2HO1 2HO2 2HO4 2HO5 2HOB 2HOC 2HOD 2HOE 2HOG 2HOH 2HOQ 2HOR 2HOW 2HOX 2HOY 2HOZ 2HP0 2HP1 2HP2 2HP3 2HP4 2HP5 2HP6 2HP7 2HP9 2HPA 2HPB 2HPC 2HPD 2HPE 2HPF 2HPG 2HPH 2HPI 2HPJ 2HPL 2HPM 2HPO 2HPP 2HPQ 2HPR 2HPS 2HPT 2HPV 2HPW 2HPY 2HPZ 2HQ0 2HQ1 2HQ4 2HQ5 2HQ6 2HQ7 2HQ8 2HQ9 2HQA 2HQB 2HQC 2HQD 2HQE 2HQF 2HQG 2HQH 2HQJ 2HQK 2HQL 2HQM 2HQQ 2HQS 2HQT 2HQU 2HQV 2HQW 2HQX 2HQY 2HQZ 2HR2 2HR3 2HR5 2HR6 2HR7 2HR8 2HRA 2HRB 2HRC 2HRD 2HRE 2HRG 2HRH 2HRK 2HRL 2HRM 2HRO 2HRP 2HRQ 2HRR 2HRS 2HRT 2HRU 2HRV 2HRW 2HRX 2HRY 2HRZ 2HS0 2HS1 2HS2 2HS3 2HS4 2HS5 2HS6 2HS8 2HSA 2HSB 2HSD 2HSE 2HSG 2HSH 2HSI 2HSJ 2HSM 2HSN 2HSQ 2HSW 2HSZ 2HT2 2HT3 2HT4 2HT5 2HT6 2HT7 2HT8 2HT9 2HTA 2HTB 2HTD 2HTE 2HTH 2HTI 2HTK 2HTL 2HTM 2HTN 2HTQ 2HTR 2HTS 2HTU 2HTV 2HTW 2HTX 2HTY 2HU0 2HU1 2HU2 2HU3 2HU4 2HU5 2HU6 2HU7 2HU8 2HU9 2HUB 2HUC 2HUE 2HUF 2HUH 2HUI 2HUJ 2HUK 2HUL 2HUM 2HUN 2HUO 2HUP 2HUQ 2HUR 2HUT 2HUU 2HUV 2HUW 2HUX 2HUZ 2HV2 2HV5 2HV6 2HV7 2HV8 2HV9 2HVB 2HVC 2HVD 2HVE 2HVF 2HVG 2HVJ 2HVK 2HVL 2HVM 2HVN 2HVO 2HVP 2HVQ 2HVV 2HVW 2HVX 2HW1 2HW2 2HW4 2HW5 2HW6 2HW7 2HW9 2HWA 2HWB 2HWC 2HWD 2HWE 2HWF 2HWG 2HWH 2HWI 2HWJ 2HWK 2HWL 2HWM 2HWN 2HWO 2HWP 2HWQ 2HWR 2HWU 2HWV 2HWW 2HWX 2HWY 2HWZ 2HX0 2HX1 2HX2 2HX3 2HX4 2HX5 2HX7 2HX8 2HX9 2HXA 2HXB 2HXC 2HXD 2HXG 2HXI 2HXK 2HXL 2HXM 2HXO 2HXP 2HXQ 2HXR 2HXS 2HXT 2HXU 2HXV 2HXW 2HXX 2HXY 2HXZ 2HY0 2HY1 2HY3 2HY5 2HY6 2HY7 2HY8 2HYB 2HYD 2HYE 2HYF 2HYG 2HYJ 2HYK 2HYO 2HYP 2HYQ 2HYR 2HYS 2HYT 2HYU 2HYV 2HYW 2HYX 2HYY 2HYZ 2HZ0 2HZ1 2HZ2 2HZ3 2HZ4 2HZ5 2HZ6 2HZ7 2HZ9 2HZA 2HZB 2HZC 2HZE 2HZF 2HZG 2HZH 2HZI 2HZK 2HZL 2HZM 2HZN 2HZP 2HZQ 2HZR 2HZS 2HZT 2HZY 2I00 2I02 2I03 2I04 2I07 2I08 2I0A 2I0B 2I0C 2I0D 2I0E 2I0F 2I0G 2I0H 2I0I 2I0J 2I0K 2I0L 2I0M 2I0O 2I0R 2I0S 2I0T 2I0U 2I0V 2I0W 2I0X 2I0Y 2I0Z 2I10 2I14 2I15 2I16 2I17 2I19 2I1A 2I1B 2I1J 2I1K 2I1L 2I1M 2I1N 2I1O 2I1Q 2I1R 2I1S 2I1U 2I1V 2I1W 2I1X 2I1Y 2I20 2I21 2I22 2I24 2I25 2I26 2I27 2I29 2I2A 2I2B 2I2C 2I2D 2I2F 2I2L 2I2O 2I2Q 2I2R 2I2S 2I2W 2I2X 2I2Z 2I30 2I32 2I33 2I34 2I35 2I36 2I37 2I39 2I3A 2I3C 2I3D 2I3F 2I3G 2I3H 2I3I 2I3O 2I3R 2I3S 2I3T 2I3U 2I3V 2I3W 2I3Y 2I3Z 2I40 2I42 2I44 2I45 2I46 2I47 2I48 2I49 2I4A 2I4B 2I4C 2I4D 2I4E 2I4G 2I4H 2I4I 2I4J 2I4L 2I4M 2I4N 2I4O 2I4P 2I4Q 2I4R 2I4S 2I4T 2I4U 2I4V 2I4W 2I4X 2I4Z 2I51 2I52 2I53 2I54 2I55 2I56 2I57 2I58 2I5B 2I5C 2I5D 2I5E 2I5F 2I5G 2I5H 2I5I 2I5J 2I5K 2I5L 2I5M 2I5N 2I5P 2I5Q 2I5R 2I5T 2I5U 2I5V 2I5X 2I5Y 2I5Z 2I60 2I61 2I62 2I65 2I66 2I67 2I69 2I6A 2I6B 2I6D 2I6E 2I6F 2I6G 2I6H 2I6I 2I6J 2I6K 2I6L 2I6M 2I6O 2I6P 2I6Q 2I6R 2I6S 2I6T 2I6U 2I6V 2I6W 2I6X 2I6Y 2I6Z 2I71 2I72 2I74 2I75 2I76 2I78 2I79 2I7A 2I7B 2I7C 2I7D 2I7F 2I7G 2I7H 2I7N 2I7O 2I7P 2I7Q 2I7R 2I7S 2I7T 2I7V 2I7X 2I80 2I81 2I87 2I88 2I89 2I8A 2I8B 2I8C 2I8D 2I8E 2I8T 2I8U 2I99 2I9A 2I9B 2I9C 2I9D 2I9E 2I9F 2I9I 2I9L 2I9P 2I9U 2I9V 2I9W 2I9X 2I9Z 2IA0 2IA1 2IA2 2IA4 2IA5 2IA7 2IA8 2IA9 2IAA 2IAB 2IAD 2IAE 2IAF 2IAG 2IAH 2IAI 2IAJ 2IAK 2IAL 2IAM 2IAN 2IAO 2IAP 2IAQ 2IAR 2IAS 2IAT 2IAU 2IAV 2IAW 2IAX 2IAY 2IAZ 2IB0 2IB5 2IB6 2IB7 2IB8 2IB9 2IBA 2IBB 2IBD 2IBF 2IBG 2IBI 2IBJ 2IBL 2IBM 2IBN 2IBO 2IBP 2IBU 2IBW 2IBX 2IBY 2IBZ 2IC0 2IC1 2IC2 2IC3 2IC5 2IC6 2IC7 2IC8 2IC9 2ICA 2ICC 2ICE 2ICF 2ICG 2ICH 2ICI 2ICJ 2ICK 2ICP 2ICQ 2ICR 2ICS 2ICT 2ICU 2ICV 2ICW 2ICX 2ICY 2ID0 2ID1 2ID2 2ID3 2ID4 2ID5 2ID6 2ID7 2ID8 2ID9 2IDB 2IDC 2IDE 2IDF 2IDG 2IDH 2IDJ 2IDK 2IDL 2IDM 2IDO 2IDQ 2IDR 2IDS 2IDT 2IDU 2IDV 2IDW 2IDX 2IDZ 2IE0 2IE2 2IE3 2IE4 2IE6 2IE7 2IE8 2IEA 2IEB 2IEC 2IED 2IEE 2IEG 2IEH 2IEI 2IEJ 2IEK 2IEL 2IEN 2IEO 2IEP 2IEQ 2IER 2IES 2IEW 2IEX 2IEY 2IEZ 2IF0 2IF2 2IF4 2IF5 2IF6 2IF7 2IF8 2IF9 2IFA 2IFB 2IFC 2IFD 2IFF 2IFG 2IFQ 2IFR 2IFT 2IFU 2IFV 2IFW 2IFX 2IFY 2IG0 2IG2 2IG3 2IG6 2IG7 2IG8 2IG9 2IGA 2IGB 2IGC 2IGD 2IGF 2IGI 2IGK 2IGL 2IGM 2IGN 2IGO 2IGP 2IGQ 2IGS 2IGT 2IGV 2IGW 2IGX 2IGY 2IH1 2IH3 2IH8 2IH9 2IHB 2IHC 2IHD 2IHE 2IHF 2IHJ 2IHK 2IHL 2IHO 2IHP 2IHQ 2IHR 2IHS 2IHT 2IHU 2IHV 2IHW 2IHY 2IHZ 2II0 2II1 2II2 2II3 2II4 2II5 2II6 2II7 2II8 2II9 2IIA 2IIB 2IIC 2IID 2IIH 2III 2IIK 2IIM 2IIP 2IIQ 2IIR 2IIT 2IIU 2IIV 2IIY 2IIZ 2IJ0 2IJ2 2IJ3 2IJ4 2IJ5 2IJ7 2IJ9 2IJA 2IJC 2IJD 2IJE 2IJF 2IJG 2IJH 2IJI 2IJJ 2IJK 2IJL 2IJM 2IJN 2IJO 2IJQ 2IJR 2IJX 2IJZ 2IK0 2IK1 2IK2 2IK4 2IK6 2IK7 2IK8 2IK9 2IKB 2IKC 2IKF 2IKG 2IKH 2IKI 2IKJ 2IKK 2IKO 2IKQ 2IKS 2IKU 2IL1 2IL2 2IL3 2IL4 2IL5 2ILA 2ILI 2ILK 2ILL 2ILM 2ILN 2ILP 2ILR 2ILT 2ILU 2ILV 2ILY 2ILZ 2IM0 2IM1 2IM2 2IM3 2IM5 2IM8 2IM9 2IMA 2IMB 2IMC 2IMD 2IME 2IMF 2IMG 2IMH 2IMI 2IMJ 2IMK 2IML 2IMM 2IMN 2IMO 2IMP 2IMQ 2IMR 2IMS 2IMT 2IMZ 2IN0 2IN3 2IN4 2IN5 2IN6 2IN8 2IN9 2INB 2INC 2IND 2INE 2INF 2ING 2INN 2INP 2INR 2INS 2INT 2INU 2INV 2INW 2INX 2INY 2INZ 2IO0 2IO1 2IO2 2IO3 2IO4 2IO5 2IO6 2IO7 2IO8 2IO9 2IOA 2IOB 2IOC 2IOD 2IOF 2IOG 2IOH 2IOI 2IOJ 2IOK 2IOL 2IOM 2ION 2IOO 2IOP 2IOQ 2IOR 2IOS 2IOT 2IOU 2IOV 2IOY 2IP1 2IP2 2IP4 2IP6 2IPB 2IPC 2IPF 2IPG 2IPH 2IPI 2IPJ 2IPK 2IPL 2IPM 2IPN 2IPO 2IPP 2IPQ 2IPR 2IPS 2IPT 2IPU 2IPW 2IPX 2IPZ 2IQ0 2IQ1 2IQ5 2IQ6 2IQ7 2IQ9 2IQA 2IQC 2IQD 2IQF 2IQG 2IQH 2IQI 2IQJ 2IQQ 2IQT 2IQX 2IQY 2IRM 2IRP 2IRT 2IRU 2IRV 2IRW 2IRX 2IRY 2IRZ 2IS0 2IS3 2IS5 2IS7 2IS8 2IS9 2ISA 2ISB 2ISC 2ISD 2ISE 2ISF 2ISG 2ISH 2ISI 2ISJ 2ISK 2ISL 2ISM 2ISN 2ISQ 2ISS 2IST 2ISV 2ISW 2ISY 2IT1 2IT2 2IT3 2IT4 2IT5 2IT6 2IT9 2ITB 2ITC 2ITD 2ITE 2ITF 2ITG 2ITJ 2ITK 2ITM 2ITN 2ITO 2ITP 2ITQ 2ITT 2ITU 2ITV 2ITW 2ITX 2ITY 2ITZ 2IU0 2IU1 2IU2 2IU3 2IU4 2IU5 2IU6 2IU7 2IU8 2IU9 2IUA 2IUB 2IUC 2IUF 2IUG 2IUH 2IUI 2IUJ 2IUK 2IUL 2IUM 2IUN 2IUO 2IUP 2IUQ 2IUR 2IUS 2IUT 2IUU 2IUV 2IUW 2IUX 2IUY 2IUZ 2IV0 2IV1 2IV2 2IV3 2IV7 2IV8 2IV9 2IVB 2IVD 2IVE 2IVF 2IVG 2IVI 2IVJ 2IVM 2IVN 2IVO 2IVP 2IVQ 2IVS 2IVT 2IVU 2IVV 2IVX 2IVY 2IVZ 2IW0 2IW1 2IW2 2IW3 2IW4 2IW5 2IW6 2IW8 2IW9 2IWA 2IWB 2IWC 2IWD 2IWE 2IWF 2IWG 2IWH 2IWI 2IWK 2IWL 2IWM 2IWN 2IWO 2IWP 2IWQ 2IWR 2IWS 2IWT 2IWU 2IWV 2IWW 2IWX 2IWY 2IWZ 2IX0 2IX2 2IX3 2IX4 2IX5 2IX6 2IX7 2IX9 2IXA 2IXB 2IXC 2IXD 2IXE 2IXF 2IXG 2IXH 2IXI 2IXJ 2IXK 2IXL 2IXM 2IXN 2IXO 2IXP 2IXR 2IXS 2IXT 2IXU 2IXV 2IY0 2IY1 2IY2 2IY4 2IY6 2IY7 2IY8 2IY9 2IYA 2IYB 2IYC 2IYD 2IYE 2IYF 2IYG 2IYI 2IYJ 2IYK 2IYL 2IYN 2IYO 2IYP 2IYQ 2IYR 2IYS 2IYT 2IYU 2IYV 2IYW 2IYX 2IYY 2IYZ 2IZ0 2IZ1 2IZ5 2IZ6 2IZ7 2IZA 2IZB 2IZC 2IZD 2IZE 2IZF 2IZG 2IZH 2IZI 2IZJ 2IZK 2IZL 2IZO 2IZP 2IZQ 2IZR 2IZS 2IZT 2IZU 2IZV 2IZW 2IZX 2IZY 2IZZ 2J04 2J05 2J06 2J07 2J08 2J09 2J0A 2J0B 2J0D 2J0E 2J0F 2J0G 2J0H 2J0I 2J0J 2J0K 2J0L 2J0M 2J0N 2J0O 2J0P 2J0R 2J0T 2J0U 2J0V 2J0W 2J0X 2J0Y 2J12 2J13 2J14 2J16 2J17 2J18 2J19 2J1A 2J1D 2J1E 2J1G 2J1K 2J1L 2J1M 2J1N 2J1O 2J1P 2J1Q 2J1R 2J1S 2J1T 2J1U 2J1V 2J1W 2J1X 2J1Y 2J1Z 2J20 2J21 2J22 2J23 2J24 2J25 2J27 2J2C 2J2F 2J2I 2J2J 2J2M 2J2P 2J2U 2J2Z 2J30 2J31 2J32 2J33 2J34 2J38 2J3D 2J3E 2J3F 2J3G 2J3H 2J3I 2J3J 2J3K 2J3L 2J3M 2J3N 2J3O 2J3P 2J3Q 2J3R 2J3S 2J3T 2J3U 2J3V 2J3W 2J3X 2J3Z 2J40 2J41 2J42 2J43 2J44 2J45 2J46 2J47 2J49 2J4A 2J4B 2J4C 2J4D 2J4E 2J4F 2J4G 2J4H 2J4I 2J4J 2J4K 2J4L 2J4O 2J4Q 2J4R 2J4S 2J4T 2J4U 2J4W 2J4X 2J4Y 2J4Z 2J50 2J51 2J55 2J56 2J57 2J58 2J59 2J5A 2J5B 2J5C 2J5E 2J5F 2J5G 2J5I 2J5K 2J5L 2J5M 2J5N 2J5Q 2J5R 2J5S 2J5T 2J5U 2J5V 2J5W 2J5X 2J5Y 2J5Z 2J60 2J61 2J62 2J63 2J64 2J65 2J66 2J67 2J68 2J69 2J6A 2J6B 2J6C 2J6E 2J6F 2J6G 2J6H 2J6I 2J6J 2J6K 2J6L 2J6M 2J6O 2J6P 2J6R 2J6V 2J6W 2J6X 2J6Y 2J6Z 2J70 2J71 2J72 2J73 2J74 2J75 2J77 2J78 2J79 2J7A 2J7B 2J7C 2J7D 2J7E 2J7F 2J7G 2J7H 2J7I 2J7J 2J7K 2J7L 2J7M 2J7N 2J7O 2J7P 2J7Q 2J7T 2J7U 2J7V 2J7W 2J7X 2J7Y 2J7Z 2J80 2J82 2J83 2J85 2J86 2J87 2J88 2J89 2J8A 2J8B 2J8C 2J8D 2J8F 2J8G 2J8H 2J8I 2J8K 2J8M 2J8N 2J8O 2J8Q 2J8R 2J8S 2J8T 2J8U 2J8W 2J8X 2J8Y 2J8Z 2J90 2J91 2J92 2J94 2J95 2J96 2J97 2J98 2J9A 2J9B 2J9C 2J9D 2J9E 2J9F 2J9G 2J9H 2J9J 2J9K 2J9L 2J9M 2J9N 2J9O 2J9P 2J9Q 2J9R 2J9T 2J9U 2J9V 2J9W 2J9X 2J9Y 2J9Z 2JA1 2JA2 2JA3 2JA4 2JA9 2JAA 2JAB 2JAC 2JAD 2JAE 2JAF 2JAG 2JAH 2JAI 2JAJ 2JAK 2JAL 2JAM 2JAN 2JAO 2JAP 2JAQ 2JAR 2JAS 2JAT 2JAU 2JAV 2JAW 2JAX 2JAY 2JAZ 2JB0 2JB1 2JB2 2JB3 2JB4 2JB5 2JB6 2JB7 2JB8 2JB9 2JBA 2JBF 2JBG 2JBH 2JBJ 2JBK 2JBL 2JBM 2JBO 2JBP 2JBR 2JBS 2JBT 2JBU 2JBV 2JBW 2JBX 2JBY 2JBZ 2JC0 2JC1 2JC2 2JC3 2JC4 2JC5 2JC6 2JC7 2JC9 2JCA 2JCB 2JCC 2JCD 2JCG 2JCH 2JCJ 2JCK 2JCL 2JCM 2JCN 2JCO 2JCP 2JCQ 2JCR 2JCS 2JCV 2JCW 2JCX 2JCY 2JD0 2JD1 2JD2 2JD3 2JD4 2JD5 2JD6 2JD7 2JD8 2JD9 2JDA 2JDC 2JDD 2JDF 2JDG 2JDH 2JDI 2JDJ 2JDK 2JDL 2JDM 2JDN 2JDO 2JDP 2JDQ 2JDR 2JDS 2JDT 2JDU 2JDV 2JDW 2JDX 2JDY 2JDZ 2JE0 2JE1 2JE2 2JE3 2JE4 2JE5 2JE6 2JE7 2JE8 2JE9 2JEB 2JEC 2JED 2JEE 2JEK 2JEL 2JEM 2JEN 2JEO 2JEP 2JEQ 2JER 2JES 2JET 2JEU 2JEV 2JEW 2JEX 2JEY 2JEZ 2JF0 2JF1 2JF2 2JF3 2JF4 2JF5 2JF6 2JF7 2JF9 2JFA 2JFB 2JFC 2JFD 2JFE 2JFF 2JFG 2JFH 2JFK 2JFL 2JFM 2JFN 2JFO 2JFP 2JFQ 2JFR 2JFS 2JFT 2JFU 2JFV 2JFW 2JFX 2JFY 2JFZ 2JG0 2JG1 2JG2 2JG4 2JG5 2JG6 2JG7 2JG8 2JG9 2JGA 2JGB 2JGC 2JGD 2JGE 2JGF 2JGI 2JGJ 2JGK 2JGL 2JGM 2JGN 2JGO 2JGP 2JGQ 2JGR 2JGS 2JGT 2JGU 2JGV 2JGY 2JGZ 2JH0 2JH1 2JH2 2JH3 2JH5 2JH6 2JH7 2JH8 2JH9 2JHA 2JHC 2JHD 2JHE 2JHF 2JHG 2JHH 2JHI 2JHJ 2JHK 2JHL 2JHM 2JHN 2JHO 2JHP 2JHQ 2JHR 2JHS 2JHT 2JHU 2JHV 2JHW 2JHX 2JHY 2JHZ 2JI0 2JI1 2JI2 2JI3 2JI4 2JI5 2JI6 2JI7 2JI8 2JI9 2JIA 2JIB 2JIC 2JID 2JIE 2JIF 2JIG 2JIH 2JII 2JIJ 2JIK 2JIL 2JIM 2JIN 2JIO 2JIP 2JIQ 2JIR 2JIS 2JIT 2JIU 2JIV 2JIW 2JIX 2JIY 2JIZ 2JJ0 2JJ1 2JJ2 2JJ3 2JJ4 2JJ6 2JJ7 2JJ8 2JJ9 2JJB 2JJC 2JJD 2JJE 2JJF 2JJG 2JJH 2JJI 2JJJ 2JJK 2JJL 2JJM 2JJN 2JJO 2JJP 2JJQ 2JJR 2JJS 2JJT 2JJU 2JJV 2JJW 2JJX 2JJY 2JJZ 2JK0 2JK1 2JK2 2JK3 2JK4 2JK5 2JK6 2JK7 2JK8 2JK9 2JKA 2JKB 2JKC 2JKD 2JKE 2JKF 2JKG 2JKH 2JKI 2JKJ 2JKK 2JKL 2JKM 2JKN 2JKO 2JKP 2JKQ 2JKR 2JKS 2JKT 2JKU 2JKV 2JKW 2JKX 2JKY 2JKZ 2JL1 2JL4 2JL9 2JLA 2JLB 2JLC 2JLD 2JLE 2JLF 2JLH 2JLI 2JLJ 2JLL 2JLM 2JLN 2JLP 2JLQ 2JLR 2JLS 2JXR 2KAI 2KAU 2KCE 2KI5 2KIN 2KMB 2KNT 2L78 2LAL 2LAO 2LBD 2LBP 2LDB 2LDX 2LGS 2LH1 2LH2 2LH3 2LH5 2LH6 2LH7 2LHB 2LHM 2LIG 2LIP 2LIS 2LIV 2LJR 2LKF 2LPR 2LTN 2LVE 2LYM 2LYN 2LYO 2LYZ 2LZ2 2LZH 2LZM 2LZT 2MAD 2MAN 2MAS 2MAT 2MBR 2MBW 2MCG 2MCM 2MCP 2MEA 2MEB 2MEC 2MED 2MEE 2MEF 2MEG 2MEH 2MEI 2MEV 2MGA 2MGB 2MGC 2MGD 2MGE 2MGF 2MGG 2MGH 2MGI 2MGJ 2MGK 2MGL 2MGM 2MHA 2MHB 2MHR 2MIB 2MIN 2MIP 2MJP 2MLL 2MLT 2MNR 2MPA 2MPR 2MS2 2MSB 2MSI 2MSJ 2MSP 2MTA 2MUC 2MYA 2MYB 2MYC 2MYD 2MYE 2MYS 2NAC 2NAD 2NAP 2NCD 2NCK 2NG1 2NGR 2NIP 2NL9 2NLA 2NLB 2NLC 2NLD 2NLE 2NLF 2NLG 2NLH 2NLI 2NLJ 2NLK 2NLO 2NLP 2NLQ 2NLR 2NLS 2NLV 2NLX 2NLY 2NLZ 2NM0 2NM1 2NM2 2NM3 2NML 2NMM 2NMN 2NMO 2NMP 2NMR 2NMS 2NMT 2NMU 2NMX 2NMY 2NMZ 2NN1 2NN2 2NN3 2NN4 2NN5 2NN6 2NN7 2NN8 2NNA 2NNB 2NNC 2NND 2NNE 2NNF 2NNG 2NNH 2NNI 2NNJ 2NNK 2NNL 2NNN 2NNO 2NNP 2NNQ 2NNR 2NNS 2NNU 2NNV 2NNW 2NNX 2NO0 2NO1 2NO2 2NO3 2NO4 2NO5 2NO6 2NO7 2NO9 2NOA 2NOD 2NOG 2NOJ 2NOM 2NOO 2NOP 2NOS 2NOT 2NOV 2NOW 2NOX 2NOY 2NP0 2NP1 2NP3 2NP5 2NP8 2NP9 2NPA 2NPC 2NPD 2NPE 2NPF 2NPG 2NPH 2NPI 2NPJ 2NPK 2NPM 2NPN 2NPO 2NPP 2NPQ 2NPS 2NPT 2NPX 2NQ2 2NQ3 2NQ5 2NQ6 2NQ7 2NQ8 2NQA 2NQC 2NQD 2NQG 2NQH 2NQI 2NQK 2NQL 2NQM 2NQN 2NQO 2NQQ 2NQR 2NQS 2NQT 2NQU 2NQV 2NQW 2NQX 2NQY 2NQZ 2NR4 2NR5 2NR6 2NR7 2NR9 2NRB 2NRC 2NRD 2NRF 2NRH 2NRJ 2NRK 2NRL 2NRM 2NRN 2NRO 2NRP 2NRQ 2NRR 2NRS 2NRT 2NRU 2NRV 2NRW 2NRX 2NRY 2NRZ 2NS0 2NS1 2NS2 2NS6 2NS7 2NS8 2NS9 2NSA 2NSB 2NSC 2NSD 2NSE 2NSF 2NSG 2NSH 2NSI 2NSJ 2NSL 2NSM 2NSN 2NSO 2NSP 2NSQ 2NSR 2NSS 2NST 2NSX 2NSY 2NSZ 2NT0 2NT1 2NT2 2NT3 2NT4 2NT6 2NT7 2NT8 2NT9 2NTA 2NTB 2NTD 2NTE 2NTF 2NTG 2NTH 2NTI 2NTJ 2NTK 2NTL 2NTM 2NTN 2NTO 2NTP 2NTQ 2NTR 2NTS 2NTT 2NTU 2NTV 2NTW 2NTX 2NTY 2NU0 2NU1 2NU2 2NU3 2NU4 2NU5 2NU6 2NU7 2NU8 2NU9 2NUA 2NUB 2NUC 2NUD 2NUH 2NUI 2NUJ 2NUK 2NUL 2NUM 2NUN 2NUO 2NUP 2NUT 2NUU 2NUV 2NUW 2NUX 2NUY 2NUZ 2NV0 2NV1 2NV2 2NV4 2NV5 2NV6 2NV7 2NV9 2NVA 2NVB 2NVC 2NVD 2NVE 2NVF 2NVG 2NVH 2NVK 2NVL 2NVM 2NVN 2NVO 2NVP 2NVU 2NVV 2NVW 2NVY 2NW0 2NW2 2NW3 2NW4 2NW6 2NW7 2NW8 2NW9 2NWA 2NWB 2NWC 2NWD 2NWF 2NWG 2NWH 2NWI 2NWJ 2NWL 2NWN 2NWO 2NWP 2NWQ 2NWR 2NWS 2NWU 2NWV 2NWW 2NWX 2NWY 2NWZ 2NX0 2NX1 2NX2 2NX3 2NX4 2NX5 2NX8 2NX9 2NXA 2NXB 2NXC 2NXD 2NXE 2NXF 2NXG 2NXH 2NXI 2NXJ 2NXL 2NXM 2NXN 2NXO 2NXP 2NXQ 2NXR 2NXS 2NXT 2NXV 2NXW 2NXX 2NXY 2NXZ 2NY0 2NY1 2NY2 2NY3 2NY4 2NY5 2NY6 2NY7 2NYA 2NYB 2NYC 2NYD 2NYE 2NYF 2NYG 2NYH 2NYI 2NYJ 2NYK 2NYL 2NYM 2NYN 2NYP 2NYQ 2NYR 2NYS 2NYT 2NYU 2NYV 2NYX 2NYY 2NYZ 2NZ0 2NZ1 2NZ2 2NZ5 2NZ6 2NZ7 2NZ8 2NZ9 2NZA 2NZC 2NZE 2NZF 2NZH 2NZI 2NZJ 2NZL 2NZM 2NZO 2NZT 2NZU 2NZV 2NZW 2NZX 2NZY 2O01 2O02 2O03 2O04 2O05 2O06 2O07 2O08 2O09 2O0A 2O0B 2O0C 2O0D 2O0E 2O0G 2O0H 2O0I 2O0J 2O0K 2O0L 2O0M 2O0O 2O0P 2O0Q 2O0R 2O0T 2O0U 2O0V 2O0W 2O0X 2O0Y 2O0Z 2O11 2O12 2O14 2O15 2O16 2O17 2O18 2O1A 2O1B 2O1C 2O1D 2O1E 2O1F 2O1G 2O1H 2O1J 2O1K 2O1L 2O1M 2O1N 2O1O 2O1P 2O1Q 2O1S 2O1T 2O1U 2O1V 2O1W 2O1X 2O1Z 2O20 2O23 2O24 2O25 2O26 2O27 2O28 2O29 2O2A 2O2B 2O2C 2O2D 2O2E 2O2G 2O2H 2O2I 2O2J 2O2K 2O2L 2O2P 2O2Q 2O2R 2O2S 2O2T 2O2U 2O2V 2O2X 2O2Y 2O2Z 2O30 2O31 2O34 2O35 2O36 2O38 2O39 2O3A 2O3B 2O3C 2O3E 2O3F 2O3G 2O3H 2O3I 2O3J 2O3K 2O3L 2O3O 2O3P 2O3Q 2O3R 2O3S 2O3T 2O3U 2O3Z 2O40 2O42 2O48 2O4C 2O4D 2O4G 2O4H 2O4J 2O4K 2O4L 2O4M 2O4N 2O4P 2O4Q 2O4R 2O4S 2O4T 2O4U 2O4V 2O4W 2O4X 2O4Z 2O50 2O51 2O52 2O53 2O55 2O56 2O57 2O58 2O5A 2O5B 2O5D 2O5F 2O5G 2O5H 2O5K 2O5L 2O5M 2O5N 2O5O 2O5P 2O5Q 2O5R 2O5S 2O5T 2O5U 2O5V 2O5W 2O5X 2O5Y 2O5Z 2O60 2O62 2O63 2O64 2O65 2O66 2O67 2O68 2O69 2O6A 2O6B 2O6C 2O6D 2O6E 2O6F 2O6H 2O6I 2O6K 2O6L 2O6N 2O6P 2O6Q 2O6R 2O6S 2O6T 2O6U 2O6V 2O6W 2O6X 2O6Y 2O70 2O71 2O72 2O73 2O74 2O78 2O79 2O7A 2O7B 2O7C 2O7D 2O7E 2O7F 2O7G 2O7H 2O7I 2O7K 2O7L 2O7M 2O7N 2O7O 2O7P 2O7Q 2O7R 2O7S 2O7T 2O7U 2O7V 2O84 2O85 2O86 2O87 2O88 2O89 2O8A 2O8G 2O8H 2O8I 2O8J 2O8L 2O8M 2O8N 2O8O 2O8P 2O8Q 2O8R 2O8S 2O8T 2O8U 2O8V 2O8W 2O8X 2O8Y 2O90 2O92 2O94 2O95 2O96 2O97 2O98 2O99 2O9A 2O9B 2O9C 2O9D 2O9E 2O9F 2O9G 2O9I 2O9J 2O9K 2O9O 2O9P 2O9Q 2O9R 2O9S 2O9T 2O9U 2O9V 2O9X 2O9Z 2OA0 2OA1 2OA2 2OA5 2OA6 2OA7 2OA9 2OAC 2OAD 2OAE 2OAF 2OAG 2OAH 2OAI 2OAJ 2OAL 2OAM 2OAN 2OAP 2OAQ 2OAR 2OAS 2OAT 2OAU 2OAW 2OAX 2OAY 2OAZ 2OB0 2OB1 2OB2 2OB3 2OB4 2OB5 2OB9 2OBA 2OBB 2OBC 2OBD 2OBE 2OBF 2OBG 2OBH 2OBI 2OBJ 2OBK 2OBL 2OBM 2OBN 2OBO 2OBP 2OBQ 2OBR 2OBS 2OBT 2OBV 2OBX 2OBY 2OC0 2OC1 2OC2 2OC3 2OC4 2OC5 2OC6 2OC7 2OC8 2OC9 2OCA 2OCB 2OCC 2OCD 2OCE 2OCF 2OCG 2OCH 2OCI 2OCJ 2OCK 2OCL 2OCP 2OCS 2OCT 2OCU 2OCV 2OCX 2OCY 2OCZ 2OD0 2OD2 2OD3 2OD4 2OD5 2OD6 2OD7 2OD8 2OD9 2ODA 2ODB 2ODE 2ODF 2ODH 2ODJ 2ODK 2ODL 2ODM 2ODN 2ODO 2ODP 2ODQ 2ODR 2ODT 2ODU 2ODV 2ODW 2ODY 2OE0 2OE1 2OE2 2OE3 2OE4 2OE7 2OE9 2OEA 2OEB 2OEC 2OEE 2OEF 2OEG 2OEI 2OEJ 2OEK 2OEL 2OEM 2OEN 2OEO 2OEP 2OEQ 2OER 2OES 2OEV 2OEW 2OEX 2OEZ 2OF0 2OF1 2OF2 2OF3 2OF4 2OF5 2OF7 2OF8 2OF9 2OFA 2OFB 2OFC 2OFD 2OFE 2OFF 2OFJ 2OFK 2OFM 2OFO 2OFP 2OFR 2OFS 2OFU 2OFV 2OFW 2OFX 2OFY 2OFZ 2OG1 2OG2 2OG3 2OG4 2OG5 2OG6 2OG7 2OG8 2OG9 2OGA 2OGB 2OGD 2OGE 2OGF 2OGG 2OGI 2OGJ 2OGK 2OGQ 2OGR 2OGS 2OGT 2OGU 2OGV 2OGW 2OGX 2OGY 2OGZ 2OH0 2OH1 2OH3 2OH4 2OH5 2OH6 2OH7 2OH8 2OH9 2OHA 2OHB 2OHC 2OHD 2OHE 2OHF 2OHG 2OHH 2OHI 2OHJ 2OHK 2OHL 2OHM 2OHN 2OHO 2OHP 2OHQ 2OHR 2OHS 2OHT 2OHU 2OHV 2OHW 2OHX 2OHY 2OI0 2OI2 2OI4 2OI5 2OI6 2OI7 2OI8 2OI9 2OIB 2OIC 2OID 2OIE 2OIF 2OIG 2OIK 2OIL 2OIN 2OIP 2OIQ 2OIT 2OIV 2OIW 2OIX 2OIZ 2OJ1 2OJ4 2OJ5 2OJ6 2OJ9 2OJE 2OJF 2OJG 2OJH 2OJI 2OJJ 2OJK 2OJL 2OJP 2OJQ 2OJR 2OJT 2OJU 2OJV 2OJW 2OJX 2OJY 2OJZ 2OK1 2OK2 2OK3 2OK4 2OK5 2OK6 2OK7 2OK8 2OK9 2OKA 2OKB 2OKC 2OKD 2OKE 2OKF 2OKG 2OKH 2OKI 2OKJ 2OKK 2OKL 2OKM 2OKN 2OKO 2OKQ 2OKR 2OKT 2OKU 2OKV 2OKW 2OKX 2OKY 2OKZ 2OL0 2OL1 2OL2 2OL3 2OL4 2OL5 2OL6 2OL7 2OL8 2OL9 2OLA 2OLB 2OLC 2OLD 2OLE 2OLG 2OLH 2OLI 2OLJ 2OLK 2OLM 2OLN 2OLO 2OLP 2OLQ 2OLR 2OLS 2OLT 2OLU 2OLV 2OLW 2OLX 2OLY 2OLZ 2OM0 2OM1 2OM2 2OM5 2OM6 2OM9 2OMA 2OMB 2OMD 2OME 2OMF 2OMG 2OMH 2OMI 2OMK 2OML 2OMM 2OMN 2OMO 2OMP 2OMQ 2OMT 2OMU 2OMV 2OMW 2OMX 2OMY 2OMZ 2ON3 2ON5 2ON6 2ON7 2ON8 2ON9 2ONA 2ONB 2ONC 2OND 2ONE 2ONF 2ONG 2ONH 2ONI 2ONJ 2ONK 2ONL 2ONM 2ONN 2ONO 2ONP 2ONQ 2ONR 2ONS 2ONT 2ONU 2ONV 2ONW 2ONX 2ONY 2ONZ 2OO0 2OO1 2OO2 2OO3 2OO4 2OO5 2OO6 2OO7 2OO8 2OO9 2OOA 2OOB 2OOC 2OOD 2OOE 2OOF 2OOG 2OOH 2OOI 2OOJ 2OOK 2OOL 2OOQ 2OOR 2OOS 2OOT 2OOV 2OOW 2OOX 2OOY 2OOZ 2OP0 2OP1 2OP2 2OP3 2OP4 2OP5 2OP6 2OP8 2OP9 2OPA 2OPB 2OPC 2OPD 2OPE 2OPG 2OPH 2OPI 2OPJ 2OPK 2OPL 2OPM 2OPN 2OPO 2OPP 2OPQ 2OPR 2OPS 2OPT 2OPW 2OPX 2OPY 2OPZ 2OQ0 2OQ1 2OQ2 2OQ5 2OQ6 2OQ7 2OQA 2OQB 2OQC 2OQD 2OQE 2OQF 2OQG 2OQH 2OQI 2OQJ 2OQK 2OQL 2OQM 2OQN 2OQO 2OQQ 2OQR 2OQT 2OQU 2OQV 2OQW 2OQX 2OQY 2OQZ 2OR0 2OR2 2OR3 2OR4 2OR7 2OR8 2OR9 2ORA 2ORB 2ORD 2ORE 2ORI 2ORJ 2ORK 2ORM 2ORO 2ORP 2ORQ 2ORR 2ORS 2ORT 2ORV 2ORW 2ORX 2ORY 2ORZ 2OS0 2OS1 2OS2 2OS3 2OS5 2OS7 2OS8 2OS9 2OSA 2OSB 2OSC 2OSD 2OSE 2OSF 2OSH 2OSL 2OSM 2OSN 2OSO 2OSS 2OSU 2OSV 2OSW 2OSX 2OSY 2OSZ 2OT0 2OT1 2OT3 2OT4 2OT5 2OT7 2OT8 2OT9 2OTA 2OTB 2OTC 2OTD 2OTE 2OTF 2OTG 2OTH 2OTM 2OTN 2OTO 2OTP 2OTT 2OTU 2OTV 2OTW 2OTX 2OTY 2OTZ 2OU0 2OU1 2OU2 2OU3 2OU4 2OU5 2OU6 2OU7 2OU9 2OUA 2OUB 2OUC 2OUD 2OUG 2OUH 2OUI 2OUJ 2OUK 2OUL 2OUM 2OUN 2OUO 2OUP 2OUQ 2OUR 2OUS 2OUU 2OUV 2OUW 2OUX 2OUY 2OUZ 2OV0 2OV1 2OV2 2OV3 2OV4 2OV5 2OV7 2OV8 2OV9 2OVA 2OVB 2OVC 2OVD 2OVE 2OVF 2OVG 2OVH 2OVI 2OVJ 2OVL 2OVM 2OVO 2OVP 2OVQ 2OVR 2OVS 2OVU 2OVV 2OVW 2OVX 2OVY 2OVZ 2OW0 2OW1 2OW2 2OW3 2OW4 2OW6 2OW7 2OW9 2OWA 2OWB 2OWC 2OWD 2OWE 2OWF 2OWG 2OWH 2OWJ 2OWK 2OWL 2OWM 2OWN 2OWP 2OWQ 2OWR 2OWS 2OWT 2OWU 2OWV 2OWW 2OWX 2OWY 2OWZ 2OX0 2OX1 2OX3 2OX4 2OX5 2OX6 2OX7 2OX8 2OX9 2OXB 2OXC 2OXD 2OXE 2OXF 2OXG 2OXH 2OXI 2OXJ 2OXK 2OXL 2OXN 2OXO 2OXP 2OXQ 2OXR 2OXS 2OXT 2OXU 2OXW 2OXX 2OXY 2OXZ 2OY0 2OY1 2OY2 2OY3 2OY4 2OY5 2OY7 2OY8 2OY9 2OYA 2OYB 2OYC 2OYE 2OYF 2OYH 2OYI 2OYK 2OYL 2OYM 2OYN 2OYO 2OYP 2OYR 2OYS 2OYU 2OYY 2OYZ 2OZ0 2OZ1 2OZ2 2OZ3 2OZ4 2OZ5 2OZ6 2OZ7 2OZ8 2OZ9 2OZA 2OZE 2OZF 2OZG 2OZH 2OZJ 2OZK 2OZL 2OZN 2OZO 2OZP 2OZQ 2OZR 2OZT 2OZU 2OZV 2OZY 2OZZ 2P02 2P04 2P05 2P06 2P08 2P09 2P0A 2P0B 2P0C 2P0D 2P0E 2P0F 2P0G 2P0H 2P0I 2P0K 2P0L 2P0M 2P0N 2P0O 2P0R 2P0S 2P0T 2P0U 2P0V 2P0W 2P0Y 2P10 2P11 2P12 2P13 2P14 2P15 2P16 2P17 2P18 2P19 2P1A 2P1B 2P1C 2P1D 2P1E 2P1F 2P1G 2P1H 2P1I 2P1J 2P1L 2P1M 2P1N 2P1O 2P1P 2P1Q 2P1R 2P1S 2P1T 2P1U 2P1V 2P1W 2P1X 2P1Y 2P1Z 2P20 2P22 2P23 2P24 2P25 2P26 2P27 2P28 2P2A 2P2B 2P2C 2P2D 2P2E 2P2F 2P2G 2P2H 2P2I 2P2J 2P2K 2P2L 2P2M 2P2N 2P2O 2P2Q 2P2S 2P2T 2P2U 2P2V 2P2W 2P2X 2P2Y 2P2Z 2P30 2P31 2P32 2P33 2P34 2P35 2P37 2P38 2P39 2P3A 2P3B 2P3C 2P3D 2P3E 2P3F 2P3G 2P3H 2P3I 2P3J 2P3K 2P3L 2P3N 2P3O 2P3P 2P3Q 2P3S 2P3T 2P3U 2P3V 2P3W 2P3X 2P3Y 2P3Z 2P40 2P41 2P42 2P43 2P44 2P45 2P46 2P47 2P48 2P49 2P4A 2P4B 2P4D 2P4E 2P4F 2P4G 2P4H 2P4I 2P4J 2P4K 2P4M 2P4O 2P4P 2P4Q 2P4R 2P4S 2P4T 2P4U 2P4V 2P4W 2P4X 2P4Y 2P4Z 2P50 2P51 2P52 2P53 2P54 2P55 2P56 2P57 2P58 2P59 2P5B 2P5C 2P5D 2P5E 2P5F 2P5I 2P5K 2P5M 2P5N 2P5P 2P5Q 2P5R 2P5S 2P5T 2P5U 2P5V 2P5W 2P5X 2P5Y 2P5Z 2P61 2P62 2P63 2P64 2P65 2P67 2P68 2P69 2P6A 2P6B 2P6C 2P6D 2P6E 2P6F 2P6G 2P6H 2P6I 2P6K 2P6L 2P6M 2P6N 2P6O 2P6P 2P6S 2P6T 2P6U 2P6V 2P6W 2P6X 2P6Y 2P6Z 2P70 2P71 2P72 2P73 2P74 2P75 2P76 2P77 2P78 2P79 2P7A 2P7G 2P7H 2P7I 2P7J 2P7K 2P7L 2P7M 2P7N 2P7O 2P7P 2P7Q 2P7S 2P7T 2P7U 2P7V 2P7Z 2P82 2P83 2P84 2P85 2P86 2P87 2P88 2P8B 2P8C 2P8E 2P8G 2P8H 2P8I 2P8J 2P8L 2P8M 2P8N 2P8O 2P8P 2P8Q 2P8R 2P8S 2P8T 2P8U 2P8V 2P90 2P91 2P93 2P94 2P95 2P97 2P98 2P99 2P9A 2P9B 2P9C 2P9D 2P9E 2P9F 2P9G 2P9H 2P9I 2P9J 2P9K 2P9L 2P9M 2P9N 2P9P 2P9Q 2P9R 2P9S 2P9T 2P9U 2P9V 2P9W 2P9X 2P9Y 2P9Z 2PA0 2PA1 2PA2 2PA3 2PA4 2PA5 2PA6 2PA7 2PA8 2PAA 2PAB 2PAD 2PAE 2PAF 2PAG 2PAH 2PAJ 2PAK 2PAL 2PAM 2PAN 2PAQ 2PAR 2PAU 2PAV 2PAW 2PAX 2PB0 2PB1 2PB2 2PB4 2PB5 2PB6 2PB7 2PB8 2PB9 2PBC 2PBD 2PBE 2PBF 2PBG 2PBH 2PBI 2PBJ 2PBK 2PBL 2PBN 2PBO 2PBP 2PBQ 2PBR 2PBW 2PBX 2PBY 2PBZ 2PC0 2PC1 2PC2 2PC4 2PC5 2PC6 2PC8 2PC9 2PCA 2PCB 2PCC 2PCD 2PCE 2PCG 2PCH 2PCI 2PCJ 2PCK 2PCL 2PCM 2PCN 2PCP 2PCQ 2PCR 2PCS 2PCU 2PCX 2PCY 2PD0 2PD1 2PD2 2PD3 2PD4 2PD5 2PD6 2PD7 2PD8 2PD9 2PDA 2PDB 2PDC 2PDF 2PDG 2PDH 2PDI 2PDJ 2PDK 2PDL 2PDM 2PDN 2PDO 2PDP 2PDQ 2PDR 2PDT 2PDU 2PDW 2PDX 2PDY 2PE0 2PE1 2PE2 2PE3 2PE4 2PE6 2PE7 2PE8 2PEB 2PEC 2PED 2PEE 2PEF 2PEG 2PEH 2PEI 2PEJ 2PEK 2PEL 2PEM 2PEN 2PEO 2PEQ 2PER 2PES 2PET 2PEV 2PEX 2PEY 2PEZ 2PF0 2PF1 2PF2 2PF4 2PF5 2PF6 2PF8 2PFB 2PFC 2PFD 2PFE 2PFF 2PFG 2PFH 2PFI 2PFK 2PFL 2PFM 2PFR 2PFS 2PFT 2PFV 2PFW 2PFX 2PFY 2PFZ 2PG0 2PG1 2PG2 2PG3 2PG4 2PG5 2PG6 2PG7 2PG8 2PGA 2PGB 2PGC 2PGD 2PGE 2PGF 2PGG 2PGH 2PGI 2PGJ 2PGK 2PGL 2PGN 2PGO 2PGQ 2PGR 2PGS 2PGT 2PGV 2PGW 2PGX 2PGY 2PGZ 2PH0 2PH1 2PH3 2PH4 2PH5 2PH6 2PH7 2PH8 2PH9 2PHA 2PHB 2PHC 2PHD 2PHF 2PHH 2PHI 2PHK 2PHL 2PHM 2PHN 2PHO 2PHP 2PHR 2PHT 2PHU 2PHW 2PHX 2PHY 2PHZ 2PI2 2PI3 2PI6 2PI7 2PI8 2PIA 2PIC 2PID 2PIE 2PIF 2PIG 2PIH 2PII 2PIJ 2PIL 2PIM 2PIN 2PIO 2PIP 2PIQ 2PIR 2PIT 2PIU 2PIV 2PIW 2PIX 2PIY 2PIZ 2PJ0 2PJ1 2PJ2 2PJ3 2PJ4 2PJ5 2PJ6 2PJ7 2PJ8 2PJ9 2PJA 2PJB 2PJC 2PJD 2PJJ 2PJL 2PJO 2PJQ 2PJS 2PJT 2PJU 2PJW 2PJY 2PJZ 2PK0 2PK2 2PK3 2PK4 2PK5 2PK6 2PK7 2PK8 2PK9 2PKA 2PKC 2PKD 2PKE 2PKF 2PKG 2PKH 2PKK 2PKL 2PKM 2PKN 2PKO 2PKP 2PKQ 2PKR 2PKS 2PKT 2PKW 2PKX 2PKY 2PL0 2PL1 2PL2 2PL3 2PL5 2PL6 2PL7 2PL9 2PLA 2PLC 2PLF 2PLG 2PLH 2PLI 2PLJ 2PLK 2PLL 2PLM 2PLN 2PLQ 2PLR 2PLS 2PLT 2PLU 2PLV 2PLW 2PLX 2PLZ 2PM1 2PM4 2PM5 2PM6 2PM7 2PM8 2PM9 2PMA 2PMB 2PMC 2PMD 2PME 2PMF 2PMH 2PMI 2PMJ 2PMK 2PML 2PMN 2PMO 2PMP 2PMQ 2PMR 2PMS 2PMT 2PMU 2PMV 2PMW 2PMY 2PMZ 2PN0 2PN1 2PN2 2PN5 2PN6 2PN7 2PN8 2PNC 2PND 2PNE 2PNF 2PNH 2PNJ 2PNK 2PNL 2PNM 2PNN 2PNO 2PNQ 2PNR 2PNS 2PNT 2PNU 2PNV 2PNW 2PNX 2PNY 2PNZ 2PO0 2PO2 2PO3 2PO4 2PO5 2PO6 2PO7 2POB 2POC 2POD 2POE 2POF 2POG 2POH 2POI 2POK 2POL 2POM 2POO 2POP 2POQ 2POR 2POS 2POT 2POU 2POV 2POW 2POX 2POY 2POZ 2PP0 2PP1 2PP3 2PP6 2PP7 2PP8 2PP9 2PPA 2PPC 2PPD 2PPE 2PPF 2PPG 2PPI 2PPL 2PPN 2PPO 2PPP 2PPQ 2PPS 2PPT 2PPV 2PPW 2PPX 2PPY 2PQ0 2PQ2 2PQ3 2PQ5 2PQ6 2PQ7 2PQ8 2PQ9 2PQA 2PQB 2PQC 2PQD 2PQF 2PQG 2PQI 2PQJ 2PQK 2PQL 2PQM 2PQN 2PQQ 2PQR 2PQS 2PQT 2PQV 2PQW 2PQX 2PQY 2PQZ 2PR0 2PR1 2PR2 2PR3 2PR4 2PR5 2PR6 2PR7 2PR8 2PR9 2PRB 2PRC 2PRD 2PRE 2PRG 2PRH 2PRI 2PRJ 2PRK 2PRL 2PRM 2PRN 2PRO 2PRQ 2PRR 2PRS 2PRV 2PRX 2PRY 2PRZ 2PS0 2PS1 2PS2 2PS3 2PS4 2PS5 2PS6 2PS7 2PS8 2PS9 2PSB 2PSD 2PSE 2PSF 2PSG 2PSH 2PSJ 2PSM 2PSN 2PSO 2PSP 2PSQ 2PSR 2PSS 2PST 2PSU 2PSV 2PSW 2PSX 2PSY 2PSZ 2PT0 2PT1 2PT2 2PT3 2PT5 2PT6 2PT7 2PT9 2PTC 2PTD 2PTF 2PTG 2PTH 2PTK 2PTM 2PTN 2PTQ 2PTR 2PTS 2PTT 2PTU 2PTV 2PTW 2PTX 2PTY 2PTZ 2PU0 2PU1 2PU2 2PU3 2PU4 2PU5 2PU7 2PU8 2PU9 2PUH 2PUI 2PUJ 2PUK 2PUL 2PUM 2PUN 2PUO 2PUP 2PUQ 2PUR 2PUS 2PUT 2PUU 2PUV 2PUW 2PUX 2PUY 2PUZ 2PV0 2PV1 2PV2 2PV3 2PV4 2PV7 2PV9 2PVA 2PVB 2PVC 2PVD 2PVE 2PVF 2PVG 2PVH 2PVJ 2PVK 2PVL 2PVM 2PVN 2PVO 2PVP 2PVQ 2PVR 2PVS 2PVT 2PVU 2PVV 2PVW 2PVX 2PVY 2PVZ 2PW0 2PW1 2PW2 2PW3 2PW5 2PW6 2PW7 2PW8 2PW9 2PWA 2PWB 2PWC 2PWD 2PWE 2PWF 2PWG 2PWH 2PWJ 2PWL 2PWM 2PWN 2PWO 2PWP 2PWQ 2PWR 2PWS 2PWU 2PWV 2PWW 2PWX 2PWY 2PWZ 2PX0 2PX1 2PX2 2PX3 2PX4 2PX5 2PX6 2PX7 2PX8 2PXA 2PXC 2PXH 2PXJ 2PXR 2PXS 2PXW 2PXX 2PXY 2PXZ 2PY0 2PY2 2PY3 2PY4 2PY6 2PY7 2PY8 2PYA 2PYB 2PYC 2PYD 2PYE 2PYF 2PYG 2PYH 2PYI 2PYK 2PYM 2PYN 2PYP 2PYQ 2PYR 2PYS 2PYT 2PYU 2PYW 2PYX 2PYY 2PYZ 2PZ0 2PZ1 2PZ5 2PZ8 2PZ9 2PZA 2PZB 2PZD 2PZE 2PZF 2PZG 2PZH 2PZI 2PZJ 2PZK 2PZL 2PZM 2PZN 2PZP 2PZR 2PZT 2PZU 2PZV 2PZW 2PZX 2PZY 2PZZ 2Q00 2Q01 2Q02 2Q03 2Q04 2Q05 2Q06 2Q07 2Q08 2Q09 2Q0A 2Q0B 2Q0C 2Q0D 2Q0E 2Q0F 2Q0G 2Q0H 2Q0I 2Q0J 2Q0K 2Q0L 2Q0M 2Q0N 2Q0O 2Q0Q 2Q0R 2Q0S 2Q0T 2Q0U 2Q0V 2Q0X 2Q0Y 2Q0Z 2Q11 2Q12 2Q13 2Q14 2Q15 2Q16 2Q17 2Q18 2Q19 2Q1A 2Q1B 2Q1C 2Q1D 2Q1E 2Q1F 2Q1H 2Q1J 2Q1K 2Q1L 2Q1M 2Q1N 2Q1P 2Q1Q 2Q1S 2Q1T 2Q1U 2Q1V 2Q1W 2Q1X 2Q1Y 2Q1Z 2Q20 2Q21 2Q22 2Q24 2Q27 2Q28 2Q29 2Q2A 2Q2B 2Q2C 2Q2E 2Q2F 2Q2G 2Q2H 2Q2I 2Q2J 2Q2L 2Q2M 2Q2N 2Q2O 2Q2P 2Q2Q 2Q2R 2Q2V 2Q2W 2Q2X 2Q2Y 2Q2Z 2Q30 2Q31 2Q32 2Q33 2Q34 2Q35 2Q36 2Q37 2Q38 2Q39 2Q3A 2Q3B 2Q3C 2Q3D 2Q3E 2Q3F 2Q3G 2Q3H 2Q3I 2Q3J 2Q3K 2Q3L 2Q3M 2Q3N 2Q3O 2Q3P 2Q3Q 2Q3R 2Q3S 2Q3T 2Q3U 2Q3V 2Q3W 2Q3X 2Q3Y 2Q3Z 2Q40 2Q41 2Q42 2Q43 2Q44 2Q45 2Q46 2Q47 2Q48 2Q49 2Q4A 2Q4B 2Q4C 2Q4D 2Q4E 2Q4F 2Q4G 2Q4H 2Q4I 2Q4J 2Q4K 2Q4L 2Q4M 2Q4N 2Q4O 2Q4P 2Q4Q 2Q4R 2Q4S 2Q4T 2Q4U 2Q4V 2Q4W 2Q4X 2Q4Y 2Q4Z 2Q50 2Q51 2Q52 2Q53 2Q54 2Q55 2Q57 2Q58 2Q59 2Q5A 2Q5B 2Q5C 2Q5D 2Q5E 2Q5F 2Q5G 2Q5H 2Q5I 2Q5J 2Q5K 2Q5L 2Q5O 2Q5P 2Q5Q 2Q5R 2Q5S 2Q5T 2Q5U 2Q5W 2Q5X 2Q5Y 2Q5Z 2Q60 2Q61 2Q62 2Q63 2Q64 2Q67 2Q68 2Q69 2Q6A 2Q6B 2Q6C 2Q6D 2Q6E 2Q6F 2Q6G 2Q6H 2Q6I 2Q6J 2Q6K 2Q6L 2Q6M 2Q6N 2Q6O 2Q6P 2Q6Q 2Q6R 2Q6S 2Q6T 2Q6U 2Q6V 2Q6W 2Q6Z 2Q70 2Q71 2Q72 2Q73 2Q74 2Q76 2Q78 2Q79 2Q7A 2Q7B 2Q7C 2Q7D 2Q7E 2Q7F 2Q7G 2Q7H 2Q7I 2Q7J 2Q7K 2Q7L 2Q7M 2Q7N 2Q7O 2Q7Q 2Q7R 2Q7S 2Q7T 2Q7U 2Q7V 2Q7W 2Q7X 2Q7Y 2Q80 2Q81 2Q82 2Q83 2Q85 2Q86 2Q87 2Q88 2Q89 2Q8A 2Q8B 2Q8C 2Q8D 2Q8E 2Q8F 2Q8G 2Q8H 2Q8I 2Q8J 2Q8K 2Q8L 2Q8M 2Q8N 2Q8O 2Q8P 2Q8Q 2Q8R 2Q8S 2Q8T 2Q8U 2Q8V 2Q8W 2Q8X 2Q8Y 2Q8Z 2Q91 2Q92 2Q93 2Q94 2Q95 2Q96 2Q97 2Q98 2Q99 2Q9A 2Q9B 2Q9C 2Q9F 2Q9G 2Q9H 2Q9I 2Q9J 2Q9K 2Q9L 2Q9M 2Q9N 2Q9O 2Q9P 2Q9Q 2Q9R 2Q9S 2Q9T 2Q9U 2Q9V 2Q9X 2Q9Y 2Q9Z 2QA0 2QA1 2QA2 2QA3 2QA5 2QA6 2QA7 2QA8 2QA9 2QAA 2QAB 2QAC 2QAD 2QAE 2QAF 2QAG 2QAI 2QAJ 2QAK 2QAP 2QAR 2QAS 2QAZ 2QB0 2QB1 2QB2 2QB3 2QB4 2QB5 2QB6 2QB7 2QB8 2QBL 2QBM 2QBN 2QBO 2QBP 2QBQ 2QBR 2QBS 2QBT 2QBU 2QBV 2QBW 2QBX 2QC1 2QC2 2QC3 2QC5 2QC6 2QC7 2QC8 2QC9 2QCA 2QCB 2QCC 2QCD 2QCE 2QCF 2QCG 2QCH 2QCI 2QCJ 2QCK 2QCL 2QCM 2QCN 2QCO 2QCP 2QCQ 2QCS 2QCT 2QCU 2QCV 2QCW 2QCX 2QCY 2QCZ 2QD0 2QD1 2QD2 2QD3 2QD4 2QD5 2QD6 2QD7 2QD8 2QD9 2QDB 2QDC 2QDD 2QDE 2QDF 2QDG 2QDH 2QDI 2QDJ 2QDK 2QDL 2QDM 2QDN 2QDO 2QDP 2QDQ 2QDR 2QDS 2QDT 2QDV 2QDW 2QDX 2QDY 2QE0 2QE2 2QE3 2QE4 2QE5 2QE6 2QE7 2QE8 2QE9 2QEA 2QEB 2QEC 2QED 2QEE 2QEH 2QEI 2QEJ 2QEL 2QEN 2QEO 2QEP 2QEQ 2QER 2QES 2QET 2QEU 2QEV 2QEW 2QEY 2QEZ 2QF0 2QF1 2QF2 2QF3 2QF4 2QF5 2QF6 2QF7 2QF8 2QF9 2QFA 2QFB 2QFC 2QFD 2QFE 2QFF 2QFI 2QFK 2QFL 2QFN 2QFO 2QFP 2QFQ 2QFR 2QFS 2QFT 2QFU 2QFV 2QFW 2QFX 2QFY 2QFZ 2QG0 2QG1 2QG2 2QG3 2QG4 2QG5 2QG6 2QG7 2QG8 2QG9 2QGA 2QGB 2QGC 2QGD 2QGE 2QGF 2QGG 2QGH 2QGI 2QGM 2QGN 2QGO 2QGQ 2QGR 2QGS 2QGT 2QGU 2QGV 2QGW 2QGX 2QGY 2QGZ 2QH0 2QH1 2QH5 2QH6 2QH7 2QH9 2QHA 2QHC 2QHD 2QHE 2QHF 2QHK 2QHL 2QHM 2QHN 2QHO 2QHP 2QHQ 2QHR 2QHS 2QHT 2QHU 2QHV 2QHW 2QHX 2QHY 2QHZ 2QI0 2QI1 2QI2 2QI3 2QI4 2QI5 2QI6 2QI7 2QI8 2QI9 2QIA 2QIB 2QIC 2QIE 2QIF 2QIH 2QII 2QIJ 2QIK 2QIL 2QIM 2QIN 2QIO 2QIP 2QIQ 2QIR 2QIS 2QIU 2QIV 2QIW 2QIY 2QIZ 2QJ0 2QJ1 2QJ2 2QJ3 2QJ4 2QJ5 2QJ6 2QJ7 2QJ8 2QJ9 2QJA 2QJB 2QJC 2QJD 2QJE 2QJF 2QJG 2QJH 2QJI 2QJJ 2QJK 2QJL 2QJM 2QJN 2QJO 2QJP 2QJR 2QJS 2QJT 2QJU 2QJV 2QJW 2QJX 2QJY 2QJZ 2QK0 2QK1 2QK2 2QK4 2QK5 2QK7 2QK8 2QKA 2QKC 2QKD 2QKE 2QKF 2QKH 2QKI 2QKL 2QKM 2QKN 2QKO 2QKP 2QKQ 2QKR 2QKS 2QKT 2QKU 2QKV 2QKW 2QKX 2QKY 2QL1 2QL3 2QL5 2QL6 2QL7 2QL8 2QL9 2QLA 2QLB 2QLC 2QLD 2QLE 2QLF 2QLG 2QLH 2QLI 2QLJ 2QLK 2QLL 2QLM 2QLN 2QLP 2QLQ 2QLR 2QLS 2QLT 2QLU 2QLV 2QLW 2QLX 2QLY 2QLZ 2QM0 2QM1 2QM2 2QM3 2QM4 2QM6 2QM7 2QM8 2QM9 2QMA 2QMB 2QMC 2QMD 2QME 2QMF 2QMG 2QMH 2QMI 2QMJ 2QMK 2QML 2QMM 2QMO 2QMP 2QMQ 2QMR 2QMS 2QMT 2QMU 2QMW 2QMX 2QMY 2QMZ 2QN0 2QN1 2QN2 2QN3 2QN4 2QN5 2QN6 2QN7 2QN8 2QN9 2QNA 2QNB 2QND 2QNE 2QNG 2QNI 2QNJ 2QNK 2QNL 2QNN 2QNO 2QNP 2QNQ 2QNR 2QNT 2QNU 2QNV 2QNW 2QNX 2QNY 2QNZ 2QO0 2QO1 2QO2 2QO3 2QO4 2QO5 2QO6 2QO7 2QO8 2QO9 2QOA 2QOB 2QOC 2QOD 2QOE 2QOF 2QOG 2QOH 2QOI 2QOK 2QOL 2QOM 2QON 2QOO 2QOP 2QOQ 2QOR 2QOS 2QP2 2QP3 2QP4 2QP6 2QP8 2QP9 2QPA 2QPD 2QPE 2QPF 2QPJ 2QPK 2QPL 2QPM 2QPN 2QPO 2QPP 2QPQ 2QPS 2QPT 2QPU 2QPV 2QPW 2QPX 2QPY 2QPZ 2QQ0 2QQ1 2QQ2 2QQ3 2QQ4 2QQ5 2QQ6 2QQ7 2QQ8 2QQ9 2QQA 2QQB 2QQC 2QQD 2QQE 2QQF 2QQG 2QQH 2QQI 2QQJ 2QQK 2QQL 2QQM 2QQN 2QQO 2QQQ 2QQR 2QQS 2QQT 2QQU 2QQV 2QQW 2QQY 2QQZ 2QR0 2QR1 2QR2 2QR3 2QR4 2QR5 2QR6 2QR7 2QR8 2QR9 2QRA 2QRB 2QRC 2QRD 2QRE 2QRF 2QRG 2QRH 2QRI 2QRJ 2QRK 2QRL 2QRM 2QRN 2QRO 2QRP 2QRQ 2QRR 2QRS 2QRT 2QRU 2QRV 2QRW 2QRX 2QRY 2QRZ 2QS1 2QS2 2QS3 2QS4 2QS7 2QS8 2QS9 2QSA 2QSB 2QSC 2QSD 2QSE 2QSF 2QSI 2QSJ 2QSK 2QSP 2QSQ 2QSR 2QSS 2QST 2QSU 2QSV 2QSW 2QSX 2QSY 2QSZ 2QT0 2QT1 2QT2 2QT3 2QT4 2QT5 2QT6 2QT7 2QT8 2QT9 2QTA 2QTB 2QTC 2QTD 2QTE 2QTF 2QTG 2QTH 2QTI 2QTK 2QTL 2QTM 2QTN 2QTO 2QTP 2QTQ 2QTR 2QTS 2QTT 2QTU 2QTV 2QTW 2QTX 2QTY 2QTZ 2QU0 2QU1 2QU2 2QU3 2QU5 2QU6 2QU7 2QU8 2QU9 2QUA 2QUB 2QUD 2QUE 2QUF 2QUG 2QUH 2QUI 2QUJ 2QUK 2QUL 2QUM 2QUN 2QUO 2QUP 2QUQ 2QUR 2QUT 2QUU 2QUV 2QUY 2QUZ 2QV0 2QV1 2QV2 2QV3 2QV4 2QV5 2QV6 2QV7 2QV8 2QVA 2QVB 2QVC 2QVD 2QVE 2QVF 2QVG 2QVH 2QVI 2QVJ 2QVK 2QVL 2QVM 2QVN 2QVO 2QVP 2QVR 2QVS 2QVT 2QVU 2QVV 2QVW 2QVX 2QVY 2QVZ 2QW0 2QW1 2QW4 2QW5 2QW6 2QW7 2QW8 2QW9 2QWA 2QWB 2QWC 2QWD 2QWE 2QWF 2QWG 2QWH 2QWI 2QWJ 2QWK 2QWL 2QWM 2QWN 2QWO 2QWP 2QWQ 2QWR 2QWS 2QWT 2QWU 2QWV 2QWW 2QWX 2QWZ 2QX0 2QX1 2QX2 2QX3 2QX4 2QX5 2QX6 2QX7 2QX8 2QX9 2QXF 2QXG 2QXH 2QXI 2QXJ 2QXL 2QXM 2QXS 2QXT 2QXU 2QXV 2QXW 2QXX 2QXY 2QXZ 2QY0 2QY1 2QY2 2QY6 2QY7 2QY9 2QYA 2QYB 2QYC 2QYF 2QYG 2QYH 2QYI 2QYJ 2QYK 2QYL 2QYM 2QYN 2QYO 2QYP 2QYQ 2QYS 2QYT 2QYU 2QYV 2QYW 2QYZ 2QZ0 2QZ2 2QZ3 2QZ4 2QZ5 2QZ6 2QZ7 2QZ8 2QZ9 2QZA 2QZB 2QZC 2QZE 2QZG 2QZI 2QZJ 2QZK 2QZL 2QZO 2QZP 2QZQ 2QZR 2QZS 2QZT 2QZU 2QZV 2QZW 2QZX 2QZY 2QZZ 2R00 2R01 2R02 2R03 2R04 2R05 2R06 2R07 2R09 2R0B 2R0C 2R0D 2R0F 2R0G 2R0H 2R0I 2R0J 2R0K 2R0L 2R0M 2R0N 2R0O 2R0P 2R0R 2R0S 2R0T 2R0U 2R0V 2R0W 2R0X 2R0Y 2R0Z 2R10 2R11 2R13 2R14 2R15 2R16 2R17 2R18 2R19 2R1A 2R1B 2R1D 2R1F 2R1H 2R1I 2R1K 2R1L 2R1M 2R1N 2R1P 2R1Q 2R1R 2R1T 2R1U 2R1V 2R1W 2R1X 2R1Y 2R1Z 2R23 2R25 2R26 2R27 2R28 2R29 2R2A 2R2B 2R2C 2R2D 2R2E 2R2F 2R2G 2R2H 2R2I 2R2J 2R2K 2R2L 2R2M 2R2N 2R2O 2R2P 2R2Q 2R2V 2R2W 2R2X 2R2Y 2R2Z 2R30 2R31 2R32 2R33 2R34 2R35 2R36 2R37 2R38 2R39 2R3A 2R3B 2R3C 2R3D 2R3E 2R3F 2R3G 2R3H 2R3I 2R3J 2R3K 2R3L 2R3M 2R3N 2R3O 2R3P 2R3Q 2R3R 2R3S 2R3T 2R3U 2R3V 2R3W 2R3X 2R3Y 2R3Z 2R40 2R41 2R42 2R43 2R44 2R45 2R46 2R47 2R48 2R49 2R4B 2R4E 2R4F 2R4G 2R4H 2R4I 2R4J 2R4L 2R4N 2R4O 2R4P 2R4Q 2R4R 2R4S 2R4T 2R4U 2R4V 2R4W 2R4X 2R4Y 2R4Z 2R50 2R51 2R52 2R53 2R55 2R56 2R57 2R58 2R59 2R5A 2R5B 2R5C 2R5D 2R5E 2R5F 2R5G 2R5H 2R5I 2R5J 2R5K 2R5L 2R5M 2R5N 2R5O 2R5P 2R5Q 2R5R 2R5S 2R5T 2R5U 2R5V 2R5W 2R5X 2R60 2R61 2R62 2R64 2R65 2R66 2R68 2R69 2R6A 2R6C 2R6D 2R6E 2R6F 2R6G 2R6H 2R6I 2R6J 2R6K 2R6M 2R6N 2R6O 2R6Q 2R6R 2R6S 2R6T 2R6U 2R6V 2R6W 2R6X 2R6Y 2R6Z 2R70 2R71 2R72 2R73 2R74 2R75 2R76 2R77 2R78 2R79 2R7A 2R7B 2R7C 2R7D 2R7E 2R7F 2R7G 2R7H 2R7I 2R7J 2R7K 2R7L 2R7M 2R7N 2R7O 2R7P 2R7Q 2R80 2R82 2R83 2R84 2R85 2R86 2R87 2R88 2R89 2R8A 2R8B 2R8D 2R8E 2R8F 2R8N 2R8O 2R8P 2R8Q 2R8R 2R8T 2R8U 2R8V 2R8W 2R8X 2R8Y 2R8Z 2R90 2R91 2R94 2R96 2R97 2R98 2R99 2R9A 2R9B 2R9C 2R9E 2R9F 2R9G 2R9H 2R9I 2R9J 2R9K 2R9M 2R9N 2R9O 2R9P 2R9Q 2R9R 2R9S 2R9U 2R9V 2R9W 2R9X 2R9Y 2R9Z 2RA0 2RA1 2RA2 2RA3 2RA4 2RA5 2RA6 2RA8 2RA9 2RAA 2RAB 2RAC 2RAD 2RAE 2RAF 2RAG 2RAH 2RAI 2RAJ 2RAK 2RAL 2RAN 2RAO 2RAP 2RAQ 2RAR 2RAS 2RAT 2RAU 2RAV 2RAW 2RAX 2RAY 2RAZ 2RB0 2RB1 2RB2 2RB3 2RB4 2RB5 2RB6 2RB7 2RB8 2RB9 2RBB 2RBC 2RBD 2RBE 2RBG 2RBH 2RBI 2RBK 2RBL 2RBM 2RBN 2RBO 2RBP 2RBQ 2RBR 2RBS 2RBT 2RBU 2RBV 2RBW 2RBX 2RBY 2RBZ 2RC0 2RC1 2RC2 2RC3 2RC4 2RC5 2RC6 2RC7 2RC8 2RC9 2RCA 2RCB 2RCC 2RCD 2RCE 2RCF 2RCH 2RCI 2RCK 2RCL 2RCM 2RCN 2RCQ 2RCR 2RCS 2RCT 2RCU 2RCV 2RCW 2RCX 2RCY 2RCZ 2RD0 2RD1 2RD3 2RD4 2RD5 2RD6 2RD7 2RD8 2RD9 2RDA 2RDB 2RDC 2RDD 2RDE 2RDF 2RDG 2RDH 2RDI 2RDK 2RDL 2RDM 2RDN 2RDP 2RDQ 2RDR 2RDS 2RDT 2RDU 2RDV 2RDW 2RDX 2RDY 2RDZ 2RE1 2RE2 2RE3 2RE7 2RE9 2REA 2REB 2RED 2REE 2REF 2REG 2REH 2REI 2REJ 2REK 2REM 2REN 2REO 2REP 2REQ 2RER 2RES 2RET 2REU 2REW 2REX 2REY 2REZ 2RF0 2RF1 2RF2 2RF4 2RF5 2RF6 2RF7 2RF8 2RF9 2RFA 2RFB 2RFC 2RFD 2RFE 2RFF 2RFG 2RFH 2RFI 2RFJ 2RFL 2RFM 2RFN 2RFO 2RFQ 2RFR 2RFS 2RFT 2RFU 2RFV 2RFW 2RFX 2RFY 2RFZ 2RG0 2RG1 2RG2 2RG3 2RG4 2RG5 2RG6 2RG7 2RG8 2RG9 2RGA 2RGB 2RGC 2RGD 2RGE 2RGG 2RGH 2RGI 2RGJ 2RGK 2RGL 2RGM 2RGN 2RGO 2RGP 2RGQ 2RGS 2RGT 2RGU 2RGV 2RGW 2RGX 2RGY 2RGZ 2RH0 2RH1 2RH2 2RH3 2RH4 2RH5 2RH6 2RH7 2RH8 2RH9 2RHA 2RHB 2RHC 2RHD 2RHE 2RHF 2RHG 2RHH 2RHI 2RHJ 2RHK 2RHL 2RHM 2RHO 2RHP 2RHQ 2RHR 2RHS 2RHT 2RHU 2RHW 2RHX 2RHY 2RHZ 2RI0 2RI1 2RI2 2RI3 2RI4 2RI5 2RI6 2RI7 2RI8 2RI9 2RIA 2RIB 2RIC 2RID 2RIE 2RIF 2RIG 2RIH 2RII 2RIJ 2RIK 2RIL 2RIM 2RIN 2RIO 2RIP 2RIQ 2RIR 2RIS 2RIT 2RIU 2RIV 2RIW 2RIX 2RIY 2RIZ 2RJ0 2RJ1 2RJ2 2RJ3 2RJ4 2RJ5 2RJ6 2RJ7 2RJ8 2RJ9 2RJB 2RJC 2RJD 2RJE 2RJF 2RJG 2RJH 2RJI 2RJK 2RJL 2RJM 2RJN 2RJO 2RJP 2RJQ 2RJR 2RJS 2RJT 2RJV 2RJW 2RJX 2RJY 2RJZ 2RK0 2RK1 2RK2 2RK3 2RK4 2RK5 2RK6 2RK7 2RK8 2RK9 2RKA 2RKB 2RKC 2RKD 2RKE 2RKF 2RKG 2RKH 2RKI 2RKK 2RKL 2RKM 2RKN 2RKO 2RKQ 2RKS 2RKT 2RKU 2RKV 2RKW 2RKX 2RKY 2RKZ 2RL0 2RL1 2RL2 2RL3 2RL5 2RL7 2RL8 2RL9 2RLA 2RLB 2RLC 2RLD 2RLN 2RM2 2RMA 2RMB 2RMC 2RMP 2RMU 2RN2 2RNF 2RNS 2RNT 2ROM 2ROX 2ROY 2RR1 2RS1 2RS3 2RS5 2RSL 2RSP 2RTA 2RTB 2RTC 2RTD 2RTE 2RTF 2RTG 2RTH 2RTI 2RTJ 2RTK 2RTL 2RTM 2RTN 2RTO 2RTP 2RTQ 2RTR 2RUS 2SAK 2SAM 2SAR 2SAS 2SBA 2SBL 2SBT 2SCP 2SCU 2SEB 2SEC 2SEM 2SFA 2SFP 2SGA 2SGD 2SGE 2SGF 2SGP 2SGQ 2SHK 2SHP 2SIC 2SIL 2SIM 2SIV 2SKC 2SKD 2SKE 2SLI 2SN3 2SNI 2SNM 2SNS 2SNV 2SNW 2SOD 2SPC 2SPG 2SPL 2SPM 2SPN 2SPO 2SPT 2SQC 2SRC 2ST1 2STA 2STB 2STD 2TAA 2TBS 2TBV 2TCI 2TCL 2TCT 2TDD 2TDM 2TDT 2TDX 2TEC 2TEP 2TGA 2TGD 2TGI 2TGP 2TGT 2THF 2THI 2TIO 2TIR 2TLD 2TLI 2TLX 2TMA 2TMD 2TMG 2TMK 2TMN 2TMY 2TN4 2TNF 2TOD 2TOH 2TPI 2TPL 2TPR 2TPS 2TPT 2TRC 2TRH 2TRM 2TRS 2TRT 2TRX 2TRY 2TS1 2TSA 2TSB 2TSC 2TSR 2TSS 2TSY 2TUN 2TYS 2UAG 2UBP 2UCZ 2UDP 2UGI 2UKD 2ULL 2UPJ 2USH 2USN 2UTG 2UU7 2UU8 2UUD 2UUE 2UUF 2UUG 2UUH 2UUI 2UUJ 2UUK 2UUL 2UUM 2UUN 2UUO 2UUP 2UUQ 2UUR 2UUS 2UUT 2UUU 2UUV 2UUW 2UUX 2UUY 2UUZ 2UV0 2UV1 2UV2 2UV3 2UV4 2UV5 2UV6 2UV7 2UV8 2UVD 2UVE 2UVF 2UVG 2UVH 2UVI 2UVJ 2UVK 2UVL 2UVM 2UVN 2UVO 2UVP 2UVQ 2UVX 2UVY 2UVZ 2UW0 2UW1 2UW2 2UW3 2UW4 2UW5 2UW6 2UW7 2UW8 2UW9 2UWA 2UWB 2UWC 2UWD 2UWE 2UWF 2UWH 2UWI 2UWJ 2UWL 2UWN 2UWO 2UWP 2UWR 2UWS 2UWT 2UWU 2UWV 2UWW 2UWX 2UX0 2UX1 2UX2 2UX3 2UX4 2UX5 2UX6 2UX7 2UX8 2UX9 2UXA 2UXE 2UXF 2UXG 2UXH 2UXI 2UXJ 2UXK 2UXL 2UXM 2UXN 2UXO 2UXP 2UXQ 2UXR 2UXS 2UXT 2UXU 2UXV 2UXW 2UXX 2UXY 2UXZ 2UY0 2UY1 2UY2 2UY3 2UY4 2UY5 2UY6 2UY7 2UY8 2UY9 2UYA 2UYB 2UYD 2UYE 2UYF 2UYG 2UYI 2UYJ 2UYK 2UYL 2UYM 2UYN 2UYO 2UYP 2UYQ 2UYR 2UYT 2UYU 2UYV 2UYW 2UYX 2UYY 2UYZ 2UZ0 2UZ1 2UZ2 2UZ3 2UZ6 2UZ8 2UZ9 2UZA 2UZB 2UZC 2UZD 2UZE 2UZF 2UZH 2UZI 2UZJ 2UZL 2UZN 2UZO 2UZP 2UZQ 2UZR 2UZS 2UZT 2UZU 2UZV 2UZW 2UZX 2UZY 2UZZ 2V00 2V01 2V02 2V03 2V04 2V05 2V06 2V07 2V08 2V09 2V0A 2V0B 2V0C 2V0D 2V0H 2V0I 2V0J 2V0K 2V0L 2V0M 2V0N 2V0O 2V0P 2V0R 2V0S 2V0T 2V0U 2V0V 2V0W 2V0X 2V0Y 2V0Z 2V10 2V11 2V12 2V13 2V14 2V15 2V16 2V17 2V18 2V19 2V1A 2V1B 2V1C 2V1D 2V1E 2V1F 2V1G 2V1H 2V1I 2V1J 2V1K 2V1L 2V1M 2V1O 2V1P 2V1Q 2V1R 2V1S 2V1T 2V1W 2V1X 2V1Y 2V1Z 2V20 2V21 2V22 2V23 2V24 2V25 2V26 2V27 2V28 2V29 2V2A 2V2B 2V2C 2V2D 2V2E 2V2F 2V2G 2V2H 2V2I 2V2J 2V2K 2V2L 2V2M 2V2N 2V2O 2V2P 2V2Q 2V2R 2V2S 2V2U 2V2V 2V2W 2V2X 2V2Z 2V30 2V32 2V33 2V34 2V35 2V36 2V38 2V3A 2V3B 2V3D 2V3E 2V3F 2V3G 2V3H 2V3I 2V3J 2V3K 2V3M 2V3N 2V3O 2V3P 2V3Q 2V3R 2V3S 2V3T 2V3U 2V3V 2V3W 2V3X 2V3Y 2V3Z 2V40 2V41 2V42 2V43 2V45 2V4A 2V4B 2V4C 2V4D 2V4E 2V4H 2V4I 2V4J 2V4L 2V4M 2V4N 2V4O 2V4U 2V4V 2V4X 2V4Y 2V4Z 2V50 2V51 2V52 2V53 2V54 2V55 2V57 2V58 2V59 2V5A 2V5B 2V5C 2V5D 2V5E 2V5F 2V5G 2V5H 2V5I 2V5J 2V5K 2V5L 2V5M 2V5N 2V5O 2V5P 2V5Q 2V5R 2V5S 2V5T 2V5U 2V5V 2V5W 2V5X 2V5Y 2V5Z 2V60 2V61 2V62 2V63 2V64 2V65 2V66 2V67 2V68 2V69 2V6A 2V6B 2V6C 2V6F 2V6G 2V6H 2V6I 2V6J 2V6K 2V6M 2V6N 2V6O 2V6Q 2V6S 2V6T 2V6U 2V6V 2V6X 2V6Y 2V70 2V71 2V72 2V73 2V74 2V75 2V76 2V77 2V78 2V79 2V7A 2V7B 2V7C 2V7D 2V7E 2V7F 2V7G 2V7H 2V7I 2V7J 2V7K 2V7L 2V7M 2V7N 2V7O 2V7P 2V7Q 2V7S 2V7T 2V7U 2V7V 2V7W 2V7X 2V7Y 2V7Z 2V81 2V82 2V83 2V84 2V85 2V86 2V87 2V88 2V89 2V8A 2V8B 2V8C 2V8D 2V8E 2V8F 2V8G 2V8H 2V8I 2V8J 2V8K 2V8L 2V8M 2V8N 2V8O 2V8P 2V8Q 2V8S 2V8T 2V8U 2V8V 2V8W 2V8X 2V8Y 2V8Z 2V90 2V91 2V92 2V94 2V95 2V96 2V97 2V98 2V9A 2V9B 2V9C 2V9D 2V9E 2V9F 2V9G 2V9I 2V9J 2V9K 2V9L 2V9M 2V9N 2V9O 2V9P 2V9Q 2V9R 2V9S 2V9T 2V9U 2V9V 2V9X 2V9Y 2V9Z 2VA0 2VA1 2VA5 2VA6 2VA7 2VA8 2VA9 2VAA 2VAB 2VAC 2VAD 2VAE 2VAF 2VAG 2VAJ 2VAK 2VAM 2VAN 2VAO 2VAP 2VAQ 2VAR 2VAS 2VAT 2VAU 2VAV 2VAW 2VAX 2VAY 2VB0 2VB1 2VB2 2VB3 2VB6 2VB7 2VB8 2VB9 2VBA 2VBB 2VBC 2VBD 2VBE 2VBF 2VBG 2VBI 2VBK 2VBM 2VBP 2VBQ 2VBS 2VBT 2VBU 2VBV 2VBW 2VBX 2VBY 2VBZ 2VC0 2VC1 2VC2 2VC3 2VC4 2VC5 2VC6 2VC7 2VC8 2VC9 2VCA 2VCB 2VCC 2VCE 2VCF 2VCG 2VCH 2VCI 2VCJ 2VCK 2VCL 2VCM 2VCN 2VCO 2VCP 2VCQ 2VCS 2VCT 2VCV 2VCW 2VCX 2VCY 2VCZ 2VD0 2VD1 2VD2 2VD3 2VD4 2VD5 2VD6 2VD7 2VD8 2VD9 2VDB 2VDD 2VDE 2VDF 2VDG 2VDH 2VDI 2VDJ 2VDK 2VDL 2VDM 2VDN 2VDO 2VDP 2VDQ 2VDR 2VDT 2VDU 2VDV 2VDW 2VDX 2VDY 2VE1 2VE3 2VE4 2VE6 2VE7 2VE8 2VEA 2VEB 2VEC 2VED 2VEE 2VEF 2VEG 2VEI 2VEK 2VEL 2VEM 2VEN 2VEO 2VEP 2VEQ 2VES 2VET 2VEU 2VEV 2VEW 2VEX 2VEY 2VEZ 2VF0 2VF1 2VF2 2VF3 2VF4 2VF5 2VF6 2VF7 2VF8 2VF9 2VFA 2VFB 2VFC 2VFD 2VFE 2VFF 2VFG 2VFH 2VFI 2VFJ 2VFK 2VFL 2VFM 2VFN 2VFO 2VFP 2VFQ 2VFR 2VFS 2VFT 2VFU 2VFV 2VFW 2VFX 2VFY 2VFZ 2VG0 2VG1 2VG2 2VG3 2VG4 2VG5 2VG6 2VG7 2VG8 2VG9 2VGA 2VGB 2VGC 2VGD 2VGE 2VGF 2VGG 2VGI 2VGJ 2VGK 2VGL 2VGM 2VGN 2VGO 2VGP 2VGQ 2VGR 2VGS 2VGT 2VGU 2VGV 2VGW 2VGX 2VGY 2VGZ 2VH0 2VH1 2VH2 2VH3 2VH4 2VH5 2VH6 2VH7 2VH9 2VHA 2VHB 2VHC 2VHD 2VHE 2VHF 2VHH 2VHI 2VHJ 2VHK 2VHL 2VHQ 2VHR 2VHS 2VHT 2VHU 2VHV 2VHW 2VHX 2VHY 2VHZ 2VI0 2VI1 2VI2 2VI3 2VI4 2VI5 2VI6 2VI7 2VI8 2VI9 2VIA 2VIB 2VID 2VIE 2VIF 2VIG 2VII 2VIJ 2VIM 2VIN 2VIO 2VIP 2VIQ 2VIR 2VIS 2VIT 2VIU 2VIV 2VIW 2VIX 2VIY 2VIZ 2VJ0 2VJ1 2VJ2 2VJ3 2VJ4 2VJ5 2VJ6 2VJ7 2VJ8 2VJ9 2VJA 2VJB 2VJC 2VJD 2VJE 2VJF 2VJH 2VJI 2VJJ 2VJK 2VJL 2VJM 2VJN 2VJO 2VJP 2VJQ 2VJR 2VJT 2VJW 2VJX 2VJY 2VJZ 2VK0 2VK1 2VK2 2VK3 2VK4 2VK5 2VK6 2VK7 2VK8 2VK9 2VKA 2VKD 2VKE 2VKF 2VKG 2VKH 2VKI 2VKJ 2VKL 2VKM 2VKN 2VKO 2VKP 2VKQ 2VKR 2VKS 2VKT 2VKU 2VKV 2VKW 2VKX 2VKY 2VKZ 2VL0 2VL1 2VL2 2VL3 2VL4 2VL5 2VL6 2VL7 2VL8 2VL9 2VLB 2VLC 2VLD 2VLE 2VLF 2VLG 2VLH 2VLI 2VLJ 2VLK 2VLL 2VLM 2VLN 2VLO 2VLP 2VLQ 2VLR 2VLT 2VLU 2VLV 2VLW 2VLX 2VLY 2VLZ 2VM0 2VM1 2VM2 2VM3 2VM4 2VM5 2VM6 2VM8 2VM9 2VMA 2VMB 2VMC 2VMD 2VME 2VMF 2VMG 2VMH 2VMI 2VMJ 2VMK 2VML 2VMN 2VMO 2VMP 2VMQ 2VMR 2VMS 2VMT 2VMU 2VMV 2VMW 2VMX 2VMY 2VMZ 2VN0 2VN1 2VN2 2VN3 2VN4 2VN5 2VN6 2VN7 2VN8 2VN9 2VNA 2VNC 2VND 2VNE 2VNF 2VNG 2VNH 2VNI 2VNJ 2VNK 2VNL 2VNM 2VNN 2VNO 2VNP 2VNQ 2VNR 2VNS 2VNT 2VNV 2VNW 2VNX 2VNY 2VNZ 2VO0 2VO1 2VO2 2VO3 2VO4 2VO5 2VO6 2VO7 2VO8 2VO9 2VOB 2VOC 2VOE 2VOF 2VOG 2VOH 2VOI 2VOJ 2VOK 2VOM 2VOR 2VOS 2VOT 2VOU 2VOV 2VOW 2VOX 2VOZ 2VP0 2VP1 2VP2 2VP3 2VP4 2VP5 2VP6 2VP7 2VP8 2VP9 2VPA 2VPB 2VPD 2VPE 2VPF 2VPG 2VPH 2VPI 2VPJ 2VPK 2VPM 2VPN 2VPO 2VPP 2VPQ 2VPR 2VPS 2VPT 2VPV 2VPW 2VPX 2VPY 2VPZ 2VQ0 2VQ1 2VQ2 2VQ3 2VQ4 2VQ5 2VQ6 2VQ7 2VQ8 2VQ9 2VQA 2VQB 2VQC 2VQD 2VQG 2VQH 2VQI 2VQJ 2VQK 2VQL 2VQM 2VQO 2VQP 2VQQ 2VQR 2VQS 2VQT 2VQU 2VQV 2VQW 2VQX 2VQY 2VQZ 2VR0 2VR1 2VR2 2VR3 2VR4 2VR5 2VR6 2VR7 2VR8 2VR9 2VRA 2VRB 2VRC 2VRE 2VRF 2VRI 2VRJ 2VRK 2VRL 2VRM 2VRN 2VRO 2VRP 2VRQ 2VRR 2VRS 2VRW 2VRX 2VRY 2VRZ 2VS0 2VS1 2VS3 2VS4 2VS5 2VS6 2VSA 2VSC 2VSD 2VSE 2VSF 2VSG 2VSH 2VSI 2VSK 2VSL 2VSM 2VSN 2VSO 2VSP 2VSQ 2VSR 2VSS 2VST 2VSU 2VSV 2VSW 2VSX 2VSY 2VSZ 2VT0 2VT1 2VT2 2VT3 2VT4 2VT5 2VT6 2VT7 2VT8 2VTA 2VTC 2VTD 2VTE 2VTF 2VTG 2VTH 2VTI 2VTJ 2VTK 2VTL 2VTM 2VTN 2VTO 2VTP 2VTQ 2VTR 2VTS 2VTT 2VTU 2VTV 2VTW 2VTX 2VTY 2VTZ 2VU0 2VU1 2VU2 2VU3 2VU4 2VU5 2VU6 2VU7 2VU8 2VU9 2VUA 2VUB 2VUC 2VUD 2VUE 2VUF 2VUG 2VUH 2VUI 2VUJ 2VUK 2VUL 2VUN 2VUO 2VUP 2VUR 2VUS 2VUT 2VUU 2VUV 2VUW 2VUX 2VUY 2VUZ 2VV0 2VV1 2VV2 2VV3 2VV4 2VV5 2VV6 2VV7 2VV8 2VV9 2VVA 2VVB 2VVC 2VVD 2VVE 2VVF 2VVG 2VVH 2VVI 2VVJ 2VVK 2VVL 2VVM 2VVN 2VVO 2VVP 2VVQ 2VVR 2VVS 2VVT 2VVU 2VVV 2VVW 2VVX 2VVY 2VVZ 2VW0 2VW1 2VW2 2VW4 2VW5 2VW6 2VW7 2VW8 2VWA 2VWB 2VWC 2VWD 2VWE 2VWF 2VWG 2VWH 2VWI 2VWK 2VWL 2VWM 2VWN 2VWO 2VWP 2VWQ 2VWR 2VWS 2VWT 2VWU 2VWV 2VWW 2VWX 2VWY 2VWZ 2VX0 2VX1 2VX2 2VX3 2VX4 2VX5 2VX6 2VX7 2VX8 2VX9 2VXA 2VXB 2VXC 2VXG 2VXH 2VXI 2VXJ 2VXK 2VXL 2VXM 2VXN 2VXO 2VXP 2VXQ 2VXR 2VXS 2VXT 2VXU 2VXV 2VXW 2VXX 2VXY 2VXZ 2VY0 2VY3 2VY6 2VY7 2VY8 2VY9 2VYA 2VYC 2VYF 2VYI 2VYN 2VYO 2VYP 2VYQ 2VYR 2VYT 2VYU 2VYV 2VYW 2VYX 2VYY 2VYZ 2VZ0 2VZ1 2VZ2 2VZ3 2VZ5 2VZ6 2VZ7 2VZ8 2VZ9 2VZA 2VZB 2VZC 2VZD 2VZE 2VZG 2VZI 2VZK 2VZL 2VZM 2VZN 2VZO 2VZP 2VZQ 2VZR 2VZS 2VZT 2VZU 2VZV 2VZW 2VZX 2VZY 2VZZ 2W00 2W01 2W02 2W03 2W04 2W05 2W06 2W07 2W08 2W09 2W0A 2W0B 2W0C 2W0D 2W0F 2W0G 2W0H 2W0I 2W0J 2W0K 2W0L 2W0M 2W0O 2W0P 2W0Q 2W0R 2W0S 2W0U 2W0V 2W0W 2W0X 2W0Z 2W10 2W11 2W12 2W13 2W14 2W15 2W16 2W17 2W18 2W19 2W1A 2W1B 2W1C 2W1D 2W1E 2W1F 2W1G 2W1H 2W1I 2W1J 2W1K 2W1L 2W1M 2W1N 2W1P 2W1Q 2W1R 2W1S 2W1T 2W1U 2W1V 2W1W 2W1X 2W1Y 2W1Z 2W20 2W21 2W22 2W23 2W24 2W25 2W26 2W27 2W29 2W2A 2W2B 2W2C 2W2D 2W2E 2W2F 2W2G 2W2I 2W2J 2W2K 2W2L 2W2M 2W2N 2W2O 2W2P 2W2Q 2W2R 2W2S 2W2T 2W2U 2W2V 2W2W 2W2X 2W31 2W37 2W38 2W39 2W3A 2W3B 2W3C 2W3D 2W3E 2W3F 2W3G 2W3H 2W3I 2W3J 2W3K 2W3L 2W3M 2W3N 2W3O 2W3P 2W3Q 2W3R 2W3S 2W3T 2W3U 2W3V 2W3W 2W3X 2W3Y 2W3Z 2W40 2W41 2W43 2W44 2W45 2W46 2W47 2W48 2W4B 2W4C 2W4D 2W4E 2W4F 2W4I 2W4J 2W4K 2W4L 2W4M 2W4O 2W4P 2W4Q 2W4R 2W4S 2W4X 2W4Y 2W4Z 2W50 2W51 2W52 2W53 2W54 2W55 2W56 2W57 2W58 2W59 2W5A 2W5B 2W5E 2W5F 2W5G 2W5H 2W5I 2W5J 2W5K 2W5L 2W5M 2W5N 2W5O 2W5P 2W5Q 2W5R 2W5S 2W5T 2W5U 2W5V 2W5W 2W5X 2W5Y 2W5Z 2W60 2W61 2W62 2W63 2W65 2W66 2W67 2W68 2W69 2W6A 2W6B 2W6C 2W6E 2W6F 2W6G 2W6H 2W6I 2W6J 2W6K 2W6L 2W6M 2W6N 2W6O 2W6P 2W6Q 2W6R 2W6T 2W6U 2W6V 2W6W 2W6X 2W6Y 2W6Z 2W70 2W71 2W72 2W73 2W75 2W76 2W77 2W78 2W79 2W7A 2W7D 2W7E 2W7F 2W7G 2W7H 2W7I 2W7J 2W7K 2W7L 2W7M 2W7Q 2W7R 2W7S 2W7T 2W7U 2W7V 2W7W 2W7X 2W7Y 2W7Z 2W80 2W81 2W82 2W83 2W86 2W87 2W88 2W8B 2W8C 2W8D 2W8F 2W8G 2W8H 2W8I 2W8J 2W8M 2W8N 2W8O 2W8P 2W8Q 2W8R 2W8S 2W8T 2W8U 2W8V 2W8W 2W8X 2W8Y 2W8Z 2W90 2W91 2W92 2W93 2W94 2W95 2W96 2W97 2W98 2W99 2W9D 2W9E 2W9F 2W9G 2W9H 2W9I 2W9J 2W9L 2W9M 2W9N 2W9P 2W9Q 2W9R 2W9S 2W9T 2W9X 2W9Y 2W9Z 2WA0 2WA1 2WA2 2WA3 2WA4 2WA5 2WA6 2WA7 2WA8 2WA9 2WAA 2WAB 2WAC 2WAD 2WAE 2WAF 2WAG 2WAH 2WAJ 2WAL 2WAM 2WAN 2WAO 2WAP 2WAQ 2WAR 2WAS 2WAT 2WAU 2WAW 2WAX 2WAY 2WAZ 2WB0 2WB1 2WB3 2WB4 2WB5 2WB6 2WB7 2WB8 2WB9 2WBA 2WBB 2WBC 2WBD 2WBF 2WBG 2WBH 2WBI 2WBJ 2WBK 2WBL 2WBM 2WBN 2WBO 2WBP 2WBQ 2WBT 2WBV 2WBW 2WBX 2WBY 2WBZ 2WC0 2WC1 2WC3 2WC4 2WC5 2WC6 2WC7 2WC8 2WC9 2WCA 2WCB 2WCD 2WCE 2WCF 2WCG 2WCH 2WCI 2WCJ 2WCK 2WCL 2WCM 2WCO 2WCP 2WCQ 2WCR 2WCS 2WCT 2WCU 2WCV 2WCW 2WCX 2WCZ 2WD0 2WD1 2WD2 2WD3 2WD4 2WD5 2WD6 2WD7 2WD8 2WD9 2WDA 2WDB 2WDC 2WDD 2WDE 2WDF 2WDO 2WDP 2WDQ 2WDR 2WDS 2WDT 2WDU 2WDV 2WDW 2WDY 2WDZ 2WE0 2WE1 2WE2 2WE3 2WE4 2WE5 2WE6 2WE7 2WE8 2WE9 2WEA 2WEB 2WEC 2WED 2WEE 2WEF 2WEG 2WEH 2WEI 2WEJ 2WEK 2WEL 2WEO 2WEP 2WEQ 2WER 2WES 2WET 2WEU 2WEV 2WEW 2WEX 2WEY 2WEZ 2WF0 2WF1 2WF2 2WF3 2WF4 2WF5 2WF6 2WF7 2WF8 2WF9 2WFA 2WFB 2WFC 2WFD 2WFE 2WFF 2WFG 2WFH 2WFI 2WFJ 2WFK 2WFL 2WFM 2WFN 2WFO 2WFP 2WFQ 2WFR 2WFT 2WFU 2WFV 2WFW 2WFX 2WFY 2WFZ 2WG0 2WG1 2WG2 2WG3 2WG4 2WG5 2WG6 2WG7 2WG8 2WG9 2WGB 2WGC 2WGD 2WGE 2WGF 2WGG 2WGH 2WGI 2WGJ 2WGL 2WGM 2WGP 2WGQ 2WGR 2WGS 2WGT 2WGU 2WGV 2WGW 2WGX 2WGY 2WGZ 2WH0 2WH5 2WH6 2WH7 2WH8 2WHB 2WHD 2WHE 2WHF 2WHG 2WHH 2WHI 2WHJ 2WHK 2WHL 2WHM 2WHN 2WHO 2WHP 2WHQ 2WHR 2WHS 2WHT 2WHU 2WHV 2WHW 2WHX 2WHY 2WHZ 2WI0 2WI1 2WI2 2WI3 2WI4 2WI5 2WI6 2WI7 2WI8 2WI9 2WIA 2WIB 2WIC 2WID 2WIE 2WIF 2WIG 2WIH 2WII 2WIJ 2WIK 2WIL 2WIM 2WIN 2WIO 2WIP 2WIQ 2WIS 2WIT 2WIU 2WIV 2WIY 2WJ1 2WJ2 2WJ3 2WJ4 2WJ5 2WJ6 2WJ7 2WJ9 2WJA 2WJD 2WJE 2WJF 2WJG 2WJH 2WJI 2WJJ 2WJK 2WJL 2WJM 2WJN 2WJO 2WJP 2WJQ 2WJR 2WJS 2WJU 2WJV 2WJW 2WJX 2WJY 2WJZ 2WK0 2WK1 2WK2 2WK3 2WK4 2WK5 2WK6 2WK7 2WK8 2WK9 2WKA 2WKB 2WKC 2WKD 2WKE 2WKF 2WKG 2WKH 2WKI 2WKJ 2WKK 2WKL 2WKM 2WKN 2WKO 2WKP 2WKQ 2WKR 2WKS 2WKT 2WKU 2WKV 2WKW 2WKX 2WKY 2WKZ 2WL0 2WL1 2WL3 2WL4 2WL5 2WL6 2WL7 2WL8 2WL9 2WLA 2WLB 2WLC 2WLD 2WLE 2WLF 2WLG 2WLH 2WLI 2WLJ 2WLK 2WLL 2WLM 2WLN 2WLO 2WLP 2WLQ 2WLR 2WLS 2WLT 2WLU 2WLV 2WLW 2WLX 2WLY 2WLZ 2WM0 2WM1 2WM2 2WM3 2WM4 2WM5 2WM8 2WM9 2WMA 2WMB 2WMC 2WMD 2WME 2WMF 2WMG 2WMH 2WMI 2WMJ 2WMK 2WML 2WMM 2WMN 2WMO 2WMP 2WMQ 2WMR 2WMS 2WMT 2WMU 2WMV 2WMW 2WMX 2WMY 2WMZ 2WN2 2WN3 2WN4 2WN5 2WN6 2WN7 2WN8 2WN9 2WNB 2WNC 2WND 2WNE 2WNF 2WNG 2WNH 2WNI 2WNJ 2WNK 2WNL 2WNN 2WNO 2WNP 2WNQ 2WNR 2WNS 2WNT 2WNU 2WNV 2WNW 2WNX 2WNY 2WNZ 2WO0 2WO1 2WO2 2WO3 2WO4 2WO5 2WO6 2WO7 2WO8 2WO9 2WOA 2WOB 2WOC 2WOD 2WOE 2WOF 2WOG 2WOH 2WOI 2WOJ 2WOK 2WOL 2WOM 2WON 2WOO 2WOP 2WOQ 2WOR 2WOS 2WOT 2WOU 2WOV 2WOW 2WOX 2WOY 2WOZ 2WP0 2WP1 2WP2 2WP3 2WP4 2WP5 2WP6 2WP7 2WP8 2WP9 2WPA 2WPB 2WPC 2WPD 2WPE 2WPF 2WPG 2WPH 2WPI 2WPJ 2WPK 2WPL 2WPM 2WPN 2WPO 2WPQ 2WPR 2WPS 2WPT 2WPU 2WPV 2WPW 2WPX 2WPY 2WPZ 2WQ0 2WQ1 2WQ2 2WQ3 2WQ4 2WQ5 2WQ8 2WQ9 2WQA 2WQB 2WQD 2WQE 2WQF 2WQH 2WQI 2WQJ 2WQK 2WQL 2WQM 2WQN 2WQO 2WQP 2WQQ 2WQR 2WQS 2WQT 2WQU 2WQV 2WQW 2WQX 2WQY 2WQZ 2WR0 2WR1 2WR2 2WR3 2WR4 2WR5 2WR6 2WR7 2WR8 2WR9 2WRA 2WRB 2WRC 2WRD 2WRE 2WRF 2WRG 2WRH 2WRM 2WRS 2WRT 2WRU 2WRV 2WRW 2WRX 2WRY 2WRZ 2WS0 2WS1 2WS2 2WS3 2WS4 2WS6 2WS7 2WS9 2WSA 2WSB 2WSC 2WSD 2WSE 2WSF 2WSH 2WSI 2WSJ 2WSK 2WSL 2WSM 2WSN 2WSO 2WSP 2WSQ 2WSR 2WSS 2WST 2WSU 2WSV 2WSW 2WSX 2WSY 2WT0 2WT1 2WT2 2WT4 2WT8 2WT9 2WTA 2WTB 2WTC 2WTD 2WTE 2WTG 2WTH 2WTI 2WTJ 2WTK 2WTL 2WTM 2WTN 2WTO 2WTP 2WTR 2WTS 2WTT 2WTV 2WTW 2WTX 2WTZ 2WU0 2WU1 2WU2 2WU3 2WU4 2WU5 2WU6 2WU7 2WU8 2WU9 2WUA 2WUB 2WUC 2WUD 2WUE 2WUF 2WUG 2WUH 2WUI 2WUJ 2WUK 2WUL 2WUQ 2WUR 2WUS 2WUT 2WUU 2WUV 2WUW 2WUX 2WUY 2WUZ 2WV0 2WV1 2WV2 2WV3 2WV4 2WV5 2WV6 2WV7 2WV8 2WV9 2WVA 2WVB 2WVC 2WVD 2WVE 2WVF 2WVG 2WVH 2WVI 2WVJ 2WVK 2WVL 2WVM 2WVN 2WVO 2WVP 2WVQ 2WVR 2WVS 2WVT 2WVU 2WVV 2WVX 2WVY 2WVZ 2WW0 2WW1 2WW2 2WW3 2WW4 2WW5 2WW6 2WW7 2WW8 2WWC 2WWD 2WWE 2WWF 2WWG 2WWH 2WWI 2WWJ 2WWK 2WWM 2WWN 2WWO 2WWP 2WWR 2WWS 2WWT 2WWU 2WWW 2WWX 2WWZ 2WX0 2WX1 2WX2 2WX3 2WX4 2WX5 2WX9 2WXB 2WXD 2WXF 2WXG 2WXH 2WXI 2WXJ 2WXK 2WXL 2WXM 2WXN 2WXO 2WXP 2WXQ 2WXR 2WXT 2WXU 2WXV 2WXW 2WXX 2WXY 2WXZ 2WY0 2WY1 2WY3 2WY4 2WY6 2WY7 2WY8 2WYA 2WYB 2WYC 2WYD 2WYE 2WYF 2WYG 2WYH 2WYI 2WYJ 2WYK 2WYL 2WYM 2WYN 2WYO 2WYP 2WYQ 2WYR 2WYS 2WYT 2WYU 2WYV 2WYW 2WYZ 2WZ0 2WZ1 2WZ5 2WZ6 2WZ7 2WZ8 2WZ9 2WZA 2WZB 2WZC 2WZD 2WZE 2WZF 2WZG 2WZH 2WZI 2WZJ 2WZK 2WZL 2WZM 2WZN 2WZO 2WZP 2WZQ 2WZR 2WZS 2WZT 2WZV 2WZW 2WZX 2WZY 2WZZ 2X00 2X01 2X02 2X03 2X04 2X05 2X06 2X07 2X08 2X09 2X0A 2X0B 2X0C 2X0D 2X0E 2X0F 2X0G 2X0H 2X0I 2X0J 2X0K 2X0L 2X0N 2X0O 2X0P 2X0Q 2X0R 2X0S 2X0U 2X0V 2X0W 2X0X 2X0Y 2X10 2X11 2X12 2X13 2X14 2X15 2X16 2X17 2X18 2X19 2X1B 2X1C 2X1D 2X1E 2X1G 2X1H 2X1I 2X1J 2X1K 2X1L 2X1M 2X1N 2X1O 2X1P 2X1Q 2X1R 2X1S 2X1T 2X1U 2X1V 2X1W 2X1X 2X1Z 2X20 2X21 2X22 2X23 2X24 2X25 2X26 2X27 2X28 2X29 2X2A 2X2B 2X2C 2X2D 2X2E 2X2F 2X2G 2X2H 2X2I 2X2J 2X2K 2X2L 2X2M 2X2N 2X2O 2X2P 2X2R 2X2S 2X2T 2X2U 2X2V 2X2W 2X2Y 2X2Z 2X30 2X32 2X34 2X35 2X36 2X38 2X39 2X3A 2X3B 2X3C 2X3D 2X3E 2X3F 2X3G 2X3H 2X3J 2X3K 2X3L 2X3M 2X3N 2X3O 2X3T 2X3U 2X3V 2X3W 2X3X 2X3Y 2X40 2X41 2X42 2X44 2X45 2X46 2X47 2X48 2X49 2X4A 2X4D 2X4F 2X4G 2X4H 2X4I 2X4J 2X4K 2X4L 2X4M 2X4N 2X4O 2X4P 2X4Q 2X4R 2X4S 2X4T 2X4U 2X4W 2X4X 2X4Y 2X4Z 2X50 2X51 2X52 2X53 2X55 2X56 2X57 2X58 2X5C 2X5D 2X5F 2X5G 2X5H 2X5I 2X5J 2X5K 2X5L 2X5N 2X5O 2X5P 2X5Q 2X5R 2X5S 2X5T 2X5U 2X5V 2X5W 2X5X 2X5Y 2X5Z 2X60 2X61 2X62 2X63 2X64 2X65 2X66 2X67 2X68 2X69 2X6A 2X6B 2X6C 2X6D 2X6E 2X6F 2X6G 2X6H 2X6I 2X6J 2X6K 2X6L 2X6M 2X6N 2X6O 2X6P 2X6Q 2X6R 2X6S 2X6T 2X6U 2X6W 2X6X 2X6Y 2X70 2X71 2X72 2X74 2X75 2X76 2X77 2X78 2X79 2X7A 2X7B 2X7C 2X7D 2X7E 2X7F 2X7G 2X7H 2X7I 2X7J 2X7K 2X7L 2X7M 2X7O 2X7P 2X7Q 2X7R 2X7S 2X7T 2X7U 2X7V 2X7W 2X7X 2X7Y 2X7Z 2X80 2X81 2X82 2X83 2X85 2X86 2X87 2X88 2X89 2X8A 2X8B 2X8C 2X8D 2X8E 2X8F 2X8G 2X8H 2X8I 2X8J 2X8K 2X8L 2X8M 2X8O 2X8P 2X8R 2X8S 2X8T 2X8U 2X8W 2X8X 2X8Y 2X8Z 2X90 2X91 2X92 2X93 2X94 2X95 2X96 2X97 2X98 2X99 2X9A 2X9B 2X9C 2X9D 2X9E 2X9F 2X9G 2X9H 2X9I 2X9J 2X9K 2X9L 2X9M 2X9N 2X9O 2X9P 2X9Q 2X9V 2X9W 2X9X 2X9Y 2X9Z 2XA0 2XA1 2XA2 2XA3 2XA4 2XA5 2XA7 2XA8 2XA9 2XAA 2XAB 2XAC 2XAD 2XAE 2XAF 2XAG 2XAH 2XAJ 2XAK 2XAL 2XAM 2XAN 2XAO 2XAP 2XAQ 2XAR 2XAS 2XAT 2XAU 2XAV 2XAW 2XAX 2XAY 2XAZ 2XB0 2XB1 2XB3 2XB4 2XB5 2XB6 2XB7 2XB8 2XB9 2XBA 2XBB 2XBF 2XBG 2XBI 2XBJ 2XBK 2XBL 2XBN 2XBO 2XBP 2XBQ 2XBR 2XBS 2XBT 2XBU 2XBV 2XBW 2XBX 2XBY 2XBZ 2XC0 2XC1 2XC2 2XC3 2XC4 2XC5 2XC8 2XCB 2XCC 2XCD 2XCE 2XCF 2XCG 2XCH 2XCI 2XCJ 2XCK 2XCL 2XCM 2XCN 2XCO 2XCQ 2XCU 2XCV 2XCW 2XCX 2XCY 2XCZ 2XD1 2XD2 2XD3 2XD4 2XD5 2XD6 2XD7 2XD9 2XDA 2XDC 2XDE 2XDG 2XDH 2XDJ 2XDK 2XDL 2XDM 2XDN 2XDO 2XDP 2XDQ 2XDR 2XDS 2XDU 2XDV 2XDW 2XDX 2XDY 2XE1 2XE2 2XE3 2XE4 2XE5 2XE6 2XE7 2XE8 2XEC 2XED 2XEE 2XEF 2XEG 2XEH 2XEI 2XEJ 2XEL 2XEM 2XEN 2XEP 2XEQ 2XER 2XES 2XET 2XEU 2XEV 2XEW 2XEX 2XEY 2XEZ 2XF0 2XF1 2XF2 2XF3 2XF4 2XF5 2XF6 2XF7 2XF8 2XFA 2XFD 2XFE 2XFF 2XFG 2XFH 2XFI 2XFJ 2XFK 2XFL 2XFN 2XFO 2XFP 2XFQ 2XFR 2XFS 2XFT 2XFU 2XFV 2XFW 2XFX 2XFY 2XG3 2XG4 2XG5 2XG6 2XG7 2XG8 2XG9 2XGA 2XGB 2XGC 2XGD 2XGE 2XGF 2XGG 2XGI 2XGK 2XGL 2XGM 2XGN 2XGO 2XGR 2XGS 2XGT 2XGU 2XGV 2XGW 2XGX 2XGY 2XGZ 2XH0 2XH1 2XH2 2XH3 2XH4 2XH5 2XH6 2XH7 2XH8 2XH9 2XHA 2XHC 2XHD 2XHE 2XHF 2XHG 2XHH 2XHJ 2XHK 2XHL 2XHM 2XHN 2XHR 2XHS 2XHT 2XHU 2XHV 2XHW 2XHX 2XHY 2XHZ 2XI1 2XI2 2XI3 2XI4 2XI5 2XI6 2XI7 2XI8 2XI9 2XIB 2XIC 2XID 2XIF 2XIG 2XIH 2XII 2XIJ 2XIK 2XIL 2XIM 2XIN 2XIO 2XIQ 2XIR 2XIS 2XIT 2XIU 2XIV 2XIW 2XIX 2XIY 2XIZ 2XJ0 2XJ1 2XJ2 2XJ3 2XJ4 2XJ5 2XJ6 2XJ7 2XJ8 2XJ9 2XJA 2XJB 2XJC 2XJD 2XJE 2XJF 2XJG 2XJH 2XJI 2XJJ 2XJK 2XJL 2XJM 2XJN 2XJO 2XJP 2XJQ 2XJR 2XJS 2XJT 2XJU 2XJV 2XJW 2XJX 2XJY 2XJZ 2XK1 2XK2 2XK3 2XK4 2XK5 2XK6 2XK7 2XK8 2XK9 2XKA 2XKB 2XKC 2XKD 2XKE 2XKF 2XKG 2XKH 2XKI 2XKJ 2XKL 2XKN 2XKO 2XKP 2XKQ 2XKR 2XKW 2XL2 2XL3 2XL4 2XL6 2XL7 2XL8 2XL9 2XLA 2XLB 2XLC 2XLD 2XLE 2XLF 2XLG 2XLH 2XLL 2XLM 2XLN 2XLO 2XLP 2XLQ 2XLR 2XLS 2XLT 2XLU 2XLV 2XLW 2XLY 2XM0 2XM1 2XM2 2XM4 2XM5 2XM7 2XM8 2XM9 2XMB 2XMC 2XMD 2XME 2XMF 2XMG 2XMH 2XMI 2XMJ 2XMK 2XML 2XMM 2XMN 2XMO 2XMP 2XMQ 2XMR 2XMS 2XMT 2XMU 2XMV 2XMW 2XMX 2XMY 2XMZ 2XN0 2XN1 2XN2 2XN3 2XN4 2XN5 2XN6 2XN7 2XN8 2XN9 2XNA 2XNB 2XNC 2XND 2XNE 2XNG 2XNH 2XNI 2XNJ 2XNK 2XNM 2XNN 2XNO 2XNP 2XNQ 2XNS 2XNT 2XNU 2XNV 2XNX 2XNY 2XO2 2XO3 2XO4 2XO5 2XO8 2XOA 2XOC 2XOD 2XOE 2XOF 2XOG 2XOI 2XOK 2XOL 2XOM 2XON 2XOT 2XOV 2XOW 2XOX 2XOY 2XOZ 2XP0 2XP1 2XP2 2XP3 2XP4 2XP5 2XP6 2XP7 2XP8 2XP9 2XPA 2XPB 2XPC 2XPD 2XPE 2XPG 2XPH 2XPI 2XPK 2XPL 2XPN 2XPO 2XPP 2XPU 2XPV 2XPW 2XPX 2XPY 2XPZ 2XQ0 2XQ1 2XQ2 2XQ3 2XQ4 2XQ5 2XQ6 2XQ7 2XQ8 2XQ9 2XQA 2XQB 2XQF 2XQG 2XQH 2XQI 2XQJ 2XQK 2XQN 2XQO 2XQQ 2XQR 2XQS 2XQT 2XQU 2XQV 2XQW 2XQX 2XQY 2XR0 2XR1 2XR4 2XR5 2XR6 2XR7 2XR8 2XR9 2XRA 2XRB 2XRC 2XRD 2XRE 2XRF 2XRG 2XRH 2XRI 2XRL 2XRM 2XRN 2XRQ 2XRS 2XRU 2XRW 2XRX 2XRY 2XS0 2XS1 2XS3 2XS4 2XS6 2XS8 2XSA 2XSB 2XSC 2XSE 2XSF 2XSG 2XSH 2XSI 2XSJ 2XSK 2XSM 2XSN 2XSO 2XSP 2XSQ 2XSR 2XSS 2XST 2XSU 2XSV 2XSW 2XSX 2XSZ 2XT0 2XT1 2XT2 2XT3 2XT4 2XT6 2XT9 2XTA 2XTB 2XTC 2XTD 2XTE 2XTH 2XTI 2XTJ 2XTK 2XTL 2XTM 2XTN 2XTO 2XTP 2XTQ 2XTR 2XTS 2XTT 2XTU 2XTV 2XTW 2XTX 2XTY 2XTZ 2XU0 2XU1 2XU2 2XU3 2XU4 2XU5 2XU6 2XU7 2XU8 2XU9 2XUA 2XUB 2XUC 2XUD 2XUE 2XUF 2XUG 2XUH 2XUI 2XUJ 2XUK 2XUL 2XUM 2XUO 2XUP 2XUQ 2XUR 2XUS 2XUT 2XUU 2XUV 2XUW 2XUZ 2XV0 2XV1 2XV2 2XV3 2XV4 2XV5 2XV6 2XV7 2XVA 2XVB 2XVC 2XVD 2XVE 2XVF 2XVG 2XVH 2XVI 2XVJ 2XVK 2XVL 2XVM 2XVN 2XVO 2XVP 2XVQ 2XVS 2XVT 2XVU 2XVV 2XVW 2XVX 2XVY 2XVZ 2XW0 2XW1 2XW6 2XW7 2XW9 2XWA 2XWB 2XWC 2XWD 2XWE 2XWG 2XWH 2XWI 2XWJ 2XWK 2XWL 2XWM 2XWN 2XWO 2XWP 2XWQ 2XWR 2XWS 2XWT 2XWU 2XWV 2XWX 2XWY 2XWZ 2XX0 2XX1 2XX2 2XX3 2XX4 2XX5 2XX6 2XX7 2XX8 2XX9 2XXB 2XXC 2XXD 2XXF 2XXG 2XXH 2XXI 2XXJ 2XXK 2XXL 2XXM 2XXN 2XXP 2XXQ 2XXR 2XXT 2XXU 2XXV 2XXW 2XXX 2XXY 2XXZ 2XY1 2XY2 2XY3 2XY4 2XY9 2XYA 2XYB 2XYC 2XYD 2XYE 2XYF 2XYG 2XYH 2XYI 2XYJ 2XYK 2XYL 2XYM 2XYN 2XYO 2XYP 2XYQ 2XYR 2XYS 2XYT 2XYU 2XYV 2XYW 2XYX 2XZ0 2XZ1 2XZ2 2XZ3 2XZ4 2XZ5 2XZ6 2XZ7 2XZ8 2XZ9 2XZA 2XZC 2XZD 2XZE 2XZG 2XZI 2XZJ 2XZK 2XZP 2XZQ 2XZR 2XZS 2XZT 2XZV 2XZW 2XZZ 2Y00 2Y01 2Y02 2Y03 2Y04 2Y05 2Y06 2Y07 2Y08 2Y09 2Y0A 2Y0B 2Y0C 2Y0D 2Y0E 2Y0F 2Y0G 2Y0H 2Y0I 2Y0J 2Y0K 2Y0L 2Y0M 2Y0N 2Y0O 2Y0P 2Y0Q 2Y0R 2Y0S 2Y0T 2Y1A 2Y1B 2Y1C 2Y1D 2Y1E 2Y1F 2Y1G 2Y1H 2Y1K 2Y1L 2Y1M 2Y1N 2Y1O 2Y1P 2Y1Q 2Y1R 2Y1T 2Y1V 2Y1W 2Y1X 2Y1Y 2Y1Z 2Y20 2Y21 2Y22 2Y23 2Y24 2Y25 2Y26 2Y27 2Y28 2Y29 2Y2A 2Y2B 2Y2C 2Y2D 2Y2E 2Y2F 2Y2G 2Y2H 2Y2I 2Y2J 2Y2K 2Y2L 2Y2M 2Y2N 2Y2O 2Y2P 2Y2Q 2Y2T 2Y2U 2Y2V 2Y2W 2Y2X 2Y2Y 2Y2Z 2Y30 2Y31 2Y32 2Y33 2Y34 2Y36 2Y37 2Y38 2Y39 2Y3A 2Y3B 2Y3C 2Y3D 2Y3E 2Y3F 2Y3G 2Y3H 2Y3I 2Y3J 2Y3K 2Y3L 2Y3M 2Y3N 2Y3P 2Y3Q 2Y3R 2Y3S 2Y3U 2Y3V 2Y3W 2Y3X 2Y3Y 2Y3Z 2Y40 2Y41 2Y42 2Y43 2Y44 2Y46 2Y48 2Y4A 2Y4D 2Y4E 2Y4F 2Y4G 2Y4I 2Y4J 2Y4K 2Y4L 2Y4M 2Y4N 2Y4O 2Y4P 2Y4R 2Y4S 2Y4T 2Y4U 2Y4V 2Y4X 2Y4Y 2Y4Z 2Y50 2Y51 2Y52 2Y53 2Y54 2Y55 2Y56 2Y57 2Y58 2Y59 2Y5A 2Y5B 2Y5C 2Y5D 2Y5E 2Y5F 2Y5G 2Y5H 2Y5I 2Y5J 2Y5K 2Y5L 2Y5M 2Y5N 2Y5P 2Y5Q 2Y5S 2Y5T 2Y5W 2Y5Y 2Y5Z 2Y60 2Y61 2Y62 2Y63 2Y64 2Y65 2Y66 2Y67 2Y68 2Y69 2Y6A 2Y6B 2Y6C 2Y6D 2Y6E 2Y6F 2Y6G 2Y6H 2Y6I 2Y6J 2Y6K 2Y6L 2Y6M 2Y6N 2Y6O 2Y6P 2Y6Q 2Y6R 2Y6S 2Y6T 2Y6U 2Y6V 2Y6W 2Y6X 2Y6Y 2Y6Z 2Y70 2Y71 2Y72 2Y73 2Y74 2Y75 2Y76 2Y77 2Y78 2Y79 2Y7A 2Y7B 2Y7D 2Y7E 2Y7F 2Y7G 2Y7I 2Y7J 2Y7K 2Y7L 2Y7M 2Y7N 2Y7O 2Y7P 2Y7Q 2Y7R 2Y7S 2Y7W 2Y7X 2Y7Y 2Y7Z 2Y80 2Y81 2Y82 2Y84 2Y85 2Y87 2Y88 2Y89 2Y8A 2Y8B 2Y8C 2Y8D 2Y8E 2Y8F 2Y8G 2Y8H 2Y8I 2Y8K 2Y8L 2Y8N 2Y8O 2Y8P 2Y8Q 2Y8R 2Y8S 2Y8T 2Y8U 2Y8V 2Y90 2Y91 2Y92 2Y93 2Y96 2Y98 2Y99 2Y9E 2Y9F 2Y9G 2Y9M 2Y9N 2Y9P 2Y9Q 2Y9R 2Y9U 2Y9W 2Y9X 2Y9Y 2YA0 2YA1 2YA2 2YA3 2YA4 2YA5 2YA6 2YA7 2YA8 2YA9 2YAA 2YAB 2YAC 2YAD 2YAE 2YAF 2YAH 2YAJ 2YAK 2YAL 2YAM 2YAN 2YAO 2YAP 2YAQ 2YAR 2YAS 2YAT 2YAU 2YAV 2YAW 2YAX 2YAY 2YAZ 2YB0 2YB1 2YB4 2YB5 2YB6 2YB7 2YB8 2YB9 2YBA 2YBD 2YBE 2YBF 2YBG 2YBH 2YBI 2YBJ 2YBK 2YBL 2YBM 2YBN 2YBO 2YBP 2YBQ 2YBR 2YBS 2YBT 2YBU 2YBV 2YBX 2YBY 2YC0 2YC1 2YC2 2YC3 2YC4 2YC5 2YCA 2YCB 2YCC 2YCD 2YCE 2YCF 2YCG 2YCH 2YCI 2YCJ 2YCK 2YCL 2YCM 2YCN 2YCP 2YCQ 2YCR 2YCS 2YCT 2YCU 2YCW 2YCX 2YCY 2YCZ 2YD0 2YD1 2YD2 2YD3 2YD4 2YD5 2YD6 2YD7 2YD8 2YD9 2YDA 2YDB 2YDC 2YDD 2YDE 2YDF 2YDG 2YDI 2YDJ 2YDK 2YDL 2YDM 2YDO 2YDP 2YDQ 2YDR 2YDS 2YDT 2YDU 2YDV 2YDW 2YDX 2YDY 2YDZ 2YE0 2YE1 2YE2 2YE3 2YE4 2YE5 2YE6 2YE7 2YE8 2YE9 2YEA 2YEB 2YEC 2YED 2YEE 2YEF 2YEG 2YEH 2YEI 2YEJ 2YEK 2YEL 2YEM 2YEO 2YEP 2YEQ 2YER 2YES 2YET 2YEU 2YEV 2YEX 2YEY 2YEZ 2YF0 2YF1 2YF2 2YF3 2YF4 2YF9 2YFA 2YFB 2YFC 2YFD 2YFE 2YFH 2YFI 2YFJ 2YFK 2YFL 2YFN 2YFO 2YFP 2YFQ 2YFR 2YFS 2YFT 2YFU 2YFV 2YFW 2YFX 2YFY 2YFZ 2YG0 2YG1 2YG2 2YG3 2YG4 2YG5 2YG6 2YG7 2YG8 2YG9 2YGA 2YGB 2YGC 2YGE 2YGF 2YGG 2YGI 2YGJ 2YGK 2YGL 2YGM 2YGN 2YGO 2YGP 2YGQ 2YGS 2YGT 2YGU 2YGV 2YGW 2YGX 2YGY 2YH2 2YH3 2YH5 2YH6 2YH9 2YHA 2YHB 2YHC 2YHD 2YHE 2YHF 2YHG 2YHI 2YHJ 2YHK 2YHN 2YHO 2YHS 2YHT 2YHU 2YHV 2YHW 2YHX 2YHY 2YI0 2YI1 2YI5 2YI6 2YI7 2YI8 2YI9 2YIA 2YIB 2YIC 2YID 2YIG 2YIH 2YII 2YIJ 2YIK 2YIL 2YIM 2YIN 2YIO 2YIP 2YIQ 2YIR 2YIS 2YIT 2YIU 2YIV 2YIW 2YIX 2YIY 2YIZ 2YJ0 2YJ1 2YJ2 2YJ3 2YJ4 2YJ5 2YJ6 2YJ7 2YJ8 2YJ9 2YJA 2YJB 2YJC 2YJD 2YJE 2YJF 2YJG 2YJH 2YJJ 2YJK 2YJL 2YJM 2YJN 2YJP 2YJQ 2YJR 2YJS 2YJT 2YJV 2YJW 2YJX 2YJZ 2YK0 2YK1 2YK2 2YK3 2YK4 2YK5 2YK6 2YK7 2YK9 2YKB 2YKC 2YKD 2YKE 2YKF 2YKH 2YKI 2YKJ 2YKK 2YKL 2YKM 2YKN 2YKO 2YKP 2YKQ 2YKS 2YKT 2YKU 2YKV 2YKX 2YKY 2YKZ 2YL0 2YL1 2YL2 2YL3 2YL5 2YL6 2YL7 2YL8 2YL9 2YLA 2YLB 2YLC 2YLD 2YLE 2YLF 2YLG 2YLH 2YLI 2YLJ 2YLK 2YLL 2YLM 2YLN 2YLO 2YLP 2YLQ 2YLR 2YLS 2YLT 2YLW 2YLX 2YLY 2YLZ 2YM0 2YM1 2YM2 2YM3 2YM4 2YM5 2YM6 2YM7 2YM8 2YM9 2YMA 2YMB 2YMD 2YME 2YMK 2YML 2YMM 2YMO 2YMP 2YMQ 2YMS 2YMT 2YMU 2YMV 2YMW 2YMX 2YMY 2YMZ 2YN0 2YN1 2YN2 2YN3 2YN4 2YN5 2YN6 2YN7 2YN8 2YNA 2YNB 2YNC 2YND 2YNE 2YNF 2YNG 2YNH 2YNI 2YNK 2YNM 2YNN 2YNO 2YNP 2YNQ 2YNR 2YNS 2YNT 2YNU 2YNV 2YNW 2YNX 2YNY 2YNZ 2YO0 2YO1 2YO2 2YO3 2YOA 2YOB 2YOC 2YOE 2YOF 2YOG 2YOH 2YOI 2YOJ 2YOK 2YOL 2YOO 2YOP 2YOQ 2YOR 2YOW 2YOX 2YOY 2YOZ 2YP0 2YP1 2YP2 2YP3 2YP4 2YP5 2YP6 2YP7 2YP8 2YP9 2YPC 2YPD 2YPE 2YPG 2YPH 2YPI 2YPJ 2YPK 2YPL 2YPM 2YPN 2YPO 2YPP 2YPQ 2YPR 2YPS 2YPT 2YPU 2YPV 2YPY 2YPZ 2YQ0 2YQ1 2YQ2 2YQ3 2YQ4 2YQ5 2YQ6 2YQ7 2YQ8 2YQ9 2YQB 2YQC 2YQH 2YQJ 2YQS 2YQU 2YQY 2YQZ 2YR0 2YR1 2YR2 2YR4 2YR5 2YR6 2YRF 2YRI 2YRR 2YRS 2YRW 2YRX 2YS6 2YS7 2YSK 2YSS 2YSU 2YSW 2YT4 2YTZ 2YU1 2YU2 2YUT 2YV0 2YV1 2YV2 2YV3 2YV4 2YV5 2YV6 2YV7 2YV8 2YV9 2YVA 2YVB 2YVC 2YVE 2YVF 2YVG 2YVI 2YVJ 2YVK 2YVL 2YVM 2YVN 2YVO 2YVP 2YVQ 2YVR 2YVS 2YVT 2YVU 2YVV 2YVW 2YVX 2YVY 2YVZ 2YW0 2YW2 2YW3 2YW4 2YW6 2YW7 2YW8 2YW9 2YWA 2YWB 2YWC 2YWD 2YWE 2YWF 2YWG 2YWH 2YWI 2YWJ 2YWK 2YWL 2YWM 2YWN 2YWO 2YWP 2YWQ 2YWR 2YWV 2YWW 2YWX 2YWY 2YWZ 2YX0 2YX1 2YX2 2YX4 2YX5 2YX6 2YX7 2YX8 2YX9 2YXB 2YXC 2YXD 2YXE 2YXF 2YXG 2YXH 2YXJ 2YXL 2YXM 2YXN 2YXO 2YXP 2YXQ 2YXR 2YXS 2YXT 2YXU 2YXV 2YXW 2YXX 2YXY 2YXZ 2YY0 2YY1 2YY2 2YY3 2YY4 2YY5 2YY6 2YY7 2YY8 2YY9 2YYA 2YYB 2YYE 2YYG 2YYH 2YYI 2YYJ 2YYK 2YYL 2YYM 2YYN 2YYO 2YYR 2YYS 2YYT 2YYU 2YYV 2YYW 2YYX 2YYY 2YYZ 2YZ1 2YZ2 2YZ3 2YZ5 2YZ7 2YZ8 2YZA 2YZB 2YZC 2YZD 2YZE 2YZG 2YZH 2YZI 2YZJ 2YZK 2YZL 2YZM 2YZN 2YZO 2YZQ 2YZR 2YZS 2YZT 2YZU 2YZV 2YZW 2YZY 2Z00 2Z01 2Z02 2Z04 2Z06 2Z07 2Z08 2Z09 2Z0A 2Z0B 2Z0D 2Z0E 2Z0F 2Z0G 2Z0I 2Z0J 2Z0K 2Z0L 2Z0M 2Z0N 2Z0O 2Z0P 2Z0Q 2Z0R 2Z0S 2Z0T 2Z0U 2Z0V 2Z0W 2Z0X 2Z0Y 2Z0Z 2Z10 2Z11 2Z12 2Z13 2Z14 2Z15 2Z16 2Z17 2Z18 2Z19 2Z1A 2Z1B 2Z1C 2Z1D 2Z1E 2Z1F 2Z1G 2Z1H 2Z1I 2Z1J 2Z1K 2Z1M 2Z1N 2Z1O 2Z1P 2Z1Q 2Z1S 2Z1T 2Z1U 2Z1V 2Z1W 2Z1X 2Z1Y 2Z1Z 2Z20 2Z21 2Z22 2Z23 2Z24 2Z25 2Z26 2Z27 2Z28 2Z29 2Z2A 2Z2B 2Z2C 2Z2E 2Z2F 2Z2I 2Z2J 2Z2K 2Z2L 2Z2M 2Z2N 2Z2O 2Z2P 2Z2R 2Z2S 2Z2T 2Z2U 2Z2W 2Z2X 2Z2Y 2Z2Z 2Z30 2Z31 2Z32 2Z34 2Z35 2Z36 2Z37 2Z38 2Z39 2Z3A 2Z3B 2Z3C 2Z3D 2Z3E 2Z3F 2Z3G 2Z3H 2Z3I 2Z3J 2Z3K 2Z3L 2Z3M 2Z3N 2Z3O 2Z3P 2Z3Q 2Z3R 2Z3T 2Z3U 2Z3V 2Z3W 2Z3Y 2Z3Z 2Z41 2Z42 2Z43 2Z44 2Z45 2Z46 2Z47 2Z48 2Z49 2Z4B 2Z4E 2Z4G 2Z4H 2Z4I 2Z4J 2Z4O 2Z4P 2Z4Q 2Z4R 2Z4S 2Z4T 2Z4U 2Z4V 2Z4W 2Z4X 2Z4Y 2Z4Z 2Z50 2Z51 2Z52 2Z53 2Z54 2Z55 2Z56 2Z57 2Z58 2Z5B 2Z5C 2Z5D 2Z5E 2Z5F 2Z5G 2Z5H 2Z5I 2Z5J 2Z5K 2Z5L 2Z5M 2Z5N 2Z5O 2Z5P 2Z5Q 2Z5R 2Z5S 2Z5T 2Z5U 2Z5W 2Z5X 2Z5Y 2Z5Z 2Z60 2Z61 2Z62 2Z63 2Z64 2Z65 2Z66 2Z67 2Z68 2Z69 2Z6B 2Z6C 2Z6D 2Z6E 2Z6F 2Z6G 2Z6H 2Z6I 2Z6J 2Z6K 2Z6M 2Z6N 2Z6O 2Z6P 2Z6R 2Z6S 2Z6T 2Z6V 2Z6W 2Z6X 2Z6Y 2Z6Z 2Z71 2Z72 2Z73 2Z76 2Z77 2Z78 2Z79 2Z7A 2Z7B 2Z7C 2Z7E 2Z7F 2Z7G 2Z7H 2Z7I 2Z7J 2Z7K 2Z7L 2Z7Q 2Z7R 2Z7S 2Z7U 2Z7W 2Z7X 2Z7Y 2Z7Z 2Z80 2Z81 2Z82 2Z83 2Z84 2Z85 2Z86 2Z87 2Z8A 2Z8C 2Z8D 2Z8E 2Z8F 2Z8G 2Z8H 2Z8I 2Z8J 2Z8K 2Z8L 2Z8M 2Z8N 2Z8O 2Z8P 2Z8Q 2Z8R 2Z8S 2Z8U 2Z8V 2Z8W 2Z8X 2Z8Y 2Z8Z 2Z90 2Z91 2Z92 2Z93 2Z94 2Z95 2Z97 2Z98 2Z99 2Z9A 2Z9B 2Z9C 2Z9D 2Z9G 2Z9H 2Z9I 2Z9J 2Z9K 2Z9L 2Z9N 2Z9S 2Z9T 2Z9U 2Z9V 2Z9W 2Z9X 2Z9Y 2Z9Z 2ZA0 2ZA1 2ZA2 2ZA3 2ZA4 2ZA5 2ZA6 2ZA7 2ZA8 2ZA9 2ZAA 2ZAB 2ZAC 2ZAD 2ZAE 2ZAF 2ZAG 2ZAH 2ZAI 2ZAK 2ZAL 2ZAM 2ZAN 2ZAO 2ZAS 2ZAT 2ZAU 2ZAV 2ZAW 2ZAX 2ZAY 2ZAZ 2ZB0 2ZB1 2ZB2 2ZB3 2ZB4 2ZB5 2ZB6 2ZB7 2ZB8 2ZB9 2ZBA 2ZBB 2ZBC 2ZBD 2ZBE 2ZBF 2ZBG 2ZBH 2ZBI 2ZBJ 2ZBK 2ZBL 2ZBM 2ZBN 2ZBO 2ZBP 2ZBQ 2ZBR 2ZBS 2ZBT 2ZBU 2ZBV 2ZBW 2ZBX 2ZBY 2ZBZ 2ZC0 2ZC1 2ZC2 2ZC3 2ZC4 2ZC5 2ZC6 2ZC7 2ZC8 2ZC9 2ZCA 2ZCB 2ZCC 2ZCE 2ZCF 2ZCG 2ZCH 2ZCI 2ZCK 2ZCL 2ZCM 2ZCN 2ZCO 2ZCQ 2ZCR 2ZCS 2ZCT 2ZCU 2ZCV 2ZCW 2ZCX 2ZCY 2ZCZ 2ZD0 2ZD1 2ZD2 2ZD7 2ZD8 2ZD9 2ZDA 2ZDC 2ZDG 2ZDH 2ZDI 2ZDJ 2ZDK 2ZDL 2ZDM 2ZDN 2ZDO 2ZDP 2ZDQ 2ZDR 2ZDS 2ZDT 2ZDU 2ZDV 2ZDX 2ZDY 2ZDZ 2ZE0 2ZE1 2ZE2 2ZE3 2ZE4 2ZE5 2ZE6 2ZE7 2ZE8 2ZE9 2ZEB 2ZEC 2ZED 2ZEE 2ZEF 2ZEG 2ZEH 2ZEJ 2ZEL 2ZEM 2ZEN 2ZEO 2ZEP 2ZEQ 2ZET 2ZEU 2ZEV 2ZEW 2ZEX 2ZEY 2ZEZ 2ZF0 2ZF3 2ZF4 2ZF5 2ZF8 2ZF9 2ZFA 2ZFB 2ZFC 2ZFD 2ZFE 2ZFF 2ZFG 2ZFH 2ZFI 2ZFJ 2ZFK 2ZFL 2ZFM 2ZFN 2ZFO 2ZFP 2ZFQ 2ZFR 2ZFS 2ZFT 2ZFU 2ZFW 2ZFX 2ZFY 2ZFZ 2ZG0 2ZG1 2ZG2 2ZG3 2ZG6 2ZG7 2ZG8 2ZG9 2ZGA 2ZGB 2ZGC 2ZGD 2ZGG 2ZGH 2ZGI 2ZGJ 2ZGK 2ZGL 2ZGM 2ZGN 2ZGO 2ZGP 2ZGQ 2ZGR 2ZGS 2ZGT 2ZGU 2ZGV 2ZGW 2ZGX 2ZGY 2ZGZ 2ZHD 2ZHE 2ZHF 2ZHH 2ZHI 2ZHJ 2ZHK 2ZHL 2ZHM 2ZHN 2ZHO 2ZHP 2ZHQ 2ZHR 2ZHS 2ZHT 2ZHU 2ZHV 2ZHW 2ZHX 2ZHY 2ZHZ 2ZI2 2ZI3 2ZI4 2ZI5 2ZI6 2ZI7 2ZI8 2ZI9 2ZIA 2ZIB 2ZIC 2ZID 2ZIE 2ZIF 2ZIG 2ZIH 2ZII 2ZIJ 2ZIK 2ZIL 2ZIM 2ZIN 2ZIO 2ZIQ 2ZIR 2ZIS 2ZIT 2ZIU 2ZIV 2ZIW 2ZIX 2ZIY 2ZIZ 2ZJ0 2ZJ1 2ZJ2 2ZJ3 2ZJ4 2ZJ5 2ZJ6 2ZJ7 2ZJ8 2ZJ9 2ZJA 2ZJB 2ZJC 2ZJD 2ZJF 2ZJG 2ZJH 2ZJI 2ZJJ 2ZJK 2ZJL 2ZJM 2ZJN 2ZJO 2ZJS 2ZJT 2ZJU 2ZJV 2ZJW 2ZJX 2ZJY 2ZJZ 2ZK0 2ZK1 2ZK2 2ZK3 2ZK4 2ZK5 2ZK6 2ZK7 2ZK9 2ZKA 2ZKB 2ZKC 2ZKG 2ZKH 2ZKI 2ZKJ 2ZKL 2ZKM 2ZKN 2ZKS 2ZKT 2ZKU 2ZKW 2ZKX 2ZKY 2ZKZ 2ZL0 2ZL1 2ZL2 2ZL3 2ZL4 2ZL5 2ZL6 2ZL7 2ZL8 2ZL9 2ZLA 2ZLB 2ZLC 2ZLD 2ZLF 2ZLG 2ZLT 2ZLU 2ZLV 2ZLW 2ZLX 2ZLY 2ZM0 2ZM1 2ZM2 2ZM3 2ZM4 2ZM7 2ZM8 2ZM9 2ZMA 2ZMB 2ZMC 2ZMD 2ZME 2ZMF 2ZMH 2ZMI 2ZMJ 2ZMK 2ZML 2ZMM 2ZMN 2ZMU 2ZMV 2ZMW 2ZMX 2ZMY 2ZMZ 2ZN7 2ZN8 2ZN9 2ZNB 2ZNC 2ZND 2ZNE 2ZNH 2ZNJ 2ZNK 2ZNL 2ZNM 2ZNN 2ZNO 2ZNP 2ZNQ 2ZNR 2ZNS 2ZNT 2ZNU 2ZNV 2ZNW 2ZNX 2ZNY 2ZNZ 2ZO3 2ZO4 2ZO5 2ZO6 2ZO7 2ZO9 2ZOA 2ZOC 2ZOD 2ZOE 2ZOF 2ZOG 2ZOH 2ZOK 2ZOL 2ZOM 2ZON 2ZOO 2ZOP 2ZOQ 2ZOS 2ZOT 2ZOU 2ZOV 2ZOW 2ZOX 2ZOY 2ZOZ 2ZP0 2ZP1 2ZP2 2ZP3 2ZP4 2ZP5 2ZP6 2ZP7 2ZP8 2ZP9 2ZPA 2ZPB 2ZPC 2ZPD 2ZPE 2ZPF 2ZPG 2ZPH 2ZPI 2ZPK 2ZPL 2ZPM 2ZPN 2ZPO 2ZPQ 2ZPR 2ZPS 2ZPT 2ZPU 2ZPX 2ZPY 2ZQ0 2ZQ1 2ZQ2 2ZQ3 2ZQ4 2ZQ5 2ZQ7 2ZQ8 2ZQ9 2ZQA 2ZQB 2ZQC 2ZQD 2ZQE 2ZQJ 2ZQK 2ZQM 2ZQN 2ZQO 2ZQP 2ZQQ 2ZQR 2ZQS 2ZQT 2ZQU 2ZQV 2ZQX 2ZQY 2ZQZ 2ZR0 2ZR1 2ZR2 2ZR3 2ZR4 2ZR5 2ZR6 2ZR7 2ZR8 2ZR9 2ZRA 2ZRB 2ZRC 2ZRD 2ZRE 2ZRF 2ZRG 2ZRH 2ZRI 2ZRJ 2ZRK 2ZRL 2ZRM 2ZRN 2ZRO 2ZRP 2ZRQ 2ZRR 2ZRS 2ZRT 2ZRU 2ZRV 2ZRW 2ZRX 2ZRY 2ZRZ 2ZS0 2ZS1 2ZS6 2ZS7 2ZS8 2ZS9 2ZSA 2ZSB 2ZSC 2ZSD 2ZSE 2ZSF 2ZSG 2ZSH 2ZSI 2ZSJ 2ZSK 2ZSL 2ZSM 2ZSN 2ZSO 2ZSP 2ZSQ 2ZSR 2ZSS 2ZST 2ZSU 2ZSV 2ZSW 2ZSX 2ZSY 2ZSZ 2ZT0 2ZT1 2ZT2 2ZT3 2ZT4 2ZT5 2ZT6 2ZT7 2ZT8 2ZT9 2ZTA 2ZTB 2ZTC 2ZTD 2ZTE 2ZTG 2ZTH 2ZTI 2ZTJ 2ZTK 2ZTL 2ZTM 2ZTN 2ZTS 2ZTT 2ZTU 2ZTV 2ZTW 2ZTX 2ZTY 2ZTZ 2ZU0 2ZU1 2ZU2 2ZU3 2ZU4 2ZU5 2ZU6 2ZU7 2ZU8 2ZU9 2ZUA 2ZUB 2ZUC 2ZUD 2ZUG 2ZUH 2ZUI 2ZUJ 2ZUK 2ZUL 2ZUM 2ZUN 2ZUP 2ZUQ 2ZUR 2ZUS 2ZUT 2ZUU 2ZUV 2ZUW 2ZUX 2ZUY 2ZV2 2ZV3 2ZV6 2ZV7 2ZV8 2ZV9 2ZVA 2ZVB 2ZVC 2ZVD 2ZVF 2ZVI 2ZVJ 2ZVK 2ZVL 2ZVM 2ZVN 2ZVO 2ZVP 2ZVQ 2ZVR 2ZVS 2ZVT 2ZVU 2ZVV 2ZVW 2ZVX 2ZVY 2ZVZ 2ZW0 2ZW1 2ZW2 2ZW3 2ZW4 2ZW5 2ZW6 2ZW7 2ZW9 2ZWA 2ZWD 2ZWE 2ZWF 2ZWG 2ZWI 2ZWJ 2ZWK 2ZWL 2ZWM 2ZWN 2ZWO 2ZWP 2ZWR 2ZWS 2ZWT 2ZWU 2ZWV 2ZWY 2ZWZ 2ZX0 2ZX1 2ZX2 2ZX3 2ZX4 2ZX5 2ZX6 2ZX7 2ZX8 2ZX9 2ZXA 2ZXB 2ZXC 2ZXD 2ZXE 2ZXF 2ZXG 2ZXH 2ZXI 2ZXJ 2ZXK 2ZXL 2ZXM 2ZXN 2ZXO 2ZXP 2ZXQ 2ZXR 2ZXT 2ZXV 2ZXW 2ZXX 2ZXY 2ZXZ 2ZY0 2ZY1 2ZY2 2ZY3 2ZY4 2ZY5 2ZY9 2ZYA 2ZYB 2ZYC 2ZYD 2ZYF 2ZYG 2ZYH 2ZYI 2ZYJ 2ZYK 2ZYL 2ZYM 2ZYN 2ZYO 2ZYP 2ZYQ 2ZYR 2ZYS 2ZYT 2ZYU 2ZYV 2ZYW 2ZYZ 2ZZ0 2ZZ1 2ZZ2 2ZZ3 2ZZ4 2ZZ5 2ZZ6 2ZZ7 2ZZ8 2ZZA 2ZZB 2ZZC 2ZZD 2ZZE 2ZZF 2ZZG 2ZZI 2ZZJ 2ZZK 2ZZL 2ZZO 2ZZP 2ZZQ 2ZZR 2ZZS 2ZZT 2ZZU 2ZZV 2ZZW 2ZZX 31BI 32C2 351C 35C8 3A02 3A03 3A04 3A05 3A06 3A07 3A08 3A09 3A0A 3A0B 3A0C 3A0D 3A0E 3A0F 3A0G 3A0H 3A0I 3A0J 3A0K 3A0M 3A0N 3A0O 3A0R 3A0S 3A0T 3A0U 3A0V 3A0W 3A0X 3A0Y 3A0Z 3A10 3A11 3A12 3A13 3A14 3A15 3A16 3A17 3A18 3A19 3A1A 3A1B 3A1C 3A1D 3A1E 3A1F 3A1G 3A1H 3A1I 3A1J 3A1K 3A1L 3A1M 3A1N 3A1P 3A1Q 3A1S 3A1T 3A1U 3A1V 3A1W 3A1Y 3A1Z 3A20 3A21 3A22 3A23 3A24 3A25 3A26 3A27 3A28 3A29 3A2A 3A2B 3A2C 3A2E 3A2F 3A2G 3A2H 3A2I 3A2J 3A2L 3A2M 3A2N 3A2O 3A2P 3A2Q 3A2S 3A2V 3A2W 3A2X 3A2Y 3A2Z 3A30 3A31 3A32 3A33 3A34 3A35 3A36 3A37 3A38 3A39 3A3B 3A3C 3A3D 3A3E 3A3F 3A3G 3A3H 3A3I 3A3J 3A3K 3A3N 3A3O 3A3P 3A3Q 3A3R 3A3T 3A3U 3A3V 3A3W 3A3X 3A3Y 3A3Z 3A40 3A42 3A43 3A44 3A45 3A47 3A4A 3A4C 3A4D 3A4E 3A4F 3A4G 3A4H 3A4I 3A4J 3A4L 3A4M 3A4N 3A4O 3A4P 3A4R 3A4S 3A4T 3A4U 3A4V 3A4W 3A4X 3A4Y 3A4Z 3A50 3A51 3A52 3A54 3A55 3A56 3A57 3A58 3A59 3A5A 3A5B 3A5C 3A5D 3A5E 3A5F 3A5G 3A5I 3A5J 3A5K 3A5L 3A5M 3A5N 3A5O 3A5P 3A5Q 3A5R 3A5S 3A5V 3A5W 3A5Y 3A5Z 3A60 3A61 3A62 3A64 3A65 3A66 3A67 3A68 3A6B 3A6C 3A6D 3A6E 3A6F 3A6G 3A6H 3A6J 3A6K 3A6L 3A6M 3A6O 3A6Q 3A6R 3A6S 3A6T 3A6U 3A6V 3A6Z 3A70 3A71 3A72 3A73 3A74 3A75 3A76 3A77 3A78 3A79 3A7A 3A7B 3A7C 3A7D 3A7E 3A7F 3A7G 3A7H 3A7I 3A7J 3A7K 3A7L 3A7M 3A7N 3A7O 3A7P 3A7Q 3A7R 3A7S 3A7T 3A7U 3A7V 3A7W 3A7X 3A7Y 3A7Z 3A80 3A81 3A82 3A83 3A84 3A85 3A86 3A87 3A88 3A89 3A8A 3A8B 3A8C 3A8D 3A8E 3A8G 3A8H 3A8I 3A8J 3A8K 3A8L 3A8M 3A8N 3A8O 3A8P 3A8Q 3A8R 3A8S 3A8T 3A8U 3A8W 3A8X 3A8Y 3A8Z 3A90 3A91 3A92 3A93 3A94 3A95 3A96 3A98 3A99 3A9B 3A9C 3A9E 3A9F 3A9G 3A9H 3A9I 3A9J 3A9K 3A9L 3A9M 3A9Q 3A9R 3A9S 3A9T 3A9U 3A9V 3A9W 3A9X 3A9Y 3A9Z 3AA0 3AA1 3AA2 3AA3 3AA4 3AA5 3AA6 3AA7 3AA8 3AA9 3AAA 3AAB 3AAC 3AAD 3AAE 3AAG 3AAI 3AAJ 3AAK 3AAL 3AAM 3AAP 3AAQ 3AAR 3AAS 3AAT 3AAU 3AAV 3AAW 3AAX 3AAY 3AAZ 3AB0 3AB1 3AB2 3AB3 3AB4 3AB5 3AB6 3AB7 3AB8 3AB9 3ABA 3ABB 3ABD 3ABE 3ABF 3ABG 3ABH 3ABI 3ABK 3ABL 3ABM 3ABN 3ABO 3ABQ 3ABR 3ABS 3ABT 3ABU 3ABV 3ABW 3ABX 3ABZ 3AC0 3AC1 3AC2 3AC3 3AC4 3AC5 3AC8 3AC9 3ACA 3ACB 3ACC 3ACD 3ACF 3ACG 3ACH 3ACI 3ACJ 3ACK 3ACL 3ACO 3ACP 3ACS 3ACT 3ACW 3ACX 3ACY 3ACZ 3AD4 3AD5 3AD6 3AD7 3AD8 3AD9 3ADA 3ADE 3ADF 3ADG 3ADJ 3ADK 3ADM 3ADO 3ADP 3ADR 3ADS 3ADT 3ADU 3ADV 3ADW 3ADX 3ADY 3ADZ 3AE0 3AE1 3AE2 3AE3 3AE4 3AE5 3AE6 3AE7 3AE8 3AE9 3AEA 3AEB 3AEC 3AED 3AEE 3AEF 3AEG 3AEH 3AEI 3AEJ 3AEK 3AEL 3AEM 3AEN 3AEO 3AEP 3AEQ 3AER 3AES 3AET 3AEU 3AEX 3AEY 3AEZ 3AF0 3AF1 3AF2 3AF3 3AF4 3AF5 3AF7 3AF8 3AF9 3AFB 3AFC 3AFE 3AFF 3AFG 3AFH 3AFI 3AFJ 3AFK 3AFL 3AFM 3AFN 3AFO 3AFP 3AFQ 3AFR 3AFV 3AG0 3AG1 3AG2 3AG3 3AG4 3AG5 3AG6 3AG7 3AG9 3AGA 3AGB 3AGC 3AGD 3AGE 3AGF 3AGG 3AGH 3AGI 3AGK 3AGL 3AGM 3AGN 3AGO 3AGP 3AGQ 3AGR 3AGT 3AGU 3AGW 3AGX 3AGY 3AGZ 3AH1 3AH2 3AH3 3AH4 3AH5 3AH6 3AH7 3AH8 3AH9 3AHA 3AHC 3AHD 3AHE 3AHF 3AHG 3AHH 3AHI 3AHJ 3AHM 3AHN 3AHO 3AHP 3AHQ 3AHR 3AHS 3AHT 3AHV 3AHW 3AHX 3AHY 3AHZ 3AI0 3AI1 3AI2 3AI3 3AI4 3AI5 3AI6 3AI7 3AI8 3AI9 3AIA 3AIB 3AIC 3AID 3AIE 3AIG 3AIH 3AII 3AIK 3AIL 3AIM 3AIN 3AIO 3AIQ 3AIR 3AIS 3AIU 3AIV 3AIW 3AIX 3AIZ 3AJ1 3AJ2 3AJ3 3AJ4 3AJ5 3AJ6 3AJ7 3AJ8 3AJ9 3AJA 3AJB 3AJC 3AJD 3AJE 3AJF 3AJG 3AJH 3AJI 3AJM 3AJN 3AJO 3AJP 3AJQ 3AJR 3AJV 3AJW 3AJX 3AJY 3AJZ 3AK0 3AK1 3AK2 3AK3 3AK4 3AK5 3AK8 3AK9 3AKA 3AKB 3AKC 3AKD 3AKE 3AKF 3AKG 3AKH 3AKI 3AKJ 3AKK 3AKL 3AKM 3AKN 3AKO 3AKP 3AKQ 3AKR 3AKS 3AKT 3AKY 3AL1 3AL2 3AL3 3AL4 3AL5 3AL6 3AL7 3AL8 3AL9 3ALA 3ALB 3ALD 3ALE 3ALF 3ALG 3ALJ 3ALL 3ALM 3ALN 3ALO 3ALP 3ALQ 3ALR 3ALS 3ALT 3ALU 3ALW 3ALX 3ALY 3ALZ 3AM2 3AM3 3AM4 3AM5 3AM6 3AM7 3AM8 3AM9 3AMA 3AMB 3AMC 3AMD 3AME 3AMF 3AMG 3AMH 3AMI 3AMJ 3AMK 3AML 3AMM 3AMN 3AMO 3AMP 3AMQ 3AMR 3AMS 3AMV 3AMY 3AMZ 3AN1 3AN3 3AN4 3ANG 3ANI 3ANJ 3ANK 3ANL 3ANM 3ANN 3ANO 3ANP 3ANQ 3ANR 3ANS 3ANT 3ANU 3ANV 3ANW 3ANX 3ANY 3ANZ 3AO0 3AO1 3AO2 3AO3 3AO4 3AO5 3AO9 3AOA 3AOB 3AOC 3AOD 3AOE 3AOF 3AOG 3AOK 3AON 3AOP 3AOS 3AOT 3AOU 3AOV 3AOW 3AOX 3AP1 3AP2 3AP3 3AP4 3AP5 3AP6 3AP7 3AP9 3APA 3APB 3APC 3APD 3APF 3APG 3APM 3APN 3APO 3APP 3APQ 3APR 3APS 3APT 3APU 3APV 3APW 3APX 3APY 3APZ 3AQ0 3AQ1 3AQ2 3AQ3 3AQ4 3AQ5 3AQ6 3AQ7 3AQ8 3AQ9 3AQA 3AQB 3AQC 3AQD 3AQE 3AQF 3AQG 3AQI 3AQJ 3AQK 3AQL 3AQM 3AQN 3AQO 3AQP 3AQQ 3AQS 3AQT 3AQU 3AQV 3AQX 3AQY 3AQZ 3AR2 3AR3 3AR4 3AR5 3AR6 3AR7 3AR8 3AR9 3ARA 3ARB 3ARD 3ARE 3ARF 3ARG 3ARJ 3ARK 3ARL 3ARN 3ARO 3ARP 3ARQ 3ARR 3ARS 3ART 3ARU 3ARV 3ARW 3ARX 3ARY 3ARZ 3AS0 3AS1 3AS2 3AS3 3AS4 3AS5 3AS8 3ASA 3ASB 3ASD 3ASE 3ASF 3ASG 3ASH 3ASI 3ASJ 3ASK 3ASL 3ASM 3ASN 3ASO 3ASP 3ASQ 3ASR 3ASS 3AST 3ASU 3ASV 3ASW 3ASX 3ASY 3ASZ 3AT0 3AT1 3AT2 3AT3 3AT4 3AT5 3AT6 3AT7 3AT8 3AT9 3ATA 3ATB 3ATD 3ATE 3ATF 3ATG 3ATH 3ATI 3ATJ 3ATK 3ATL 3ATM 3ATN 3ATO 3ATP 3ATQ 3ATR 3ATS 3ATT 3ATU 3ATV 3ATW 3ATY 3ATZ 3AU0 3AU1 3AU2 3AU3 3AU4 3AU5 3AU7 3AU8 3AU9 3AUA 3AUB 3AUC 3AUD 3AUE 3AUF 3AUG 3AUH 3AUI 3AUJ 3AUK 3AUL 3AUM 3AUN 3AUP 3AUQ 3AUR 3AUS 3AUT 3AUU 3AUV 3AUW 3AUX 3AUY 3AUZ 3AV0 3AV3 3AV4 3AV5 3AV6 3AV7 3AV8 3AV9 3AVA 3AVB 3AVC 3AVE 3AVF 3AVG 3AVH 3AVI 3AVJ 3AVK 3AVL 3AVM 3AVN 3AVO 3AVP 3AVQ 3AVR 3AVS 3AVZ 3AW0 3AW1 3AW5 3AW6 3AW7 3AW8 3AW9 3AWD 3AWE 3AWF 3AWG 3AWH 3AWI 3AWJ 3AWK 3AWM 3AWN 3AWO 3AWP 3AWQ 3AWR 3AWS 3AWT 3AWU 3AWV 3AWW 3AWX 3AWY 3AWZ 3AX0 3AX1 3AX2 3AX3 3AX4 3AX5 3AX6 3AX7 3AX8 3AX9 3AXA 3AXB 3AXC 3AXD 3AXE 3AXF 3AXG 3AXH 3AXI 3AXJ 3AXK 3AXL 3AXM 3AXS 3AXT 3AXW 3AXX 3AXY 3AXZ 3AY0 3AY2 3AY3 3AY4 3AY5 3AY6 3AY7 3AY8 3AY9 3AYA 3AYC 3AYD 3AYE 3AYF 3AYG 3AYH 3AYI 3AYJ 3AYL 3AYM 3AYN 3AYQ 3AYR 3AYS 3AYT 3AYU 3AYV 3AYX 3AYY 3AYZ 3AZ1 3AZ2 3AZ3 3AZ4 3AZ5 3AZ6 3AZ7 3AZ8 3AZ9 3AZA 3AZB 3AZC 3AZD 3AZO 3AZP 3AZQ 3AZR 3AZS 3AZT 3AZU 3AZV 3AZW 3AZX 3AZY 3AZZ 3B00 3B01 3B02 3B03 3B04 3B05 3B06 3B07 3B08 3B09 3B0A 3B0B 3B0C 3B0D 3B0F 3B0G 3B0H 3B0I 3B0J 3B0K 3B0L 3B0M 3B0N 3B0O 3B0P 3B0Q 3B0R 3B0S 3B0T 3B0W 3B0X 3B0Y 3B0Z 3B12 3B13 3B18 3B1B 3B1C 3B1D 3B1E 3B1F 3B1J 3B1K 3B1L 3B1M 3B1N 3B1O 3B1P 3B1Q 3B1R 3B1S 3B1T 3B1U 3B1V 3B1W 3B1X 3B1Y 3B1Z 3B20 3B21 3B23 3B24 3B25 3B26 3B27 3B28 3B29 3B2C 3B2D 3B2E 3B2F 3B2G 3B2H 3B2I 3B2J 3B2K 3B2L 3B2M 3B2N 3B2P 3B2Q 3B2R 3B2S 3B2T 3B2U 3B2V 3B2W 3B2X 3B2Y 3B2Z 3B30 3B32 3B33 3B34 3B35 3B36 3B37 3B38 3B3A 3B3B 3B3C 3B3D 3B3F 3B3G 3B3I 3B3J 3B3K 3B3L 3B3M 3B3N 3B3O 3B3P 3B3Q 3B3R 3B3S 3B3T 3B3V 3B3W 3B3X 3B40 3B42 3B43 3B44 3B45 3B46 3B47 3B48 3B49 3B4D 3B4F 3B4M 3B4N 3B4O 3B4P 3B4Q 3B4R 3B4S 3B4T 3B4U 3B4V 3B4W 3B4X 3B4Y 3B50 3B51 3B52 3B53 3B54 3B55 3B56 3B57 3B59 3B5B 3B5D 3B5E 3B5G 3B5H 3B5I 3B5J 3B5K 3B5L 3B5M 3B5N 3B5O 3B5P 3B5Q 3B5R 3B5T 3B5W 3B5X 3B5Y 3B5Z 3B60 3B61 3B62 3B64 3B65 3B66 3B67 3B68 3B69 3B6A 3B6B 3B6C 3B6D 3B6E 3B6H 3B6I 3B6J 3B6K 3B6L 3B6M 3B6N 3B6O 3B6P 3B6Q 3B6R 3B6S 3B6T 3B6U 3B6V 3B6W 3B6X 3B6Y 3B6Z 3B70 3B71 3B72 3B73 3B74 3B75 3B76 3B77 3B78 3B79 3B7A 3B7B 3B7C 3B7D 3B7E 3B7F 3B7G 3B7H 3B7I 3B7J 3B7K 3B7L 3B7M 3B7N 3B7O 3B7P 3B7Q 3B7R 3B7S 3B7T 3B7U 3B7V 3B7W 3B7X 3B7Y 3B7Z 3B80 3B81 3B82 3B83 3B84 3B85 3B86 3B87 3B88 3B89 3B8A 3B8B 3B8C 3B8D 3B8E 3B8F 3B8G 3B8H 3B8I 3B8J 3B8L 3B8M 3B8N 3B8O 3B8P 3B8Q 3B8R 3B8S 3B8T 3B8U 3B8V 3B8W 3B8X 3B8Y 3B8Z 3B90 3B92 3B93 3B94 3B95 3B96 3B97 3B98 3B99 3B9A 3B9B 3B9C 3B9D 3B9E 3B9F 3B9G 3B9H 3B9I 3B9J 3B9K 3B9L 3B9M 3B9N 3B9O 3B9P 3B9Q 3B9R 3B9S 3B9T 3B9U 3B9V 3B9W 3B9X 3B9Y 3B9Z 3BA0 3BA1 3BA2 3BA3 3BA4 3BA5 3BA6 3BA7 3BA8 3BA9 3BAA 3BAB 3BAC 3BAD 3BAE 3BAF 3BAG 3BAH 3BAI 3BAJ 3BAK 3BAL 3BAN 3BAO 3BAP 3BAQ 3BAR 3BAS 3BAT 3BAU 3BAV 3BAW 3BAX 3BAY 3BAZ 3BB0 3BB1 3BB2 3BB3 3BB4 3BB5 3BB6 3BB7 3BB8 3BB9 3BBA 3BBB 3BBC 3BBD 3BBE 3BBF 3BBH 3BBJ 3BBL 3BBP 3BBR 3BBT 3BBW 3BBY 3BBZ 3BC1 3BC2 3BC3 3BC4 3BC5 3BC8 3BC9 3BCA 3BCB 3BCC 3BCD 3BCE 3BCF 3BCG 3BCH 3BCI 3BCJ 3BCK 3BCM 3BCN 3BCO 3BCP 3BCQ 3BCR 3BCS 3BCT 3BCU 3BCV 3BCW 3BCX 3BCY 3BCZ 3BD0 3BD1 3BD2 3BD3 3BD4 3BD5 3BD6 3BD7 3BD8 3BD9 3BDA 3BDB 3BDC 3BDD 3BDE 3BDF 3BDG 3BDH 3BDI 3BDJ 3BDK 3BDL 3BDM 3BDQ 3BDR 3BDU 3BDV 3BDW 3BDX 3BDY 3BDZ 3BE0 3BE1 3BE2 3BE3 3BE4 3BE5 3BE6 3BE7 3BE8 3BE9 3BEA 3BEB 3BEC 3BED 3BEE 3BEF 3BEG 3BEH 3BEI 3BEJ 3BEL 3BEM 3BEN 3BEO 3BEQ 3BER 3BES 3BET 3BEU 3BEV 3BEW 3BEX 3BEY 3BEZ 3BF0 3BF1 3BF2 3BF3 3BF4 3BF5 3BF6 3BF7 3BF8 3BFA 3BFB 3BFC 3BFD 3BFE 3BFF 3BFG 3BFH 3BFI 3BFJ 3BFK 3BFM 3BFN 3BFO 3BFP 3BFQ 3BFR 3BFT 3BFU 3BFV 3BFW 3BFX 3BG0 3BG1 3BG2 3BG3 3BG4 3BG5 3BG6 3BG7 3BG8 3BG9 3BGA 3BGB 3BGC 3BGD 3BGE 3BGF 3BGG 3BGH 3BGI 3BGJ 3BGK 3BGL 3BGM 3BGO 3BGP 3BGQ 3BGR 3BGS 3BGT 3BGU 3BGV 3BGW 3BGX 3BGY 3BGZ 3BH0 3BH1 3BH2 3BH3 3BH4 3BH6 3BH7 3BH8 3BH9 3BHB 3BHD 3BHE 3BHF 3BHG 3BHH 3BHI 3BHJ 3BHK 3BHL 3BHM 3BHN 3BHO 3BHP 3BHQ 3BHR 3BHS 3BHT 3BHU 3BHV 3BHW 3BHX 3BHY 3BI0 3BI1 3BI2 3BI4 3BI5 3BI6 3BI7 3BI9 3BIA 3BIB 3BIC 3BID 3BIF 3BIG 3BIH 3BII 3BIJ 3BIK 3BIL 3BIM 3BIN 3BIO 3BIP 3BIQ 3BIR 3BIS 3BIT 3BIU 3BIV 3BIW 3BIX 3BIY 3BIZ 3BJ1 3BJ2 3BJ3 3BJ4 3BJ5 3BJ6 3BJ7 3BJ8 3BJ9 3BJA 3BJB 3BJC 3BJD 3BJE 3BJF 3BJG 3BJH 3BJI 3BJK 3BJL 3BJM 3BJN 3BJO 3BJP 3BJQ 3BJR 3BJS 3BJT 3BJU 3BJV 3BJW 3BJX 3BJZ 3BK0 3BK1 3BK2 3BK3 3BK5 3BK6 3BK7 3BK8 3BK9 3BKB 3BKC 3BKD 3BKF 3BKH 3BKI 3BKJ 3BKK 3BKL 3BKM 3BKN 3BKP 3BKQ 3BKR 3BKS 3BKT 3BKU 3BKV 3BKW 3BKX 3BKY 3BL0 3BL1 3BL2 3BL3 3BL4 3BL5 3BL6 3BL7 3BL8 3BL9 3BLA 3BLB 3BLC 3BLD 3BLE 3BLF 3BLG 3BLH 3BLI 3BLJ 3BLK 3BLL 3BLM 3BLN 3BLO 3BLP 3BLQ 3BLR 3BLS 3BLT 3BLU 3BLV 3BLW 3BLX 3BLY 3BLZ 3BM1 3BM2 3BM4 3BM5 3BM6 3BM7 3BM8 3BM9 3BMA 3BMB 3BMC 3BMN 3BMO 3BMP 3BMQ 3BMV 3BMW 3BMX 3BMY 3BMZ 3BN0 3BN1 3BN3 3BN4 3BN6 3BN7 3BN8 3BN9 3BNB 3BNC 3BND 3BNE 3BNF 3BNG 3BNH 3BNI 3BNJ 3BNK 3BNM 3BNU 3BNV 3BNW 3BNX 3BNY 3BNZ 3BO5 3BO6 3BO7 3BO8 3BO9 3BOA 3BOB 3BOC 3BOD 3BOE 3BOF 3BOG 3BOH 3BOI 3BOJ 3BOK 3BOL 3BOM 3BON 3BOO 3BOP 3BOQ 3BOR 3BOS 3BOV 3BOW 3BOX 3BP1 3BP2 3BP3 3BP4 3BP5 3BP6 3BP7 3BP8 3BP9 3BPB 3BPC 3BPD 3BPF 3BPJ 3BPK 3BPL 3BPM 3BPN 3BPO 3BPP 3BPQ 3BPR 3BPS 3BPT 3BPU 3BPV 3BPW 3BPX 3BPZ 3BQ3 3BQ4 3BQ5 3BQ6 3BQ7 3BQ8 3BQ9 3BQA 3BQB 3BQC 3BQD 3BQE 3BQF 3BQG 3BQH 3BQI 3BQJ 3BQK 3BQL 3BQM 3BQN 3BQO 3BQP 3BQQ 3BQR 3BQT 3BQU 3BQV 3BQW 3BQX 3BQY 3BQZ 3BR0 3BR1 3BR2 3BR3 3BR5 3BR6 3BR8 3BR9 3BRA 3BRB 3BRC 3BRE 3BRH 3BRI 3BRJ 3BRK 3BRL 3BRM 3BRN 3BRO 3BRP 3BRQ 3BRS 3BRT 3BRU 3BRV 3BRW 3BRX 3BRY 3BRZ 3BS0 3BS2 3BS3 3BS4 3BS5 3BS6 3BS7 3BS8 3BS9 3BSA 3BSC 3BSD 3BSF 3BSG 3BSH 3BSM 3BSQ 3BSS 3BSW 3BSY 3BSZ 3BT0 3BT1 3BT2 3BT3 3BT4 3BT5 3BT6 3BT8 3BT9 3BTA 3BTC 3BTD 3BTE 3BTF 3BTG 3BTH 3BTI 3BTJ 3BTK 3BTL 3BTM 3BTN 3BTO 3BTP 3BTQ 3BTR 3BTS 3BTT 3BTU 3BTV 3BTW 3BU1 3BU2 3BU3 3BU4 3BU5 3BU6 3BU7 3BU8 3BU9 3BUA 3BUB 3BUD 3BUE 3BUF 3BUG 3BUH 3BUI 3BUJ 3BUK 3BUL 3BUM 3BUN 3BUO 3BUP 3BUQ 3BUR 3BUS 3BUT 3BUU 3BUV 3BUW 3BUX 3BUY 3BUZ 3BV0 3BV2 3BV3 3BV4 3BV6 3BV7 3BV8 3BV9 3BVA 3BVB 3BVC 3BVD 3BVE 3BVF 3BVG 3BVH 3BVI 3BVJ 3BVK 3BVL 3BVM 3BVN 3BVO 3BVP 3BVQ 3BVS 3BVT 3BVU 3BVV 3BVW 3BVX 3BVZ 3BW1 3BW2 3BW3 3BW4 3BW6 3BW7 3BW8 3BW9 3BWA 3BWB 3BWC 3BWD 3BWE 3BWF 3BWG 3BWH 3BWI 3BWJ 3BWK 3BWL 3BWM 3BWN 3BWO 3BWQ 3BWR 3BWS 3BWT 3BWU 3BWV 3BWW 3BWX 3BWY 3BWZ 3BX1 3BX4 3BX5 3BX7 3BX8 3BX9 3BXA 3BXB 3BXC 3BXD 3BXE 3BXF 3BXG 3BXH 3BXI 3BXJ 3BXK 3BXL 3BXM 3BXN 3BXO 3BXP 3BXQ 3BXR 3BXS 3BXU 3BXV 3BXW 3BXX 3BXY 3BXZ 3BY0 3BY1 3BY2 3BY4 3BY5 3BY6 3BY7 3BY8 3BY9 3BYA 3BYB 3BYD 3BYI 3BYJ 3BYK 3BYL 3BYM 3BYN 3BYO 3BYP 3BYQ 3BYR 3BYS 3BYT 3BYU 3BYV 3BYW 3BYX 3BYY 3BYZ 3BZ0 3BZ3 3BZ4 3BZ5 3BZ6 3BZ7 3BZ8 3BZ9 3BZA 3BZB 3BZC 3BZD 3BZE 3BZF 3BZG 3BZH 3BZI 3BZJ 3BZK 3BZL 3BZM 3BZN 3BZO 3BZP 3BZQ 3BZR 3BZS 3BZT 3BZU 3BZV 3BZW 3BZX 3BZY 3BZZ 3C00 3C01 3C02 3C03 3C04 3C05 3C06 3C07 3C08 3C09 3C0A 3C0B 3C0C 3C0D 3C0E 3C0F 3C0G 3C0H 3C0I 3C0J 3C0K 3C0L 3C0M 3C0N 3C0O 3C0P 3C0Q 3C0R 3C0S 3C0T 3C0U 3C0V 3C0Y 3C0Z 3C10 3C11 3C12 3C13 3C14 3C15 3C16 3C17 3C18 3C19 3C1A 3C1D 3C1E 3C1F 3C1G 3C1H 3C1I 3C1J 3C1K 3C1L 3C1M 3C1N 3C1O 3C1Q 3C1R 3C1S 3C1T 3C1U 3C1V 3C1X 3C1Y 3C1Z 3C20 3C21 3C22 3C23 3C24 3C26 3C27 3C2A 3C2B 3C2C 3C2E 3C2F 3C2G 3C2H 3C2O 3C2Q 3C2R 3C2S 3C2T 3C2U 3C2V 3C2W 3C2X 3C2Y 3C30 3C31 3C32 3C33 3C34 3C35 3C36 3C37 3C38 3C39 3C3A 3C3B 3C3C 3C3D 3C3E 3C3F 3C3G 3C3H 3C3I 3C3J 3C3K 3C3M 3C3N 3C3O 3C3P 3C3Q 3C3R 3C3S 3C3T 3C3U 3C3V 3C3W 3C3X 3C3Y 3C41 3C43 3C45 3C48 3C49 3C4A 3C4B 3C4C 3C4E 3C4F 3C4H 3C4J 3C4M 3C4N 3C4O 3C4P 3C4Q 3C4R 3C4S 3C4T 3C4U 3C4V 3C4W 3C4X 3C4Y 3C4Z 3C50 3C51 3C52 3C56 3C57 3C59 3C5A 3C5C 3C5E 3C5H 3C5I 3C5J 3C5K 3C5L 3C5M 3C5N 3C5O 3C5P 3C5Q 3C5R 3C5S 3C5T 3C5U 3C5V 3C5W 3C5X 3C5Y 3C5Z 3C60 3C61 3C62 3C63 3C64 3C65 3C66 3C6A 3C6B 3C6C 3C6E 3C6F 3C6G 3C6H 3C6K 3C6L 3C6M 3C6N 3C6O 3C6P 3C6Q 3C6S 3C6T 3C6U 3C6V 3C6W 3C6X 3C6Y 3C6Z 3C70 3C71 3C72 3C73 3C74 3C75 3C76 3C77 3C78 3C79 3C7A 3C7C 3C7D 3C7E 3C7F 3C7G 3C7H 3C7I 3C7J 3C7K 3C7L 3C7M 3C7N 3C7O 3C7P 3C7Q 3C7T 3C7U 3C7V 3C7W 3C7X 3C7Y 3C7Z 3C80 3C81 3C82 3C83 3C84 3C85 3C86 3C87 3C88 3C89 3C8A 3C8B 3C8C 3C8D 3C8E 3C8F 3C8G 3C8H 3C8I 3C8J 3C8K 3C8L 3C8M 3C8N 3C8O 3C8P 3C8Q 3C8R 3C8S 3C8T 3C8U 3C8V 3C8W 3C8X 3C8Y 3C8Z 3C90 3C94 3C95 3C96 3C97 3C98 3C99 3C9A 3C9B 3C9C 3C9D 3C9E 3C9F 3C9G 3C9H 3C9I 3C9J 3C9L 3C9M 3C9N 3C9P 3C9Q 3C9R 3C9S 3C9T 3C9U 3C9W 3C9X 3C9Z 3CA0 3CA1 3CA2 3CA3 3CA4 3CA5 3CA6 3CA7 3CA8 3CA9 3CAA 3CAB 3CAD 3CAE 3CAF 3CAG 3CAH 3CAI 3CAJ 3CAK 3CAL 3CAM 3CAN 3CAO 3CAP 3CAQ 3CAR 3CAS 3CAV 3CAW 3CAX 3CAY 3CAZ 3CB0 3CB2 3CB3 3CB4 3CB5 3CB6 3CB7 3CB8 3CB9 3CBA 3CBC 3CBD 3CBE 3CBF 3CBG 3CBH 3CBI 3CBJ 3CBK 3CBL 3CBM 3CBN 3CBO 3CBP 3CBQ 3CBR 3CBS 3CBT 3CBU 3CBW 3CBX 3CBY 3CBZ 3CC0 3CC1 3CC5 3CC6 3CC8 3CC9 3CCB 3CCC 3CCD 3CCF 3CCG 3CCH 3CCK 3CCN 3CCP 3CCT 3CCW 3CCX 3CCY 3CCZ 3CD0 3CD1 3CD2 3CD3 3CD4 3CD5 3CD7 3CD8 3CD9 3CDA 3CDB 3CDC 3CDD 3CDE 3CDF 3CDG 3CDH 3CDI 3CDJ 3CDK 3CDL 3CDN 3CDO 3CDP 3CDQ 3CDR 3CDS 3CDT 3CDU 3CDV 3CDW 3CDX 3CDY 3CDZ 3CE0 3CE1 3CE2 3CE3 3CE4 3CE6 3CE7 3CE8 3CE9 3CEA 3CEB 3CEC 3CED 3CEG 3CEH 3CEI 3CEJ 3CEK 3CEL 3CEM 3CEN 3CEP 3CEQ 3CER 3CES 3CET 3CEU 3CEV 3CEW 3CEX 3CEY 3CEZ 3CF0 3CF1 3CF2 3CF3 3CF4 3CF6 3CF8 3CF9 3CFA 3CFB 3CFC 3CFD 3CFE 3CFF 3CFH 3CFI 3CFJ 3CFK 3CFL 3CFM 3CFN 3CFO 3CFQ 3CFS 3CFT 3CFU 3CFV 3CFW 3CFX 3CFY 3CFZ 3CG0 3CG1 3CG3 3CG4 3CG5 3CG6 3CG7 3CG8 3CG9 3CGA 3CGB 3CGC 3CGD 3CGE 3CGF 3CGG 3CGH 3CGI 3CGL 3CGM 3CGN 3CGO 3CGT 3CGU 3CGW 3CGX 3CGY 3CGZ 3CH0 3CH1 3CH2 3CH3 3CH4 3CH5 3CH6 3CH7 3CH8 3CH9 3CHB 3CHC 3CHD 3CHE 3CHF 3CHG 3CHH 3CHI 3CHJ 3CHK 3CHL 3CHM 3CHO 3CHP 3CHQ 3CHR 3CHS 3CHT 3CHU 3CHV 3CHW 3CHX 3CHY 3CI0 3CI1 3CI3 3CI4 3CI5 3CI6 3CI7 3CI8 3CI9 3CIA 3CIB 3CIC 3CID 3CIF 3CIG 3CIH 3CII 3CIJ 3CIK 3CIM 3CIN 3CIO 3CIP 3CIQ 3CIR 3CIS 3CIT 3CIU 3CIV 3CIW 3CIX 3CIZ 3CJ0 3CJ1 3CJ2 3CJ3 3CJ4 3CJ5 3CJ7 3CJ8 3CJ9 3CJA 3CJB 3CJC 3CJD 3CJE 3CJF 3CJG 3CJH 3CJI 3CJJ 3CJK 3CJL 3CJM 3CJN 3CJO 3CJP 3CJQ 3CJR 3CJS 3CJT 3CJW 3CJX 3CJY 3CK0 3CK1 3CK2 3CK4 3CK5 3CK6 3CK7 3CK8 3CK9 3CKA 3CKB 3CKC 3CKD 3CKE 3CKF 3CKG 3CKH 3CKI 3CKJ 3CKK 3CKL 3CKM 3CKN 3CKO 3CKP 3CKQ 3CKR 3CKS 3CKT 3CKU 3CKV 3CKW 3CKX 3CKY 3CKZ 3CL0 3CL1 3CL2 3CL3 3CL4 3CL5 3CL6 3CL7 3CL8 3CL9 3CLA 3CLB 3CLD 3CLE 3CLF 3CLH 3CLI 3CLJ 3CLK 3CLL 3CLM 3CLN 3CLO 3CLP 3CLQ 3CLR 3CLS 3CLT 3CLU 3CLV 3CLW 3CLX 3CLY 3CM0 3CM1 3CM2 3CM3 3CM4 3CM5 3CM6 3CM7 3CM8 3CMB 3CMC 3CMD 3CMF 3CMG 3CMI 3CMJ 3CML 3CMM 3CMN 3CMO 3CMP 3CMQ 3CMR 3CMS 3CMV 3CMZ 3CN0 3CN1 3CN2 3CN3 3CN4 3CN5 3CN6 3CN7 3CN8 3CN9 3CNA 3CNB 3CNC 3CND 3CNE 3CNG 3CNH 3CNI 3CNJ 3CNK 3CNL 3CNM 3CNN 3CNO 3CNP 3CNQ 3CNR 3CNS 3CNT 3CNU 3CNV 3CNW 3CNX 3CNY 3CNZ 3CO0 3CO1 3CO2 3CO4 3CO5 3CO8 3CO9 3COB 3COC 3COD 3COG 3COH 3COI 3COJ 3COK 3COL 3COM 3CON 3COO 3COP 3COR 3COS 3COT 3COU 3COV 3COW 3COX 3COY 3COZ 3CP0 3CP1 3CP2 3CP3 3CP4 3CP5 3CP6 3CP7 3CP8 3CP9 3CPA 3CPB 3CPC 3CPE 3CPF 3CPG 3CPH 3CPI 3CPJ 3CPK 3CPL 3CPM 3CPO 3CPP 3CPQ 3CPR 3CPS 3CPT 3CPU 3CPX 3CPZ 3CQ0 3CQ1 3CQ2 3CQ3 3CQ4 3CQ5 3CQ6 3CQ9 3CQA 3CQB 3CQC 3CQD 3CQE 3CQF 3CQG 3CQH 3CQI 3CQJ 3CQK 3CQL 3CQN 3CQO 3CQP 3CQQ 3CQR 3CQT 3CQU 3CQV 3CQW 3CQX 3CQY 3CQZ 3CR0 3CR2 3CR3 3CR4 3CR5 3CR6 3CR7 3CR8 3CR9 3CRA 3CRB 3CRC 3CRG 3CRH 3CRI 3CRJ 3CRK 3CRL 3CRM 3CRN 3CRP 3CRQ 3CRR 3CRT 3CRU 3CRV 3CRW 3CRY 3CRZ 3CS0 3CS1 3CS2 3CS3 3CS4 3CS5 3CS6 3CS7 3CS8 3CS9 3CSB 3CSC 3CSD 3CSE 3CSF 3CSG 3CSH 3CSI 3CSJ 3CSK 3CSL 3CSM 3CSN 3CSO 3CSP 3CSQ 3CSR 3CSS 3CST 3CSU 3CSV 3CSW 3CSX 3CSY 3CSZ 3CT0 3CT1 3CT2 3CT4 3CT5 3CT6 3CT7 3CT8 3CT9 3CTA 3CTB 3CTD 3CTF 3CTG 3CTH 3CTJ 3CTK 3CTL 3CTM 3CTO 3CTP 3CTQ 3CTR 3CTS 3CTT 3CTV 3CTW 3CTY 3CTZ 3CU0 3CU1 3CU2 3CU3 3CU4 3CU5 3CU7 3CU8 3CU9 3CUC 3CUE 3CUF 3CUG 3CUH 3CUI 3CUJ 3CUK 3CUO 3CUP 3CUQ 3CUR 3CUS 3CUT 3CUU 3CUV 3CUW 3CUX 3CUY 3CUZ 3CV0 3CV1 3CV2 3CV3 3CV5 3CV6 3CV7 3CV8 3CV9 3CVA 3CVB 3CVC 3CVD 3CVE 3CVF 3CVG 3CVH 3CVI 3CVJ 3CVK 3CVL 3CVM 3CVN 3CVO 3CVP 3CVQ 3CVR 3CVZ 3CW0 3CW2 3CW3 3CW4 3CW8 3CW9 3CWB 3CWC 3CWD 3CWE 3CWF 3CWG 3CWI 3CWJ 3CWK 3CWL 3CWM 3CWN 3CWO 3CWQ 3CWR 3CWV 3CWW 3CWX 3CWY 3CWZ 3CX2 3CX3 3CX4 3CX5 3CX6 3CX7 3CX8 3CX9 3CXA 3CXB 3CXD 3CXF 3CXG 3CXH 3CXI 3CXJ 3CXK 3CXL 3CXM 3CXN 3CXO 3CXP 3CXQ 3CXR 3CXS 3CXU 3CXV 3CXW 3CXX 3CXY 3CXZ 3CY0 3CY1 3CY2 3CY3 3CY4 3CY5 3CY6 3CYE 3CYF 3CYG 3CYH 3CYI 3CYJ 3CYL 3CYM 3CYN 3CYO 3CYP 3CYQ 3CYR 3CYT 3CYU 3CYV 3CYW 3CYX 3CYY 3CYZ 3CZ0 3CZ1 3CZ2 3CZ4 3CZ5 3CZ6 3CZ7 3CZ8 3CZ9 3CZA 3CZB 3CZC 3CZD 3CZE 3CZF 3CZG 3CZH 3CZJ 3CZK 3CZL 3CZM 3CZN 3CZO 3CZP 3CZQ 3CZR 3CZS 3CZT 3CZU 3CZV 3CZX 3CZY 3CZZ 3D00 3D01 3D02 3D03 3D04 3D05 3D06 3D07 3D08 3D09 3D0B 3D0C 3D0E 3D0F 3D0G 3D0H 3D0I 3D0J 3D0K 3D0L 3D0N 3D0O 3D0Q 3D0R 3D0S 3D0T 3D0V 3D0W 3D0Y 3D0Z 3D10 3D11 3D12 3D14 3D15 3D17 3D18 3D19 3D1A 3D1B 3D1C 3D1D 3D1E 3D1F 3D1G 3D1H 3D1I 3D1J 3D1K 3D1L 3D1M 3D1O 3D1P 3D1Q 3D1R 3D1T 3D1V 3D1X 3D1Y 3D1Z 3D20 3D21 3D22 3D23 3D24 3D25 3D26 3D27 3D28 3D29 3D2A 3D2B 3D2C 3D2D 3D2E 3D2F 3D2H 3D2I 3D2J 3D2K 3D2L 3D2M 3D2N 3D2O 3D2P 3D2Q 3D2R 3D2T 3D2U 3D2Y 3D2Z 3D30 3D31 3D32 3D33 3D34 3D36 3D37 3D38 3D39 3D3A 3D3B 3D3C 3D3D 3D3E 3D3F 3D3H 3D3I 3D3J 3D3K 3D3L 3D3M 3D3N 3D3O 3D3P 3D3Q 3D3R 3D3S 3D3T 3D3U 3D3V 3D3W 3D3X 3D3Y 3D3Z 3D40 3D41 3D42 3D43 3D44 3D45 3D46 3D47 3D48 3D49 3D4A 3D4B 3D4C 3D4D 3D4E 3D4F 3D4G 3D4I 3D4J 3D4K 3D4L 3D4M 3D4N 3D4O 3D4P 3D4Q 3D4R 3D4S 3D4T 3D4U 3D4W 3D4X 3D4Y 3D4Z 3D50 3D51 3D52 3D53 3D54 3D55 3D57 3D59 3D5E 3D5F 3D5G 3D5H 3D5I 3D5J 3D5K 3D5L 3D5M 3D5N 3D5O 3D5P 3D5Q 3D5R 3D5S 3D5T 3D5U 3D5V 3D5W 3D5X 3D5Y 3D5Z 3D60 3D61 3D62 3D63 3D64 3D65 3D66 3D67 3D68 3D69 3D6A 3D6B 3D6C 3D6D 3D6E 3D6F 3D6G 3D6H 3D6I 3D6J 3D6K 3D6L 3D6M 3D6N 3D6O 3D6P 3D6Q 3D6R 3D6S 3D6T 3D6U 3D6V 3D6W 3D6X 3D72 3D73 3D74 3D75 3D76 3D77 3D78 3D79 3D7A 3D7B 3D7C 3D7D 3D7E 3D7F 3D7G 3D7H 3D7I 3D7J 3D7K 3D7L 3D7M 3D7N 3D7O 3D7P 3D7Q 3D7R 3D7S 3D7T 3D7U 3D7V 3D7W 3D7Z 3D80 3D81 3D82 3D83 3D84 3D85 3D87 3D89 3D8A 3D8B 3D8C 3D8D 3D8E 3D8F 3D8G 3D8H 3D8K 3D8L 3D8M 3D8N 3D8P 3D8R 3D8S 3D8T 3D8U 3D8V 3D8W 3D8X 3D8Y 3D8Z 3D90 3D91 3D92 3D93 3D94 3D95 3D96 3D97 3D98 3D9A 3D9B 3D9C 3D9D 3D9E 3D9F 3D9G 3D9H 3D9I 3D9J 3D9K 3D9L 3D9M 3D9N 3D9O 3D9P 3D9Q 3D9R 3D9S 3D9T 3D9U 3D9V 3D9W 3D9X 3D9Y 3D9Z 3DA0 3DA1 3DA2 3DA3 3DA4 3DA5 3DA6 3DA7 3DA8 3DA9 3DAA 3DAB 3DAC 3DAD 3DAE 3DAF 3DAG 3DAH 3DAI 3DAJ 3DAK 3DAL 3DAM 3DAN 3DAO 3DAP 3DAQ 3DAR 3DAS 3DAT 3DAU 3DAV 3DAW 3DAX 3DAY 3DAZ 3DB0 3DB1 3DB2 3DB3 3DB4 3DB5 3DB6 3DB7 3DB8 3DB9 3DBA 3DBC 3DBD 3DBE 3DBF 3DBG 3DBH 3DBI 3DBJ 3DBK 3DBL 3DBM 3DBN 3DBO 3DBP 3DBQ 3DBR 3DBS 3DBU 3DBV 3DBX 3DBY 3DBZ 3DC0 3DC1 3DC2 3DC3 3DC4 3DC5 3DC6 3DC7 3DC8 3DC9 3DCA 3DCB 3DCC 3DCD 3DCF 3DCG 3DCI 3DCJ 3DCK 3DCL 3DCM 3DCN 3DCP 3DCQ 3DCR 3DCS 3DCT 3DCU 3DCV 3DCW 3DCX 3DCY 3DCZ 3DD0 3DD1 3DD3 3DD4 3DD5 3DD6 3DD7 3DD8 3DD9 3DDA 3DDB 3DDC 3DDD 3DDE 3DDF 3DDG 3DDH 3DDI 3DDJ 3DDK 3DDL 3DDM 3DDN 3DDO 3DDP 3DDQ 3DDR 3DDS 3DDT 3DDU 3DDV 3DDW 3DDY 3DDZ 3DE0 3DE1 3DE2 3DE3 3DE4 3DE5 3DE6 3DE7 3DE8 3DE9 3DEA 3DEB 3DEC 3DED 3DEE 3DEF 3DEH 3DEI 3DEJ 3DEK 3DEL 3DEM 3DEN 3DEO 3DEP 3DEQ 3DER 3DES 3DET 3DEU 3DEV 3DEW 3DEX 3DEY 3DEZ 3DF0 3DF6 3DF7 3DF8 3DF9 3DFA 3DFC 3DFE 3DFF 3DFG 3DFH 3DFI 3DFJ 3DFK 3DFL 3DFM 3DFN 3DFO 3DFP 3DFQ 3DFR 3DFS 3DFT 3DFU 3DFY 3DFZ 3DG1 3DG3 3DG6 3DG7 3DG8 3DG9 3DGA 3DGB 3DGC 3DGD 3DGE 3DGF 3DGG 3DGH 3DGI 3DGJ 3DGK 3DGL 3DGM 3DGN 3DGO 3DGP 3DGQ 3DGR 3DGS 3DGT 3DGV 3DGY 3DGZ 3DH0 3DH1 3DH2 3DH4 3DH5 3DH6 3DH7 3DH8 3DH9 3DHA 3DHB 3DHC 3DHD 3DHE 3DHF 3DHG 3DHH 3DHI 3DHJ 3DHK 3DHM 3DHN 3DHO 3DHP 3DHQ 3DHR 3DHT 3DHU 3DHV 3DHW 3DHX 3DHY 3DHZ 3DI0 3DI1 3DI2 3DI3 3DI4 3DI5 3DI6 3DI7 3DI8 3DI9 3DIB 3DIC 3DID 3DIE 3DIF 3DIH 3DIN 3DIP 3DIT 3DIV 3DIW 3DJ1 3DJ3 3DJ4 3DJ5 3DJ6 3DJ7 3DJ8 3DJ9 3DJA 3DJB 3DJC 3DJD 3DJE 3DJF 3DJG 3DJH 3DJI 3DJJ 3DJK 3DJL 3DJM 3DJN 3DJO 3DJP 3DJQ 3DJR 3DJS 3DJT 3DJU 3DJV 3DJW 3DJX 3DJY 3DJZ 3DK0 3DK1 3DK2 3DK3 3DK4 3DK5 3DK6 3DK7 3DK8 3DK9 3DKA 3DKB 3DKC 3DKD 3DKE 3DKF 3DKG 3DKH 3DKI 3DKJ 3DKK 3DKL 3DKM 3DKO 3DKP 3DKQ 3DKR 3DKS 3DKT 3DKU 3DKV 3DKW 3DKX 3DKY 3DKZ 3DL0 3DL1 3DL2 3DL3 3DL4 3DL5 3DL6 3DL7 3DL8 3DL9 3DLA 3DLC 3DLD 3DLE 3DLG 3DLI 3DLJ 3DLK 3DLM 3DLN 3DLO 3DLP 3DLQ 3DLR 3DLS 3DLT 3DLU 3DLV 3DLW 3DLX 3DLZ 3DM0 3DM1 3DM2 3DM3 3DM5 3DM6 3DM7 3DM8 3DM9 3DMB 3DMC 3DMD 3DME 3DMF 3DMG 3DMH 3DMI 3DMJ 3DMK 3DML 3DMM 3DMN 3DMO 3DMP 3DMQ 3DMR 3DMS 3DMT 3DMU 3DMV 3DMW 3DMX 3DMY 3DMZ 3DN0 3DN1 3DN2 3DN3 3DN4 3DN5 3DN6 3DN7 3DN8 3DN9 3DNA 3DNC 3DND 3DNE 3DNF 3DNG 3DNH 3DNI 3DNJ 3DNK 3DNM 3DNP 3DNS 3DNT 3DNU 3DNX 3DNZ 3DO0 3DO1 3DO2 3DO3 3DO4 3DO5 3DO6 3DO8 3DO9 3DOA 3DOB 3DOC 3DOD 3DOE 3DOF 3DOG 3DOH 3DOI 3DOJ 3DOK 3DOL 3DOM 3DON 3DOO 3DOP 3DOR 3DOS 3DOU 3DOW 3DOX 3DOY 3DOZ 3DP0 3DP1 3DP2 3DP3 3DP4 3DP5 3DP6 3DP7 3DP8 3DP9 3DPA 3DPB 3DPC 3DPD 3DPE 3DPF 3DPH 3DPI 3DPJ 3DPK 3DPL 3DPM 3DPN 3DPO 3DPP 3DPQ 3DPR 3DPS 3DPT 3DPU 3DPW 3DPX 3DPY 3DPZ 3DQ0 3DQ1 3DQ2 3DQ3 3DQ4 3DQ5 3DQ6 3DQ7 3DQ8 3DQ9 3DQA 3DQB 3DQC 3DQD 3DQE 3DQF 3DQG 3DQH 3DQI 3DQJ 3DQK 3DQL 3DQM 3DQN 3DQO 3DQP 3DQQ 3DQR 3DQS 3DQT 3DQU 3DQV 3DQW 3DQX 3DQY 3DQZ 3DR0 3DR1 3DR2 3DR3 3DR4 3DR5 3DR6 3DR7 3DR8 3DR9 3DRA 3DRB 3DRC 3DRD 3DRE 3DRF 3DRG 3DRH 3DRI 3DRJ 3DRK 3DRM 3DRN 3DRO 3DRP 3DRQ 3DRR 3DRS 3DRT 3DRU 3DRW 3DRX 3DRY 3DRZ 3DS0 3DS1 3DS2 3DS3 3DS4 3DS5 3DS6 3DS8 3DS9 3DSB 3DSE 3DSF 3DSG 3DSH 3DSI 3DSJ 3DSK 3DSL 3DSM 3DSN 3DSO 3DSP 3DSQ 3DSR 3DSS 3DST 3DSU 3DSV 3DSW 3DSX 3DSY 3DSZ 3DT0 3DT1 3DT2 3DT3 3DT4 3DT5 3DT6 3DT7 3DT8 3DT9 3DTA 3DTB 3DTC 3DTD 3DTE 3DTF 3DTG 3DTI 3DTJ 3DTK 3DTM 3DTN 3DTO 3DTQ 3DTR 3DTS 3DTT 3DTU 3DTV 3DTW 3DTX 3DTY 3DTZ 3DU0 3DU1 3DU2 3DU3 3DU4 3DU5 3DU6 3DU7 3DU8 3DUE 3DUF 3DUG 3DUH 3DUI 3DUK 3DUL 3DUP 3DUQ 3DUR 3DUS 3DUT 3DUU 3DUV 3DUW 3DUX 3DUY 3DUZ 3DV0 3DV1 3DV2 3DV3 3DV4 3DV5 3DV6 3DV7 3DV8 3DV9 3DVA 3DVB 3DVC 3DVD 3DVE 3DVF 3DVG 3DVH 3DVI 3DVJ 3DVK 3DVL 3DVM 3DVN 3DVP 3DVQ 3DVR 3DVS 3DVT 3DVU 3DVW 3DVX 3DW0 3DW1 3DW3 3DW8 3DWA 3DWB 3DWC 3DWD 3DWE 3DWF 3DWG 3DWH 3DWI 3DWJ 3DWK 3DWL 3DWM 3DWN 3DWO 3DWP 3DWQ 3DWR 3DWS 3DWT 3DWV 3DWY 3DWZ 3DX0 3DX1 3DX2 3DX3 3DX4 3DX5 3DX6 3DX7 3DX8 3DX9 3DXA 3DXB 3DXC 3DXD 3DXE 3DXF 3DXG 3DXH 3DXI 3DXJ 3DXK 3DXL 3DXM 3DXN 3DXO 3DXP 3DXQ 3DXR 3DXS 3DXT 3DXU 3DXV 3DXW 3DXX 3DXY 3DXZ 3DY0 3DY3 3DY4 3DY5 3DY6 3DY7 3DY8 3DY9 3DYA 3DYB 3DYC 3DYD 3DYE 3DYF 3DYG 3DYH 3DYI 3DYJ 3DYL 3DYM 3DYN 3DYO 3DYP 3DYQ 3DYR 3DYS 3DYT 3DYU 3DYV 3DZ1 3DZ2 3DZ3 3DZ4 3DZ5 3DZ6 3DZ7 3DZ8 3DZA 3DZB 3DZC 3DZD 3DZE 3DZF 3DZG 3DZH 3DZI 3DZJ 3DZK 3DZL 3DZM 3DZN 3DZO 3DZP 3DZQ 3DZR 3DZT 3DZV 3DZW 3DZX 3DZZ 3E01 3E02 3E03 3E04 3E05 3E07 3E08 3E0A 3E0B 3E0C 3E0E 3E0F 3E0G 3E0H 3E0I 3E0J 3E0K 3E0L 3E0M 3E0N 3E0O 3E0P 3E0Q 3E0R 3E0S 3E0U 3E0V 3E0W 3E0X 3E0Y 3E0Z 3E10 3E11 3E12 3E13 3E15 3E16 3E17 3E18 3E19 3E1E 3E1F 3E1G 3E1H 3E1I 3E1J 3E1K 3E1L 3E1M 3E1N 3E1O 3E1P 3E1Q 3E1R 3E1S 3E1T 3E1U 3E1V 3E1W 3E1X 3E1Y 3E1Z 3E20 3E21 3E22 3E23 3E24 3E25 3E26 3E27 3E28 3E29 3E2A 3E2B 3E2C 3E2D 3E2F 3E2H 3E2I 3E2J 3E2K 3E2L 3E2M 3E2N 3E2O 3E2P 3E2Q 3E2R 3E2S 3E2T 3E2U 3E2V 3E2W 3E2X 3E2Y 3E2Z 3E30 3E31 3E32 3E33 3E34 3E35 3E37 3E38 3E39 3E3A 3E3B 3E3C 3E3D 3E3E 3E3F 3E3G 3E3H 3E3I 3E3K 3E3L 3E3M 3E3N 3E3O 3E3P 3E3Q 3E3R 3E3S 3E3T 3E3U 3E3V 3E3X 3E3Z 3E46 3E47 3E48 3E49 3E4A 3E4B 3E4C 3E4D 3E4E 3E4F 3E4G 3E4H 3E4N 3E4O 3E4P 3E4Q 3E4R 3E4U 3E4V 3E4W 3E4Y 3E4Z 3E50 3E51 3E53 3E55 3E56 3E57 3E58 3E59 3E5A 3E5B 3E5D 3E5H 3E5I 3E5J 3E5K 3E5L 3E5M 3E5N 3E5O 3E5P 3E5Q 3E5R 3E5S 3E5T 3E5U 3E5V 3E5W 3E5X 3E5Y 3E5Z 3E60 3E61 3E62 3E63 3E64 3E65 3E66 3E67 3E68 3E6A 3E6B 3E6D 3E6E 3E6F 3E6G 3E6H 3E6I 3E6J 3E6K 3E6L 3E6M 3E6N 3E6O 3E6P 3E6Q 3E6R 3E6S 3E6T 3E6U 3E6V 3E6Y 3E6Z 3E70 3E73 3E74 3E76 3E77 3E78 3E79 3E7A 3E7B 3E7C 3E7D 3E7F 3E7G 3E7H 3E7I 3E7J 3E7K 3E7L 3E7M 3E7N 3E7O 3E7P 3E7Q 3E7R 3E7S 3E7T 3E7U 3E7V 3E7W 3E7X 3E7Y 3E7Z 3E80 3E81 3E82 3E83 3E84 3E85 3E86 3E87 3E88 3E89 3E8B 3E8C 3E8D 3E8E 3E8F 3E8G 3E8H 3E8J 3E8K 3E8L 3E8M 3E8N 3E8O 3E8P 3E8Q 3E8R 3E8S 3E8T 3E8U 3E8V 3E8W 3E8X 3E8Y 3E8Z 3E90 3E91 3E92 3E93 3E94 3E95 3E96 3E97 3E98 3E99 3E9A 3E9B 3E9C 3E9D 3E9E 3E9F 3E9G 3E9H 3E9I 3E9J 3E9K 3E9L 3E9M 3E9N 3E9O 3E9P 3E9Q 3E9R 3E9S 3E9T 3E9U 3E9V 3E9X 3E9Y 3E9Z 3EA0 3EA1 3EA2 3EA3 3EA4 3EA5 3EA6 3EA7 3EA8 3EA9 3EAA 3EAB 3EAC 3EAD 3EAE 3EAF 3EAG 3EAH 3EAI 3EAJ 3EAK 3EAM 3EAN 3EAO 3EAP 3EAQ 3EAR 3EAS 3EAT 3EAU 3EAW 3EAX 3EAY 3EAZ 3EB0 3EB1 3EB2 3EB3 3EB4 3EB5 3EB6 3EB7 3EB8 3EB9 3EBA 3EBB 3EBD 3EBE 3EBF 3EBG 3EBH 3EBI 3EBJ 3EBK 3EBL 3EBM 3EBN 3EBO 3EBP 3EBQ 3EBR 3EBS 3EBT 3EBU 3EBV 3EBW 3EBX 3EBY 3EBZ 3EC0 3EC1 3EC2 3EC3 3EC4 3EC5 3EC6 3EC7 3EC8 3EC9 3ECA 3ECB 3ECC 3ECD 3ECF 3ECG 3ECH 3ECI 3ECJ 3ECK 3ECL 3ECM 3ECN 3ECO 3ECQ 3ECR 3ECS 3ECT 3ECU 3ECV 3ECW 3ECX 3ECY 3ECZ 3ED0 3ED1 3ED3 3ED4 3ED5 3ED7 3ED8 3ED9 3EDA 3EDB 3EDC 3EDD 3EDE 3EDF 3EDG 3EDH 3EDI 3EDJ 3EDK 3EDM 3EDN 3EDO 3EDP 3EDQ 3EDR 3EDT 3EDU 3EDV 3EDW 3EDX 3EDY 3EDZ 3EE0 3EE1 3EE2 3EE3 3EE4 3EE5 3EE6 3EE7 3EE8 3EE9 3EEA 3EEB 3EEC 3EED 3EEE 3EEF 3EEG 3EEH 3EEI 3EEJ 3EEK 3EEL 3EEM 3EEQ 3EER 3EES 3EET 3EEU 3EEV 3EEX 3EEY 3EEZ 3EF0 3EF1 3EF2 3EF3 3EF4 3EF5 3EF6 3EF7 3EF8 3EF9 3EFA 3EFB 3EFC 3EFD 3EFE 3EFF 3EFG 3EFH 3EFI 3EFJ 3EFK 3EFL 3EFM 3EFO 3EFP 3EFQ 3EFR 3EFS 3EFT 3EFU 3EFV 3EFW 3EFX 3EFY 3EFZ 3EG0 3EG1 3EG2 3EG3 3EG4 3EG5 3EG6 3EG9 3EGA 3EGB 3EGC 3EGD 3EGE 3EGG 3EGH 3EGI 3EGJ 3EGK 3EGL 3EGM 3EGN 3EGO 3EGQ 3EGR 3EGS 3EGT 3EGU 3EGV 3EGW 3EGX 3EGY 3EH0 3EH1 3EH2 3EH3 3EH4 3EH5 3EH7 3EH9 3EHA 3EHB 3EHC 3EHD 3EHE 3EHF 3EHG 3EHH 3EHI 3EHJ 3EHK 3EHM 3EHN 3EHQ 3EHR 3EHS 3EHT 3EHU 3EHV 3EHW 3EHX 3EHY 3EHZ 3EI0 3EI3 3EI4 3EI5 3EI6 3EI7 3EI8 3EI9 3EIA 3EIB 3EIC 3EID 3EIE 3EIF 3EIG 3EIH 3EII 3EIJ 3EIK 3EIM 3EIN 3EIO 3EIP 3EIQ 3EIR 3EIS 3EIT 3EIU 3EIV 3EIW 3EIX 3EIY 3EIZ 3EJ0 3EJ1 3EJ2 3EJ3 3EJ5 3EJ6 3EJ7 3EJ8 3EJ9 3EJA 3EJB 3EJC 3EJD 3EJE 3EJF 3EJG 3EJH 3EJI 3EJJ 3EJK 3EJL 3EJM 3EJN 3EJO 3EJP 3EJQ 3EJR 3EJS 3EJT 3EJU 3EJV 3EJW 3EJX 3EJY 3EJZ 3EK1 3EK2 3EK3 3EK4 3EK5 3EK6 3EK7 3EK8 3EK9 3EKA 3EKB 3EKC 3EKD 3EKE 3EKF 3EKG 3EKH 3EKI 3EKJ 3EKK 3EKL 3EKM 3EKN 3EKO 3EKP 3EKQ 3EKR 3EKS 3EKT 3EKU 3EKV 3EKW 3EKX 3EKY 3EKZ 3EL0 3EL1 3EL2 3EL3 3EL4 3EL5 3EL6 3EL7 3EL8 3EL9 3ELA 3ELB 3ELC 3ELD 3ELE 3ELF 3ELG 3ELH 3ELI 3ELJ 3ELK 3ELL 3ELM 3ELN 3ELO 3ELP 3ELQ 3ELS 3ELU 3ELV 3ELW 3ELX 3ELY 3ELZ 3EM0 3EM1 3EM3 3EM4 3EM6 3EMB 3EMC 3EMD 3EMF 3EMG 3EMH 3EMI 3EMJ 3EMK 3EML 3EMM 3EMN 3EMO 3EMP 3EMQ 3EMR 3EMS 3EMT 3EMU 3EMV 3EMW 3EMX 3EMY 3EMZ 3EN0 3EN1 3EN2 3EN3 3EN4 3EN5 3EN6 3EN7 3EN8 3EN9 3ENA 3ENB 3ENC 3ENE 3ENG 3ENH 3ENI 3ENJ 3ENK 3ENL 3ENM 3ENN 3ENO 3ENP 3ENQ 3ENR 3ENS 3ENT 3ENU 3ENV 3ENW 3ENZ 3EO0 3EO1 3EO2 3EO3 3EO4 3EO5 3EO6 3EO7 3EO8 3EO9 3EOA 3EOB 3EOC 3EOD 3EOE 3EOF 3EOI 3EOJ 3EOK 3EOL 3EOM 3EON 3EOO 3EOP 3EOQ 3EOR 3EOS 3EOT 3EOU 3EOV 3EOX 3EOY 3EOZ 3EP0 3EP1 3EP3 3EP4 3EP5 3EP6 3EP7 3EP8 3EP9 3EPA 3EPB 3EPE 3EPM 3EPN 3EPO 3EPP 3EPR 3EPS 3EPT 3EPU 3EPV 3EPW 3EPX 3EPY 3EPZ 3EQ0 3EQ1 3EQ2 3EQ5 3EQ6 3EQ7 3EQ8 3EQ9 3EQA 3EQB 3EQC 3EQD 3EQE 3EQF 3EQG 3EQH 3EQI 3EQL 3EQM 3EQN 3EQO 3EQP 3EQQ 3EQR 3EQS 3EQU 3EQV 3EQX 3EQY 3EQZ 3ER0 3ER3 3ER5 3ER6 3ER7 3ERA 3ERB 3ERD 3ERF 3ERG 3ERH 3ERI 3ERJ 3ERK 3ERM 3ERN 3ERO 3ERP 3ERQ 3ERR 3ERS 3ERT 3ERV 3ERW 3ERX 3ERY 3ERZ 3ES1 3ES2 3ES3 3ES4 3ES5 3ES6 3ES7 3ES8 3ES9 3ESA 3ESB 3ESC 3ESD 3ESF 3ESG 3ESH 3ESI 3ESJ 3ESK 3ESL 3ESM 3ESN 3ESO 3ESP 3ESQ 3ESR 3ESS 3EST 3ESU 3ESV 3ESW 3ESX 3ESY 3ESZ 3ET0 3ET1 3ET2 3ET3 3ET4 3ET5 3ET6 3ET7 3ET9 3ETA 3ETB 3ETC 3ETD 3ETE 3ETF 3ETG 3ETH 3ETI 3ETJ 3ETL 3ETM 3ETN 3ETO 3ETP 3ETQ 3ETR 3ETS 3ETT 3ETU 3ETV 3ETW 3ETX 3ETY 3ETZ 3EU0 3EU1 3EU3 3EU4 3EU5 3EU7 3EU8 3EU9 3EUA 3EUB 3EUC 3EUD 3EUE 3EUF 3EUG 3EUH 3EUJ 3EUK 3EUL 3EUN 3EUO 3EUP 3EUQ 3EUR 3EUS 3EUT 3EUU 3EUV 3EUW 3EUX 3EUY 3EUZ 3EV0 3EV1 3EV2 3EV3 3EV4 3EV5 3EV6 3EVA 3EVB 3EVC 3EVD 3EVE 3EVF 3EVG 3EVI 3EVJ 3EVK 3EVM 3EVN 3EVO 3EVP 3EVQ 3EVR 3EVS 3EVT 3EVU 3EVV 3EVW 3EVX 3EVY 3EVZ 3EW0 3EW1 3EW2 3EW3 3EW5 3EW7 3EW8 3EW9 3EWA 3EWB 3EWC 3EWD 3EWE 3EWF 3EWG 3EWH 3EWI 3EWJ 3EWK 3EWL 3EWM 3EWN 3EWO 3EWP 3EWQ 3EWR 3EWS 3EWT 3EWU 3EWV 3EWW 3EWX 3EWY 3EWZ 3EX1 3EX2 3EX3 3EX4 3EX6 3EX8 3EX9 3EXA 3EXB 3EXC 3EXD 3EXE 3EXF 3EXG 3EXH 3EXI 3EXM 3EXN 3EXO 3EXQ 3EXR 3EXS 3EXT 3EXU 3EXV 3EXW 3EXX 3EXY 3EXZ 3EY4 3EY5 3EY6 3EY7 3EY8 3EY9 3EYA 3EYB 3EYC 3EYD 3EYE 3EYF 3EYG 3EYH 3EYJ 3EYK 3EYL 3EYM 3EYO 3EYP 3EYQ 3EYS 3EYT 3EYU 3EYV 3EYW 3EYX 3EYY 3EZ0 3EZ1 3EZ2 3EZ3 3EZ4 3EZ6 3EZ7 3EZ8 3EZ9 3EZF 3EZG 3EZH 3EZI 3EZJ 3EZL 3EZM 3EZN 3EZO 3EZP 3EZQ 3EZR 3EZS 3EZT 3EZU 3EZV 3EZW 3EZX 3EZY 3EZZ 3F00 3F01 3F02 3F03 3F04 3F05 3F06 3F07 3F08 3F0A 3F0B 3F0C 3F0D 3F0E 3F0F 3F0G 3F0H 3F0I 3F0L 3F0M 3F0N 3F0O 3F0P 3F0Q 3F0R 3F0S 3F0T 3F0U 3F0V 3F0W 3F0X 3F0Y 3F0Z 3F10 3F11 3F12 3F13 3F14 3F15 3F16 3F17 3F18 3F19 3F1A 3F1B 3F1C 3F1I 3F1J 3F1K 3F1L 3F1N 3F1O 3F1P 3F1Q 3F1R 3F1S 3F1T 3F1V 3F1W 3F1X 3F1Y 3F1Z 3F28 3F29 3F2A 3F2E 3F2F 3F2G 3F2H 3F2I 3F2K 3F2L 3F2M 3F2N 3F2O 3F2P 3F2R 3F2U 3F2V 3F2Z 3F31 3F32 3F33 3F34 3F35 3F36 3F37 3F38 3F39 3F3A 3F3B 3F3C 3F3D 3F3E 3F3F 3F3G 3F3H 3F3K 3F3M 3F3P 3F3Q 3F3R 3F3S 3F3T 3F3U 3F3V 3F3W 3F3X 3F3Y 3F3Z 3F40 3F41 3F42 3F43 3F44 3F45 3F46 3F47 3F48 3F49 3F4A 3F4B 3F4C 3F4D 3F4F 3F4I 3F4J 3F4K 3F4L 3F4M 3F4N 3F4R 3F4S 3F4T 3F4V 3F4W 3F4X 3F4Y 3F4Z 3F50 3F51 3F52 3F53 3F56 3F57 3F58 3F59 3F5A 3F5B 3F5C 3F5D 3F5E 3F5F 3F5G 3F5H 3F5J 3F5K 3F5L 3F5M 3F5N 3F5O 3F5P 3F5Q 3F5R 3F5S 3F5T 3F5U 3F5V 3F5W 3F5X 3F61 3F62 3F63 3F64 3F65 3F66 3F67 3F68 3F69 3F6A 3F6B 3F6C 3F6D 3F6E 3F6F 3F6G 3F6H 3F6I 3F6J 3F6K 3F6L 3F6M 3F6N 3F6O 3F6P 3F6Q 3F6R 3F6S 3F6T 3F6U 3F6V 3F6W 3F6X 3F6Y 3F6Z 3F70 3F71 3F72 3F74 3F75 3F78 3F79 3F7A 3F7B 3F7C 3F7D 3F7E 3F7F 3F7G 3F7H 3F7I 3F7J 3F7K 3F7L 3F7M 3F7N 3F7O 3F7P 3F7Q 3F7R 3F7S 3F7T 3F7U 3F7V 3F7W 3F7X 3F7Y 3F7Z 3F80 3F81 3F82 3F83 3F84 3F85 3F86 3F87 3F88 3F89 3F8A 3F8B 3F8C 3F8D 3F8E 3F8F 3F8G 3F8H 3F8K 3F8L 3F8M 3F8N 3F8P 3F8R 3F8S 3F8T 3F8U 3F8V 3F8W 3F8X 3F8Y 3F8Z 3F90 3F91 3F92 3F95 3F96 3F97 3F98 3F99 3F9A 3F9B 3F9C 3F9D 3F9E 3F9F 3F9G 3F9H 3F9I 3F9K 3F9L 3F9M 3F9N 3F9O 3F9P 3F9Q 3F9R 3F9S 3F9T 3F9U 3F9V 3F9W 3F9X 3F9Y 3F9Z 3FA0 3FA2 3FA3 3FA4 3FA5 3FA6 3FA7 3FA8 3FA9 3FAA 3FAC 3FAD 3FAH 3FAI 3FAJ 3FAK 3FAL 3FAN 3FAO 3FAP 3FAQ 3FAS 3FAT 3FAU 3FAV 3FAW 3FAX 3FAY 3FAZ 3FB0 3FB1 3FB2 3FB3 3FB4 3FB5 3FB6 3FB7 3FB8 3FB9 3FBA 3FBB 3FBC 3FBE 3FBF 3FBG 3FBI 3FBK 3FBL 3FBM 3FBN 3FBO 3FBP 3FBQ 3FBR 3FBS 3FBT 3FBU 3FBV 3FBW 3FBX 3FBY 3FBZ 3FC0 3FC1 3FC2 3FC4 3FC5 3FC6 3FC7 3FC8 3FC9 3FCA 3FCB 3FCC 3FCD 3FCE 3FCF 3FCG 3FCH 3FCI 3FCJ 3FCK 3FCL 3FCM 3FCN 3FCO 3FCP 3FCQ 3FCR 3FCS 3FCT 3FCU 3FCV 3FCW 3FCX 3FCY 3FCZ 3FD0 3FD3 3FD4 3FD5 3FD6 3FD7 3FD8 3FD9 3FDB 3FDC 3FDD 3FDF 3FDG 3FDH 3FDI 3FDJ 3FDK 3FDL 3FDM 3FDN 3FDO 3FDR 3FDS 3FDT 3FDU 3FDW 3FDX 3FDY 3FDZ 3FE0 3FE1 3FE2 3FE3 3FE4 3FE5 3FE6 3FE7 3FE8 3FE9 3FEA 3FEC 3FED 3FEE 3FEF 3FEG 3FEH 3FEI 3FEJ 3FEK 3FEL 3FEM 3FEN 3FEO 3FEP 3FEQ 3FER 3FES 3FET 3FEU 3FEV 3FEW 3FEX 3FEY 3FEZ 3FF0 3FF1 3FF2 3FF3 3FF4 3FF5 3FF6 3FF7 3FF8 3FF9 3FFA 3FFB 3FFC 3FFD 3FFE 3FFG 3FFH 3FFI 3FFK 3FFL 3FFM 3FFN 3FFO 3FFP 3FFQ 3FFR 3FFS 3FFT 3FFU 3FFV 3FFW 3FFX 3FFY 3FFZ 3FG1 3FG2 3FG3 3FG4 3FG5 3FG6 3FG7 3FG8 3FG9 3FGA 3FGB 3FGC 3FGD 3FGE 3FGG 3FGH 3FGM 3FGN 3FGO 3FGP 3FGQ 3FGR 3FGS 3FGT 3FGU 3FGV 3FGW 3FGX 3FGY 3FGZ 3FH0 3FH1 3FH2 3FH3 3FH4 3FH5 3FH6 3FH7 3FH8 3FH9 3FHA 3FHB 3FHC 3FHD 3FHE 3FHF 3FHG 3FHH 3FHI 3FHJ 3FHK 3FHL 3FHM 3FHN 3FHO 3FHQ 3FHR 3FHU 3FHV 3FHW 3FHX 3FHY 3FI0 3FI2 3FI3 3FI4 3FI5 3FI6 3FI7 3FI8 3FI9 3FIA 3FIB 3FID 3FIE 3FIF 3FIG 3FII 3FIJ 3FIL 3FIM 3FIP 3FIQ 3FIR 3FIS 3FIT 3FIU 3FIV 3FIW 3FIX 3FJ1 3FJ2 3FJ4 3FJ5 3FJ6 3FJ7 3FJ8 3FJ9 3FJA 3FJB 3FJC 3FJD 3FJE 3FJF 3FJG 3FJH 3FJI 3FJJ 3FJK 3FJL 3FJM 3FJN 3FJO 3FJP 3FJQ 3FJS 3FJT 3FJU 3FJV 3FJW 3FJX 3FJY 3FJZ 3FK0 3FK1 3FK2 3FK3 3FK4 3FK5 3FK6 3FK7 3FK8 3FK9 3FKA 3FKB 3FKC 3FKD 3FKE 3FKF 3FKG 3FKH 3FKI 3FKJ 3FKK 3FKL 3FKM 3FKN 3FKO 3FKQ 3FKR 3FKS 3FKT 3FKU 3FKV 3FKW 3FKY 3FKZ 3FL0 3FL1 3FL2 3FL3 3FL4 3FL5 3FL7 3FL8 3FL9 3FLA 3FLB 3FLC 3FLD 3FLE 3FLF 3FLG 3FLH 3FLI 3FLJ 3FLK 3FLL 3FLM 3FLN 3FLO 3FLP 3FLQ 3FLR 3FLS 3FLT 3FLU 3FLV 3FLW 3FLY 3FLZ 3FM0 3FM1 3FM2 3FM3 3FM4 3FM5 3FM6 3FM7 3FM8 3FM9 3FMA 3FMB 3FMC 3FMD 3FME 3FMF 3FMG 3FMH 3FMI 3FMJ 3FMK 3FML 3FMM 3FMN 3FMO 3FMP 3FMQ 3FMR 3FMS 3FMU 3FMV 3FMW 3FMX 3FMY 3FMZ 3FN0 3FN1 3FN2 3FN3 3FN4 3FN5 3FN6 3FN7 3FN8 3FN9 3FNA 3FNB 3FNC 3FND 3FNE 3FNF 3FNG 3FNH 3FNI 3FNJ 3FNK 3FNL 3FNM 3FNN 3FNQ 3FNR 3FNS 3FNT 3FNU 3FNV 3FO0 3FO1 3FO2 3FO3 3FO5 3FO7 3FO8 3FO9 3FOA 3FOB 3FOC 3FOD 3FOG 3FOJ 3FOK 3FOL 3FOM 3FON 3FOO 3FOP 3FOQ 3FOR 3FOT 3FOU 3FOV 3FOW 3FP0 3FP2 3FP3 3FP4 3FP5 3FP6 3FP7 3FP8 3FP9 3FPA 3FPB 3FPC 3FPD 3FPE 3FPF 3FPG 3FPH 3FPI 3FPJ 3FPK 3FPL 3FPM 3FPN 3FPO 3FPP 3FPQ 3FPR 3FPS 3FPT 3FPU 3FPV 3FPW 3FPX 3FPY 3FPZ 3FQ0 3FQ1 3FQ2 3FQ3 3FQ4 3FQ6 3FQ7 3FQ8 3FQ9 3FQA 3FQC 3FQD 3FQE 3FQF 3FQG 3FQH 3FQI 3FQJ 3FQK 3FQL 3FQM 3FQN 3FQO 3FQQ 3FQR 3FQS 3FQT 3FQU 3FQV 3FQW 3FQX 3FQY 3FQZ 3FR0 3FR1 3FR2 3FR3 3FR4 3FR5 3FR6 3FR7 3FR8 3FR9 3FRA 3FRB 3FRC 3FRD 3FRE 3FRF 3FRG 3FRH 3FRI 3FRJ 3FRK 3FRL 3FRM 3FRN 3FRO 3FRP 3FRQ 3FRR 3FRS 3FRT 3FRU 3FRV 3FRW 3FRX 3FRY 3FRZ 3FS1 3FS2 3FS3 3FS4 3FS5 3FS6 3FS7 3FS8 3FS9 3FSA 3FSB 3FSC 3FSD 3FSE 3FSF 3FSG 3FSH 3FSJ 3FSK 3FSL 3FSM 3FSN 3FSO 3FSR 3FSS 3FST 3FSU 3FSV 3FSW 3FSX 3FSY 3FSZ 3FT0 3FT1 3FT2 3FT3 3FT4 3FT5 3FT7 3FT8 3FT9 3FTB 3FTC 3FTD 3FTG 3FTH 3FTJ 3FTK 3FTL 3FTN 3FTO 3FTP 3FTQ 3FTR 3FTS 3FTT 3FTU 3FTV 3FTW 3FTX 3FTY 3FTZ 3FU0 3FU1 3FU3 3FU5 3FU6 3FU7 3FU8 3FU9 3FUA 3FUB 3FUC 3FUD 3FUE 3FUF 3FUG 3FUH 3FUI 3FUJ 3FUK 3FUL 3FUM 3FUN 3FUO 3FUP 3FUQ 3FUR 3FUS 3FUT 3FUU 3FUV 3FUW 3FUX 3FUY 3FUZ 3FV1 3FV2 3FV3 3FV4 3FV5 3FV6 3FV7 3FV8 3FV9 3FVA 3FVB 3FVC 3FVD 3FVE 3FVF 3FVG 3FVH 3FVI 3FVJ 3FVK 3FVL 3FVM 3FVN 3FVO 3FVP 3FVQ 3FVR 3FVS 3FVT 3FVU 3FVV 3FVW 3FVX 3FVY 3FVZ 3FW0 3FW1 3FW2 3FW3 3FW4 3FW5 3FW6 3FW7 3FW8 3FW9 3FWA 3FWB 3FWC 3FWE 3FWF 3FWG 3FWH 3FWI 3FWJ 3FWK 3FWL 3FWN 3FWP 3FWQ 3FWR 3FWS 3FWT 3FWU 3FWV 3FWW 3FWX 3FWY 3FWZ 3FX0 3FX2 3FX3 3FX4 3FX5 3FX6 3FX7 3FXA 3FXB 3FXD 3FXE 3FXG 3FXH 3FXI 3FXJ 3FXK 3FXL 3FXM 3FXO 3FXP 3FXQ 3FXR 3FXS 3FXT 3FXU 3FXV 3FXW 3FXX 3FXY 3FXZ 3FY0 3FY1 3FY2 3FY3 3FY4 3FY5 3FY6 3FY7 3FY8 3FY9 3FYA 3FYB 3FYC 3FYD 3FYE 3FYF 3FYG 3FYH 3FYI 3FYJ 3FYK 3FYM 3FYN 3FYO 3FYP 3FYQ 3FYR 3FYS 3FYT 3FYU 3FYV 3FYW 3FYX 3FYY 3FYZ 3FZ0 3FZ1 3FZ2 3FZ3 3FZ4 3FZ5 3FZ6 3FZ7 3FZ8 3FZ9 3FZA 3FZB 3FZC 3FZD 3FZE 3FZF 3FZG 3FZH 3FZI 3FZJ 3FZK 3FZL 3FZM 3FZN 3FZO 3FZP 3FZQ 3FZR 3FZS 3FZT 3FZU 3FZV 3FZW 3FZX 3FZY 3FZZ 3G01 3G02 3G03 3G04 3G05 3G06 3G07 3G08 3G0A 3G0B 3G0C 3G0D 3G0E 3G0F 3G0G 3G0I 3G0J 3G0K 3G0L 3G0M 3G0O 3G0S 3G0T 3G0U 3G0V 3G0W 3G0X 3G0Y 3G0Z 3G10 3G11 3G12 3G13 3G14 3G15 3G16 3G17 3G18 3G19 3G1A 3G1B 3G1C 3G1D 3G1E 3G1F 3G1G 3G1H 3G1I 3G1J 3G1K 3G1L 3G1M 3G1N 3G1O 3G1P 3G1Q 3G1R 3G1S 3G1T 3G1U 3G1V 3G1W 3G1X 3G1Y 3G1Z 3G20 3G21 3G22 3G23 3G24 3G25 3G26 3G27 3G28 3G29 3G2B 3G2E 3G2F 3G2G 3G2H 3G2I 3G2J 3G2K 3G2L 3G2M 3G2N 3G2O 3G2P 3G2Q 3G2S 3G2T 3G2U 3G2V 3G2W 3G2X 3G2Y 3G2Z 3G30 3G31 3G32 3G33 3G34 3G35 3G36 3G39 3G3A 3G3B 3G3D 3G3E 3G3F 3G3G 3G3H 3G3I 3G3J 3G3K 3G3L 3G3M 3G3N 3G3O 3G3P 3G3Q 3G3R 3G3S 3G3T 3G3U 3G3V 3G3W 3G3X 3G3Z 3G40 3G42 3G43 3G45 3G46 3G48 3G49 3G4A 3G4C 3G4D 3G4E 3G4F 3G4G 3G4H 3G4I 3G4K 3G4L 3G4N 3G4O 3G4P 3G4Q 3G4R 3G4U 3G4V 3G4W 3G4X 3G4Y 3G4Z 3G50 3G51 3G52 3G53 3G56 3G58 3G59 3G5A 3G5B 3G5C 3G5D 3G5E 3G5F 3G5G 3G5H 3G5I 3G5J 3G5K 3G5L 3G5M 3G5N 3G5O 3G5P 3G5Q 3G5R 3G5S 3G5T 3G5U 3G5V 3G5W 3G5X 3G5Y 3G5Z 3G60 3G61 3G64 3G65 3G66 3G67 3G68 3G69 3G6A 3G6B 3G6D 3G6G 3G6H 3G6I 3G6J 3G6K 3G6L 3G6M 3G6N 3G6O 3G6S 3G6W 3G6Z 3G70 3G72 3G74 3G75 3G76 3G77 3G79 3G7A 3G7B 3G7C 3G7D 3G7E 3G7F 3G7G 3G7I 3G7J 3G7K 3G7L 3G7M 3G7N 3G7P 3G7Q 3G7R 3G7S 3G7T 3G7U 3G7V 3G7W 3G7X 3G7Y 3G7Z 3G80 3G81 3G82 3G83 3G84 3G85 3G86 3G87 3G88 3G89 3G8A 3G8B 3G8C 3G8D 3G8E 3G8F 3G8G 3G8H 3G8I 3G8K 3G8L 3G8M 3G8O 3G8Q 3G8R 3G8V 3G8W 3G8Y 3G8Z 3G90 3G91 3G93 3G98 3G9A 3G9B 3G9D 3G9E 3G9G 3G9H 3G9K 3G9L 3G9N 3G9Q 3G9R 3G9T 3G9U 3G9V 3G9W 3G9X 3G9Z 3GA0 3GA1 3GA2 3GA3 3GA4 3GA5 3GA7 3GA8 3GA9 3GAA 3GAB 3GAC 3GAD 3GAE 3GAF 3GAG 3GAH 3GAI 3GAJ 3GAK 3GAL 3GAM 3GAN 3GAQ 3GAR 3GAS 3GAX 3GAY 3GAZ 3GB0 3GB2 3GB3 3GB4 3GB5 3GB6 3GB7 3GB8 3GB9 3GBA 3GBB 3GBD 3GBE 3GBF 3GBG 3GBH 3GBJ 3GBK 3GBL 3GBM 3GBN 3GBO 3GBP 3GBR 3GBS 3GBT 3GBU 3GBV 3GBW 3GBX 3GBY 3GBZ 3GC0 3GC1 3GC2 3GC3 3GC4 3GC5 3GC6 3GC7 3GC8 3GC9 3GCB 3GCD 3GCE 3GCF 3GCG 3GCH 3GCI 3GCJ 3GCK 3GCL 3GCM 3GCN 3GCO 3GCP 3GCQ 3GCS 3GCT 3GCU 3GCV 3GCW 3GCX 3GCZ 3GD0 3GD1 3GD2 3GD3 3GD4 3GD5 3GD6 3GD7 3GD8 3GD9 3GDB 3GDC 3GDE 3GDF 3GDG 3GDH 3GDI 3GDJ 3GDK 3GDL 3GDM 3GDN 3GDO 3GDP 3GDQ 3GDR 3GDS 3GDT 3GDU 3GDV 3GDW 3GDZ 3GE1 3GE2 3GE3 3GE4 3GE5 3GE6 3GE7 3GE8 3GE9 3GEA 3GEB 3GEC 3GED 3GEE 3GEF 3GEG 3GEH 3GEI 3GEK 3GEL 3GEM 3GEN 3GEO 3GEP 3GEQ 3GET 3GEU 3GEW 3GEX 3GEY 3GEZ 3GF0 3GF2 3GF3 3GF4 3GF5 3GF6 3GF7 3GF8 3GF9 3GFA 3GFB 3GFC 3GFD 3GFE 3GFF 3GFG 3GFH 3GFJ 3GFK 3GFL 3GFM 3GFO 3GFP 3GFQ 3GFR 3GFS 3GFT 3GFU 3GFV 3GFW 3GFX 3GFY 3GFZ 3GG0 3GG1 3GG2 3GG3 3GG4 3GG5 3GG6 3GG7 3GG8 3GG9 3GGA 3GGC 3GGD 3GGE 3GGF 3GGG 3GGH 3GGJ 3GGL 3GGM 3GGN 3GGO 3GGP 3GGQ 3GGR 3GGS 3GGU 3GGV 3GGW 3GGX 3GGY 3GGZ 3GH0 3GH1 3GH2 3GH3 3GH4 3GH5 3GH6 3GH7 3GH8 3GH9 3GHA 3GHB 3GHC 3GHD 3GHE 3GHF 3GHG 3GHH 3GHJ 3GHM 3GHN 3GHP 3GHQ 3GHR 3GHS 3GHT 3GHU 3GHV 3GHW 3GHY 3GHZ 3GI0 3GI1 3GI2 3GI3 3GI4 3GI5 3GI6 3GI7 3GI8 3GI9 3GIA 3GIC 3GID 3GIE 3GIF 3GIG 3GIN 3GIO 3GIP 3GIQ 3GIR 3GIS 3GIT 3GIU 3GIV 3GIW 3GIX 3GIY 3GIZ 3GJ0 3GJ1 3GJ2 3GJ3 3GJ4 3GJ5 3GJ6 3GJ7 3GJ8 3GJ9 3GJA 3GJB 3GJC 3GJD 3GJE 3GJF 3GJN 3GJO 3GJP 3GJQ 3GJR 3GJS 3GJT 3GJU 3GJW 3GJX 3GJY 3GJZ 3GK0 3GK1 3GK2 3GK3 3GK4 3GK5 3GK6 3GK7 3GK8 3GK9 3GKA 3GKB 3GKE 3GKF 3GKH 3GKI 3GKJ 3GKK 3GKL 3GKM 3GKN 3GKO 3GKQ 3GKR 3GKT 3GKU 3GKV 3GKW 3GKX 3GKY 3GKZ 3GL0 3GL1 3GL2 3GL3 3GL4 3GL5 3GL6 3GL9 3GLA 3GLB 3GLC 3GLD 3GLE 3GLH 3GLJ 3GLK 3GLL 3GLM 3GLN 3GLQ 3GLR 3GLS 3GLT 3GLU 3GLV 3GLW 3GLX 3GLY 3GLZ 3GM0 3GM1 3GM2 3GM3 3GM5 3GM6 3GM8 3GMA 3GMB 3GMC 3GMD 3GME 3GMF 3GMG 3GMH 3GMI 3GMJ 3GML 3GMM 3GMN 3GMO 3GMP 3GMQ 3GMR 3GMS 3GMT 3GMU 3GMV 3GMW 3GMX 3GMY 3GMZ 3GN0 3GN1 3GN2 3GN3 3GN4 3GN5 3GN6 3GN7 3GN8 3GN9 3GNC 3GND 3GNE 3GNF 3GNG 3GNI 3GNJ 3GNL 3GNM 3GNN 3GNO 3GNP 3GNQ 3GNR 3GNS 3GNT 3GNU 3GNV 3GNW 3GNX 3GNY 3GNZ 3GO0 3GO1 3GO2 3GO4 3GO5 3GO6 3GO7 3GO9 3GOA 3GOB 3GOC 3GOD 3GOE 3GOF 3GOH 3GOI 3GOK 3GOL 3GON 3GOP 3GOQ 3GOR 3GOS 3GOU 3GOV 3GOY 3GOZ 3GP0 3GP2 3GP3 3GP4 3GP5 3GP6 3GP7 3GP9 3GPA 3GPB 3GPC 3GPD 3GPE 3GPG 3GPH 3GPI 3GPJ 3GPK 3GPM 3GPN 3GPO 3GPR 3GPS 3GPT 3GPV 3GPW 3GQ0 3GQ1 3GQ2 3GQ7 3GQ8 3GQ9 3GQA 3GQB 3GQE 3GQF 3GQG 3GQH 3GQI 3GQJ 3GQK 3GQL 3GQM 3GQO 3GQP 3GQQ 3GQR 3GQS 3GQT 3GQU 3GQV 3GQX 3GQY 3GQZ 3GR0 3GR1 3GR2 3GR3 3GR4 3GR5 3GR6 3GR7 3GR8 3GR9 3GRA 3GRB 3GRC 3GRD 3GRE 3GRF 3GRG 3GRH 3GRI 3GRJ 3GRK 3GRL 3GRN 3GRO 3GRP 3GRR 3GRS 3GRT 3GRU 3GRV 3GRW 3GRY 3GRZ 3GS0 3GS2 3GS3 3GS4 3GS6 3GS7 3GS9 3GSB 3GSD 3GSE 3GSG 3GSH 3GSI 3GSL 3GSM 3GSN 3GSO 3GSP 3GSQ 3GSR 3GSS 3GST 3GSU 3GSV 3GSW 3GSX 3GSY 3GSZ 3GT0 3GT2 3GT3 3GT4 3GT5 3GT6 3GT7 3GT8 3GT9 3GTA 3GTC 3GTD 3GTE 3GTF 3GTH 3GTI 3GTN 3GTS 3GTT 3GTU 3GTV 3GTX 3GTY 3GTZ 3GU0 3GU1 3GU2 3GU3 3GU4 3GU5 3GU6 3GU7 3GU8 3GU9 3GUA 3GUB 3GUC 3GUD 3GUE 3GUF 3GUG 3GUH 3GUI 3GUJ 3GUK 3GUL 3GUM 3GUN 3GUO 3GUP 3GUQ 3GUR 3GUS 3GUU 3GUV 3GUW 3GUX 3GUY 3GUZ 3GV0 3GV1 3GV2 3GV3 3GV4 3GV6 3GV9 3GVA 3GVB 3GVC 3GVD 3GVE 3GVF 3GVG 3GVH 3GVI 3GVJ 3GVK 3GVL 3GVM 3GVO 3GVP 3GVQ 3GVR 3GVT 3GVU 3GVV 3GVW 3GVX 3GVY 3GVZ 3GW0 3GW1 3GW2 3GW3 3GW4 3GW5 3GW6 3GW7 3GW8 3GW9 3GWA 3GWB 3GWC 3GWD 3GWE 3GWF 3GWG 3GWH 3GWI 3GWJ 3GWK 3GWL 3GWM 3GWN 3GWO 3GWP 3GWQ 3GWR 3GWS 3GWT 3GWU 3GWV 3GWW 3GWX 3GWY 3GWZ 3GX0 3GX1 3GX8 3GX9 3GXA 3GXB 3GXD 3GXE 3GXF 3GXG 3GXH 3GXI 3GXK 3GXL 3GXM 3GXN 3GXO 3GXP 3GXR 3GXT 3GXU 3GXV 3GXW 3GXX 3GXY 3GXZ 3GY0 3GY1 3GY2 3GY3 3GY4 3GY5 3GY6 3GY7 3GY8 3GY9 3GYA 3GYB 3GYC 3GYD 3GYE 3GYF 3GYG 3GYI 3GYJ 3GYK 3GYL 3GYM 3GYN 3GYO 3GYP 3GYQ 3GYR 3GYS 3GYT 3GYU 3GYV 3GYW 3GYX 3GYY 3GYZ 3GZ0 3GZ1 3GZ2 3GZ3 3GZ4 3GZ5 3GZ7 3GZ8 3GZ9 3GZA 3GZB 3GZC 3GZD 3GZE 3GZF 3GZG 3GZH 3GZI 3GZJ 3GZK 3GZL 3GZM 3GZN 3GZO 3GZP 3GZQ 3GZR 3GZS 3GZX 3GZY 3H00 3H01 3H02 3H03 3H04 3H05 3H06 3H07 3H08 3H09 3H0A 3H0B 3H0C 3H0E 3H0F 3H0G 3H0H 3H0I 3H0J 3H0K 3H0L 3H0M 3H0N 3H0O 3H0P 3H0Q 3H0R 3H0S 3H0T 3H0U 3H0V 3H0W 3H0X 3H0Y 3H0Z 3H10 3H11 3H12 3H13 3H14 3H16 3H17 3H18 3H19 3H1A 3H1B 3H1C 3H1D 3H1E 3H1F 3H1G 3H1H 3H1I 3H1J 3H1K 3H1L 3H1M 3H1N 3H1O 3H1P 3H1Q 3H1R 3H1S 3H1T 3H1U 3H1V 3H1W 3H1X 3H1Y 3H1Z 3H20 3H21 3H22 3H23 3H24 3H26 3H2A 3H2B 3H2C 3H2D 3H2E 3H2F 3H2G 3H2H 3H2I 3H2J 3H2K 3H2L 3H2M 3H2N 3H2O 3H2P 3H2Q 3H2S 3H2T 3H2U 3H2V 3H2W 3H2X 3H2Y 3H2Z 3H30 3H31 3H32 3H33 3H34 3H35 3H36 3H37 3H38 3H39 3H3A 3H3B 3H3C 3H3D 3H3E 3H3F 3H3G 3H3H 3H3I 3H3J 3H3K 3H3L 3H3M 3H3N 3H3O 3H3P 3H3Q 3H3R 3H3S 3H3T 3H3U 3H3X 3H3Z 3H41 3H42 3H43 3H44 3H45 3H46 3H47 3H49 3H4C 3H4E 3H4F 3H4G 3H4H 3H4I 3H4J 3H4K 3H4L 3H4M 3H4N 3H4O 3H4P 3H4Q 3H4R 3H4S 3H4T 3H4V 3H4W 3H4X 3H4Y 3H4Z 3H50 3H51 3H52 3H53 3H54 3H55 3H56 3H57 3H58 3H59 3H5A 3H5B 3H5C 3H5E 3H5F 3H5G 3H5H 3H5I 3H5J 3H5K 3H5L 3H5N 3H5O 3H5Q 3H5R 3H5S 3H5T 3H5U 3H5V 3H5W 3H5Z 3H60 3H61 3H62 3H63 3H64 3H65 3H66 3H67 3H68 3H69 3H6A 3H6C 3H6D 3H6E 3H6F 3H6G 3H6H 3H6I 3H6J 3H6K 3H6M 3H6N 3H6O 3H6P 3H6Q 3H6R 3H6S 3H6T 3H6U 3H6V 3H6W 3H6X 3H6Z 3H70 3H71 3H72 3H73 3H74 3H75 3H76 3H77 3H78 3H79 3H7A 3H7B 3H7C 3H7D 3H7F 3H7G 3H7H 3H7I 3H7J 3H7K 3H7L 3H7M 3H7N 3H7O 3H7P 3H7Q 3H7R 3H7S 3H7T 3H7U 3H7V 3H7W 3H7X 3H7Y 3H7Z 3H80 3H81 3H82 3H83 3H84 3H85 3H86 3H87 3H89 3H8A 3H8B 3H8C 3H8D 3H8E 3H8F 3H8G 3H8H 3H8I 3H8J 3H8K 3H8L 3H8M 3H8N 3H8Q 3H8S 3H8T 3H8U 3H8V 3H8W 3H8Y 3H8Z 3H90 3H91 3H92 3H93 3H94 3H95 3H96 3H97 3H98 3H99 3H9A 3H9B 3H9C 3H9D 3H9E 3H9F 3H9G 3H9H 3H9J 3H9K 3H9M 3H9N 3H9O 3H9P 3H9Q 3H9R 3H9S 3H9U 3H9V 3H9W 3H9X 3H9Y 3H9Z 3HA0 3HA1 3HA2 3HA3 3HA4 3HA5 3HA6 3HA7 3HA8 3HA9 3HAB 3HAC 3HAD 3HAE 3HAF 3HAG 3HAH 3HAI 3HAJ 3HAK 3HAL 3HAM 3HAN 3HAO 3HAP 3HAQ 3HAR 3HAS 3HAT 3HAU 3HAV 3HAW 3HAZ 3HB0 3HB1 3HB2 3HB3 3HB4 3HB5 3HB6 3HB7 3HB8 3HB9 3HBA 3HBB 3HBC 3HBD 3HBE 3HBF 3HBG 3HBH 3HBJ 3HBK 3HBL 3HBM 3HBN 3HBO 3HBP 3HBQ 3HBR 3HBT 3HBU 3HBV 3HBW 3HBX 3HBZ 3HC0 3HC1 3HC2 3HC3 3HC4 3HC5 3HC6 3HC7 3HC8 3HC9 3HCA 3HCB 3HCC 3HCD 3HCE 3HCF 3HCG 3HCH 3HCI 3HCJ 3HCM 3HCN 3HCO 3HCP 3HCQ 3HCR 3HCS 3HCT 3HCU 3HCV 3HCW 3HCX 3HCY 3HCZ 3HD0 3HD1 3HD2 3HD3 3HD4 3HD5 3HD6 3HD7 3HD8 3HDA 3HDB 3HDC 3HDE 3HDF 3HDG 3HDH 3HDI 3HDJ 3HDK 3HDL 3HDM 3HDN 3HDO 3HDP 3HDQ 3HDS 3HDT 3HDU 3HDV 3HDX 3HDY 3HDZ 3HE0 3HE1 3HE2 3HE3 3HE4 3HE5 3HE6 3HE7 3HE8 3HEA 3HEB 3HEC 3HEE 3HEF 3HEG 3HEI 3HEJ 3HEK 3HEM 3HEN 3HEO 3HEP 3HEQ 3HER 3HES 3HET 3HEU 3HEV 3HEW 3HEX 3HEY 3HEZ 3HF0 3HF1 3HF2 3HF3 3HF4 3HF5 3HF6 3HF7 3HF8 3HF9 3HFA 3HFB 3HFC 3HFD 3HFE 3HFF 3HFG 3HFH 3HFI 3HFJ 3HFK 3HFM 3HFN 3HFO 3HFP 3HFQ 3HFR 3HFS 3HFT 3HFU 3HFV 3HFW 3HFX 3HFY 3HFZ 3HG0 3HG1 3HG2 3HG3 3HG4 3HG5 3HG6 3HG7 3HG9 3HGB 3HGF 3HGG 3HGI 3HGJ 3HGK 3HGL 3HGM 3HGO 3HGP 3HGQ 3HGR 3HGS 3HGT 3HGU 3HGV 3HGW 3HGX 3HGY 3HGZ 3HH0 3HH1 3HH2 3HH3 3HH4 3HH5 3HH6 3HH7 3HH8 3HHA 3HHB 3HHC 3HHD 3HHE 3HHF 3HHG 3HHH 3HHI 3HHJ 3HHK 3HHL 3HHM 3HHP 3HHQ 3HHR 3HHS 3HHT 3HHU 3HHV 3HHW 3HHX 3HHY 3HI0 3HI1 3HI2 3HI4 3HI5 3HI6 3HI7 3HI8 3HI9 3HIA 3HIB 3HIC 3HID 3HIE 3HIF 3HIG 3HIH 3HII 3HIJ 3HIK 3HIL 3HIM 3HIN 3HIO 3HIP 3HIQ 3HIS 3HIT 3HIU 3HIV 3HIW 3HIX 3HIY 3HIZ 3HJ0 3HJ1 3HJ2 3HJ3 3HJ4 3HJ5 3HJ6 3HJ7 3HJ8 3HJ9 3HJA 3HJB 3HJC 3HJD 3HJE 3HJG 3HJH 3HJI 3HJJ 3HJK 3HJL 3HJM 3HJN 3HJO 3HJP 3HJQ 3HJR 3HJS 3HJT 3HJU 3HJV 3HJX 3HJZ 3HK0 3HK1 3HK3 3HK4 3HK5 3HK6 3HK7 3HK8 3HK9 3HKA 3HKB 3HKC 3HKD 3HKE 3HKF 3HKI 3HKJ 3HKK 3HKL 3HKM 3HKN 3HKO 3HKP 3HKQ 3HKR 3HKS 3HKT 3HKU 3HKV 3HKW 3HKX 3HKY 3HKZ 3HL0 3HL1 3HL3 3HL4 3HL5 3HL6 3HL7 3HL8 3HL9 3HLA 3HLB 3HLC 3HLD 3HLE 3HLF 3HLG 3HLH 3HLI 3HLJ 3HLK 3HLL 3HLM 3HLN 3HLO 3HLP 3HLR 3HLS 3HLT 3HLU 3HLV 3HLW 3HLX 3HLY 3HLZ 3HM0 3HM1 3HM2 3HM3 3HM4 3HM5 3HM6 3HM7 3HM8 3HMB 3HMC 3HME 3HMF 3HMG 3HMH 3HMI 3HMJ 3HMK 3HML 3HMM 3HMN 3HMO 3HMP 3HMQ 3HMR 3HMS 3HMT 3HMU 3HMV 3HMW 3HMX 3HMY 3HMZ 3HN0 3HN1 3HN2 3HN3 3HN4 3HN5 3HN6 3HN7 3HN8 3HNA 3HNB 3HNC 3HND 3HNE 3HNF 3HNG 3HNH 3HNI 3HNJ 3HNK 3HNL 3HNM 3HNO 3HNP 3HNQ 3HNR 3HNS 3HNT 3HNU 3HNV 3HNW 3HNX 3HNY 3HNZ 3HO0 3HO2 3HO3 3HO4 3HO5 3HO6 3HO7 3HO8 3HO9 3HOA 3HOB 3HOC 3HOD 3HOE 3HOF 3HOG 3HOH 3HOI 3HOJ 3HOK 3HOL 3HOM 3HON 3HOP 3HOR 3HP0 3HP1 3HP2 3HP3 3HP4 3HP5 3HP7 3HP8 3HP9 3HPA 3HPB 3HPC 3HPD 3HPE 3HPF 3HPG 3HPH 3HPI 3HPJ 3HPK 3HPL 3HPM 3HPN 3HPQ 3HPR 3HPS 3HPT 3HPV 3HPW 3HPX 3HPY 3HPZ 3HQ0 3HQ1 3HQ2 3HQ4 3HQ5 3HQ6 3HQ7 3HQ8 3HQ9 3HQA 3HQB 3HQC 3HQD 3HQH 3HQI 3HQJ 3HQL 3HQM 3HQN 3HQO 3HQP 3HQQ 3HQR 3HQT 3HQU 3HQW 3HQX 3HQY 3HQZ 3HR0 3HR1 3HR4 3HR5 3HR6 3HR7 3HR8 3HR9 3HRA 3HRB 3HRC 3HRD 3HRE 3HRF 3HRG 3HRH 3HRI 3HRK 3HRL 3HRM 3HRN 3HRO 3HRP 3HRQ 3HRR 3HRS 3HRT 3HRU 3HRV 3HRW 3HRX 3HRY 3HRZ 3HS0 3HS2 3HS3 3HS4 3HS5 3HS6 3HS7 3HS8 3HS9 3HSA 3HSC 3HSD 3HSE 3HSG 3HSH 3HSI 3HSJ 3HSK 3HSL 3HSM 3HSN 3HSO 3HSP 3HSQ 3HSR 3HSS 3HST 3HSU 3HSV 3HSW 3HSY 3HSZ 3HT0 3HT1 3HT2 3HT4 3HT5 3HT6 3HT7 3HT8 3HT9 3HTA 3HTB 3HTC 3HTD 3HTE 3HTF 3HTG 3HTH 3HTI 3HTJ 3HTK 3HTL 3HTM 3HTN 3HTO 3HTP 3HTQ 3HTR 3HTT 3HTU 3HTV 3HTW 3HTY 3HTZ 3HU1 3HU2 3HU3 3HU5 3HU6 3HU7 3HU8 3HU9 3HUA 3HUB 3HUC 3HUD 3HUE 3HUF 3HUG 3HUH 3HUI 3HUJ 3HUK 3HUL 3HUM 3HUN 3HUO 3HUP 3HUQ 3HUR 3HUS 3HUT 3HUU 3HUV 3HV0 3HV1 3HV2 3HV3 3HV4 3HV5 3HV6 3HV7 3HV8 3HV9 3HVA 3HVC 3HVD 3HVE 3HVF 3HVG 3HVH 3HVI 3HVJ 3HVK 3HVL 3HVM 3HVN 3HVO 3HVP 3HVQ 3HVS 3HVT 3HVU 3HVV 3HVW 3HVX 3HVY 3HVZ 3HW1 3HW2 3HW3 3HW4 3HW5 3HW6 3HW7 3HW9 3HWB 3HWC 3HWD 3HWE 3HWF 3HWG 3HWI 3HWJ 3HWK 3HWL 3HWN 3HWO 3HWP 3HWR 3HWS 3HWU 3HWW 3HWX 3HX1 3HX3 3HX4 3HX6 3HX8 3HX9 3HXA 3HXB 3HXC 3HXD 3HXE 3HXF 3HXG 3HXI 3HXJ 3HXK 3HXL 3HXN 3HXP 3HXR 3HXS 3HXT 3HXU 3HXV 3HXW 3HXX 3HXY 3HXZ 3HY0 3HY1 3HY2 3HY3 3HY4 3HY5 3HY6 3HY7 3HY8 3HY9 3HYB 3HYC 3HYD 3HYE 3HYF 3HYG 3HYH 3HYI 3HYJ 3HYK 3HYL 3HYM 3HYN 3HYO 3HYP 3HYQ 3HYR 3HYS 3HYT 3HYU 3HYV 3HYW 3HYX 3HYY 3HYZ 3HZ1 3HZ2 3HZ3 3HZ4 3HZ5 3HZ6 3HZ7 3HZ8 3HZA 3HZB 3HZC 3HZD 3HZE 3HZF 3HZG 3HZH 3HZJ 3HZK 3HZL 3HZM 3HZN 3HZO 3HZP 3HZQ 3HZR 3HZS 3HZT 3HZU 3HZV 3HZW 3HZX 3HZY 3HZZ 3I00 3I01 3I02 3I03 3I04 3I05 3I06 3I07 3I08 3I09 3I0A 3I0C 3I0D 3I0E 3I0F 3I0G 3I0H 3I0I 3I0J 3I0K 3I0L 3I0M 3I0N 3I0O 3I0P 3I0Q 3I0R 3I0S 3I0T 3I0U 3I0V 3I0Y 3I0Z 3I10 3I11 3I12 3I13 3I14 3I15 3I16 3I17 3I18 3I19 3I1A 3I1C 3I1E 3I1F 3I1G 3I1H 3I1I 3I1J 3I1K 3I1L 3I1U 3I1Y 3I23 3I24 3I25 3I26 3I27 3I28 3I29 3I2A 3I2B 3I2C 3I2D 3I2E 3I2F 3I2G 3I2H 3I2I 3I2J 3I2K 3I2L 3I2M 3I2N 3I2T 3I2V 3I2W 3I2X 3I2Y 3I2Z 3I30 3I31 3I32 3I33 3I34 3I35 3I36 3I37 3I38 3I39 3I3A 3I3B 3I3C 3I3D 3I3E 3I3F 3I3G 3I3H 3I3I 3I3J 3I3L 3I3N 3I3O 3I3Q 3I3R 3I3S 3I3T 3I3U 3I3V 3I3W 3I3X 3I3Y 3I3Z 3I40 3I41 3I42 3I43 3I44 3I45 3I46 3I47 3I48 3I4A 3I4B 3I4C 3I4D 3I4E 3I4F 3I4G 3I4H 3I4I 3I4J 3I4K 3I4L 3I4O 3I4P 3I4Q 3I4R 3I4S 3I4T 3I4U 3I4V 3I4W 3I4X 3I4Y 3I4Z 3I50 3I51 3I52 3I53 3I54 3I57 3I58 3I59 3I5A 3I5B 3I5C 3I5D 3I5F 3I5G 3I5H 3I5I 3I5J 3I5K 3I5M 3I5N 3I5O 3I5P 3I5Q 3I5R 3I5S 3I5T 3I5U 3I5V 3I5W 3I5Z 3I60 3I63 3I64 3I65 3I67 3I68 3I69 3I6A 3I6B 3I6C 3I6D 3I6E 3I6F 3I6G 3I6H 3I6I 3I6J 3I6K 3I6L 3I6M 3I6N 3I6O 3I6P 3I6Q 3I6R 3I6S 3I6T 3I6U 3I6V 3I6W 3I6X 3I6Y 3I6Z 3I70 3I71 3I72 3I73 3I74 3I75 3I76 3I77 3I78 3I79 3I7A 3I7B 3I7C 3I7D 3I7E 3I7F 3I7G 3I7H 3I7I 3I7J 3I7K 3I7L 3I7M 3I7N 3I7O 3I7P 3I7Q 3I7R 3I7S 3I7T 3I7U 3I7V 3I7W 3I7X 3I7Y 3I7Z 3I80 3I81 3I82 3I83 3I84 3I85 3I86 3I87 3I89 3I8A 3I8B 3I8C 3I8E 3I8N 3I8O 3I8P 3I8R 3I8S 3I8T 3I8U 3I8V 3I8W 3I8X 3I8Z 3I90 3I91 3I92 3I93 3I94 3I95 3I96 3I97 3I98 3I99 3I9A 3I9F 3I9G 3I9H 3I9I 3I9J 3I9K 3I9L 3I9M 3I9N 3I9O 3I9P 3I9Q 3I9S 3I9T 3I9U 3I9V 3I9W 3I9X 3I9Y 3I9Z 3IA0 3IA1 3IA2 3IA3 3IA4 3IA5 3IA6 3IA7 3IA8 3IA9 3IAA 3IAC 3IAD 3IAE 3IAF 3IAH 3IAI 3IAJ 3IAK 3IAL 3IAM 3IAN 3IAO 3IAP 3IAQ 3IAR 3IAS 3IAU 3IAV 3IAW 3IAX 3IAZ 3IB0 3IB1 3IB2 3IB3 3IB4 3IB5 3IB6 3IB7 3IB8 3IB9 3IBA 3IBB 3IBC 3IBD 3IBE 3IBF 3IBG 3IBH 3IBI 3IBJ 3IBL 3IBM 3IBN 3IBO 3IBP 3IBQ 3IBR 3IBS 3IBT 3IBU 3IBV 3IBW 3IBX 3IBY 3IBZ 3IC0 3IC1 3IC2 3IC3 3IC4 3IC5 3IC6 3IC7 3IC8 3IC9 3ICA 3ICB 3ICC 3ICD 3ICF 3ICH 3ICI 3ICJ 3ICK 3ICL 3ICM 3ICN 3ICO 3ICP 3ICR 3ICS 3ICT 3ICU 3ICV 3ICW 3ICX 3ICY 3ICZ 3ID0 3ID1 3ID2 3ID3 3ID4 3ID6 3ID7 3ID8 3ID9 3IDA 3IDB 3IDC 3IDD 3IDE 3IDF 3IDG 3IDH 3IDI 3IDJ 3IDM 3IDN 3IDO 3IDP 3IDQ 3IDS 3IDU 3IDV 3IDW 3IDX 3IDY 3IDZ 3IE0 3IE2 3IE3 3IE4 3IE5 3IE7 3IE9 3IEA 3IEB 3IEC 3IED 3IEE 3IEF 3IEG 3IEH 3IEI 3IEJ 3IEK 3IEL 3IEO 3IEP 3IEQ 3IER 3IES 3IET 3IEU 3IEW 3IEX 3IEY 3IEZ 3IF0 3IF1 3IF2 3IF4 3IF5 3IF6 3IF7 3IF8 3IF9 3IFA 3IFC 3IFD 3IFE 3IFJ 3IFK 3IFL 3IFN 3IFO 3IFP 3IFQ 3IFR 3IFS 3IFT 3IFU 3IFV 3IFW 3IFZ 3IG0 3IG1 3IG2 3IG3 3IG4 3IG5 3IG6 3IG7 3IG8 3IG9 3IGA 3IGB 3IGD 3IGE 3IGF 3IGG 3IGH 3IGJ 3IGN 3IGO 3IGP 3IGQ 3IGR 3IGS 3IGU 3IGV 3IGX 3IGY 3IGZ 3IH0 3IH2 3IH3 3IH4 3IH5 3IH6 3IH8 3IH9 3IHA 3IHB 3IHC 3IHD 3IHE 3IHF 3IHG 3IHI 3IHJ 3IHK 3IHL 3IHM 3IHO 3IHP 3IHQ 3IHR 3IHS 3IHT 3IHU 3IHV 3IHW 3IHX 3IHY 3IHZ 3II0 3II1 3II2 3II3 3II4 3II5 3II6 3II7 3II9 3IIA 3IIB 3IIC 3IID 3IIE 3IIF 3IIG 3IIH 3III 3IIJ 3IIK 3IIL 3IIM 3IIO 3IIP 3IIQ 3IIR 3IIS 3IIT 3IIU 3IIV 3IIW 3IIX 3IIY 3IIZ 3IJ0 3IJ1 3IJ2 3IJ3 3IJ4 3IJ5 3IJ6 3IJ7 3IJ8 3IJ9 3IJC 3IJD 3IJE 3IJF 3IJG 3IJH 3IJI 3IJJ 3IJL 3IJM 3IJO 3IJP 3IJQ 3IJR 3IJS 3IJT 3IJU 3IJV 3IJW 3IJX 3IJY 3IJZ 3IK0 3IK1 3IK2 3IK3 3IK4 3IK5 3IK6 3IK7 3IK8 3IK9 3IKA 3IKB 3IKC 3IKD 3IKE 3IKF 3IKG 3IKH 3IKJ 3IKK 3IKL 3IKM 3IKN 3IKO 3IKP 3IKQ 3IKR 3IKV 3IKW 3IL0 3IL1 3IL3 3IL4 3IL5 3IL6 3IL7 3IL8 3IL9 3ILA 3ILB 3ILC 3ILD 3ILE 3ILF 3ILG 3ILH 3ILI 3ILJ 3ILK 3ILL 3ILM 3ILN 3ILO 3ILP 3ILQ 3ILR 3ILS 3ILT 3ILU 3ILV 3ILW 3ILX 3ILY 3ILZ 3IM0 3IM1 3IM2 3IM3 3IM4 3IM5 3IM6 3IM7 3IM8 3IM9 3IMA 3IMC 3IMD 3IME 3IMF 3IMG 3IMH 3IMI 3IMJ 3IMK 3IML 3IMM 3IMN 3IMO 3IMP 3IMQ 3IMR 3IMS 3IMT 3IMU 3IMV 3IMW 3IMX 3IMY 3IN0 3IN1 3IN2 3IN3 3IN4 3IN6 3IN7 3IN8 3IN9 3INA 3INB 3INC 3IND 3INE 3INF 3ING 3INH 3INJ 3INK 3INL 3INM 3INN 3INO 3INP 3INQ 3INR 3INT 3INU 3INV 3INW 3INX 3INY 3INZ 3IO0 3IO1 3IO2 3IO3 3IO4 3IO5 3IO6 3IO7 3IO8 3IO9 3IOB 3IOC 3IOD 3IOE 3IOF 3IOG 3IOH 3IOI 3IOJ 3IOK 3IOL 3IOM 3ION 3IOP 3IOQ 3IOR 3IOS 3IOT 3IOU 3IOV 3IOW 3IOX 3IOY 3IOZ 3IP0 3IP1 3IP2 3IP3 3IP4 3IP5 3IP6 3IP7 3IP8 3IP9 3IPA 3IPB 3IPC 3IPD 3IPE 3IPF 3IPH 3IPI 3IPJ 3IPK 3IPL 3IPM 3IPN 3IPO 3IPP 3IPQ 3IPR 3IPS 3IPT 3IPU 3IPV 3IPW 3IPX 3IPY 3IPZ 3IQ0 3IQ1 3IQ2 3IQ3 3IQ5 3IQ6 3IQ7 3IQA 3IQB 3IQC 3IQD 3IQE 3IQF 3IQG 3IQH 3IQI 3IQJ 3IQL 3IQM 3IQO 3IQQ 3IQS 3IQT 3IQU 3IQV 3IQW 3IQX 3IQY 3IQZ 3IR0 3IR1 3IR2 3IR3 3IR4 3IR5 3IR6 3IR7 3IR8 3IR9 3IRA 3IRB 3IRC 3IRD 3IRH 3IRM 3IRN 3IRO 3IRP 3IRS 3IRT 3IRU 3IRV 3IRX 3IRZ 3IS0 3IS1 3IS2 3IS3 3IS4 3IS5 3IS6 3IS7 3IS8 3IS9 3ISA 3ISE 3ISF 3ISG 3ISH 3ISI 3ISJ 3ISL 3ISM 3ISN 3ISO 3ISP 3ISQ 3ISR 3ISS 3IST 3ISU 3ISV 3ISW 3ISX 3ISY 3ISZ 3IT0 3IT1 3IT2 3IT3 3IT4 3IT5 3IT6 3IT7 3IT8 3IT9 3ITA 3ITB 3ITC 3ITD 3ITE 3ITF 3ITG 3ITH 3ITI 3ITJ 3ITK 3ITL 3ITM 3ITN 3ITO 3ITP 3ITQ 3ITT 3ITU 3ITV 3ITW 3ITX 3ITY 3ITZ 3IU0 3IU1 3IU2 3IU3 3IU4 3IU5 3IU6 3IU7 3IU8 3IU9 3IUB 3IUC 3IUD 3IUE 3IUF 3IUG 3IUH 3IUI 3IUJ 3IUK 3IUL 3IUM 3IUN 3IUO 3IUP 3IUQ 3IUR 3IUS 3IUT 3IUU 3IUV 3IUW 3IUX 3IUY 3IUZ 3IV0 3IV1 3IV2 3IV3 3IV4 3IV6 3IV7 3IV8 3IV9 3IVA 3IVB 3IVC 3IVD 3IVE 3IVF 3IVG 3IVH 3IVI 3IVL 3IVM 3IVP 3IVQ 3IVR 3IVS 3IVT 3IVU 3IVV 3IVX 3IVY 3IVZ 3IW0 3IW1 3IW2 3IW3 3IW4 3IW5 3IW6 3IW7 3IW8 3IWA 3IWB 3IWC 3IWD 3IWE 3IWF 3IWG 3IWH 3IWI 3IWJ 3IWK 3IWL 3IWM 3IWO 3IWP 3IWQ 3IWR 3IWT 3IWU 3IWV 3IWW 3IWX 3IWY 3IWZ 3IX0 3IX1 3IX3 3IX4 3IX6 3IX7 3IX8 3IX9 3IXA 3IXB 3IXC 3IXD 3IXE 3IXF 3IXG 3IXH 3IXJ 3IXK 3IXL 3IXM 3IXO 3IXP 3IXQ 3IXR 3IXS 3IXT 3JDW 3JPU 3JPV 3JPW 3JPX 3JPY 3JPZ 3JQ0 3JQ1 3JQ3 3JQ5 3JQ6 3JQ7 3JQ8 3JQ9 3JQA 3JQB 3JQC 3JQD 3JQE 3JQF 3JQG 3JQH 3JQJ 3JQK 3JQL 3JQM 3JQO 3JQP 3JQQ 3JQR 3JQW 3JQX 3JQY 3JQZ 3JR1 3JR2 3JR3 3JR6 3JR7 3JR8 3JRK 3JRM 3JRN 3JRO 3JRP 3JRQ 3JRR 3JRS 3JRT 3JRU 3JRV 3JRW 3JRX 3JRY 3JRZ 3JS1 3JS2 3JS3 3JS4 3JS5 3JS6 3JS8 3JS9 3JSA 3JSB 3JSC 3JSD 3JSE 3JSF 3JSG 3JSI 3JSJ 3JSK 3JSL 3JSN 3JSQ 3JSR 3JSS 3JST 3JSU 3JSV 3JSW 3JSX 3JSY 3JSZ 3JT0 3JT1 3JT2 3JT3 3JT4 3JT5 3JT6 3JT7 3JT8 3JT9 3JTA 3JTB 3JTC 3JTD 3JTE 3JTF 3JTH 3JTI 3JTJ 3JTK 3JTL 3JTM 3JTN 3JTO 3JTP 3JTQ 3JTR 3JTS 3JTT 3JTU 3JTW 3JTX 3JTY 3JTZ 3JU0 3JU1 3JU2 3JU3 3JU4 3JU5 3JU6 3JU7 3JU8 3JU9 3JUA 3JUB 3JUC 3JUD 3JUE 3JUG 3JUH 3JUI 3JUJ 3JUK 3JUL 3JUM 3JUN 3JUO 3JUP 3JUQ 3JUR 3JUS 3JUT 3JUU 3JUV 3JUW 3JUX 3JUY 3JUZ 3JV0 3JV1 3JV2 3JV3 3JV4 3JV5 3JV6 3JV7 3JV9 3JVA 3JVB 3JVC 3JVD 3JVE 3JVF 3JVG 3JVH 3JVI 3JVJ 3JVK 3JVL 3JVM 3JVN 3JVO 3JVR 3JVS 3JVT 3JVU 3JVV 3JVW 3JVX 3JVY 3JVZ 3JW0 3JW1 3JW2 3JW3 3JW4 3JW5 3JW6 3JW7 3JW8 3JW9 3JWA 3JWB 3JWC 3JWD 3JWE 3JWF 3JWG 3JWH 3JWI 3JWJ 3JWK 3JWM 3JWN 3JWO 3JWP 3JWQ 3JWR 3JWS 3JWT 3JWU 3JWV 3JWW 3JWX 3JWY 3JWZ 3JX0 3JX1 3JX2 3JX3 3JX4 3JX5 3JX6 3JX8 3JX9 3JXA 3JXE 3JXF 3JXG 3JXH 3JXI 3JXJ 3JXO 3JXP 3JXS 3JXT 3JXU 3JXV 3JXW 3JY0 3JY6 3JY9 3JYA 3JYB 3JYC 3JYF 3JYG 3JYH 3JYI 3JYJ 3JYL 3JYM 3JYN 3JYO 3JYP 3JYQ 3JYR 3JYS 3JYU 3JYY 3JYZ 3JZ0 3JZ1 3JZ2 3JZ3 3JZ4 3JZ6 3JZ7 3JZ9 3JZA 3JZB 3JZC 3JZD 3JZE 3JZF 3JZG 3JZH 3JZI 3JZJ 3JZK 3JZL 3JZM 3JZN 3JZO 3JZP 3JZQ 3JZR 3JZS 3JZT 3JZU 3JZV 3JZY 3JZZ 3K00 3K01 3K02 3K03 3K04 3K05 3K06 3K07 3K08 3K09 3K0A 3K0B 3K0C 3K0D 3K0E 3K0F 3K0G 3K0H 3K0I 3K0K 3K0L 3K0M 3K0N 3K0O 3K0P 3K0Q 3K0R 3K0T 3K0V 3K0W 3K0X 3K0Y 3K0Z 3K10 3K11 3K12 3K13 3K14 3K15 3K16 3K17 3K19 3K1A 3K1B 3K1D 3K1E 3K1F 3K1G 3K1H 3K1I 3K1J 3K1K 3K1L 3K1M 3K1N 3K1O 3K1P 3K1R 3K1S 3K1T 3K1U 3K1W 3K1X 3K1Y 3K1Z 3K20 3K21 3K22 3K23 3K24 3K25 3K26 3K27 3K28 3K29 3K2A 3K2B 3K2C 3K2D 3K2E 3K2F 3K2G 3K2H 3K2I 3K2J 3K2K 3K2L 3K2M 3K2N 3K2O 3K2P 3K2Q 3K2R 3K2T 3K2U 3K2V 3K2W 3K2X 3K2Y 3K2Z 3K30 3K31 3K32 3K33 3K34 3K35 3K36 3K37 3K38 3K39 3K3A 3K3B 3K3C 3K3D 3K3E 3K3F 3K3G 3K3H 3K3I 3K3J 3K3K 3K3L 3K3N 3K3O 3K3P 3K3Q 3K3S 3K3T 3K3U 3K3V 3K3W 3K40 3K41 3K42 3K43 3K44 3K45 3K46 3K47 3K48 3K4A 3K4B 3K4C 3K4D 3K4F 3K4G 3K4H 3K4I 3K4J 3K4K 3K4L 3K4M 3K4N 3K4O 3K4P 3K4Q 3K4S 3K4T 3K4U 3K4V 3K4W 3K4Y 3K4Z 3K50 3K51 3K52 3K53 3K54 3K55 3K56 3K5B 3K5C 3K5D 3K5E 3K5F 3K5G 3K5H 3K5I 3K5J 3K5K 3K5O 3K5P 3K5R 3K5S 3K5T 3K5U 3K5V 3K5W 3K5X 3K60 3K63 3K65 3K66 3K67 3K69 3K6A 3K6B 3K6C 3K6D 3K6E 3K6F 3K6G 3K6H 3K6I 3K6J 3K6K 3K6L 3K6M 3K6N 3K6O 3K6P 3K6Q 3K6R 3K6S 3K6T 3K6U 3K6V 3K6W 3K6X 3K6Y 3K6Z 3K71 3K72 3K73 3K74 3K75 3K77 3K79 3K7A 3K7B 3K7C 3K7D 3K7E 3K7F 3K7G 3K7H 3K7I 3K7J 3K7K 3K7L 3K7M 3K7N 3K7O 3K7P 3K7Q 3K7R 3K7S 3K7T 3K7U 3K7V 3K7W 3K7X 3K7Y 3K7Z 3K80 3K81 3K82 3K83 3K84 3K85 3K86 3K87 3K88 3K89 3K8A 3K8B 3K8C 3K8D 3K8E 3K8G 3K8H 3K8I 3K8J 3K8K 3K8L 3K8M 3K8N 3K8O 3K8P 3K8Q 3K8R 3K8S 3K8T 3K8U 3K8V 3K8W 3K8X 3K8Y 3K8Z 3K90 3K91 3K92 3K93 3K94 3K96 3K97 3K98 3K99 3K9A 3K9B 3K9C 3K9D 3K9E 3K9G 3K9H 3K9I 3K9J 3K9K 3K9L 3K9M 3K9N 3K9O 3K9P 3K9Q 3K9R 3K9S 3K9T 3K9U 3K9V 3K9W 3K9X 3K9Y 3K9Z 3KA0 3KA2 3KA3 3KA4 3KA5 3KA6 3KA7 3KA8 3KA9 3KAA 3KAB 3KAC 3KAD 3KAE 3KAF 3KAG 3KAH 3KAI 3KAJ 3KAK 3KAL 3KAM 3KAN 3KAO 3KAP 3KAQ 3KAR 3KAS 3KAT 3KAV 3KAW 3KAY 3KAZ 3KB0 3KB1 3KB2 3KB3 3KB4 3KB5 3KB6 3KB7 3KB8 3KB9 3KBA 3KBB 3KBC 3KBE 3KBF 3KBG 3KBH 3KBJ 3KBK 3KBL 3KBM 3KBN 3KBO 3KBP 3KBQ 3KBR 3KBS 3KBT 3KBU 3KBV 3KBW 3KBX 3KBY 3KBZ 3KC0 3KC1 3KC2 3KC3 3KC6 3KCC 3KCE 3KCF 3KCG 3KCH 3KCI 3KCK 3KCM 3KCN 3KCP 3KCQ 3KCS 3KCT 3KCU 3KCV 3KCW 3KCX 3KCY 3KCZ 3KD0 3KD2 3KD3 3KD4 3KD6 3KD7 3KD8 3KD9 3KDA 3KDB 3KDC 3KDD 3KDF 3KDG 3KDH 3KDI 3KDJ 3KDK 3KDM 3KDN 3KDO 3KDP 3KDQ 3KDR 3KDS 3KDT 3KDU 3KDW 3KDY 3KDZ 3KE0 3KE1 3KE2 3KE3 3KE4 3KE5 3KE6 3KE7 3KE8 3KE9 3KEA 3KEB 3KEC 3KED 3KEE 3KEF 3KEG 3KEH 3KEI 3KEJ 3KEK 3KEL 3KEM 3KEN 3KEO 3KEP 3KEQ 3KER 3KES 3KEU 3KEV 3KEW 3KEX 3KEY 3KEZ 3KF0 3KF2 3KF3 3KF4 3KF5 3KF6 3KF7 3KF8 3KF9 3KFA 3KFB 3KFC 3KFD 3KFE 3KFF 3KFG 3KFH 3KFI 3KFJ 3KFK 3KFL 3KFM 3KFN 3KFO 3KFP 3KFQ 3KFR 3KFS 3KFT 3KFV 3KFW 3KFX 3KFY 3KG0 3KG1 3KG2 3KG4 3KG5 3KG6 3KG7 3KG8 3KG9 3KGA 3KGB 3KGC 3KGD 3KGF 3KGG 3KGK 3KGL 3KGP 3KGQ 3KGR 3KGS 3KGT 3KGU 3KGV 3KGW 3KGX 3KGY 3KGZ 3KH0 3KH1 3KH2 3KH3 3KH4 3KH5 3KH7 3KH8 3KH9 3KHB 3KHD 3KHE 3KHF 3KHI 3KHJ 3KHK 3KHM 3KHN 3KHO 3KHP 3KHQ 3KHS 3KHT 3KHU 3KHV 3KHW 3KHX 3KHY 3KHZ 3KI0 3KI1 3KI2 3KI3 3KI4 3KI5 3KI6 3KI7 3KI8 3KI9 3KIA 3KIC 3KID 3KIE 3KIF 3KIG 3KIH 3KII 3KIJ 3KIK 3KIN 3KIO 3KIP 3KIV 3KIZ 3KJ0 3KJ1 3KJ2 3KJ4 3KJ6 3KJ7 3KJD 3KJE 3KJF 3KJG 3KJH 3KJI 3KJJ 3KJK 3KJL 3KJM 3KJN 3KJQ 3KJR 3KJS 3KJT 3KJX 3KJY 3KJZ 3KK0 3KK4 3KK6 3KK7 3KK8 3KK9 3KKA 3KKB 3KKC 3KKD 3KKE 3KKF 3KKG 3KKI 3KKJ 3KKK 3KKL 3KKM 3KKN 3KKO 3KKP 3KKQ 3KKR 3KKS 3KKT 3KKU 3KKV 3KKW 3KKY 3KKZ 3KL0 3KL1 3KL2 3KL3 3KL4 3KL5 3KL6 3KL7 3KL8 3KL9 3KLA 3KLB 3KLC 3KLD 3KLI 3KLJ 3KLK 3KLL 3KLM 3KLN 3KLO 3KLP 3KLQ 3KLR 3KLS 3KLT 3KLU 3KLW 3KLX 3KLY 3KLZ 3KM0 3KM1 3KM2 3KM3 3KM4 3KM5 3KM6 3KM8 3KM9 3KMA 3KMB 3KMC 3KME 3KMG 3KMH 3KMI 3KMJ 3KML 3KMM 3KMN 3KMO 3KMR 3KMT 3KMU 3KMV 3KMW 3KMX 3KMY 3KMZ 3KN0 3KN1 3KN2 3KN3 3KN4 3KN5 3KN6 3KN7 3KN8 3KNB 3KND 3KNE 3KNF 3KNG 3KNP 3KNQ 3KNR 3KNS 3KNU 3KNV 3KNW 3KNX 3KNY 3KNZ 3KO0 3KO1 3KO3 3KO4 3KO5 3KO6 3KO7 3KO8 3KO9 3KOB 3KOC 3KOD 3KOF 3KOG 3KOH 3KOI 3KOJ 3KOK 3KOL 3KOM 3KON 3KOO 3KOP 3KOQ 3KOR 3KOS 3KOT 3KOU 3KOW 3KOX 3KOY 3KOZ 3KP0 3KP1 3KP2 3KP3 3KP4 3KP5 3KP6 3KP7 3KP8 3KP9 3KPA 3KPB 3KPC 3KPD 3KPE 3KPF 3KPH 3KPJ 3KPK 3KPL 3KPM 3KPN 3KPO 3KPP 3KPQ 3KPR 3KPS 3KPT 3KPU 3KPV 3KPW 3KPX 3KPY 3KPZ 3KQ0 3KQ4 3KQ5 3KQ6 3KQ7 3KQA 3KQB 3KQC 3KQD 3KQE 3KQF 3KQG 3KQI 3KQJ 3KQM 3KQO 3KQP 3KQQ 3KQR 3KQS 3KQT 3KQV 3KQW 3KQX 3KQY 3KQZ 3KR0 3KR1 3KR2 3KR3 3KR4 3KR5 3KR6 3KR7 3KR8 3KR9 3KRA 3KRB 3KRC 3KRD 3KRE 3KRF 3KRG 3KRJ 3KRK 3KRL 3KRM 3KRN 3KRO 3KRP 3KRQ 3KRR 3KRS 3KRT 3KRU 3KRV 3KRW 3KRX 3KRY 3KRZ 3KS0 3KS2 3KS3 3KS4 3KS5 3KS6 3KS7 3KS9 3KSC 3KSD 3KSE 3KSF 3KSG 3KSH 3KSI 3KSJ 3KSK 3KSL 3KSM 3KSN 3KSO 3KSP 3KSQ 3KSR 3KSS 3KST 3KSU 3KSV 3KSW 3KSX 3KSY 3KSZ 3KT0 3KT1 3KT2 3KT3 3KT4 3KT5 3KT6 3KT7 3KT8 3KT9 3KTA 3KTB 3KTC 3KTD 3KTF 3KTG 3KTH 3KTI 3KTJ 3KTK 3KTL 3KTM 3KTN 3KTO 3KTP 3KTR 3KTS 3KTX 3KTY 3KTZ 3KU0 3KU1 3KU2 3KU3 3KU4 3KU5 3KU6 3KU7 3KU9 3KUC 3KUD 3KUE 3KUF 3KUG 3KUH 3KUI 3KUJ 3KUK 3KUL 3KUM 3KUN 3KUO 3KUP 3KUQ 3KUR 3KUS 3KUT 3KUU 3KUV 3KUW 3KUX 3KUZ 3KV0 3KV1 3KV2 3KV3 3KV4 3KV5 3KV6 3KV7 3KV8 3KV9 3KVA 3KVB 3KVC 3KVD 3KVE 3KVF 3KVG 3KVH 3KVI 3KVJ 3KVK 3KVL 3KVM 3KVN 3KVO 3KVP 3KVQ 3KVR 3KVS 3KVT 3KVU 3KVV 3KVW 3KVX 3KVY 3KVZ 3KW0 3KW1 3KW2 3KW3 3KW4 3KW5 3KW6 3KW7 3KW8 3KW9 3KWA 3KWB 3KWC 3KWD 3KWE 3KWF 3KWG 3KWI 3KWJ 3KWK 3KWL 3KWM 3KWN 3KWO 3KWP 3KWR 3KWS 3KWT 3KWU 3KWV 3KWW 3KWX 3KWY 3KWZ 3KX0 3KX1 3KX2 3KX3 3KX4 3KX5 3KX6 3KX7 3KX8 3KX9 3KXA 3KXC 3KXD 3KXE 3KXF 3KXG 3KXH 3KXI 3KXK 3KXL 3KXM 3KXN 3KXO 3KXP 3KXQ 3KXR 3KXS 3KXU 3KXV 3KXW 3KXX 3KXY 3KXZ 3KY2 3KY7 3KY8 3KY9 3KYA 3KYB 3KYC 3KYD 3KYE 3KYF 3KYG 3KYH 3KYI 3KYJ 3KYK 3KYM 3KYN 3KYO 3KYP 3KYQ 3KYR 3KYS 3KYT 3KYU 3KYV 3KYW 3KYZ 3KZ0 3KZ1 3KZ3 3KZ4 3KZ5 3KZ7 3KZ9 3KZA 3KZB 3KZC 3KZD 3KZE 3KZF 3KZG 3KZH 3KZI 3KZJ 3KZK 3KZL 3KZM 3KZN 3KZO 3KZP 3KZQ 3KZS 3KZT 3KZU 3KZV 3KZW 3KZX 3KZY 3KZZ 3L00 3L01 3L02 3L03 3L04 3L05 3L06 3L07 3L08 3L09 3L0A 3L0B 3L0C 3L0D 3L0E 3L0F 3L0G 3L0H 3L0I 3L0J 3L0K 3L0L 3L0M 3L0N 3L0O 3L0P 3L0Q 3L0R 3L0S 3L0T 3L0V 3L0W 3L0X 3L0Y 3L0Z 3L10 3L11 3L12 3L13 3L14 3L15 3L16 3L17 3L18 3L19 3L1A 3L1B 3L1C 3L1E 3L1F 3L1G 3L1H 3L1I 3L1J 3L1K 3L1L 3L1M 3L1N 3L1O 3L1R 3L1S 3L1T 3L1U 3L1V 3L1W 3L1X 3L1Y 3L1Z 3L20 3L21 3L22 3L23 3L24 3L27 3L28 3L29 3L2A 3L2B 3L2D 3L2E 3L2H 3L2I 3L2J 3L2K 3L2L 3L2M 3L2N 3L2O 3L2X 3L2Y 3L2Z 3L30 3L31 3L32 3L33 3L34 3L35 3L36 3L37 3L38 3L39 3L3A 3L3B 3L3D 3L3F 3L3G 3L3H 3L3I 3L3J 3L3K 3L3L 3L3M 3L3N 3L3O 3L3P 3L3Q 3L3R 3L3S 3L3T 3L3U 3L3V 3L3X 3L3Z 3L40 3L41 3L42 3L43 3L44 3L46 3L47 3L48 3L49 3L4A 3L4B 3L4C 3L4D 3L4E 3L4F 3L4G 3L4H 3L4I 3L4L 3L4M 3L4N 3L4O 3L4P 3L4Q 3L4R 3L4S 3L4T 3L4U 3L4V 3L4W 3L4X 3L4Y 3L4Z 3L50 3L51 3L54 3L56 3L57 3L58 3L59 3L5A 3L5B 3L5C 3L5D 3L5E 3L5F 3L5H 3L5I 3L5J 3L5K 3L5L 3L5M 3L5N 3L5O 3L5P 3L5R 3L5S 3L5T 3L5U 3L5V 3L5W 3L5X 3L5Z 3L60 3L61 3L62 3L63 3L64 3L65 3L66 3L67 3L68 3L6A 3L6B 3L6C 3L6D 3L6E 3L6F 3L6G 3L6H 3L6I 3L6J 3L6N 3L6O 3L6P 3L6Q 3L6R 3L6T 3L6U 3L6V 3L6W 3L6X 3L6Y 3L70 3L71 3L72 3L73 3L74 3L75 3L76 3L77 3L78 3L79 3L7A 3L7B 3L7C 3L7D 3L7E 3L7F 3L7G 3L7H 3L7I 3L7J 3L7K 3L7L 3L7M 3L7N 3L7O 3L7P 3L7Q 3L7R 3L7T 3L7U 3L7V 3L7W 3L7X 3L7Y 3L7Z 3L81 3L82 3L84 3L85 3L86 3L87 3L88 3L89 3L8A 3L8C 3L8D 3L8E 3L8F 3L8G 3L8H 3L8I 3L8J 3L8K 3L8L 3L8M 3L8N 3L8P 3L8Q 3L8R 3L8S 3L8U 3L8V 3L8W 3L8X 3L8Y 3L8Z 3L91 3L92 3L93 3L94 3L95 3L9A 3L9B 3L9C 3L9D 3L9E 3L9F 3L9G 3L9H 3L9I 3L9J 3L9K 3L9L 3L9M 3L9N 3L9P 3L9Q 3L9R 3L9S 3L9T 3L9U 3L9V 3L9W 3L9X 3L9Y 3L9Z 3LA0 3LA1 3LA2 3LA3 3LA4 3LA6 3LA7 3LA8 3LA9 3LAA 3LAC 3LAD 3LAE 3LAF 3LAG 3LAH 3LAI 3LAK 3LAL 3LAM 3LAN 3LAO 3LAQ 3LAR 3LAS 3LAT 3LAU 3LAW 3LAX 3LAY 3LAZ 3LB0 3LB1 3LB2 3LB3 3LB4 3LB5 3LB6 3LB8 3LB9 3LBA 3LBB 3LBC 3LBD 3LBE 3LBF 3LBG 3LBH 3LBI 3LBJ 3LBK 3LBL 3LBM 3LBN 3LBO 3LBS 3LBW 3LBX 3LBY 3LBZ 3LC0 3LC1 3LC2 3LC3 3LC4 3LC5 3LC6 3LC7 3LC8 3LC9 3LCA 3LCB 3LCC 3LCD 3LCE 3LCF 3LCG 3LCH 3LCI 3LCJ 3LCK 3LCL 3LCM 3LCN 3LCO 3LCP 3LCR 3LCS 3LCT 3LCU 3LCV 3LCW 3LCX 3LCY 3LCZ 3LD0 3LD1 3LD2 3LD3 3LD4 3LD5 3LD6 3LD7 3LD8 3LD9 3LDA 3LDB 3LDC 3LDD 3LDE 3LDF 3LDG 3LDH 3LDI 3LDJ 3LDK 3LDL 3LDM 3LDN 3LDO 3LDP 3LDQ 3LDR 3LDT 3LDU 3LDV 3LDW 3LDX 3LDZ 3LE0 3LE1 3LE2 3LE3 3LE4 3LE5 3LE6 3LE7 3LE8 3LE9 3LEA 3LEC 3LED 3LEE 3LEF 3LEG 3LEH 3LEI 3LEK 3LEM 3LEN 3LEO 3LEP 3LEQ 3LER 3LES 3LET 3LEV 3LEW 3LEX 3LEY 3LEZ 3LF0 3LF1 3LF2 3LF3 3LF4 3LF5 3LF6 3LF7 3LF9 3LFA 3LFB 3LFC 3LFD 3LFE 3LFF 3LFG 3LFH 3LFI 3LFJ 3LFK 3LFL 3LFM 3LFN 3LFO 3LFP 3LFQ 3LFR 3LFS 3LFT 3LFU 3LFV 3LFY 3LFZ 3LG0 3LG1 3LG2 3LG3 3LG4 3LG5 3LG6 3LG7 3LG8 3LGA 3LGB 3LGC 3LGD 3LGE 3LGF 3LGG 3LGH 3LGI 3LGJ 3LGK 3LGL 3LGM 3LGN 3LGO 3LGP 3LGQ 3LGR 3LGS 3LGT 3LGU 3LGV 3LGW 3LGX 3LGY 3LGZ 3LH0 3LH1 3LH2 3LH3 3LH4 3LH5 3LH8 3LH9 3LHA 3LHB 3LHC 3LHD 3LHE 3LHF 3LHG 3LHH 3LHI 3LHJ 3LHK 3LHL 3LHM 3LHN 3LHO 3LHP 3LHQ 3LHR 3LHS 3LHT 3LHU 3LHV 3LHW 3LHX 3LHY 3LHZ 3LI0 3LI1 3LI2 3LI3 3LI4 3LI5 3LI6 3LI8 3LI9 3LIA 3LIB 3LIC 3LID 3LIE 3LIF 3LIG 3LIH 3LII 3LIJ 3LIK 3LIL 3LIM 3LIN 3LIO 3LIP 3LIQ 3LIR 3LIS 3LIT 3LIU 3LIV 3LIW 3LIX 3LIY 3LIZ 3LJ0 3LJ1 3LJ2 3LJ3 3LJ5 3LJ6 3LJ7 3LJ8 3LJ9 3LJB 3LJC 3LJD 3LJE 3LJF 3LJG 3LJI 3LJJ 3LJK 3LJL 3LJM 3LJN 3LJO 3LJP 3LJQ 3LJR 3LJS 3LJT 3LJU 3LJW 3LJX 3LJY 3LJZ 3LK0 3LK1 3LK2 3LK3 3LK4 3LK5 3LK6 3LK7 3LK8 3LKA 3LKB 3LKD 3LKE 3LKF 3LKH 3LKI 3LKJ 3LKK 3LKL 3LKM 3LKN 3LKO 3LKP 3LKQ 3LKR 3LKS 3LKT 3LKU 3LKV 3LKW 3LKX 3LKY 3LKZ 3LL0 3LL1 3LL2 3LL3 3LL4 3LL5 3LL7 3LL8 3LL9 3LLA 3LLB 3LLC 3LLD 3LLE 3LLF 3LLH 3LLI 3LLK 3LLL 3LLM 3LLO 3LLP 3LLQ 3LLR 3LLS 3LLT 3LLU 3LLV 3LLW 3LLX 3LLY 3LLZ 3LM0 3LM1 3LM2 3LM3 3LM4 3LM5 3LM6 3LM7 3LM8 3LM9 3LMA 3LMB 3LMC 3LMD 3LME 3LMF 3LMG 3LMH 3LMI 3LMJ 3LMK 3LML 3LMM 3LMN 3LMO 3LMP 3LMS 3LMT 3LMU 3LMV 3LMW 3LMX 3LMY 3LMZ 3LN0 3LN1 3LN2 3LN3 3LN4 3LN5 3LN6 3LN7 3LN8 3LN9 3LNB 3LNC 3LND 3LNE 3LNF 3LNG 3LNH 3LNI 3LNJ 3LNK 3LNL 3LNM 3LNN 3LNO 3LNP 3LNR 3LNS 3LNT 3LNU 3LNV 3LNW 3LNX 3LNY 3LNZ 3LO0 3LO1 3LO2 3LO3 3LO4 3LO5 3LO6 3LO7 3LO8 3LO9 3LOC 3LOD 3LOE 3LOF 3LOG 3LOH 3LOI 3LOJ 3LOK 3LOM 3LON 3LOO 3LOP 3LOQ 3LOR 3LOT 3LOU 3LOV 3LOW 3LOX 3LOY 3LOZ 3LP0 3LP1 3LP2 3LP3 3LP4 3LP5 3LP6 3LP7 3LP8 3LP9 3LPA 3LPB 3LPC 3LPD 3LPE 3LPF 3LPG 3LPH 3LPI 3LPJ 3LPK 3LPL 3LPM 3LPN 3LPO 3LPP 3LPQ 3LPR 3LPS 3LPT 3LPU 3LPW 3LPX 3LPY 3LPZ 3LQ0 3LQ1 3LQ2 3LQ3 3LQ4 3LQ5 3LQ6 3LQ7 3LQ8 3LQ9 3LQA 3LQB 3LQC 3LQD 3LQE 3LQF 3LQG 3LQH 3LQI 3LQJ 3LQK 3LQL 3LQM 3LQN 3LQQ 3LQR 3LQS 3LQU 3LQV 3LQW 3LQY 3LQZ 3LR0 3LR1 3LR2 3LR3 3LR4 3LR5 3LR6 3LR7 3LR8 3LR9 3LRA 3LRB 3LRC 3LRD 3LRE 3LRF 3LRG 3LRH 3LRJ 3LRK 3LRL 3LRM 3LRP 3LRQ 3LRS 3LRT 3LRU 3LRV 3LRX 3LRY 3LS0 3LS1 3LS2 3LS3 3LS4 3LS5 3LS6 3LS7 3LS8 3LS9 3LSA 3LSB 3LSC 3LSD 3LSE 3LSF 3LSG 3LSH 3LSI 3LSJ 3LSK 3LSL 3LSM 3LSN 3LSO 3LSQ 3LSS 3LST 3LSU 3LSV 3LSW 3LSX 3LSY 3LSZ 3LT0 3LT1 3LT2 3LT3 3LT4 3LT5 3LT6 3LT7 3LT8 3LT9 3LTA 3LTB 3LTC 3LTD 3LTE 3LTF 3LTG 3LTH 3LTI 3LTJ 3LTL 3LTM 3LTO 3LTP 3LTQ 3LTS 3LTV 3LTW 3LTY 3LU1 3LU2 3LU6 3LU7 3LU8 3LU9 3LUA 3LUB 3LUC 3LUD 3LUF 3LUG 3LUH 3LUI 3LUJ 3LUK 3LUL 3LUM 3LUN 3LUO 3LUP 3LUQ 3LUR 3LUS 3LUT 3LUU 3LUY 3LUZ 3LV0 3LV1 3LV2 3LV3 3LV4 3LV5 3LV6 3LV8 3LV9 3LVA 3LVB 3LVC 3LVD 3LVE 3LVF 3LVG 3LVH 3LVJ 3LVK 3LVL 3LVM 3LVP 3LVQ 3LVR 3LVS 3LVT 3LVU 3LVV 3LVW 3LVX 3LVY 3LVZ 3LW0 3LW1 3LW2 3LW3 3LW5 3LW6 3LW7 3LW8 3LW9 3LWA 3LWB 3LWC 3LWD 3LWE 3LWF 3LWG 3LWJ 3LWK 3LWN 3LWS 3LWT 3LWU 3LWW 3LWX 3LWZ 3LX0 3LX1 3LX2 3LX3 3LX4 3LX5 3LX6 3LX7 3LX8 3LX9 3LXA 3LXB 3LXC 3LXD 3LXE 3LXF 3LXG 3LXH 3LXI 3LXJ 3LXK 3LXL 3LXM 3LXN 3LXO 3LXP 3LXQ 3LXR 3LXS 3LXT 3LXU 3LXV 3LXX 3LXY 3LXZ 3LY0 3LY1 3LY2 3LY3 3LY4 3LY5 3LY6 3LY7 3LY8 3LY9 3LYA 3LYB 3LYC 3LYD 3LYE 3LYF 3LYG 3LYH 3LYI 3LYK 3LYL 3LYM 3LYN 3LYO 3LYP 3LYQ 3LYR 3LYS 3LYT 3LYU 3LYV 3LYW 3LYX 3LYY 3LYZ 3LZ2 3LZ3 3LZ5 3LZ6 3LZ7 3LZ8 3LZ9 3LZA 3LZB 3LZC 3LZD 3LZE 3LZF 3LZG 3LZK 3LZL 3LZM 3LZN 3LZO 3LZP 3LZQ 3LZR 3LZS 3LZT 3LZU 3LZV 3LZW 3LZX 3LZY 3LZZ 3M00 3M01 3M02 3M03 3M04 3M05 3M06 3M07 3M08 3M09 3M0A 3M0B 3M0C 3M0D 3M0E 3M0F 3M0G 3M0H 3M0I 3M0J 3M0K 3M0L 3M0M 3M0N 3M0O 3M0P 3M0Q 3M0R 3M0S 3M0T 3M0U 3M0V 3M0W 3M0X 3M0Y 3M0Z 3M10 3M11 3M12 3M13 3M14 3M15 3M16 3M17 3M18 3M19 3M1A 3M1B 3M1C 3M1D 3M1E 3M1F 3M1G 3M1H 3M1I 3M1J 3M1K 3M1L 3M1M 3M1N 3M1O 3M1P 3M1Q 3M1R 3M1S 3M1T 3M1U 3M1V 3M1W 3M1X 3M1Y 3M1Z 3M20 3M21 3M22 3M23 3M24 3M25 3M26 3M27 3M28 3M29 3M2A 3M2B 3M2C 3M2D 3M2E 3M2F 3M2G 3M2H 3M2I 3M2J 3M2K 3M2L 3M2M 3M2N 3M2P 3M2R 3M2T 3M2U 3M2V 3M2W 3M2X 3M2Y 3M2Z 3M30 3M31 3M32 3M33 3M34 3M35 3M36 3M37 3M38 3M39 3M3A 3M3B 3M3C 3M3D 3M3E 3M3F 3M3G 3M3H 3M3I 3M3J 3M3K 3M3L 3M3M 3M3N 3M3O 3M3P 3M3Q 3M3R 3M3S 3M3T 3M3U 3M3V 3M3W 3M3X 3M3Z 3M40 3M41 3M42 3M43 3M44 3M45 3M46 3M47 3M48 3M49 3M4B 3M4C 3M4D 3M4E 3M4F 3M4G 3M4H 3M4I 3M4J 3M4N 3M4P 3M4Q 3M4R 3M4S 3M4T 3M4U 3M4V 3M4W 3M4X 3M4Y 3M4Z 3M50 3M51 3M52 3M53 3M54 3M55 3M56 3M57 3M58 3M59 3M5A 3M5B 3M5C 3M5D 3M5E 3M5G 3M5H 3M5I 3M5J 3M5K 3M5L 3M5M 3M5N 3M5O 3M5P 3M5Q 3M5R 3M5S 3M5T 3M5U 3M5V 3M5W 3M5X 3M5Y 3M5Z 3M61 3M62 3M63 3M64 3M65 3M66 3M67 3M6A 3M6B 3M6C 3M6D 3M6E 3M6F 3M6G 3M6H 3M6I 3M6J 3M6K 3M6L 3M6M 3M6N 3M6O 3M6P 3M6Q 3M6R 3M6S 3M6U 3M6V 3M6W 3M6X 3M6Y 3M6Z 3M70 3M71 3M72 3M73 3M74 3M75 3M76 3M77 3M78 3M79 3M7A 3M7B 3M7C 3M7D 3M7E 3M7F 3M7G 3M7H 3M7I 3M7J 3M7L 3M7M 3M7O 3M7P 3M7Q 3M7R 3M7S 3M7T 3M7U 3M7V 3M7W 3M81 3M82 3M83 3M84 3M86 3M88 3M89 3M8A 3M8B 3M8C 3M8D 3M8E 3M8F 3M8J 3M8K 3M8L 3M8M 3M8N 3M8O 3M8P 3M8Q 3M8T 3M8U 3M8V 3M8W 3M8Y 3M8Z 3M91 3M92 3M93 3M94 3M95 3M96 3M97 3M98 3M99 3M9A 3M9B 3M9C 3M9D 3M9F 3M9G 3M9H 3M9J 3M9K 3M9L 3M9Q 3M9S 3M9U 3M9V 3M9W 3M9X 3M9Y 3M9Z 3MA0 3MA2 3MA3 3MA5 3MA6 3MA7 3MA8 3MA9 3MAA 3MAB 3MAC 3MAD 3MAE 3MAF 3MAG 3MAH 3MAJ 3MAK 3MAL 3MAM 3MAN 3MAO 3MAP 3MAR 3MAS 3MAT 3MAU 3MAV 3MAW 3MAX 3MAY 3MAZ 3MB2 3MB3 3MB4 3MB5 3MB6 3MB7 3MB8 3MB9 3MBA 3MBB 3MBC 3MBD 3MBE 3MBF 3MBG 3MBH 3MBI 3MBJ 3MBK 3MBL 3MBM 3MBO 3MBP 3MBQ 3MBR 3MBT 3MBU 3MBV 3MBW 3MBX 3MBZ 3MC0 3MC1 3MC2 3MC3 3MC4 3MC5 3MC6 3MC8 3MC9 3MCA 3MCB 3MCD 3MCE 3MCF 3MCG 3MCH 3MCI 3MCJ 3MCK 3MCL 3MCM 3MCN 3MCO 3MCP 3MCQ 3MCR 3MCS 3MCT 3MCU 3MCV 3MCW 3MCX 3MCY 3MCZ 3MD0 3MD1 3MD2 3MD3 3MD4 3MD5 3MD7 3MD9 3MDB 3MDD 3MDE 3MDF 3MDJ 3MDK 3MDL 3MDM 3MDN 3MDO 3MDP 3MDQ 3MDR 3MDS 3MDT 3MDU 3MDV 3MDW 3MDX 3MDY 3MDZ 3ME0 3ME1 3ME2 3ME3 3ME4 3ME5 3ME6 3ME7 3ME8 3ME9 3MEA 3MEB 3MEC 3MED 3MEE 3MEG 3MEH 3MEJ 3MEK 3MEL 3MEM 3MEN 3MEP 3MEQ 3MER 3MES 3MET 3MEU 3MEV 3MEW 3MEX 3MEY 3MEZ 3MF0 3MF1 3MF2 3MF3 3MF4 3MF5 3MF6 3MF7 3MF8 3MF9 3MFA 3MFB 3MFC 3MFD 3MFE 3MFF 3MFG 3MFJ 3MFL 3MFM 3MFN 3MFQ 3MFR 3MFS 3MFT 3MFU 3MFV 3MFW 3MFX 3MFY 3MG0 3MG1 3MG2 3MG3 3MG4 3MG5 3MG6 3MG7 3MG8 3MG9 3MGA 3MGB 3MGC 3MGD 3MGE 3MGF 3MGG 3MGJ 3MGK 3MGL 3MGM 3MGN 3MGO 3MGT 3MGU 3MGW 3MGX 3MGY 3MGZ 3MH0 3MH1 3MH2 3MH3 3MH4 3MH5 3MH6 3MH7 3MH8 3MH9 3MHA 3MHB 3MHC 3MHD 3MHE 3MHF 3MHG 3MHH 3MHI 3MHJ 3MHK 3MHL 3MHM 3MHO 3MHP 3MHR 3MHS 3MHU 3MHV 3MHW 3MHX 3MHY 3MHZ 3MI0 3MI1 3MI2 3MI3 3MI4 3MI5 3MI6 3MI7 3MI8 3MI9 3MIA 3MIB 3MIC 3MID 3MIE 3MIF 3MIG 3MIH 3MII 3MIL 3MIM 3MIN 3MIO 3MIT 3MIU 3MIV 3MIW 3MIX 3MIY 3MIZ 3MJ1 3MJ2 3MJ4 3MJ5 3MJ6 3MJ7 3MJ8 3MJ9 3MJC 3MJD 3MJE 3MJF 3MJG 3MJH 3MJI 3MJK 3MJL 3MJM 3MJN 3MJO 3MJP 3MJQ 3MJR 3MJS 3MJT 3MJU 3MJV 3MJW 3MJX 3MJY 3MJZ 3MK0 3MK1 3MK2 3MK3 3MK4 3MK5 3MK6 3MK7 3MK8 3MK9 3MKA 3MKB 3MKC 3MKD 3MKE 3MKF 3MKG 3MKH 3MKI 3MKJ 3MKK 3MKL 3MKM 3MKN 3MKO 3MKP 3MKQ 3MKR 3MKS 3MKT 3MKU 3MKV 3ML0 3ML1 3ML2 3ML3 3ML4 3ML5 3ML6 3ML8 3ML9 3MLA 3MLB 3MLC 3MLE 3MLF 3MLG 3MLH 3MLI 3MLJ 3MLK 3MLL 3MLM 3MLQ 3MLR 3MLS 3MLT 3MLU 3MLV 3MLW 3MLX 3MLY 3MLZ 3MM0 3MM1 3MM2 3MM3 3MM4 3MM5 3MM6 3MM7 3MM8 3MM9 3MMA 3MMB 3MMC 3MMD 3MME 3MMF 3MMG 3MMH 3MMI 3MMJ 3MMK 3MML 3MMN 3MMO 3MMP 3MMR 3MMS 3MMT 3MMU 3MMV 3MMW 3MMX 3MMY 3MMZ 3MN0 3MN1 3MN2 3MN3 3MN5 3MN6 3MN7 3MN8 3MN9 3MNA 3MNB 3MNC 3MND 3MNE 3MNF 3MNG 3MNH 3MNI 3MNJ 3MNK 3MNL 3MNM 3MNO 3MNP 3MNQ 3MNR 3MNS 3MNU 3MNV 3MNW 3MNX 3MNZ 3MO0 3MO1 3MO2 3MO3 3MO4 3MO5 3MO6 3MO7 3MO8 3MO9 3MOA 3MOB 3MOC 3MOD 3MOE 3MOF 3MOG 3MOH 3MOI 3MOK 3MOL 3MOM 3MON 3MOO 3MOP 3MOQ 3MOR 3MOS 3MOU 3MOW 3MOY 3MOZ 3MP1 3MP2 3MP3 3MP4 3MP5 3MP6 3MP7 3MP8 3MP9 3MPA 3MPB 3MPC 3MPD 3MPE 3MPF 3MPG 3MPH 3MPI 3MPJ 3MPK 3MPL 3MPM 3MPN 3MPO 3MPP 3MPQ 3MPR 3MPS 3MPT 3MPU 3MPV 3MPW 3MPX 3MPY 3MPZ 3MQ0 3MQ1 3MQ2 3MQ3 3MQ4 3MQ7 3MQ9 3MQB 3MQC 3MQD 3MQE 3MQF 3MQG 3MQH 3MQI 3MQL 3MQM 3MQO 3MQP 3MQQ 3MQR 3MQS 3MQT 3MQW 3MQZ 3MR0 3MR1 3MR7 3MR9 3MRB 3MRC 3MRD 3MRE 3MRF 3MRG 3MRH 3MRI 3MRJ 3MRK 3MRL 3MRM 3MRN 3MRO 3MRP 3MRQ 3MRR 3MRS 3MRT 3MRU 3MRV 3MRW 3MRX 3MRY 3MS2 3MS3 3MS4 3MS5 3MS6 3MS7 3MS8 3MS9 3MSA 3MSC 3MSD 3MSE 3MSF 3MSG 3MSH 3MSI 3MSJ 3MSK 3MSL 3MSN 3MSO 3MSQ 3MSR 3MSS 3MST 3MSU 3MSV 3MSW 3MSX 3MSY 3MSZ 3MT0 3MT1 3MT5 3MT6 3MT7 3MT8 3MT9 3MTA 3MTB 3MTC 3MTD 3MTE 3MTF 3MTG 3MTH 3MTI 3MTJ 3MTK 3MTL 3MTN 3MTQ 3MTR 3MTS 3MTT 3MTU 3MTV 3MTW 3MTX 3MTY 3MU0 3MU1 3MU3 3MU4 3MU5 3MU7 3MU8 3MUA 3MUC 3MUD 3MUE 3MUF 3MUG 3MUH 3MUI 3MUJ 3MUK 3MUL 3MUN 3MUO 3MUP 3MUQ 3MUS 3MUU 3MUX 3MUY 3MUZ 3MV0 3MV1 3MV2 3MV3 3MV4 3MV5 3MV6 3MV7 3MV8 3MV9 3MVC 3MVE 3MVF 3MVG 3MVH 3MVI 3MVJ 3MVK 3MVL 3MVM 3MVN 3MVO 3MVP 3MVQ 3MVR 3MVS 3MVT 3MVU 3MVV 3MVW 3MVX 3MVY 3MVZ 3MW0 3MW1 3MW2 3MW3 3MW4 3MW6 3MW7 3MW8 3MW9 3MWA 3MWB 3MWC 3MWD 3MWE 3MWF 3MWG 3MWH 3MWI 3MWJ 3MWK 3MWL 3MWM 3MWN 3MWO 3MWP 3MWQ 3MWR 3MWS 3MWT 3MWU 3MWV 3MWW 3MWX 3MWY 3MWZ 3MX0 3MX1 3MX2 3MX3 3MX5 3MX6 3MX7 3MX8 3MXC 3MXD 3MXE 3MXF 3MXG 3MXJ 3MXL 3MXN 3MXO 3MXP 3MXQ 3MXR 3MXS 3MXT 3MXU 3MXV 3MXW 3MXX 3MXY 3MXZ 3MY0 3MY1 3MY2 3MY5 3MY6 3MY7 3MY9 3MYA 3MYB 3MYC 3MYD 3MYE 3MYF 3MYG 3MYH 3MYI 3MYJ 3MYK 3MYL 3MYM 3MYN 3MYO 3MYP 3MYQ 3MYR 3MYT 3MYU 3MYV 3MYW 3MYX 3MYY 3MYZ 3MZ0 3MZ1 3MZ2 3MZ3 3MZ4 3MZ5 3MZ6 3MZ7 3MZ8 3MZ9 3MZB 3MZC 3MZD 3MZE 3MZF 3MZG 3MZI 3MZK 3MZL 3MZN 3MZO 3MZQ 3MZR 3MZS 3MZT 3MZV 3MZW 3MZY 3MZZ 3N00 3N01 3N02 3N03 3N04 3N05 3N06 3N07 3N08 3N0A 3N0B 3N0C 3N0D 3N0E 3N0F 3N0G 3N0H 3N0I 3N0K 3N0L 3N0M 3N0N 3N0P 3N0Q 3N0R 3N0S 3N0T 3N0U 3N0V 3N0W 3N0X 3N0Y 3N0Z 3N10 3N11 3N12 3N13 3N14 3N15 3N17 3N18 3N19 3N1A 3N1B 3N1C 3N1D 3N1E 3N1F 3N1G 3N1H 3N1M 3N1N 3N1O 3N1P 3N1Q 3N1R 3N1S 3N1T 3N1U 3N1V 3N1W 3N1X 3N1Y 3N1Z 3N20 3N21 3N23 3N24 3N25 3N26 3N27 3N28 3N29 3N2A 3N2B 3N2C 3N2D 3N2E 3N2G 3N2I 3N2J 3N2K 3N2L 3N2M 3N2N 3N2O 3N2P 3N2Q 3N2R 3N2S 3N2T 3N2U 3N2V 3N2W 3N2X 3N2Y 3N2Z 3N30 3N31 3N32 3N33 3N34 3N35 3N36 3N37 3N38 3N39 3N3A 3N3B 3N3C 3N3D 3N3E 3N3F 3N3G 3N3H 3N3I 3N3J 3N3K 3N3L 3N3M 3N3N 3N3O 3N3P 3N3Q 3N3R 3N3S 3N3T 3N3U 3N3W 3N3X 3N3Y 3N3Z 3N40 3N41 3N42 3N43 3N44 3N45 3N46 3N49 3N4A 3N4B 3N4C 3N4D 3N4E 3N4F 3N4G 3N4H 3N4I 3N4J 3N4K 3N4L 3N4P 3N4Q 3N4R 3N4S 3N4T 3N4U 3N4V 3N4W 3N4X 3N4Y 3N4Z 3N50 3N51 3N52 3N53 3N54 3N55 3N56 3N57 3N58 3N59 3N5A 3N5B 3N5C 3N5D 3N5E 3N5F 3N5G 3N5H 3N5I 3N5J 3N5K 3N5L 3N5M 3N5N 3N5O 3N5P 3N5Q 3N5R 3N5S 3N5T 3N5U 3N5V 3N5W 3N5X 3N5Y 3N5Z 3N60 3N61 3N62 3N63 3N64 3N65 3N66 3N67 3N68 3N69 3N6A 3N6B 3N6C 3N6D 3N6E 3N6F 3N6G 3N6H 3N6I 3N6J 3N6K 3N6L 3N6M 3N6N 3N6O 3N6Q 3N6R 3N6T 3N6U 3N6V 3N6W 3N6X 3N6Y 3N6Z 3N70 3N71 3N72 3N73 3N74 3N75 3N76 3N77 3N79 3N7A 3N7C 3N7D 3N7E 3N7H 3N7J 3N7K 3N7L 3N7M 3N7N 3N7O 3N7P 3N7R 3N7S 3N7T 3N7U 3N7W 3N7X 3N7Y 3N7Z 3N80 3N81 3N82 3N83 3N84 3N85 3N86 3N87 3N89 3N8B 3N8D 3N8E 3N8F 3N8G 3N8H 3N8I 3N8K 3N8L 3N8M 3N8N 3N8R 3N8S 3N8T 3N8U 3N8V 3N8W 3N8X 3N8Y 3N8Z 3N90 3N91 3N92 3N93 3N94 3N95 3N96 3N98 3N99 3N9A 3N9B 3N9C 3N9D 3N9E 3N9G 3N9H 3N9I 3N9J 3N9K 3N9L 3N9M 3N9N 3N9O 3N9P 3N9Q 3N9R 3N9S 3N9T 3N9U 3N9V 3N9W 3N9X 3N9Y 3N9Z 3NA0 3NA1 3NA2 3NA4 3NA5 3NA6 3NA7 3NA8 3NA9 3NAA 3NAB 3NAC 3NAD 3NAF 3NAG 3NAH 3NAI 3NAK 3NAL 3NAM 3NAN 3NAP 3NAQ 3NAR 3NAS 3NAT 3NAU 3NAV 3NAW 3NAX 3NAY 3NAZ 3NB0 3NB2 3NB5 3NB6 3NB7 3NB8 3NB9 3NBA 3NBB 3NBC 3NBD 3NBE 3NBF 3NBH 3NBI 3NBJ 3NBK 3NBL 3NBM 3NBP 3NBQ 3NBR 3NBS 3NBT 3NBU 3NBV 3NBW 3NBX 3NBY 3NBZ 3NC0 3NC1 3NC2 3NC3 3NC4 3NC5 3NC6 3NC7 3NC8 3NC9 3NCA 3NCB 3NCC 3NCE 3NCF 3NCG 3NCH 3NCJ 3NCK 3NCL 3NCO 3NCP 3NCQ 3NCR 3NCT 3NCV 3NCW 3NCX 3NCY 3NCZ 3ND0 3ND1 3ND2 3ND5 3ND6 3ND7 3ND8 3ND9 3NDA 3NDC 3NDD 3NDE 3NDF 3NDG 3NDI 3NDJ 3NDM 3NDN 3NDO 3NDP 3NDQ 3NDR 3NDS 3NDT 3NDU 3NDV 3NDW 3NDX 3NDY 3NDZ 3NE0 3NE1 3NE2 3NE3 3NE4 3NE5 3NE7 3NE8 3NE9 3NEA 3NEC 3NED 3NEE 3NEF 3NEG 3NEH 3NEI 3NEJ 3NEK 3NEL 3NEM 3NEN 3NEO 3NEP 3NEQ 3NER 3NES 3NET 3NEU 3NEV 3NEW 3NEX 3NEY 3NEZ 3NF0 3NF1 3NF2 3NF3 3NF4 3NF5 3NF6 3NF7 3NF8 3NF9 3NFA 3NFB 3NFC 3NFD 3NFE 3NFF 3NFG 3NFH 3NFI 3NFK 3NFL 3NFM 3NFN 3NFP 3NFQ 3NFR 3NFS 3NFT 3NFU 3NFV 3NFW 3NFY 3NFZ 3NG0 3NG1 3NG2 3NG3 3NG4 3NG5 3NG6 3NG7 3NG8 3NG9 3NGA 3NGB 3NGC 3NGF 3NGG 3NGH 3NGJ 3NGK 3NGL 3NGM 3NGN 3NGP 3NGQ 3NGR 3NGS 3NGT 3NGU 3NGV 3NGW 3NGX 3NGY 3NH3 3NH4 3NH5 3NH6 3NH7 3NH8 3NH9 3NHA 3NHB 3NHC 3NHD 3NHE 3NHF 3NHH 3NHI 3NHJ 3NHK 3NHL 3NHM 3NHN 3NHO 3NHP 3NHQ 3NHR 3NHS 3NHT 3NHU 3NHV 3NHW 3NHX 3NHY 3NHZ 3NI0 3NI2 3NI3 3NI5 3NI6 3NI7 3NI8 3NI9 3NIA 3NIB 3NID 3NIE 3NIF 3NIG 3NIH 3NII 3NIJ 3NIK 3NIL 3NIM 3NIN 3NIO 3NIP 3NIQ 3NIR 3NIS 3NIT 3NIU 3NIV 3NIW 3NIX 3NIY 3NIZ 3NJ0 3NJ1 3NJ2 3NJ3 3NJ4 3NJ5 3NJ8 3NJ9 3NJA 3NJB 3NJC 3NJD 3NJE 3NJF 3NJG 3NJH 3NJI 3NJJ 3NJK 3NJL 3NJM 3NJN 3NJO 3NJP 3NJQ 3NJR 3NJS 3NJT 3NJU 3NJV 3NJW 3NJX 3NJY 3NJZ 3NK0 3NK1 3NK2 3NK3 3NK4 3NK5 3NK6 3NK7 3NK8 3NK9 3NKA 3NKC 3NKD 3NKE 3NKF 3NKG 3NKH 3NKJ 3NKK 3NKL 3NKM 3NKN 3NKO 3NKP 3NKQ 3NKR 3NKS 3NKT 3NKU 3NKV 3NKX 3NKY 3NKZ 3NL1 3NL2 3NL3 3NL5 3NL6 3NL7 3NL9 3NLB 3NLC 3NLD 3NLE 3NLF 3NLG 3NLH 3NLI 3NLJ 3NLK 3NLL 3NLM 3NLN 3NLO 3NLP 3NLQ 3NLR 3NLS 3NLT 3NLU 3NLV 3NLW 3NLX 3NLY 3NLZ 3NM0 3NM1 3NM2 3NM3 3NM4 3NM5 3NM6 3NM7 3NM8 3NMB 3NMD 3NME 3NMH 3NMI 3NMJ 3NMK 3NML 3NMM 3NMN 3NMO 3NMP 3NMQ 3NMS 3NMT 3NMV 3NMW 3NMX 3NMZ 3NN0 3NN1 3NN2 3NN3 3NN4 3NN6 3NN7 3NN8 3NN9 3NNB 3NND 3NNE 3NNF 3NNG 3NNJ 3NNK 3NNL 3NNM 3NNN 3NNO 3NNQ 3NNR 3NNS 3NNT 3NNU 3NNV 3NNW 3NNX 3NNY 3NNZ 3NO0 3NO1 3NO2 3NO3 3NO4 3NO5 3NO6 3NO7 3NO8 3NO9 3NOA 3NOB 3NOC 3NOD 3NOE 3NOF 3NOG 3NOH 3NOI 3NOJ 3NOK 3NOL 3NOM 3NON 3NOO 3NOP 3NOQ 3NOR 3NOS 3NOT 3NOU 3NOV 3NOW 3NOX 3NOY 3NOZ 3NP0 3NP1 3NP2 3NP3 3NP4 3NP5 3NP7 3NP8 3NP9 3NPA 3NPC 3NPD 3NPE 3NPF 3NPG 3NPH 3NPI 3NPK 3NPL 3NPM 3NPO 3NPP 3NPR 3NPS 3NPU 3NPV 3NPW 3NPX 3NPY 3NPZ 3NQ0 3NQ1 3NQ2 3NQ3 3NQ4 3NQ5 3NQ6 3NQ7 3NQ8 3NQ9 3NQA 3NQB 3NQC 3NQD 3NQE 3NQF 3NQG 3NQH 3NQI 3NQJ 3NQK 3NQM 3NQN 3NQO 3NQP 3NQR 3NQS 3NQT 3NQU 3NQV 3NQW 3NQX 3NQY 3NQZ 3NR0 3NR1 3NR2 3NR3 3NR4 3NR5 3NR6 3NR7 3NR8 3NR9 3NRA 3NRB 3NRC 3NRD 3NRE 3NRF 3NRG 3NRH 3NRI 3NRJ 3NRK 3NRL 3NRM 3NRN 3NRO 3NRP 3NRQ 3NRR 3NRS 3NRT 3NRU 3NRV 3NRW 3NRX 3NRY 3NRZ 3NS0 3NS1 3NS2 3NS4 3NS5 3NS6 3NS7 3NS8 3NS9 3NSB 3NSC 3NSD 3NSE 3NSF 3NSG 3NSH 3NSI 3NSJ 3NSK 3NSL 3NSM 3NSN 3NSO 3NSP 3NSQ 3NSS 3NST 3NSU 3NSW 3NSX 3NSY 3NSZ 3NT0 3NT1 3NT2 3NT3 3NT4 3NT5 3NT6 3NT7 3NT8 3NT9 3NTA 3NTB 3NTC 3NTD 3NTE 3NTG 3NTH 3NTI 3NTJ 3NTK 3NTL 3NTM 3NTN 3NTO 3NTP 3NTQ 3NTR 3NTS 3NTT 3NTU 3NTV 3NTW 3NTX 3NTY 3NTZ 3NU0 3NU1 3NU3 3NU4 3NU5 3NU6 3NU7 3NU8 3NU9 3NUA 3NUB 3NUC 3NUD 3NUE 3NUF 3NUG 3NUH 3NUI 3NUJ 3NUK 3NUL 3NUM 3NUN 3NUO 3NUP 3NUQ 3NUR 3NUS 3NUT 3NUU 3NUV 3NUX 3NUY 3NUZ 3NV0 3NV1 3NV2 3NV3 3NV4 3NV5 3NV6 3NV7 3NV8 3NV9 3NVA 3NVC 3NVD 3NVE 3NVF 3NVG 3NVH 3NVJ 3NVL 3NVM 3NVN 3NVO 3NVQ 3NVR 3NVS 3NVT 3NVU 3NVV 3NVW 3NVX 3NVY 3NVZ 3NW0 3NW2 3NW3 3NW4 3NW5 3NW6 3NW7 3NW8 3NW9 3NWA 3NWB 3NWC 3NWD 3NWE 3NWF 3NWG 3NWH 3NWI 3NWJ 3NWK 3NWL 3NWM 3NWN 3NWO 3NWP 3NWQ 3NWR 3NWS 3NWT 3NWU 3NWV 3NWW 3NWX 3NWY 3NWZ 3NX0 3NX1 3NX2 3NX3 3NX4 3NX6 3NX7 3NX8 3NX9 3NXA 3NXB 3NXC 3NXD 3NXE 3NXF 3NXG 3NXH 3NXJ 3NXK 3NXL 3NXN 3NXO 3NXP 3NXQ 3NXR 3NXS 3NXT 3NXU 3NXV 3NXW 3NXX 3NXY 3NXZ 3NY0 3NY1 3NY2 3NY3 3NY4 3NY5 3NY6 3NY7 3NY8 3NY9 3NYA 3NYB 3NYC 3NYD 3NYE 3NYF 3NYG 3NYH 3NYI 3NYJ 3NYK 3NYL 3NYM 3NYN 3NYO 3NYQ 3NYR 3NYS 3NYT 3NYU 3NYV 3NYW 3NYX 3NYY 3NYZ 3NZ0 3NZ1 3NZ2 3NZ3 3NZ4 3NZ6 3NZ8 3NZ9 3NZA 3NZB 3NZC 3NZD 3NZE 3NZG 3NZH 3NZI 3NZJ 3NZK 3NZL 3NZM 3NZN 3NZP 3NZQ 3NZR 3NZS 3NZT 3NZU 3NZW 3NZX 3NZZ 3O00 3O01 3O02 3O03 3O04 3O05 3O06 3O07 3O08 3O0A 3O0D 3O0E 3O0F 3O0G 3O0H 3O0I 3O0J 3O0K 3O0L 3O0M 3O0N 3O0O 3O0P 3O0Q 3O0R 3O0T 3O0U 3O0V 3O0W 3O0X 3O0Y 3O0Z 3O10 3O11 3O12 3O13 3O14 3O15 3O16 3O17 3O18 3O19 3O1A 3O1B 3O1C 3O1D 3O1E 3O1F 3O1G 3O1H 3O1I 3O1J 3O1K 3O1L 3O1N 3O1Q 3O1W 3O1X 3O1Y 3O1Z 3O20 3O21 3O22 3O23 3O24 3O26 3O27 3O28 3O29 3O2A 3O2B 3O2C 3O2D 3O2E 3O2F 3O2G 3O2H 3O2I 3O2J 3O2K 3O2L 3O2M 3O2N 3O2O 3O2P 3O2Q 3O2R 3O2S 3O2T 3O2U 3O2V 3O2W 3O2X 3O2Y 3O31 3O32 3O33 3O34 3O35 3O36 3O37 3O38 3O39 3O3A 3O3B 3O3C 3O3D 3O3E 3O3J 3O3K 3O3L 3O3M 3O3N 3O3O 3O3P 3O3Q 3O3R 3O3T 3O3U 3O3V 3O3W 3O3X 3O3Y 3O3Z 3O40 3O41 3O42 3O43 3O44 3O45 3O46 3O47 3O48 3O49 3O4A 3O4B 3O4C 3O4D 3O4F 3O4G 3O4H 3O4I 3O4J 3O4K 3O4L 3O4M 3O4N 3O4O 3O4P 3O4Q 3O4R 3O4S 3O4T 3O4U 3O4V 3O4W 3O4X 3O4Y 3O4Z 3O50 3O51 3O52 3O53 3O55 3O56 3O57 3O59 3O5A 3O5B 3O5C 3O5D 3O5E 3O5F 3O5G 3O5I 3O5J 3O5K 3O5L 3O5M 3O5N 3O5O 3O5P 3O5Q 3O5R 3O5S 3O5T 3O5U 3O5V 3O5W 3O5X 3O5Y 3O5Z 3O60 3O61 3O63 3O64 3O65 3O66 3O69 3O6A 3O6B 3O6C 3O6D 3O6F 3O6G 3O6H 3O6I 3O6J 3O6K 3O6L 3O6M 3O6N 3O6O 3O6P 3O6Q 3O6R 3O6T 3O6U 3O6V 3O6W 3O6X 3O6Y 3O6Z 3O70 3O71 3O72 3O73 3O74 3O75 3O76 3O77 3O78 3O79 3O7A 3O7B 3O7H 3O7I 3O7J 3O7K 3O7L 3O7M 3O7N 3O7O 3O7P 3O7Q 3O7R 3O7S 3O7T 3O7U 3O7W 3O7X 3O80 3O81 3O82 3O83 3O84 3O85 3O86 3O87 3O88 3O89 3O8A 3O8B 3O8D 3O8E 3O8G 3O8H 3O8I 3O8J 3O8L 3O8M 3O8N 3O8O 3O8P 3O8Q 3O8S 3O8T 3O8U 3O8V 3O8W 3O8X 3O8Y 3O8Z 3O90 3O91 3O92 3O93 3O94 3O95 3O96 3O97 3O98 3O99 3O9A 3O9B 3O9C 3O9D 3O9E 3O9F 3O9G 3O9H 3O9I 3O9J 3O9K 3O9L 3O9M 3O9N 3O9O 3O9P 3O9Q 3O9R 3O9S 3O9T 3O9U 3O9V 3O9W 3O9Z 3OA0 3OA1 3OA2 3OA3 3OA4 3OA5 3OA7 3OA8 3OA9 3OAA 3OAB 3OAC 3OAD 3OAE 3OAF 3OAG 3OAH 3OAI 3OAJ 3OAK 3OAM 3OAN 3OAO 3OAP 3OAU 3OAW 3OAX 3OAY 3OAZ 3OB0 3OB1 3OB2 3OB4 3OB6 3OB7 3OB8 3OB9 3OBA 3OBB 3OBE 3OBF 3OBG 3OBH 3OBI 3OBJ 3OBK 3OBL 3OBP 3OBQ 3OBR 3OBS 3OBT 3OBU 3OBV 3OBW 3OBX 3OBY 3OBZ 3OC0 3OC1 3OC2 3OC3 3OC4 3OC5 3OC6 3OC7 3OC8 3OC9 3OCA 3OCB 3OCC 3OCD 3OCE 3OCF 3OCG 3OCH 3OCI 3OCJ 3OCL 3OCM 3OCN 3OCO 3OCP 3OCQ 3OCR 3OCS 3OCT 3OCU 3OCV 3OCW 3OCX 3OCY 3OCZ 3OD0 3OD1 3OD2 3OD3 3OD4 3OD5 3OD6 3OD7 3OD9 3ODB 3ODD 3ODF 3ODG 3ODI 3ODJ 3ODK 3ODL 3ODM 3ODN 3ODO 3ODP 3ODQ 3ODR 3ODS 3ODT 3ODU 3ODV 3ODW 3ODX 3ODY 3ODZ 3OE0 3OE1 3OE2 3OE3 3OE4 3OE5 3OE6 3OE7 3OE8 3OE9 3OEA 3OEB 3OEC 3OED 3OEE 3OEF 3OEH 3OEI 3OEK 3OEL 3OEM 3OEN 3OEO 3OEP 3OEQ 3OER 3OES 3OET 3OEU 3OEV 3OEW 3OEX 3OEY 3OEZ 3OF0 3OF1 3OF2 3OF3 3OF4 3OF5 3OF6 3OF7 3OF8 3OF9 3OFE 3OFF 3OFG 3OFH 3OFI 3OFJ 3OFK 3OFL 3OFM 3OFN 3OFS 3OFT 3OFU 3OFV 3OFW 3OG2 3OG3 3OG4 3OG5 3OG6 3OG7 3OG9 3OGA 3OGB 3OGC 3OGF 3OGG 3OGH 3OGI 3OGJ 3OGK 3OGL 3OGM 3OGN 3OGO 3OGP 3OGQ 3OGR 3OGS 3OGT 3OGV 3OGW 3OGX 3OGZ 3OH0 3OH1 3OH2 3OH3 3OH4 3OH8 3OHE 3OHF 3OHG 3OHH 3OHI 3OHL 3OHM 3OHN 3OHO 3OHP 3OHR 3OHS 3OHT 3OHU 3OHV 3OHW 3OHX 3OI7 3OI8 3OI9 3OIA 3OIB 3OIC 3OID 3OIF 3OIG 3OIH 3OII 3OIK 3OIL 3OIM 3OIO 3OIP 3OIQ 3OIR 3OIS 3OIT 3OIU 3OIV 3OIW 3OIX 3OIY 3OIZ 3OJ0 3OJ1 3OJ2 3OJ3 3OJ4 3OJ5 3OJ6 3OJ7 3OJ8 3OJA 3OJB 3OJC 3OJD 3OJE 3OJF 3OJG 3OJI 3OJJ 3OJK 3OJL 3OJM 3OJN 3OJO 3OJP 3OJT 3OJV 3OJW 3OJX 3OJY 3OK0 3OK5 3OK8 3OK9 3OKA 3OKC 3OKD 3OKE 3OKF 3OKG 3OKH 3OKI 3OKJ 3OKK 3OKL 3OKM 3OKN 3OKO 3OKP 3OKQ 3OKR 3OKS 3OKT 3OKU 3OKV 3OKW 3OKX 3OKY 3OKZ 3OL0 3OL2 3OL3 3OL4 3OL5 3OLC 3OLD 3OLE 3OLF 3OLG 3OLH 3OLI 3OLJ 3OLL 3OLM 3OLN 3OLO 3OLP 3OLQ 3OLR 3OLS 3OLT 3OLU 3OLV 3OLW 3OLX 3OLY 3OLZ 3OM0 3OM1 3OM2 3OM3 3OM4 3OM5 3OM6 3OM7 3OM8 3OM9 3OMA 3OMB 3OMC 3OMD 3OME 3OMF 3OMG 3OMH 3OMI 3OMK 3OML 3OMM 3OMN 3OMO 3OMP 3OMQ 3OMS 3OMT 3OMU 3OMV 3OMW 3OMX 3OMY 3OMZ 3ON1 3ON2 3ON3 3ON4 3ON5 3ON6 3ON7 3ON9 3ONA 3ONB 3ONC 3OND 3ONE 3ONF 3ONG 3ONH 3ONI 3ONJ 3ONK 3ONL 3ONM 3ONN 3ONO 3ONP 3ONQ 3ONR 3ONS 3ONT 3ONU 3ONV 3ONW 3ONX 3ONY 3ONZ 3OO0 3OO1 3OO2 3OO3 3OO4 3OO5 3OO6 3OO7 3OO8 3OO9 3OOA 3OOB 3OOC 3OOD 3OOE 3OOF 3OOG 3OOH 3OOI 3OOJ 3OOK 3OOM 3OON 3OOO 3OOP 3OOQ 3OOS 3OOT 3OOU 3OOV 3OOW 3OOX 3OOY 3OOZ 3OP0 3OP1 3OP2 3OP3 3OP4 3OP5 3OP6 3OP7 3OP8 3OP9 3OPB 3OPC 3OPD 3OPE 3OPF 3OPH 3OPK 3OPL 3OPM 3OPN 3OPO 3OPP 3OPQ 3OPR 3OPS 3OPT 3OPU 3OPV 3OPW 3OPX 3OPY 3OPZ 3OQ0 3OQ1 3OQ2 3OQ3 3OQ4 3OQ5 3OQ6 3OQ7 3OQ8 3OQ9 3OQA 3OQB 3OQC 3OQD 3OQE 3OQF 3OQH 3OQI 3OQJ 3OQK 3OQL 3OQP 3OQQ 3OQR 3OQS 3OQT 3OQU 3OQV 3OQY 3OQZ 3OR0 3OR1 3OR2 3OR5 3OR6 3OR7 3ORD 3ORE 3ORF 3ORG 3ORH 3ORI 3ORJ 3ORK 3ORL 3ORM 3ORN 3ORO 3ORP 3ORQ 3ORR 3ORS 3ORT 3ORU 3ORV 3ORW 3ORX 3ORY 3ORZ 3OS3 3OS4 3OS5 3OS6 3OS7 3OS8 3OS9 3OSA 3OSD 3OSE 3OSH 3OSI 3OSJ 3OSK 3OSL 3OSM 3OSO 3OSQ 3OSR 3OSS 3OST 3OSU 3OSV 3OSW 3OSX 3OSY 3OSZ 3OT1 3OT2 3OT3 3OT4 3OT5 3OT6 3OT7 3OT8 3OT9 3OTB 3OTC 3OTD 3OTE 3OTF 3OTG 3OTH 3OTI 3OTK 3OTL 3OTM 3OTN 3OTP 3OTQ 3OTR 3OTS 3OTT 3OTU 3OTV 3OTW 3OTX 3OTY 3OTZ 3OU0 3OU1 3OU2 3OU3 3OU4 3OU5 3OU6 3OU7 3OU8 3OU9 3OUA 3OUB 3OUC 3OUD 3OUE 3OUF 3OUG 3OUH 3OUI 3OUJ 3OUK 3OUL 3OUM 3OUN 3OUO 3OUP 3OUQ 3OUR 3OUS 3OUT 3OUU 3OUV 3OUW 3OUX 3OUZ 3OV0 3OV1 3OV2 3OV3 3OV4 3OV5 3OV6 3OV8 3OV9 3OVE 3OVG 3OVJ 3OVK 3OVL 3OVM 3OVN 3OVO 3OVP 3OVQ 3OVR 3OVU 3OVV 3OVW 3OVX 3OVZ 3OW1 3OW3 3OW4 3OW5 3OW6 3OW7 3OW8 3OW9 3OWA 3OWB 3OWC 3OWD 3OWE 3OWF 3OWG 3OWH 3OWJ 3OWK 3OWL 3OWM 3OWN 3OWO 3OWP 3OWQ 3OWR 3OWS 3OWT 3OWU 3OWV 3OWX 3OWY 3OX1 3OX2 3OX3 3OX4 3OX5 3OX6 3OX7 3OX8 3OX9 3OXA 3OXC 3OXF 3OXG 3OXH 3OXI 3OXK 3OXL 3OXN 3OXO 3OXP 3OXQ 3OXR 3OXS 3OXT 3OXU 3OXV 3OXW 3OXX 3OXZ 3OY0 3OY1 3OY2 3OY3 3OY4 3OY5 3OY6 3OY7 3OY8 3OYO 3OYP 3OYQ 3OYR 3OYS 3OYT 3OYV 3OYW 3OYX 3OYY 3OYZ 3OZ0 3OZ1 3OZ2 3OZ6 3OZ7 3OZ9 3OZA 3OZB 3OZC 3OZD 3OZE 3OZF 3OZG 3OZH 3OZI 3OZJ 3OZK 3OZL 3OZM 3OZO 3OZP 3OZQ 3OZR 3OZS 3OZT 3OZU 3OZV 3OZW 3OZX 3OZY 3OZZ 3P01 3P02 3P03 3P04 3P05 3P06 3P08 3P09 3P0A 3P0B 3P0C 3P0E 3P0F 3P0G 3P0H 3P0I 3P0J 3P0K 3P0L 3P0M 3P0N 3P0P 3P0Q 3P0R 3P0S 3P0T 3P0U 3P0V 3P0W 3P0X 3P0Y 3P0Z 3P10 3P11 3P12 3P13 3P14 3P16 3P17 3P19 3P1A 3P1B 3P1C 3P1D 3P1E 3P1F 3P1G 3P1H 3P1I 3P1J 3P1L 3P1M 3P1N 3P1O 3P1P 3P1Q 3P1R 3P1S 3P1T 3P1U 3P1V 3P1W 3P1X 3P1Y 3P1Z 3P20 3P23 3P24 3P26 3P27 3P28 3P2A 3P2B 3P2C 3P2D 3P2E 3P2F 3P2H 3P2I 3P2J 3P2K 3P2L 3P2M 3P2N 3P2O 3P2P 3P2Q 3P2R 3P2S 3P2T 3P2U 3P2V 3P2W 3P2X 3P2Y 3P2Z 3P30 3P31 3P32 3P33 3P34 3P35 3P36 3P37 3P38 3P39 3P3A 3P3B 3P3C 3P3D 3P3E 3P3F 3P3G 3P3H 3P3I 3P3J 3P3K 3P3L 3P3N 3P3O 3P3P 3P3Q 3P3R 3P3S 3P3T 3P3U 3P3V 3P3W 3P3X 3P3Y 3P3Z 3P40 3P41 3P42 3P43 3P44 3P45 3P46 3P47 3P48 3P4E 3P4F 3P4G 3P4H 3P4I 3P4K 3P4L 3P4M 3P4N 3P4O 3P4P 3P4Q 3P4R 3P4S 3P4T 3P4U 3P4V 3P4W 3P4X 3P4Y 3P4Z 3P50 3P51 3P52 3P53 3P54 3P55 3P56 3P58 3P5A 3P5B 3P5C 3P5D 3P5E 3P5F 3P5G 3P5H 3P5I 3P5J 3P5K 3P5L 3P5M 3P5N 3P5O 3P5P 3P5Q 3P5R 3P5S 3P5T 3P5U 3P5V 3P5W 3P5X 3P5Y 3P5Z 3P60 3P61 3P62 3P63 3P64 3P65 3P66 3P67 3P68 3P69 3P6A 3P6B 3P6C 3P6D 3P6E 3P6F 3P6G 3P6H 3P6I 3P6J 3P6K 3P6L 3P6M 3P6N 3P6O 3P6P 3P6Q 3P6R 3P6S 3P6T 3P6U 3P6V 3P6W 3P6X 3P6Z 3P70 3P71 3P72 3P73 3P74 3P75 3P76 3P77 3P78 3P79 3P7A 3P7B 3P7C 3P7F 3P7G 3P7H 3P7I 3P7J 3P7K 3P7L 3P7M 3P7N 3P7O 3P7P 3P7Q 3P7R 3P7S 3P7T 3P7U 3P7V 3P7W 3P7X 3P7Y 3P7Z 3P80 3P81 3P82 3P83 3P84 3P85 3P86 3P87 3P88 3P89 3P8A 3P8B 3P8C 3P8D 3P8E 3P8F 3P8G 3P8H 3P8I 3P8J 3P8K 3P8L 3P8M 3P8N 3P8O 3P8P 3P8R 3P8S 3P8T 3P8U 3P8V 3P8W 3P8X 3P8Y 3P8Z 3P90 3P91 3P92 3P93 3P94 3P95 3P96 3P97 3P98 3P99 3P9A 3P9C 3P9F 3P9G 3P9H 3P9I 3P9J 3P9K 3P9L 3P9M 3P9N 3P9O 3P9P 3P9Q 3P9R 3P9S 3P9T 3P9U 3P9V 3P9W 3P9X 3P9Y 3P9Z 3PA1 3PA2 3PA3 3PA4 3PA5 3PA6 3PA7 3PA8 3PA9 3PAA 3PAB 3PAC 3PAE 3PAF 3PAG 3PAH 3PAJ 3PAK 3PAL 3PAM 3PAN 3PAO 3PAQ 3PAR 3PAS 3PAU 3PAV 3PAW 3PAX 3PAY 3PAZ 3PB0 3PB1 3PB2 3PB3 3PB4 3PB5 3PB6 3PB7 3PB8 3PB9 3PBA 3PBB 3PBC 3PBD 3PBE 3PBF 3PBG 3PBH 3PBI 3PBJ 3PBK 3PBL 3PBM 3PBN 3PBO 3PBP 3PBQ 3PBR 3PBS 3PBT 3PBU 3PBV 3PBW 3PBY 3PBZ 3PC0 3PC2 3PC3 3PC4 3PC6 3PC7 3PC8 3PCA 3PCB 3PCC 3PCD 3PCE 3PCF 3PCG 3PCH 3PCI 3PCJ 3PCK 3PCL 3PCM 3PCN 3PCO 3PCQ 3PCR 3PCS 3PCT 3PCU 3PCV 3PCW 3PCX 3PCY 3PCZ 3PD0 3PD1 3PD2 3PD3 3PD4 3PD5 3PD6 3PD7 3PD8 3PD9 3PDB 3PDC 3PDD 3PDE 3PDF 3PDG 3PDH 3PDI 3PDJ 3PDK 3PDN 3PDO 3PDQ 3PDS 3PDT 3PDU 3PDV 3PDW 3PDX 3PDY 3PE0 3PE1 3PE2 3PE3 3PE4 3PE5 3PE6 3PE7 3PE8 3PE9 3PEA 3PEB 3PEC 3PED 3PEE 3PEF 3PEG 3PEH 3PEI 3PEJ 3PEL 3PEN 3PEO 3PEP 3PEQ 3PER 3PES 3PET 3PEU 3PEV 3PF0 3PF1 3PF2 3PF3 3PF6 3PF7 3PF8 3PF9 3PFB 3PFC 3PFD 3PFE 3PFF 3PFG 3PFH 3PFI 3PFJ 3PFK 3PFL 3PFM 3PFN 3PFO 3PFP 3PFQ 3PFR 3PFS 3PFT 3PFU 3PFV 3PFW 3PFX 3PFY 3PFZ 3PG0 3PG1 3PG2 3PG3 3PG4 3PG5 3PG6 3PG7 3PG8 3PG9 3PGA 3PGB 3PGC 3PGD 3PGE 3PGF 3PGG 3PGH 3PGI 3PGJ 3PGK 3PGL 3PGM 3PGP 3PGQ 3PGR 3PGS 3PGT 3PGU 3PGV 3PGX 3PGY 3PGZ 3PH0 3PH1 3PH2 3PH3 3PH4 3PH5 3PH6 3PH7 3PH9 3PHA 3PHB 3PHC 3PHD 3PHE 3PHF 3PHG 3PHH 3PHI 3PHJ 3PHL 3PHM 3PHN 3PHO 3PHQ 3PHS 3PHT 3PHU 3PHV 3PHW 3PHX 3PHZ 3PI0 3PI1 3PI2 3PI3 3PI4 3PI5 3PI6 3PI7 3PI8 3PI9 3PIA 3PIB 3PIC 3PID 3PIE 3PIF 3PIG 3PII 3PIJ 3PIK 3PIL 3PIM 3PIN 3PIQ 3PIR 3PIS 3PIT 3PIU 3PIV 3PIW 3PIX 3PIY 3PIZ 3PJ0 3PJ1 3PJ2 3PJ3 3PJ5 3PJ6 3PJ7 3PJ8 3PJ9 3PJA 3PJB 3PJC 3PJD 3PJE 3PJF 3PJG 3PJI 3PJJ 3PJK 3PJL 3PJN 3PJP 3PJQ 3PJS 3PJT 3PJU 3PJV 3PJW 3PJX 3PJY 3PJZ 3PK0 3PK1 3PK2 3PK3 3PK4 3PK5 3PK6 3PK7 3PK8 3PKA 3PKB 3PKC 3PKD 3PKE 3PKF 3PKG 3PKH 3PKI 3PKJ 3PKK 3PKL 3PKN 3PKO 3PKP 3PKQ 3PKS 3PKT 3PKU 3PKV 3PKW 3PKX 3PKZ 3PL0 3PL1 3PL2 3PL3 3PL5 3PL6 3PL7 3PL8 3PL9 3PLB 3PLC 3PLD 3PLE 3PLF 3PLG 3PLH 3PLI 3PLJ 3PLK 3PLL 3PLM 3PLN 3PLP 3PLQ 3PLR 3PLS 3PLT 3PLU 3PLV 3PLW 3PLX 3PLY 3PLZ 3PM0 3PM1 3PM2 3PM3 3PM4 3PM5 3PM6 3PM7 3PM8 3PM9 3PMA 3PMB 3PMC 3PMD 3PME 3PMF 3PMG 3PMH 3PMI 3PMJ 3PMK 3PMM 3PMO 3PMP 3PMQ 3PMR 3PMS 3PMT 3PMU 3PMV 3PMW 3PMX 3PMY 3PMZ 3PN1 3PN2 3PN3 3PN4 3PN5 3PN6 3PN7 3PN8 3PN9 3PNA 3PNB 3PND 3PNE 3PNF 3PNG 3PNH 3PNI 3PNK 3PNL 3PNM 3PNN 3PNO 3PNP 3PNQ 3PNR 3PNS 3PNT 3PNU 3PNV 3PNW 3PNX 3PNY 3PNZ 3PO0 3PO1 3PO6 3PO7 3PO8 3PO9 3POA 3POB 3POC 3POD 3POE 3POF 3POG 3POH 3POI 3POJ 3POK 3POL 3POM 3PON 3POO 3POP 3POQ 3POR 3POS 3POT 3POU 3POW 3POX 3POY 3POZ 3PP0 3PP1 3PP2 3PP3 3PP4 3PP5 3PP6 3PP7 3PP8 3PP9 3PPA 3PPB 3PPC 3PPD 3PPE 3PPF 3PPG 3PPH 3PPI 3PPJ 3PPK 3PPL 3PPM 3PPN 3PPO 3PPP 3PPQ 3PPR 3PPS 3PPT 3PPU 3PPV 3PPW 3PPX 3PPY 3PPZ 3PQ1 3PQ2 3PQ3 3PQ4 3PQ5 3PQ6 3PQ7 3PQ8 3PQA 3PQB 3PQC 3PQD 3PQE 3PQF 3PQH 3PQI 3PQJ 3PQK 3PQR 3PQS 3PQU 3PQV 3PQY 3PQZ 3PR0 3PR1 3PR2 3PR3 3PR6 3PR7 3PR8 3PR9 3PRA 3PRB 3PRC 3PRD 3PRE 3PRF 3PRG 3PRH 3PRI 3PRJ 3PRK 3PRL 3PRM 3PRN 3PRO 3PRP 3PRS 3PRT 3PRU 3PRV 3PRW 3PRX 3PRY 3PRZ 3PS0 3PS1 3PS2 3PS3 3PS4 3PS5 3PS6 3PS7 3PS8 3PS9 3PSA 3PSB 3PSC 3PSD 3PSE 3PSF 3PSG 3PSH 3PSI 3PSJ 3PSK 3PSL 3PSM 3PSN 3PSO 3PSP 3PSQ 3PSR 3PSS 3PST 3PSU 3PSV 3PSW 3PSX 3PSY 3PSZ 3PT1 3PT2 3PT3 3PT5 3PT7 3PT8 3PT9 3PTB 3PTD 3PTE 3PTF 3PTG 3PTH 3PTJ 3PTK 3PTL 3PTM 3PTN 3PTO 3PTQ 3PTR 3PTW 3PTY 3PTZ 3PU2 3PU3 3PU5 3PU6 3PU7 3PU8 3PU9 3PUA 3PUB 3PUC 3PUD 3PUE 3PUF 3PUG 3PUH 3PUI 3PUJ 3PUK 3PUL 3PUM 3PUN 3PUO 3PUP 3PUQ 3PUR 3PUS 3PUT 3PUU 3PUV 3PUW 3PUX 3PUY 3PUZ 3PV0 3PV1 3PV2 3PV3 3PV4 3PV5 3PV6 3PV7 3PVA 3PVB 3PVC 3PVD 3PVE 3PVF 3PVG 3PVH 3PVJ 3PVK 3PVL 3PVM 3PVN 3PVO 3PVQ 3PVR 3PVS 3PVT 3PVU 3PVW 3PVY 3PVZ 3PW1 3PW3 3PW8 3PW9 3PWA 3PWB 3PWC 3PWD 3PWE 3PWF 3PWG 3PWH 3PWI 3PWJ 3PWK 3PWL 3PWM 3PWN 3PWP 3PWQ 3PWR 3PWS 3PWT 3PWU 3PWV 3PWW 3PWX 3PWY 3PWZ 3PX1 3PX2 3PX3 3PX8 3PX9 3PXA 3PXB 3PXC 3PXD 3PXE 3PXF 3PXG 3PXH 3PXI 3PXJ 3PXK 3PXL 3PXM 3PXN 3PXO 3PXP 3PXQ 3PXR 3PXS 3PXT 3PXU 3PXV 3PXW 3PXX 3PXY 3PXZ 3PY0 3PY1 3PY2 3PY3 3PY4 3PY5 3PY6 3PY7 3PY9 3PYA 3PYB 3PYC 3PYD 3PYE 3PYF 3PYG 3PYH 3PYI 3PYJ 3PYK 3PYL 3PYM 3PYP 3PYW 3PYX 3PYY 3PYZ 3PZ0 3PZ1 3PZ2 3PZ3 3PZ4 3PZ5 3PZ6 3PZ7 3PZ8 3PZ9 3PZA 3PZB 3PZC 3PZD 3PZE 3PZF 3PZG 3PZH 3PZI 3PZJ 3PZK 3PZL 3PZM 3PZN 3PZO 3PZQ 3PZR 3PZS 3PZT 3PZU 3PZV 3PZW 3PZY 3PZZ 3Q00 3Q01 3Q02 3Q03 3Q04 3Q07 3Q08 3Q09 3Q0E 3Q0G 3Q0H 3Q0I 3Q0J 3Q0K 3Q0T 3Q0U 3Q0V 3Q0W 3Q0X 3Q0Y 3Q0Z 3Q10 3Q11 3Q12 3Q13 3Q14 3Q15 3Q16 3Q17 3Q18 3Q19 3Q1C 3Q1D 3Q1E 3Q1F 3Q1G 3Q1H 3Q1I 3Q1J 3Q1K 3Q1L 3Q1N 3Q1O 3Q1P 3Q1S 3Q1T 3Q1X 3Q1Y 3Q20 3Q25 3Q26 3Q27 3Q28 3Q29 3Q2A 3Q2B 3Q2C 3Q2D 3Q2E 3Q2F 3Q2G 3Q2H 3Q2I 3Q2J 3Q2K 3Q2L 3Q2M 3Q2N 3Q2O 3Q2P 3Q2Q 3Q2R 3Q2S 3Q2U 3Q2V 3Q2W 3Q2X 3Q2Z 3Q30 3Q31 3Q32 3Q33 3Q34 3Q35 3Q36 3Q37 3Q38 3Q39 3Q3A 3Q3B 3Q3C 3Q3E 3Q3F 3Q3G 3Q3H 3Q3I 3Q3J 3Q3K 3Q3M 3Q3N 3Q3O 3Q3Q 3Q3S 3Q3T 3Q3U 3Q3V 3Q3W 3Q3X 3Q3Y 3Q40 3Q41 3Q43 3Q44 3Q45 3Q46 3Q47 3Q48 3Q49 3Q4A 3Q4B 3Q4C 3Q4D 3Q4F 3Q4G 3Q4H 3Q4I 3Q4J 3Q4K 3Q4L 3Q4N 3Q4O 3Q4P 3Q4Q 3Q4R 3Q4S 3Q4T 3Q4U 3Q4W 3Q4Y 3Q4Z 3Q52 3Q53 3Q54 3Q58 3Q5D 3Q5E 3Q5G 3Q5H 3Q5I 3Q5J 3Q5K 3Q5L 3Q5M 3Q5O 3Q5T 3Q5U 3Q5V 3Q5W 3Q5X 3Q5Y 3Q5Z 3Q60 3Q62 3Q63 3Q64 3Q65 3Q66 3Q67 3Q68 3Q69 3Q6A 3Q6B 3Q6C 3Q6D 3Q6E 3Q6F 3Q6G 3Q6I 3Q6J 3Q6K 3Q6L 3Q6M 3Q6N 3Q6O 3Q6P 3Q6Q 3Q6R 3Q6S 3Q6T 3Q6U 3Q6V 3Q6W 3Q6X 3Q6Y 3Q6Z 3Q70 3Q71 3Q72 3Q73 3Q74 3Q75 3Q76 3Q77 3Q78 3Q79 3Q7A 3Q7B 3Q7C 3Q7D 3Q7E 3Q7F 3Q7G 3Q7H 3Q7I 3Q7J 3Q7K 3Q7L 3Q7M 3Q7N 3Q7O 3Q7P 3Q7Q 3Q7R 3Q7S 3Q7T 3Q7U 3Q7V 3Q7W 3Q7X 3Q7Y 3Q7Z 3Q80 3Q81 3Q82 3Q83 3Q84 3Q85 3Q86 3Q87 3Q88 3Q89 3Q8A 3Q8B 3Q8C 3Q8D 3Q8E 3Q8F 3Q8G 3Q8H 3Q8I 3Q8J 3Q8N 3Q8T 3Q8U 3Q8V 3Q8W 3Q8X 3Q8Y 3Q90 3Q91 3Q92 3Q93 3Q94 3Q95 3Q96 3Q97 3Q98 3Q99 3Q9A 3Q9B 3Q9C 3Q9D 3Q9E 3Q9F 3Q9G 3Q9H 3Q9I 3Q9J 3Q9K 3Q9L 3Q9M 3Q9N 3Q9O 3Q9P 3Q9Q 3Q9S 3Q9T 3Q9U 3Q9V 3Q9W 3Q9X 3Q9Y 3Q9Z 3QA0 3QA2 3QA3 3QA8 3QA9 3QAA 3QAC 3QAE 3QAG 3QAH 3QAI 3QAK 3QAL 3QAM 3QAN 3QAO 3QAP 3QAQ 3QAR 3QAS 3QAT 3QAU 3QAV 3QAW 3QAX 3QAY 3QAZ 3QB0 3QB1 3QB2 3QB3 3QB4 3QB5 3QB7 3QB8 3QB9 3QBC 3QBD 3QBE 3QBF 3QBG 3QBH 3QBI 3QBJ 3QBK 3QBL 3QBM 3QBN 3QBO 3QBP 3QBQ 3QBR 3QBT 3QBU 3QBV 3QBW 3QBX 3QBY 3QBZ 3QC0 3QC1 3QC2 3QC3 3QC4 3QC5 3QC6 3QC7 3QC8 3QC9 3QCA 3QCB 3QCC 3QCD 3QCE 3QCF 3QCG 3QCH 3QCI 3QCJ 3QCK 3QCL 3QCM 3QCN 3QCP 3QCQ 3QCS 3QCT 3QCU 3QCV 3QCW 3QCX 3QCY 3QCZ 3QD0 3QD2 3QD3 3QD4 3QD5 3QD6 3QD7 3QD8 3QD9 3QDA 3QDC 3QDD 3QDE 3QDF 3QDG 3QDH 3QDJ 3QDK 3QDL 3QDM 3QDN 3QDO 3QDP 3QDQ 3QDR 3QDS 3QDT 3QDU 3QDV 3QDW 3QDX 3QDY 3QDZ 3QE0 3QE1 3QE2 3QE3 3QE4 3QE5 3QE6 3QE7 3QE8 3QEC 3QED 3QEE 3QEF 3QEG 3QEH 3QEJ 3QEK 3QEL 3QEM 3QEN 3QEO 3QEQ 3QEU 3QEZ 3QF0 3QF1 3QF2 3QF3 3QF4 3QF7 3QF9 3QFA 3QFB 3QFC 3QFD 3QFE 3QFF 3QFG 3QFH 3QFI 3QFJ 3QFK 3QFL 3QFM 3QFN 3QFO 3QFP 3QFR 3QFS 3QFT 3QFU 3QFV 3QFW 3QFX 3QFY 3QFZ 3QG0 3QG1 3QG2 3QG5 3QG6 3QG7 3QGA 3QGD 3QGE 3QGF 3QGG 3QGH 3QGI 3QGJ 3QGK 3QGL 3QGM 3QGN 3QGO 3QGP 3QGT 3QGU 3QGV 3QGW 3QGY 3QGZ 3QH0 3QH1 3QH2 3QH3 3QH4 3QH5 3QH6 3QH7 3QH8 3QH9 3QHA 3QHB 3QHC 3QHD 3QHE 3QHF 3QHM 3QHN 3QHO 3QHP 3QHQ 3QHR 3QHS 3QHT 3QHW 3QHX 3QHY 3QHZ 3QI0 3QI1 3QI2 3QI3 3QI4 3QI6 3QI7 3QI8 3QI9 3QIA 3QIB 3QIC 3QID 3QIH 3QII 3QIJ 3QIK 3QIL 3QIM 3QIN 3QIO 3QIP 3QIR 3QIS 3QIT 3QIU 3QIV 3QIW 3QIX 3QIY 3QIZ 3QJ0 3QJ1 3QJ3 3QJ4 3QJ5 3QJ6 3QJ7 3QJ8 3QJ9 3QJA 3QJB 3QJC 3QJD 3QJE 3QJF 3QJG 3QJH 3QJI 3QJK 3QJM 3QJN 3QJO 3QJQ 3QJR 3QJS 3QJT 3QJU 3QJV 3QJX 3QJY 3QJZ 3QK0 3QK1 3QK2 3QK3 3QK5 3QK6 3QK7 3QK8 3QK9 3QKA 3QKB 3QKC 3QKD 3QKE 3QKG 3QKI 3QKJ 3QKK 3QKL 3QKM 3QKP 3QKQ 3QKR 3QKS 3QKT 3QKU 3QKV 3QKW 3QKX 3QKY 3QKZ 3QL0 3QL1 3QL2 3QL3 3QL6 3QL8 3QL9 3QLA 3QLB 3QLC 3QLD 3QLE 3QLF 3QLG 3QLH 3QLI 3QLJ 3QLK 3QLL 3QLM 3QLN 3QLQ 3QLR 3QLS 3QLT 3QLU 3QLV 3QLW 3QLX 3QLY 3QLZ 3QM0 3QM1 3QM2 3QM3 3QM4 3QM5 3QM6 3QM7 3QM8 3QM9 3QMA 3QME 3QMF 3QMJ 3QMK 3QML 3QMM 3QMN 3QMO 3QMP 3QMQ 3QMR 3QMS 3QMT 3QMU 3QMV 3QMW 3QMX 3QMZ 3QN0 3QN1 3QN2 3QN3 3QN6 3QN7 3QN8 3QN9 3QNA 3QNB 3QNC 3QND 3QNE 3QNF 3QNG 3QNI 3QNJ 3QNK 3QNL 3QNM 3QNQ 3QNR 3QNS 3QNT 3QNU 3QNV 3QNW 3QNX 3QNY 3QNZ 3QO0 3QO1 3QO2 3QO3 3QO4 3QO5 3QO6 3QO7 3QO8 3QO9 3QOA 3QOC 3QOD 3QOE 3QOF 3QOJ 3QOK 3QOL 3QOM 3QON 3QOO 3QOP 3QOR 3QOS 3QOT 3QOU 3QOW 3QOX 3QOY 3QP0 3QP1 3QP2 3QP3 3QP4 3QP5 3QP6 3QP8 3QP9 3QPA 3QPB 3QPC 3QPD 3QPE 3QPF 3QPG 3QPH 3QPI 3QPJ 3QPK 3QPL 3QPM 3QPN 3QPO 3QPP 3QPQ 3QPR 3QPS 3QPT 3QPU 3QPV 3QPW 3QPX 3QPY 3QPZ 3QQ0 3QQ1 3QQ2 3QQ3 3QQ4 3QQ5 3QQ6 3QQ7 3QQ8 3QQ9 3QQA 3QQB 3QQC 3QQD 3QQE 3QQF 3QQG 3QQH 3QQI 3QQJ 3QQK 3QQL 3QQM 3QQN 3QQO 3QQP 3QQQ 3QQR 3QQS 3QQT 3QQU 3QQV 3QQW 3QQX 3QQZ 3QR0 3QR1 3QR2 3QR3 3QR5 3QR6 3QR7 3QR8 3QR9 3QRA 3QRB 3QRC 3QRD 3QRE 3QRG 3QRH 3QRI 3QRJ 3QRK 3QRL 3QRM 3QRO 3QRS 3QRT 3QRU 3QRV 3QRW 3QRX 3QRY 3QS0 3QS1 3QS2 3QS3 3QS4 3QS5 3QS6 3QS7 3QS8 3QS9 3QSA 3QSB 3QSD 3QSE 3QSG 3QSI 3QSJ 3QSK 3QSL 3QSM 3QSP 3QSQ 3QSR 3QSS 3QST 3QSZ 3QT0 3QT1 3QT2 3QT3 3QT4 3QT5 3QT6 3QT7 3QT8 3QT9 3QTA 3QTB 3QTC 3QTD 3QTE 3QTF 3QTG 3QTH 3QTI 3QTK 3QTL 3QTM 3QTN 3QTO 3QTP 3QTQ 3QTR 3QTS 3QTT 3QTU 3QTV 3QTW 3QTX 3QTY 3QTZ 3QU0 3QU1 3QU2 3QU3 3QU4 3QU5 3QU6 3QU7 3QU8 3QU9 3QUA 3QUB 3QUC 3QUD 3QUE 3QUF 3QUG 3QUH 3QUI 3QUJ 3QUK 3QUL 3QUM 3QUN 3QUO 3QUP 3QUQ 3QUR 3QUS 3QUT 3QUV 3QUW 3QUX 3QUY 3QUZ 3QV0 3QV1 3QV2 3QV4 3QV6 3QV7 3QV8 3QV9 3QVA 3QVB 3QVC 3QVD 3QVE 3QVF 3QVG 3QVH 3QVI 3QVJ 3QVK 3QVL 3QVM 3QVN 3QVO 3QVP 3QVQ 3QVR 3QVS 3QVT 3QVU 3QVV 3QVW 3QVX 3QVY 3QVZ 3QW0 3QW1 3QW2 3QW3 3QW4 3QW5 3QW6 3QW7 3QW8 3QW9 3QWA 3QWB 3QWC 3QWD 3QWE 3QWF 3QWG 3QWH 3QWI 3QWJ 3QWK 3QWL 3QWM 3QWN 3QWO 3QWP 3QWQ 3QWR 3QWT 3QWU 3QWV 3QWW 3QWX 3QWY 3QWZ 3QX1 3QX2 3QX4 3QX5 3QX7 3QX8 3QX9 3QXA 3QXB 3QXC 3QXD 3QXE 3QXF 3QXG 3QXH 3QXI 3QXJ 3QXL 3QXM 3QXO 3QXP 3QXQ 3QXS 3QXT 3QXU 3QXV 3QXW 3QXX 3QXY 3QXZ 3QY0 3QY1 3QY2 3QY3 3QY4 3QY5 3QY6 3QY7 3QY8 3QY9 3QYA 3QYB 3QYC 3QYD 3QYE 3QYF 3QYG 3QYH 3QYJ 3QYK 3QYL 3QYO 3QYP 3QYQ 3QYR 3QYS 3QYT 3QYU 3QYW 3QYY 3QYZ 3QZ0 3QZ1 3QZ2 3QZ3 3QZ4 3QZ5 3QZ6 3QZ9 3QZB 3QZC 3QZE 3QZF 3QZG 3QZH 3QZI 3QZL 3QZM 3QZN 3QZO 3QZP 3QZQ 3QZR 3QZS 3QZT 3QZU 3QZV 3QZW 3QZX 3QZY 3QZZ 3R00 3R01 3R02 3R03 3R04 3R05 3R06 3R07 3R08 3R09 3R0A 3R0D 3R0E 3R0F 3R0G 3R0H 3R0I 3R0J 3R0K 3R0L 3R0M 3R0N 3R0O 3R0P 3R0Q 3R0R 3R0S 3R0T 3R0U 3R0V 3R0W 3R0X 3R0Y 3R0Z 3R10 3R11 3R12 3R13 3R15 3R16 3R17 3R18 3R19 3R1A 3R1B 3R1F 3R1G 3R1I 3R1J 3R1K 3R1M 3R1N 3R1O 3R1P 3R1Q 3R1R 3R1S 3R1V 3R1W 3R1X 3R1Y 3R1Z 3R20 3R21 3R22 3R23 3R24 3R25 3R26 3R27 3R28 3R29 3R2A 3R2B 3R2E 3R2F 3R2G 3R2H 3R2I 3R2J 3R2K 3R2L 3R2M 3R2N 3R2O 3R2P 3R2Q 3R2R 3R2S 3R2T 3R2U 3R2V 3R2W 3R2X 3R2Y 3R30 3R31 3R32 3R33 3R34 3R35 3R36 3R37 3R38 3R3A 3R3B 3R3C 3R3D 3R3E 3R3F 3R3G 3R3H 3R3I 3R3J 3R3K 3R3L 3R3M 3R3O 3R3P 3R3Q 3R3R 3R3S 3R3T 3R3U 3R3V 3R3W 3R3X 3R3Y 3R3Z 3R40 3R41 3R42 3R43 3R44 3R45 3R46 3R47 3R48 3R49 3R4A 3R4B 3R4C 3R4D 3R4G 3R4H 3R4I 3R4K 3R4L 3R4M 3R4N 3R4O 3R4P 3R4Q 3R4R 3R4S 3R4T 3R4U 3R4V 3R4X 3R4Y 3R4Z 3R50 3R51 3R52 3R54 3R55 3R56 3R57 3R58 3R59 3R5A 3R5B 3R5C 3R5D 3R5E 3R5F 3R5G 3R5H 3R5I 3R5J 3R5K 3R5L 3R5M 3R5N 3R5O 3R5P 3R5Q 3R5R 3R5S 3R5T 3R5U 3R5V 3R5W 3R5X 3R5Y 3R5Z 3R60 3R61 3R62 3R63 3R64 3R65 3R66 3R67 3R68 3R69 3R6A 3R6B 3R6C 3R6D 3R6E 3R6F 3R6G 3R6H 3R6I 3R6J 3R6K 3R6L 3R6M 3R6N 3R6O 3R6P 3R6Q 3R6S 3R6T 3R6U 3R6V 3R6W 3R6X 3R6Y 3R71 3R72 3R73 3R74 3R75 3R76 3R77 3R79 3R7A 3R7B 3R7C 3R7D 3R7E 3R7F 3R7G 3R7I 3R7K 3R7L 3R7M 3R7N 3R7O 3R7Q 3R7R 3R7S 3R7T 3R7U 3R7V 3R7W 3R7X 3R7Y 3R83 3R84 3R85 3R87 3R88 3R89 3R8A 3R8B 3R8C 3R8D 3R8E 3R8G 3R8H 3R8I 3R8J 3R8K 3R8L 3R8M 3R8P 3R8Q 3R8R 3R8U 3R8V 3R8W 3R8X 3R8Y 3R8Z 3R90 3R91 3R92 3R93 3R94 3R95 3R96 3R97 3R9A 3R9B 3R9C 3R9D 3R9E 3R9F 3R9G 3R9H 3R9I 3R9J 3R9K 3R9L 3R9M 3R9N 3R9O 3R9P 3R9Q 3R9R 3R9S 3R9T 3R9U 3R9V 3R9Y 3R9Z 3RA2 3RA3 3RA5 3RA6 3RA7 3RA8 3RAA 3RAB 3RAC 3RAG 3RAH 3RAI 3RAJ 3RAK 3RAL 3RAM 3RAN 3RAO 3RAP 3RAR 3RAS 3RAT 3RAU 3RAV 3RAW 3RAY 3RAZ 3RB5 3RB7 3RB8 3RB9 3RBA 3RBB 3RBC 3RBF 3RBG 3RBH 3RBI 3RBJ 3RBK 3RBL 3RBM 3RBN 3RBQ 3RBS 3RBT 3RBU 3RBV 3RBW 3RBX 3RBY 3RBZ 3RC0 3RC1 3RC2 3RC3 3RC4 3RC5 3RC6 3RC7 3RC9 3RCB 3RCC 3RCD 3RCE 3RCF 3RCG 3RCH 3RCI 3RCJ 3RCK 3RCL 3RCM 3RCN 3RCO 3RCP 3RCW 3RCY 3RCZ 3RD0 3RD2 3RD3 3RD4 3RD5 3RD6 3RD7 3RD8 3RD9 3RDA 3RDB 3RDC 3RDD 3RDE 3RDH 3RDI 3RDJ 3RDK 3RDM 3RDO 3RDP 3RDQ 3RDR 3RDS 3RDT 3RDU 3RDV 3RDW 3RDX 3RDY 3RDZ 3RE0 3RE1 3RE2 3RE3 3RE4 3RE5 3RE6 3RE7 3RE8 3RE9 3REA 3REB 3RED 3REE 3REF 3REG 3REM 3REN 3REO 3REP 3REQ 3RES 3RET 3REU 3REV 3REW 3REX 3REY 3REZ 3RF0 3RF1 3RF2 3RF3 3RF4 3RF5 3RF6 3RF7 3RF9 3RFA 3RFB 3RFC 3RFE 3RFF 3RFG 3RFH 3RFI 3RFJ 3RFM 3RFN 3RFQ 3RFR 3RFS 3RFT 3RFU 3RFV 3RFW 3RFX 3RFY 3RFZ 3RG0 3RG1 3RG2 3RG3 3RG4 3RG6 3RG8 3RG9 3RGA 3RGB 3RGC 3RGD 3RGE 3RGF 3RGG 3RGH 3RGI 3RGK 3RGL 3RGM 3RGN 3RGO 3RGP 3RGQ 3RGR 3RGS 3RGT 3RGU 3RGV 3RGW 3RGX 3RGY 3RGZ 3RH0 3RH1 3RH2 3RH3 3RH7 3RH8 3RH9 3RHA 3RHB 3RHC 3RHD 3RHE 3RHF 3RHG 3RHH 3RHI 3RHJ 3RHK 3RHL 3RHM 3RHN 3RHO 3RHP 3RHQ 3RHR 3RHS 3RHT 3RHU 3RHW 3RHX 3RHY 3RHZ 3RI0 3RI1 3RI3 3RI5 3RI6 3RI7 3RI8 3RI9 3RIA 3RIB 3RIC 3RID 3RIE 3RIF 3RIG 3RIH 3RII 3RIJ 3RIK 3RIL 3RIM 3RIN 3RIO 3RIP 3RIQ 3RIR 3RIS 3RIT 3RIU 3RIV 3RIW 3RIX 3RIY 3RIZ 3RJ0 3RJ1 3RJ2 3RJ3 3RJ4 3RJ5 3RJ6 3RJ7 3RJ8 3RJ9 3RJA 3RJC 3RJD 3RJL 3RJM 3RJN 3RJO 3RJP 3RJQ 3RJR 3RJS 3RJT 3RJU 3RJV 3RJW 3RJX 3RJY 3RJZ 3RK0 3RK1 3RK2 3RK3 3RK4 3RK5 3RK6 3RK7 3RK8 3RK9 3RKB 3RKC 3RKD 3RKE 3RKG 3RKH 3RKI 3RKJ 3RKK 3RKL 3RKO 3RKP 3RKR 3RKS 3RKT 3RKU 3RKV 3RKW 3RKX 3RKY 3RKZ 3RL0 3RL1 3RL2 3RL3 3RL4 3RL5 3RL6 3RL7 3RL8 3RL9 3RLA 3RLB 3RLC 3RLD 3RLE 3RLF 3RLG 3RLH 3RLI 3RLJ 3RLK 3RLL 3RLM 3RLN 3RLO 3RLP 3RLQ 3RLR 3RLS 3RLU 3RLV 3RLW 3RLY 3RLZ 3RM0 3RM1 3RM2 3RM3 3RM4 3RM5 3RM6 3RM7 3RM8 3RM9 3RME 3RMF 3RMG 3RMH 3RMI 3RMJ 3RMK 3RML 3RMM 3RMN 3RMO 3RMQ 3RMR 3RMS 3RMT 3RMU 3RMV 3RMW 3RMX 3RMY 3RMZ 3RN0 3RN1 3RN3 3RN4 3RN6 3RN8 3RN9 3RNA 3RNB 3RNC 3RND 3RNE 3RNF 3RNG 3RNI 3RNJ 3RNK 3RNL 3RNM 3RNN 3RNO 3RNQ 3RNR 3RNS 3RNT 3RNV 3RNX 3RNY 3RNZ 3RO0 3RO1 3RO2 3RO3 3RO4 3RO5 3RO6 3RO7 3RO8 3RO9 3ROA 3ROB 3ROC 3ROD 3ROE 3ROF 3ROG 3ROH 3ROI 3ROJ 3ROK 3ROL 3ROM 3RON 3ROO 3ROP 3ROQ 3ROR 3ROS 3ROT 3ROU 3ROV 3ROW 3ROX 3ROY 3ROZ 3RP1 3RP2 3RP6 3RP7 3RP8 3RP9 3RPC 3RPD 3RPE 3RPF 3RPG 3RPH 3RPI 3RPJ 3RPK 3RPL 3RPM 3RPN 3RPO 3RPP 3RPQ 3RPR 3RPS 3RPT 3RPU 3RPV 3RPW 3RPX 3RPY 3RPZ 3RQ0 3RQ1 3RQ2 3RQ3 3RQ4 3RQ5 3RQ6 3RQ7 3RQ8 3RQ9 3RQA 3RQB 3RQC 3RQD 3RQE 3RQF 3RQG 3RQH 3RQI 3RQJ 3RQK 3RQL 3RQM 3RQN 3RQO 3RQP 3RQQ 3RQR 3RQS 3RQT 3RQU 3RQW 3RQX 3RQZ 3RR1 3RR2 3RR3 3RR4 3RR5 3RR6 3RRA 3RRB 3RRC 3RRD 3RRE 3RRF 3RRI 3RRJ 3RRK 3RRL 3RRM 3RRN 3RRO 3RRP 3RRQ 3RRR 3RRS 3RRT 3RRU 3RRV 3RRW 3RRX 3RRY 3RRZ 3RS0 3RS1 3RS2 3RS3 3RS4 3RS5 3RS6 3RS7 3RS8 3RS9 3RSB 3RSC 3RSD 3RSE 3RSF 3RSG 3RSH 3RSI 3RSJ 3RSK 3RSL 3RSM 3RSN 3RSO 3RSP 3RSQ 3RSR 3RSS 3RST 3RSV 3RSW 3RSX 3RSY 3RSZ 3RT0 3RT1 3RT2 3RT3 3RT4 3RT5 3RT6 3RT7 3RT8 3RT9 3RTA 3RTB 3RTC 3RTD 3RTE 3RTF 3RTG 3RTH 3RTI 3RTK 3RTL 3RTM 3RTN 3RTO 3RTP 3RTQ 3RTR 3RTS 3RTT 3RTW 3RTX 3RTY 3RU0 3RU1 3RU2 3RU3 3RU4 3RU5 3RU6 3RU7 3RU8 3RU9 3RUA 3RUB 3RUC 3RUD 3RUE 3RUF 3RUG 3RUH 3RUI 3RUJ 3RUK 3RUL 3RUM 3RUN 3RUO 3RUP 3RUQ 3RUR 3RUS 3RUT 3RUU 3RUV 3RUW 3RUX 3RUY 3RUZ 3RV0 3RV1 3RV2 3RV3 3RV4 3RV5 3RV6 3RV7 3RV8 3RV9 3RVA 3RVB 3RVC 3RVD 3RVF 3RVG 3RVH 3RVI 3RVJ 3RVK 3RVL 3RVM 3RVN 3RVO 3RVP 3RVQ 3RVR 3RVS 3RVT 3RVU 3RVV 3RVW 3RVX 3RVY 3RVZ 3RW0 3RW7 3RW8 3RW9 3RWA 3RWB 3RWC 3RWD 3RWE 3RWF 3RWG 3RWH 3RWI 3RWJ 3RWK 3RWL 3RWM 3RWN 3RWO 3RWP 3RWQ 3RWR 3RWT 3RWV 3RWX 3RX2 3RX3 3RX4 3RX5 3RX6 3RX7 3RX8 3RX9 3RXA 3RXB 3RXC 3RXD 3RXE 3RXF 3RXG 3RXH 3RXI 3RXJ 3RXK 3RXL 3RXM 3RXO 3RXP 3RXQ 3RXR 3RXS 3RXT 3RXU 3RXV 3RXW 3RXX 3RXY 3RXZ 3RY0 3RY1 3RY2 3RY3 3RY4 3RY5 3RY6 3RY7 3RY8 3RY9 3RYA 3RYB 3RYC 3RYD 3RYE 3RYF 3RYH 3RYI 3RYJ 3RYK 3RYL 3RYM 3RYO 3RYP 3RYR 3RYS 3RYT 3RYV 3RYW 3RYX 3RYY 3RYZ 3RZ0 3RZ1 3RZ2 3RZ3 3RZ4 3RZ5 3RZ7 3RZ8 3RZ9 3RZA 3RZB 3RZC 3RZE 3RZF 3RZI 3RZN 3RZP 3RZS 3RZU 3RZV 3RZW 3RZX 3RZY 3RZZ 3S00 3S01 3S02 3S03 3S04 3S05 3S06 3S0A 3S0B 3S0C 3S0D 3S0E 3S0F 3S0G 3S0H 3S0I 3S0J 3S0K 3S0M 3S0N 3S0O 3S0P 3S0Q 3S0R 3S0T 3S0W 3S0X 3S0Y 3S0Z 3S11 3S12 3S13 3S18 3S19 3S1A 3S1B 3S1C 3S1D 3S1E 3S1F 3S1G 3S1H 3S1I 3S1J 3S1K 3S1L 3S1S 3S1T 3S1U 3S1V 3S1W 3S1X 3S1Y 3S1Z 3S20 3S21 3S22 3S23 3S24 3S25 3S26 3S27 3S28 3S29 3S2A 3S2C 3S2E 3S2F 3S2G 3S2I 3S2J 3S2K 3S2L 3S2M 3S2N 3S2O 3S2P 3S2Q 3S2R 3S2S 3S2U 3S2V 3S2W 3S2X 3S2Y 3S2Z 3S30 3S32 3S33 3S34 3S35 3S36 3S37 3S38 3S39 3S3A 3S3B 3S3C 3S3D 3S3E 3S3F 3S3G 3S3H 3S3I 3S3J 3S3K 3S3L 3S3P 3S3Q 3S3R 3S3S 3S3T 3S3U 3S3V 3S3W 3S3X 3S3Y 3S3Z 3S40 3S41 3S42 3S43 3S44 3S45 3S46 3S47 3S48 3S4A 3S4B 3S4C 3S4D 3S4E 3S4F 3S4J 3S4K 3S4L 3S4M 3S4O 3S4Q 3S4R 3S4S 3S4T 3S4U 3S4W 3S4X 3S4Y 3S4Z 3S51 3S52 3S53 3S54 3S55 3S56 3S5B 3S5C 3S5D 3S5E 3S5F 3S5H 3S5I 3S5J 3S5K 3S5L 3S5M 3S5N 3S5O 3S5P 3S5Q 3S5R 3S5S 3S5T 3S5U 3S5V 3S5W 3S5X 3S5Y 3S5Z 3S60 3S61 3S62 3S63 3S64 3S65 3S66 3S67 3S68 3S69 3S6A 3S6B 3S6C 3S6D 3S6E 3S6F 3S6G 3S6H 3S6J 3S6K 3S6L 3S6M 3S6N 3S6O 3S6P 3S6S 3S6T 3S6U 3S6V 3S6W 3S6X 3S6Y 3S6Z 3S70 3S71 3S72 3S73 3S74 3S75 3S76 3S77 3S78 3S79 3S7A 3S7B 3S7D 3S7E 3S7F 3S7G 3S7H 3S7I 3S7J 3S7K 3S7L 3S7M 3S7N 3S7O 3S7P 3S7Q 3S7R 3S7S 3S7T 3S7V 3S7W 3S7X 3S7Y 3S7Z 3S81 3S82 3S83 3S84 3S85 3S86 3S87 3S88 3S8A 3S8B 3S8C 3S8D 3S8E 3S8F 3S8G 3S8H 3S8I 3S8J 3S8K 3S8L 3S8M 3S8N 3S8O 3S8P 3S8R 3S8S 3S8V 3S8W 3S8X 3S8Y 3S8Z 3S90 3S91 3S92 3S93 3S94 3S95 3S96 3S97 3S98 3S99 3S9A 3S9B 3S9C 3S9D 3S9E 3S9F 3S9G 3S9I 3S9J 3S9K 3S9L 3S9M 3S9N 3S9O 3S9Q 3S9S 3S9T 3S9U 3S9V 3S9W 3S9X 3S9Y 3S9Z 3SA0 3SA1 3SA2 3SA3 3SA4 3SA5 3SA6 3SA7 3SA8 3SA9 3SAA 3SAB 3SAC 3SAD 3SAE 3SAF 3SAG 3SAH 3SAI 3SAJ 3SAL 3SAM 3SAN 3SAO 3SAP 3SAQ 3SAX 3SAY 3SAZ 3SB0 3SB1 3SB2 3SB3 3SB4 3SB5 3SB6 3SB7 3SB8 3SB9 3SBA 3SBB 3SBC 3SBD 3SBE 3SBF 3SBG 3SBH 3SBI 3SBK 3SBL 3SBM 3SBN 3SBO 3SBP 3SBQ 3SBR 3SBS 3SBT 3SBU 3SBW 3SBX 3SBY 3SBZ 3SC0 3SC1 3SC2 3SC3 3SC4 3SC6 3SC7 3SCE 3SCF 3SCG 3SCH 3SCI 3SCJ 3SCK 3SCL 3SCM 3SCN 3SCO 3SCP 3SCQ 3SCR 3SCS 3SCT 3SCU 3SCV 3SCW 3SCY 3SCZ 3SD0 3SD2 3SD4 3SD5 3SD6 3SD7 3SD9 3SDA 3SDB 3SDC 3SDD 3SDE 3SDF 3SDG 3SDH 3SDI 3SDJ 3SDK 3SDL 3SDM 3SDN 3SDO 3SDP 3SDQ 3SDR 3SDS 3SDT 3SDU 3SDV 3SDW 3SDX 3SDY 3SDZ 3SE0 3SE1 3SE2 3SE3 3SE4 3SE5 3SE6 3SE7 3SE8 3SE9 3SEA 3SEB 3SEC 3SED 3SEE 3SEF 3SEI 3SEJ 3SEK 3SEL 3SEM 3SEN 3SEO 3SEP 3SEQ 3SER 3SES 3SET 3SEU 3SEV 3SEW 3SEX 3SEY 3SEZ 3SF0 3SF4 3SF5 3SF6 3SF8 3SFC 3SFD 3SFE 3SFF 3SFG 3SFH 3SFI 3SFJ 3SFK 3SFM 3SFP 3SFT 3SFU 3SFV 3SFW 3SFX 3SFY 3SFZ 3SG0 3SG1 3SG2 3SG3 3SG4 3SG5 3SG6 3SG7 3SG8 3SG9 3SGA 3SGB 3SGC 3SGD 3SGE 3SGG 3SGH 3SGI 3SGJ 3SGK 3SGL 3SGM 3SGN 3SGO 3SGP 3SGQ 3SGR 3SGS 3SGT 3SGU 3SGV 3SGW 3SGX 3SGY 3SGZ 3SH0 3SH1 3SH2 3SH3 3SH4 3SH5 3SH6 3SH7 3SH8 3SH9 3SHA 3SHB 3SHC 3SHD 3SHE 3SHF 3SHG 3SHI 3SHJ 3SHL 3SHM 3SHO 3SHP 3SHQ 3SHR 3SHS 3SHT 3SHU 3SHV 3SHW 3SHX 3SHY 3SHZ 3SI0 3SI1 3SI2 3SI3 3SI4 3SI5 3SI7 3SI9 3SIA 3SIB 3SIC 3SID 3SIE 3SIG 3SIH 3SII 3SIJ 3SIK 3SIL 3SIM 3SIO 3SIP 3SIQ 3SIR 3SIS 3SIT 3SIW 3SIX 3SIY 3SIZ 3SJ0 3SJ1 3SJ3 3SJ4 3SJ5 3SJ6 3SJ7 3SJ8 3SJ9 3SJA 3SJB 3SJC 3SJD 3SJE 3SJF 3SJG 3SJH 3SJI 3SJK 3SJL 3SJN 3SJO 3SJP 3SJQ 3SJR 3SJS 3SJT 3SJU 3SJV 3SJX 3SJZ 3SK0 3SK1 3SK2 3SK3 3SK4 3SK5 3SK6 3SK7 3SK8 3SK9 3SKA 3SKB 3SKC 3SKD 3SKE 3SKF 3SKG 3SKH 3SKJ 3SKK 3SKM 3SKN 3SKO 3SKP 3SKQ 3SKS 3SKU 3SKV 3SKX 3SKY 3SL0 3SL1 3SL2 3SL3 3SL4 3SL5 3SL6 3SL7 3SL8 3SL9 3SLA 3SLB 3SLC 3SLD 3SLE 3SLF 3SLG 3SLH 3SLI 3SLJ 3SLK 3SLL 3SLN 3SLO 3SLR 3SLS 3SLT 3SLU 3SLZ 3SM0 3SM1 3SM2 3SM3 3SM5 3SM8 3SM9 3SMA 3SMB 3SMC 3SMD 3SME 3SMH 3SMI 3SMJ 3SMK 3SML 3SMM 3SMN 3SMO 3SMP 3SMQ 3SMR 3SMS 3SMT 3SMV 3SMZ 3SN0 3SN1 3SN4 3SN5 3SN6 3SN7 3SN8 3SN9 3SNA 3SNB 3SNC 3SND 3SNE 3SNF 3SNG 3SNH 3SNI 3SNK 3SNL 3SNM 3SNO 3SNS 3SNV 3SNX 3SNY 3SNZ 3SO0 3SO1 3SO2 3SO3 3SO4 3SO5 3SO6 3SO7 3SO8 3SO9 3SOA 3SOB 3SOC 3SOD 3SOE 3SOG 3SOH 3SOI 3SOJ 3SOK 3SOL 3SOM 3SON 3SOO 3SOP 3SOQ 3SOR 3SOS 3SOT 3SOU 3SOV 3SOW 3SOX 3SOY 3SOZ 3SP1 3SP3 3SP4 3SP6 3SP7 3SP8 3SP9 3SPA 3SPB 3SPC 3SPE 3SPF 3SPG 3SPH 3SPI 3SPJ 3SPK 3SPR 3SPS 3SPT 3SPU 3SPV 3SPW 3SPX 3SQ3 3SQ5 3SQ6 3SQ7 3SQ8 3SQ9 3SQB 3SQC 3SQD 3SQE 3SQF 3SQG 3SQH 3SQJ 3SQL 3SQM 3SQN 3SQO 3SQP 3SQQ 3SQR 3SQS 3SQV 3SQY 3SQZ 3SR0 3SR1 3SR2 3SR3 3SR4 3SR5 3SR6 3SR7 3SR9 3SRA 3SRB 3SRC 3SRD 3SRE 3SRF 3SRG 3SRH 3SRI 3SRJ 3SRK 3SRN 3SRP 3SRQ 3SRR 3SRS 3SRT 3SRU 3SRV 3SRW 3SRX 3SRY 3SRZ 3SS0 3SS1 3SS3 3SS4 3SS5 3SS6 3SS7 3SS8 3SS9 3SSA 3SSB 3SSG 3SSH 3SSI 3SSJ 3SSK 3SSL 3SSM 3SSN 3SSO 3SSP 3SSQ 3SSR 3SSS 3SST 3SSU 3SSV 3SSW 3SSX 3SSY 3SSZ 3ST0 3ST1 3ST2 3ST3 3ST4 3ST5 3ST6 3ST7 3ST8 3ST9 3STA 3STB 3STC 3STD 3STE 3STF 3STG 3STH 3STI 3STJ 3STK 3STL 3STM 3STN 3STO 3STP 3STQ 3STR 3STT 3STU 3STV 3STW 3STX 3STY 3STZ 3SU0 3SU1 3SU2 3SU3 3SU4 3SU5 3SU6 3SU8 3SU9 3SUA 3SUB 3SUC 3SUD 3SUE 3SUF 3SUG 3SUI 3SUJ 3SUK 3SUL 3SUM 3SUR 3SUS 3SUT 3SUU 3SUV 3SUW 3SUZ 3SV0 3SV1 3SV2 3SV5 3SV6 3SV7 3SV8 3SV9 3SVA 3SVB 3SVC 3SVD 3SVE 3SVF 3SVG 3SVH 3SVI 3SVJ 3SVK 3SVL 3SVM 3SVN 3SVO 3SVP 3SVQ 3SVR 3SVS 3SVT 3SVU 3SVV 3SVW 3SVZ 3SW0 3SW1 3SW2 3SW3 3SW4 3SW5 3SW6 3SW7 3SW8 3SW9 3SWA 3SWB 3SWC 3SWD 3SWE 3SWF 3SWG 3SWH 3SWI 3SWJ 3SWK 3SWL 3SWN 3SWO 3SWQ 3SWR 3SWS 3SWT 3SWV 3SWW 3SWX 3SWY 3SWZ 3SX0 3SX1 3SX2 3SX3 3SX4 3SX5 3SX6 3SX7 3SX8 3SX9 3SXA 3SXB 3SXC 3SXD 3SXE 3SXF 3SXG 3SXH 3SXI 3SXJ 3SXK 3SXL 3SXM 3SXN 3SXO 3SXP 3SXQ 3SXR 3SXS 3SXT 3SXU 3SXV 3SXW 3SXX 3SXY 3SXZ 3SY0 3SY1 3SY2 3SY3 3SY4 3SY5 3SY6 3SY7 3SY8 3SY9 3SYA 3SYB 3SYC 3SYI 3SYJ 3SYK 3SYL 3SYM 3SYN 3SYO 3SYP 3SYQ 3SYR 3SYS 3SYT 3SYU 3SYV 3SYX 3SYY 3SZ0 3SZ1 3SZ3 3SZ4 3SZ6 3SZ7 3SZ8 3SZ9 3SZA 3SZB 3SZC 3SZD 3SZE 3SZF 3SZG 3SZH 3SZI 3SZJ 3SZK 3SZL 3SZM 3SZN 3SZO 3SZP 3SZR 3SZS 3SZT 3SZU 3SZV 3SZW 3SZY 3SZZ 3T00 3T01 3T02 3T03 3T04 3T05 3T06 3T07 3T08 3T09 3T0A 3T0B 3T0C 3T0D 3T0E 3T0F 3T0G 3T0H 3T0I 3T0J 3T0K 3T0L 3T0M 3T0O 3T0P 3T0Q 3T0R 3T0S 3T0T 3T0U 3T0V 3T0W 3T0X 3T0Y 3T0Z 3T10 3T11 3T12 3T13 3T14 3T15 3T16 3T19 3T1A 3T1B 3T1C 3T1D 3T1E 3T1F 3T1G 3T1I 3T1K 3T1L 3T1M 3T1N 3T1O 3T1P 3T1Q 3T1R 3T1S 3T1T 3T1U 3T1V 3T1W 3T1X 3T20 3T22 3T24 3T25 3T26 3T27 3T28 3T29 3T2A 3T2B 3T2C 3T2D 3T2E 3T2F 3T2G 3T2H 3T2I 3T2J 3T2K 3T2L 3T2M 3T2N 3T2O 3T2P 3T2Q 3T2S 3T2T 3T2V 3T2W 3T2X 3T2Y 3T2Z 3T30 3T31 3T32 3T33 3T34 3T35 3T36 3T37 3T38 3T39 3T3A 3T3C 3T3D 3T3E 3T3G 3T3H 3T3I 3T3J 3T3K 3T3L 3T3M 3T3P 3T3Q 3T3R 3T3S 3T3T 3T3U 3T3V 3T3W 3T3X 3T3Y 3T3Z 3T40 3T41 3T42 3T43 3T44 3T45 3T46 3T47 3T48 3T49 3T4A 3T4C 3T4D 3T4E 3T4F 3T4G 3T4H 3T4J 3T4K 3T4L 3T4M 3T4N 3T4O 3T4P 3T4Q 3T4R 3T4S 3T4T 3T4U 3T4V 3T4W 3T4X 3T4Y 3T4Z 3T50 3T51 3T52 3T53 3T54 3T55 3T56 3T57 3T58 3T59 3T5A 3T5B 3T5C 3T5D 3T5F 3T5G 3T5I 3T5M 3T5O 3T5P 3T5S 3T5T 3T5U 3T5V 3T5W 3T5X 3T5Y 3T5Z 3T60 3T61 3T62 3T63 3T64 3T65 3T66 3T67 3T69 3T6A 3T6B 3T6C 3T6D 3T6E 3T6F 3T6G 3T6H 3T6I 3T6J 3T6K 3T6L 3T6N 3T6O 3T6P 3T6Q 3T6R 3T6S 3T6U 3T6V 3T6W 3T6X 3T6Y 3T6Z 3T70 3T71 3T73 3T74 3T77 3T78 3T7A 3T7B 3T7C 3T7D 3T7E 3T7F 3T7G 3T7H 3T7I 3T7J 3T7K 3T7L 3T7M 3T7N 3T7O 3T7P 3T7Q 3T7R 3T7S 3T7T 3T7U 3T7V 3T7X 3T7Y 3T7Z 3T80 3T81 3T82 3T83 3T84 3T85 3T87 3T88 3T89 3T8A 3T8B 3T8C 3T8D 3T8E 3T8F 3T8G 3T8H 3T8I 3T8J 3T8K 3T8L 3T8M 3T8N 3T8O 3T8Q 3T8R 3T8S 3T8T 3T8U 3T8V 3T8W 3T8X 3T8Y 3T90 3T91 3T92 3T93 3T94 3T95 3T96 3T97 3T98 3T99 3T9A 3T9B 3T9C 3T9D 3T9E 3T9F 3T9G 3T9H 3T9I 3T9J 3T9K 3T9L 3T9M 3T9N 3T9O 3T9P 3T9Q 3T9T 3T9U 3T9V 3T9W 3T9X 3T9Y 3T9Z 3TA0 3TA1 3TA2 3TA3 3TA4 3TA5 3TA6 3TA7 3TA8 3TA9 3TAC 3TAD 3TAH 3TAI 3TAJ 3TAK 3TAL 3TAM 3TAO 3TAS 3TAT 3TAU 3TAV 3TAW 3TAX 3TAY 3TAZ 3TB0 3TB2 3TB3 3TB4 3TB5 3TB6 3TB7 3TB8 3TB9 3TBA 3TBB 3TBC 3TBD 3TBE 3TBF 3TBG 3TBH 3TBI 3TBJ 3TBK 3TBL 3TBM 3TBN 3TBO 3TBS 3TBT 3TBV 3TBW 3TBY 3TC1 3TC2 3TC3 3TC5 3TC6 3TC7 3TC8 3TC9 3TCA 3TCE 3TCF 3TCG 3TCH 3TCJ 3TCK 3TCL 3TCM 3TCN 3TCO 3TCP 3TCQ 3TCR 3TCS 3TCT 3TCU 3TCV 3TCX 3TCY 3TCZ 3TD2 3TD3 3TD4 3TD5 3TD6 3TD7 3TD8 3TD9 3TDA 3TDB 3TDC 3TDD 3TDE 3TDF 3TDG 3TDH 3TDI 3TDJ 3TDK 3TDL 3TDM 3TDN 3TDO 3TDP 3TDQ 3TDR 3TDS 3TDT 3TDU 3TDV 3TDW 3TDX 3TDZ 3TE0 3TE1 3TE2 3TE3 3TE4 3TE5 3TE6 3TE7 3TE8 3TE9 3TEA 3TEB 3TEC 3TEE 3TEF 3TEG 3TEH 3TEI 3TEJ 3TEK 3TEL 3TEM 3TEN 3TEO 3TEP 3TEQ 3TER 3TES 3TET 3TEU 3TEV 3TEW 3TEX 3TEY 3TEZ 3TF0 3TF1 3TF2 3TF3 3TF4 3TF5 3TF6 3TF7 3TF8 3TF9 3TFA 3TFB 3TFC 3TFD 3TFE 3TFF 3TFG 3TFH 3TFI 3TFJ 3TFK 3TFL 3TFM 3TFN 3TFO 3TFP 3TFQ 3TFT 3TFU 3TFV 3TFW 3TFX 3TFY 3TFZ 3TG0 3TG1 3TG2 3TG3 3TG4 3TG5 3TG6 3TG7 3TG8 3TG9 3TGA 3TGB 3TGC 3TGD 3TGE 3TGG 3TGH 3TGI 3TGJ 3TGK 3TGL 3TGM 3TGN 3TGO 3TGP 3TGQ 3TGR 3TGS 3TGT 3TGU 3TGV 3TGW 3TGX 3TGY 3TGZ 3TH0 3TH1 3TH2 3TH3 3TH4 3TH5 3TH6 3TH7 3TH8 3TH9 3THA 3THB 3THC 3THD 3THE 3THF 3THG 3THH 3THI 3THJ 3THK 3THM 3THN 3THO 3THP 3THQ 3THR 3THS 3THT 3THU 3TI1 3TI2 3TI3 3TI4 3TI5 3TI6 3TI7 3TI8 3TI9 3TIA 3TIB 3TIC 3TID 3TIE 3TIF 3TIG 3TIH 3TII 3TIJ 3TIK 3TIM 3TIN 3TIO 3TIP 3TIQ 3TIR 3TIS 3TIT 3TIU 3TIV 3TIW 3TIX 3TIY 3TIZ 3TJ0 3TJ1 3TJ2 3TJ3 3TJ4 3TJ5 3TJ6 3TJ7 3TJ8 3TJ9 3TJA 3TJB 3TJC 3TJD 3TJE 3TJF 3TJG 3TJH 3TJI 3TJJ 3TJK 3TJL 3TJM 3TJN 3TJO 3TJP 3TJQ 3TJR 3TJS 3TJT 3TJU 3TJV 3TJW 3TJX 3TJY 3TJZ 3TK0 3TK1 3TK2 3TK3 3TK4 3TK5 3TK6 3TK7 3TK8 3TK9 3TKA 3TKB 3TKC 3TKD 3TKF 3TKG 3TKH 3TKI 3TKJ 3TKK 3TKL 3TKM 3TKN 3TKP 3TKQ 3TKR 3TKS 3TKT 3TKU 3TKW 3TKY 3TKZ 3TL0 3TL1 3TL2 3TL3 3TL4 3TL5 3TL6 3TL8 3TL9 3TLA 3TLB 3TLC 3TLD 3TLE 3TLF 3TLG 3TLH 3TLI 3TLJ 3TLK 3TLL 3TLM 3TLO 3TLP 3TLQ 3TLR 3TLS 3TLT 3TLU 3TLV 3TLW 3TLX 3TLY 3TLZ 3TM0 3TM1 3TM2 3TM3 3TM4 3TM5 3TM6 3TM7 3TM8 3TM9 3TMA 3TMB 3TMC 3TMD 3TME 3TMG 3TMH 3TMK 3TML 3TMN 3TMO 3TMP 3TMQ 3TMR 3TMS 3TMT 3TMU 3TMV 3TMW 3TMX 3TMY 3TMZ 3TN0 3TN2 3TN3 3TN4 3TN5 3TN6 3TN7 3TN8 3TN9 3TNB 3TND 3TNE 3TNF 3TNG 3TNH 3TNI 3TNJ 3TNL 3TNM 3TNN 3TNO 3TNP 3TNQ 3TNS 3TNT 3TNU 3TNV 3TNW 3TNX 3TNY 3TNZ 3TO0 3TO1 3TO2 3TO3 3TO4 3TO5 3TO6 3TO7 3TO8 3TO9 3TOA 3TOB 3TOC 3TOD 3TOE 3TOF 3TOG 3TOH 3TOI 3TOJ 3TOL 3TOM 3TON 3TOP 3TOQ 3TOR 3TOS 3TOT 3TOU 3TOV 3TOW 3TOX 3TOY 3TOZ 3TP0 3TP1 3TP2 3TP3 3TP4 3TP5 3TP6 3TP7 3TP8 3TP9 3TPA 3TPB 3TPC 3TPD 3TPE 3TPF 3TPI 3TPJ 3TPK 3TPL 3TPM 3TPN 3TPO 3TPP 3TPQ 3TPR 3TPS 3TPT 3TPU 3TPV 3TPW 3TPX 3TPY 3TPZ 3TQ0 3TQ2 3TQ3 3TQ4 3TQ5 3TQ7 3TQ8 3TQ9 3TQA 3TQB 3TQC 3TQD 3TQE 3TQF 3TQG 3TQH 3TQI 3TQJ 3TQK 3TQL 3TQM 3TQN 3TQO 3TQP 3TQQ 3TQR 3TQS 3TQT 3TQU 3TQV 3TQW 3TQX 3TQY 3TQZ 3TR0 3TR1 3TR2 3TR3 3TR4 3TR5 3TR6 3TR7 3TR8 3TR9 3TRB 3TRC 3TRD 3TRE 3TRF 3TRG 3TRH 3TRI 3TRJ 3TRK 3TRL 3TRN 3TRO 3TRP 3TRQ 3TRR 3TRS 3TRT 3TRU 3TRV 3TRW 3TRY 3TS1 3TS3 3TS4 3TS5 3TS6 3TS7 3TS9 3TSA 3TSB 3TSC 3TSD 3TSG 3TSH 3TSI 3TSJ 3TSK 3TSL 3TSM 3TSN 3TSO 3TSP 3TSQ 3TSR 3TSS 3TSU 3TSV 3TSW 3TSY 3TSZ 3TT0 3TT1 3TT2 3TT3 3TT4 3TT6 3TT7 3TT8 3TT9 3TTA 3TTB 3TTC 3TTD 3TTE 3TTF 3TTG 3TTH 3TTI 3TTJ 3TTK 3TTL 3TTM 3TTN 3TTO 3TTP 3TTQ 3TTR 3TTS 3TTT 3TTU 3TTV 3TTW 3TTX 3TTY 3TTZ 3TU0 3TU1 3TU3 3TU5 3TU6 3TU7 3TU8 3TU9 3TUA 3TUB 3TUC 3TUD 3TUE 3TUF 3TUG 3TUH 3TUI 3TUJ 3TUL 3TUO 3TUR 3TUS 3TUT 3TUU 3TUV 3TUW 3TUX 3TUY 3TUZ 3TV0 3TV1 3TV2 3TV3 3TV4 3TV5 3TV6 3TV7 3TV8 3TV9 3TVA 3TVC 3TVD 3TVI 3TVJ 3TVK 3TVL 3TVM 3TVN 3TVO 3TVQ 3TVR 3TVT 3TVU 3TVV 3TVW 3TVX 3TVY 3TVZ 3TW0 3TW1 3TW2 3TW3 3TW4 3TW5 3TW6 3TW7 3TW8 3TW9 3TWA 3TWB 3TWC 3TWD 3TWE 3TWF 3TWG 3TWI 3TWJ 3TWK 3TWL 3TWO 3TWP 3TWQ 3TWR 3TWS 3TWT 3TWU 3TWV 3TWW 3TWX 3TWY 3TWZ 3TX0 3TX1 3TX2 3TX3 3TX4 3TX6 3TX7 3TX8 3TX9 3TXA 3TXB 3TXD 3TXE 3TXF 3TXG 3TXH 3TXI 3TXJ 3TXK 3TXM 3TXN 3TXO 3TXQ 3TXS 3TXT 3TXV 3TXX 3TXY 3TXZ 3TY0 3TY1 3TY2 3TY3 3TY4 3TY5 3TY6 3TY7 3TY8 3TY9 3TYA 3TYB 3TYC 3TYD 3TYE 3TYF 3TYG 3TYH 3TYI 3TYJ 3TYK 3TYL 3TYM 3TYN 3TYO 3TYP 3TYQ 3TYR 3TYS 3TYT 3TYU 3TYV 3TYW 3TYX 3TYY 3TYZ 3TZ0 3TZ1 3TZ2 3TZ3 3TZ4 3TZ5 3TZ6 3TZ7 3TZ8 3TZ9 3TZA 3TZB 3TZC 3TZD 3TZE 3TZF 3TZG 3TZH 3TZI 3TZK 3TZL 3TZM 3TZN 3TZO 3TZQ 3TZS 3TZT 3TZU 3TZV 3TZW 3TZX 3TZY 3TZZ 3U00 3U01 3U02 3U03 3U04 3U06 3U07 3U09 3U0A 3U0B 3U0C 3U0D 3U0E 3U0F 3U0G 3U0H 3U0I 3U0J 3U0K 3U0L 3U0M 3U0N 3U0O 3U0P 3U0R 3U0S 3U0T 3U0V 3U0W 3U0X 3U0Y 3U0Z 3U10 3U11 3U12 3U13 3U14 3U15 3U16 3U17 3U18 3U19 3U1A 3U1B 3U1C 3U1D 3U1H 3U1I 3U1J 3U1K 3U1L 3U1M 3U1N 3U1O 3U1P 3U1Q 3U1R 3U1S 3U1T 3U1U 3U1V 3U1W 3U1X 3U1Y 3U21 3U22 3U23 3U24 3U25 3U26 3U27 3U28 3U29 3U2A 3U2C 3U2D 3U2F 3U2G 3U2H 3U2I 3U2K 3U2L 3U2M 3U2O 3U2P 3U2Q 3U2R 3U2S 3U2T 3U2U 3U2V 3U2W 3U2X 3U2Y 3U2Z 3U30 3U31 3U32 3U33 3U34 3U35 3U36 3U37 3U39 3U3A 3U3B 3U3D 3U3E 3U3F 3U3G 3U3H 3U3I 3U3J 3U3K 3U3L 3U3M 3U3N 3U3O 3U3P 3U3Q 3U3R 3U3S 3U3T 3U3U 3U3V 3U3X 3U3Z 3U40 3U41 3U43 3U45 3U46 3U47 3U48 3U49 3U4A 3U4B 3U4C 3U4D 3U4E 3U4F 3U4G 3U4H 3U4I 3U4J 3U4K 3U4L 3U4N 3U4O 3U4R 3U4S 3U4T 3U4U 3U4V 3U4W 3U4X 3U4Y 3U4Z 3U50 3U51 3U52 3U53 3U54 3U55 3U57 3U59 3U5J 3U5K 3U5L 3U5M 3U5N 3U5O 3U5P 3U5R 3U5S 3U5T 3U5U 3U5V 3U5W 3U5Y 3U62 3U64 3U65 3U66 3U67 3U69 3U6A 3U6B 3U6G 3U6H 3U6I 3U6J 3U6K 3U6N 3U6R 3U6T 3U6U 3U6V 3U6W 3U6X 3U6Z 3U70 3U71 3U72 3U73 3U74 3U75 3U78 3U79 3U7A 3U7B 3U7C 3U7D 3U7E 3U7I 3U7J 3U7K 3U7L 3U7M 3U7N 3U7Q 3U7R 3U7S 3U7T 3U7U 3U7V 3U7W 3U7X 3U7Y 3U7Z 3U80 3U81 3U82 3U83 3U84 3U85 3U86 3U87 3U88 3U8A 3U8B 3U8C 3U8D 3U8E 3U8F 3U8G 3U8H 3U8I 3U8J 3U8K 3U8L 3U8M 3U8N 3U8O 3U8P 3U8Q 3U8R 3U8T 3U8U 3U8V 3U8W 3U8X 3U8Z 3U90 3U91 3U92 3U93 3U94 3U95 3U96 3U97 3U98 3U99 3U9A 3U9B 3U9C 3U9D 3U9E 3U9F 3U9G 3U9H 3U9I 3U9J 3U9L 3U9M 3U9N 3U9O 3U9P 3U9Q 3U9R 3U9S 3U9T 3U9U 3U9W 3U9X 3U9Y 3U9Z 3UA0 3UA1 3UA3 3UA4 3UA5 3UA6 3UA7 3UA8 3UA9 3UAA 3UAB 3UAC 3UAD 3UAE 3UAF 3UAG 3UAH 3UAI 3UAJ 3UAK 3UAL 3UAM 3UAN 3UAO 3UAP 3UAQ 3UAR 3UAS 3UAT 3UAU 3UAV 3UAW 3UAX 3UAY 3UAZ 3UB0 3UB1 3UB2 3UB3 3UB4 3UB5 3UB6 3UB7 3UB8 3UB9 3UBA 3UBB 3UBC 3UBD 3UBE 3UBF 3UBG 3UBH 3UBJ 3UBK 3UBL 3UBM 3UBN 3UBO 3UBP 3UBQ 3UBR 3UBU 3UBV 3UBW 3UBX 3UC0 3UC1 3UC2 3UC3 3UC4 3UC5 3UC7 3UC8 3UC9 3UCA 3UCB 3UCC 3UCD 3UCE 3UCF 3UCG 3UCH 3UCI 3UCJ 3UCK 3UCL 3UCM 3UCN 3UCO 3UCP 3UCQ 3UCR 3UCS 3UCT 3UCW 3UCX 3UCY 3UD0 3UD1 3UD2 3UD5 3UD6 3UD7 3UD8 3UD9 3UDA 3UDB 3UDC 3UDD 3UDE 3UDF 3UDH 3UDI 3UDJ 3UDK 3UDL 3UDM 3UDN 3UDO 3UDP 3UDQ 3UDR 3UDS 3UDT 3UDU 3UDV 3UDW 3UDX 3UDY 3UDZ 3UE0 3UE1 3UE2 3UE3 3UE4 3UE5 3UE6 3UE7 3UE8 3UE9 3UEB 3UEC 3UED 3UEE 3UEF 3UEG 3UEH 3UEI 3UEJ 3UEK 3UEL 3UEM 3UEN 3UEO 3UEP 3UEQ 3UER 3UES 3UET 3UEU 3UEV 3UEW 3UEX 3UEY 3UEZ 3UF0 3UF1 3UF2 3UF3 3UF4 3UF5 3UF6 3UF7 3UF8 3UF9 3UFA 3UFB 3UFC 3UFE 3UFF 3UFG 3UFH 3UFI 3UFK 3UFL 3UFM 3UFN 3UFO 3UFP 3UFQ 3UFR 3UFS 3UFT 3UFU 3UFV 3UFW 3UFX 3UFY 3UFZ 3UG0 3UG1 3UG2 3UG3 3UG4 3UG5 3UG6 3UG7 3UG8 3UG9 3UGB 3UGC 3UGD 3UGE 3UGF 3UGG 3UGH 3UGI 3UGJ 3UGK 3UGL 3UGQ 3UGR 3UGS 3UGT 3UGU 3UGV 3UGW 3UGX 3UGY 3UGZ 3UH0 3UH1 3UH2 3UH3 3UH4 3UH5 3UH6 3UH7 3UH8 3UHA 3UHB 3UHC 3UHD 3UHE 3UHF 3UHG 3UHH 3UHI 3UHJ 3UHK 3UHL 3UHM 3UHN 3UHO 3UHP 3UHQ 3UHR 3UHS 3UHT 3UHU 3UHV 3UHW 3UHX 3UHY 3UHZ 3UI0 3UI2 3UI3 3UI4 3UI5 3UI6 3UI7 3UIA 3UIB 3UIC 3UID 3UIE 3UIF 3UIG 3UIH 3UII 3UIJ 3UIK 3UIL 3UIM 3UIN 3UIO 3UIP 3UIR 3UIT 3UIU 3UIV 3UIW 3UIX 3UIY 3UIZ 3UJ0 3UJ1 3UJ2 3UJ3 3UJ4 3UJ6 3UJ7 3UJ8 3UJ9 3UJA 3UJB 3UJC 3UJD 3UJE 3UJF 3UJG 3UJH 3UJI 3UJJ 3UJK 3UJL 3UJM 3UJN 3UJO 3UJP 3UJQ 3UJR 3UJS 3UJT 3UJZ 3UK0 3UK1 3UK2 3UK4 3UK6 3UK7 3UK8 3UK9 3UKA 3UKD 3UKF 3UKH 3UKI 3UKJ 3UKK 3UKL 3UKM 3UKN 3UKO 3UKP 3UKQ 3UKR 3UKT 3UKU 3UKV 3UKW 3UKX 3UKY 3UKZ 3UL0 3UL1 3UL2 3UL3 3UL4 3UL5 3UL6 3UL7 3UL8 3UL9 3ULA 3ULB 3ULC 3ULE 3ULF 3ULG 3ULH 3ULI 3ULJ 3ULK 3ULL 3ULQ 3ULR 3ULS 3ULT 3ULU 3ULV 3ULX 3ULY 3ULZ 3UM0 3UM1 3UM2 3UM3 3UM5 3UM6 3UM7 3UM8 3UM9 3UMA 3UMB 3UMC 3UMD 3UME 3UMF 3UMG 3UMH 3UMI 3UMJ 3UMK 3UML 3UMM 3UMN 3UMO 3UMP 3UMQ 3UMR 3UMS 3UMT 3UMV 3UMW 3UMX 3UMZ 3UN0 3UN1 3UN2 3UN3 3UN4 3UN5 3UN6 3UN7 3UN8 3UN9 3UNA 3UNB 3UNC 3UND 3UNE 3UNF 3UNG 3UNH 3UNI 3UNJ 3UNK 3UNL 3UNM 3UNN 3UNO 3UNP 3UNQ 3UNR 3UNS 3UNT 3UNV 3UNW 3UNX 3UNY 3UNZ 3UO0 3UO1 3UO2 3UO3 3UO4 3UO5 3UO6 3UO8 3UO9 3UOA 3UOD 3UOE 3UOF 3UOG 3UOH 3UOI 3UOJ 3UOK 3UOL 3UOM 3UON 3UOP 3UOR 3UOT 3UOU 3UOV 3UOW 3UOX 3UOY 3UOZ 3UP0 3UP1 3UP2 3UP3 3UP4 3UP5 3UP6 3UP7 3UP8 3UP9 3UPA 3UPB 3UPC 3UPD 3UPE 3UPF 3UPG 3UPH 3UPI 3UPJ 3UPK 3UPL 3UPM 3UPN 3UPO 3UPP 3UPR 3UPS 3UPT 3UPV 3UPW 3UPX 3UPY 3UPZ 3UQ3 3UQ4 3UQ5 3UQ6 3UQ7 3UQ8 3UQ9 3UQA 3UQB 3UQC 3UQD 3UQE 3UQF 3UQG 3UQH 3UQI 3UQN 3UQO 3UQP 3UQR 3UQS 3UQU 3UQV 3UQW 3UQX 3UQY 3UQZ 3UR0 3UR1 3UR2 3UR3 3UR4 3UR5 3UR6 3UR7 3UR8 3UR9 3URA 3URB 3URC 3URD 3URE 3URF 3URG 3URH 3URI 3URJ 3URK 3URL 3URM 3URN 3URO 3URP 3URQ 3URR 3URY 3URZ 3US3 3US4 3US5 3US6 3US8 3US9 3USB 3USC 3USD 3USE 3USF 3USG 3USH 3USI 3USJ 3USK 3USL 3USM 3USO 3USP 3USQ 3USR 3USS 3UST 3USU 3USV 3USW 3USX 3USY 3USZ 3UT0 3UT1 3UT2 3UT3 3UT4 3UT5 3UT6 3UT7 3UT8 3UTC 3UTD 3UTE 3UTF 3UTG 3UTH 3UTK 3UTL 3UTM 3UTN 3UTO 3UTP 3UTQ 3UTS 3UTT 3UTU 3UTV 3UTW 3UTX 3UTY 3UTZ 3UU0 3UU1 3UU2 3UU3 3UU4 3UU5 3UU6 3UU7 3UU8 3UU9 3UUA 3UUB 3UUC 3UUD 3UUE 3UUF 3UUG 3UUL 3UUM 3UUN 3UUO 3UUS 3UUW 3UUX 3UUZ 3UV0 3UV1 3UV2 3UV3 3UV4 3UV5 3UV6 3UV7 3UV9 3UVA 3UVC 3UVD 3UVE 3UVH 3UVI 3UVJ 3UVK 3UVL 3UVM 3UVN 3UVO 3UVP 3UVQ 3UVR 3UVT 3UVU 3UVV 3UVW 3UVX 3UVY 3UW0 3UW1 3UW2 3UW3 3UW4 3UW5 3UW6 3UW8 3UW9 3UWA 3UWB 3UWC 3UWD 3UWE 3UWI 3UWJ 3UWK 3UWL 3UWM 3UWN 3UWO 3UWP 3UWQ 3UWS 3UWT 3UWU 3UWV 3UWW 3UWX 3UWY 3UWZ 3UX0 3UX1 3UX2 3UX3 3UX4 3UX7 3UX8 3UX9 3UXA 3UXD 3UXE 3UXF 3UXG 3UXH 3UXI 3UXJ 3UXK 3UXL 3UXM 3UXN 3UXO 3UXU 3UXV 3UXY 3UY4 3UY5 3UY6 3UY7 3UY8 3UY9 3UYC 3UYI 3UYJ 3UYK 3UYL 3UYN 3UYO 3UYP 3UYQ 3UYR 3UYS 3UYT 3UYU 3UYV 3UYW 3UYX 3UYY 3UZ0 3UZ5 3UZA 3UZB 3UZC 3UZD 3UZE 3UZJ 3UZO 3UZP 3UZQ 3UZR 3UZU 3UZV 3UZW 3UZX 3UZY 3UZZ 3V00 3V01 3V03 3V04 3V05 3V08 3V09 3V0A 3V0B 3V0C 3V0D 3V0E 3V0F 3V0G 3V0H 3V0I 3V0J 3V0L 3V0M 3V0N 3V0O 3V0P 3V0Q 3V0R 3V0S 3V0T 3V0U 3V0V 3V0W 3V0X 3V10 3V12 3V13 3V14 3V15 3V16 3V17 3V18 3V19 3V1A 3V1B 3V1C 3V1D 3V1E 3V1F 3V1G 3V1H 3V1K 3V1L 3V1M 3V1N 3V1O 3V1P 3V1Q 3V1R 3V1S 3V1T 3V1U 3V1V 3V1W 3V1X 3V1Y 3V2A 3V2B 3V2G 3V2H 3V2I 3V2J 3V2K 3V2L 3V2M 3V2N 3V2O 3V2P 3V2Q 3V2R 3V2T 3V2U 3V2V 3V2W 3V2X 3V2Y 3V2Z 3V30 3V31 3V32 3V33 3V34 3V35 3V36 3V38 3V39 3V3B 3V3C 3V3D 3V3E 3V3F 3V3G 3V3H 3V3I 3V3J 3V3K 3V3L 3V3M 3V3N 3V3O 3V3Q 3V3R 3V3S 3V3T 3V3V 3V3W 3V3X 3V3Y 3V3Z 3V42 3V43 3V44 3V45 3V46 3V47 3V48 3V49 3V4A 3V4B 3V4C 3V4D 3V4E 3V4F 3V4G 3V4H 3V4J 3V4K 3V4L 3V4M 3V4N 3V4O 3V4P 3V4Q 3V4S 3V4T 3V4U 3V4V 3V4W 3V4X 3V4Y 3V4Z 3V50 3V51 3V52 3V53 3V55 3V56 3V57 3V58 3V5A 3V5B 3V5C 3V5D 3V5E 3V5F 3V5G 3V5H 3V5I 3V5J 3V5K 3V5L 3V5M 3V5N 3V5O 3V5P 3V5Q 3V5R 3V5S 3V5T 3V5U 3V5V 3V5W 3V5X 3V5Y 3V5Z 3V60 3V61 3V62 3V64 3V65 3V66 3V67 3V68 3V69 3V6A 3V6B 3V6C 3V6E 3V6F 3V6G 3V6I 3V6L 3V6M 3V6N 3V6O 3V6P 3V6Q 3V6R 3V6S 3V6Z 3V70 3V75 3V76 3V77 3V78 3V7A 3V7B 3V7C 3V7D 3V7F 3V7I 3V7L 3V7M 3V7N 3V7O 3V7P 3V7Q 3V7R 3V7S 3V7T 3V7U 3V7V 3V7W 3V7X 3V7Y 3V7Z 3V80 3V82 3V83 3V84 3V85 3V86 3V87 3V88 3V89 3V8A 3V8B 3V8C 3V8D 3V8E 3V8F 3V8G 3V8H 3V8I 3V8J 3V8K 3V8L 3V8M 3V8N 3V8O 3V8P 3V8Q 3V8R 3V8S 3V8T 3V8U 3V8V 3V8W 3V8X 3V8Y 3V8Z 3V90 3V91 3V92 3V93 3V94 3V95 3V96 3V97 3V98 3V99 3V9A 3V9B 3V9C 3V9E 3V9F 3V9G 3V9H 3V9I 3V9J 3V9K 3V9L 3V9M 3V9O 3V9P 3V9R 3V9T 3V9V 3V9Y 3VA1 3VA2 3VA4 3VA5 3VA6 3VA7 3VA8 3VA9 3VAA 3VAB 3VAC 3VAD 3VAE 3VAP 3VAQ 3VAR 3VAS 3VAT 3VAU 3VAV 3VAW 3VAX 3VAY 3VAZ 3VB0 3VB1 3VB2 3VB3 3VB4 3VB5 3VB6 3VB7 3VB8 3VB9 3VBA 3VBB 3VBC 3VBD 3VBE 3VBF 3VBG 3VBH 3VBI 3VBJ 3VBK 3VBL 3VBM 3VBN 3VBO 3VBP 3VBQ 3VBR 3VBS 3VBT 3VBU 3VBV 3VBW 3VBX 3VBY 3VBZ 3VC0 3VC1 3VC2 3VC3 3VC4 3VC5 3VC6 3VC7 3VC8 3VCA 3VCB 3VCC 3VCD 3VCE 3VCF 3VCG 3VCH 3VCI 3VCJ 3VCK 3VCL 3VCM 3VCN 3VCO 3VCP 3VCR 3VCX 3VCY 3VCZ 3VD3 3VD4 3VD5 3VD7 3VD8 3VD9 3VDA 3VDB 3VDC 3VDD 3VDF 3VDG 3VDH 3VDI 3VDJ 3VDK 3VDL 3VDM 3VDN 3VDP 3VDQ 3VDR 3VDU 3VDX 3VDZ 3VE0 3VE1 3VE2 3VE3 3VE4 3VE5 3VE6 3VE7 3VE9 3VEC 3VED 3VEE 3VEF 3VEG 3VEH 3VEJ 3VEM 3VEN 3VEO 3VEP 3VEQ 3VER 3VES 3VET 3VEU 3VEV 3VEW 3VEX 3VEY 3VEZ 3VF0 3VF1 3VF2 3VF3 3VF4 3VF5 3VF6 3VF7 3VF8 3VF9 3VFA 3VFB 3VFC 3VFD 3VFE 3VFF 3VFG 3VFH 3VFI 3VFJ 3VFK 3VFL 3VFM 3VFN 3VFO 3VFP 3VFQ 3VFR 3VFS 3VFT 3VFU 3VFV 3VFW 3VFZ 3VG0 3VG1 3VG2 3VG3 3VG4 3VG5 3VG6 3VG7 3VG8 3VG9 3VGA 3VGB 3VGC 3VGD 3VGE 3VGF 3VGG 3VGH 3VGI 3VGJ 3VGK 3VGL 3VGM 3VGN 3VGO 3VGP 3VGS 3VGT 3VGU 3VGV 3VGW 3VGX 3VGY 3VGZ 3VH1 3VH2 3VH3 3VH4 3VH5 3VH6 3VH7 3VH8 3VH9 3VHA 3VHB 3VHC 3VHD 3VHE 3VHF 3VHG 3VHH 3VHI 3VHJ 3VHK 3VHL 3VHM 3VHN 3VHO 3VHP 3VHQ 3VHR 3VHS 3VHT 3VHU 3VHV 3VHW 3VHX 3VHZ 3VI0 3VI1 3VI2 3VI3 3VI4 3VI5 3VI6 3VI7 3VI8 3VIA 3VIB 3VIC 3VID 3VIE 3VIF 3VIG 3VIH 3VII 3VIJ 3VIK 3VIL 3VIM 3VIN 3VIO 3VIP 3VIQ 3VIR 3VIS 3VIU 3VIV 3VJ6 3VJ7 3VJ8 3VJ9 3VJA 3VJB 3VJC 3VJD 3VJE 3VJF 3VJH 3VJI 3VJJ 3VJK 3VJL 3VJM 3VJN 3VJO 3VJP 3VJQ 3VJS 3VJT 3VJZ 3VK0 3VK1 3VK2 3VK3 3VK4 3VK5 3VK6 3VK9 3VKA 3VKB 3VKC 3VKD 3VKF 3VKG 3VKH 3VKI 3VKJ 3VKK 3VKL 3VKM 3VKN 3VKO 3VKP 3VKQ 3VKR 3VKS 3VKT 3VKU 3VKV 3VKW 3VKX 3VKZ 3VL1 3VL2 3VL3 3VL4 3VL6 3VL7 3VL8 3VL9 3VLA 3VLB 3VLC 3VLD 3VLE 3VLF 3VLG 3VLH 3VLI 3VLJ 3VLK 3VLL 3VLM 3VLN 3VLU 3VLV 3VLW 3VLX 3VLY 3VLZ 3VM0 3VM1 3VM4 3VM5 3VM6 3VM7 3VM8 3VM9 3VMA 3VMF 3VMG 3VMH 3VMI 3VMJ 3VMK 3VML 3VMM 3VMN 3VMO 3VMP 3VMQ 3VMR 3VMS 3VMT 3VMV 3VMW 3VMX 3VMY 3VMZ 3VN0 3VN2 3VN3 3VN4 3VN5 3VN9 3VNA 3VNB 3VNC 3VND 3VNE 3VNF 3VNG 3VNH 3VNI 3VNJ 3VNK 3VNL 3VNM 3VNN 3VNO 3VNP 3VNQ 3VNR 3VNS 3VNT 3VNW 3VNX 3VNY 3VNZ 3VO0 3VO1 3VO2 3VO3 3VO8 3VO9 3VOA 3VOB 3VOC 3VOD 3VOE 3VOF 3VOG 3VOH 3VOI 3VOJ 3VOL 3VOM 3VON 3VOO 3VOP 3VOQ 3VOR 3VOS 3VOT 3VOU 3VOV 3VOW 3VOX 3VOY 3VOZ 3VP0 3VP1 3VP2 3VP3 3VP4 3VP5 3VP6 3VP7 3VP8 3VP9 3VPA 3VPB 3VPC 3VPD 3VPE 3VPF 3VPG 3VPH 3VPI 3VPJ 3VPK 3VPL 3VPM 3VPN 3VPO 3VPP 3VPQ 3VPR 3VPS 3VPT 3VPV 3VPX 3VPY 3VPZ 3VQ1 3VQ2 3VQ4 3VQ5 3VQ6 3VQ7 3VQ8 3VQ9 3VQA 3VQB 3VQC 3VQD 3VQE 3VQF 3VQG 3VQH 3VQI 3VQJ 3VQK 3VQL 3VQM 3VQP 3VQQ 3VQR 3VQS 3VQT 3VQU 3VQV 3VQW 3VQX 3VQY 3VQZ 3VR0 3VR1 3VR2 3VR3 3VR4 3VR5 3VR6 3VR8 3VR9 3VRA 3VRB 3VRC 3VRD 3VRE 3VRF 3VRG 3VRH 3VRI 3VRJ 3VRK 3VRL 3VRM 3VRN 3VRO 3VRP 3VRQ 3VRR 3VRT 3VRU 3VRV 3VRW 3VRY 3VRZ 3VS0 3VS1 3VS2 3VS3 3VS4 3VS5 3VS6 3VS7 3VS8 3VS9 3VSA 3VSB 3VSC 3VSD 3VSE 3VSF 3VSG 3VSH 3VSI 3VSJ 3VSK 3VSL 3VSM 3VSN 3VSO 3VSP 3VSQ 3VSR 3VSS 3VST 3VSU 3VSV 3VSW 3VSX 3VSY 3VSZ 3VT0 3VT1 3VT2 3VT3 3VT4 3VT5 3VT6 3VT7 3VT8 3VT9 3VTA 3VTB 3VTC 3VTD 3VTE 3VTF 3VTG 3VTH 3VTI 3VTJ 3VTK 3VTM 3VTN 3VTO 3VTP 3VTQ 3VTR 3VTS 3VTT 3VTU 3VTV 3VTW 3VTX 3VTY 3VTZ 3VU0 3VU1 3VU2 3VU3 3VU4 3VU5 3VU6 3VU7 3VU8 3VU9 3VUA 3VUB 3VUC 3VUD 3VUE 3VUF 3VUG 3VUH 3VUI 3VUK 3VUL 3VUM 3VUN 3VUO 3VUP 3VUQ 3VUR 3VUS 3VUT 3VUU 3VUV 3VUW 3VUX 3VUY 3VUZ 3VV0 3VV1 3VV2 3VV3 3VV4 3VV5 3VV6 3VV7 3VV8 3VV9 3VVA 3VVB 3VVC 3VVD 3VVE 3VVF 3VVG 3VVH 3VVI 3VVJ 3VVK 3VVL 3VVM 3VVN 3VVO 3VVP 3VVR 3VVS 3VVT 3VVU 3VVV 3VVW 3VVX 3VVY 3VVZ 3VW0 3VW1 3VW2 3VW5 3VW6 3VW7 3VW8 3VW9 3VWA 3VWC 3VWD 3VWE 3VWI 3VWJ 3VWK 3VWL 3VWM 3VWN 3VWO 3VWP 3VWQ 3VWR 3VWS 3VWU 3VWV 3VWW 3VWX 3VX0 3VX1 3VX3 3VX4 3VX6 3VX7 3VX8 3VXB 3VXC 3VXD 3VXE 3VXG 3VXI 3VXJ 3VXK 3VXM 3VXN 3VXO 3VXP 3VXQ 3VXR 3VXS 3VXT 3VXU 3VXW 3VY2 3VY5 3VY6 3VY7 3VY8 3VY9 3VYA 3VYC 3VYD 3VYE 3VYF 3VYG 3VYH 3VYI 3VYJ 3VYK 3VYL 3VYM 3VYN 3VYO 3VYP 3VYR 3VYS 3VYT 3VYU 3VYV 3VYW 3VZ0 3VZ1 3VZ2 3VZ3 3VZ6 3VZ7 3VZ8 3VZ9 3VZA 3VZB 3VZC 3VZD 3VZE 3VZF 3VZG 3VZH 3VZI 3VZJ 3VZK 3VZL 3VZM 3VZN 3VZO 3VZP 3VZQ 3VZR 3VZS 3VZT 3VZU 3VZV 3VZW 3VZX 3VZY 3VZZ 3W00 3W01 3W02 3W03 3W04 3W05 3W06 3W07 3W08 3W09 3W0A 3W0C 3W0D 3W0E 3W0F 3W0G 3W0H 3W0I 3W0J 3W0K 3W0L 3W0M 3W0N 3W0O 3W0P 3W0Q 3W0R 3W0S 3W0T 3W0U 3W0W 3W0Y 3W10 3W11 3W12 3W13 3W14 3W15 3W16 3W18 3W19 3W1A 3W1B 3W1C 3W1D 3W1E 3W1F 3W1G 3W1H 3W1I 3W1J 3W1L 3W1M 3W1N 3W1O 3W1P 3W1Q 3W1R 3W1S 3W1T 3W1U 3W1V 3W1W 3W1X 3W1Y 3W1Z 3W20 3W21 3W22 3W23 3W24 3W25 3W26 3W27 3W28 3W29 3W2C 3W2D 3W2E 3W2F 3W2G 3W2H 3W2I 3W2J 3W2K 3W2L 3W2M 3W2N 3W2O 3W2P 3W2Q 3W2R 3W2S 3W2T 3W2U 3W2V 3W2W 3W2X 3W2Y 3W2Z 3W30 3W31 3W32 3W33 3W34 3W35 3W36 3W37 3W38 3W39 3W3A 3W3B 3W3D 3W3E 3W3G 3W3J 3W3K 3W3L 3W3M 3W3N 3W3O 3W3T 3W3U 3W3V 3W3W 3W3X 3W3Y 3W3Z 3W40 3W41 3W42 3W43 3W44 3W45 3W4I 3W4J 3W4K 3W4O 3W4P 3W4Q 3W4R 3W4S 3W4T 3W4U 3W4Y 3W51 3W52 3W53 3W54 3W55 3W56 3W57 3W58 3W59 3W5A 3W5B 3W5C 3W5D 3W5E 3W5F 3W5G 3W5H 3W5I 3W5J 3W5K 3W5M 3W5N 3W5O 3W5P 3W5Q 3W5R 3W5S 3W5T 3W5U 3W5V 3W5W 3W5X 3W5Y 3W5Z 3W60 3W61 3W62 3W63 3W64 3W65 3W66 3W67 3W68 3W69 3W6A 3W6B 3W6C 3W6D 3W6E 3W6F 3W6G 3W6H 3W6I 3W6J 3W6K 3W6L 3W6M 3W6N 3W6O 3W6P 3W6Q 3W6R 3W6S 3W6U 3W6W 3W6X 3W6Y 3W6Z 3W70 3W71 3W72 3W73 3W74 3W75 3W76 3W77 3W78 3W79 3W7A 3W7B 3W7C 3W7D 3W7E 3W7F 3W7G 3W7H 3W7I 3W7J 3W7K 3W7L 3W7M 3W7N 3W7O 3W7P 3W7Q 3W7R 3W7S 3W7T 3W7U 3W7V 3W7W 3W7X 3W7Y 3W7Z 3W80 3W81 3W82 3W83 3W84 3W85 3W86 3W87 3W88 3W8D 3W8E 3W8F 3W8G 3W8H 3W8I 3W8J 3W8K 3W8L 3W8M 3W8N 3W8O 3W8P 3W8Q 3W8R 3W8S 3W8V 3W8W 3W8X 3W8Z 3W90 3W91 3W92 3W93 3W94 3W95 3W9A 3W9B 3W9C 3W9D 3W9E 3W9F 3W9G 3W9H 3W9I 3W9J 3W9K 3W9P 3W9R 3W9S 3W9T 3W9U 3W9V 3W9W 3W9Y 3W9Z 3WA0 3WA1 3WA2 3WA3 3WA4 3WA5 3WA6 3WA7 3WA8 3WAB 3WAD 3WAE 3WAF 3WAG 3WAH 3WAI 3WAJ 3WAK 3WAL 3WAM 3WAN 3WAO 3WAP 3WAQ 3WAR 3WAS 3WAT 3WAU 3WAV 3WAW 3WAX 3WAY 3WB0 3WB1 3WB2 3WB4 3WB5 3WB8 3WB9 3WBA 3WBB 3WBD 3WBE 3WBF 3WBG 3WBH 3WBI 3WBJ 3WBK 3WBL 3WBN 3WBP 3WBQ 3WBR 3WBW 3WBX 3WBY 3WBZ 3WC0 3WC3 3WC4 3WC5 3WC6 3WC7 3WC8 3WC9 3WCA 3WCB 3WCC 3WCD 3WCE 3WCF 3WCG 3WCH 3WCI 3WCJ 3WCK 3WCL 3WCM 3WCN 3WCO 3WCP 3WCQ 3WCR 3WCS 3WCT 3WCU 3WCV 3WCW 3WCX 3WCY 3WCZ 3WD0 3WD1 3WD2 3WD3 3WD4 3WD5 3WD6 3WD7 3WD8 3WD9 3WDB 3WDC 3WDD 3WDE 3WDF 3WDG 3WDH 3WDI 3WDJ 3WDK 3WDL 3WDM 3WDN 3WDO 3WDP 3WDQ 3WDR 3WDS 3WDT 3WDU 3WDV 3WDW 3WDX 3WDY 3WDZ 3WE0 3WE1 3WE2 3WE3 3WE4 3WE5 3WE6 3WE7 3WE8 3WE9 3WEA 3WEB 3WEC 3WEE 3WEF 3WEG 3WEH 3WEI 3WEJ 3WEK 3WEL 3WEM 3WEN 3WEO 3WEU 3WEV 3WEW 3WEX 3WEZ 3WF0 3WF1 3WF2 3WF3 3WF4 3WF5 3WF6 3WF7 3WF8 3WF9 3WFA 3WFB 3WFC 3WFD 3WFE 3WFF 3WFG 3WFH 3WFI 3WFJ 3WFL 3WFN 3WFO 3WFP 3WFT 3WFU 3WFV 3WFW 3WFX 3WFZ 3WG1 3WG2 3WG3 3WG4 3WG5 3WG6 3WG7 3WG8 3WG9 3WGB 3WGC 3WGD 3WGE 3WGG 3WGH 3WGJ 3WGK 3WGL 3WGM 3WGN 3WGO 3WGP 3WGQ 3WGT 3WGU 3WGV 3WGW 3WGX 3WGY 3WGZ 3WH0 3WH1 3WH2 3WH3 3WH4 3WH5 3WH6 3WH7 3WH8 3WH9 3WHA 3WHB 3WHC 3WHD 3WHE 3WHI 3WHJ 3WHK 3WHL 3WHM 3WHN 3WHO 3WHP 3WHQ 3WHR 3WHS 3WHT 3WHU 3WHW 3WHX 3WI0 3WI1 3WI2 3WI3 3WI4 3WI5 3WI6 3WI7 3WI8 3WI9 3WIA 3WIB 3WIC 3WID 3WIE 3WIF 3WIG 3WIH 3WII 3WIJ 3WIK 3WIL 3WIM 3WIN 3WIO 3WIP 3WIQ 3WIR 3WIS 3WIT 3WIU 3WIV 3WIW 3WIX 3WIY 3WIZ 3WJ1 3WJ2 3WJ4 3WJ5 3WJ6 3WJ7 3WJ8 3WJ9 3WJA 3WJB 3WJC 3WJD 3WJE 3WJF 3WJG 3WJJ 3WJK 3WJL 3WJM 3WJN 3WJO 3WJP 3WJQ 3WJR 3WJS 3WJT 3WJU 3WJV 3WJW 3WJX 3WJY 3WJZ 3WK0 3WK1 3WK2 3WK3 3WK4 3WK5 3WK6 3WK7 3WK8 3WK9 3WKA 3WKB 3WKC 3WKD 3WKE 3WKF 3WKG 3WKH 3WKI 3WKL 3WKM 3WKN 3WKP 3WKQ 3WKR 3WKS 3WKT 3WKU 3WKV 3WKW 3WKX 3WKY 3WKZ 3WL0 3WL1 3WL2 3WL3 3WL4 3WL5 3WL6 3WL7 3WL8 3WL9 3WLA 3WLB 3WLC 3WLD 3WLE 3WLF 3WLH 3WLI 3WLJ 3WLK 3WLL 3WLM 3WLN 3WLO 3WLP 3WLQ 3WLR 3WLS 3WLT 3WLU 3WLV 3WLW 3WLX 3WMB 3WMC 3WMD 3WME 3WMF 3WMG 3WMH 3WMI 3WMJ 3WMK 3WML 3WMM 3WMP 3WMQ 3WMR 3WMS 3WMT 3WMU 3WMV 3WMW 3WMX 3WMY 3WMZ 3WN0 3WN1 3WN2 3WN4 3WN5 3WN6 3WN7 3WN8 3WNB 3WNC 3WND 3WNE 3WNF 3WNG 3WNH 3WNI 3WNJ 3WNK 3WNL 3WNM 3WNN 3WNO 3WNP 3WNQ 3WNR 3WNS 3WNT 3WNU 3WNV 3WNW 3WNX 3WNY 3WNZ 3WO0 3WO1 3WO2 3WO3 3WO4 3WO5 3WO6 3WO7 3WO8 3WO9 3WOA 3WOB 3WOC 3WOD 3WOE 3WOF 3WOG 3WOH 3WOI 3WOJ 3WOK 3WOL 3WOM 3WON 3WOO 3WOP 3WOQ 3WOR 3WOU 3WOV 3WOW 3WOY 3WOZ 3WP0 3WP1 3WP3 3WP4 3WP5 3WP6 3WP8 3WP9 3WPA 3WPB 3WPF 3WPJ 3WPK 3WPL 3WPM 3WPN 3WPO 3WPP 3WPQ 3WPR 3WPS 3WPT 3WPU 3WPV 3WPW 3WPX 3WPY 3WPZ 3WQ0 3WQ1 3WQ4 3WQ5 3WQ6 3WQ7 3WQ8 3WQ9 3WQA 3WQB 3WQC 3WQD 3WQE 3WQF 3WQG 3WQH 3WQJ 3WQK 3WQL 3WQM 3WQN 3WQO 3WQP 3WQQ 3WQR 3WQS 3WQT 3WQU 3WQV 3WQW 3WR0 3WR1 3WR2 3WR3 3WR4 3WR5 3WR7 3WR8 3WR9 3WRA 3WRB 3WRC 3WRD 3WRE 3WRF 3WRG 3WRH 3WRI 3WRJ 3WRK 3WRL 3WRM 3WRN 3WRO 3WRP 3WRQ 3WRR 3WRS 3WRT 3WRV 3WRW 3WRX 3WRY 3WRZ 3WS0 3WS1 3WS2 3WS3 3WS4 3WS5 3WS6 3WS7 3WS8 3WS9 3WSA 3WSB 3WSC 3WSD 3WSE 3WSF 3WSG 3WSH 3WSI 3WSJ 3WSO 3WSP 3WSQ 3WSR 3WST 3WSU 3WSV 3WSW 3WSX 3WSY 3WSZ 3WT0 3WT1 3WT2 3WT3 3WT4 3WT5 3WT6 3WT7 3WTB 3WTC 3WTD 3WTF 3WTG 3WTH 3WTI 3WTJ 3WTK 3WTL 3WTM 3WTN 3WTO 3WTQ 3WTR 3WTZ 3WU0 3WU2 3WU3 3WU4 3WU5 3WU6 3WU7 3WU8 3WU9 3WUA 3WUB 3WUC 3WUD 3WUE 3WUF 3WUG 3WUH 3WUI 3WUL 3WUM 3WUN 3WUP 3WUQ 3WUR 3WUS 3WUT 3WUU 3WUV 3WUW 3WUX 3WUY 3WUZ 3WV0 3WV1 3WV2 3WV3 3WV4 3WV5 3WV6 3WV7 3WV8 3WV9 3WVA 3WVB 3WVC 3WVD 3WVE 3WVF 3WVJ 3WVL 3WVM 3WVN 3WVO 3WVQ 3WVR 3WVS 3WVT 3WVU 3WVV 3WVW 3WVX 3WVY 3WVZ 3WW0 3WW1 3WW2 3WW3 3WW4 3WW5 3WW6 3WW7 3WW8 3WW9 3WWA 3WWB 3WWC 3WWD 3WWE 3WWF 3WWG 3WWH 3WWI 3WWJ 3WWK 3WWL 3WWM 3WWN 3WWO 3WWP 3WWQ 3WWR 3WWS 3WWT 3WWV 3WWX 3WWY 3WWZ 3WX0 3WX1 3WX2 3WX4 3WX5 3WX6 3WX7 3WX8 3WX9 3WXA 3WXB 3WXC 3WXE 3WXF 3WXG 3WXI 3WXJ 3WXK 3WXL 3WXM 3WXO 3WXP 3WXQ 3WXR 3WXS 3WXT 3WXU 3WXV 3WXW 3WXX 3WXY 3WXZ 3WY0 3WY1 3WY2 3WY3 3WY4 3WY6 3WY7 3WY8 3WY9 3WYA 3WYB 3WYC 3WYD 3WYE 3WYF 3WYG 3WYH 3WYI 3WYJ 3WYK 3WYL 3WYM 3WYN 3WYO 3WYP 3WYQ 3WYR 3WYW 3WYX 3WYY 3WYZ 3WZ0 3WZ1 3WZ2 3WZ3 3WZ4 3WZ5 3WZ6 3WZ7 3WZ8 3WZD 3WZE 3WZF 3WZG 3WZH 3WZJ 3WZK 3WZL 3WZM 3WZN 3WZO 3WZP 3WZQ 3WZS 3WZT 3WZU 3WZV 3WZW 3WZX 3WZY 3WZZ 3X00 3X01 3X02 3X03 3X04 3X05 3X06 3X07 3X08 3X09 3X0A 3X0B 3X0C 3X0D 3X0E 3X0F 3X0G 3X0H 3X0T 3X0U 3X0V 3X0W 3X0X 3X0Y 3X11 3X12 3X13 3X14 3X15 3X16 3X17 3X1B 3X1D 3X1E 3X1F 3X1G 3X1H 3X1I 3X1J 3X1K 3X1M 3X1N 3X1O 3X1W 3X1X 3X1Y 3X1Z 3X20 3X21 3X22 3X23 3X24 3X25 3X26 3X27 3X28 3X29 3X2D 3X2E 3X2F 3X2G 3X2H 3X2I 3X2J 3X2K 3X2L 3X2M 3X2N 3X2Q 3X2R 3X2S 3X2T 3X2U 3X2V 3X2W 3X31 3X32 3X33 3X34 3X35 3X36 3X37 3X38 3X39 3X3B 3X3C 3X3F 3X3G 3X3M 3X3N 3X3U 3X3X 3X3Y 3X3Z 3X40 3X41 3X42 3X43 3X44 3XIM 3XIN 3XIS 3YAS 3YGS 3YPI 3ZBD 3ZBF 3ZBG 3ZBH 3ZBK 3ZBL 3ZBM 3ZBN 3ZBO 3ZBP 3ZBQ 3ZBR 3ZBS 3ZBT 3ZBU 3ZBV 3ZBW 3ZBX 3ZBY 3ZBZ 3ZC1 3ZC3 3ZC4 3ZC5 3ZC6 3ZC7 3ZC8 3ZC9 3ZCB 3ZCC 3ZCD 3ZCF 3ZCG 3ZCH 3ZCI 3ZCJ 3ZCL 3ZCM 3ZCN 3ZCO 3ZCP 3ZCQ 3ZCR 3ZCS 3ZCT 3ZCU 3ZCV 3ZCW 3ZCX 3ZCY 3ZCZ 3ZD1 3ZD2 3ZD8 3ZD9 3ZDE 3ZDF 3ZDG 3ZDH 3ZDI 3ZDJ 3ZDL 3ZDM 3ZDN 3ZDO 3ZDP 3ZDQ 3ZDR 3ZDS 3ZDT 3ZDU 3ZDV 3ZDW 3ZDX 3ZDY 3ZDZ 3ZE0 3ZE1 3ZE2 3ZE3 3ZE4 3ZE5 3ZE6 3ZE7 3ZE8 3ZE9 3ZEA 3ZEB 3ZEC 3ZED 3ZEF 3ZEI 3ZEJ 3ZEK 3ZEP 3ZET 3ZEU 3ZEV 3ZEW 3ZEZ 3ZF0 3ZF1 3ZF2 3ZF3 3ZF4 3ZF5 3ZF6 3ZF8 3ZFB 3ZFC 3ZFD 3ZFE 3ZFF 3ZFG 3ZFH 3ZFI 3ZFK 3ZFL 3ZFM 3ZFN 3ZFO 3ZFP 3ZFQ 3ZFR 3ZFT 3ZFU 3ZFV 3ZFW 3ZFX 3ZFY 3ZFZ 3ZG0 3ZG1 3ZG2 3ZG3 3ZG5 3ZG6 3ZG7 3ZG8 3ZG9 3ZGA 3ZGB 3ZGC 3ZGD 3ZGE 3ZGF 3ZGG 3ZGH 3ZGI 3ZGJ 3ZGL 3ZGN 3ZGO 3ZGQ 3ZGV 3ZGX 3ZGY 3ZH0 3ZH3 3ZH4 3ZH5 3ZH6 3ZH7 3ZH8 3ZH9 3ZHA 3ZHB 3ZHC 3ZHD 3ZHE 3ZHF 3ZHG 3ZHH 3ZHI 3ZHK 3ZHL 3ZHN 3ZHO 3ZHP 3ZHQ 3ZHR 3ZHS 3ZHT 3ZHU 3ZHV 3ZHW 3ZHX 3ZHY 3ZHZ 3ZI0 3ZI1 3ZI3 3ZI4 3ZI6 3ZI7 3ZI8 3ZIA 3ZIB 3ZID 3ZIE 3ZIG 3ZIH 3ZII 3ZIJ 3ZIK 3ZIL 3ZIM 3ZIN 3ZIO 3ZIP 3ZIQ 3ZIR 3ZIT 3ZIU 3ZIV 3ZIW 3ZIX 3ZIY 3ZIZ 3ZJ0 3ZJ4 3ZJ5 3ZJ6 3ZJ7 3ZJ8 3ZJA 3ZJB 3ZJC 3ZJD 3ZJE 3ZJF 3ZJG 3ZJH 3ZJI 3ZJJ 3ZJK 3ZJL 3ZJM 3ZJN 3ZJO 3ZJP 3ZJQ 3ZJR 3ZJS 3ZJX 3ZJY 3ZJZ 3ZK0 3ZK1 3ZK2 3ZK4 3ZK5 3ZK6 3ZK7 3ZK8 3ZK9 3ZKA 3ZKB 3ZKD 3ZKE 3ZKF 3ZKG 3ZKI 3ZKJ 3ZKK 3ZKL 3ZKM 3ZKN 3ZKP 3ZKQ 3ZKR 3ZKS 3ZKU 3ZKV 3ZKW 3ZKX 3ZKY 3ZL1 3ZL2 3ZL4 3ZL5 3ZL6 3ZL7 3ZL8 3ZL9 3ZLB 3ZLC 3ZLD 3ZLE 3ZLF 3ZLG 3ZLH 3ZLI 3ZLK 3ZLL 3ZLM 3ZLN 3ZLO 3ZLP 3ZLQ 3ZLR 3ZLS 3ZLT 3ZLU 3ZLV 3ZLW 3ZLX 3ZLY 3ZLZ 3ZM0 3ZM1 3ZM2 3ZM3 3ZM4 3ZM5 3ZM6 3ZM7 3ZM8 3ZM9 3ZMB 3ZMC 3ZMD 3ZME 3ZMF 3ZMG 3ZMH 3ZMI 3ZMJ 3ZMK 3ZML 3ZMM 3ZMN 3ZMO 3ZMP 3ZMQ 3ZMR 3ZMS 3ZMT 3ZMU 3ZMV 3ZMZ 3ZN0 3ZN1 3ZN2 3ZN3 3ZN4 3ZN5 3ZN6 3ZNB 3ZNC 3ZNG 3ZNH 3ZNI 3ZNJ 3ZNK 3ZNL 3ZNM 3ZNN 3ZNO 3ZNP 3ZNQ 3ZNR 3ZNS 3ZNT 3ZNU 3ZNV 3ZNX 3ZNY 3ZNZ 3ZO0 3ZO1 3ZO2 3ZO3 3ZO4 3ZO5 3ZO6 3ZO7 3ZO8 3ZO9 3ZOA 3ZOC 3ZOD 3ZOE 3ZOF 3ZOG 3ZOH 3ZOI 3ZOJ 3ZOK 3ZOL 3ZOM 3ZON 3ZOO 3ZOP 3ZOQ 3ZOR 3ZOS 3ZOT 3ZOU 3ZOV 3ZOW 3ZOX 3ZOY 3ZOZ 3ZP0 3ZP1 3ZP2 3ZP3 3ZP4 3ZP6 3ZP7 3ZP9 3ZPA 3ZPB 3ZPC 3ZPE 3ZPF 3ZPG 3ZPH 3ZPI 3ZPJ 3ZPN 3ZPO 3ZPP 3ZPQ 3ZPR 3ZPS 3ZPT 3ZPU 3ZPV 3ZPX 3ZPY 3ZQ3 3ZQ4 3ZQ5 3ZQ6 3ZQ7 3ZQ8 3ZQ9 3ZQA 3ZQB 3ZQE 3ZQF 3ZQG 3ZQH 3ZQI 3ZQJ 3ZQK 3ZQM 3ZQN 3ZQO 3ZQP 3ZQQ 3ZQR 3ZQS 3ZQT 3ZQU 3ZQV 3ZQW 3ZQX 3ZQY 3ZQZ 3ZR0 3ZR1 3ZR4 3ZR5 3ZR6 3ZR7 3ZR8 3ZR9 3ZRA 3ZRB 3ZRC 3ZRD 3ZRE 3ZRF 3ZRG 3ZRH 3ZRI 3ZRJ 3ZRK 3ZRL 3ZRM 3ZRP 3ZRQ 3ZRR 3ZRS 3ZRT 3ZRV 3ZRW 3ZRX 3ZRY 3ZRZ 3ZS0 3ZS1 3ZS2 3ZS3 3ZS4 3ZS5 3ZS6 3ZS7 3ZS8 3ZS9 3ZSC 3ZSE 3ZSF 3ZSG 3ZSH 3ZSI 3ZSJ 3ZSK 3ZSL 3ZSM 3ZSN 3ZSO 3ZSQ 3ZSR 3ZSS 3ZST 3ZSU 3ZSV 3ZSW 3ZSX 3ZSY 3ZSZ 3ZT0 3ZT1 3ZT2 3ZT3 3ZT4 3ZT5 3ZT6 3ZT7 3ZT9 3ZTA 3ZTB 3ZTC 3ZTD 3ZTE 3ZTF 3ZTH 3ZTJ 3ZTL 3ZTM 3ZTN 3ZTO 3ZTP 3ZTQ 3ZTR 3ZTS 3ZTT 3ZTV 3ZTW 3ZTX 3ZTY 3ZTZ 3ZU0 3ZU1 3ZU2 3ZU3 3ZU4 3ZU5 3ZU6 3ZU7 3ZU8 3ZUC 3ZUD 3ZUF 3ZUG 3ZUI 3ZUJ 3ZUK 3ZUL 3ZUM 3ZUN 3ZUO 3ZUP 3ZUQ 3ZUR 3ZUS 3ZUT 3ZUU 3ZUV 3ZUW 3ZUX 3ZUY 3ZUZ 3ZV0 3ZV2 3ZV3 3ZV4 3ZV5 3ZV6 3ZV7 3ZV8 3ZV9 3ZVA 3ZVB 3ZVC 3ZVD 3ZVE 3ZVF 3ZVG 3ZVH 3ZVI 3ZVJ 3ZVL 3ZVQ 3ZVR 3ZVS 3ZVT 3ZVU 3ZVV 3ZVW 3ZVX 3ZVY 3ZVZ 3ZW0 3ZW1 3ZW2 3ZW3 3ZW5 3ZW7 3ZW8 3ZW9 3ZWA 3ZWB 3ZWC 3ZWD 3ZWE 3ZWF 3ZWG 3ZWH 3ZWI 3ZWJ 3ZWK 3ZWL 3ZWM 3ZWN 3ZWO 3ZWP 3ZWQ 3ZWS 3ZWT 3ZWU 3ZWV 3ZWW 3ZWX 3ZWY 3ZWZ 3ZX0 3ZX1 3ZX2 3ZX3 3ZX4 3ZX5 3ZX6 3ZX7 3ZXA 3ZXB 3ZXC 3ZXD 3ZXE 3ZXF 3ZXG 3ZXH 3ZXI 3ZXJ 3ZXK 3ZXL 3ZXN 3ZXO 3ZXP 3ZXQ 3ZXR 3ZXS 3ZXT 3ZXU 3ZXV 3ZXW 3ZXX 3ZXY 3ZXZ 3ZY0 3ZY1 3ZY2 3ZY3 3ZY4 3ZY5 3ZY6 3ZY7 3ZYA 3ZYB 3ZYC 3ZYD 3ZYE 3ZYF 3ZYG 3ZYH 3ZYI 3ZYJ 3ZYK 3ZYL 3ZYM 3ZYN 3ZYO 3ZYP 3ZYQ 3ZYR 3ZYT 3ZYU 3ZYV 3ZYW 3ZYX 3ZYY 3ZYZ 3ZZ0 3ZZ1 3ZZ3 3ZZ4 3ZZ5 3ZZ6 3ZZ7 3ZZ8 3ZZ9 3ZZA 3ZZB 3ZZC 3ZZD 3ZZE 3ZZF 3ZZG 3ZZH 3ZZI 3ZZJ 3ZZK 3ZZL 3ZZM 3ZZN 3ZZO 3ZZP 3ZZQ 3ZZR 3ZZS 3ZZT 3ZZU 3ZZV 3ZZW 3ZZX 3ZZY 3ZZZ 41BI 421P 43C9 43CA 451C 456C 4A00 4A01 4A02 4A03 4A05 4A06 4A07 4A0C 4A0D 4A0E 4A0F 4A0G 4A0H 4A0I 4A0J 4A0M 4A0N 4A0P 4A0Q 4A0R 4A0S 4A0T 4A0U 4A0X 4A0Y 4A0Z 4A10 4A11 4A14 4A16 4A1F 4A1G 4A1H 4A1I 4A1J 4A1K 4A1N 4A1O 4A1R 4A1S 4A1T 4A1U 4A1V 4A1W 4A1X 4A1Y 4A1Z 4A20 4A21 4A22 4A23 4A25 4A26 4A27 4A28 4A29 4A2A 4A2B 4A2C 4A2D 4A2E 4A2F 4A2G 4A2H 4A2J 4A2L 4A2M 4A2N 4A2O 4A2P 4A2Q 4A2R 4A2S 4A2U 4A2V 4A2W 4A2Y 4A2Z 4A30 4A31 4A32 4A33 4A34 4A35 4A37 4A38 4A39 4A3H 4A3N 4A3O 4A3P 4A3Q 4A3R 4A3S 4A3T 4A3U 4A3V 4A3W 4A3X 4A3Y 4A3Z 4A41 4A42 4A44 4A45 4A46 4A47 4A48 4A49 4A4A 4A4B 4A4C 4A4D 4A4I 4A4J 4A4K 4A4L 4A4M 4A4O 4A4P 4A4Q 4A4V 4A4W 4A4X 4A4Y 4A4Z 4A50 4A51 4A55 4A56 4A57 4A59 4A5A 4A5B 4A5G 4A5K 4A5L 4A5M 4A5N 4A5O 4A5P 4A5R 4A5S 4A5T 4A5U 4A5W 4A5X 4A5Y 4A5Z 4A60 4A61 4A62 4A63 4A64 4A65 4A66 4A67 4A68 4A69 4A6A 4A6B 4A6C 4A6D 4A6E 4A6F 4A6G 4A6H 4A6K 4A6L 4A6N 4A6O 4A6P 4A6Q 4A6R 4A6S 4A6T 4A6U 4A6V 4A6W 4A6X 4A6Y 4A6Z 4A71 4A72 4A73 4A78 4A79 4A7A 4A7B 4A7C 4A7D 4A7E 4A7G 4A7I 4A7J 4A7K 4A7M 4A7P 4A7Q 4A7S 4A7T 4A7U 4A7V 4A7W 4A7X 4A7Y 4A7Z 4A80 4A81 4A83 4A84 4A85 4A86 4A87 4A88 4A8E 4A8G 4A8H 4A8I 4A8J 4A8L 4A8N 4A8O 4A8P 4A8R 4A8T 4A8U 4A8V 4A8X 4A8Z 4A90 4A91 4A92 4A94 4A95 4A97 4A98 4A99 4A9A 4A9C 4A9E 4A9F 4A9H 4A9I 4A9J 4A9K 4A9L 4A9M 4A9N 4A9O 4A9R 4A9S 4A9T 4A9U 4A9V 4A9W 4A9X 4A9Y 4A9Z 4AA0 4AA1 4AA2 4AA4 4AA5 4AA7 4AA8 4AA9 4AAA 4AAC 4AAH 4AAJ 4AAL 4AAM 4AAN 4AAO 4AAP 4AAW 4AAX 4AAY 4AAZ 4AB0 4AB1 4AB4 4AB5 4AB6 4AB7 4AB8 4AB9 4ABA 4ABB 4ABD 4ABE 4ABF 4ABG 4ABH 4ABI 4ABJ 4ABK 4ABL 4ABM 4ABN 4ABQ 4ABU 4ABV 4ABW 4ABX 4ABY 4ABZ 4AC0 4AC1 4AC2 4AC3 4AC4 4AC5 4AC6 4AC7 4AC8 4AC9 4ACA 4ACB 4ACC 4ACD 4ACF 4ACG 4ACH 4ACI 4ACJ 4ACK 4ACL 4ACM 4ACO 4ACP 4ACQ 4ACR 4ACS 4ACT 4ACU 4ACV 4ACX 4ACY 4ACZ 4AD0 4AD1 4AD2 4AD3 4AD4 4AD5 4AD6 4AD7 4AD8 4AD9 4ADB 4ADC 4ADD 4ADE 4ADF 4ADG 4ADI 4ADJ 4ADL 4ADM 4ADN 4ADO 4ADP 4ADQ 4ADS 4ADT 4ADU 4ADW 4ADY 4ADZ 4AE0 4AE1 4AE2 4AE3 4AE4 4AE5 4AE6 4AE7 4AE8 4AE9 4AEA 4AEC 4AED 4AEE 4AEF 4AEH 4AEI 4AEJ 4AEK 4AEL 4AEM 4AEN 4AEO 4AEP 4AEQ 4AEX 4AEY 4AEZ 4AF0 4AF1 4AF2 4AF3 4AF5 4AF6 4AF7 4AF8 4AF9 4AFA 4AFB 4AFC 4AFD 4AFE 4AFF 4AFG 4AFH 4AFI 4AFJ 4AFK 4AFL 4AFM 4AFN 4AFP 4AFQ 4AFR 4AFS 4AFT 4AFU 4AFV 4AFX 4AFZ 4AG0 4AG1 4AG2 4AG3 4AG4 4AG5 4AG6 4AG7 4AG8 4AG9 4AGA 4AGC 4AGD 4AGE 4AGF 4AGG 4AGH 4AGI 4AGJ 4AGK 4AGL 4AGM 4AGN 4AGO 4AGP 4AGQ 4AGR 4AGS 4AGT 4AGU 4AGV 4AGW 4AH2 4AH3 4AH4 4AH6 4AH7 4AH9 4AHA 4AHC 4AHD 4AHE 4AHF 4AHG 4AHH 4AHI 4AHJ 4AHK 4AHL 4AHM 4AHN 4AHO 4AHP 4AHQ 4AHR 4AHS 4AHT 4AHU 4AHV 4AHW 4AHX 4AHY 4AHZ 4AI0 4AI1 4AI2 4AI3 4AI4 4AI5 4AI6 4AI7 4AI8 4AI9 4AIA 4AIB 4AIC 4AID 4AIE 4AIF 4AIG 4AIH 4AII 4AIM 4AIN 4AIO 4AIP 4AIQ 4AIR 4AIS 4AIU 4AIV 4AIW 4AIX 4AIZ 4AJ0 4AJ1 4AJ2 4AJ3 4AJ4 4AJ5 4AJ6 4AJ7 4AJ8 4AJ9 4AJA 4AJB 4AJC 4AJD 4AJE 4AJF 4AJG 4AJH 4AJI 4AJJ 4AJK 4AJL 4AJM 4AJN 4AJO 4AJP 4AJR 4AJS 4AJT 4AJU 4AJV 4AJW 4AJX 4AJY 4AJZ 4AK0 4AK1 4AK2 4AK3 4AK4 4AK5 4AK6 4AK7 4AK8 4AK9 4AKB 4AKC 4AKD 4AKE 4AKF 4AKG 4AKH 4AKI 4AKJ 4AKK 4AKL 4AKM 4AKN 4AKO 4AKP 4AKQ 4AKR 4AKS 4AKT 4AKV 4AKX 4AKY 4AKZ 4AL0 4AL1 4AL2 4AL3 4AL4 4AL8 4AL9 4ALA 4ALB 4ALC 4ALD 4ALE 4ALF 4ALG 4ALH 4ALI 4ALJ 4ALK 4ALL 4ALM 4ALN 4ALO 4ALQ 4ALR 4ALS 4ALT 4ALU 4ALV 4ALW 4ALX 4ALY 4ALZ 4AM0 4AM1 4AM2 4AM4 4AM5 4AM6 4AM7 4AM8 4AM9 4AMA 4AMB 4AMC 4AME 4AMF 4AMG 4AMH 4AMI 4AMJ 4AMK 4AML 4AMM 4AMN 4AMO 4AMP 4AMQ 4AMS 4AMT 4AMU 4AMV 4AMW 4AMX 4AMY 4AMZ 4AN0 4AN1 4AN2 4AN3 4AN4 4AN6 4AN7 4AN8 4AN9 4ANB 4ANC 4AND 4ANE 4ANF 4ANI 4ANJ 4ANK 4ANL 4ANM 4ANN 4ANO 4ANP 4ANQ 4ANR 4ANS 4ANU 4ANV 4ANW 4ANX 4AO1 4AO4 4AO5 4AO6 4AO7 4AO8 4AO9 4AOA 4AOC 4AOF 4AOH 4AOI 4AOJ 4AOK 4AOM 4AON 4AOO 4AOP 4AOQ 4AOR 4AOS 4AOT 4AOU 4AOV 4AOW 4AOX 4AOY 4AOZ 4AP0 4AP1 4AP2 4AP3 4AP4 4AP5 4AP6 4AP7 4AP8 4AP9 4APA 4APB 4APC 4APE 4APF 4APH 4APJ 4APL 4APM 4APN 4APO 4APP 4APQ 4APR 4APS 4APT 4APU 4APV 4APX 4APY 4APZ 4AQ0 4AQ1 4AQ2 4AQ3 4AQ4 4AQ6 4AQ8 4AQA 4AQB 4AQC 4AQD 4AQE 4AQF 4AQG 4AQH 4AQI 4AQJ 4AQK 4AQL 4AQN 4AQO 4AQP 4AQQ 4AQR 4AQS 4AQT 4AR1 4AR2 4AR5 4AR6 4AR7 4AR8 4AR9 4ARA 4ARB 4ARE 4ARF 4ARH 4ARJ 4ARK 4ARL 4ARM 4ARN 4ARO 4ARP 4ARQ 4ARR 4ARS 4ART 4ARU 4ARV 4ARW 4ARX 4ARY 4ARZ 4AS0 4AS2 4AS3 4AS4 4AS5 4AS7 4AS8 4AS9 4ASA 4ASB 4ASC 4ASD 4ASE 4ASF 4ASG 4ASH 4ASI 4ASJ 4ASK 4ASL 4ASM 4ASN 4ASQ 4ASR 4AST 4ASU 4ASX 4ASY 4ASZ 4AT0 4AT1 4AT2 4AT3 4AT4 4AT5 4AT6 4AT7 4AT8 4AT9 4ATB 4ATD 4ATE 4ATF 4ATG 4ATH 4ATJ 4ATL 4ATM 4ATN 4ATP 4ATQ 4ATS 4ATT 4ATV 4ATW 4ATY 4ATZ 4AU0 4AU1 4AU2 4AU3 4AU4 4AU5 4AU7 4AU8 4AU9 4AUA 4AUB 4AUC 4AUD 4AUE 4AUI 4AUJ 4AUK 4AUL 4AUM 4AUN 4AUO 4AUP 4AUQ 4AUR 4AUT 4AUU 4AUV 4AUX 4AUY 4AV0 4AV3 4AV4 4AV5 4AV6 4AV7 4AV8 4AV9 4AVA 4AVB 4AVC 4AVD 4AVE 4AVF 4AVG 4AVH 4AVI 4AVJ 4AVK 4AVL 4AVM 4AVN 4AVO 4AVP 4AVQ 4AVR 4AVS 4AVT 4AVU 4AVV 4AVW 4AVX 4AVY 4AVZ 4AW0 4AW1 4AW2 4AW3 4AW4 4AW5 4AW6 4AW7 4AW8 4AW9 4AWA 4AWB 4AWD 4AWE 4AWF 4AWG 4AWH 4AWI 4AWJ 4AWK 4AWM 4AWN 4AWO 4AWP 4AWQ 4AWS 4AWT 4AWU 4AWX 4AWY 4AWZ 4AX0 4AX1 4AX2 4AX3 4AX4 4AX6 4AX7 4AX8 4AX9 4AXA 4AXB 4AXC 4AXD 4AXE 4AXF 4AXG 4AXH 4AXI 4AXJ 4AXK 4AXL 4AXM 4AXN 4AXO 4AXQ 4AXR 4AXS 4AXT 4AXU 4AXV 4AXW 4AXX 4AXY 4AXZ 4AY0 4AY1 4AY3 4AY4 4AY5 4AY6 4AY7 4AY8 4AY9 4AYA 4AYB 4AYC 4AYD 4AYE 4AYF 4AYG 4AYH 4AYI 4AYJ 4AYL 4AYM 4AYN 4AYO 4AYP 4AYQ 4AYR 4AYS 4AYT 4AYU 4AYV 4AYW 4AYX 4AYY 4AYZ 4AZ0 4AZ1 4AZ2 4AZ3 4AZ4 4AZ5 4AZ6 4AZ7 4AZ8 4AZ9 4AZA 4AZB 4AZC 4AZD 4AZE 4AZF 4AZG 4AZH 4AZI 4AZJ 4AZK 4AZL 4AZM 4AZN 4AZO 4AZP 4AZQ 4AZR 4AZS 4AZT 4AZU 4AZV 4AZW 4AZY 4AZZ 4B00 4B02 4B04 4B05 4B08 4B09 4B0A 4B0B 4B0C 4B0D 4B0E 4B0F 4B0G 4B0H 4B0I 4B0J 4B0M 4B0N 4B0O 4B0P 4B0Q 4B0R 4B0S 4B0T 4B0Y 4B0Z 4B10 4B11 4B12 4B13 4B14 4B15 4B16 4B17 4B18 4B1A 4B1B 4B1C 4B1D 4B1E 4B1F 4B1G 4B1H 4B1I 4B1J 4B1L 4B1M 4B1R 4B1T 4B1U 4B1V 4B1W 4B1X 4B1Y 4B1Z 4B27 4B28 4B29 4B2A 4B2B 4B2C 4B2D 4B2F 4B2G 4B2H 4B2I 4B2J 4B2K 4B2L 4B2M 4B2N 4B2O 4B2P 4B2T 4B2W 4B2X 4B2Y 4B2Z 4B30 4B31 4B32 4B33 4B34 4B35 4B36 4B3A 4B3B 4B3C 4B3D 4B3E 4B3F 4B3H 4B3I 4B3J 4B3K 4B3L 4B3N 4B3U 4B3V 4B3W 4B3X 4B3Z 4B40 4B41 4B42 4B43 4B44 4B45 4B46 4B47 4B48 4B49 4B4A 4B4B 4B4C 4B4D 4B4E 4B4F 4B4G 4B4H 4B4I 4B4J 4B4K 4B4L 4B4M 4B4N 4B4O 4B4P 4B4Q 4B4R 4B4S 4B4U 4B4V 4B4W 4B4X 4B4Y 4B4Z 4B50 4B52 4B53 4B54 4B55 4B56 4B5B 4B5C 4B5D 4B5E 4B5K 4B5L 4B5N 4B5O 4B5P 4B5Q 4B5S 4B5T 4B5U 4B5V 4B5W 4B5X 4B5Y 4B5Z 4B60 4B61 4B62 4B63 4B64 4B65 4B66 4B67 4B68 4B69 4B6C 4B6D 4B6E 4B6F 4B6G 4B6H 4B6I 4B6J 4B6L 4B6M 4B6O 4B6P 4B6Q 4B6R 4B6S 4B6W 4B6X 4B6Z 4B70 4B71 4B72 4B73 4B74 4B75 4B76 4B77 4B78 4B79 4B7A 4B7B 4B7C 4B7D 4B7E 4B7F 4B7G 4B7H 4B7I 4B7J 4B7K 4B7L 4B7M 4B7N 4B7O 4B7P 4B7Q 4B7R 4B7S 4B7T 4B7U 4B7V 4B7W 4B7X 4B7Y 4B7Z 4B80 4B81 4B82 4B83 4B84 4B85 4B86 4B87 4B88 4B89 4B8A 4B8B 4B8C 4B8E 4B8J 4B8L 4B8M 4B8N 4B8O 4B8P 4B8R 4B8S 4B8U 4B8V 4B8W 4B8X 4B8Y 4B8Z 4B90 4B91 4B92 4B93 4B94 4B95 4B96 4B97 4B98 4B99 4B9A 4B9B 4B9C 4B9D 4B9E 4B9F 4B9G 4B9H 4B9I 4B9J 4B9K 4B9O 4B9P 4B9Q 4B9R 4B9W 4B9X 4B9Y 4B9Z 4BA0 4BA1 4BA3 4BA4 4BA5 4BA6 4BA7 4BA9 4BAA 4BAB 4BAD 4BAE 4BAF 4BAG 4BAH 4BAI 4BAJ 4BAK 4BAL 4BAM 4BAN 4BAO 4BAP 4BAQ 4BAR 4BAS 4BAT 4BAU 4BAX 4BAY 4BAZ 4BB0 4BB2 4BB3 4BB4 4BB5 4BB6 4BB7 4BB9 4BBA 4BBB 4BBC 4BBD 4BBE 4BBF 4BBG 4BBH 4BBJ 4BBK 4BBM 4BBN 4BBO 4BBP 4BBQ 4BBR 4BBT 4BBU 4BBV 4BBW 4BBX 4BBY 4BBZ 4BC0 4BC1 4BC2 4BC3 4BC4 4BC5 4BC6 4BC7 4BC9 4BCA 4BCB 4BCC 4BCD 4BCE 4BCF 4BCG 4BCH 4BCI 4BCJ 4BCK 4BCL 4BCM 4BCN 4BCO 4BCP 4BCQ 4BCR 4BCS 4BCT 4BCU 4BCW 4BCX 4BCY 4BCZ 4BD0 4BD2 4BD4 4BD6 4BD7 4BD8 4BD9 4BDA 4BDB 4BDC 4BDD 4BDE 4BDF 4BDG 4BDH 4BDI 4BDJ 4BDK 4BDL 4BDM 4BDN 4BDO 4BDQ 4BDR 4BDS 4BDT 4BDU 4BDV 4BDW 4BDX 4BE3 4BE4 4BE5 4BE6 4BE7 4BE8 4BE9 4BEA 4BEB 4BEC 4BEG 4BEI 4BEJ 4BEK 4BEL 4BEM 4BEN 4BEP 4BEQ 4BER 4BES 4BET 4BEU 4BEV 4BEW 4BEX 4BEY 4BEZ 4BF1 4BF2 4BF3 4BF4 4BF5 4BF6 4BF7 4BF9 4BFA 4BFB 4BFC 4BFD 4BFE 4BFF 4BFG 4BFH 4BFI 4BFJ 4BFK 4BFL 4BFM 4BFN 4BFO 4BFP 4BFQ 4BFR 4BFS 4BFT 4BFU 4BFV 4BFW 4BFX 4BFY 4BFZ 4BG0 4BG1 4BG2 4BG4 4BG5 4BG6 4BG7 4BG8 4BG9 4BGA 4BGB 4BGC 4BGD 4BGE 4BGF 4BGG 4BGH 4BGI 4BGJ 4BGK 4BGL 4BGM 4BGO 4BGP 4BGQ 4BGU 4BGV 4BGW 4BGX 4BGY 4BGZ 4BH0 4BH1 4BH2 4BH3 4BH4 4BH5 4BH6 4BH7 4BH8 4BHB 4BHC 4BHD 4BHF 4BHG 4BHI 4BHL 4BHN 4BHQ 4BHR 4BHT 4BHU 4BHV 4BHW 4BHX 4BHY 4BHZ 4BI0 4BI1 4BI2 4BI3 4BI4 4BI5 4BI6 4BI7 4BI8 4BI9 4BIA 4BIB 4BIC 4BID 4BIE 4BIF 4BIG 4BIH 4BII 4BIK 4BIM 4BIN 4BIO 4BIR 4BIS 4BIU 4BIV 4BIW 4BIX 4BIY 4BIZ 4BJ0 4BJ1 4BJ3 4BJ4 4BJ5 4BJ6 4BJ8 4BJ9 4BJA 4BJB 4BJC 4BJH 4BJI 4BJJ 4BJK 4BJL 4BJM 4BJN 4BJO 4BJP 4BJQ 4BJR 4BJS 4BJT 4BJU 4BJX 4BJY 4BJZ 4BK0 4BK1 4BK2 4BK3 4BK4 4BK5 4BK6 4BK7 4BK8 4BK9 4BKA 4BKC 4BKD 4BKE 4BKF 4BKG 4BKJ 4BKL 4BKM 4BKN 4BKO 4BKP 4BKQ 4BKR 4BKS 4BKT 4BKU 4BKW 4BKX 4BKY 4BKZ 4BL0 4BL1 4BL2 4BL3 4BL4 4BL5 4BL6 4BL7 4BL8 4BL9 4BLA 4BLB 4BLC 4BLD 4BLG 4BLI 4BLJ 4BLK 4BLL 4BLM 4BLN 4BLO 4BLP 4BLQ 4BLR 4BLS 4BLT 4BLU 4BLV 4BLW 4BLX 4BLY 4BLZ 4BM0 4BM1 4BM2 4BM3 4BM4 4BM5 4BM7 4BM8 4BM9 4BMA 4BMB 4BMC 4BMD 4BME 4BMG 4BMH 4BMJ 4BMK 4BMM 4BMN 4BMO 4BMP 4BMQ 4BMR 4BMS 4BMT 4BMU 4BMV 4BMW 4BMX 4BMY 4BMZ 4BN0 4BN1 4BN2 4BN4 4BN5 4BN6 4BN7 4BN8 4BN9 4BNB 4BND 4BNE 4BNF 4BNG 4BNH 4BNI 4BNJ 4BNK 4BNL 4BNM 4BNN 4BNP 4BNQ 4BNR 4BNT 4BNU 4BNV 4BNW 4BNX 4BNY 4BNZ 4BO0 4BO1 4BO2 4BO3 4BO4 4BO5 4BO6 4BO7 4BO8 4BO9 4BOB 4BOD 4BOE 4BOF 4BOH 4BOJ 4BOK 4BOL 4BOP 4BOQ 4BOS 4BOU 4BOW 4BOY 4BOZ 4BP0 4BP1 4BP2 4BP3 4BP8 4BP9 4BPA 4BPC 4BPD 4BPF 4BPG 4BPH 4BPI 4BPJ 4BPK 4BPL 4BPM 4BPR 4BPS 4BPT 4BPU 4BPV 4BPW 4BPX 4BPY 4BPZ 4BQ0 4BQ1 4BQ2 4BQ3 4BQ4 4BQ5 4BQ6 4BQ7 4BQ8 4BQ9 4BQB 4BQC 4BQD 4BQE 4BQF 4BQG 4BQH 4BQI 4BQJ 4BQK 4BQL 4BQM 4BQN 4BQO 4BQP 4BQQ 4BQR 4BQS 4BQT 4BQU 4BQV 4BQW 4BQX 4BQY 4BQZ 4BR0 4BR1 4BR2 4BR3 4BR4 4BR5 4BR6 4BR7 4BR9 4BRA 4BRB 4BRC 4BRD 4BRE 4BRF 4BRG 4BRH 4BRI 4BRJ 4BRK 4BRL 4BRM 4BRN 4BRO 4BRP 4BRQ 4BRR 4BRS 4BRU 4BRV 4BRW 4BRX 4BRY 4BRZ 4BS0 4BS3 4BS4 4BS5 4BS6 4BS7 4BS9 4BSA 4BSB 4BSC 4BSD 4BSE 4BSF 4BSG 4BSH 4BSI 4BSJ 4BSK 4BSM 4BSN 4BSO 4BSP 4BSQ 4BSR 4BSS 4BST 4BSU 4BSV 4BSW 4BSX 4BSZ 4BT2 4BT3 4BT4 4BT5 4BT6 4BT7 4BT8 4BT9 4BTA 4BTB 4BTE 4BTF 4BTH 4BTI 4BTJ 4BTK 4BTL 4BTM 4BTP 4BTT 4BTU 4BTV 4BTW 4BTX 4BTY 4BTZ 4BU0 4BU1 4BU2 4BU3 4BU4 4BU5 4BU6 4BU7 4BU8 4BU9 4BUA 4BUB 4BUC 4BUD 4BUE 4BUF 4BUG 4BUH 4BUI 4BUJ 4BUM 4BUO 4BUP 4BUQ 4BUR 4BUS 4BUT 4BUU 4BUV 4BUW 4BUX 4BUY 4BUZ 4BV0 4BV1 4BV2 4BV3 4BV4 4BV5 4BV6 4BV7 4BV8 4BV9 4BVA 4BVB 4BVC 4BVD 4BVE 4BVF 4BVG 4BVH 4BVJ 4BVK 4BVL 4BVM 4BVN 4BVO 4BVP 4BVQ 4BVR 4BVS 4BVT 4BVU 4BVV 4BVW 4BVX 4BVY 4BW1 4BW2 4BW3 4BW4 4BW5 4BW7 4BW8 4BW9 4BWA 4BWB 4BWC 4BWD 4BWE 4BWF 4BWG 4BWI 4BWK 4BWL 4BWN 4BWO 4BWP 4BWQ 4BWR 4BWS 4BWT 4BWU 4BWV 4BWW 4BWX 4BWY 4BWZ 4BX0 4BX2 4BX3 4BX5 4BX6 4BX7 4BX8 4BX9 4BXA 4BXB 4BXC 4BXD 4BXE 4BXF 4BXH 4BXI 4BXJ 4BXK 4BXM 4BXN 4BXP 4BXQ 4BXR 4BXS 4BXT 4BXV 4BXW 4BXZ 4BY0 4BY2 4BY3 4BY4 4BY5 4BY6 4BY8 4BYF 4BYG 4BYH 4BYI 4BYJ 4BYM 4BYY 4BYZ 4BZ0 4BZ1 4BZ2 4BZ3 4BZ4 4BZ5 4BZ6 4BZ7 4BZ8 4BZ9 4BZA 4BZB 4BZC 4BZD 4BZE 4BZF 4BZG 4BZH 4BZN 4BZO 4BZP 4BZQ 4BZR 4BZS 4BZW 4BZX 4BZY 4BZZ 4C00 4C01 4C02 4C03 4C04 4C05 4C06 4C07 4C08 4C09 4C0A 4C0B 4C0C 4C0D 4C0E 4C0F 4C0G 4C0H 4C0J 4C0K 4C0L 4C0M 4C0N 4C0O 4C0P 4C0Q 4C0R 4C0S 4C0T 4C0W 4C0X 4C0Z 4C11 4C12 4C13 4C14 4C16 4C18 4C1A 4C1B 4C1C 4C1D 4C1E 4C1F 4C1G 4C1H 4C1I 4C1K 4C1L 4C1M 4C1N 4C1O 4C1P 4C1Q 4C1R 4C1S 4C1T 4C1U 4C1W 4C1X 4C1Y 4C20 4C21 4C22 4C23 4C24 4C25 4C27 4C28 4C29 4C2A 4C2B 4C2C 4C2D 4C2E 4C2F 4C2G 4C2H 4C2J 4C2K 4C2L 4C2M 4C2N 4C2O 4C2P 4C2Q 4C2R 4C2S 4C2V 4C2W 4C2X 4C2Y 4C2Z 4C31 4C33 4C34 4C35 4C36 4C37 4C38 4C39 4C3A 4C3B 4C3C 4C3D 4C3E 4C3F 4C3H 4C3I 4C3J 4C3K 4C3L 4C3M 4C3O 4C3P 4C3R 4C3S 4C3T 4C3U 4C3V 4C3W 4C3X 4C3Y 4C3Z 4C41 4C43 4C44 4C45 4C46 4C47 4C48 4C49 4C4A 4C4B 4C4C 4C4D 4C4E 4C4F 4C4G 4C4H 4C4I 4C4J 4C4K 4C4M 4C4N 4C4O 4C4P 4C4R 4C4S 4C4T 4C4U 4C4V 4C4X 4C4Y 4C4Z 4C50 4C51 4C52 4C53 4C54 4C55 4C56 4C57 4C58 4C59 4C5A 4C5B 4C5C 4C5D 4C5E 4C5F 4C5G 4C5H 4C5I 4C5J 4C5K 4C5L 4C5M 4C5N 4C5O 4C5P 4C5Q 4C5R 4C5S 4C5U 4C5W 4C5Y 4C5Z 4C60 4C61 4C62 4C65 4C66 4C67 4C68 4C69 4C6A 4C6B 4C6C 4C6D 4C6E 4C6F 4C6G 4C6H 4C6I 4C6J 4C6K 4C6L 4C6M 4C6N 4C6O 4C6P 4C6Q 4C6R 4C6S 4C6T 4C6U 4C6V 4C6W 4C6X 4C6Y 4C6Z 4C70 4C71 4C72 4C73 4C74 4C75 4C76 4C77 4C78 4C79 4C7A 4C7B 4C7D 4C7F 4C7G 4C7H 4C7I 4C7J 4C7K 4C7L 4C7M 4C7N 4C7P 4C7R 4C7T 4C7U 4C7V 4C7W 4C7X 4C7Y 4C7Z 4C80 4C81 4C82 4C83 4C84 4C85 4C86 4C87 4C88 4C89 4C8A 4C8B 4C8C 4C8D 4C8E 4C8F 4C8G 4C8H 4C8I 4C8P 4C8Q 4C8R 4C8S 4C8T 4C8U 4C8V 4C8W 4C8X 4C90 4C91 4C92 4C93 4C94 4C95 4C97 4C98 4C99 4C9A 4C9B 4C9C 4C9E 4C9F 4C9G 4C9H 4C9I 4C9J 4C9K 4C9L 4C9M 4C9N 4C9O 4C9P 4C9Q 4C9R 4C9S 4C9T 4C9U 4C9V 4C9W 4C9X 4C9Y 4C9Z 4CA0 4CA1 4CA2 4CA4 4CA5 4CA6 4CA7 4CA8 4CAA 4CAB 4CAC 4CAD 4CAE 4CAF 4CAG 4CAH 4CAI 4CAJ 4CAM 4CAN 4CAO 4CAP 4CAQ 4CAR 4CAS 4CAT 4CAV 4CAW 4CAX 4CAY 4CAZ 4CB0 4CB4 4CB5 4CB6 4CB7 4CB8 4CB9 4CBA 4CBB 4CBC 4CBE 4CBG 4CBH 4CBI 4CBJ 4CBK 4CBL 4CBM 4CBN 4CBO 4CBP 4CBQ 4CBR 4CBS 4CBT 4CBU 4CBV 4CBW 4CBX 4CBY 4CBZ 4CC0 4CC1 4CC2 4CC3 4CC4 4CC5 4CC6 4CC7 4CC9 4CCA 4CCB 4CCC 4CCD 4CCE 4CCF 4CCG 4CCJ 4CCK 4CCL 4CCM 4CCN 4CCO 4CCP 4CCQ 4CCR 4CCS 4CCU 4CCV 4CCW 4CCX 4CCY 4CCZ 4CD0 4CD1 4CD2 4CD3 4CD4 4CD5 4CD6 4CD7 4CD8 4CDA 4CDB 4CDC 4CDD 4CDE 4CDF 4CDG 4CDH 4CDI 4CDJ 4CDK 4CDL 4CDM 4CDN 4CDO 4CDP 4CDQ 4CDR 4CDT 4CDU 4CDV 4CDW 4CDX 4CDY 4CDZ 4CE0 4CE1 4CE2 4CE3 4CE5 4CE6 4CE7 4CE8 4CE9 4CEA 4CEB 4CEC 4CED 4CEE 4CEF 4CEG 4CEK 4CEL 4CEM 4CEO 4CEQ 4CER 4CES 4CET 4CEU 4CEV 4CEW 4CEX 4CEY 4CEZ 4CF0 4CF1 4CF2 4CF3 4CF4 4CF5 4CF6 4CF7 4CF8 4CF9 4CFA 4CFB 4CFC 4CFD 4CFE 4CFF 4CFG 4CFH 4CFI 4CFK 4CFL 4CFM 4CFN 4CFO 4CFP 4CFQ 4CFR 4CFS 4CFT 4CFU 4CFV 4CFW 4CFX 4CFY 4CFZ 4CG0 4CG1 4CG2 4CG3 4CG4 4CG8 4CG9 4CGA 4CGB 4CGC 4CGD 4CGE 4CGF 4CGG 4CGH 4CGI 4CGJ 4CGK 4CGL 4CGM 4CGN 4CGO 4CGP 4CGQ 4CGR 4CGS 4CGT 4CGU 4CGV 4CGW 4CGX 4CGY 4CH2 4CH3 4CH4 4CH5 4CH6 4CH7 4CH8 4CH9 4CHA 4CHB 4CHC 4CHD 4CHE 4CHF 4CHG 4CHH 4CHI 4CHJ 4CHK 4CHL 4CHM 4CHN 4CHO 4CHP 4CHQ 4CHS 4CHT 4CHX 4CHY 4CHZ 4CI1 4CI2 4CI3 4CI4 4CI5 4CI6 4CI7 4CI8 4CI9 4CIA 4CIB 4CIC 4CID 4CIE 4CIF 4CIG 4CIH 4CII 4CIJ 4CIK 4CIL 4CIM 4CIN 4CIP 4CIT 4CIU 4CIV 4CIW 4CIX 4CIY 4CIZ 4CJ0 4CJ1 4CJ2 4CJ3 4CJ4 4CJ5 4CJ6 4CJ7 4CJ8 4CJ9 4CJB 4CJC 4CJD 4CJE 4CJF 4CJG 4CJK 4CJL 4CJM 4CJN 4CJO 4CJP 4CJQ 4CJR 4CJS 4CJT 4CJU 4CJV 4CJW 4CJX 4CJZ 4CK0 4CK1 4CK2 4CK3 4CK4 4CK8 4CK9 4CKA 4CKB 4CKC 4CKE 4CKI 4CKJ 4CKK 4CKL 4CKM 4CKN 4CKP 4CKQ 4CKR 4CKT 4CKU 4CKV 4CKW 4CKX 4CKY 4CKZ 4CL0 4CL1 4CL2 4CL3 4CL6 4CL7 4CL8 4CL9 4CLA 4CLB 4CLC 4CLD 4CLE 4CLF 4CLH 4CLI 4CLJ 4CLK 4CLL 4CLM 4CLN 4CLO 4CLP 4CLQ 4CLR 4CLS 4CLT 4CLU 4CLV 4CLW 4CLX 4CLY 4CLZ 4CM0 4CM1 4CM2 4CM3 4CM4 4CM5 4CM6 4CM7 4CM8 4CM9 4CMA 4CMB 4CMC 4CMD 4CME 4CMF 4CMG 4CMH 4CMI 4CMJ 4CMK 4CML 4CMM 4CMN 4CMO 4CMP 4CMQ 4CMR 4CMS 4CMT 4CMU 4CMV 4CMW 4CMX 4CMY 4CMZ 4CN0 4CN1 4CN4 4CN6 4CN8 4CN9 4CNB 4CNC 4CND 4CNE 4CNF 4CNG 4CNH 4CNI 4CNJ 4CNK 4CNL 4CNM 4CNN 4CNO 4CNP 4CNQ 4CNR 4CNS 4CNT 4CNU 4CNV 4CNW 4CNX 4CNY 4CNZ 4CO0 4CO1 4CO2 4CO3 4CO4 4CO5 4CO6 4CO7 4CO8 4CO9 4COB 4COC 4COD 4COE 4COF 4COG 4COH 4COI 4COJ 4COK 4COL 4COM 4CON 4COO 4COP 4COQ 4COS 4COT 4COU 4COV 4COW 4COX 4COY 4COZ 4CP0 4CP1 4CP2 4CP3 4CP4 4CP5 4CP6 4CP7 4CP8 4CP9 4CPA 4CPB 4CPC 4CPD 4CPE 4CPF 4CPH 4CPI 4CPK 4CPL 4CPM 4CPN 4CPO 4CPP 4CPQ 4CPR 4CPS 4CPT 4CPU 4CPV 4CPW 4CPX 4CPY 4CPZ 4CQ0 4CQ1 4CQ4 4CQ5 4CQ6 4CQ7 4CQ8 4CQ9 4CQA 4CQB 4CQC 4CQD 4CQE 4CQF 4CQG 4CQH 4CQI 4CQJ 4CQK 4CQL 4CQM 4CQO 4CQP 4CQQ 4CQR 4CQS 4CQU 4CQV 4CQW 4CQX 4CQY 4CQZ 4CR0 4CR5 4CR6 4CR7 4CR8 4CR9 4CRA 4CRB 4CRC 4CRD 4CRE 4CRF 4CRG 4CRH 4CRI 4CRJ 4CRL 4CRQ 4CRR 4CRS 4CRT 4CRU 4CRV 4CRW 4CRY 4CRZ 4CS0 4CS2 4CS3 4CS4 4CS5 4CS6 4CS7 4CS8 4CS9 4CSB 4CSC 4CSD 4CSE 4CSG 4CSH 4CSI 4CSJ 4CSK 4CSM 4CSO 4CSP 4CSR 4CSS 4CST 4CSV 4CSW 4CSY 4CSZ 4CT0 4CT1 4CT2 4CT3 4CT4 4CT5 4CT6 4CT7 4CT8 4CT9 4CTA 4CTB 4CTC 4CTD 4CTE 4CTH 4CTI 4CTJ 4CTK 4CTM 4CTN 4CTO 4CTP 4CTQ 4CTR 4CTS 4CTT 4CTU 4CTV 4CTW 4CTX 4CTY 4CTZ 4CU0 4CU1 4CU2 4CU4 4CU5 4CU6 4CU7 4CU8 4CU9 4CUA 4CUB 4CUC 4CUD 4CUE 4CUF 4CUG 4CUJ 4CUK 4CUL 4CUM 4CUN 4CUO 4CUP 4CUQ 4CUR 4CUS 4CUT 4CUU 4CUZ 4CV0 4CV1 4CV2 4CV3 4CV4 4CV5 4CV7 4CV8 4CV9 4CVA 4CVB 4CVC 4CVD 4CVG 4CVH 4CVK 4CVL 4CVM 4CVN 4CVO 4CVP 4CVQ 4CVR 4CVS 4CVT 4CVU 4CVW 4CVX 4CVY 4CVZ 4CW0 4CW1 4CW2 4CW3 4CW4 4CW5 4CW6 4CW7 4CW8 4CW9 4CWA 4CWB 4CWC 4CWD 4CWE 4CWF 4CWM 4CWN 4CWO 4CWP 4CWQ 4CWR 4CWS 4CWT 4CWU 4CWV 4CWW 4CWX 4CWY 4CWZ 4CX0 4CX1 4CX2 4CX3 4CX4 4CX5 4CX6 4CX7 4CX8 4CX9 4CXA 4CXF 4CXI 4CXJ 4CXK 4CXL 4CXM 4CXN 4CXO 4CXP 4CXQ 4CXR 4CXS 4CXT 4CXU 4CXV 4CXW 4CXX 4CXY 4CY1 4CY2 4CY3 4CY5 4CY6 4CY7 4CY8 4CY9 4CYA 4CYB 4CYD 4CYE 4CYF 4CYG 4CYH 4CYI 4CYJ 4CYM 4CYN 4CYO 4CYP 4CYQ 4CYR 4CYS 4CYU 4CYV 4CYW 4CYY 4CYZ 4CZ0 4CZ1 4CZ2 4CZ5 4CZ6 4CZ7 4CZ8 4CZ9 4CZA 4CZB 4CZC 4CZD 4CZE 4CZF 4CZG 4CZH 4CZI 4CZJ 4CZK 4CZL 4CZM 4CZN 4CZO 4CZP 4CZQ 4CZR 4CZS 4CZT 4CZU 4CZV 4CZW 4CZX 4CZY 4CZZ 4D00 4D01 4D02 4D03 4D04 4D05 4D06 4D07 4D08 4D09 4D0B 4D0C 4D0D 4D0E 4D0F 4D0G 4D0J 4D0K 4D0L 4D0M 4D0N 4D0O 4D0P 4D0Q 4D0R 4D0S 4D0T 4D0U 4D0V 4D0W 4D0X 4D0Y 4D0Z 4D10 4D11 4D12 4D13 4D17 4D18 4D19 4D1A 4D1B 4D1C 4D1D 4D1E 4D1F 4D1G 4D1I 4D1J 4D1L 4D1M 4D1N 4D1O 4D1P 4D1S 4D1T 4D1U 4D1V 4D1W 4D1X 4D1Y 4D1Z 4D28 4D2B 4D2C 4D2D 4D2E 4D2G 4D2H 4D2I 4D2J 4D2K 4D2L 4D2M 4D2N 4D2O 4D2P 4D2R 4D2S 4D2T 4D2V 4D2W 4D2Y 4D2Z 4D30 4D31 4D32 4D33 4D34 4D35 4D36 4D37 4D38 4D39 4D3A 4D3B 4D3C 4D3D 4D3F 4D3G 4D3H 4D3I 4D3J 4D3K 4D3L 4D3M 4D3N 4D3O 4D3P 4D3Q 4D3R 4D3S 4D3T 4D3U 4D3V 4D3W 4D3X 4D3Y 4D3Z 4D40 4D41 4D42 4D43 4D44 4D45 4D46 4D47 4D48 4D49 4D4A 4D4B 4D4C 4D4D 4D4E 4D4F 4D4G 4D4H 4D4I 4D4J 4D4K 4D4L 4D4M 4D4N 4D4O 4D4P 4D4Q 4D4R 4D4S 4D4T 4D4U 4D4V 4D4X 4D4Y 4D4Z 4D50 4D51 4D52 4D53 4D55 4D56 4D57 4D58 4D59 4D5A 4D5B 4D5C 4D5D 4D5E 4D5F 4D5G 4D5H 4D5I 4D5J 4D5K 4D5M 4D5O 4D5P 4D5Q 4D5R 4D5S 4D5T 4D5U 4D5V 4D60 4D62 4D63 4D69 4D6A 4D6B 4D6C 4D6D 4D6E 4D6F 4D6G 4D6H 4D6I 4D6J 4D6K 4D6P 4D6Q 4D6R 4D6S 4D6T 4D6U 4D6V 4D6W 4D6X 4D6Y 4D6Z 4D70 4D71 4D72 4D73 4D74 4D75 4D76 4D77 4D78 4D79 4D7A 4D7B 4D7C 4D7D 4D7E 4D7F 4D7G 4D7H 4D7I 4D7J 4D7K 4D7L 4D7M 4D7N 4D7O 4D7P 4D7Q 4D7R 4D7S 4D7T 4D7U 4D7V 4D7W 4D7Y 4D7Z 4D80 4D81 4D82 4D83 4D85 4D86 4D87 4D88 4D89 4D8A 4D8B 4D8C 4D8D 4D8E 4D8F 4D8G 4D8H 4D8I 4D8K 4D8L 4D8M 4D8N 4D8O 4D8P 4D8S 4D8T 4D8U 4D8V 4D8W 4D8X 4D8Y 4D8Z 4D90 4D91 4D92 4D94 4D96 4D97 4D98 4D99 4D9B 4D9C 4D9E 4D9F 4D9G 4D9H 4D9I 4D9J 4D9K 4D9L 4D9M 4D9N 4D9O 4D9P 4D9Q 4D9R 4D9S 4D9T 4D9U 4D9V 4D9W 4D9Z 4DA0 4DA1 4DA2 4DA5 4DA6 4DA7 4DA8 4DA9 4DAA 4DAB 4DAC 4DAD 4DAE 4DAF 4DAG 4DAI 4DAJ 4DAL 4DAM 4DAN 4DAO 4DAP 4DAR 4DAS 4DAT 4DAU 4DAW 4DAY 4DB1 4DB3 4DB5 4DB6 4DB7 4DB8 4DB9 4DBA 4DBB 4DBC 4DBD 4DBE 4DBF 4DBG 4DBH 4DBK 4DBL 4DBM 4DBN 4DBP 4DBQ 4DBR 4DBS 4DBU 4DBV 4DBW 4DBX 4DBZ 4DC0 4DC1 4DC2 4DC3 4DC4 4DC5 4DC6 4DC7 4DC8 4DC9 4DCA 4DCB 4DCC 4DCD 4DCE 4DCF 4DCG 4DCH 4DCI 4DCJ 4DCK 4DCL 4DCM 4DCN 4DCO 4DCP 4DCQ 4DCS 4DCT 4DCU 4DCV 4DCX 4DCY 4DCZ 4DD0 4DD1 4DD2 4DD3 4DD4 4DD5 4DD6 4DD7 4DD8 4DD9 4DDA 4DDB 4DDC 4DDD 4DDF 4DDG 4DDH 4DDI 4DDJ 4DDK 4DDL 4DDM 4DDN 4DDO 4DDP 4DDQ 4DDR 4DDS 4DDT 4DDU 4DDV 4DDW 4DDX 4DDY 4DDZ 4DE0 4DE1 4DE2 4DE3 4DE4 4DE5 4DE6 4DE7 4DE8 4DE9 4DEA 4DEB 4DEC 4DED 4DEE 4DEF 4DEG 4DEH 4DEI 4DEJ 4DEL 4DEM 4DEN 4DEP 4DEQ 4DER 4DES 4DET 4DEU 4DEV 4DEW 4DEX 4DEY 4DEZ 4DF0 4DF1 4DF2 4DF3 4DF6 4DF7 4DF9 4DFA 4DFB 4DFC 4DFD 4DFE 4DFF 4DFG 4DFH 4DFI 4DFL 4DFN 4DFR 4DFS 4DFU 4DFW 4DFX 4DFY 4DFZ 4DG0 4DG1 4DG2 4DG3 4DG4 4DG5 4DG6 4DG7 4DG8 4DG9 4DGA 4DGB 4DGC 4DGD 4DGE 4DGF 4DGG 4DGH 4DGI 4DGJ 4DGK 4DGL 4DGM 4DGN 4DGO 4DGP 4DGQ 4DGR 4DGS 4DGT 4DGU 4DGV 4DGW 4DGX 4DGY 4DGZ 4DH0 4DH1 4DH2 4DH3 4DH4 4DH5 4DH6 4DH7 4DH8 4DHD 4DHE 4DHF 4DHG 4DHI 4DHJ 4DHK 4DHL 4DHM 4DHN 4DHO 4DHP 4DHQ 4DHR 4DHS 4DHT 4DHU 4DHV 4DHW 4DHX 4DHY 4DHZ 4DI0 4DI1 4DI2 4DI3 4DI4 4DI5 4DI6 4DI8 4DI9 4DIA 4DIB 4DID 4DIE 4DIF 4DIG 4DIJ 4DIK 4DIL 4DIM 4DIN 4DIO 4DIP 4DIQ 4DIR 4DIT 4DIU 4DIX 4DIY 4DIZ 4DJ0 4DJ1 4DJ2 4DJ3 4DJ4 4DJ5 4DJ6 4DJ7 4DJ8 4DJ9 4DJA 4DJB 4DJC 4DJD 4DJE 4DJF 4DJG 4DJH 4DJI 4DJJ 4DJK 4DJL 4DJM 4DJN 4DJO 4DJP 4DJQ 4DJR 4DJS 4DJT 4DJU 4DJV 4DJW 4DJX 4DJY 4DJZ 4DK0 4DK1 4DK2 4DK3 4DK4 4DK5 4DK6 4DK7 4DK8 4DKA 4DKB 4DKC 4DKD 4DKE 4DKF 4DKI 4DKK 4DKL 4DKM 4DKN 4DKO 4DKP 4DKQ 4DKR 4DKS 4DKT 4DKU 4DKV 4DKW 4DKX 4DKY 4DL0 4DL1 4DL8 4DL9 4DLA 4DLB 4DLC 4DLD 4DLF 4DLH 4DLI 4DLJ 4DLK 4DLL 4DLM 4DLN 4DLO 4DLP 4DLQ 4DLR 4DLS 4DLT 4DLU 4DLV 4DLW 4DLX 4DLY 4DLZ 4DM1 4DM2 4DM3 4DM4 4DM5 4DM6 4DM7 4DM8 4DM9 4DMA 4DMB 4DMC 4DMD 4DME 4DMF 4DMG 4DMH 4DMI 4DMK 4DML 4DMM 4DMN 4DMO 4DMR 4DMT 4DMU 4DMV 4DMW 4DMX 4DMY 4DMZ 4DN0 4DN1 4DN2 4DN3 4DN4 4DN5 4DN6 4DN7 4DN8 4DN9 4DNA 4DNC 4DND 4DNE 4DNF 4DNG 4DNH 4DNI 4DNJ 4DNK 4DNL 4DNM 4DNN 4DNO 4DNP 4DNQ 4DNR 4DNS 4DNT 4DNU 4DNV 4DNW 4DNX 4DNY 4DNZ 4DO0 4DO1 4DO2 4DO3 4DO4 4DO5 4DO6 4DO7 4DO8 4DOD 4DOE 4DOF 4DOG 4DOH 4DOI 4DOJ 4DOK 4DOL 4DOM 4DON 4DOO 4DOP 4DOQ 4DOR 4DOS 4DOT 4DOU 4DOV 4DOW 4DOX 4DOY 4DOZ 4DP0 4DP1 4DP2 4DP3 4DP4 4DP5 4DP6 4DP7 4DP8 4DP9 4DPA 4DPB 4DPC 4DPD 4DPE 4DPF 4DPG 4DPH 4DPI 4DPK 4DPL 4DPM 4DPN 4DPO 4DPP 4DPQ 4DPR 4DPT 4DPU 4DPW 4DPX 4DPY 4DPZ 4DQ0 4DQ1 4DQ2 4DQ3 4DQ4 4DQ5 4DQ6 4DQ7 4DQ8 4DQ9 4DQA 4DQB 4DQC 4DQD 4DQE 4DQF 4DQG 4DQH 4DQJ 4DQK 4DQL 4DQM 4DQN 4DQO 4DQU 4DQV 4DQW 4DQX 4DQZ 4DR0 4DR8 4DR9 4DRA 4DRB 4DRE 4DRF 4DRH 4DRI 4DRJ 4DRK 4DRM 4DRN 4DRO 4DRP 4DRQ 4DRR 4DRS 4DRT 4DRU 4DRV 4DRW 4DRX 4DRY 4DRZ 4DS0 4DS1 4DS2 4DS3 4DS7 4DS8 4DSA 4DSB 4DSC 4DSD 4DSG 4DSH 4DSN 4DSO 4DSQ 4DSR 4DSS 4DST 4DSU 4DSY 4DSZ 4DT0 4DT1 4DT2 4DT3 4DT4 4DT5 4DT6 4DT7 4DT8 4DT9 4DTA 4DTB 4DTC 4DTD 4DTE 4DTF 4DTG 4DTH 4DTI 4DTK 4DTL 4DTT 4DTW 4DTY 4DTZ 4DU0 4DU2 4DU5 4DU6 4DU7 4DU8 4DU9 4DUA 4DUB 4DUC 4DUD 4DUE 4DUF 4DUG 4DUH 4DUI 4DUK 4DUL 4DUM 4DUN 4DUO 4DUP 4DUQ 4DUR 4DUS 4DUT 4DUU 4DUV 4DUW 4DUX 4DV8 4DV9 4DVA 4DVB 4DVC 4DVD 4DVE 4DVF 4DVG 4DVH 4DVI 4DVJ 4DVK 4DVL 4DVN 4DVQ 4DVR 4DVS 4DVT 4DVV 4DVW 4DVX 4DVY 4DVZ 4DW0 4DW1 4DW2 4DW3 4DW4 4DW5 4DW6 4DW7 4DW8 4DWB 4DWC 4DWD 4DWE 4DWF 4DWG 4DWH 4DWJ 4DWK 4DWL 4DWM 4DWN 4DWO 4DWQ 4DWR 4DWS 4DWT 4DWU 4DWV 4DWW 4DWX 4DWZ 4DX0 4DX1 4DX2 4DX3 4DX5 4DX6 4DX7 4DX8 4DX9 4DXA 4DXB 4DXC 4DXD 4DXE 4DXF 4DXG 4DXH 4DXI 4DXJ 4DXK 4DXL 4DXM 4DXN 4DXO 4DXP 4DXQ 4DXR 4DXS 4DXT 4DXU 4DXV 4DXW 4DXX 4DXY 4DXZ 4DY0 4DY1 4DY3 4DY4 4DY5 4DY6 4DY7 4DY9 4DYA 4DYB 4DYC 4DYD 4DYE 4DYG 4DYH 4DYJ 4DYK 4DYL 4DYM 4DYN 4DYO 4DYP 4DYQ 4DYR 4DYS 4DYT 4DYU 4DYV 4DYW 4DYX 4DYY 4DYZ 4DZ0 4DZ1 4DZ2 4DZ3 4DZ4 4DZ5 4DZ6 4DZ7 4DZ8 4DZ9 4DZA 4DZB 4DZD 4DZG 4DZH 4DZI 4DZJ 4DZK 4DZL 4DZM 4DZN 4DZO 4DZP 4DZR 4DZT 4DZU 4DZV 4DZW 4DZY 4DZZ 4E00 4E01 4E02 4E03 4E04 4E05 4E06 4E07 4E08 4E09 4E0A 4E0B 4E0C 4E0E 4E0F 4E0H 4E0I 4E0K 4E0L 4E0M 4E0N 4E0O 4E0Q 4E0R 4E0S 4E0T 4E0U 4E0V 4E0W 4E0X 4E11 4E12 4E13 4E14 4E15 4E16 4E17 4E18 4E19 4E1A 4E1B 4E1C 4E1D 4E1E 4E1F 4E1G 4E1H 4E1I 4E1J 4E1K 4E1L 4E1M 4E1N 4E1O 4E1P 4E1Q 4E1R 4E1S 4E1T 4E1V 4E1Y 4E1Z 4E20 4E21 4E22 4E26 4E27 4E28 4E29 4E2A 4E2B 4E2C 4E2D 4E2E 4E2F 4E2G 4E2H 4E2I 4E2J 4E2K 4E2L 4E2O 4E2P 4E2Q 4E2S 4E2T 4E2U 4E2V 4E2W 4E2X 4E2Y 4E2Z 4E30 4E31 4E32 4E33 4E34 4E35 4E36 4E37 4E38 4E3A 4E3B 4E3C 4E3D 4E3E 4E3F 4E3G 4E3H 4E3I 4E3J 4E3K 4E3L 4E3M 4E3N 4E3O 4E3Q 4E3R 4E3T 4E3U 4E3V 4E3W 4E3X 4E3Y 4E3Z 4E40 4E41 4E42 4E43 4E44 4E45 4E46 4E47 4E49 4E4A 4E4B 4E4C 4E4D 4E4E 4E4F 4E4G 4E4H 4E4J 4E4K 4E4L 4E4M 4E4N 4E4P 4E4Q 4E4R 4E4S 4E4T 4E4U 4E4V 4E4W 4E4X 4E4Y 4E4Z 4E50 4E51 4E52 4E53 4E55 4E56 4E57 4E5A 4E5B 4E5D 4E5E 4E5F 4E5G 4E5H 4E5I 4E5J 4E5K 4E5L 4E5M 4E5N 4E5O 4E5P 4E5Q 4E5R 4E5S 4E5T 4E5U 4E5V 4E5W 4E5X 4E5Y 4E61 4E67 4E69 4E6A 4E6C 4E6D 4E6E 4E6F 4E6H 4E6I 4E6K 4E6M 4E6N 4E6P 4E6Q 4E6R 4E6S 4E6T 4E6U 4E6W 4E6X 4E6Y 4E6Z 4E70 4E71 4E72 4E73 4E74 4E75 4E76 4E77 4E79 4E7B 4E7C 4E7D 4E7E 4E7F 4E7G 4E7N 4E7O 4E7P 4E7R 4E7S 4E7T 4E7U 4E7V 4E7W 4E7X 4E7Z 4E80 4E81 4E82 4E83 4E84 4E85 4E86 4E88 4E89 4E8A 4E8B 4E8C 4E8D 4E8E 4E8F 4E8G 4E8H 4E8O 4E8U 4E8W 4E8Y 4E8Z 4E90 4E91 4E92 4E93 4E94 4E96 4E97 4E98 4E99 4E9A 4E9B 4E9C 4E9D 4E9E 4E9I 4E9J 4E9K 4E9L 4E9M 4E9O 4E9Q 4E9R 4E9S 4E9T 4E9U 4E9V 4E9W 4E9X 4E9Y 4E9Z 4EA0 4EA1 4EA2 4EA3 4EA6 4EA7 4EA8 4EA9 4EAA 4EAB 4EAC 4EAD 4EAE 4EAF 4EAG 4EAH 4EAI 4EAJ 4EAK 4EAL 4EAM 4EAN 4EAQ 4EAR 4EAT 4EAW 4EAX 4EAY 4EAZ 4EB0 4EB1 4EB2 4EB3 4EB4 4EB5 4EB6 4EB7 4EB8 4EB9 4EBA 4EBB 4EBF 4EBG 4EBJ 4EBK 4EBL 4EBN 4EBP 4EBQ 4EBR 4EBU 4EBV 4EBW 4EBY 4EBZ 4EC0 4EC2 4EC3 4EC4 4EC5 4EC6 4EC7 4EC8 4EC9 4ECA 4ECB 4ECC 4ECD 4ECE 4ECF 4ECG 4ECH 4ECI 4ECJ 4ECK 4ECL 4ECM 4ECN 4ECO 4ECP 4ED4 4ED9 4EDA 4EDB 4EDE 4EDF 4EDG 4EDH 4EDI 4EDJ 4EDK 4EDL 4EDM 4EDN 4EDO 4EDP 4EDQ 4EDR 4EDS 4EDT 4EDU 4EDV 4EDW 4EDX 4EDY 4EDZ 4EE0 4EE1 4EE2 4EE3 4EE4 4EE5 4EE6 4EE7 4EE8 4EE9 4EEA 4EEB 4EEC 4EED 4EEE 4EEF 4EEG 4EEH 4EEI 4EEJ 4EEK 4EEL 4EEM 4EEN 4EEO 4EEP 4EEQ 4EER 4EES 4EET 4EEU 4EEV 4EEW 4EEX 4EEZ 4EF0 4EF1 4EF2 4EF3 4EF4 4EF5 4EF6 4EF8 4EF9 4EFA 4EFB 4EFC 4EFD 4EFE 4EFF 4EFG 4EFH 4EFI 4EFK 4EFL 4EFM 4EFN 4EFO 4EFP 4EFQ 4EFR 4EFS 4EFT 4EFU 4EFV 4EFX 4EFZ 4EG0 4EG1 4EG2 4EG3 4EG4 4EG5 4EG6 4EG7 4EG8 4EG9 4EGA 4EGB 4EGC 4EGD 4EGE 4EGF 4EGG 4EGH 4EGI 4EGJ 4EGK 4EGL 4EGM 4EGN 4EGO 4EGP 4EGQ 4EGR 4EGS 4EGT 4EGU 4EGV 4EGW 4EGX 4EH1 4EH2 4EH3 4EH4 4EH5 4EH6 4EH7 4EH8 4EH9 4EHA 4EHB 4EHC 4EHD 4EHE 4EHF 4EHG 4EHH 4EHI 4EHJ 4EHK 4EHL 4EHM 4EHN 4EHP 4EHQ 4EHR 4EHS 4EHT 4EHU 4EHV 4EHW 4EHX 4EHY 4EHZ 4EI0 4EI2 4EI4 4EI5 4EI6 4EI7 4EI8 4EI9 4EIA 4EIB 4EIC 4EID 4EIE 4EIF 4EIG 4EIH 4EII 4EIJ 4EIK 4EIL 4EIN 4EIP 4EIQ 4EIR 4EIS 4EIT 4EIU 4EIV 4EIW 4EIX 4EIY 4EIZ 4EJ0 4EJ1 4EJ2 4EJ3 4EJ4 4EJ5 4EJ6 4EJ7 4EJ8 4EJD 4EJE 4EJF 4EJG 4EJH 4EJI 4EJJ 4EJK 4EJL 4EJM 4EJN 4EJO 4EJQ 4EJR 4EJS 4EJU 4EJV 4EJW 4EJX 4EK0 4EK1 4EK2 4EK3 4EK4 4EK5 4EK6 4EK7 4EK8 4EK9 4EKA 4EKB 4EKC 4EKD 4EKE 4EKF 4EKG 4EKH 4EKI 4EKJ 4EKK 4EKL 4EKN 4EKO 4EKP 4EKQ 4EKR 4EKS 4EKT 4EKU 4EKV 4EKW 4EKX 4EKY 4EKZ 4EL0 4EL1 4EL2 4EL3 4EL4 4EL5 4EL6 4EL7 4EL8 4EL9 4ELA 4ELB 4ELC 4ELD 4ELE 4ELF 4ELG 4ELH 4ELJ 4ELK 4ELL 4ELM 4ELN 4ELO 4ELP 4ELQ 4ELR 4ELS 4ELW 4ELX 4ELY 4ELZ 4EM0 4EM1 4EM2 4EM3 4EM4 4EM5 4EM6 4EM7 4EM8 4EM9 4EMA 4EMB 4EMC 4EMD 4EME 4EMF 4EMG 4EMH 4EMI 4EMJ 4EMK 4EML 4EMM 4EMN 4EMO 4EMP 4EMQ 4EMR 4EMS 4EMT 4EMU 4EMV 4EMW 4EMX 4EMY 4EMZ 4EN0 4EN1 4EN2 4EN3 4EN4 4EN6 4EN7 4EN8 4EN9 4END 4ENE 4ENF 4ENG 4ENH 4ENL 4ENO 4ENP 4ENQ 4ENR 4ENS 4ENT 4ENU 4ENV 4ENW 4ENX 4ENY 4ENZ 4EO0 4EO1 4EO2 4EO3 4EO4 4EO5 4EO6 4EO7 4EO8 4EO9 4EOB 4EOC 4EOD 4EOE 4EOF 4EOG 4EOH 4EOI 4EOJ 4EOK 4EOL 4EOM 4EON 4EOO 4EOP 4EOQ 4EOR 4EOS 4EOU 4EOW 4EOX 4EOY 4EOZ 4EP0 4EP1 4EP2 4EP3 4EP4 4EP5 4EP6 4EP7 4EP8 4EP9 4EPA 4EPB 4EPC 4EPD 4EPE 4EPF 4EPH 4EPI 4EPJ 4EPK 4EPL 4EPM 4EPP 4EPQ 4EPR 4EPS 4EPT 4EPU 4EPV 4EPW 4EPX 4EPY 4EPZ 4EQ0 4EQ1 4EQ2 4EQ3 4EQ4 4EQ5 4EQ6 4EQ7 4EQ8 4EQ9 4EQA 4EQB 4EQC 4EQE 4EQF 4EQG 4EQH 4EQI 4EQJ 4EQK 4EQL 4EQM 4EQN 4EQO 4EQP 4EQQ 4EQR 4EQS 4EQU 4EQV 4EQW 4EQX 4EQY 4EQZ 4ER0 4ER1 4ER2 4ER3 4ER4 4ER5 4ER6 4ER7 4ER9 4ERA 4ERC 4ERE 4ERF 4ERG 4ERH 4ERI 4ERK 4ERM 4ERN 4ERO 4ERP 4ERQ 4ERR 4ERS 4ERT 4ERU 4ERV 4ERW 4ERX 4ERY 4ERZ 4ES0 4ES1 4ES2 4ES3 4ES4 4ES5 4ES6 4ES7 4ES8 4ES9 4ESA 4ESB 4ESE 4ESF 4ESG 4ESH 4ESI 4ESK 4ESM 4ESN 4ESO 4ESP 4ESQ 4ESR 4ESS 4EST 4ESU 4ESW 4ESX 4ESY 4ET0 4ET7 4ET8 4ET9 4ETA 4ETB 4ETC 4ETD 4ETE 4ETG 4ETI 4ETJ 4ETK 4ETL 4ETM 4ETN 4ETO 4ETP 4ETQ 4ETR 4ETS 4ETT 4ETU 4ETV 4ETW 4ETX 4ETY 4ETZ 4EU0 4EU1 4EU2 4EU3 4EU4 4EU5 4EU6 4EU7 4EU8 4EU9 4EUA 4EUB 4EUC 4EUD 4EUE 4EUF 4EUG 4EUH 4EUI 4EUK 4EUL 4EUM 4EUN 4EUO 4EUP 4EUS 4EUT 4EUU 4EUV 4EUX 4EUY 4EUZ 4EV0 4EV1 4EV2 4EV4 4EV5 4EV6 4EV8 4EV9 4EVA 4EVB 4EVC 4EVD 4EVE 4EVF 4EVG 4EVH 4EVI 4EVM 4EVN 4EVO 4EVP 4EVQ 4EVR 4EVS 4EVT 4EVU 4EVW 4EVX 4EVY 4EVZ 4EW1 4EW2 4EW3 4EW5 4EW6 4EW7 4EW9 4EWA 4EWC 4EWD 4EWE 4EWF 4EWG 4EWH 4EWI 4EWJ 4EWL 4EWN 4EWO 4EWP 4EWQ 4EWR 4EWS 4EWT 4EWV 4EWW 4EWX 4EWZ 4EX0 4EX1 4EX4 4EX5 4EX6 4EX7 4EX8 4EX9 4EXA 4EXB 4EXG 4EXH 4EXJ 4EXK 4EXL 4EXM 4EXN 4EXO 4EXP 4EXQ 4EXR 4EXS 4EXT 4EXV 4EXW 4EXX 4EXY 4EXZ 4EY0 4EY1 4EY2 4EY3 4EY4 4EY5 4EY6 4EY7 4EY8 4EY9 4EYB 4EYC 4EYD 4EYE 4EYF 4EYG 4EYJ 4EYK 4EYL 4EYM 4EYN 4EYO 4EYP 4EYQ 4EYR 4EYS 4EYT 4EYU 4EYV 4EYW 4EYY 4EYZ 4EZ1 4EZ3 4EZ4 4EZ5 4EZ7 4EZ8 4EZA 4EZB 4EZC 4EZD 4EZE 4EZF 4EZG 4EZH 4EZI 4EZJ 4EZK 4EZL 4EZM 4EZN 4EZO 4EZP 4EZQ 4EZR 4EZS 4EZT 4EZU 4EZV 4EZW 4EZX 4EZY 4EZZ 4F00 4F01 4F03 4F04 4F06 4F07 4F08 4F09 4F0A 4F0B 4F0C 4F0D 4F0E 4F0F 4F0G 4F0H 4F0I 4F0J 4F0K 4F0L 4F0M 4F0N 4F0O 4F0P 4F0Q 4F0R 4F0S 4F0T 4F0U 4F0V 4F0W 4F0X 4F0Y 4F0Z 4F10 4F11 4F12 4F13 4F14 4F15 4F16 4F17 4F18 4F19 4F1A 4F1B 4F1C 4F1D 4F1E 4F1F 4F1G 4F1I 4F1J 4F1K 4F1L 4F1M 4F1O 4F1P 4F1Q 4F1R 4F1S 4F1T 4F1U 4F1V 4F1W 4F1Y 4F1Z 4F20 4F21 4F22 4F23 4F24 4F25 4F26 4F27 4F28 4F29 4F2A 4F2B 4F2C 4F2D 4F2E 4F2F 4F2G 4F2H 4F2I 4F2K 4F2L 4F2M 4F2N 4F2O 4F2P 4F2Q 4F2T 4F2U 4F2V 4F2W 4F2Z 4F30 4F31 4F32 4F33 4F34 4F35 4F36 4F37 4F38 4F39 4F3A 4F3B 4F3C 4F3D 4F3E 4F3F 4F3G 4F3H 4F3I 4F3J 4F3K 4F3L 4F3M 4F3N 4F3P 4F3Q 4F3R 4F3S 4F3V 4F3W 4F3X 4F3Y 4F3Z 4F40 4F42 4F44 4F45 4F46 4F47 4F48 4F49 4F4A 4F4B 4F4C 4F4D 4F4E 4F4F 4F4H 4F4I 4F4J 4F4L 4F4M 4F4O 4F4P 4F4Q 4F4R 4F4S 4F4T 4F4U 4F4V 4F51 4F52 4F53 4F54 4F55 4F56 4F57 4F58 4F59 4F5A 4F5B 4F5C 4F5D 4F5E 4F5F 4F5G 4F5H 4F5I 4F5J 4F5K 4F5L 4F5M 4F5S 4F5T 4F5U 4F5V 4F5W 4F5X 4F5Y 4F5Z 4F60 4F61 4F62 4F63 4F64 4F65 4F66 4F67 4F68 4F69 4F6A 4F6B 4F6C 4F6D 4F6E 4F6F 4F6G 4F6H 4F6I 4F6J 4F6L 4F6O 4F6P 4F6R 4F6S 4F6T 4F6U 4F6V 4F6W 4F6X 4F6Z 4F70 4F71 4F72 4F73 4F74 4F75 4F76 4F78 4F79 4F7A 4F7B 4F7C 4F7D 4F7E 4F7F 4F7G 4F7H 4F7I 4F7J 4F7K 4F7L 4F7M 4F7N 4F7O 4F7P 4F7R 4F7S 4F7T 4F7U 4F7V 4F7W 4F7X 4F7Z 4F80 4F82 4F83 4F84 4F85 4F86 4F87 4F88 4F8A 4F8B 4F8C 4F8D 4F8E 4F8F 4F8H 4F8J 4F8K 4F8L 4F8M 4F8N 4F8O 4F8P 4F8Q 4F8T 4F8X 4F8Y 4F8Z 4F91 4F92 4F93 4F94 4F95 4F96 4F97 4F98 4F99 4F9A 4F9B 4F9C 4F9D 4F9E 4F9F 4F9G 4F9J 4F9K 4F9L 4F9M 4F9N 4F9O 4F9P 4F9T 4F9U 4F9V 4F9W 4F9Y 4F9Z 4FA0 4FA1 4FA2 4FA3 4FA4 4FA5 4FA6 4FA7 4FA8 4FA9 4FAA 4FAB 4FAC 4FAD 4FAE 4FAF 4FAG 4FAH 4FAI 4FAJ 4FAK 4FAL 4FAM 4FAN 4FAO 4FAP 4FAS 4FAT 4FAV 4FAY 4FAZ 4FB1 4FB2 4FB4 4FB5 4FB7 4FB8 4FB9 4FBA 4FBB 4FBC 4FBD 4FBE 4FBF 4FBG 4FBH 4FBI 4FBJ 4FBK 4FBL 4FBM 4FBN 4FBO 4FBP 4FBQ 4FBR 4FBS 4FBV 4FBW 4FBX 4FBY 4FBZ 4FC0 4FC2 4FC3 4FC4 4FC5 4FC6 4FC7 4FC8 4FC9 4FCA 4FCB 4FCC 4FCD 4FCE 4FCF 4FCG 4FCH 4FCI 4FCJ 4FCK 4FCM 4FCN 4FCO 4FCP 4FCQ 4FCR 4FCS 4FCT 4FCU 4FCV 4FCW 4FCX 4FCZ 4FD0 4FD2 4FD3 4FD4 4FD5 4FD6 4FD7 4FD8 4FD9 4FDA 4FDB 4FDC 4FDD 4FDF 4FDG 4FDH 4FDI 4FDJ 4FDK 4FDL 4FDM 4FDN 4FDO 4FDP 4FDQ 4FDT 4FDU 4FDV 4FDW 4FDX 4FDY 4FDZ 4FE1 4FE2 4FE3 4FE4 4FE6 4FE7 4FE8 4FE9 4FEA 4FEB 4FEC 4FED 4FEE 4FEF 4FEG 4FEH 4FEI 4FEK 4FEM 4FEQ 4FER 4FES 4FET 4FEU 4FEV 4FEW 4FEX 4FEY 4FEZ 4FF5 4FF6 4FF7 4FF8 4FF9 4FFA 4FFB 4FFC 4FFD 4FFE 4FFF 4FFG 4FFH 4FFI 4FFJ 4FFK 4FFL 4FFM 4FFN 4FFO 4FFP 4FFR 4FFS 4FFT 4FFU 4FFV 4FFW 4FFX 4FFY 4FFZ 4FG0 4FG2 4FG3 4FG4 4FG5 4FG6 4FG7 4FG8 4FG9 4FGA 4FGB 4FGC 4FGD 4FGE 4FGF 4FGG 4FGH 4FGI 4FGJ 4FGK 4FGL 4FGM 4FGO 4FGP 4FGQ 4FGR 4FGS 4FGT 4FGU 4FGV 4FGW 4FGX 4FGY 4FGZ 4FH0 4FH1 4FH2 4FH3 4FH4 4FH5 4FH6 4FH7 4FH8 4FHA 4FHB 4FHC 4FHD 4FHE 4FHF 4FHG 4FHH 4FHI 4FHJ 4FHK 4FHL 4FHM 4FHN 4FHO 4FHP 4FHQ 4FHR 4FHT 4FHV 4FHW 4FHX 4FHY 4FHZ 4FI1 4FI3 4FI4 4FI5 4FI6 4FI7 4FI8 4FI9 4FIA 4FIB 4FIC 4FID 4FIE 4FIF 4FIG 4FIH 4FII 4FIJ 4FIK 4FIL 4FIM 4FIN 4FIO 4FIP 4FIQ 4FIR 4FIS 4FIT 4FIU 4FIV 4FIW 4FIX 4FIY 4FIZ 4FJ0 4FJ1 4FJ2 4FJ3 4FJ4 4FJ6 4FJC 4FJO 4FJP 4FJQ 4FJR 4FJS 4FJU 4FJV 4FJW 4FJY 4FJZ 4FK1 4FK3 4FK5 4FK6 4FK7 4FK8 4FK9 4FKA 4FKB 4FKC 4FKD 4FKE 4FKG 4FKH 4FKI 4FKJ 4FKK 4FKL 4FKM 4FKO 4FKP 4FKQ 4FKR 4FKS 4FKT 4FKU 4FKV 4FKW 4FKX 4FKY 4FKZ 4FL0 4FL1 4FL2 4FL3 4FL4 4FL5 4FL6 4FL7 4FL8 4FL9 4FLA 4FLB 4FLC 4FLE 4FLF 4FLG 4FLH 4FLI 4FLJ 4FLK 4FLL 4FLM 4FLN 4FLO 4FLP 4FLQ 4FLR 4FLS 4FM3 4FM4 4FM5 4FM6 4FM7 4FM8 4FMA 4FMB 4FMC 4FMD 4FME 4FMF 4FMG 4FMH 4FMI 4FMJ 4FMK 4FML 4FMM 4FMN 4FMO 4FMP 4FMQ 4FMR 4FMS 4FMT 4FMU 4FMV 4FMW 4FMX 4FMZ 4FN0 4FN2 4FN3 4FN4 4FN5 4FN6 4FN7 4FN8 4FN9 4FNA 4FNB 4FND 4FNE 4FNF 4FNG 4FNH 4FNI 4FNK 4FNL 4FNM 4FNN 4FNO 4FNP 4FNQ 4FNR 4FNS 4FNT 4FNU 4FNV 4FNW 4FNX 4FNY 4FNZ 4FO0 4FO1 4FO2 4FO4 4FO5 4FO7 4FO8 4FO9 4FOA 4FOB 4FOC 4FOD 4FOE 4FOF 4FOG 4FOI 4FOJ 4FOK 4FOL 4FOM 4FON 4FOO 4FOP 4FOQ 4FOR 4FOS 4FOT 4FOU 4FOV 4FOW 4FOX 4FOY 4FOZ 4FP1 4FP2 4FP3 4FP4 4FP5 4FP7 4FP8 4FP9 4FPA 4FPB 4FPC 4FPD 4FPE 4FPF 4FPG 4FPH 4FPI 4FPJ 4FPK 4FPL 4FPO 4FPP 4FPR 4FPS 4FPT 4FPW 4FPX 4FPY 4FPZ 4FQ0 4FQ1 4FQ2 4FQ3 4FQ4 4FQ5 4FQ7 4FQ8 4FQ9 4FQA 4FQB 4FQC 4FQD 4FQE 4FQF 4FQG 4FQH 4FQI 4FQJ 4FQK 4FQL 4FQM 4FQN 4FQO 4FQP 4FQQ 4FQR 4FQS 4FQT 4FQU 4FQV 4FQW 4FQX 4FQY 4FQZ 4FR0 4FR1 4FR2 4FR3 4FR4 4FR5 4FR6 4FR7 4FR8 4FR9 4FRA 4FRB 4FRC 4FRD 4FRE 4FRF 4FRH 4FRI 4FRJ 4FRK 4FRL 4FRM 4FRO 4FRP 4FRQ 4FRR 4FRS 4FRT 4FRU 4FRV 4FRW 4FRX 4FRY 4FRZ 4FS0 4FS3 4FS4 4FS7 4FS8 4FS9 4FSA 4FSB 4FSC 4FSD 4FSE 4FSF 4FSH 4FSK 4FSL 4FSM 4FSN 4FSO 4FSP 4FSQ 4FSR 4FSS 4FST 4FSU 4FSV 4FSW 4FSX 4FSY 4FSZ 4FT0 4FT2 4FT3 4FT4 4FT5 4FT6 4FT7 4FT8 4FT9 4FTA 4FTC 4FTD 4FTF 4FTG 4FTI 4FTJ 4FTK 4FTL 4FTM 4FTN 4FTO 4FTP 4FTQ 4FTR 4FTT 4FTU 4FTV 4FTW 4FTX 4FU0 4FU3 4FU4 4FU5 4FU6 4FU7 4FU8 4FU9 4FUA 4FUB 4FUC 4FUD 4FUE 4FUF 4FUG 4FUH 4FUI 4FUJ 4FUK 4FUL 4FUM 4FUN 4FUO 4FUP 4FUQ 4FUR 4FUS 4FUT 4FUU 4FUV 4FUW 4FUX 4FUY 4FV0 4FV1 4FV2 4FV3 4FV4 4FV5 4FV6 4FV7 4FV8 4FV9 4FVA 4FVB 4FVC 4FVD 4FVF 4FVG 4FVJ 4FVK 4FVL 4FVM 4FVN 4FVO 4FVP 4FVQ 4FVR 4FVS 4FVT 4FVV 4FVW 4FVX 4FVY 4FVZ 4FW0 4FW1 4FW2 4FW3 4FW4 4FW5 4FW6 4FW7 4FW8 4FW9 4FWB 4FWD 4FWE 4FWF 4FWG 4FWH 4FWI 4FWJ 4FWK 4FWL 4FWM 4FWN 4FWO 4FWP 4FWQ 4FWR 4FWS 4FWU 4FWV 4FWW 4FWX 4FWY 4FWZ 4FX0 4FX2 4FX3 4FX5 4FX6 4FX7 4FX8 4FX9 4FXA 4FXB 4FXC 4FXE 4FXF 4FXG 4FXH 4FXI 4FXJ 4FXK 4FXL 4FXO 4FXP 4FXQ 4FXR 4FXS 4FXT 4FXU 4FXV 4FXW 4FXX 4FXY 4FXZ 4FY0 4FYB 4FYC 4FYE 4FYF 4FYG 4FYH 4FYI 4FYJ 4FYK 4FYM 4FYN 4FYO 4FYP 4FYQ 4FYR 4FYS 4FYT 4FYU 4FYV 4FYW 4FYX 4FYY 4FYZ 4FZ0 4FZ1 4FZ2 4FZ3 4FZ4 4FZ5 4FZ6 4FZ7 4FZ8 4FZ9 4FZA 4FZB 4FZC 4FZD 4FZE 4FZF 4FZG 4FZH 4FZI 4FZJ 4FZL 4FZM 4FZN 4FZO 4FZP 4FZQ 4FZR 4FZS 4FZV 4FZW 4G01 4G03 4G04 4G05 4G06 4G07 4G08 4G09 4G0B 4G0D 4G0H 4G0I 4G0J 4G0K 4G0L 4G0M 4G0N 4G0O 4G0P 4G0Q 4G0S 4G0X 4G0Y 4G0Z 4G10 4G11 4G12 4G13 4G14 4G16 4G17 4G19 4G1A 4G1B 4G1C 4G1D 4G1E 4G1F 4G1G 4G1H 4G1I 4G1J 4G1K 4G1L 4G1M 4G1N 4G1O 4G1P 4G1Q 4G1R 4G1T 4G1U 4G1V 4G1W 4G1X 4G1Y 4G1Z 4G20 4G21 4G22 4G23 4G24 4G25 4G26 4G27 4G28 4G29 4G2A 4G2B 4G2C 4G2D 4G2E 4G2F 4G2G 4G2H 4G2I 4G2J 4G2K 4G2L 4G2M 4G2N 4G2O 4G2P 4G2R 4G2S 4G2T 4G2U 4G2V 4G2W 4G2Y 4G2Z 4G31 4G32 4G33 4G34 4G35 4G36 4G37 4G38 4G39 4G3A 4G3B 4G3C 4G3D 4G3E 4G3F 4G3G 4G3H 4G3J 4G3K 4G3M 4G3N 4G3O 4G3P 4G3Q 4G3R 4G3S 4G3T 4G3U 4G3V 4G3W 4G3X 4G3Y 4G41 4G42 4G43 4G44 4G45 4G46 4G47 4G48 4G49 4G4A 4G4B 4G4C 4G4E 4G4F 4G4G 4G4H 4G4I 4G4J 4G4K 4G4L 4G4M 4G4P 4G4S 4G4V 4G4W 4G4X 4G4Y 4G4Z 4G50 4G51 4G54 4G55 4G56 4G57 4G59 4G5A 4G5D 4G5E 4G5F 4G5G 4G5H 4G5I 4G5J 4G5O 4G5P 4G5Q 4G5R 4G5S 4G5X 4G5Y 4G5Z 4G61 4G63 4G65 4G67 4G68 4G69 4G6A 4G6B 4G6C 4G6D 4G6F 4G6G 4G6H 4G6I 4G6J 4G6K 4G6L 4G6M 4G6N 4G6O 4G6Q 4G6T 4G6U 4G6V 4G6W 4G6X 4G6Z 4G70 4G71 4G72 4G73 4G74 4G75 4G76 4G77 4G78 4G79 4G7A 4G7E 4G7F 4G7G 4G7L 4G7N 4G7P 4G7Q 4G7R 4G7S 4G7T 4G7U 4G7V 4G7W 4G7X 4G7Y 4G80 4G81 4G84 4G85 4G86 4G87 4G88 4G89 4G8A 4G8B 4G8C 4G8D 4G8E 4G8F 4G8G 4G8H 4G8I 4G8J 4G8K 4G8L 4G8M 4G8N 4G8O 4G8P 4G8R 4G8S 4G8T 4G8U 4G8V 4G8W 4G8X 4G8Y 4G8Z 4G90 4G91 4G93 4G94 4G95 4G96 4G97 4G98 4G99 4G9A 4G9B 4G9C 4G9D 4G9E 4G9F 4G9G 4G9H 4G9I 4G9J 4G9K 4G9L 4G9M 4G9N 4G9O 4G9P 4G9Q 4G9R 4G9S 4G9Y 4GA0 4GA1 4GA2 4GA3 4GA4 4GA5 4GA6 4GA7 4GA8 4GA9 4GAA 4GAB 4GAC 4GAD 4GAE 4GAF 4GAG 4GAH 4GAI 4GAJ 4GAK 4GAL 4GAM 4GAO 4GAP 4GAV 4GAW 4GAX 4GAY 4GAZ 4GB0 4GB1 4GB2 4GB3 4GB5 4GB7 4GB9 4GBA 4GBC 4GBD 4GBE 4GBF 4GBG 4GBI 4GBJ 4GBK 4GBL 4GBM 4GBN 4GBO 4GBP 4GBR 4GBS 4GBT 4GBU 4GBV 4GBW 4GBX 4GBY 4GBZ 4GC0 4GC1 4GC2 4GC3 4GC4 4GC5 4GC8 4GC9 4GCA 4GCB 4GCC 4GCD 4GCE 4GCF 4GCH 4GCI 4GCJ 4GCM 4GCN 4GCO 4GCP 4GCQ 4GCR 4GCS 4GCV 4GCX 4GCY 4GCZ 4GD0 4GD3 4GD4 4GD5 4GD6 4GD7 4GD8 4GD9 4GDA 4GDB 4GDC 4GDD 4GDE 4GDI 4GDJ 4GDK 4GDL 4GDM 4GDN 4GDO 4GDP 4GDX 4GDY 4GDZ 4GE0 4GE1 4GE2 4GE3 4GE4 4GE5 4GE6 4GE7 4GE8 4GE9 4GEB 4GEC 4GED 4GEE 4GEG 4GEH 4GEI 4GEJ 4GEK 4GEL 4GEM 4GEN 4GEO 4GEP 4GEQ 4GER 4GES 4GET 4GEU 4GEV 4GEW 4GEX 4GEY 4GEZ 4GF0 4GF1 4GF2 4GF3 4GF4 4GF5 4GF6 4GF7 4GF8 4GF9 4GFA 4GFC 4GFD 4GFG 4GFI 4GFJ 4GFK 4GFL 4GFM 4GFN 4GFO 4GFP 4GFQ 4GFR 4GFS 4GFT 4GFU 4GFV 4GFX 4GFY 4GG1 4GG2 4GG5 4GG6 4GG7 4GG8 4GG9 4GGA 4GGB 4GGC 4GGD 4GGF 4GGG 4GGH 4GGJ 4GGK 4GGL 4GGM 4GGN 4GGO 4GGP 4GGQ 4GGR 4GGT 4GGV 4GGZ 4GH0 4GH1 4GH2 4GH3 4GH4 4GH5 4GH6 4GH7 4GH8 4GH9 4GHB 4GHC 4GHD 4GHE 4GHF 4GHG 4GHH 4GHI 4GHJ 4GHK 4GHM 4GHN 4GHO 4GHP 4GHQ 4GHR 4GHS 4GHT 4GHU 4GHW 4GI0 4GI1 4GI2 4GI3 4GI4 4GI5 4GI6 4GI7 4GI8 4GI9 4GIA 4GIB 4GIC 4GID 4GIE 4GIF 4GIG 4GIH 4GII 4GIJ 4GIK 4GIL 4GIM 4GIN 4GIO 4GIP 4GIQ 4GIR 4GIS 4GIT 4GIU 4GIV 4GIW 4GIX 4GIY 4GIZ 4GJ0 4GJ1 4GJ2 4GJ3 4GJ4 4GJ5 4GJ6 4GJ7 4GJ8 4GJ9 4GJA 4GJB 4GJC 4GJD 4GJE 4GJF 4GJG 4GJH 4GJI 4GJJ 4GJQ 4GJS 4GJT 4GJV 4GJW 4GJX 4GJY 4GJZ 4GK0 4GK1 4GK2 4GK3 4GK4 4GK5 4GK6 4GK7 4GK8 4GK9 4GKA 4GKB 4GKC 4GKF 4GKG 4GKH 4GKI 4GKL 4GKM 4GKN 4GKO 4GKP 4GKQ 4GKR 4GKS 4GKT 4GKU 4GKV 4GKW 4GKX 4GKY 4GKZ 4GL0 4GL1 4GL3 4GL4 4GL5 4GL6 4GL7 4GL8 4GL9 4GLA 4GLB 4GLD 4GLF 4GLI 4GLJ 4GLK 4GLL 4GLM 4GLN 4GLO 4GLP 4GLQ 4GLR 4GLS 4GLT 4GLU 4GLV 4GLW 4GLY 4GM0 4GM1 4GM2 4GM3 4GM4 4GM5 4GM6 4GM7 4GM8 4GM9 4GMB 4GMC 4GMD 4GME 4GMF 4GMG 4GMH 4GMI 4GMJ 4GMK 4GML 4GMM 4GMN 4GMO 4GMP 4GMQ 4GMR 4GMS 4GMT 4GMU 4GMV 4GMW 4GMX 4GMY 4GMZ 4GN0 4GN1 4GN2 4GN3 4GN4 4GN5 4GN6 4GN7 4GN8 4GN9 4GNA 4GNB 4GNC 4GND 4GNE 4GNF 4GNG 4GNI 4GNJ 4GNK 4GNL 4GNM 4GNO 4GNP 4GNQ 4GNR 4GNT 4GNU 4GNV 4GNW 4GNY 4GNZ 4GO0 4GO1 4GO2 4GO3 4GO4 4GO5 4GO6 4GO7 4GO8 4GO9 4GOA 4GOB 4GOC 4GOD 4GOE 4GOF 4GOG 4GOH 4GOJ 4GOK 4GOL 4GOM 4GON 4GOO 4GOQ 4GOS 4GOT 4GOU 4GOV 4GOW 4GOX 4GOY 4GP0 4GP1 4GP2 4GP3 4GP4 4GP5 4GP6 4GP7 4GP8 4GP9 4GPA 4GPB 4GPC 4GPD 4GPE 4GPF 4GPH 4GPI 4GPJ 4GPK 4GPL 4GPM 4GPN 4GPO 4GPQ 4GPR 4GPS 4GPT 4GPU 4GPV 4GPZ 4GQ0 4GQ1 4GQ2 4GQ3 4GQ4 4GQ6 4GQ7 4GQ9 4GQA 4GQB 4GQC 4GQE 4GQF 4GQG 4GQH 4GQI 4GQK 4GQL 4GQM 4GQN 4GQO 4GQP 4GQQ 4GQR 4GQS 4GQT 4GQU 4GQV 4GQW 4GQX 4GQY 4GQZ 4GR0 4GR1 4GR2 4GR3 4GR4 4GR5 4GR6 4GR7 4GR8 4GR9 4GRA 4GRB 4GRC 4GRD 4GRF 4GRG 4GRH 4GRI 4GRJ 4GRK 4GRL 4GRM 4GRN 4GRO 4GRP 4GRQ 4GRR 4GRS 4GRT 4GRU 4GRV 4GRW 4GRX 4GRY 4GRZ 4GS0 4GS1 4GS3 4GS4 4GS5 4GS6 4GS7 4GS8 4GS9 4GSA 4GSB 4GSC 4GSD 4GSF 4GSJ 4GSK 4GSL 4GSM 4GSN 4GSO 4GSP 4GSQ 4GSR 4GSS 4GST 4GSU 4GSV 4GSW 4GSX 4GSY 4GSZ 4GT0 4GT1 4GT2 4GT3 4GT4 4GT5 4GT6 4GT7 4GT8 4GT9 4GTA 4GTB 4GTC 4GTD 4GTE 4GTF 4GTL 4GTM 4GTN 4GTO 4GTP 4GTQ 4GTR 4GTS 4GTT 4GTU 4GTV 4GTW 4GTX 4GTY 4GTZ 4GU0 4GU1 4GU2 4GU3 4GU4 4GU5 4GU6 4GU7 4GU8 4GU9 4GUA 4GUB 4GUC 4GUD 4GUE 4GUF 4GUG 4GUH 4GUI 4GUJ 4GUK 4GUL 4GUM 4GUN 4GUP 4GUR 4GUS 4GUT 4GUU 4GUV 4GUW 4GUX 4GUY 4GUZ 4GV0 4GV1 4GV2 4GV4 4GV5 4GV7 4GV8 4GVA 4GVB 4GVC 4GVD 4GVE 4GVF 4GVG 4GVH 4GVI 4GVJ 4GVL 4GVM 4GVO 4GVP 4GVQ 4GVR 4GVS 4GVT 4GVU 4GVV 4GVW 4GVX 4GVY 4GVZ 4GW0 4GW1 4GW2 4GW3 4GW4 4GW5 4GW6 4GW8 4GW9 4GWA 4GWB 4GWC 4GWD 4GWE 4GWF 4GWG 4GWI 4GWJ 4GWK 4GWL 4GWM 4GWN 4GWO 4GWP 4GWQ 4GWR 4GWS 4GWT 4GWU 4GWV 4GWW 4GWX 4GWY 4GWZ 4GX0 4GX1 4GX2 4GX3 4GX4 4GX5 4GX6 4GX7 4GX8 4GX9 4GXA 4GXB 4GXD 4GXE 4GXF 4GXG 4GXH 4GXL 4GXM 4GXN 4GXO 4GXP 4GXQ 4GXR 4GXS 4GXT 4GXU 4GXV 4GXW 4GXX 4GXZ 4GY0 4GY1 4GY2 4GY3 4GY4 4GY5 4GY7 4GY9 4GYB 4GYC 4GYD 4GYE 4GYF 4GYG 4GYH 4GYI 4GYJ 4GYK 4GYL 4GYM 4GYN 4GYO 4GYP 4GYQ 4GYR 4GYS 4GYT 4GYU 4GYV 4GYW 4GYX 4GYY 4GYZ 4GZ3 4GZ5 4GZ6 4GZ7 4GZ8 4GZ9 4GZA 4GZB 4GZC 4GZD 4GZE 4GZF 4GZG 4GZI 4GZJ 4GZK 4GZL 4GZM 4GZO 4GZP 4GZQ 4GZR 4GZS 4GZT 4GZU 4GZV 4GZW 4GZX 4H00 4H01 4H02 4H03 4H04 4H05 4H07 4H08 4H09 4H0A 4H0B 4H0C 4H0D 4H0F 4H0G 4H0H 4H0I 4H0J 4H0K 4H0L 4H0M 4H0N 4H0O 4H0P 4H0R 4H0S 4H0T 4H0U 4H0V 4H0W 4H0X 4H0Y 4H0Z 4H11 4H12 4H13 4H14 4H15 4H16 4H17 4H18 4H19 4H1A 4H1B 4H1D 4H1E 4H1G 4H1H 4H1I 4H1J 4H1L 4H1M 4H1N 4H1O 4H1P 4H1Q 4H1S 4H1T 4H1U 4H1V 4H1W 4H1X 4H1Y 4H1Z 4H20 4H22 4H23 4H24 4H25 4H26 4H27 4H2A 4H2B 4H2C 4H2D 4H2E 4H2F 4H2G 4H2H 4H2I 4H2J 4H2K 4H2L 4H2M 4H2N 4H2O 4H2P 4H2Q 4H2R 4H2S 4H2T 4H2U 4H2V 4H2W 4H2X 4H2Y 4H2Z 4H30 4H31 4H32 4H33 4H34 4H35 4H36 4H37 4H38 4H39 4H3A 4H3B 4H3C 4H3D 4H3E 4H3F 4H3G 4H3H 4H3I 4H3J 4H3K 4H3L 4H3M 4H3N 4H3O 4H3P 4H3Q 4H3S 4H3T 4H3U 4H3V 4H3W 4H3X 4H3Y 4H3Z 4H40 4H41 4H42 4H43 4H44 4H45 4H46 4H47 4H48 4H49 4H4A 4H4B 4H4C 4H4D 4H4E 4H4F 4H4G 4H4I 4H4J 4H4K 4H4L 4H4M 4H4N 4H4O 4H4P 4H4Q 4H4R 4H4S 4H4T 4H4U 4H4V 4H4W 4H4X 4H4Y 4H4Z 4H50 4H51 4H52 4H53 4H54 4H55 4H56 4H57 4H58 4H59 4H5B 4H5C 4H5D 4H5E 4H5F 4H5G 4H5I 4H5J 4H5L 4H5M 4H5N 4H5R 4H5S 4H5T 4H5U 4H5V 4H5W 4H5X 4H5Y 4H60 4H61 4H62 4H63 4H65 4H67 4H69 4H6A 4H6B 4H6C 4H6D 4H6E 4H6H 4H6I 4H6J 4H6K 4H6O 4H6P 4H6Q 4H6R 4H6S 4H6T 4H6U 4H6V 4H6W 4H6X 4H6Y 4H6Z 4H71 4H73 4H75 4H76 4H77 4H79 4H7A 4H7B 4H7C 4H7D 4H7E 4H7F 4H7H 4H7I 4H7J 4H7K 4H7L 4H7M 4H7N 4H7O 4H7P 4H7Q 4H7R 4H7U 4H7V 4H7W 4H7X 4H7Y 4H7Z 4H80 4H81 4H82 4H83 4H84 4H85 4H86 4H87 4H88 4H89 4H8A 4H8E 4H8F 4H8G 4H8H 4H8I 4H8J 4H8L 4H8M 4H8N 4H8O 4H8P 4H8Q 4H8R 4H8S 4H8U 4H8V 4H8W 4H8X 4H8Y 4H8Z 4H90 4H91 4H92 4H93 4H94 4H95 4H96 4H97 4H98 4H99 4H9A 4H9B 4H9C 4H9D 4H9E 4H9F 4H9G 4H9H 4H9I 4H9J 4H9K 4H9L 4H9M 4H9N 4H9O 4H9P 4H9Q 4H9R 4H9S 4H9T 4H9U 4H9V 4H9W 4H9X 4H9Y 4H9Z 4HA0 4HA1 4HA2 4HA3 4HA4 4HA5 4HA6 4HA7 4HA8 4HA9 4HAA 4HAB 4HAC 4HAD 4HAE 4HAF 4HAG 4HAH 4HAI 4HAJ 4HAK 4HAL 4HAM 4HAN 4HAO 4HAP 4HAQ 4HAR 4HAS 4HAT 4HAU 4HAV 4HAW 4HAX 4HAY 4HAZ 4HB0 4HB1 4HB2 4HB3 4HB4 4HB5 4HB6 4HB7 4HB8 4HB9 4HBA 4HBC 4HBD 4HBE 4HBF 4HBG 4HBH 4HBI 4HBJ 4HBK 4HBL 4HBM 4HBN 4HBO 4HBP 4HBQ 4HBR 4HBS 4HBT 4HBU 4HBV 4HBW 4HBX 4HBY 4HBZ 4HC1 4HC3 4HC4 4HC5 4HC6 4HC8 4HCD 4HCE 4HCF 4HCG 4HCH 4HCI 4HCJ 4HCL 4HCN 4HCO 4HCP 4HCQ 4HCR 4HCS 4HCT 4HCU 4HCV 4HCW 4HCX 4HCY 4HCZ 4HD0 4HD1 4HD4 4HD5 4HD6 4HD7 4HD8 4HD9 4HDA 4HDB 4HDC 4HDD 4HDE 4HDF 4HDG 4HDH 4HDI 4HDJ 4HDK 4HDL 4HDM 4HDN 4HDO 4HDP 4HDQ 4HDR 4HDS 4HDT 4HE0 4HE1 4HE2 4HE4 4HE5 4HE6 4HE7 4HE8 4HE9 4HEA 4HEB 4HEC 4HED 4HEE 4HEF 4HEG 4HEH 4HEI 4HEJ 4HEL 4HEM 4HEO 4HEP 4HEQ 4HES 4HET 4HEU 4HEV 4HEW 4HEX 4HEY 4HEZ 4HF0 4HF3 4HF4 4HF5 4HF7 4HF8 4HFB 4HFC 4HFD 4HFE 4HFF 4HFG 4HFH 4HFI 4HFJ 4HFK 4HFL 4HFM 4HFN 4HFO 4HFP 4HFQ 4HFR 4HFS 4HFU 4HFV 4HFW 4HFX 4HFZ 4HG0 4HG2 4HG3 4HG4 4HG5 4HG6 4HG7 4HG9 4HGA 4HGC 4HGD 4HGE 4HGF 4HGG 4HGH 4HGI 4HGJ 4HGK 4HGL 4HGM 4HGN 4HGO 4HGP 4HGQ 4HGR 4HGS 4HGT 4HGU 4HGV 4HGW 4HGX 4HGY 4HGZ 4HH0 4HH1 4HH2 4HH3 4HH4 4HH5 4HH6 4HH8 4HH9 4HHA 4HHB 4HHD 4HHE 4HHF 4HHG 4HHH 4HHJ 4HHL 4HHM 4HHO 4HHP 4HHQ 4HHR 4HHS 4HHU 4HHV 4HHW 4HHX 4HHY 4HHZ 4HI0 4HI1 4HI2 4HI3 4HI4 4HI5 4HI6 4HI7 4HI8 4HI9 4HIA 4HIB 4HIC 4HIE 4HIH 4HII 4HIJ 4HIL 4HIN 4HIP 4HIQ 4HIS 4HIT 4HIU 4HIW 4HIX 4HIY 4HIZ 4HJ0 4HJ1 4HJ2 4HJ3 4HJ4 4HJ6 4HJB 4HJC 4HJD 4HJF 4HJG 4HJH 4HJI 4HJJ 4HJK 4HJL 4HJM 4HJO 4HJP 4HJQ 4HJR 4HJS 4HJT 4HJU 4HJV 4HJW 4HJX 4HJY 4HJZ 4HK0 4HK1 4HK2 4HK3 4HK4 4HK5 4HK6 4HK7 4HK8 4HK9 4HKA 4HKB 4HKC 4HKD 4HKE 4HKF 4HKG 4HKH 4HKI 4HKJ 4HKK 4HKL 4HKM 4HKN 4HKO 4HKP 4HKR 4HKS 4HKT 4HKU 4HKV 4HKW 4HKX 4HKY 4HKZ 4HL0 4HL1 4HL2 4HL4 4HL5 4HL6 4HL7 4HL8 4HL9 4HLA 4HLB 4HLC 4HLD 4HLE 4HLF 4HLG 4HLH 4HLJ 4HLK 4HLL 4HLM 4HLN 4HLQ 4HLR 4HLS 4HLT 4HLU 4HLW 4HLX 4HLZ 4HM0 4HM1 4HM2 4HM3 4HM4 4HM5 4HM6 4HM7 4HM8 4HM9 4HMA 4HMB 4HMC 4HMD 4HME 4HMG 4HMH 4HMI 4HMJ 4HMK 4HMM 4HMN 4HMO 4HMP 4HMQ 4HMR 4HMS 4HMT 4HMU 4HMV 4HMW 4HMX 4HMY 4HMZ 4HN0 4HN1 4HN2 4HN3 4HN4 4HN7 4HN8 4HN9 4HNA 4HNB 4HNC 4HND 4HNE 4HNF 4HNG 4HNH 4HNI 4HNJ 4HNK 4HNL 4HNM 4HNN 4HNO 4HNP 4HNQ 4HNR 4HNS 4HNT 4HNU 4HNV 4HNW 4HNX 4HNY 4HNZ 4HO0 4HO1 4HO2 4HO3 4HO4 4HO5 4HO6 4HO7 4HO8 4HO9 4HOA 4HOB 4HOC 4HOD 4HOE 4HOF 4HOG 4HOH 4HOI 4HOJ 4HOK 4HOM 4HON 4HOO 4HOP 4HOQ 4HOU 4HOV 4HOW 4HOX 4HOY 4HOZ 4HP0 4HP2 4HP4 4HP5 4HP8 4HP9 4HPA 4HPB 4HPC 4HPD 4HPE 4HPF 4HPG 4HPH 4HPI 4HPJ 4HPK 4HPL 4HPM 4HPN 4HPO 4HPP 4HPQ 4HPS 4HPT 4HPU 4HPV 4HPW 4HPX 4HPY 4HPZ 4HQ0 4HQ1 4HQ6 4HQ8 4HQ9 4HQA 4HQC 4HQD 4HQF 4HQJ 4HQK 4HQL 4HQM 4HQN 4HQO 4HQP 4HQQ 4HQR 4HQS 4HQV 4HQW 4HQZ 4HR0 4HR1 4HR2 4HR3 4HR4 4HR5 4HR6 4HR7 4HR9 4HRA 4HRC 4HRD 4HRE 4HRF 4HRG 4HRH 4HRL 4HRM 4HRN 4HRO 4HRQ 4HRR 4HRS 4HRT 4HRU 4HRV 4HRW 4HRX 4HRY 4HRZ 4HS1 4HS2 4HS3 4HS4 4HS5 4HS6 4HS7 4HS8 4HS9 4HSA 4HSC 4HSD 4HSE 4HSF 4HSG 4HSH 4HSI 4HSJ 4HSL 4HSN 4HSO 4HSP 4HSQ 4HSR 4HSS 4HST 4HSU 4HSV 4HSW 4HSX 4HSZ 4HT0 4HT1 4HT2 4HT3 4HT5 4HT6 4HT7 4HTA 4HTB 4HTC 4HTE 4HTF 4HTG 4HTH 4HTI 4HTJ 4HTK 4HTL 4HTM 4HTN 4HTO 4HTP 4HTQ 4HTR 4HTS 4HTT 4HTV 4HTW 4HTX 4HTY 4HTZ 4HU0 4HU1 4HU2 4HU3 4HU4 4HU5 4HU6 4HU7 4HU8 4HU9 4HUA 4HUC 4HUD 4HUH 4HUJ 4HUK 4HUL 4HUM 4HUN 4HUO 4HUP 4HUQ 4HUR 4HUS 4HUT 4HUU 4HUV 4HUW 4HUX 4HUZ 4HV0 4HV1 4HV2 4HV3 4HV4 4HV5 4HV6 4HV7 4HV8 4HVA 4HVB 4HVC 4HVD 4HVF 4HVG 4HVH 4HVI 4HVJ 4HVK 4HVL 4HVM 4HVN 4HVO 4HVP 4HVQ 4HVR 4HVS 4HVT 4HVU 4HVV 4HVW 4HVX 4HVY 4HVZ 4HW0 4HW2 4HW3 4HW4 4HW5 4HW6 4HW7 4HW8 4HW9 4HWA 4HWB 4HWC 4HWD 4HWE 4HWF 4HWG 4HWH 4HWI 4HWJ 4HWK 4HWL 4HWM 4HWN 4HWO 4HWP 4HWR 4HWS 4HWT 4HWU 4HWV 4HWW 4HWX 4HWY 4HWZ 4HX0 4HX1 4HX2 4HX3 4HX4 4HX5 4HX6 4HX7 4HX8 4HX9 4HXA 4HXB 4HXC 4HXD 4HXE 4HXF 4HXG 4HXI 4HXJ 4HXK 4HXL 4HXM 4HXN 4HXO 4HXP 4HXQ 4HXR 4HXS 4HXT 4HXV 4HXW 4HXX 4HXY 4HXZ 4HY0 4HY1 4HY2 4HY3 4HY4 4HY5 4HY6 4HY7 4HY8 4HY9 4HYB 4HYC 4HYD 4HYE 4HYF 4HYG 4HYH 4HYI 4HYJ 4HYL 4HYM 4HYN 4HYO 4HYP 4HYQ 4HYR 4HYS 4HYT 4HYU 4HYV 4HYW 4HYX 4HYY 4HYZ 4HZ0 4HZ1 4HZ2 4HZ3 4HZ4 4HZ5 4HZ6 4HZ7 4HZ8 4HZ9 4HZA 4HZB 4HZC 4HZD 4HZE 4HZF 4HZG 4HZH 4HZI 4HZK 4HZL 4HZM 4HZN 4HZO 4HZP 4HZR 4HZS 4HZT 4HZU 4HZV 4HZW 4HZX 4HZY 4HZZ 4I00 4I01 4I02 4I03 4I04 4I05 4I06 4I07 4I08 4I09 4I0A 4I0B 4I0C 4I0D 4I0E 4I0F 4I0G 4I0H 4I0I 4I0J 4I0K 4I0N 4I0O 4I0P 4I0R 4I0S 4I0T 4I0U 4I0W 4I0X 4I0Y 4I0Z 4I10 4I11 4I12 4I13 4I14 4I15 4I16 4I17 4I18 4I19 4I1A 4I1B 4I1C 4I1D 4I1E 4I1F 4I1H 4I1I 4I1K 4I1L 4I1M 4I1N 4I1O 4I1P 4I1Q 4I1R 4I1S 4I1T 4I1U 4I1V 4I1W 4I1Y 4I1Z 4I20 4I21 4I22 4I23 4I24 4I25 4I26 4I2D 4I2I 4I2J 4I2L 4I2N 4I2P 4I2Q 4I2R 4I2S 4I2T 4I2U 4I2V 4I2W 4I2X 4I2Y 4I2Z 4I30 4I31 4I32 4I33 4I34 4I35 4I36 4I37 4I38 4I39 4I3A 4I3B 4I3C 4I3D 4I3E 4I3F 4I3G 4I3I 4I3J 4I3K 4I3L 4I3M 4I3N 4I3P 4I3Q 4I3R 4I3S 4I3T 4I3U 4I3V 4I3W 4I3X 4I3Y 4I3Z 4I40 4I41 4I42 4I43 4I44 4I45 4I46 4I47 4I48 4I49 4I4B 4I4C 4I4E 4I4F 4I4G 4I4H 4I4I 4I4J 4I4K 4I4L 4I4N 4I4O 4I4P 4I4Q 4I4R 4I4S 4I4T 4I4U 4I4V 4I4W 4I4X 4I4Y 4I4Z 4I50 4I51 4I52 4I53 4I54 4I55 4I56 4I58 4I59 4I5B 4I5C 4I5D 4I5E 4I5F 4I5G 4I5H 4I5I 4I5J 4I5K 4I5L 4I5M 4I5N 4I5O 4I5P 4I5Q 4I5R 4I5S 4I5T 4I5U 4I5V 4I5W 4I5X 4I5Y 4I5Z 4I60 4I61 4I62 4I63 4I64 4I65 4I66 4I68 4I69 4I6A 4I6B 4I6E 4I6F 4I6G 4I6H 4I6I 4I6J 4I6K 4I6L 4I6M 4I6N 4I6O 4I6P 4I6Q 4I6R 4I6S 4I6T 4I6U 4I6V 4I6W 4I6X 4I6Y 4I70 4I71 4I72 4I73 4I74 4I75 4I76 4I77 4I78 4I79 4I7A 4I7B 4I7C 4I7D 4I7E 4I7F 4I7H 4I7I 4I7J 4I7K 4I7L 4I7M 4I7N 4I7O 4I7P 4I7Q 4I7R 4I7S 4I7T 4I7U 4I7V 4I7W 4I7Z 4I80 4I81 4I82 4I83 4I84 4I85 4I86 4I87 4I88 4I89 4I8A 4I8B 4I8C 4I8D 4I8E 4I8G 4I8H 4I8I 4I8J 4I8K 4I8L 4I8M 4I8N 4I8O 4I8P 4I8Q 4I8S 4I8V 4I8W 4I8X 4I8Y 4I8Z 4I90 4I91 4I92 4I93 4I94 4I95 4I96 4I97 4I98 4I99 4I9A 4I9B 4I9C 4I9D 4I9E 4I9F 4I9G 4I9H 4I9I 4I9J 4I9K 4I9L 4I9M 4I9N 4I9O 4I9R 4I9S 4I9T 4I9U 4I9W 4I9X 4I9Y 4I9Z 4IA0 4IA1 4IA2 4IA3 4IA4 4IA5 4IA6 4IA7 4IA8 4IA9 4IAA 4IAB 4IAC 4IAD 4IAE 4IAF 4IAG 4IAH 4IAI 4IAJ 4IAK 4IAL 4IAM 4IAN 4IAO 4IAP 4IAQ 4IAR 4IAS 4IAT 4IAU 4IAV 4IAW 4IAX 4IAY 4IAZ 4IB0 4IB1 4IB2 4IB3 4IB4 4IB5 4IB6 4IB7 4IB8 4IB9 4IBA 4IBB 4IBC 4IBD 4IBE 4IBF 4IBG 4IBI 4IBJ 4IBK 4IBL 4IBM 4IBN 4IBO 4IBP 4IBQ 4IBR 4IBS 4IBT 4IBX 4IBY 4IBZ 4IC0 4IC1 4IC2 4IC3 4IC4 4IC5 4IC6 4IC7 4IC8 4IC9 4ICA 4ICB 4ICC 4ICD 4ICG 4ICH 4ICI 4ICK 4ICL 4ICM 4ICN 4ICQ 4ICR 4ICS 4ICT 4ICU 4ICV 4ICW 4ICX 4ICY 4ICZ 4ID0 4ID1 4ID2 4ID3 4ID4 4ID5 4ID6 4ID7 4ID8 4ID9 4IDA 4IDB 4IDC 4IDD 4IDE 4IDF 4IDG 4IDH 4IDI 4IDJ 4IDK 4IDL 4IDM 4IDN 4IDO 4IDP 4IDQ 4IDR 4IDS 4IDT 4IDU 4IDV 4IDX 4IDY 4IDZ 4IE0 4IE1 4IE2 4IE3 4IE4 4IE5 4IE6 4IE7 4IE9 4IEA 4IEB 4IEC 4IED 4IEE 4IEF 4IEG 4IEH 4IEI 4IEJ 4IEL 4IEN 4IEO 4IEP 4IEQ 4IER 4IES 4IET 4IEU 4IEV 4IEW 4IEX 4IEY 4IEZ 4IF2 4IF4 4IF5 4IF6 4IF7 4IF8 4IFA 4IFB 4IFC 4IFE 4IFF 4IFG 4IFH 4IFI 4IFJ 4IFK 4IFL 4IFN 4IFO 4IFP 4IFQ 4IFR 4IFS 4IFT 4IFU 4IFV 4IFW 4IFX 4IFY 4IFZ 4IG0 4IG1 4IG2 4IG3 4IG4 4IG5 4IG6 4IG7 4IG9 4IGA 4IGB 4IGD 4IGE 4IGF 4IGG 4IGH 4IGI 4IGJ 4IGK 4IGL 4IGM 4IGN 4IGO 4IGP 4IGQ 4IGR 4IGS 4IGT 4IGU 4IGV 4IGW 4IGX 4IGY 4IGZ 4IH0 4IH1 4IH2 4IH3 4IH4 4IH5 4IH6 4IH7 4IH8 4IH9 4IHA 4IHB 4IHC 4IHD 4IHE 4IHF 4IHG 4IHH 4IHI 4IHJ 4IHK 4IHL 4IHM 4IHN 4IHO 4IHP 4IHQ 4IHR 4IHU 4IHZ 4II0 4II1 4II2 4II3 4II4 4II5 4II7 4II8 4IIA 4IIB 4IIC 4IID 4IIE 4IIF 4IIG 4IIH 4IIJ 4IIK 4IIL 4IIM 4IIN 4IIO 4IIP 4IIQ 4IIR 4IIS 4IIT 4IIU 4IIV 4IIW 4IIX 4IIY 4IIZ 4IJ1 4IJ2 4IJ3 4IJ4 4IJ5 4IJ6 4IJ7 4IJ8 4IJ9 4IJA 4IJB 4IJC 4IJD 4IJE 4IJF 4IJG 4IJH 4IJI 4IJJ 4IJK 4IJL 4IJM 4IJN 4IJO 4IJP 4IJQ 4IJR 4IJT 4IJU 4IJV 4IJW 4IJX 4IJY 4IJZ 4IK0 4IK1 4IK2 4IK3 4IK4 4IK5 4IK6 4IK7 4IK8 4IK9 4IKA 4IKB 4IKC 4IKD 4IKE 4IKG 4IKH 4IKI 4IKJ 4IKK 4IKL 4IKM 4IKN 4IKO 4IKP 4IKR 4IKS 4IKT 4IKU 4IKV 4IKW 4IKX 4IKY 4IKZ 4IL0 4IL1 4IL2 4IL3 4IL4 4IL5 4IL6 4IL7 4IL8 4IL9 4ILA 4ILB 4ILC 4ILD 4ILE 4ILF 4ILG 4ILH 4ILI 4ILJ 4ILK 4ILO 4ILQ 4ILR 4ILS 4ILT 4ILU 4ILV 4ILW 4ILX 4ILY 4ILZ 4IM0 4IM2 4IM3 4IM4 4IM6 4IM7 4IM8 4IM9 4IMA 4IMB 4IMC 4IMD 4IME 4IMF 4IMG 4IMH 4IMI 4IMJ 4IMK 4IML 4IMM 4IMN 4IMO 4IMP 4IMQ 4IMR 4IMS 4IMT 4IMU 4IMV 4IMW 4IMX 4IMY 4IMZ 4IN0 4IN1 4IN2 4IN3 4IN4 4IN5 4IN6 4IN7 4IN9 4INA 4INB 4INC 4IND 4INE 4INF 4INH 4INI 4INJ 4INK 4INL 4INN 4INO 4INP 4INQ 4INR 4INS 4INT 4INU 4INW 4INX 4INZ 4IO0 4IO1 4IO2 4IO3 4IO4 4IO5 4IO6 4IO7 4IO8 4IOB 4IOD 4IOF 4IOH 4IOI 4IOJ 4IOK 4IOL 4IOM 4ION 4IOO 4IOP 4IOQ 4IOR 4IOS 4IOT 4IOU 4IOV 4IOX 4IOY 4IP0 4IP1 4IP2 4IP3 4IP4 4IP5 4IP6 4IP7 4IP8 4IP9 4IPA 4IPB 4IPC 4IPD 4IPE 4IPF 4IPG 4IPH 4IPI 4IPJ 4IPL 4IPM 4IPN 4IPP 4IPS 4IPT 4IPU 4IPV 4IPW 4IPX 4IPY 4IPZ 4IQ0 4IQ1 4IQ2 4IQ4 4IQ6 4IQ7 4IQ8 4IQ9 4IQA 4IQB 4IQC 4IQD 4IQE 4IQF 4IQG 4IQH 4IQI 4IQK 4IQL 4IQM 4IQN 4IQP 4IQQ 4IQT 4IQU 4IQY 4IQZ 4IR0 4IR3 4IR4 4IR5 4IR6 4IR7 4IR8 4IRA 4IRB 4IRE 4IRF 4IRG 4IRH 4IRJ 4IRL 4IRM 4IRN 4IRO 4IRP 4IRQ 4IRR 4IRS 4IRT 4IRU 4IRV 4IRW 4IRX 4IRY 4IRZ 4IS0 4IS2 4IS3 4IS4 4IS5 4IS6 4IS7 4IS8 4IS9 4ISA 4ISB 4ISC 4ISD 4ISE 4ISF 4ISG 4ISH 4ISI 4ISJ 4ISK 4ISL 4ISM 4ISN 4ISO 4ISP 4ISQ 4ISR 4ISS 4IST 4ISU 4ISV 4ISW 4ISX 4ISY 4ISZ 4IT0 4IT1 4IT2 4IT3 4IT4 4IT5 4IT6 4IT7 4IT8 4IT9 4ITA 4ITB 4ITC 4ITE 4ITF 4ITG 4ITH 4ITI 4ITJ 4ITK 4ITL 4ITM 4ITN 4ITO 4ITP 4ITR 4ITS 4ITT 4ITU 4ITV 4ITW 4ITX 4ITY 4ITZ 4IU0 4IU1 4IU2 4IU3 4IU4 4IU5 4IU6 4IU7 4IU8 4IU9 4IUA 4IUB 4IUC 4IUD 4IUE 4IUG 4IUH 4IUI 4IUJ 4IUK 4IUL 4IUM 4IUN 4IUO 4IUP 4IUQ 4IUR 4IUS 4IUT 4IUU 4IUV 4IUW 4IUY 4IUZ 4IV0 4IV1 4IV2 4IV3 4IV4 4IV5 4IV6 4IV8 4IV9 4IVA 4IVB 4IVC 4IVD 4IVE 4IVF 4IVG 4IVH 4IVI 4IVJ 4IVK 4IVM 4IVN 4IVO 4IVP 4IVQ 4IVR 4IVS 4IVT 4IVV 4IVW 4IVY 4IW0 4IW1 4IW2 4IW3 4IW4 4IW6 4IW7 4IW8 4IW9 4IWB 4IWC 4IWD 4IWF 4IWG 4IWH 4IWJ 4IWK 4IWM 4IWN 4IWO 4IWP 4IWQ 4IWS 4IWT 4IWV 4IWW 4IWX 4IWY 4IWZ 4IX0 4IX1 4IX2 4IX3 4IX4 4IX5 4IX6 4IX8 4IX9 4IXA 4IXC 4IXD 4IXE 4IXF 4IXG 4IXH 4IXJ 4IXK 4IXL 4IXM 4IXN 4IXO 4IXP 4IXQ 4IXR 4IXS 4IXT 4IXU 4IXV 4IXW 4IXX 4IXZ 4IY0 4IY1 4IY2 4IY3 4IY4 4IY5 4IY6 4IY7 4IY8 4IY9 4IYA 4IYB 4IYC 4IYD 4IYE 4IYF 4IYG 4IYH 4IYI 4IYJ 4IYK 4IYL 4IYM 4IYN 4IYO 4IYP 4IYQ 4IYR 4IYS 4IYT 4IZ0 4IZ5 4IZ6 4IZ7 4IZ8 4IZ9 4IZA 4IZB 4IZC 4IZD 4IZE 4IZG 4IZH 4IZI 4IZJ 4IZK 4IZL 4IZM 4IZN 4IZO 4IZS 4IZT 4IZU 4IZV 4IZW 4IZX 4IZY 4J02 4J03 4J04 4J05 4J06 4J07 4J08 4J09 4J0A 4J0B 4J0C 4J0D 4J0E 4J0F 4J0G 4J0H 4J0I 4J0J 4J0K 4J0L 4J0M 4J0N 4J0O 4J0P 4J0Q 4J0R 4J0S 4J0T 4J0U 4J0V 4J0W 4J0X 4J0Y 4J0Z 4J10 4J11 4J12 4J14 4J15 4J16 4J17 4J18 4J1A 4J1B 4J1C 4J1E 4J1F 4J1H 4J1I 4J1K 4J1L 4J1M 4J1N 4J1O 4J1P 4J1Q 4J1R 4J1S 4J1T 4J1U 4J1V 4J1W 4J1X 4J1Y 4J1Z 4J20 4J21 4J22 4J23 4J24 4J25 4J26 4J27 4J28 4J29 4J2C 4J2F 4J2G 4J2H 4J2J 4J2K 4J2L 4J2M 4J2N 4J2O 4J2P 4J2Q 4J2R 4J2S 4J2T 4J2U 4J2V 4J2W 4J2Y 4J30 4J31 4J32 4J33 4J34 4J35 4J36 4J37 4J38 4J3B 4J3C 4J3D 4J3E 4J3F 4J3G 4J3H 4J3I 4J3J 4J3K 4J3L 4J3M 4J3O 4J3P 4J3Q 4J3R 4J3S 4J3T 4J3U 4J3V 4J3W 4J3X 4J3Y 4J3Z 4J40 4J41 4J42 4J43 4J44 4J45 4J46 4J47 4J48 4J49 4J4A 4J4B 4J4C 4J4D 4J4E 4J4F 4J4G 4J4H 4J4J 4J4K 4J4L 4J4M 4J4N 4J4O 4J4P 4J4Q 4J4R 4J4S 4J4T 4J4U 4J4V 4J4W 4J4X 4J4Y 4J4Z 4J51 4J52 4J53 4J54 4J55 4J56 4J57 4J58 4J59 4J5A 4J5B 4J5C 4J5D 4J5E 4J5F 4J5G 4J5H 4J5I 4J5J 4J5K 4J5L 4J5M 4J5N 4J5O 4J5P 4J5Q 4J5R 4J5S 4J5T 4J5U 4J5W 4J5X 4J5Y 4J5Z 4J60 4J61 4J62 4J63 4J64 4J65 4J66 4J67 4J68 4J69 4J6A 4J6B 4J6C 4J6D 4J6E 4J6F 4J6G 4J6H 4J6I 4J6J 4J6K 4J6L 4J6M 4J6N 4J6O 4J6P 4J6Q 4J6R 4J6S 4J6T 4J6U 4J6V 4J6W 4J6X 4J6Y 4J70 4J71 4J72 4J73 4J74 4J75 4J76 4J77 4J78 4J79 4J7A 4J7B 4J7C 4J7D 4J7E 4J7F 4J7G 4J7H 4J7I 4J7J 4J7K 4J7N 4J7O 4J7P 4J7Q 4J7R 4J7T 4J7U 4J7V 4J7W 4J7X 4J7Y 4J81 4J82 4J83 4J84 4J86 4J87 4J88 4J89 4J8A 4J8B 4J8C 4J8D 4J8E 4J8F 4J8G 4J8L 4J8M 4J8N 4J8O 4J8P 4J8Q 4J8R 4J8S 4J8T 4J8Y 4J8Z 4J90 4J91 4J93 4J94 4J95 4J96 4J97 4J98 4J99 4J9A 4J9B 4J9C 4J9D 4J9E 4J9F 4J9G 4J9H 4J9I 4J9J 4J9T 4J9U 4J9V 4J9W 4J9X 4J9Y 4J9Z 4JA0 4JA1 4JA2 4JA3 4JA4 4JA7 4JA8 4JA9 4JAA 4JAC 4JAD 4JAE 4JAF 4JAG 4JAI 4JAJ 4JAK 4JAL 4JAM 4JAN 4JAO 4JAP 4JAQ 4JAR 4JAS 4JAT 4JAU 4JAV 4JAW 4JAX 4JAY 4JAZ 4JB0 4JB1 4JB2 4JB3 4JB4 4JB6 4JB7 4JB8 4JB9 4JBA 4JBB 4JBC 4JBD 4JBE 4JBF 4JBG 4JBH 4JBI 4JBJ 4JBL 4JBN 4JBO 4JBP 4JBQ 4JBR 4JBS 4JBT 4JBU 4JBV 4JBW 4JBX 4JBY 4JBZ 4JC0 4JC1 4JC2 4JC3 4JC4 4JC5 4JC6 4JC7 4JC8 4JCA 4JCC 4JCD 4JCE 4JCF 4JCG 4JCH 4JCI 4JCJ 4JCK 4JCL 4JCM 4JCN 4JCO 4JCP 4JCQ 4JCR 4JCS 4JCT 4JCU 4JCV 4JCW 4JCZ 4JD0 4JD1 4JD2 4JD3 4JD4 4JD5 4JD6 4JD7 4JD9 4JDA 4JDB 4JDC 4JDD 4JDE 4JDF 4JDG 4JDH 4JDI 4JDJ 4JDK 4JDL 4JDM 4JDN 4JDO 4JDP 4JDQ 4JDR 4JDS 4JDT 4JDU 4JDV 4JDW 4JDX 4JDY 4JDZ 4JE0 4JE1 4JE3 4JE4 4JE5 4JE6 4JE7 4JE8 4JE9 4JEA 4JEB 4JED 4JEF 4JEG 4JEH 4JEI 4JEJ 4JEK 4JEL 4JEM 4JEN 4JEO 4JEP 4JEQ 4JER 4JES 4JET 4JEU 4JEV 4JEW 4JEX 4JEY 4JEZ 4JF0 4JF1 4JF3 4JF4 4JF5 4JF6 4JF7 4JF8 4JF9 4JFA 4JFB 4JFC 4JFD 4JFE 4JFF 4JFG 4JFH 4JFI 4JFJ 4JFK 4JFL 4JFM 4JFN 4JFO 4JFP 4JFQ 4JFR 4JFS 4JFT 4JFU 4JFV 4JFW 4JFX 4JFY 4JFZ 4JG0 4JG1 4JG2 4JG3 4JG4 4JG5 4JG6 4JG7 4JG8 4JG9 4JGA 4JGB 4JGD 4JGE 4JGF 4JGG 4JGH 4JGI 4JGJ 4JGK 4JGL 4JGM 4JGO 4JGP 4JGQ 4JGR 4JGS 4JGT 4JGU 4JGV 4JGW 4JGX 4JGY 4JGZ 4JH0 4JH1 4JH2 4JH3 4JH4 4JH5 4JH6 4JH7 4JH8 4JH9 4JHA 4JHB 4JHC 4JHD 4JHG 4JHH 4JHI 4JHJ 4JHK 4JHL 4JHM 4JHN 4JHO 4JHP 4JHQ 4JHR 4JHS 4JHT 4JHU 4JHV 4JHW 4JHX 4JHY 4JHZ 4JI9 4JIA 4JIB 4JIC 4JID 4JIE 4JIF 4JIG 4JIH 4JII 4JIJ 4JIK 4JIM 4JIN 4JIO 4JIP 4JIQ 4JIR 4JIS 4JIT 4JIU 4JIV 4JIW 4JIX 4JIZ 4JJ0 4JJ2 4JJ3 4JJ4 4JJ5 4JJ6 4JJ7 4JJ8 4JJ9 4JJA 4JJB 4JJC 4JJD 4JJE 4JJF 4JJG 4JJH 4JJI 4JJJ 4JJK 4JJM 4JJO 4JJP 4JJQ 4JJR 4JJS 4JJT 4JJU 4JJX 4JJY 4JJZ 4JK1 4JK2 4JK3 4JK4 4JK5 4JK6 4JK7 4JK8 4JK9 4JKA 4JKB 4JKC 4JKD 4JKE 4JKF 4JKG 4JKH 4JKI 4JKJ 4JKK 4JKL 4JKM 4JKN 4JKO 4JKP 4JKQ 4JKR 4JKS 4JKT 4JKU 4JKV 4JKW 4JKX 4JKY 4JKZ 4JL0 4JL1 4JL2 4JL4 4JL5 4JL6 4JL7 4JL8 4JL9 4JLA 4JLB 4JLC 4JLD 4JLE 4JLF 4JLG 4JLH 4JLI 4JLJ 4JLK 4JLL 4JLM 4JLN 4JLO 4JLP 4JLQ 4JLR 4JLS 4JLT 4JLU 4JLV 4JLW 4JLX 4JLY 4JLZ 4JM0 4JM1 4JM2 4JM3 4JM4 4JM5 4JM6 4JM7 4JM8 4JM9 4JMA 4JMB 4JMC 4JMD 4JME 4JMF 4JMG 4JMH 4JMI 4JMJ 4JMK 4JML 4JMN 4JMO 4JMP 4JMQ 4JMR 4JMS 4JMT 4JMU 4JMV 4JMW 4JMX 4JMY 4JMZ 4JN0 4JN1 4JN2 4JN3 4JN4 4JN5 4JN6 4JN7 4JN8 4JN9 4JNA 4JNB 4JNC 4JND 4JNE 4JNF 4JNH 4JNI 4JNJ 4JNK 4JNL 4JNM 4JNN 4JNO 4JNQ 4JNT 4JNU 4JNV 4JNW 4JNY 4JNZ 4JO0 4JO1 4JO2 4JO3 4JO4 4JO5 4JO6 4JO7 4JO8 4JO9 4JOA 4JOB 4JOC 4JOD 4JOE 4JOF 4JOG 4JOH 4JOI 4JOJ 4JOK 4JOL 4JOM 4JON 4JOO 4JOP 4JOQ 4JOR 4JOS 4JOT 4JOU 4JOX 4JP0 4JP1 4JP2 4JP3 4JP4 4JP5 4JP6 4JP7 4JP8 4JP9 4JPA 4JPB 4JPC 4JPD 4JPE 4JPF 4JPG 4JPH 4JPI 4JPJ 4JPK 4JPL 4JPM 4JPN 4JPO 4JPP 4JPQ 4JPR 4JPS 4JPT 4JPU 4JPV 4JPW 4JPX 4JPY 4JPZ 4JQ0 4JQ1 4JQ2 4JQ3 4JQ4 4JQ5 4JQ6 4JQ7 4JQ8 4JQ9 4JQA 4JQC 4JQE 4JQF 4JQG 4JQH 4JQI 4JQJ 4JQK 4JQL 4JQM 4JQN 4JQO 4JQP 4JQR 4JQS 4JQT 4JQU 4JQV 4JQW 4JQX 4JQY 4JQZ 4JR0 4JR1 4JR2 4JR3 4JR4 4JR5 4JR6 4JR7 4JR8 4JR9 4JRA 4JRB 4JRE 4JRF 4JRG 4JRH 4JRI 4JRK 4JRL 4JRM 4JRN 4JRO 4JRR 4JRU 4JRV 4JRW 4JRX 4JRY 4JRZ 4JS0 4JS1 4JS2 4JS3 4JS6 4JS7 4JS8 4JS9 4JSA 4JSB 4JSC 4JSD 4JSE 4JSF 4JSG 4JSH 4JSI 4JSJ 4JSK 4JSL 4JSM 4JSN 4JSO 4JSP 4JSQ 4JSR 4JSS 4JST 4JSU 4JSV 4JSW 4JSX 4JSY 4JSZ 4JT0 4JT2 4JT3 4JT4 4JT5 4JT6 4JT8 4JT9 4JTA 4JTB 4JTC 4JTD 4JTE 4JTF 4JTG 4JTH 4JTI 4JTJ 4JTK 4JTL 4JTM 4JTN 4JTO 4JTP 4JTQ 4JTR 4JTS 4JTT 4JTU 4JTV 4JTW 4JTX 4JTY 4JTZ 4JU0 4JU1 4JU2 4JU3 4JU4 4JU5 4JU6 4JU7 4JU8 4JU9 4JUA 4JUB 4JUC 4JUD 4JUE 4JUF 4JUG 4JUH 4JUI 4JUJ 4JUK 4JUL 4JUM 4JUN 4JUP 4JUQ 4JUR 4JUS 4JUT 4JUU 4JUV 4JUY 4JV3 4JV4 4JV6 4JV7 4JV8 4JV9 4JVA 4JVB 4JVC 4JVD 4JVE 4JVF 4JVG 4JVI 4JVJ 4JVL 4JVM 4JVN 4JVO 4JVP 4JVQ 4JVR 4JVS 4JVT 4JVU 4JVV 4JVW 4JVZ 4JW0 4JW1 4JW2 4JW3 4JWC 4JWD 4JWE 4JWF 4JWG 4JWH 4JWI 4JWJ 4JWK 4JWL 4JWO 4JWP 4JWQ 4JWR 4JWS 4JWT 4JWU 4JWV 4JWX 4JWY 4JX0 4JX1 4JX2 4JX3 4JX4 4JX5 4JX6 4JX7 4JX8 4JX9 4JXB 4JXC 4JXE 4JXF 4JXG 4JXH 4JXI 4JXJ 4JXK 4JXM 4JXN 4JXQ 4JXR 4JXS 4JXT 4JXU 4JXV 4JXW 4JXY 4JY0 4JY1 4JY2 4JY3 4JY4 4JY5 4JY6 4JY7 4JY8 4JY9 4JYB 4JYC 4JYD 4JYE 4JYF 4JYG 4JYH 4JYI 4JYJ 4JYK 4JYL 4JYM 4JYO 4JYP 4JYQ 4JYS 4JYT 4JYU 4JYV 4JYW 4JYX 4JYY 4JZ0 4JZ1 4JZ2 4JZ3 4JZ4 4JZ5 4JZ6 4JZ7 4JZ8 4JZ9 4JZA 4JZB 4JZC 4JZD 4JZE 4JZF 4JZG 4JZI 4JZJ 4JZK 4JZL 4JZN 4JZO 4JZP 4JZQ 4JZR 4JZS 4JZT 4JZW 4JZX 4JZY 4JZZ 4K00 4K02 4K03 4K05 4K06 4K07 4K08 4K09 4K0A 4K0B 4K0C 4K0D 4K0E 4K0G 4K0J 4K0N 4K0O 4K0R 4K0S 4K0T 4K0U 4K0V 4K0W 4K0X 4K0Y 4K0Z 4K10 4K11 4K12 4K13 4K14 4K15 4K17 4K18 4K19 4K1B 4K1C 4K1E 4K1F 4K1H 4K1I 4K1J 4K1K 4K1L 4K1N 4K1O 4K1P 4K1Q 4K1R 4K1S 4K1T 4K1U 4K1V 4K1W 4K1X 4K1Y 4K1Z 4K20 4K21 4K22 4K23 4K24 4K25 4K26 4K28 4K29 4K2A 4K2B 4K2C 4K2D 4K2E 4K2F 4K2G 4K2H 4K2I 4K2J 4K2K 4K2L 4K2M 4K2N 4K2O 4K2P 4K2R 4K2S 4K2U 4K2X 4K2Y 4K2Z 4K30 4K33 4K34 4K35 4K36 4K37 4K38 4K39 4K3A 4K3B 4K3C 4K3D 4K3E 4K3F 4K3G 4K3H 4K3I 4K3J 4K3K 4K3L 4K3M 4K3N 4K3O 4K3P 4K3Q 4K3R 4K3S 4K3U 4K3V 4K3W 4K3X 4K3Y 4K3Z 4K40 4K41 4K42 4K43 4K44 4K45 4K46 4K47 4K48 4K49 4K4A 4K4B 4K4C 4K4D 4K4E 4K4F 4K4J 4K4K 4K4O 4K4P 4K4Q 4K4R 4K51 4K55 4K57 4K59 4K5A 4K5B 4K5C 4K5D 4K5E 4K5F 4K5G 4K5H 4K5I 4K5J 4K5K 4K5L 4K5M 4K5N 4K5O 4K5P 4K5Q 4K5R 4K5S 4K5U 4K5V 4K5W 4K5X 4K5Y 4K5Z 4K60 4K61 4K62 4K63 4K64 4K65 4K66 4K67 4K68 4K69 4K6A 4K6B 4K6C 4K6D 4K6E 4K6F 4K6G 4K6H 4K6I 4K6J 4K6K 4K6L 4K6M 4K6N 4K6O 4K6R 4K6T 4K6U 4K6V 4K6W 4K6X 4K6Y 4K6Z 4K70 4K71 4K72 4K73 4K74 4K75 4K76 4K77 4K78 4K79 4K7A 4K7B 4K7C 4K7D 4K7E 4K7F 4K7G 4K7H 4K7I 4K7J 4K7K 4K7L 4K7M 4K7N 4K7O 4K7P 4K7Q 4K7R 4K7S 4K7T 4K7U 4K7V 4K7W 4K7X 4K7Y 4K7Z 4K80 4K81 4K82 4K83 4K84 4K85 4K86 4K87 4K88 4K89 4K8A 4K8B 4K8C 4K8D 4K8E 4K8F 4K8G 4K8H 4K8I 4K8J 4K8K 4K8L 4
[truncated: 83,183 more chars]
